# Programmed serial stereochemical relay and its application in the synthesis of morphinans†
†Electronic supplementary information (ESI) available. CCDC 1526432. For ESI and crystallographic data in CIF or other electronic format see DOI: 10.1039/c7sc03189k



**DOI:** 10.1039/c7sc03189k

**Published:** 2017-08-30

**Authors:** Kun Ho (Kenny) Park, Rui Chen, David Y.-K. Chen

**Affiliations:** a Department of Chemistry , Seoul National University , Gwanak-1 Gwanak-ro, Gwanak-gu , Seoul 151-742 , South Korea . Email: davidchen@snu.ac.kr

## Abstract

Herein we report a rationally designed, serial point-to-axial and axial-to-point stereoinduction and its integration into multi-step and target-oriented organic synthesis. In this proof-of-concept study, the configurational stability of several carefully designed atropisomeric intermediates and the fidelity of their unconventional stereoinductions were systematically investigated. The highly functionalized prepared synthetic intermediate was further applied in a novel chemical method to access the morphinans and it is potentially applicable to other structurally related alkaloids.

## Abstract 2

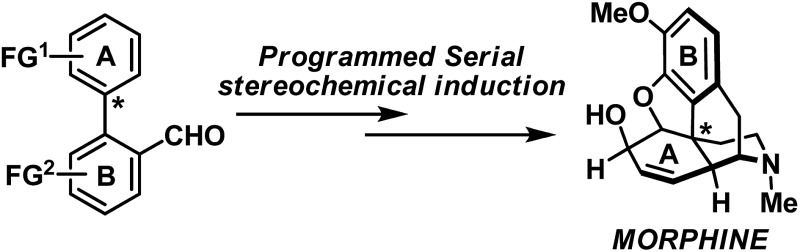
A rationally designed, serial point-to-axial and axial-to-point stereoinduction and its integration into a multi-step and target-oriented organic synthesis was demonstrated in a novel chemical method to access morphinans and it is potentially applicable to other structurally related alkaloids.

## Introduction

**Scheme 1:**
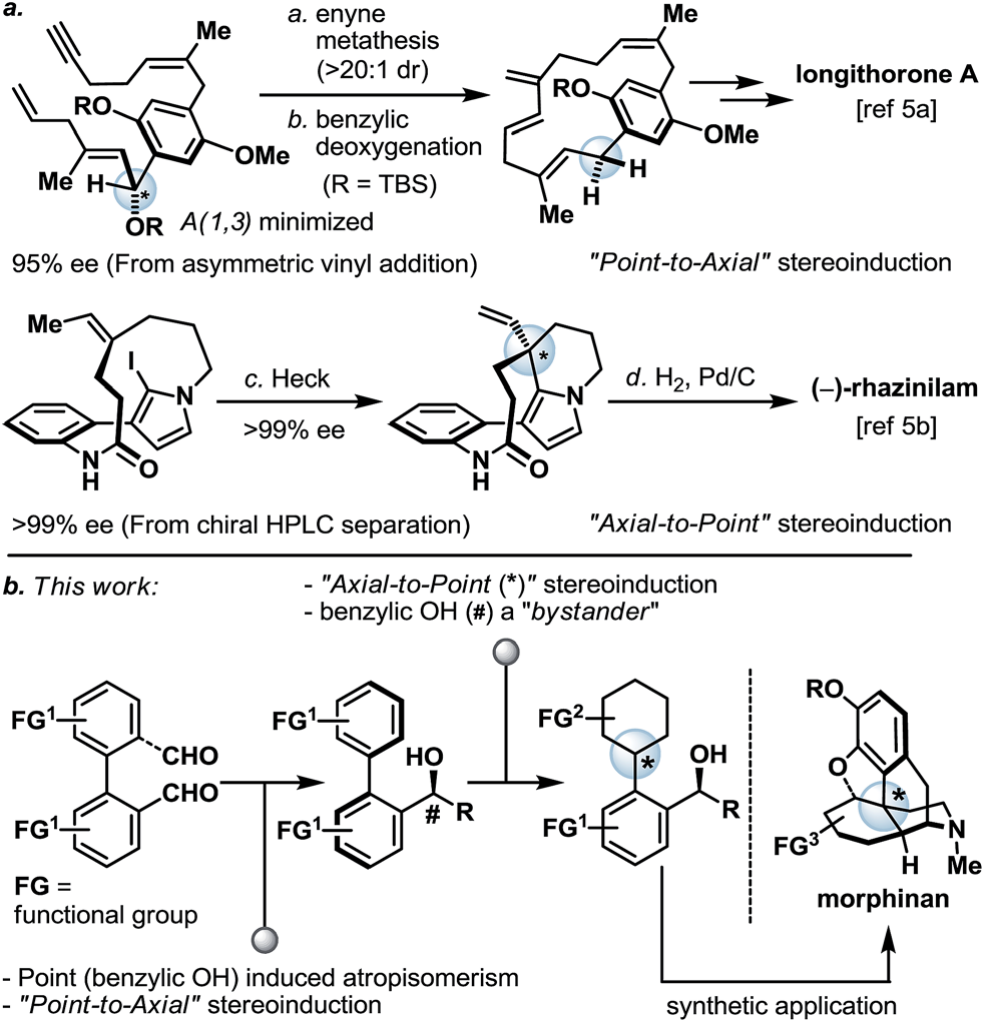
(a) Selected examples of unconventional stereochemical induction in target-oriented organic synthesis; (b) proposed point-to-axial-to-point serial stereochemical relay and its application in the synthesis of morphinans. TBS = *tert*-butyldimethylsilyl.

Thoughtful orchestration of substrate and reagent controlled stereochemical induction is essential in any stereoselective synthesis.^1^ To date, while the fundamental principles behind stereochemical induction are well-grounded, some forms of stereoinduction are much less conventional than others. For example, if we consider “point” and “axial” as two of the most common forms of stereochemical elements, one can appreciate that while the intermolecular point-to-point (for example, oxazaborolidine-mediated reduction)^2^ and axial-to-point (for example, Noyori BINAP hydrogenation)^3^ stereoinductions are routinely practiced, intramolecular point-to-axial, axial-to-point, and axial-to-axial stereoinductions are considerably more rare.^4^ In the asymmetric synthesis of longithorone A^5*a*^ and rhazinilam,^5*b*^ unconventional yet highly effective stereoinductions were elegantly illustrated by the Shair and Zakarian groups, respectively (Scheme 1a). Furthermore, we pondered if a series of these unconventional forms of stereoinduction could be logically sequenced as part of a multi-step synthetic scheme, and in doing so demonstrate their utility in the context of target-oriented synthesis (Scheme 1b).

In preparation for the proposed studies, it is important to recognize that while a “point” stereochemical element can be readily generated and/or identified, an “axial” stereochemical element is only observable if the system can attain a sufficiently high rotational energy barrier. In line with this requirement, we began our proof-of-concept studies by leveraging the well-established biaryl system as the source of the “axial” stereochemical element (Scheme 1b). Furthermore, the proposed biaryl system harbors additional functional group (FG) handles which allow for the subsequent programmed-stereoinduction events.

## Results and discussion

**Scheme 2:**
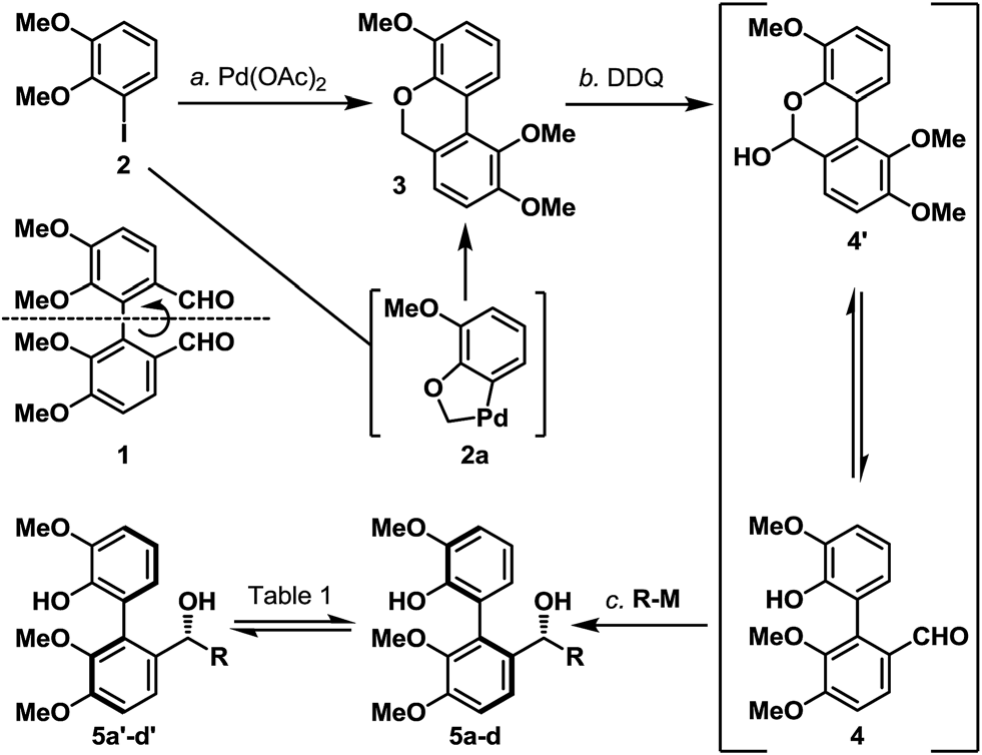
Synthesis of biaryl benzylic alcohols **5a**/**5a′** to **5d**/**5d′** exhibiting atropisomeric properties. Reagents and conditions: (a) Pd(OAc)_2_ (0.04 equiv.), K_2_CO_3_ (4.0 equiv.), TBAB (1.0 equiv.), DMF, 120 °C, 16 h, 86%; (b) DDQ (1.0 equiv.), CH_2_Cl_2_/pH 9.2 buffer (10 : 1), 0 °C, 1.5 h, 76%; (c) for **5a**/**5a′**: methylmagnesium bromide (3.0 M in Et_2_O, 3.3 equiv.), THF, –78 to 23 °C, 16 h, 92% (**5a** : **5a′** ∼ 1 : 1); for **5b**/**5b′**: phenylmagnesium bromide (1.0 M in THF, 4.7 equiv.), THF, –78 to 23 °C, 16 h, 85% (**5b** : **5b′** ∼ 6 : 1); for **5c**/**5c′**: *t*-butyllithium (1.7 M in pentane, 3.5 equiv.), THF, –78 to 23 °C, 16 h, 92% (**5c** : **5c′** ∼ 4 : 1); for **5d**/**5d′**: allylmagnesium bromide (1.0 M in THF, 2.6 equiv.), THF, –78 to 23 °C, 16 h, 97% (**5d** : **5d′** ∼ 3 : 1). DDQ = 2,3-dichloro-5,6-dicyano-1,4-benzoquinone; DMF = *N*,*N*′-dimethylformamide; OAc = acetate; TBAB = tetra-*n*-butylammonium bromide.

We first investigated if a “point” stereochemistry that resides in close proximity to the biaryl axis can render, and hence induce, an axial stereochemical property *i.e.* a point-to-axial stereoinduction. In this context, the synthesis of biaryl aldehyde **4** was originally envisioned based on the desymmetrization of the readily accessible (and potentially optically active) dialdehyde **1** (Scheme 2).^6^ However, a more direct approach was later realized through one of the earliest illustrations of CH-activation chemistry pioneered by Dyker,^7^ and the as-obtained biaryl system **3** underwent smooth benzylic oxidation (DDQ) to afford the targeted aldehyde **4** together with its equilibrating hemiacetal **4′** in 65% yield over the two steps. The phenolic aldehyde **4** (and **4′**) was next subjected to a selection of organometallic reagents, and gratifyingly, each organometallic addition reaction afforded a separable mixture of two stereoisomers in high yield (**5a**/**5a′**–**5d**/**5d′**, 85–97%). Recognizing that the biaryl aldehyde **4** does not have atropisomeric properties at ambient temperature,^8^ this finding indicated that the newly formed biaryls **5a**/**5a′**–**5d**/**5d′** exhibit restricted rotation and constitute an illustration of point-induced atropisomerism.

Before pressing onto our next objective, namely the “axial-to-point” stereochemical induction, two crucial criteria had to be considered. First, the substrate must exhibit sufficient atropisomeric configurational stability during the axial-to-point stereoinduction event. Second, any preparatory transformations leading to the axial-to-point stereoinduction event must preserve the atropisomeric property within the substrate. In order to assess the configurational stability of the organometallic addition products (**5a**/**5a′**–**5d**/**5d′**), chromatographically separated and atropisomerically pure biaryl benzylic alcohols were subjected to thermal conditions and their atropisomerization was monitored by ^1^H NMR analysis (Table 1). Our qualitative analysis revealed that all of the substrates exhibited good configurational stability from low to moderately elevated temperatures (up to 70 °C), with the *t*-butyl substrates **5c** and **5c′** demonstrating extended stability up to 100 °C (for details, see the ESI†).

**Table 1:** Configurational stability study of biaryl benzylic alcohols **5a**/**5a′** to **5d**/**5d′**. Diastereoisomeric pairs: **5a**/**5a′**: R = Me; **5b**/**5b′**: R = Ph; **5c**/**5c′**: R = *t*-Bu; **5d**/**5d′**: R = allyl^*a*^

Compd.	Temp. °C					
	40	70	90	100	110	120
	(^1^H NMR analysis after 1 h at each temperature up to 110 °C)					
**5a**	1 : 0	1 : 0.10	1 : 0.43	1 : 0.88	0.79 : 1	0.69 : 1
**5a′**	0 : 1	0.07 : 1	0.16 : 1	0.39 : 1	0.60 : 1	0.70 : 1
**5b**	1 : 0	1 : 0.13	1 : 0.43	1 : 0.98	0.68 : 1	0.63 : 1
**5b′**	0 : 1	0.06 : 1	0.23 : 1	0.44 : 1	0.58 : 1	0.65 : 1
**5c**	1 : 0	1 : 0	1 : 0	1 : 0.09	1 : 0.35	0.53 : 1
**5c′**	0 : 1	0 : 1	0 : 1	0.06 : 1	0.18 : 1	0.52 : 1
**5d**	1 : 0	1 : 0	1 : 0.36	0.93 : 1	0.71 : 1	0.71 : 1
**5d′**	0 : 1	0 : 1	0.53 : 1	0.70 : 1	0.70 : 1	0.70 : 1

^*a*^Ratio determined by ^1^H NMR integration. For details, see the ESI.†

**Scheme 3:**
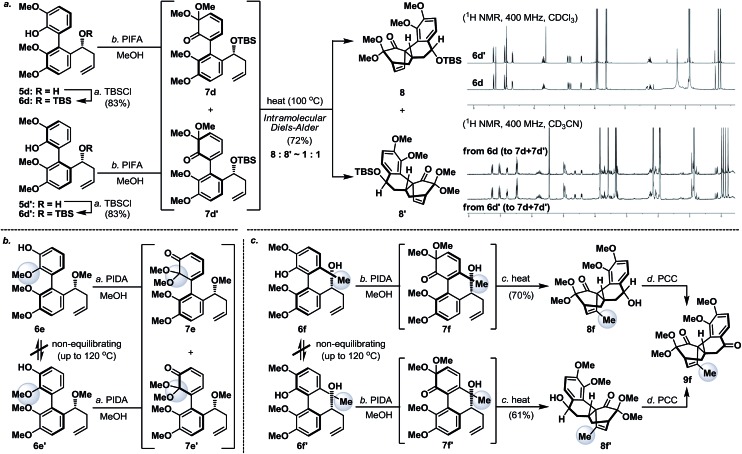
(a) Oxidative dearomatization of atropisomerically pure biaryl phenols **6d** and **6d′**; (b) oxidative dearomatization and configurational stability studies of biaryl phenols **6e** and **6e′**; and (c) oxidative dearomatization and configurational stability studies of biaryl phenols **6f** and **6f′**. PCC = pyridinium chlorochromate; PIDA = [bis(acetoxy)iodo]benzene; PIFA = [bis(trifluoroacetoxy)iodo]benzene; and TBSCl = *tert*-butyldimethylsilyl chloride.

**Scheme 4:**
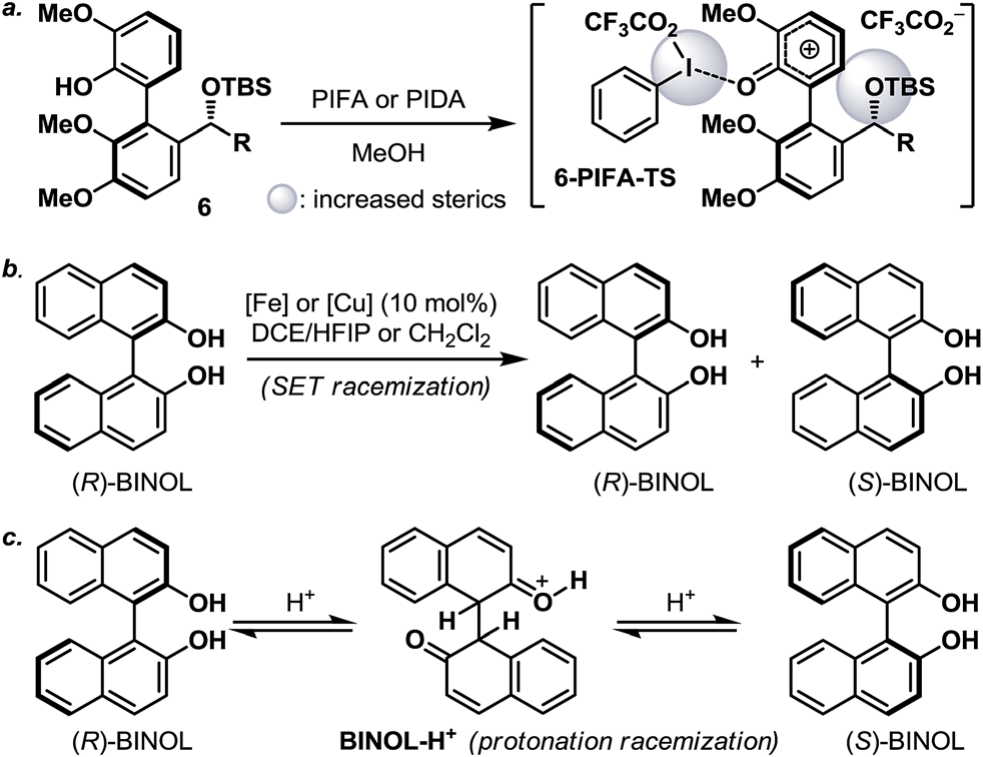
(a) Oxidative dearomatization of **6** *via* an associative mechanism; (b) racemization of (R)-BINOL under SET conditions; and (c) acid promoted racemization of (R)-BINOL. BINOL = 1,1′-bi-2-naphthol; DCE = 1,2-dichloroethane; and HFIP = hexafluoroisopropanol.

Next, we envisaged that the proposed axial-to-point stereoinduction would take place *via* a dearomatization process, in which the dearomatized product or its chemically transformed derivative would possess new “point” stereochemical element(s).^9^ After contemplating a variety of well-documented dearomatization protocols, hypervalent iodine mediated oxidative dearomatization appeared the most appealing owing to its operational ease, substrate scope and product versatility.^10^ Accordingly, benzylic alcohols **5a**/**5a′**–**5d**/**5d′** (except **5c**/**5c′**, which remained as benzylic alcohols) were protected as their corresponding TBS ethers to prevent any unwanted side reactions and to enhance their atropisomeric stabilities, followed by treatment with PIFA in the presence of methanol (**5d**/**5d′** in Scheme 3a, for **5a**/**5a′**–**5d**/**5d′** see the ESI†). Much to our surprise, each of the isomerically pure phenols **6d** and **6d′** underwent oxidative dearomatization to afford an identical mixture of two dienone products **7d** and **7d′** (**7d** : **7d′** ∼ 1 : 1). The fact that **7d** and **7d′** were observed as two distinct diastereoisomers based on our ^1^H NMR analysis suggested that the epimerization of the biaryl axis must take place during the course of the reaction. However, a postulated transition state (**6-PIFA-TS**, Scheme 4a), formed through an associative mechanism by invoking a coordinated phenol–aryliodide complex, should increase the rotational energy barrier and thus render higher configurational stability. While seeking a more concrete explanation, we also became aware of a recent report from the Pappo laboratory describing that several optically pure BINOL substrates underwent metal-catalyzed racemization under single-electron-transfer (SET) conditions (Scheme 4b), although the precise mechanistic origin behind this racemization process remained unclear.^11^ Very recently, NMR and DFT studies reported by the Koltunov group also suggested an acid-mediated atropisomerization of optically pure BINOL *via* several enone intermediates (*e.g.* **BINOL-H^+^**) that structurally closely resemble those generated during the hypervalent iodine and metal–salt mediated phenol oxidations (Scheme 4c).^12^

**Scheme 5:**
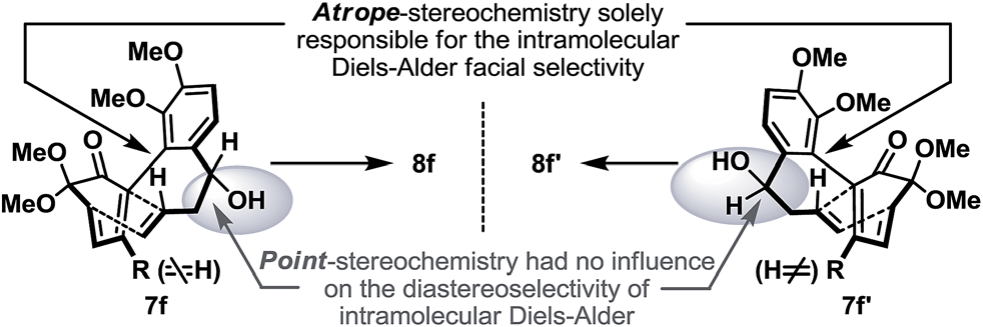
Atropisomerism dictated stereoinduction leading to the stereocontrolled formation of Diels–Alder products **8f** and **8f′**.

Continuing our studies, several related phenolic biaryl substrates were conceived in order to understand and to preserve the atropisomeric information (Scheme 3b and c). In this context, the phenolic substrates **6e** and **6e′** with the phenol and methoxy positions switched compared to those in **6d** and **6d′** demonstrated much improved thermal stability, however, when separately treated with PIDA in the presence of methanol, also afforded an identical mixture of dienones **7e** and **7e′** (∼1 : 1). Gratifyingly, phenols **6f** and **6f′** with an additional methyl substituent were found to retain their atropisomeric purity upon PIDA-mediated oxidative dearomatization, and the atropisomerically pure dienones **7f** and **7f′** faithfully underwent intramolecular Diels–Alder reactions to afford tetracycles **8f** and **8f′** in 70% and 61% yield, respectively, with their stereochemical relationship confirmed upon oxidation with PCC. An analogous intramolecular Diels–Alder reaction could also be realized with a 1 : 1 mixture of dienones **7d** and **7d′** to afford a near 1 : 1 mixture of tetracycles **8** and **8′** (Scheme 3a). Considering that dienones **7d** and **7d′** are likely to be configurationally labile at elevated temperatures (see Table 1), this latter result implied that the benzylic OTBS stereocenter offered essentially no stereoinduction during the intramolecular Diels–Alder process. Furthermore, the selective formation of the Diels–Alder product **8f** from **7f** (and **8f′** from **7f′**) strongly suggested that the stereoinduction arose exclusively from the atropisomeric property of **7f** (and **7f′**) (Scheme 5). In retrospect, the benzylic “point” stereochemical directing element was positioned in proximity to the biaryl-axis for the first “point-to-axial” stereoinduction, while it was distant from the second “axial-to-point” stereoinduction event to suppress its stereo-directing ability. From a design perspective, a single stereo-directing element at each stereochemistry inducing step is synthetically more attractive to avoid any complicated synergistic stereo-directing phenomena.

**Scheme 6:**
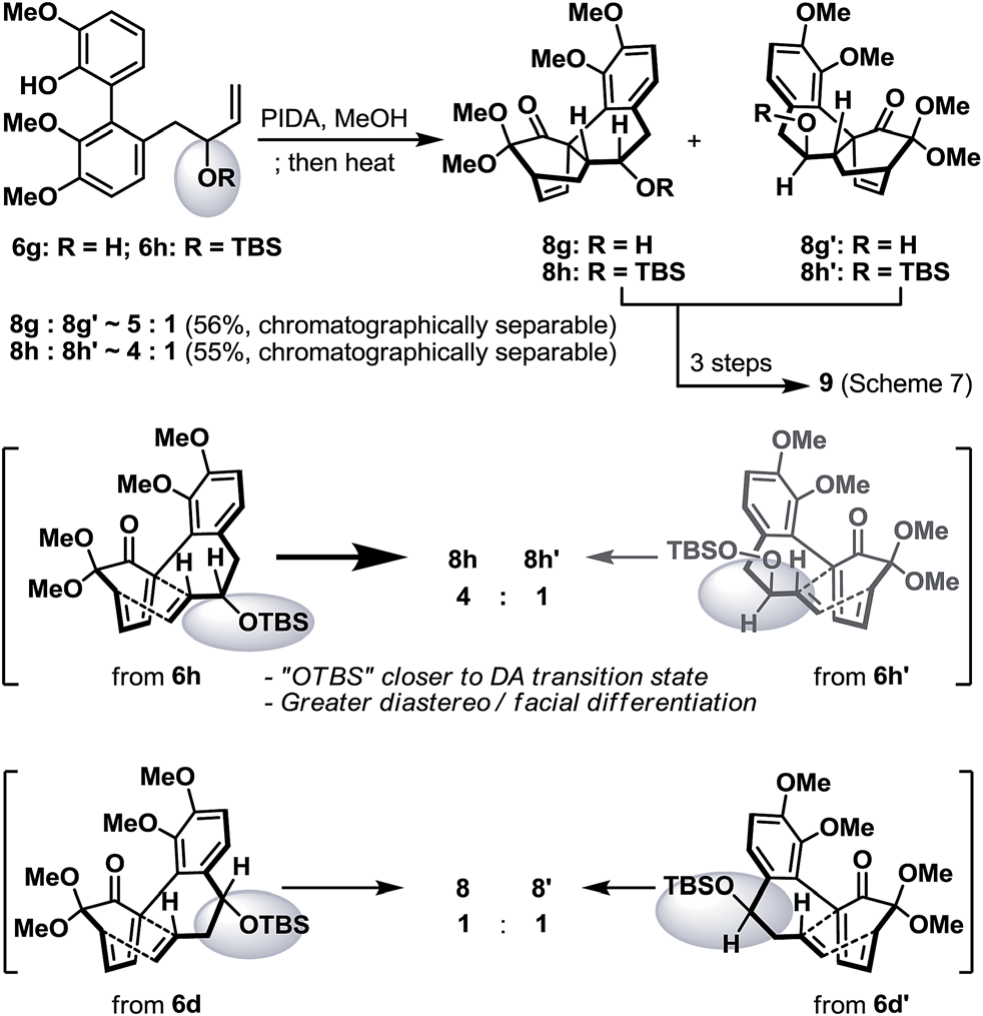
Oxidative dearomatization and intramolecular Diels–Alder reaction of biaryl phenols **6g** and **6h**.

To conclude our studies in remote stereoinductions, we found that substrates **6g** and **6h** (racemic or optically active) with their OH/OTBS stereocenter relocated in proximity to the intramolecular Diels–Alder transition-state could render a significantly higher level of point-to-point stereoinduction than **6d**/**6d′** (Scheme 6), an observation that was consistent with our previous synthetic studies towards platencin.^13^

## Synthetic applications

**Scheme 7:**
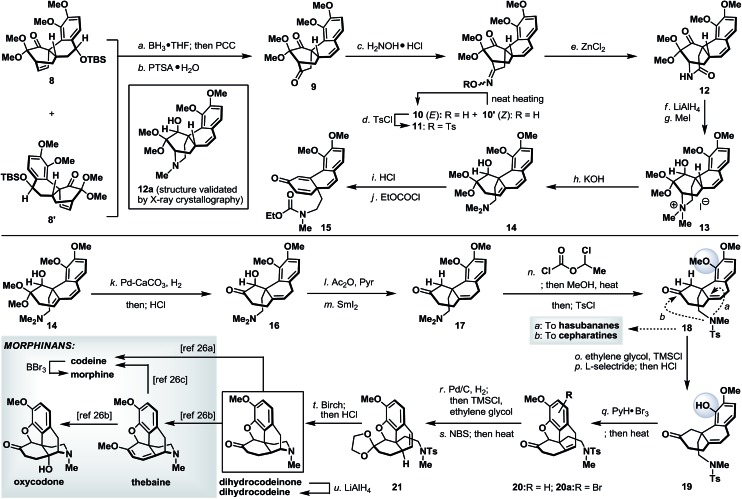
Synthesis of morphinans. Reagents and conditions: (a) BH_3_·THF (5.0 equiv.), THF, –78 to 70 °C, 4 h; then PCC (5.0 equiv.), 23 °C, 5 h, 41%; (b) *p*-TsOH·H_2_O (1.0 equiv.), toluene, 110 °C, 20 min, 95%; (c) H_2_NOH·HCl (3.0 equiv.), NaOAc (3.0 equiv.), MeOH, 23 to 70 °C, 16 h (heat to neat), 90%; (d) TsCl (1.5 equiv.), NaOH (2.5 equiv.), acetone/H_2_O (1 : 1), 23 °C, 2 h, 97%; (e) ZnCl_2_ (2.0 equiv.), MeCN, 100 °C, 4 h; then ZnCl_2_ (2.0 equiv.), 100 °C, 4 h, 73%; (f) LiAlH_4_ (10 equiv.), THF, 0 to 70 °C, 8 h; (g) MeI (14 equiv.), CH_2_Cl_2_, 23 °C, 10 h, 42% for two steps; (h) KOH (20% in MeOH), 70 °C, 10 h, 80%; (i) HCl (10% aq.)/MeOH (4 : 1), 23 °C, 1 h, 67%; (j) EtOCOCl (5.0 equiv.), NaHCO_3_ (20 equiv.), 1,2-dichloroethane, 100 °C, 2 h, 93%; (k) Pd/CaCO_3_ (1.0 equiv.), MeOH, H_2_ (1 atm), 23 °C, 16 h, (1,4 : 1,2-hydrogenated compound ∼ 5 : 1); then HCl (10% aq.)/MeOH (2 : 1), 23 °C, 1 h, 66% for two steps; (l) Ac_2_O (10 equiv.), Et_3_N (10 equiv.), DMAP (0.1 equiv.), CH_2_Cl_2_, 23 °C, 4 h; (m) SmI_2_ (0.1 M in THF), THF/MeOH (1 : 1), –78 °C, 0.5 h, 78% for two steps; (n) 1-chloroethyl chloroformate (20 equiv.), NaHCO_3_ (20 equiv.), 1,2-dichloroethane, 100 °C, 2 h; then MeOH, 60 °C, 1.5 h; then TsCl (2.0 equiv.), DMAP (0.4 equiv.), Et_3_N (2.3 equiv.), CH_2_Cl_2_, 23 °C, 1.5 h, 64% for two steps; (o) TMSCl (2.0 equiv.), ethylene glycol/CH_2_Cl_2_ (1 : 1), 23 to 50 °C, 5 h, 92%; (p) l-selectride (1.0 M in THF, 5.0 equiv.), THF, 80 °C, 24 h; then HCl (4.0 N aq.)/MeOH (1 : 15), 23 to 45 °C, 3 h, 77% for two steps; (q) PyH·Br_3_ (2.0 equiv.), CH_2_Cl_2_/AcOH (2 : 5), 23 °C, 30 min; then LiBr (5.0 equiv.), Et_3_N (10.0 equiv.), MeCN, 60 °C, 20 min, 53%; (r) Pd/C (10% wt/wt, 2.0 equiv.), EtOAc/MeOH (1 : 3), H_2_, 23 °C, 30 min, 90%; then TMSCl (excess), ethylene glycol/CH_2_Cl_2_ (1 : 1), 23 to 50 °C, 5 h, 80%; (s) NBS (1.05 equiv.), benzoyl peroxide (0.5 equiv.), CCl_4_, 80 °C, 1 h; then Et_3_N (7.8 equiv.), 80 °C, 15 min, 60%; (t) Li (excess), NH_3_, –78 °C, 10 min, 69%; then HCl (4.0 N aq.)/MeOH (1 : 15), 70 °C, 6 h, 75%; and (u) LiAlH_4_ (excess), THF, 0 °C, 45 min, 74%. DMAP = *N*,*N*′-dimethylaminopyridine; EtOAc = ethyl acetate; l-selectride = lithium tri-*sec*-butylborohydride; NBS = *N*-bromosuccinimide; PyH·Br_3_ = pyridinium tribromide; TMSCl = trimethylsilyl chloride; *p*-TsOH·H_2_O = *para*-toluenesulfonic acid monohydrate; TsCl = *para*-toluenesulfonyl chloride; and Ts = *para*-toluenesulfonyl.

Finally, we turned our attention to the synthetic utility of the synthesized advanced intermediates. To this end, we recognized that the highly functionalized phenanthrene-type tetracycle **8**/**8′** bearing a quaternary stereocenter shared some similarities with the core structure of the morphinan family of natural products.^14^ Further structural analysis implied that the [2.2.2]-bicyclic domain within **8** (or **8′**) had to be ruptured while retaining its quaternary stereocenter. As shown in Scheme 7, Diels–Alder product **8** (and **8′**) was readily converted to diketone **9** *via* a regioselective hydroboration and an oxidative work-up with PCC,^15^ followed by an acid-induced elimination. It is worth noting that, while tetracycles **8** and **8′** could be used in combination in this racemic illustration, enantio- and diastereo-isomerically pure **8** (or **8′**) will be required to access optically pure intermediates. The more sterically accessible ketone in **9** was converted to its corresponding oxime and thermally equilibrated to a single geometric isomer **10**, which was subsequently tosylated (TsCl, 87% yield from **9**) to set the stage for the Beckmann rearrangement.^16^ Under optimized conditions, tosyloxime **11** underwent regioselective ring expansion under the influence of ZnCl_2_ to afford lactam **12** in 73% yield. Further cleavage of the bridgehead C–N bond in **12** was realized through a Hoffmann elimination^17^ of its quaternary ammonium salt derivative **13** to furnish dimethylamine **14**. Unmasking the dimethoxy acetal in **14** under acidic conditions, very surprisingly, afforded a 1,2-migratory product **15** (after treatment with ethyl chloroformate).^18^ This undesired bond migration was easily rectified upon partial reduction of diene **14**,^19^ followed by acidic deketalization to yield hydroxy ketone **16** in 66% overall yield. α-Deoxygenation and *N*-demethylation were sequentially realized through the action of SmI_2_ and 1-chloroethyl chloroformate and the as-obtained ketone **18** represents a valuable common intermediate that is also amenable for a synthetic method to access the hasubananes (bridgehead cyclization “*a*”).^20^ Advancing tricyclic ketone **18** next called for the formation of a five-membered oxacycle that resides in several flagship morphinans. To this end, phenolic ketone **18** was converted to its dioxolane derivative followed by a dioxolane-directed phenolic demethylation.^22^ Dioxolane removal followed by treatment of the resulting phenolic ketone (**19**) with pyridinium tribromide^23^ and subsequent heating smoothly delivered an inconsequential mixture of oxa-tetracycle **20** and arylbromide **20a**. Next, relocation of the bridgehead olefin^24^ in **20**/**20a** first converged both compounds through hydrogenation and dioxolane formation, and on further exposure to NBS under radical conditions afforded styrene **21**. Reductive detosylation of **21** under Birch-type conditions took place with concomitant piperidine formation^21*s*^ and furnished synthetic dihydrocodeinone upon dioxolane removal (HCl, MeOH).^25^ Dihydrocodeinone serves as a valuable intermediate to readily access a diverse array of naturally occurring and designed morphinans, including but not limited to dihydrocodeine, codeine, morphine, thebaine and oxycodone.^26^

## Conclusions

In summary, we have demonstrated proof-of-concept, rationally designed serial point-to-axial-to-point stereoinductions and examined the stereochemical fidelity of these processes. During this investigation, an unexpected atropisomeric epimerization upon hypervalent iodine-mediated oxidative dearomatization of isomerically pure biaryl phenols was discovered and systematically investigated. This finding bears important ramifications in related oxidative generation^11^ and transformations of designed and naturally occurring phenolic biaryl systems and their atropisomeric integrity. While the undesired atropisomeric epimerization could be suppressed by increasing the steric pressure about the biaryl axis of the substrate (*e.g.* **6f**/**6f′**), more in depth mechanistic studies are in progress to render a substrate-independent solution.^27^ Further application of the developed synthetic strategy also enabled a novel synthetic method to access the morphinan family of natural products and potential access to other related alkaloid structures. Conceptually related programmed serial-stereoinductions, particularly those involving unconventional intramolecular axial-to-point and point-to-axial processes, are currently being designed and implemented in the context of target-oriented synthesis in our laboratory.

## Conflicts of interest

There are no conflicts to declare.
