# Supplementary material for: Programmed serial stereochemical relay and its application in the synthesis of morphinans
Source: Chem Sci. 2017 Aug 30;8(10):7031–7. doi: 10.1039/c7sc03189k (PMC5642196; doi:10.1039/c7sc03189k)

Electronic Supplementary Material (ESI) for Chemical Science.
This journal is © The Royal Society of Chemistry 2017

Programmed Serial Stereochemical Relay and Application in the Synthesis of Morphinans

Kun Ho (Kenny) Park, Rui Chen, and David Y.-K. Chen[*]

Department of Chemistry, Seoul National University

Gwanak-1 Gwanak-ro, Gwanak-gu, Seoul 151-742, South Korea

Email: davidchen@snu.ac.kr

Supporting Information Available

I) Experimental Procedures and Physical Data for Compounds

II) X-ray Crystallographic Analysis of Compound **12a**

III) Abbreviations

IV) References

V) ^{1}H and ^{13}C NMR Spectra for Compounds

I) Experimental Procedures and Physical Data for Compounds

General Procedures. Reactions were carried out under an argon atmosphere with dry solvents under anhydrous conditions, unless otherwise noted. Dry tetrahydrofuran (THF), diethyl ether (Et_2O), methylene chloride (CH_2Cl_2), toluene, *N*,*N*'-dimethylacetamide (DMA), and 1,2-dichloroethane (DCE) were dried and distilled according to the standard protocols. Methanol (MeOH), benzene, *N*,*N*'-dimethylformamide (DMF), and acetonitrile (CH_3CN) were purchased in anhydrous form and used without further purification. Acetone, ethyl acetate (EtOAc), Et_2O, CH_2Cl_2, hexanes and water were purchased at the highest commercial quality and used without further purification, unless otherwise stated. Reagents were purchased at the highest commercial quality and used without further purification, unless otherwise stated. Yields refer to chromatographically and spectroscopically (^{1}H NMR) homogeneous materials, unless otherwise stated. Reactions were monitored by thin-layers

chromatography (TLC) carried out on 0.25 mm E. Merck silica gel plates (60F−254) using UV light as visualizing agent and an ethanolic solution of ammonium molybdate, anisaldehyde or potassium permanganate, and heat as developing agents. E. Merck silica gel (60, particle size 0.040−0.063 mm) was used for flash column chromatography. NMR spectra were recorded on an Agilent 400-MR DD2 Magnetic Resonance System or Varian/Oxford As-500 instrument and calibrated using residue undeuterated solvent as internal reference. The following abbreviations were used to explain the multiplicities: s = singlet, d = doublet, t = triplet, q = quartet, m = multiplet, br = broad. IR spectra were recorded on a Thermo Scientific Nicolet 6700 spectrometer. High-resolution mass spectra (HRMS) were recorded on a Bruker (compact) Ultra High Resolution ESI Q-TOF mass spectrometer.

Iodide 2

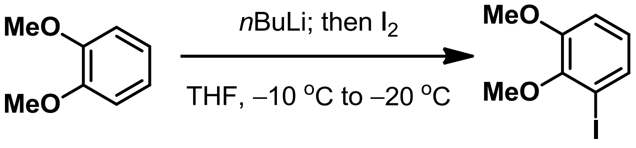

2

To a stirred solution of veratrole (5.00 g, 36.2 mmol) in THF (60 mL) at –10 °C was added *n*BuLi (1.6 M in hexanes, 25.0 mL, 40.0 mmol). The resulting mixture was warmed to room temperature and stirred for 2 h before it was cooled to –45 °C followed by the addition of a cold (–45 °C) solution of I_2 (10.1 g, 39.8 mmol) in THF (100 mL). The resulting mixture was warmed to room temperature and stirred for 2 h before it was quenched with $Na_2S_2O_3$ (100 mL, sat. aq.). The layers were separated and the aqueous layer was extracted with CH_2Cl_2 (3 × 100 mL), the combined organic layer was washed with water (100 mL), brine (100 mL), dried (Na_2SO_4) and concentrated under reduced pressure. Flash column chromatography (silica gel, hexanes:EtOAc 10:1) afforded iodide **2** (7.20 g, 75%) as an amorphous yellow solid. **2**: R_f = 0.75 (silica gel, hexanes:EtOAc 9:1); IR (film) ν_{max} 3269, 2919, 2830, 1415, 625 cm^{-1}; ^{1}H NMR (400 MHz, $CDCl_3$): δ 7.23 (d, *J* = 7.9 Hz, 1H), 6.77 (d, *J* = 8.2 Hz, 1H), 6.71–6.65 (m, 1H), 3.75 (s, 3H), 3.72 ppm (s, 3H); ^{13}C NMR (101 MHz, $CDCl_3$): δ 152.3, 148.2,

129.8, 125.5, 112.3, 92.1, 59.8, 55.4 ppm; HRMS calcd. For $C_8H_9IO_2Na^+$ $[M + Na]^+$ 286.9539, found 286.9537.

Tricycle 3

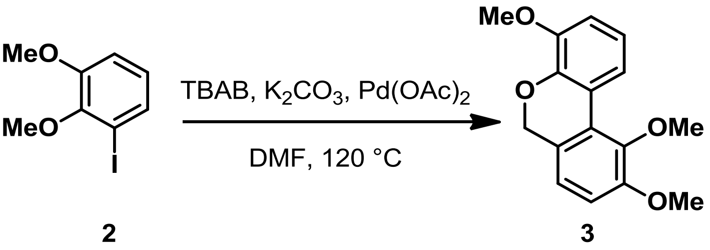

To a stirred solution of iodide **2** (20.6 g, 78.0 mmol) in DMF (190 mL) at room temperature was added K_2CO_3 (43.0 g, 311 mmol), $Pd(OAc)_2$ (0.72 g, 3.21 mmol) and tetra-*n*-butylammonium bromide (25.2 g, 78.2 mmol). The resulting mixture was warmed to 120 °C and stirred for 16 h before it was cooled to room temperature and diluted with water (100 mL). The resulting mixture was extracted with Et_2O (3 × 100 mL), the combined organic layer was washed with water (100 mL), brine (100 mL), dried (Na_2SO_4) and concentrated under reduced pressure. Flash column chromatography (silica gel, hexanes:EtOAc 10:1) afforded tricycle **3** (9.10 g, 86%) as an amorphous white solid. **3**: R_f = 0.46 (silica gel, hexanes:EtOAc 7:3); IR (film) ν_{max} 3154, 2920, 1707, 1342, 1142, 725 cm^{-1}; ^1H NMR (499 MHz, $CDCl_3$): δ 8.07 (d, J = 8.2 Hz, 1H), 6.99 (t, J = 8.1 Hz, 1H), 6.87 (d, J = 8.3 Hz, 2H), 6.81 (d, J = 8.3 Hz, 1H), 4.98 (s, 2H), 3.88 (s, 3H), 3.85 (s, 3H), 3.71 ppm (s, 3H); ^{13}C NMR (126 MHz, $CDCl_3$): δ 153.1, 148.6, 146.3, 144.5, 126.0, 123.3, 122.5, 121.3, 120.0, 120.0, 111.4, 111.1, 68.7, 59.7, 55.8, 55.8 ppm; HRMS calcd. For $C_{16}H_{16}O_4Na^+$ $[M + Na]^+$ 295.0941, found 295.0940.

Bromide 2a

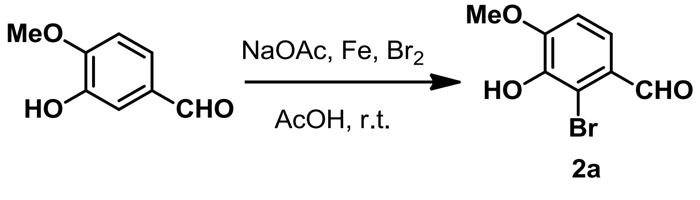

To a stirred solution of isovanillin (30.0 g, 197 mmol), NaOAc (32.3 g, 394 mmol) and iron powder (0.90 g, 16.1 mmol) in AcOH (glacial, 180 mL) at room temperature was added a solution of bromine/AcOH (1:4.2, 55.4 mL, 208 mmol). The resulting mixture was stirred for 16 h before it was quenched with ice cold water (500 mL). The resulting precipitate was filtered, washed with ice cold water (4 × 70 mL) and air dried. Recrystallization of the crude material from boiling ethanol afforded bromide **2a** (34.5 g, 76%) as a gray powder. **2a**: R_f = 0.40 (silica gel, CH_2Cl_2); IR (film) ν_{max} 3667, 2985, 1738, 1265, 1057, 736 cm^{-1}; ^1H NMR (499 MHz, $CDCl_3$): δ 10.26 (s, 1H), 7.58 (d, *J* = 8.5 Hz, 1H), 6.92 (d, *J* = 8.6 Hz, 1H), 4.00 ppm (s, 3H); ^{13}C NMR (101 MHz, DMSO): δ 191.1, 153.5, 144.2, 126.8, 122.3, 113.5, 110.5, 56.6 ppm; HRMS calcd. For $C_8H_7BrO_3$ $[M]^+$ 228.9579, found 228.9467.

Dimethyl Ether 2b

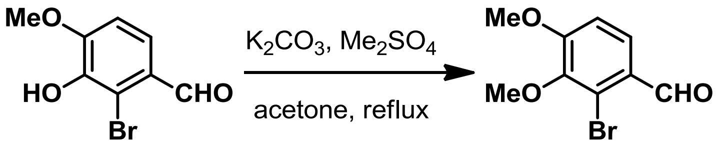

2a 2b

To a stirred solution of phenol **2a** (81.0 g, 351 mmol) in acetone (820 mL) at room temperature was added K_2CO_3 (81.0 g, 586 mmol) and dimethylsulfate (56.0 mL, 592 mmol). The resulting mixture was warmed to reflux and stirred for 16 h before it was cooled to room temperature, and evaporated to approximately half of its original volume. The resulting mixture was diluted with water (200 mL) and extracted with Et_2O (3 × 100 mL), the combined organic layer was dried (Na_2SO_4) and concentrated under reduced pressure to afford dimethyl ether **2b** (74.7 g, 87%) as a white solid. The crude material was sufficiently pure based on thin-layer-chromatography and ^1H NMR analysis for the subsequent reaction. **2b**: R_f = 0.48 (silica gel, CH_2Cl_2); IR (film) ν_{max} 3674, 2985, 2901, 1679, 1280, 779 cm^{-1}; ^1H NMR (499 MHz, $CDCl_3$): δ 10.22 (s, 1H), 7.71 (d, *J* = 8.7 Hz, 1H), 6.94 (d, *J* = 8.7 Hz, 1H), 3.94 (s, 3H), 3.86 ppm (s, 3H); ^{13}C NMR (126 MHz, $CDCl_3$): δ 190.8, 158.6, 146.3, 127.3, 126.4, 123.0, 110.9, 60.6, 56.2 ppm; HRMS calcd. For $C_9H_9BrO_3Na^+$ $[M + Na]^+$ 266.9627, found 266.9625.

Alcohol 2c

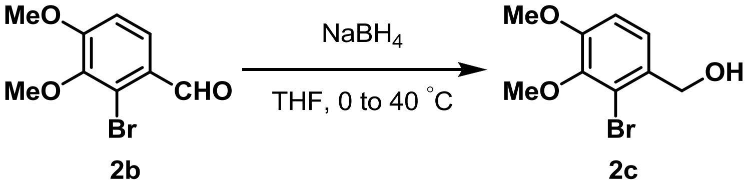

To a stirred solution of aldehyde **2b** (33.1 g, 135 mmol) in THF (250 mL) at 0 °C was added $NaBH_4$ (13.0 g, 344 mmol) in portions. The resulting mixture was warmed to 40 °C and stirred for 2 h before it was cooled to room temperature and quenched with water (200 mL). The layers were separated and the aqueous layer was extracted with Et_2O (3 × 100 mL), the combined organic layer was dried (Na_2SO_4) and concentrated under reduced pressure to afford alcohol **2c** (28.5 g, 85%) as a white solid. The crude material was sufficiently pure based on thin-layer-chromatography and ^1H NMR analysis for the subsequent reaction. **2c**: R_f = 0.10 (silica gel, CH_2Cl_2); IR (film) ν_{max} 3232, 1667, 1284, 1016, 818 cm^{-1}; ^1H NMR (499 MHz, $CDCl_3$): δ 7.14 (d, J = 8.4 Hz, 1H), 6.84 (d, J = 8.4 Hz, 1H), 4.66 (d, J = 5.7 Hz, 2H), 3.86 (s, 3H), 3.84 (s, 3H), 2.34 ppm (t, J = 6.0 Hz, 1H); ^{13}C NMR (126 MHz, $CDCl_3$): δ 152.9, 146.3, 132.7, 124.1, 118.3, 111.1, 64.9, 60.4, 56.0 ppm; HRMS calcd. For $C_9H_{11}BrO_3Na^+$ [M + Na]$^+$ 268.9784, found 268.9782.

Dibromide 2d

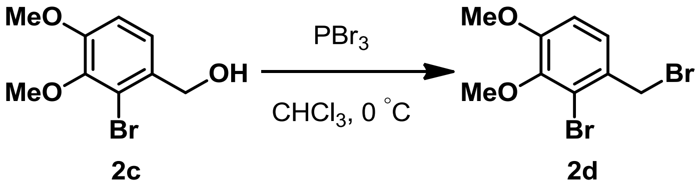

To a stirred solution of alcohol **2c** (27.7 g, 112 mmol) in $CHCl_3$ (52.2 mL) at 0 °C was added PBr_3 (8.52 mL, 89.8 mmol). The resulting mixture was stirred for 3 h before it was quenched with water (100 mL). The layers were separated and the aqueous layer was extracted with CH_2Cl_2 (3 × 100 mL), the combined organic layers were dried (Na_2SO_4) and concentrated under reduced pressure to afford dibromide **2d** (33.0 g, 95%) as a white solid. The crude material was sufficiently pure based on thin-layer-chromatography and ^1H NMR analysis for

the subsequent reaction. **2d**: R_f = 0.84 (silica gel, CH_2Cl_2); IR (film) ν_{max} 2985, 2901, 1486, 1302, 810, 660 cm^{-1}; ^1H NMR (499 MHz, $CDCl_3$): δ 7.19 (d, *J* = 8.5 Hz, 1H), 6.84 (d, *J* = 8.5 Hz, 1H), 4.62 (s, 2H), 3.87 (s, 3H), 3.85 ppm (s, 3H); ^{13}C NMR (126 MHz, $CDCl_3$): δ 153.7, 146.9, 129.8, 126.3, 120.4, 111.2, 60.4, 56.0, 34.3 ppm; HRMS calcd. For $C_9H_{10}Br_2O_2Na^+$ [M + Na]$^+$ 330.8940, found 330.8937.

Biaryl Ether 2e

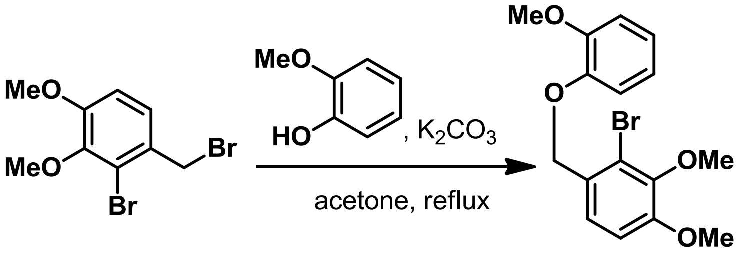

2d **2e**

To a stirred solution of dibromide **2d** (31.0 g, 100 mmol) in acetone (232 mL) at room temperature was added K_2CO_3 (27.7 g, 200 mmol) and guaiacol (9.57 mL, 86.3 mmol). The resulting mixture was warmed to reflux and stirred for 16 h before it was cooled to room temperature and diluted with water (100 mL). The resulting mixture was extracted with Et_2O (3 × 100 mL), the combined organic layer was dried (Na_2SO_4) and concentrated under reduced pressure. Flash column chromatography (silica gel, hexanes:Et_2O 5:1) afforded biaryl ether **2e** (30.6 g, 87%) as an amorphous white solid. **2e**: R_f = 0.13 (silica gel, hexanes:Et_2O 4:1); IR (film) ν_{max} 3001, 2941, 1271, 1146, 806, 752 cm^{-1}; ^1H NMR (499 MHz, $CDCl_3$): δ 7.28 (d, *J* = 8.7 Hz, 1H), 6.97–6.84 (m, 5H), 5.16 (s, 2H), 3.91 (s, 3H), 3.88 (s, 3H), 3.84 ppm (s, 3H); ^{13}C NMR (101 MHz, $CDCl_3$): δ 152.9, 149.5, 147.8, 146.2, 129.0, 123.9, 121.5, 120.7, 117.8, 114.1, 111.9, 111.1, 70.2, 60.3, 55.9, 55.8 ppm; HRMS calcd. For $C_{16}H_{17}BrO_4Na^+$ [M + Na]$^+$ 375.0202, found 375.0204.

Tricycle 3:

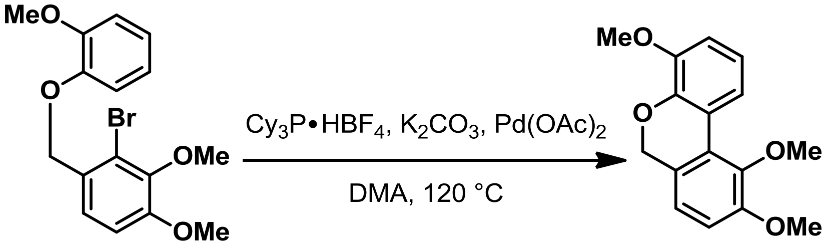

To a stirred solution of bromide **2e** (18.9 g, 53.5 mmol) in DMA (freshly distilled, 190 mL) at room temperature was added K_2CO_3 (14.9 g, 108 mmol), $Pd(OAc)_2$ (0.37 g, 1.65 mmol) and $Cy_3P\text{-}HBF_4$ (1.18 g, 3.20 mmol). The resulting mixture was warmed to 120 °C and stirred for 16 h before it was cooled to room temperature and diluted with water (100 mL). The resulting mixture was extracted with Et_2O (3 × 100 mL), the combined organic layer was washed with water (100 mL), brine (100 mL), dried (Na_2SO_4) and concentrated under reduced pressure. Flash column chromatography (silica gel, hexanes:EtOAc 10:1) afforded tricycle **3** (12.6 g, 86%) as a white solid. All physical data of tricycle **3** are identical to those obtained from the $Pd(OAc)_2$ mediated annulation of iodide **2**.

Hydroxy Aldehyde 4 and Hemiacetal 4′

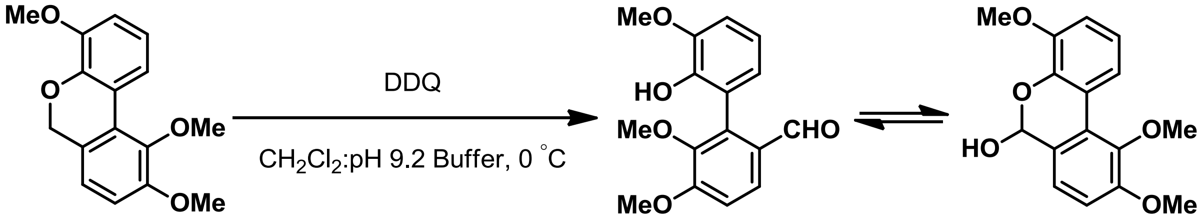

To a stirred solution of tricycle **3** (19.8 g, 72.7 mmol) in CH_2Cl_2/pH 9.2 buffer (10:1, 1.2 L) at 0 °C was added DDQ (16.5 g, 72.7 mmol) portionwise. The resulting mixture was stirred for 1.5 h before the layers were separated and the aqueous layer was extracted with CH_2Cl_2 (3 × 150 mL). The combined organic layer was washed successively with water (until the aqueous layer became colorless), brine (100 mL), dried (Na_2SO_4) and concentrated under reduced pressure. Recrystallization of the crude material from CH_2Cl_2/hexane afforded hydroxy aldehyde **4** and hemiacetal **4′** (15.9 g, 76%) as a pale amorphous yellow solid. ^{1}H NMR

analysis indicated a mixture of hydroxy aldehyde **4** and hemiacetal **4′** (**4**:**4′**~1:3 by ^{1}H NMR analysis). **4+4′**: R_f = 0.33 (silica gel, hexanes:EtOAc 1:1); IR (film) ν_{max} 3408, 2987, 1739, 1651, 1266, 737 cm^{-1}; ^{1}H NMR (499 MHz, $CDCl_3$, **4**:**4′**~1:3): δ 9.57 (s, 0.25H), 8.21 (d, *J* = 8.2 Hz, 0.75H), 7.84 (d, *J* = 8.7 Hz, 0.25H), 7.13 (d, *J* = 8.3 Hz, 0.75H), 7.10–7.03 (m, 1.25H), 6.98–6.92 (m, 1.75H), 6.82 (dd, *J* = 5.1, 4.1 Hz, 0.25H), 6.38 (d, *J* = 6.2 Hz, 0.75H), 5.80 (s, 0.25H), 3.97 (s, 0.75H), 3.94 (s, 0.75H), 3.92 (s, 2.25H), 3.91 (s, 2.25H), 3.78 (s, 2.25H), 3.62 (s, 0.75H), 3.47 ppm (d, *J* = 6.2 Hz, 0.75H); ^{13}C NMR (101 MHz, DMSO): δ 153.4, 149.6, 145.6, 141.0, 127.1, 121.7, 121.3, 121.1, 119.2, 112.3, 112.2, 92.0, 59.6, 56.1, 56.0, 55.8 ppm; HRMS calcd. For $C_{16}H_{16}O_5Na^+$ $[M + Na]^+$ 311.0890, found 311.0889.

Alcohols 5a and 5a′

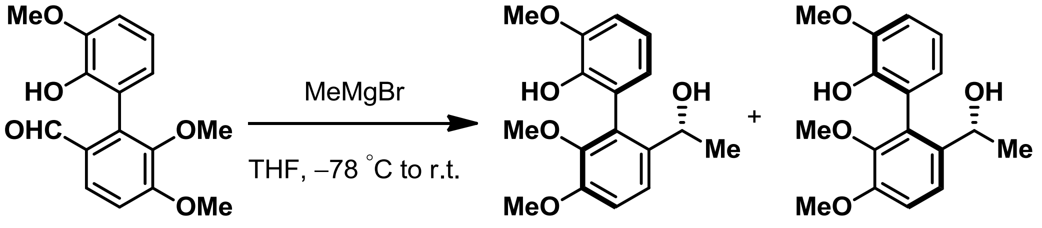

4 (mixture with hemiacetal **4'**) **5a** **5a'**

To a stirred solution of hemiacetal-hydroxy aldehyde mixture (**4+4′**, 213 mg, 0.74 mmol) in THF (15.0 mL) at –78 °C was added methylmagnesium bromide (3.0 M in Et_2O, 0.81 mL, 2.43 mmol) dropwise. The resulting mixture was warmed to room temperature and stirred for 16 h before it was quenched with water (20 mL). The layers were separated and the aqueous layer was extracted with Et_2O (3 × 30 mL), the combined organic layer was washed with water (20 mL), brine (20 mL), dried (Na_2SO_4) and concentrated under reduced pressure. Flash column chromatography (silica gel, hexanes:EtOAc 4:1) afforded alcohols **5a** and **5a′** (~1:1 based on ^{1}H NMR analysis, 207 mg, 92% combined yield) as an amorphous solid. Small amount of analytically pure isomers **5a** and **5a′** were obtained through column chromatography.

5a (relative stereochemistry arbitrarily assigned): R_f = 0.18 (silica gel, hexanes:EtOAc 1:1); IR (film) ν_{max} 3456, 3154, 2918, 1705, 1197, 726 cm^{-1}; ^{1}H NMR (400 MHz, C_6D_6): δ 7.48 (d, *J* = 9.3 Hz, 1H), 6.85 (d, *J* = 7.0 Hz, 1H), 6.76 (t, *J* = 9.2 Hz, 1H), 6.70 (d, *J* = 8.1 Hz, 1H),

6.43 (d, J = 8.1 Hz, 1H), 5.63 (br, 1H), 4.87 (q, J = 5.8 Hz, 1H), 3.63 (s, 3H), 3.36 (s, 3H), 3.14 (s, 3H), 1.46 ppm (d, J = 6.1 Hz, 3H); ^{13}C NMR (101 MHz, $CDCl_3$): δ 152.1, 146.5, 146.3, 142.5, 137.2, 130.5, 123.8, 122.6, 120.8, 119.7, 112.3, 109.8, 66.5, 60.6, 55.9, 55.8, 22.9 ppm; HRMS calcd. For $C_{17}H_{20}O_5Na^+$ $[M + Na]^+$ 327.1203, found 327.1204.

5a′ (relative stereochemistry arbitrarily assigned): R_f = 0.25 (silica gel, hexanes:EtOAc 1:1); IR (film) ν_{max} 3457, 3154, 2920, 1706, 1200, 726 cm^{-1}; ^{1}H NMR (400 MHz, C_6D_6): δ 7.44 (d, J = 8.3 Hz, 1H), 6.85 (d, J = 8.3 Hz, 1H), 6.76 (t, J = 8.3 Hz, 1H), 6.71 (d, J = 8.3 Hz, 1H), 6.45 (d, J = 8.3 Hz, 1H), 5.53 (s, 1H), 4.83 (q, J = 6.5 Hz, 1H), 3.70 (s, 3H), 3.37 (s, 3H), 3.14 (s, 3H), 1.35 ppm (d, J = 6.2 Hz, 3H); ^{13}C NMR (101 MHz, $CDCl_3$): δ 151.8, 146.6, 146.4, 143.2, 137.4, 130.3, 123.0, 122.5, 120.8, 119.5, 112.3, 109.9, 66.8, 60.5, 55.8, 55.8, 24.0 ppm; HRMS calcd. For $C_{17}H_{20}O_5Na^+$ $[M + Na]^+$ 327.1203, found 327.1203.

Alcohols 5b and 5b′

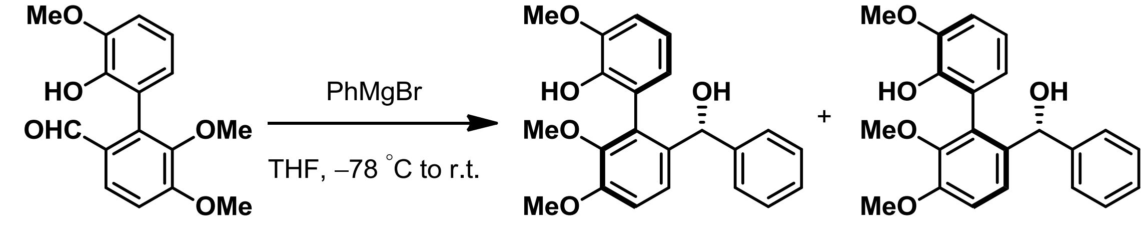

4 (mixture with hemiacetal **4'**) **5b** **5b'**

To a stirred solution of hemiacetal-hydroxy aldehyde mixture (**4+4′**, 215 mg, 0.75 mmol) in THF (15.0 mL) at –78 °C was added phenylmagnesium bromide (1.0 M in THF, 3.50 mL, 3.50 mmol) dropwise. The resulting mixture was warmed to room temperature and stirred for 16 h before it was quenched with water (20 mL). The layers were separated and the aqueous layer was extracted with Et_2O (3 × 30 mL), the combined organic layer was washed with water (20 mL), brine (20 mL), dried (Na_2SO_4) and concentrated under reduced pressure. Flash column chromatography (silica gel, hexanes:EtOAc 9:1) afforded alcohols **5b** and **5b′** (~6:1 based on ^{1}H NMR analysis, 233 mg, 85% combined yield) as an amorphous solid. Small amount of analytically pure isomers **5b** and **5b′** were obtained through column chromatography.

5b (relative stereochemistry arbitrarily assigned): R_f = 0.32 (silica gel, hexanes:EtOAc 1:1); IR (film) ν_{max} 3666, 3154, 2933, 1705, 1341, 1140, 727 cm^{-1}; ^{1}H NMR (499 MHz, C_6D_6): δ 7.55 (d, *J* = 7.8 Hz, 2H), 7.19–7.13 (m, 3H), 7.08 (t, *J* = 7.3 Hz, 1H), 6.92 (d, *J* = 7.7 Hz, 1H), 6.74 (t, *J* = 7.9 Hz, 1H), 6.55 (d, *J* = 8.7 Hz, 1H), 6.40 (d, *J* = 8.1 Hz, 1H), 5.93 (s, 1H), 5.60 (s, 1H), 3.63 (s, 3H), 3.27 (s, 3H), 3.12 (s, 3H), 3.04 ppm (s, 1H); ^{13}C NMR (126 MHz, C_6D_6): δ 152.9, 147.3, 147.0, 144.7, 143.6, 137.5, 132.2, 128.2, 126.9, 126.9, 124.6, 124.2, 123.8, 120.0, 112.9, 110.1, 72.9, 60.4, 55.4, 55.3 ppm; HRMS calcd. For $C_{22}H_{22}O_5Na^+$ [M + Na]$^+$ 389.1359, found 389.1357.

5b′ (relative stereochemistry arbitrarily assigned): R_f = 0.45 (silica gel, hexanes:EtOAc 1:1); IR (film) ν_{max} 3622, 3234, 2857, 1680, 1391, 1162, 811 cm^{-1}; ^{1}H NMR (400 MHz, C_6D_6): δ 7.42 (d, *J* = 7.6 Hz, 2H), 7.13–7.08 (m, 3H), 6.99 (t, *J* = 7.5 Hz, 1H), 6.78 (d, *J* = 7.7 Hz, 1H), 6.67 (t, *J* = 7.9 Hz, 1H), 6.50 (d, *J* = 8.6 Hz, 1H), 6.37 (d, *J* = 8.1 Hz, 1H), 5.76 (s, 1H), 5.40 (s, 1H), 3.62 (s, 3H), 3.23 (s, 3H), 3.07 ppm (s, 3H); ^{13}C NMR (101 MHz, C_6D_6): δ 152.7, 147.7, 147.0, 145.1, 144.2, 137.2, 132.5, 127.0, 126.8, 126.8, 124.1, 123.9, 123.8, 119.5, 112.6, 110.1, 73.0, 60.4, 55.4, 55.3 ppm; HRMS calcd. For $C_{22}H_{22}O_5Na^+$ [M + Na]$^+$ 389.1359, found 389.1359.

Alcohols 5c and 5c′

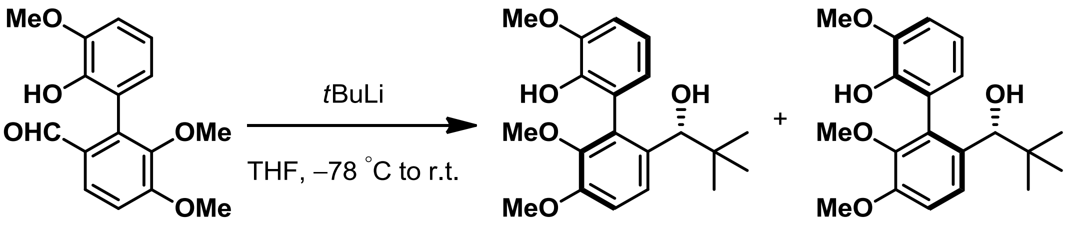

4 (mixture with hemiacetal **4'**) **5c** **5c'**

To a stirred solution of hemiacetal-hydroxy aldehyde mixture (**4+4′**, 210 mg, 0.73 mmol) in THF (15.0 mL) at −78 °C was added *tert*-butyllithium (1.7 M in pentane, 1.50 mL, 2.55 mmol). The resulting mixture was warmed to room temperature and stirred for 16 h before it was quenched with water (20 mL). The layers were separated and the aqueous layer was extracted with Et_2O (3 × 30 mL), the combined organic layer was washed with water (30 mL), brine (30 mL), dried (Na_2SO_4), and concentrated under reduced pressure. Flash column

chromatography (hexanes:EtOAc 10:1) afforded alcohols **5c** and **5c′** (~4:1 based on ^{1}H NMR analysis, 231 mg, 92% combined yield) as an amorphous solid. Small amount of analytically pure isomers **5c** and **5c′** were obtained through column chromatography.

5c (relative stereochemistry arbitrarily assigned): R_f = 0.38 (silica gel, hexanes:EtOAc 1:1); IR (film) ν_{max} 3464, 2915, 1705, 1198, 1062, 721 cm^{-1}; ^{1}H NMR (499 MHz, $CDCl_3$): δ 7.37 (d, *J* = 8.7 Hz, 1H), 6.99 (d, *J* = 8.7 Hz, 1H), 6.92–6.85 (m, 2H), 6.77 (dd, *J* = 7.2, 2.0 Hz, 1H), 5.7 (br s, 1H), 4.25 (s, 1H), 3.93 (s, 3H), 3.90 (s, 3H), 3.48 (s, 3H), 0.78 ppm (s, 9H); ^{13}C NMR (101 MHz, $CDCl_3$): δ 152.0, 146.1, 145.9, 142.2, 134.9, 131.6, 125.3, 123.4, 122.8, 119.2, 111.5, 109.5, 77.6, 60.4, 55.9, 55.7, 36.2, 26.4 ppm; HRMS calcd. For $C_{20}H_{26}O_5Na^+$ $[M + Na]^+$ 369.1672, found 369.1671.

5c′ (relative stereochemistry arbitrarily assigned): R_f = 0.55 (silica gel, hexanes:EtOAc 1:1); IR (film) ν_{max} 3456, 2920, 1704, 1204, 1050, 731 cm^{-1}; ^{1}H NMR (499 MHz, $CDCl_3$): δ 7.36 (d, *J* = 8.7 Hz, 1H), 6.99 (d, *J* = 8.7 Hz, 1H), 6.89 (d, *J* = 5.1 Hz, 2H), 6.73 (t, *J* = 4.4 Hz, 1H), 5.67 (s, 1H), 4.49 (s, 1H), 3.92 (s, 3H), 3.90 (s, 3H), 3.57 (s, 3H), 0.75 ppm (s, 9H); ^{13}C NMR (101 MHz, $CDCl_3$): δ 151.5, 147.3, 145.9, 143.9, 134.0, 132.2, 123.8, 123.6, 123.1, 119.3, 111.5, 110.0, 77.1, 60.6, 55.9, 55.7, 36.1, 25.9 ppm; HRMS calcd. For $C_{20}H_{26}O_5Na^+$ $[M + Na]^+$ 369.1672, found 369.1674.

Alcohols 5d and 5d′

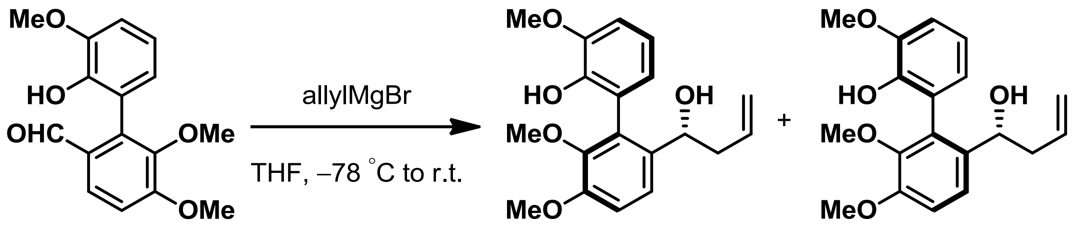

4 (mixture with hemiacetal **4'**) **5d** **5d'**

To a stirred solution of hemiacetal-hydroxy aldehyde mixture (**4+4′**, 1.80 g, 6.24 mmol) in THF (77.0 mL) at –78 °C was added allylmagnesium bromide (1.0 M in THF, 16.0 mL, 16.0 mmol) dropwise. The resulting mixture was warmed to room temperature and stirred for 16 h before it was quenched with water (100 mL). The layers were separated and the aqueous layer was extracted with Et_2O (3 × 150 mL), the combined organic layer was washed with water

(100 mL), brine (100 mL), dried (Na_2SO_4) and concentrated under reduced pressure. Flash column chromatography (silica gel, hexanes:EtOAc 9:1) afforded alcohols **5d** and **5d′** (~3:1 based on ^{1}H NMR analysis, 2.01 g, 97% combined yield) as an amorphous solid. Small amount of analytically pure isomers **5d** and **5d′** were obtained through column chromatography.

5d (relative stereochemistry arbitrarily assigned): R_f = 0.24 (silica gel, hexanes:EtOAc 1:1); IR (film) ν_{max} 3454, 3154, 2925, 1574, 1196, 722 cm^{-1}; ^{1}H NMR (499 MHz, $CDCl_3$): δ 7.33 (d, *J* = 10.0 Hz, 1H), 7.01 (d, *J* = 9.7 Hz, 1H), 6.94–6.88 (m, 2H), 6.72 (dd, *J* = 7.1, 2.1 Hz, 1H), 5.82 (s, 1H), 5.67–5.59 (m, 1H), 5.00–4.95 (m, 2H), 4.43 (t, *J* = 7.1 Hz, 1H), 3.93 (s, 3H), 3.90 (s, 3H), 3.56 (s, 3H), 2.52 (s, 1H), 2.50–2.44 (m, 1H), 2.42–2.36 ppm (m, 1H); ^{13}C NMR (101 MHz, $CDCl_3$): δ 151.6, 146.6, 146.3, 143.1, 135.4, 135.2, 130.4, 122.8, 122.4, 121.3, 119.3, 117.3, 111.9, 109.8, 69.8, 60.4, 55.7, 55.6, 42.4 ppm; HRMS calcd. For $C_{19}H_{22}O_5Na^+$ $[M + Na]^+$ 353.1359, found 353.1357.

5d′ (relative stereochemistry arbitrarily assigned): R_f = 0.37 (silica gel, hexanes:EtOAc 1:1); IR (film) ν_{max} 3539, 3154, 2931, 1600, 1208, 721 cm^{-1}; ^{1}H NMR (400 MHz, $CDCl_3$): δ 7.29 (d, *J* = 8.6 Hz, 1H), 6.97 (d, *J* = 8.7 Hz, 1H), 6.93–6.84 (m, 2H), 6.70 (dd, *J* = 7.3, 1.8 Hz, 1H), 6.16 (s, 1H), 5.70–5.63 (m, 1H), 4.98–4.91 (m, 2H), 4.40 (t, *J* = 7.8 Hz, 1H), 3.87 (s, 3H), 3.86 (s, 3H), 3.55 (s, 3H), 2.97 (br s, 1H), 2.50–2.32 ppm (m, 2H); ^{13}C NMR (101 MHz, $CDCl_3$): δ 151.8, 146.4, 146.1, 142.2, 135.5, 134.9, 130.7, 123.7, 122.4, 121.3, 119.5, 116.7, 112.0, 109.7, 69.8, 60.3, 55.6, 55.6, 41.5 ppm; HRMS calcd. For $C_{19}H_{22}O_5Na^+$ $[M + Na]^+$ 353.1359, found 353.1360.

Atropisomer Thermal Stability Studies:

NMR samples of atropisomerically pure (obtained through silica-gel flash column chromatography and purity confirmed by ^{1}H NMR analysis) compounds **5a**, **5a′**, **5b**, **5b′**, **5c**, **5c′**, **5d** and **5d′** in C_6D_6 were subjected to heating (oil bath) and held at temperatures 40 ℃, 70 ℃, 90 ℃, 100 ℃, 110 ℃, 120 ℃ for 1 hour intervals. The samples were then cooled to room temperature after each incremental heating, and degree of atropisomerization analyzed by ^{1}H NMR analysis. Finally, all samples were further heated at 120 ℃ until atropisomeric ratio remained constant.

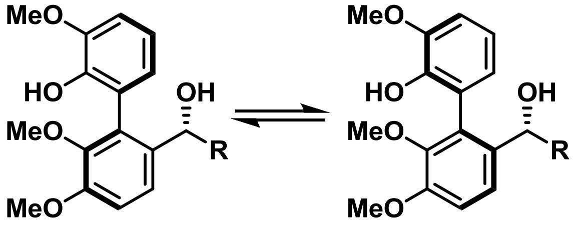

5a, 5a': R = methyl
5b, 5b': R = Ph
5c, 5c': R = ^{t}Bu
5d, 5d': R = allyl

Compd. \ Temp. °C	40	70	90	100	110	120
	Temperature-dependent configurational stability study[a]					
**5a**	1:0	1:0.10	1:0.43	1:0.88	0.79:1	0.69:1
**5a'**	0:1	0.07:1	0.16:1	0.39:1	0.60:1	0.70:1
**5b**	1:0	1:0.13	1:0.43	1:0.98	0.68:1	0.63:1
**5b'**	0:1	0.06:1	0.23:1	0.44:1	0.58:1	0.65:1
**5c**	1:0	1:0	1:0	1:0.09	1:0.35	0.53:1
**5c'**	0:1	0:1	0:1	0.06:1	0.18:1	0.52:1
**5d**	1:0	1:0	1:0.36	0.93:1	0.71:1	0.71:1
**5d'**	0:1	0:1	0.53:1	0.70:1	0.70:1	0.70:1

[a]*ratio of each atropisomeric pair indicated*

^{1}H NMR (400 MHz, C_6D_6)

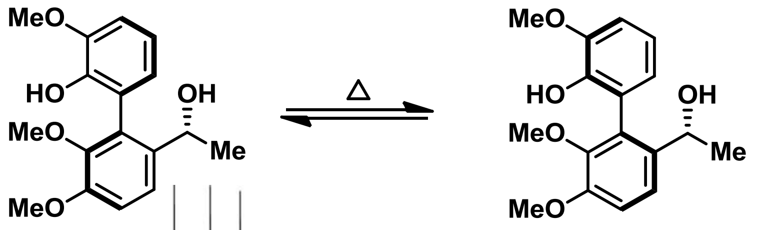

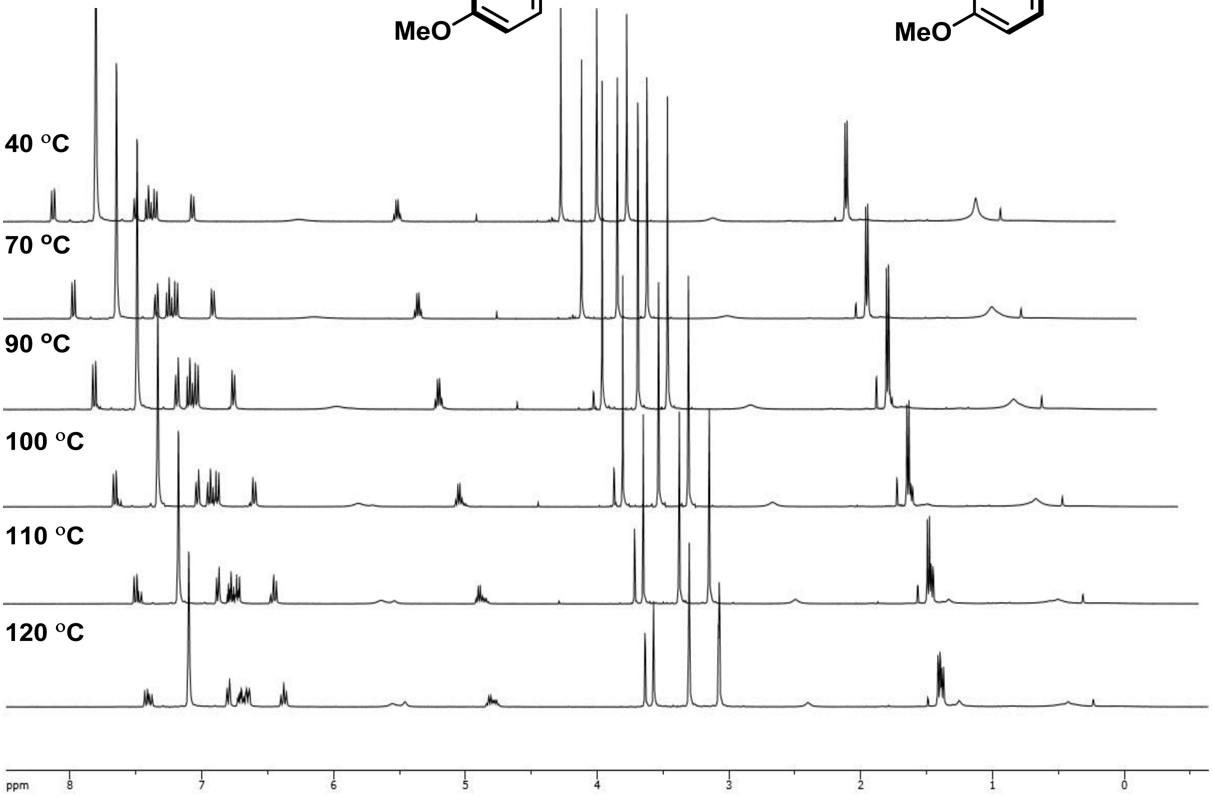

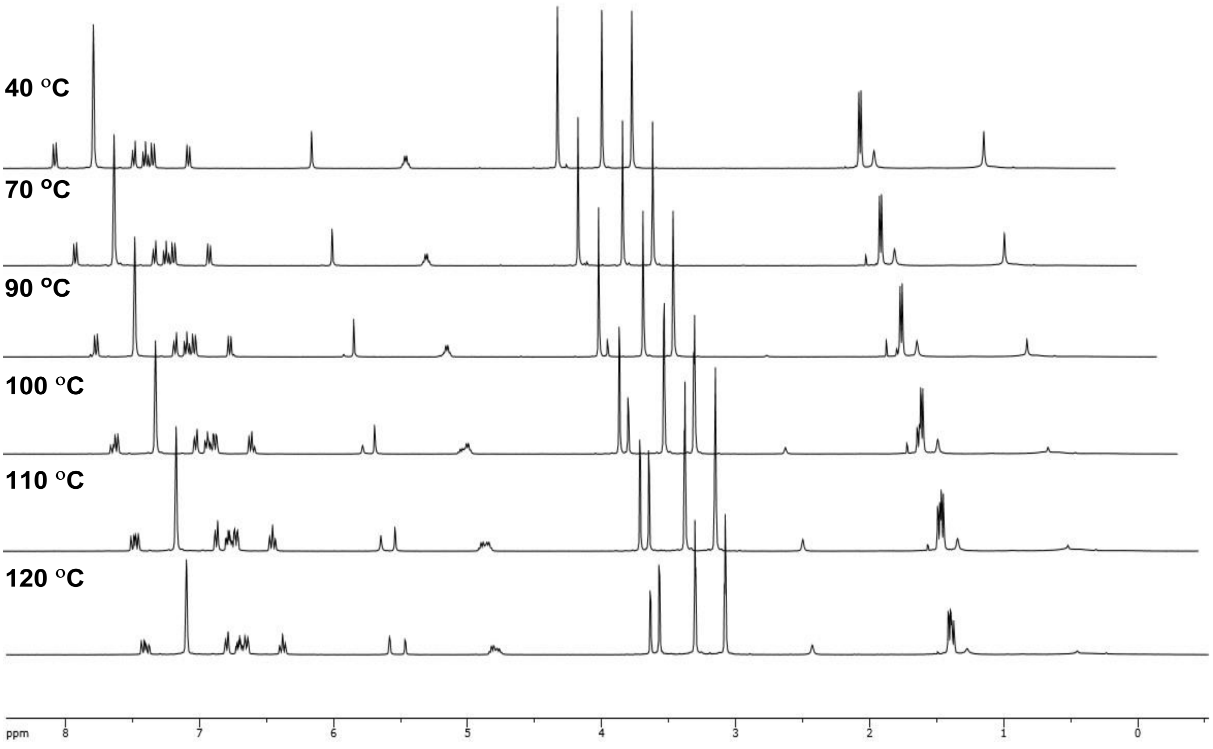

^1H NMR (400 MHz, C_6D_6)

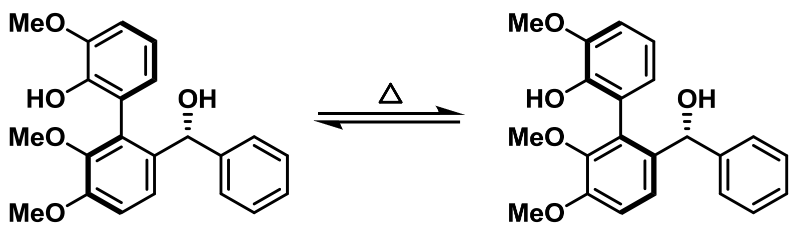

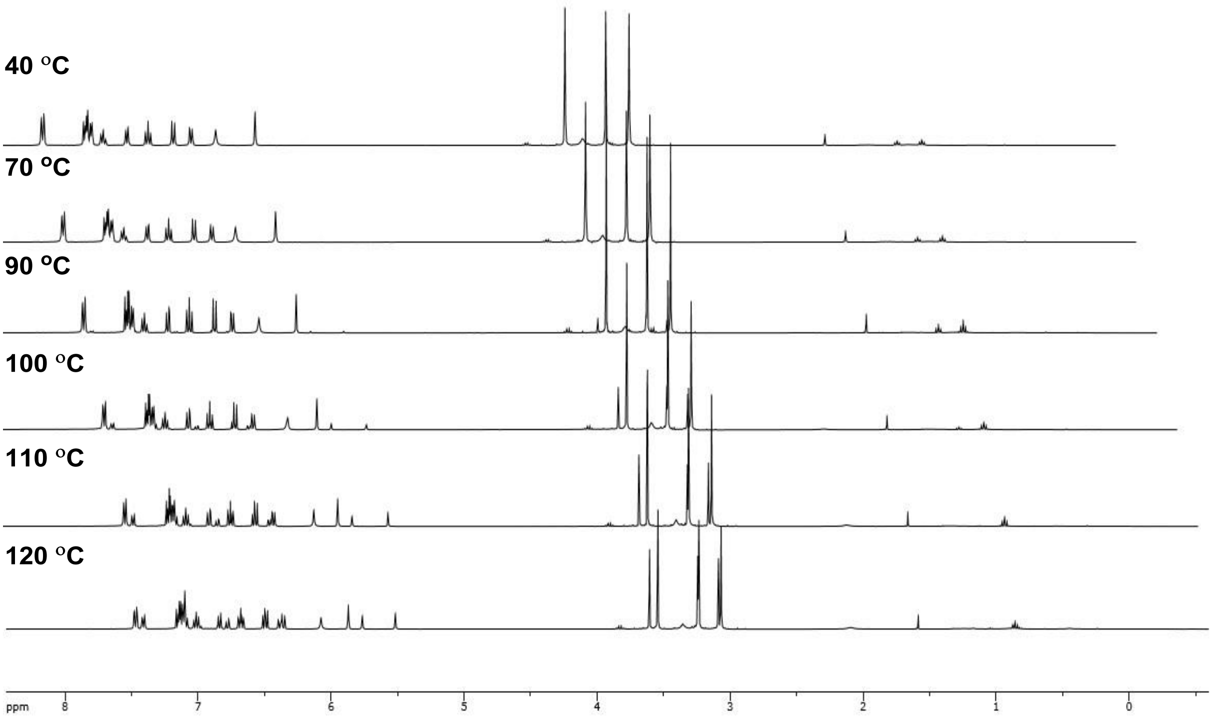

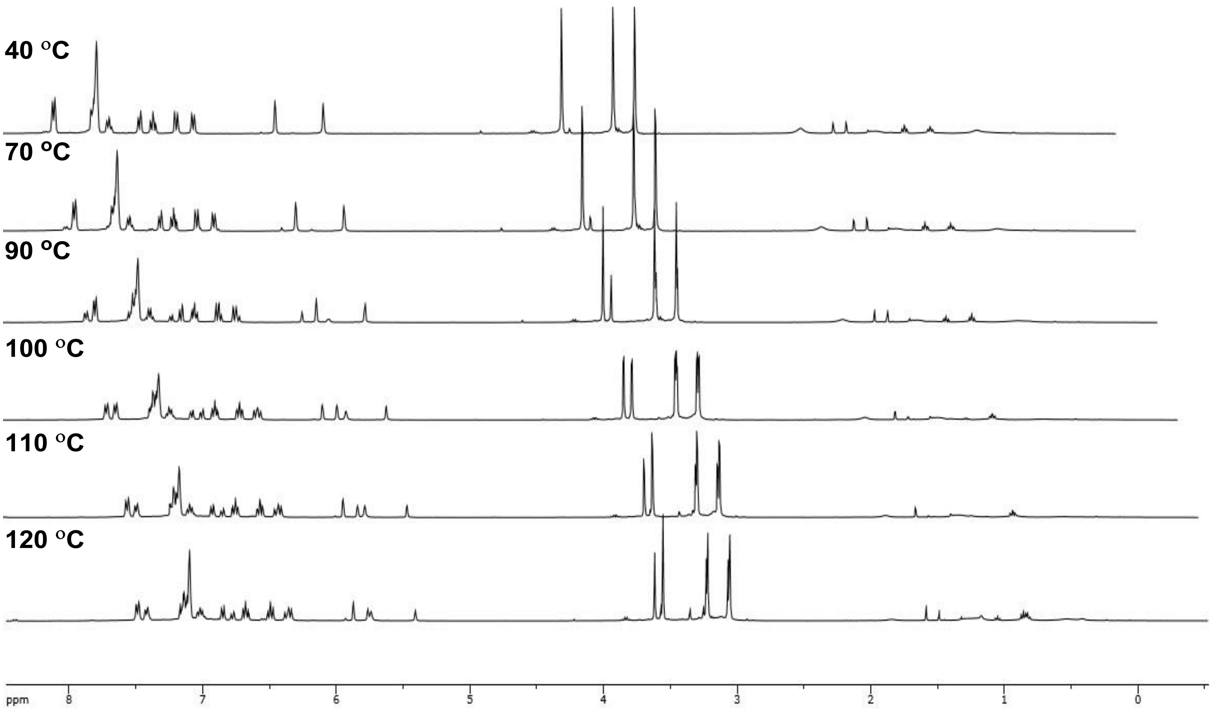

^{1}H NMR (400 MHz, C_6D_6)

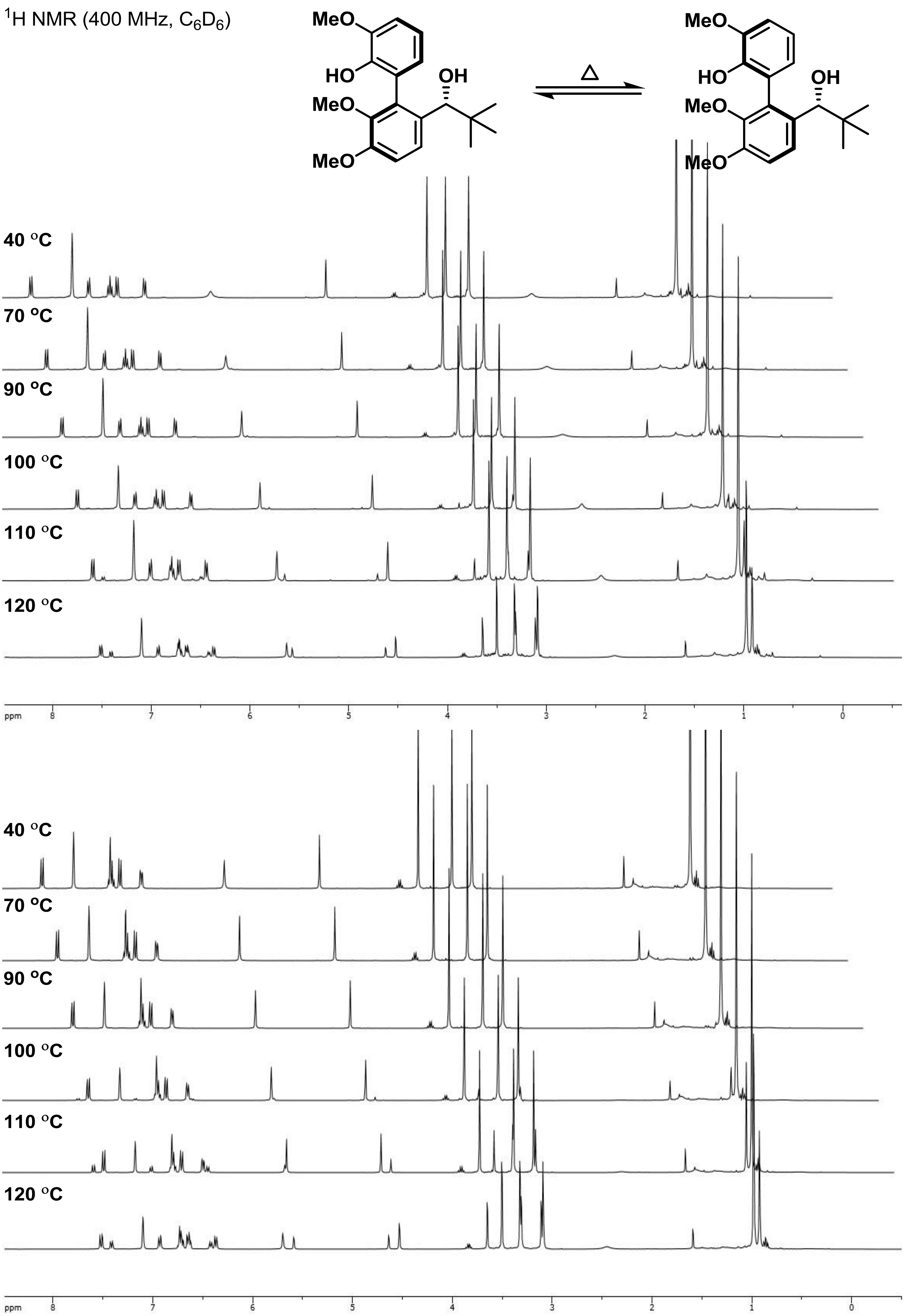

^{1}H NMR (400 MHz, C_6D_6)

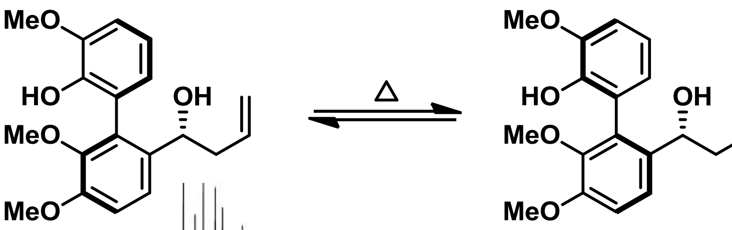

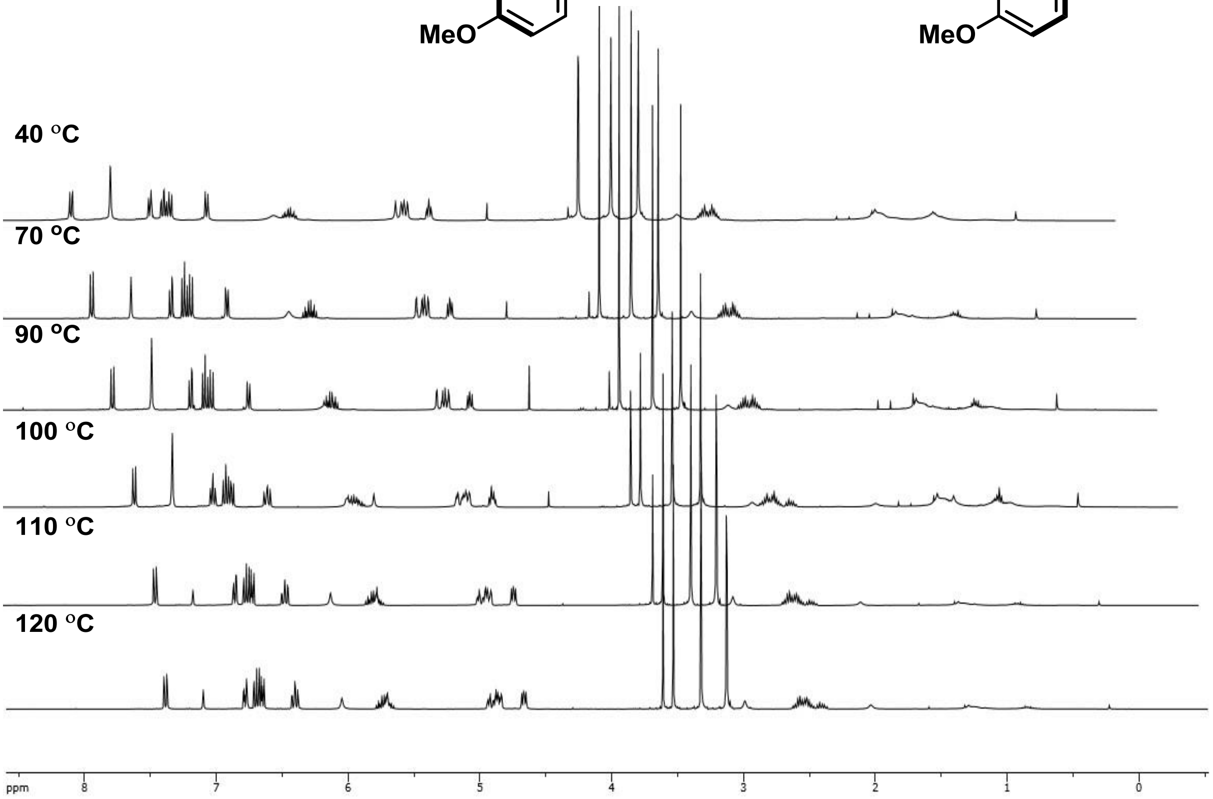

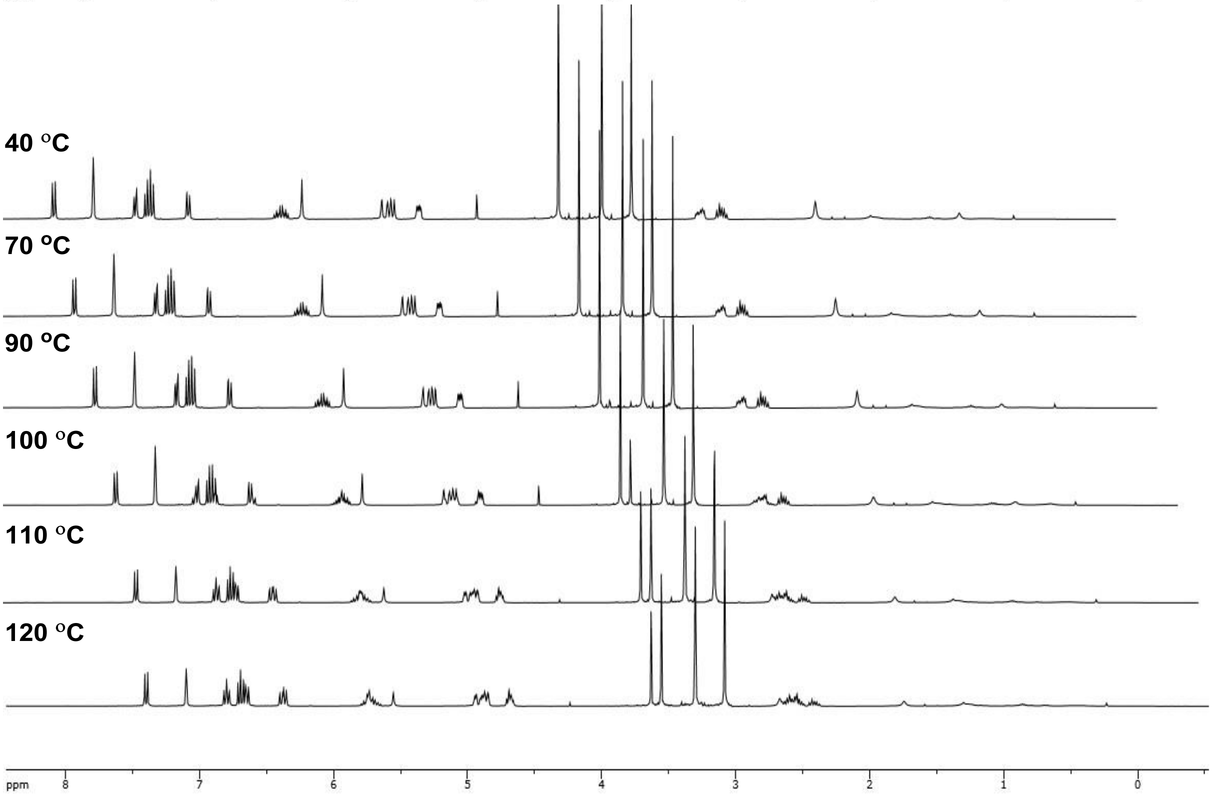

TBS Ethers 6a and 6a′

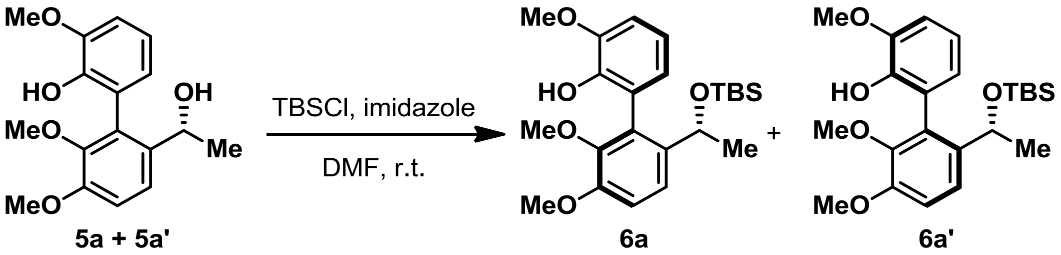

To a stirred solution of alcohol **5a** and **5a′** (335 mg, 1.10 mmol) in DMF (1.50 mL) at room temperature was added TBSCl (664 mg, 4.41 mmol) and imidazole (300 mg, 4.41 mmol). The resulting mixture was stirred for 24 h before it was quenched with NH_4Cl (40 mL, sat. aq.) and water (40 mL). The resulting mixture was extracted with ethyl acetate (3 × 100 mL), the combined organic layer was washed with water (50 mL), brine (50 mL), dried (Na_2SO_4) and concentrated under reduced pressure. Flash column chromatography (silica gel, hexanes:EtOAc 12:1) afforded TBS ethers **6a** and **6a′** (382 mg, 83% combined yield) as an amorphous solid. Small amount of analytically pure isomers **6a** and **6a′** were obtained through column chromatography.

6a (relative stereochemistry arbitrarily assigned): R_f = 0.46 (silica gel, hexanes:EtOAc 4:1); IR (film) ν_{max} 3530, 3054, 2856, 1600, 1551, 733 cm^{-1}; ^1H NMR (499 MHz, $CDCl_3$): δ 7.39 (d, *J* = 8.6 Hz, 1H), 6.99 (d, *J* = 8.6 Hz, 1H), 6.90–6.82 (m, 2H), 6.72–6.69 (m, 1H), 5.59 (s, 1H), 4.59 (q, *J* = 6.2 Hz, 1H), 3.94 (s, 3H), 3.89 (s, 3H), 3.61 (s, 3H), 1.18 (d, *J* = 6.2 Hz, 3H), 0.85 (s, 9H), –0.09 (s, 3H), –0.12 ppm (s, 3H); ^{13}C NMR (101 MHz, $CDCl_3$): δ 151.1, 146.7, 145.9, 143.4, 138.9, 128.9, 122.9, 122.8, 121.2, 119.3, 112.2, 109.8, 67.5, 60.5, 55.8, 55.7, 26.6, 25.8, 18.1, –4.7, –4.9 ppm; HRMS calcd. For $C_{23}H_{34}O_5SiNa^+$ $[M + Na]^+$ 441.2068, found 441.2069.

6a′ (relative stereochemistry arbitrarily assigned): R_f = 0.48 (silica gel, hexanes:EtOAc 4:1); IR (film) ν_{max} 3541, 3154, 2856, 1642, 1560, 742 cm^{-1}; ^1H NMR (499 MHz, $CDCl_3$): δ 7.39 (d, *J* = 8.7 Hz, 1H), 6.98 (d, *J* = 8.7 Hz, 1H), 6.89–6.79 (m, 2H), 6.73–6.69 (m, 1H), 5.57 (s, 1H), 4.55 (q, *J* = 6.2 Hz, 1H), 3.94 (s, 3H), 3.89 (s, 3H), 3.58 (s, 3H), 1.15 (d, *J* = 6.2 Hz, 3H), 0.86 (s, 9H), –0.08 (s, 3H), –0.12 ppm (s, 3H); ^{13}C NMR (101 MHz, $CDCl_3$): δ 151.1, 146.7, 145.9, 142.6, 139.2, 128.8, 124.2, 122.6, 120.9, 119.1, 111.9, 109.9, 67.4, 60.6, 56.0, 55.7,

27.2, 25.9, 25.6, 18.0, −3.6, −4.9, −5.2 ppm; HRMS calcd. For $C_{23}H_{34}O_5SiNa^+$ [M + Na]$^+$ 441.2068, found 441.2067.

TBS Ethers 6b and 6b′

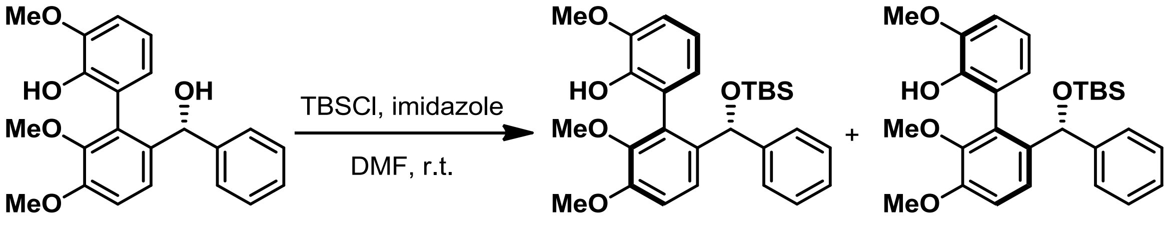

5b + 5b'   6b   6b'

To a stirred solution of alcohols **5b** and **5b′** (160 mg, 0.44 mmol) in DMF (4.00 mL) at room temperature was added TBSCl (263 mg, 1.74 mmol) and imidazole (119 mg, 1.75 mmol). The resulting mixture was stirred for 24 h before it was quenched with NH_4Cl (40 mL, sat. aq.) and water (40 mL). The resulting mixture was extracted with ethyl acetate (3 × 100 mL), the combined organic layer was washed with water (50 mL), brine (50 mL), dried (Na_2SO_4), and concentrated under reduced pressure. Flash column chromatography (silica gel, hexanes:EtOAc 12:1) afforded TBS ethers **6b** and **6b′** (168 mg, 80% combined yield) as an amorphous solid. Small amount of analytically pure isomers **6b** and **6b′** were obtained through column chromatography.

6b (relative stereochemistry arbitrarily assigned): R_f = 0.20 (silica gel, hexanes:EtOAc 4:1); IR (film) ν_{max} 3533, 3038, 2857, 1572, 1458, 725 cm^{-1}; ^{1}H NMR (400 MHz, C_6D_6): δ 7.72 (d, *J* = 8.6 Hz, 1H), 7.35 (d, *J* = 7.8 Hz, 2H), 7.12 (t, *J* = 7.0 Hz, 2H), 7.02 (t, *J* = 6.7 Hz, 2H), 6.83 (t, *J* = 7.9 Hz, 1H), 6.70 (d, *J* = 8.6 Hz, 1H), 6.48 (d, *J* = 8.1 Hz, 1H), 5.96 (s, 1H), 5.14 (s, 1H), 3.70 (s, 3H), 3.31 (s, 3H), 3.14 (s, 3H), 1.03 (s, 9H), 0.07 (s, 3H), −0.03 ppm (s, 3H); ^{13}C NMR (101 MHz, C_6D_6): δ 152.3, 147.5, 147.3, 145.6, 144.9, 137.3, 131.1, 127.9, 127.5, 126.9, 123.9, 123.8, 122.6, 119.4, 112.7, 110.3, 74.2, 60.3, 55.4, 55.3, 26.2, 18.6, −4.4, −4.5 ppm; HRMS calcd. For $C_{28}H_{36}O_5SiNa^+$ [M + Na]$^+$ 503.2224, found 503.2226.

6b′ (relative stereochemistry arbitrarily assigned): R_f = 0.22 (silica gel, hexanes:EtOAc 4:1); IR (film) ν_{max} 3533, 3055, 2956, 1597, 1470, 742 cm^{-1}; ^{1}H NMR (400 MHz, C_6D_6): δ 7.85 (d, *J* = 8.6 Hz, 1H), 7.23 (d, *J* = 7.6 Hz, 2H), 7.10 (t, *J* = 7.5 Hz, 2H), 7.01 (t, *J* = 7.3 Hz, 1H),

6.77 (d, J = 8.6 Hz, 1H), 6.59 (t, J = 7.8 Hz, 1H), 6.50 (d, J = 7.7 Hz, 1H), 6.44 (d, J = 7.9 Hz, 1H), 5.91 (s, 1H), 5.66 (s, 1H), 3.62 (s, 3H), 3.35 (s, 3H), 3.19 (s, 3H), 1.04 (s, 9H), 0.16 (s, 3H), 0.02 ppm (s, 3H); ^{13}C NMR (101 MHz, C_6D_6): δ 152.4, 147.5, 146.9, 145.9, 143.5, 137.3, 130.8, 127.7, 127.0, 125.6, 123.4, 121.9, 119.2, 112.5, 110.0, 74.5, 60.4, 55.5, 55.4, 26.3, 18.6, −4.4, −4.7 ppm; HRMS calcd. For $C_{28}H_{36}O_5SiNa^+$ [M + Na]$^+$ 503.2224, found 503.2223.

TBS Ethers 6d and 6d′

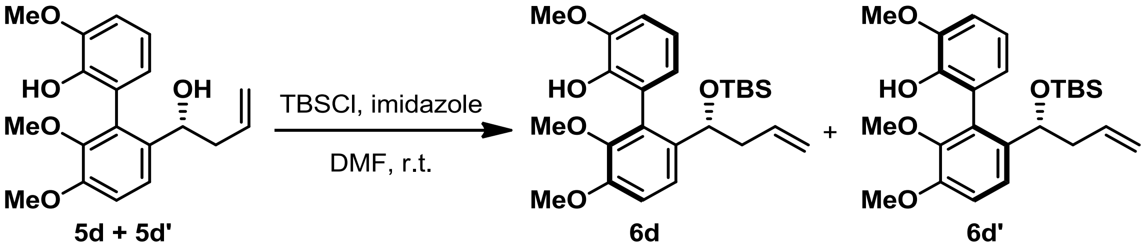

To a stirred solution of alcohols **5d** and **5d′** (170 mg, 0.51 mmol) in DMF (9.00 mL) at room temperature was added TBSCl (310 mg, 2.06 mmol) and imidazole (138 mg, 2.03 mmol). The resulting mixture was stirred for 24 h before it was quenched with NH_4Cl (20 mL, sat. aq.) and water (20 mL). The resulting mixture was extracted with ethyl acetate (3 × 100 mL), the combined organic layer was washed with water (40 mL), brine (40 mL), dried (Na_2SO_4), and concentrated under reduced pressure. Flash column chromatography (silica gel, hexanes:EtOAc 13:1) afforded TBS ethers **6d** and **6d′** (175 mg, 76% combined yield) as an amorphous solid. Small amount of analytically pure isomers **6d** and **6d′** were obtained through column chromatography.

6d (relative stereochemistry arbitrarily assigned): R_f = 0.40 (silica gel, hexanes:EtOAc 4:1); IR (film) ν_{max} 3455, 2965, 2865, 1703, 1338, 1194, 742 cm^{-1}; ^{1}H NMR (499 MHz, $CDCl_3$): δ 7.33 (d, J = 8.6 Hz, 1H), 6.97 (d, J = 8.6 Hz, 1H), 6.94–6.88 (m, 2H), 6.73–6.71 (m, 1H), 5.75–5.68 (m, 1H), 5.61 (br s, 1H), 4.87 (d, J = 15.6 Hz, 1H), 4.80 (d, J = 16.2 Hz, 1H), 4.49–4.44 (m, 1H), 3.94 (s, 3H), 3.89 (s, 3H), 3.62 (s, 3H), 2.32–2.26 (m, 1H), 2.19–2.10 (m, 1H), 0.85 (s, 9H), –0.11 (s, 3H), –0.18 ppm (s, 3H); ^{13}C NMR (101 MHz, $CDCl_3$): δ 151.2, 146.7, 146.0, 142.6, 137.2, 136.0, 129.1, 124.2, 122.4, 121.6, 119.1, 116.1, 111.5, 110.0, 71.3, 60.6,

56.0, 55.7, 44.9, 25.9, 18.1, −4.8, −5.2 ppm; HRMS calcd. For $C_{25}H_{36}O_5SiNa^+$ $[M + Na]^+$ 467.2224, found 467.2226.

6d′ (relative stereochemistry arbitrarily assigned): R_f = 0.42 (silica gel, hexanes:EtOAc 4:1); IR (film) ν_{max} 3458, 2918, 2860, 1706, 1344, 1137 cm^{-1}; ^{1}H NMR (499 MHz, $CDCl_3$): δ 7.34 (d, *J* = 8.7 Hz, 1H), 6.96 (d, *J* = 8.7 Hz, 1H), 6.92–6.89 (m, 2H), 6.73–6.68 (m, 1H), 5.80–5.68 (m, 1H), 5.62 (s, 1H), 4.89 (d, *J* = 15.2 Hz, 1H), 4.82 (d, *J* = 15.4 Hz, 1H), 4.48−4.42 (m, 1H), 3.94 (s, 3H), 3.89 (s, 3H), 3.59 (s, 3H), 2.25–2.09 (m, 2H), 0.87 (s, 9H), −0.10 (s, 3H), −0.17 ppm (s, 3H); ^{13}C NMR (101 MHz, $CDCl_3$): δ 151.2, 146.7, 146.0, 142.5, 137.2, 136.0, 129.1, 124.2, 122.4, 121.6, 119.1, 116.1, 111.5, 110.0, 71.3, 60.6, 56.0, 55.6, 44.9, 25.9, 18.1, −4.8, −5.2 ppm; HRMS calcd. For $C_{25}H_{36}O_5SiNa^+$ $[M + Na]^+$ 467.2224, found 467.2225.

Bis-TBS Ethers TBS-6d and TBS-6d′

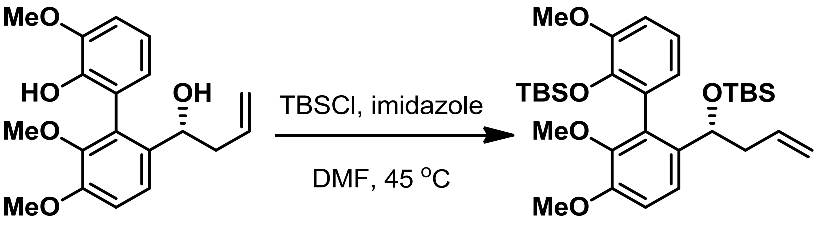

5d + 5d' **TBS-6d + TBS-6d'**

To a stirred solution of alcohols **5d** and **5d′** (0.60 g, 1.82 mmol) in DMF (1.50 mL) at room temperature was added TBSCl (1.10 g, 7.30 mmol) and imidazole (0.49 g, 7.20 mmol). The resulting mixture was warmed to 45 °C and stirred for 24 h before it was cooled to room temperature and quenched with NH_4Cl (20 mL, sat. aq.) and water (20 mL). The resulting mixture was extracted with ethyl acetate (3 × 100 mL), the combined organic layer was washed with water (70 mL), brine (70 mL), dried (Na_2SO_4) and concentrated under reduced pressure. Flash column chromatography (silica gel, hexanes:EtOAc 15:1) afforded bis-TBS ethers **TBS-6d** and **TBS-6d′** (0.95 g, 94% combined yield) as an amorphous solid. **TBS-6d** + **TBS-6d′**: R_f = 0.87 (silica gel, hexanes:EtOAc 4:1); IR (film) ν_{max} 3154, 2912, 1575, 1199, 1012, 722 cm^{-1}; ^{1}H NMR (400 MHz, $CDCl_3$): δ 7.35 (t, *J* = 8.7 Hz, 1H), 7.05–6.84 (m, 3H), 6.78–6.70 (m, 1H), 5.84–5.62 (m, 1H), 4.96–4.73 (m, 2H), 4.61–4.53 (m, 0.5H), 4.47–4.38 (m, 0.5H), 3.88 (s, 3H), 3.83 (s, 3H), 3.59 (s, 2H), 3.57 (s, 1H), 2.46–2.07 (m, 2H), 0.89 (s,

6H), 0.84 (s, 3H), 0.72 (s, 6H), 0.64 (s, 3H), 0.11 (s, 1H), 0.10 (s, 2H), –0.03 (s, 2H), –0.05 (s, 1H), –0.10 (s, 2H), –0.12 (s, 1H), –0.19 (s, 2H), –0.20 ppm (s, 1H); ^{13}C NMR (101 MHz, $CDCl_3$): δ 151.3, 151.2, 150.5, 150.1, 146.2, 146.0, 143.1, 142.6, 137.3, 136.7, 136.6, 135.4, 131.0, 130.8, 128.5, 124.0, 122.3, 121.4, 121.3, 119.9, 116.1, 115.7, 111.3, 110.8, 110.6, 110.5, 71.7, 71.3, 60.4, 60.3, 55.6, 55.6, 54.7, 54.6, 44.0, 26.3, 25.9, 25.8, 25.8, 18.6, 18.5, 18.1, 18.0, –4.0, –4.4, –4.4, –4.5, –4.6, –4.7, –4.8, –4.9 ppm; HRMS calcd. For $C_{31}H_{50}O_5Si_2Na^+$ $[M + Na]^+$ 581.3089, found 581.3090.

TBS Ethers 6d and 6d′

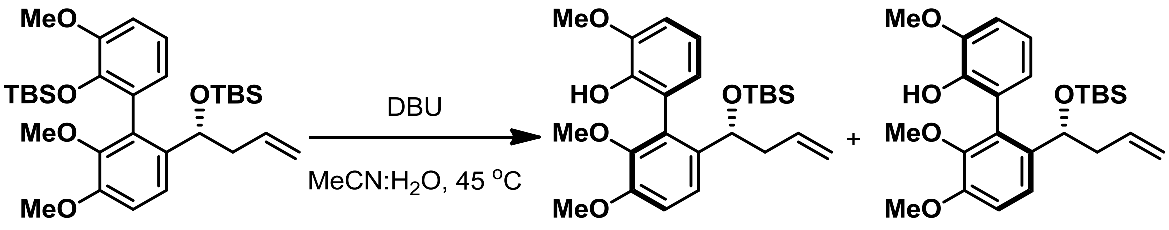

TBS-6d + TBS-6d'   6d   6d'

To a stirred solution of bis-TBS ethers **TBS-6d** and **TBS-6d′** (13.0 g, 23.3 mmol) in MeCN/H_2O (10:1, 75.0 mL) at room temperature was added DBU (3.30 mL, 22.1 mmol). The resulting mixture was warmed to 45 °C and stirred for 24 h before it was cooled to room temperature and quenched with NH_4Cl (40 mL, sat. aq.) and water (40 mL). The resulting mixture was extracted with ethyl acetate (3 × 100 mL), the combined organic layer was washed with water (40 mL), brine (40 mL), dried (Na_2SO_4) and concentrated under reduced pressure. Flash column chromatography (silica gel, hexanes:EtOAc 13:1) afforded TBS ethers **6d** and **6d′** (9.00 g, 87% combined yield) as an amorphous solid. Small amount of analytically pure isomers **6d** and **6d′** were obtained through column chromatography. All physical data of TBS ethers **6d** and **6d′** are identical to those obtained from mono-silylation of alcohols **5d** and **5d′**.

Dienones 7a and 7a′

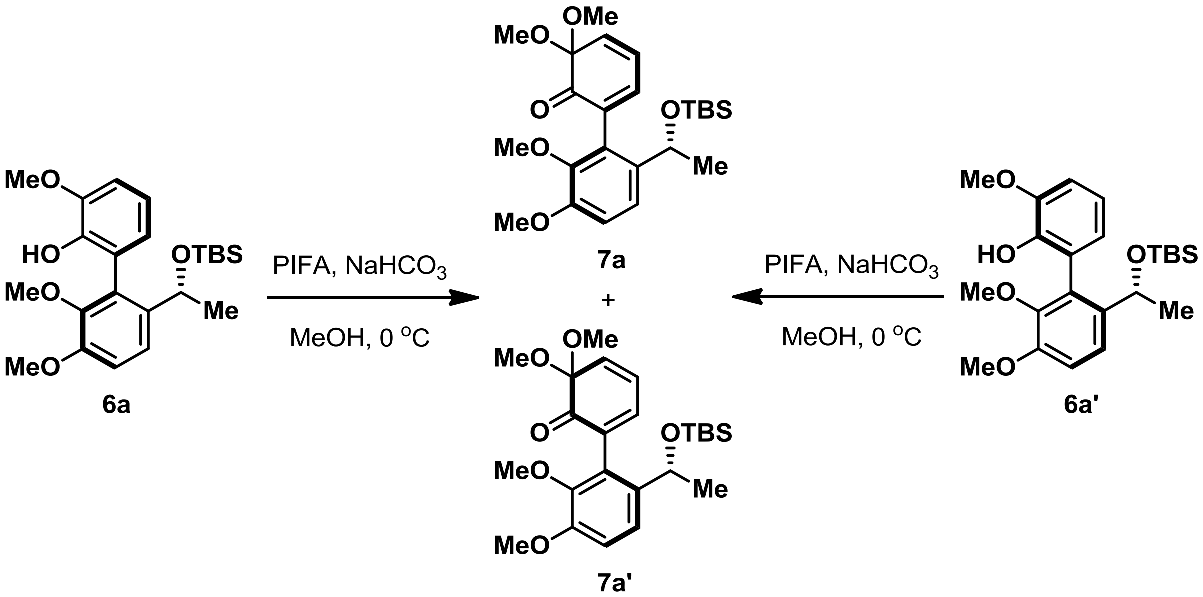

From 6a: To a stirred solution of phenol **6a** (20.0 mg, 48 μmol) in MeOH (3.0 mL) at 0 ℃ was added PIFA (20.6 mg, 48 μmol) and $NaHCO_3$ (40.0 mg, 0.48 mmol). The resulting mixture was stirred for 10 min before it was warmed to room temperature and quenched with $Na_2S_2O_3$ (3 mL, sat. aq.) and water (2 mL). The resulting mixture was extracted with Et_2O (3 × 5 mL), the combined organic layer was washed with $NaHCO_3$ (8 mL, sat. aq.), brine (8 mL), dried (Na_2SO_4) and concentrated under reduced pressure. Flash column chromatography (silica gel, CH_2Cl_2:Et_2O 15:1) afforded dienones **7a** and **7a′** (16.2 mg, 76%) as an orange amorphous solid.

From 6a′: To a stirred solution of phenol **6a′** (14.2 mg, 34 μmol) in MeOH (2.1 mL) at 0 ℃ was added PIFA (14.4 mg, 33 μmol) and $NaHCO_3$ (30.0 mg, 0.36 mmol). The resulting mixture was stirred for 10 min before it was warmed to room temperature and quenched with $Na_2S_2O_3$ (3 mL, sat. aq.) and water (2 mL). The resulting mixture was extracted with Et_2O (3 × 5 mL), the combined organic layer was washed with $NaHCO_3$ (5 mL, sat. aq.), brine (5 mL), dried (Na_2SO_4) and concentrated under reduced pressure. Flash column chromatography (silica gel, CH_2Cl_2:Et_2O 15:1) afforded dienones **7a** and **7a′** (9.1 mg, 60%) as an orange amorphous solid.

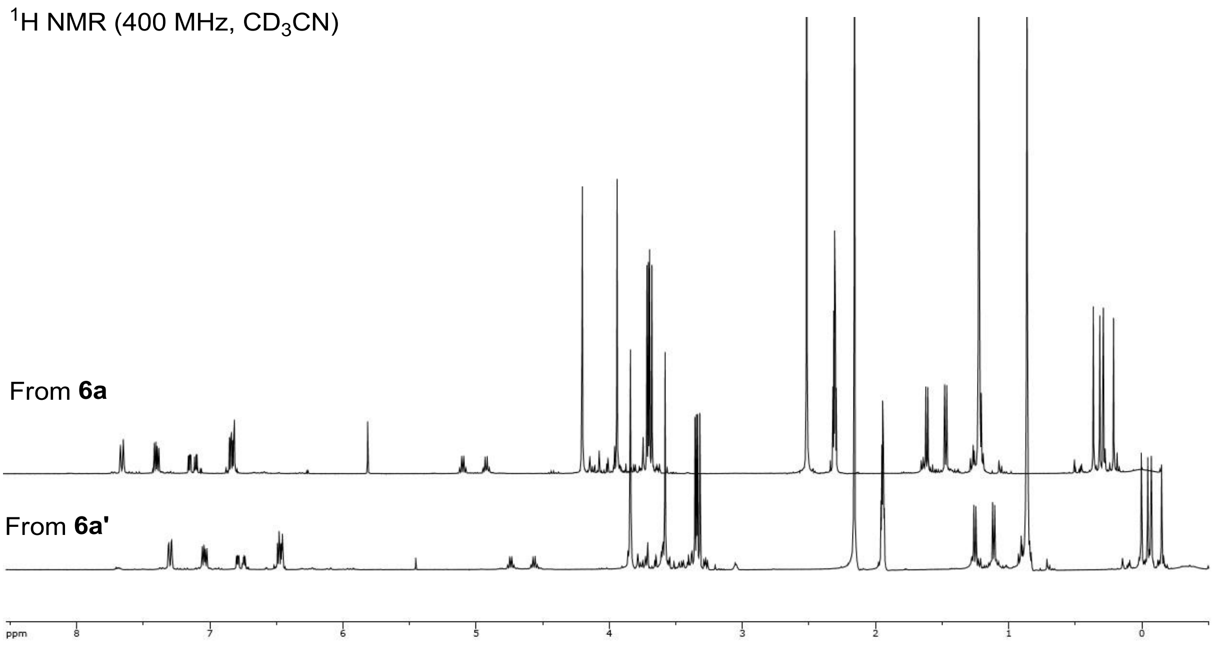

Dienones 7b and 7b′

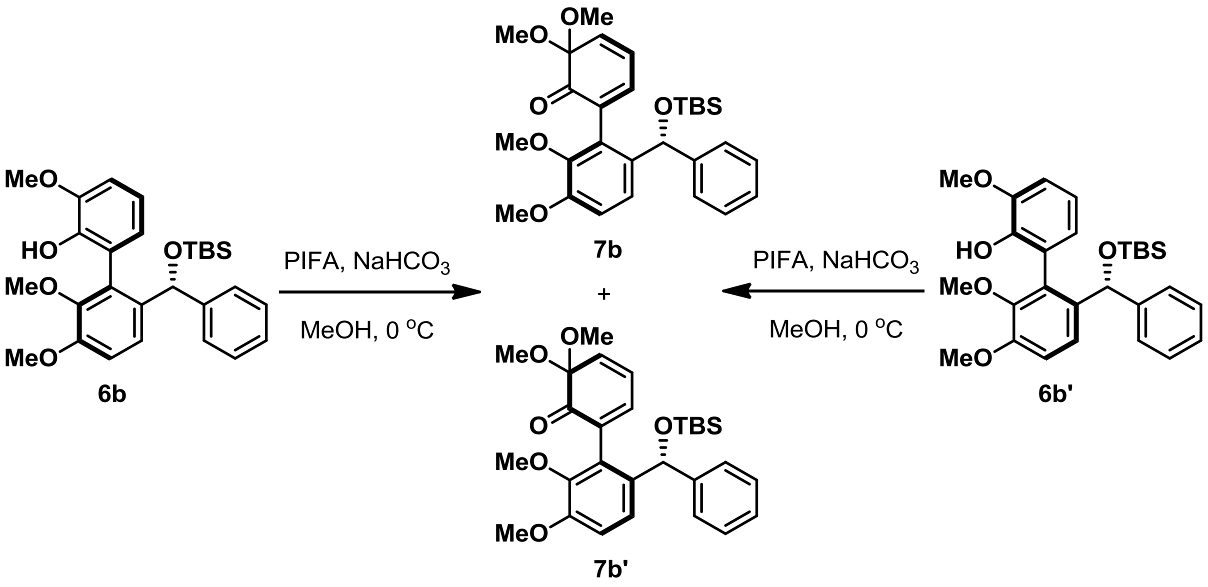

From 6b: To a stirred solution of phenol **6b** (19.0 mg, 40 μmol) in MeOH (3.2 mL) at 0 °C was added PIFA (20.5 mg, 48 μmol) and $NaHCO_3$ (33.2 mg, 0.40 mmol). The resulting mixture was stirred for 10 min before it was warmed to room temperature and quenched with $Na_2S_2O_3$ (4 mL, sat. aq.) and water (2 mL). The resulting mixture was extracted with Et_2O (3 × 5 mL), the combined organic layer was washed with $NaHCO_3$ (8 mL, sat. aq.), brine (8 mL), dried (Na_2SO_4) and concentrated under reduced pressure. Flash column chromatography

(silica gel, CH_2Cl_2:Et_2O 15:1) afforded dienones **7b** and **7b'** (11.8 mg, 58%) as an orange amorphous solid

From 6b': To a stirred solution of phenol **6b'** (12.1 mg, 25 μmol) in MeOH (2.0 mL) at 0 °C was added PIFA (13.2 mg, 31 μmol) and $NaHCO_3$ (21.1 mg, 0.25 mmol). The resulting mixture was stirred for 10 min before it was warmed to room temperature and quenched with $Na_2S_2O_3$ (3 mL, sat. aq.) and water (2 mL). The resulting mixture was extracted with Et_2O (3 × 5 mL), the combined organic layer was washed with $NaHCO_3$ (5 mL, sat. aq.), brine (5 mL), dried (Na_2SO_4) and concentrated under reduced pressure. Flash column chromatography (silica gel, CH_2Cl_2:Et_2O 15:1) afforded dienones **7b** and **7b'** (7.2 mg, 56%) as an orange amorphous solid.

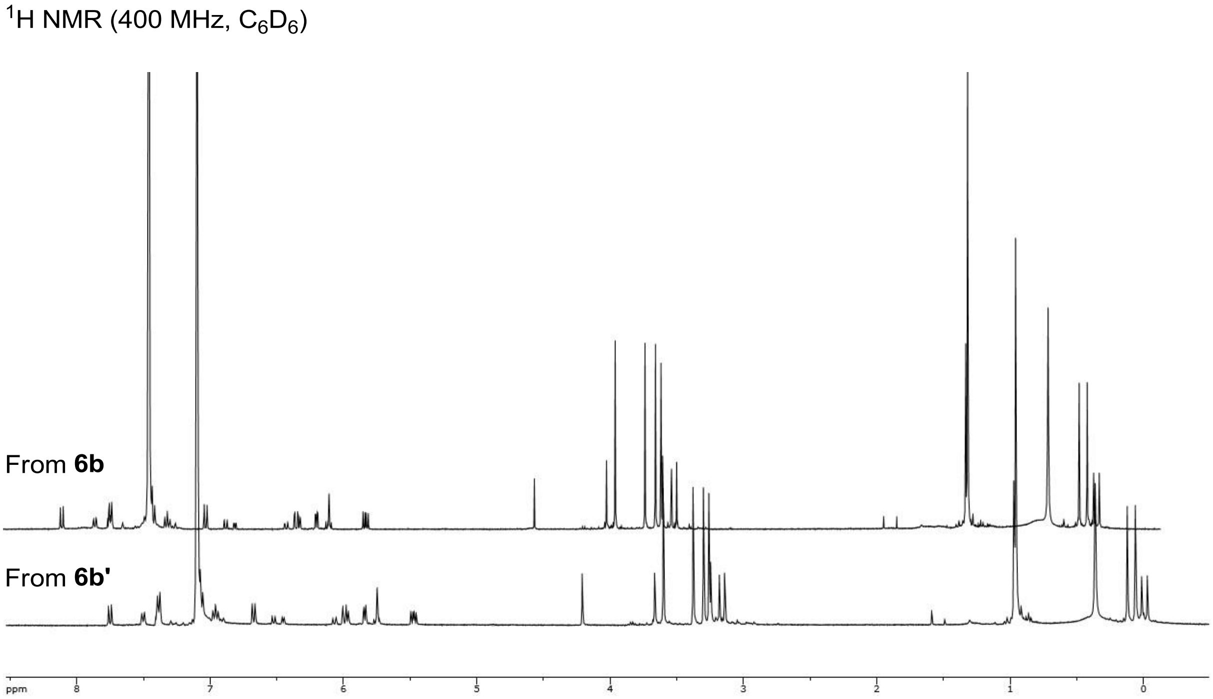

Dienones 7c and 7c′

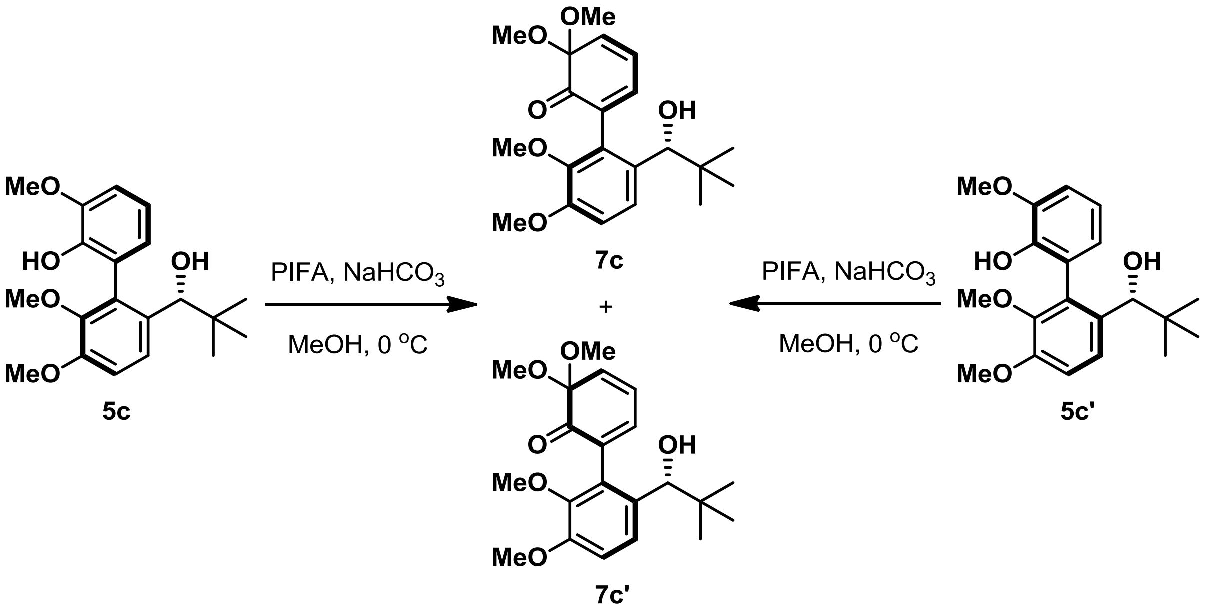

From 5c: To a stirred solution of phenol **5c** (12.8 mg, 37 μmol) in MeOH (2.0 mL) at 0 °C was added PIFA (16.7 mg, 39 μmol) and $NaHCO_3$ (31.1 mg, 0.37 mmol). The resulting mixture was stirred for 10 min before it was warmed to room temperature and quenched with $Na_2S_2O_3$ (3 mL, sat. aq.) and water (2 mL). The resulting mixture was extracted with Et_2O (3 × 5 mL), the combined organic layer was washed with $NaHCO_3$ (5 mL, sat. aq.), brine (5 mL), dried (Na_2SO_4) and concentrated under reduced pressure. Flash column chromatography (silica gel, CH_2Cl_2:Et_2O 15:1) afforded dienones **7c** and **7c′** (8.5 mg, 61%) as an orange amorphous solid.

From 5c′: To a stirred solution of phenol **5c′** (12.0 mg, 35 μmol) in MeOH (2.0 mL) at 0 °C was added PIFA (13.0 mg, 30 μmol) and $NaHCO_3$ (29.1 mg, 0.35 mmol). The resulting mixture was stirred for 10 min before it was warmed to room temperature and quenched with $Na_2S_2O_3$ (3 mL, sat. aq.) and water (2 mL). The resulting mixture was extracted with Et_2O (3 × 5 mL), the combined organic layer was washed with $NaHCO_3$ (5 mL, sat. aq.), brine (5 mL), dried (Na_2SO_4) and concentrated under reduced pressure. Flash column chromatography (silica gel, CH_2Cl_2:Et_2O 15:1) afforded dienones **7c** and **7c′** (6.5 mg, 50%) as an orange amorphous solid.

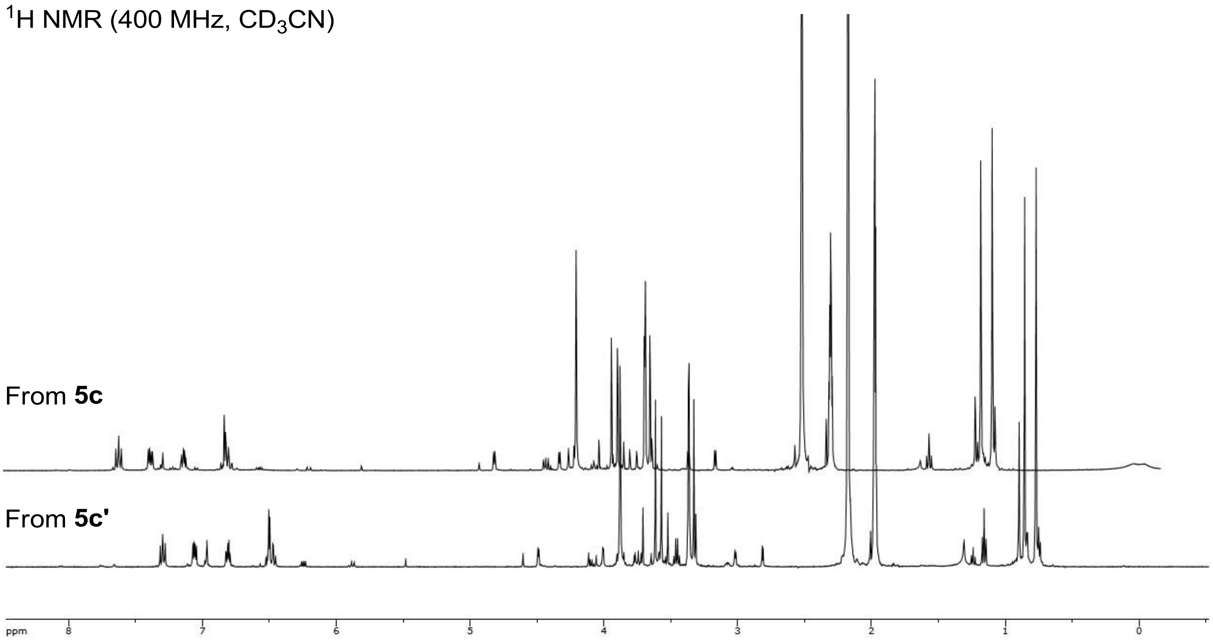

Dienones 7d and 7d’

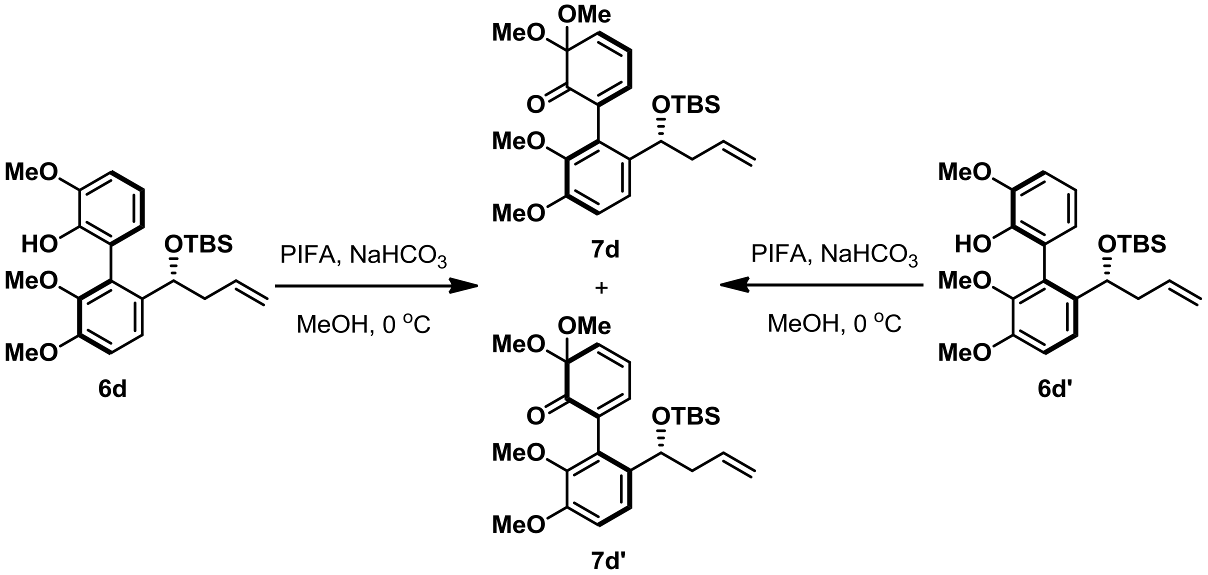

From 6d: To a stirred solution of phenol **6d** (19.1 mg, 43 μmol) in MeOH (3.0 mL) at 0 °C was added PIFA (19.4 mg, 45 μmol) and $NaHCO_3$ (34.2 mg, 0.41 mmol). The resulting mixture was stirred for 10 min before it was warmed to room temperature and quenched with $Na_2S_2O_3$ (3 mL, sat. aq.) and water (2 mL). The resulting mixture was extracted with Et_2O (3 × 5 mL), the combined organic layer was washed with $NaHCO_3$ (8 mL, sat. aq.), brine (8 mL), dried (Na_2SO_4) and concentrated under reduced pressure. Flash column chromatography

(silica gel, CH_2Cl_2:Et_2O 15:1) afforded dienones **7d** and **7d’** (10.7 mg, 52%) as an orange amorphous solid

From 6d’: To a stirred solution of phenol **6d’** (11.5 mg, 26 μmol) in MeOH (1.8 mL) at 0 ℃ was added PIFA (13.2 mg, 31 μmol) and $NaHCO_3$ (21.0 mg, 0.25 mmol). The resulting mixture was stirred for 10 min before it was warmed to room temperature and quenched with $Na_2S_2O_3$ (3 mL, sat. aq.) and water (2 mL). The resulting mixture was extracted with Et_2O (3 × 5 mL), the combined organic layer was washed with $NaHCO_3$ (5 mL, sat. aq.), brine (5 mL), dried (Na_2SO_4) and concentrated under reduced pressure. Flash column chromatography (silica gel, CH_2Cl_2:Et_2O 15:1) afforded dienones **7d** and **7d’** (7.3 mg, 59%) as an orange amorphous solid.

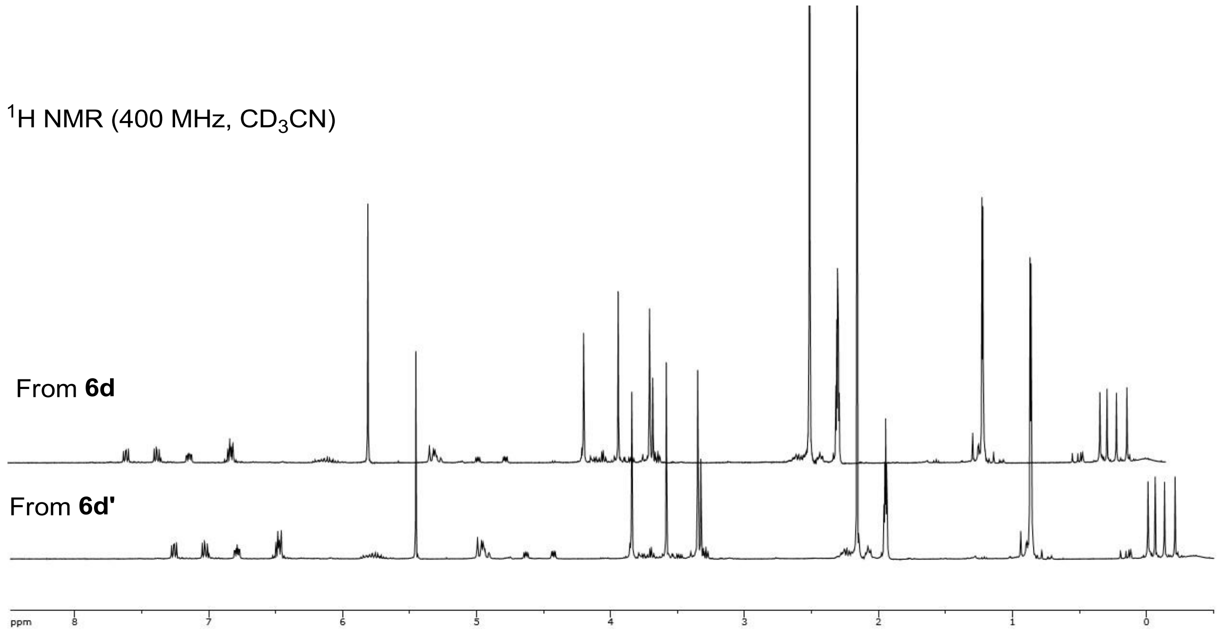

Biaryl Ether 2g

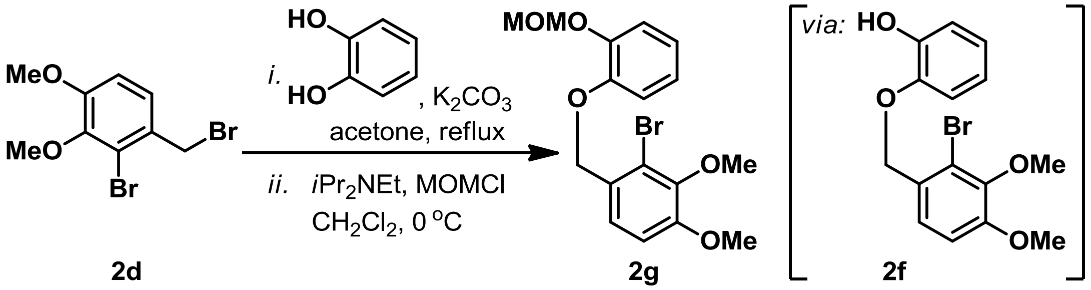

(i) To a stirred solution of dibromide **2d** (450 mg, 1.45 mmol) in acetone (150 mL) at room temperature was added K_2CO_3 (602 mg, 4.36 mmol) followed by catechol (192 mg, 1.74 mmol). The resulting mixture was warmed to reflux and stirred for 16 h before it was cooled to room temperature and diluted with water (100 mL). The resulting mixture was extracted with Et_2O (3 × 75 mL), the combined organic layer was dried (Na_2SO_4) and concentrated under reduced pressure. Flash column chromatography (silica gel, hexanes:EtOAc 4:1) afforded biaryl ether **2f** (433 mg, 88%) as an amorphous white solid.

(ii) To a stirred solution of phenol **2f** (obtained above) in CH_2Cl_2 (130 mL) at 0 °C was added *i*Pr_2NEt (0.33 mL, 1.89 mmol) followed by MOMCl (0.12 mL, 1.58 mmol). The resulting mixture was stirred for 2 h before it was warmed to room temperature and quenched with water (80 mL). The resulting mixture was extracted with Et_2O (3 × 70 mL), the combined organic layer was dried (Na_2SO_4) and concentrated under reduced pressure. Flash column chromatography (silica gel, hexanes:EtOAc 4:1) afforded biaryl ether **2g** (416 mg, 85%) as an amorphous white solid. **2g**: R_f = 0.80 (silica gel, hexanes:EtOAc 2:1); IR (film) ν_{max} 3060, 2940, 1636, 1490, 806, 1002, 750 cm^{-1}; ^1H NMR (499 MHz, $CDCl_3$): δ 7.23 (d, *J* = 9.0 Hz, 1H), 7.13 (d, *J* = 7.0 Hz, 1H), 6.97–6.87 (m, 3H), 6.85 (d, *J* = 9.0 Hz, 1H), 5.22 (s, 2H), 5.14 (s, 2H), 3.86 (s, 3H), 3.84 (s, 3H), 3.51 ppm (s, 3H); ^{13}C NMR (101 MHz, $CDCl_3$): δ 153.1, 149.0, 147.0, 146.4, 129.1, 124.1, 122.8, 121.6, 118.1, 117.7, 114.7, 111.2, 95.8, 70.4, 60.5, 56.2, 56.1 ppm; HRMS calcd. For $C_{17}H_{19}BrO_5Na^+$ $[M + Na]^+$ 405.0308, found 405.0309.

Tricycle 3a:

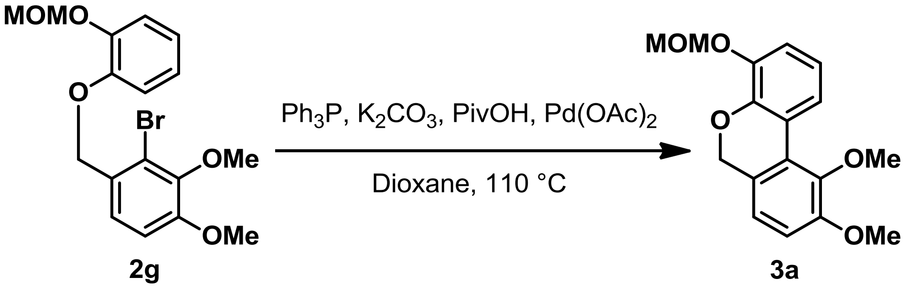

To a stirred solution of biaryl ether **2g** (420 mg, 1.10 mmol) in dioxane (6.5 mL) at room temperature was added K_2CO_3 (454 mg, 3.28 mmol), $Pd(OAc)_2$ (36.9 mg, 0.16 mmol), Ph_3P (129 mg, 0.49 mmol) and PivOH (22.4 mg, 0.22 mmol). The resulting mixture was warmed to 110 °C and stirred for 16 h before it was cooled to room temperature and diluted with water (15 mL). The resulting mixture was extracted with Et_2O (3 × 20 mL), the combined organic layer was washed with water (40 mL), brine (40 mL), dried (Na_2SO_4) and concentrated under reduced pressure. Flash column chromatography (silica gel, hexanes:EtOAc 4:1) afforded tricycle **3a** (235 mg, 71%) as an amorphous white solid. **3a**: R_f = 0.69 (silica gel, hexanes:EtOAc 2:1); IR (film) ν_{max} 3069, 2838, 1606, 1203, 1458, 1342, 720 cm^{-1}; ^{1}H NMR (400 MHz, $CDCl_3$): δ 8.13 (d, J = 8.0 Hz, 1H), 7.12 (d, J = 8.0 Hz, 1H), 6.98 (t, J = 8.2 Hz, 1H), 6.87 (d, J = 8.2 Hz, 1H), 6.82 (d, J = 8.2 Hz, 1H), 5.24 (s, 2H), 4.98 (s, 2H), 3.86 (s, 3H), 3.70 (s, 3H), 3.52 ppm (s, 3H); ^{13}C NMR (101 MHz, $CDCl_3$): δ 153.2, 146.3, 146.1, 145.4, 125.9, 123.3, 122.9, 121.7, 121.4, 120.0, 116.5, 111.2, 95.5, 68.8, 59.7, 56.1, 55.8 ppm; HRMS calcd. For $C_{17}H_{18}O_5Na^+$ $[M + Na]^+$ 325.1046, found 325.1047.

Alcohols 5e and 5e′

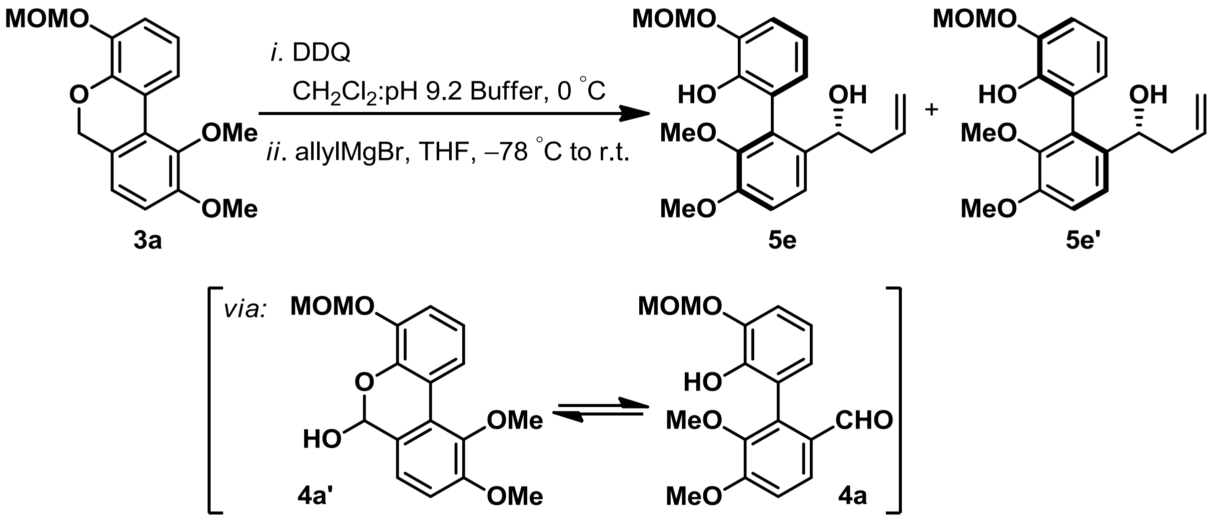

(i) To a stirred solution of biaryl ether **3a** (350 mg, 1.16 mmol) in CH_2Cl_2/pH 9.2 buffer (10:1, 12.1 mL) at 0 °C was added DDQ (315 mg, 1.39 mmol). The resulting mixture was stirred for 1.5 h before the layers were separated and the aqueous layer was extracted with CH_2Cl_2 (3 × 30 mL). The combined organic layer was washed successively with water (until the aqueous layer became colorless), brine (50 mL), dried (Na_2SO_4) and concentrated under reduced pressure afforded crude mixture of hydroxy aldehyde **4a** and hemiacetal **4a′** (**4a**:**4a′**~1:1.4, 324 mg, 88%) as a pale amorphous yellow solid, which was used directly in the subsequent step without further purification.

(ii) To a stirred solution of hemiacetal-hydroxy aldehyde mixture (**4a**+**4a′**, obtained above) in THF (28 mL) at −78 °C was added allylmagnesium bromide (1.0 M in THF, 2.00 mL, 2.00 mmol) dropwise. The resulting mixture was warmed to room temperature and stirred for 16 h before it was quenched with water (30 mL). The layers were separated and the aqueous layer was extracted with Et_2O (3 × 30 mL), the combined organic layer was washed with water (50 mL), brine (50 mL), dried (Na_2SO_4) and concentrated under reduced pressure. Flash column chromatography (silica gel, hexanes:EtOAc 4:1) afforded alcohols **5e** and **5e′** (~4:1 based on ^{1}H NMR analysis, 359 mg, 98% combined yield) as a clear amorphous solid. **5e**+**5e′**: R_f = 0.35, 0.10 (silica gel, hexanes:EtOAc 2:1); IR (film) ν_{max} 3126, 2990, 1573, 1421, 1262, 751 cm^{-1}; ^{1}H NMR (400 MHz, $CDCl_3$): δ 7.27 (d, J = 8.1 Hz, 1H), 7.10 (d, J = 8.4 Hz, 1H), 6.96 (d, J = 8.4 Hz, 1H), 6.85 (t, J = 8.0 Hz, 1H), 6.73 (d, J = 8.0 Hz, 1H), 6.31 (br s, 0.75H), 6.01

(br s, 0.25H), 5.70–5.52 (m, 1H), 5.17 (s, 2H), 4.98–4.88 (m, 2H), 4.43 (m, 0.25H), 4.39 (t, J = 6.8 Hz, 0.75H), 3.85 (s, 3H), 3.57 (s, 0.75H), 3.53 (s, 2.25H), 3.47 (s, 3H), 2.86 (br s, 0.75H), 2.45–2.22 ppm (m, 2.25H); ^{13}C NMR (101 MHz, $CDCl_3$): δ 151.9, 151.6, 146.2, 146.1, 144.5, 144.0, 143.1, 135.5, 135.4, 135.2, 134.8, 130.7, 130.3, 125.3, 124.5, 123.3, 123.1, 121.3, 119.7, 119.5, 117.2, 116.9, 114.8, 114.6, 112.1, 112.0, 95.9, 69.8, 60.4, 60.3, 56.3, 56.3, 55.6, 55.6, 42.5, 41.5 ppm; HRMS calcd. For $C_{20}H_{24}O_6Na^+$ $[M + Na]^+$ 383.1465, found 383.1464.

Phenols 6e and 6e′

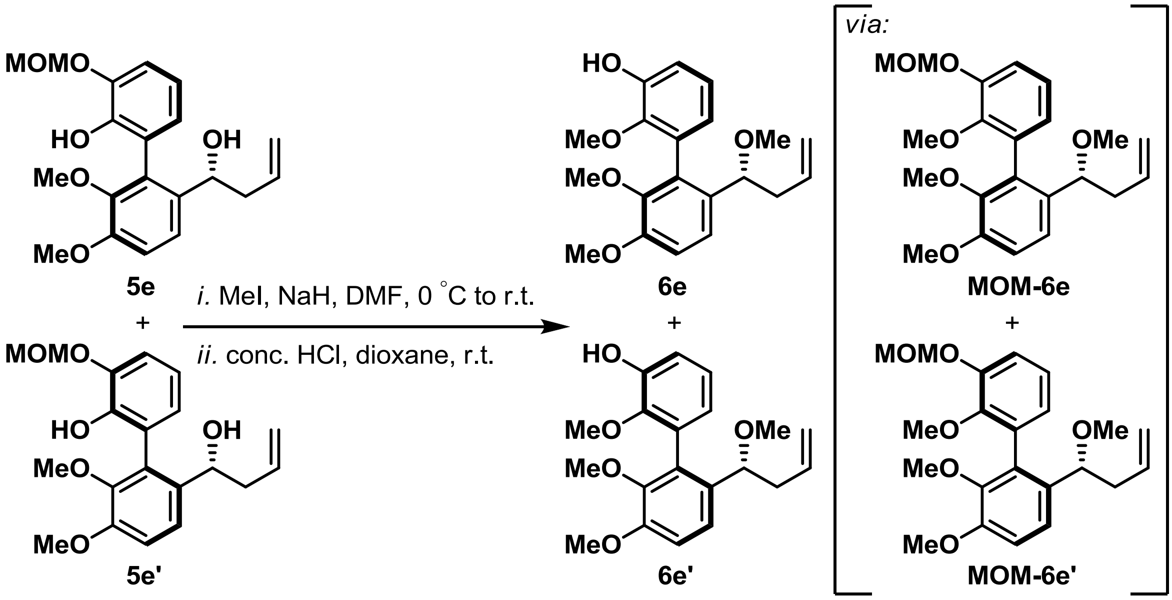

(i) To a stirred solution of alcohols **5e** and **5e′** (360 mg, 1.00 mmol) in DMF (13 mL) at 0 °C was added NaH (230 mg, 9.58 mmol) and MeI (0.18 mL, 2.89 mmol). The resulting mixture was warmed to room temperature and stirred for 10 h before it was quenched with MeOH (20 mL) and brine (20 mL). The resulting mixture was extracted with Et_2O (3 × 30 mL), the combined organic layer was washed with water (40 mL), brine (40 mL), dried (Na_2SO_4) and concentrated under reduced pressure. Flash column chromatography (silica gel, hexane:EtOAc 6:1) afforded MOM ethers **MOM-6e** and **MOM-6e′** (~4:1 based on ^{1}H NMR analysis, 338 mg, 87% combined yield) as a yellow amorphous solid. **MOM-6e** + **MOM-6e′**: R_f = 0.65, 0.70 (silica gel, hexanes:EtOAc 2:1).

(ii) To a stirred solution of MOM ethers (**MOM-6e + MOM-6e′**, obtained above) in dioxane (5.5 mL) was added HCl (0.1 M aq., 0.15 mL, 1.5 mmol). The resulting mixture was stirred for 2 h before it was carefully quenched with $NaHCO_3$ (30 mL). The layers were separated and the aqueous layer was extracted with Et_2O (3 × 20 mL), the combined organic layer was washed with water (50 mL), brine (50 mL), dried (Na_2SO_4) and concentrated under reduced pressure. Flash column chromatography (silica gel, hexanes:EtOAc 6:1) afforded phenols **6e** (206 mg, 69%) and **6e′** (52 mg, 17%) as a white amorphous solids.

6e (relative stereochemistry arbitrarily assigned): R_f = 0.57 (silica gel, hexanes:EtOAc 2:1); IR (film) ν_{max} 3692, 3154, 2988, 1215, 908, 753 cm^{-1}; ^{1}H NMR (400 MHz, $CDCl_3$): δ 7.23 (d, *J* = 8.9 Hz, 1H), 7.01–6.93 (m, 3H), 6.57 (d, *J* = 7.4 Hz, 1H), 5.99 (s, 0.75H), 5.94 (s, 0.25H), 5.66–5.54 (m, 1H), 4.86 (d, *J* = 10.1 Hz, 1H), 4.80 (d, *J* = 17.2 Hz, 1H), 3.94–3.85 (m, 4H), 3.68 (s, 3H), 3.48 (s, 3H), 3.17 (s, 3H), 2.23–2.12 ppm (m, 2H); ^{13}C NMR (101 MHz, $CDCl_3$): δ 151.6, 148.8, 145.9, 143.9, 135.1, 133.7, 131.7, 128.6, 123.7, 123.2, 121.0, 116.3, 114.6, 111.9, 79.7, 60.7, 60.4, 56.5, 55.6, 42.3 ppm; HRMS calcd. For $C_{20}H_{24}O_5Na^+$ $[M + Na]^+$ 367.1516, found 367.1514.

6e′ (relative stereochemistry arbitrarily assigned): R_f = 0.60 (silica gel, hexanes:EtOAc 2:1); IR (film) ν_{max} 3523, 3052, 2988, 1267, 914, 765 cm^{-1}; ^{1}H NMR (400 MHz, C_6D_6): δ 7.39 (d, *J* = 8.6 Hz, 1H), 7.01 (d, *J* = 7.9 Hz, 1H), 6.87 (t, *J* = 7.8 Hz, 1H), 6.69 (d, *J* = 8.6 Hz, 1H), 6.65 (d, *J* = 7.8 Hz, 1H), 6.15–6.02 (m, 1H), 5.52 (s, 1H), 5.14 (d, *J* = 16.7 Hz, 1H), 5.04 (d, *J* = 10.2 Hz, 1H), 4.08 (t, *J* = 6.2 Hz, 1H), 3.64 (s, 3H), 3.35 (s, 3H), 3.22 (s, 3H), 3.05 (s, 3H), 2.68 ppm (t, *J* = 6.2 Hz, 2H); ^{13}C NMR (101 MHz, $CDCl_3$): δ 151.7, 148.8, 145.7, 145.2, 135.8, 133.6, 132.5, 128.9, 123.8, 122.4, 121.8, 116.2, 114.5, 112.2, 79.2, 60.8, 60.5, 56.2, 55.7, 41.2 ppm; HRMS calcd. For $C_{20}H_{24}O_5Na^+$ $[M + Na]^+$ 367.1516, found 367.1517.

Atropisomer Thermal Stability Studies:

NMR samples of atropisomerically pure **6e** and **6e'** in C_6D_6 were subjected to heating (oil bath) at temperatures 40 ℃, 70 ℃, 90 ℃, 100 ℃, 110 ℃, 120 ℃ over 1 hour intervals. The samples were then cooled to room temperature after each incremental heating, and degree of atropisomerization analyzed by ^{1}H NMR analysis. Finally, heating was maintained at 120 ℃ until atropisomeric ratio remained constant.

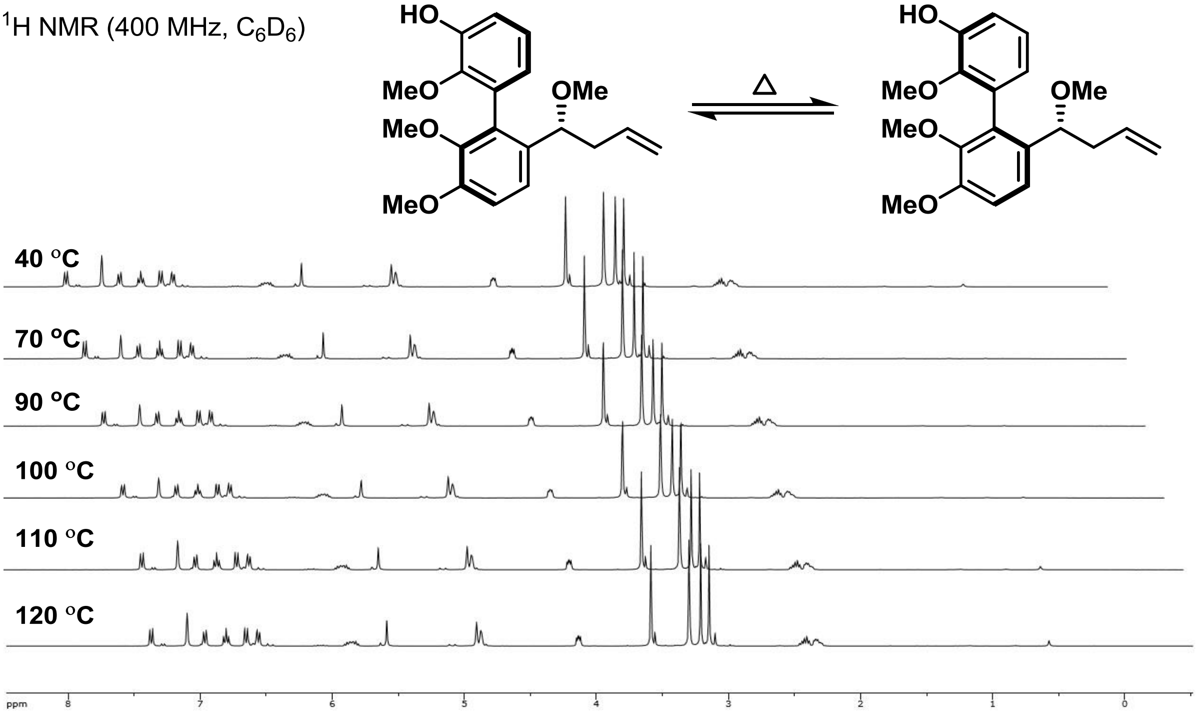

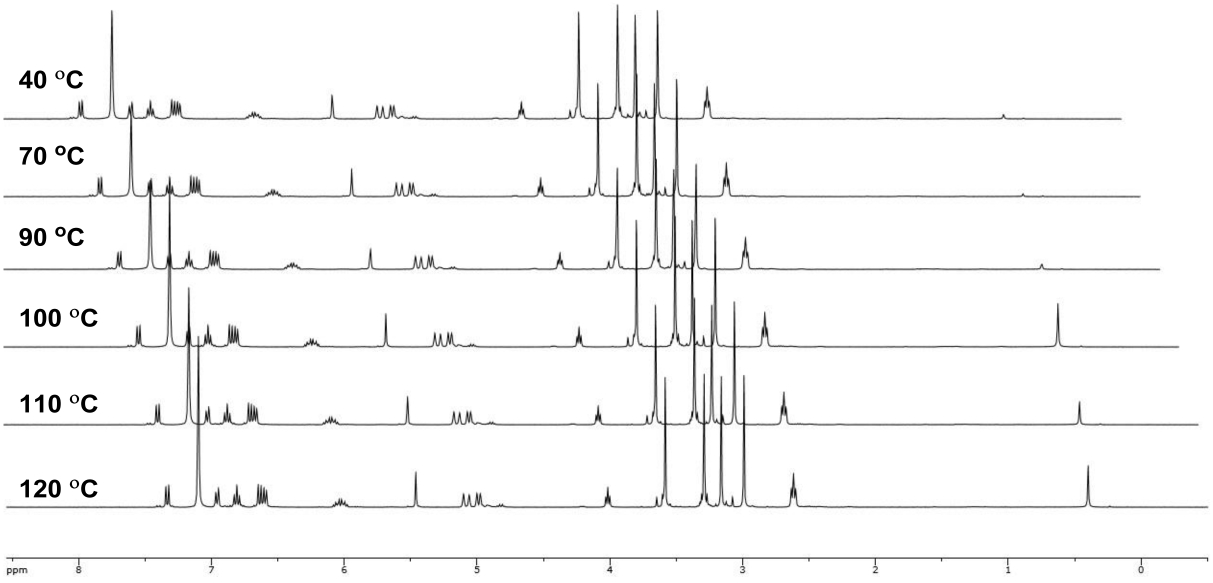

Dienone 7e and 7e′

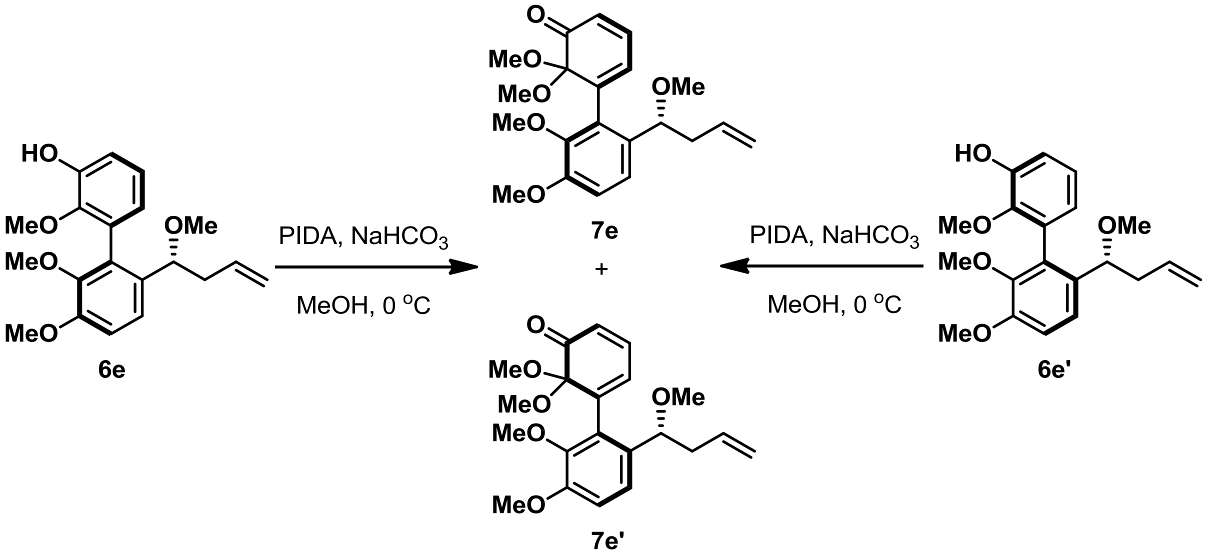

From 6e: To a stirred solution of PIDA (34.8 mg, 10.8 mmol) and $NaHCO_3$ (76.0 mg, 0.90 mmol) in MeOH (3 mL) at 0 °C was added a solution of phenol **6e** (31.0 mg, 90 μmol) in MeOH (1.5 mL) dropwise. The resulting mixture was stirred for 5 min before it was warmed to room temperature and quenched with $Na_2S_2O_3$ (8 mL, sat. aq.) and water (8 mL). The resulting mixture was extracted with Et_2O (3 × 10 mL), the combined organic layer was washed with $NaHCO_3$ (20 mL, sat. aq.), brine (20 mL), dried (Na_2SO_4) and concentrated under reduced pressure. Flash column chromatography (silica gel, hexane:EtOAc 6:1) afforded dienones **7e** and **7e′** (28.0 mg, 83%) as an amorphous yellow solid.

From 6e′: To a stirred solution of PIDA (4.5 mg, 14 μmol) and $NaHCO_3$ (9.8 mg, 0.12 mmol) in MeOH (0.5 mL) at 0 °C was added a solution of phenol **6e′** (4.0 mg, 12 μmol) in MeOH (0.5 mL) dropwise. The resulting mixture was stirred for 5 min before it was warmed to room temperature and quenched with $Na_2S_2O_3$ (3 mL, sat. aq.) and water (3 mL). The resulting mixture was extracted with Et_2O (3 × 10 mL), the combined organic layer was washed with $NaHCO_3$ (15 mL, sat. aq.), brine (15 mL), dried (Na_2SO_4) and concentrated under reduced pressure. Flash column chromatography (silica gel, hexane:EtOAc 6:1) afforded dienones **7e** and **7e′** (3.2 mg, 74%) as an amorphous yellow solid.

^1H NMR (400 MHz, $CDCl_3$)

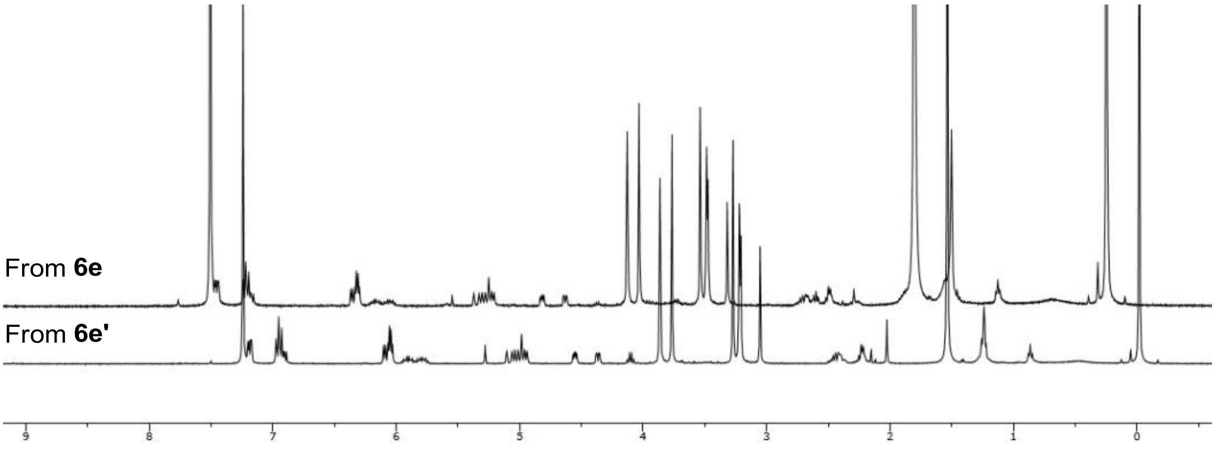

Biaryl Ester 2i

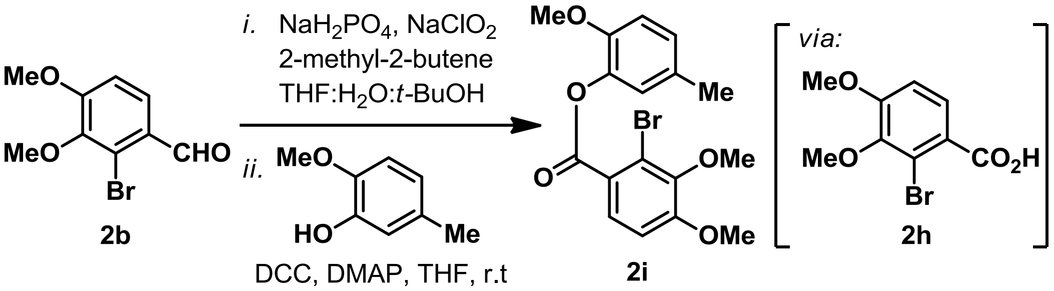

(i) To a stirred solution of aldehyde **2b** (390 mg, 1.59 mmol) in THF/H_2O/*t*BuOH (4:4:1, 36 mL) at room temperature was added NaH_2PO_4 (1.53 g, 12.8 mmol), $NaClO_2$ (576 mg, 6.37 mmol) and 2-methyl-2-butene (1.69 mL, 16.0 mmol). The resulting mixture was stirred for 3 h before it was diluted with EtOAc (30 mL). The resulting mixture was washed with HCl (1.0 N, 3 × 35 mL), the organic layer was separated and dried (Na_2SO_4), and concentrated under reduced pressure afforded crude acid **2h** (413 mg, 99%) as an amorphous white solid, which was sufficiently pure based on thin-layer-chromatography and ^1H NMR analysis and used directly in the subsequent step without further purification.

(ii) To a stirred solution of acid **2h** (crude, obtained above) and 5-methylguaiacol (198 mg, 1.43 mmol) in THF (16 mL) at 0 °C was added DCC (360 mg, 1.74 mmol) and DMAP (20.0 mg, 0.16 mmol). The resulting mixture was warmed to room temperature and stirred for 10 h before it was filtered and concentrated under reduced pressure. Flash column chromatography (silica gel, hexanes:EtOAc 4:1) afforded biaryl ester **2i** (343 mg, 57%) as an amorphous white

solid. **2i**: R_f = 0.38 (silica gel, hexanes:EtOAc 3:1); IR (film) ν_{max} 3053, 2940, 1748, 1586, 1127, 740 cm^{-1}; ^1H NMR (400 MHz, $CDCl_3$): δ 7.92 (d, *J* = 8.0 Hz, 1H), 7.02–6.98 (m, 2H), 6.93 (d, *J* = 8.7 Hz, 1H), 6.89 (d, *J* = 8.2 Hz, 1H), 3.94 (s, 3H), 3.88 (s, 3H), 3.80 (s, 3H), 2.31 ppm (s, 3H); ^{13}C NMR (101 MHz, $CDCl_3$): δ 163.3, 156.5, 148.8, 147.0, 139.2, 130.2, 128.4, 126.9, 123.7, 123.3, 118.9, 112.2, 110.2, 60.1, 55.9, 55.7, 20.2 ppm; HRMS calcd. For $C_{17}H_{17}BrO_5Na^+$ $[M + Na]^+$ 403.0152, found 403.0150.

Tricyclic Lactone 3b

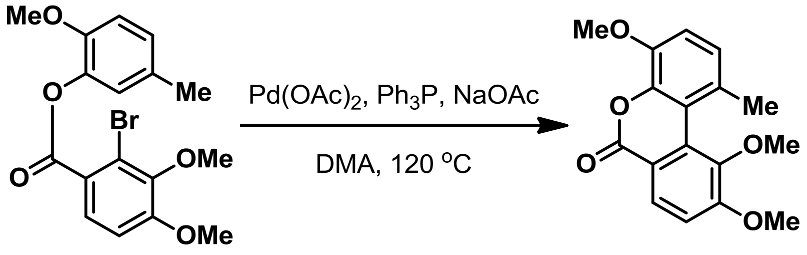

2i 3b

To a stirred solution of biaryl ester **2i** (340 mg, 0.89 mmol) in DMA (10.5 mL) at room temperature was added NaOAc (146 mg, 1.78 mmol), $Pd(OAc)_2$ (31.4 mg, 0.14 mmol) and Ph_3P (70.2 mg, 0.27 mmol). The resulting mixture was warmed to 100 °C and stirred for 24 h before it was cooled to room temperature and diluted with water (30 mL). The resulting mixture was extracted with Et_2O (3 × 30 mL), the combined organic layer was washed with water (50 mL), brine (50 mL), dried (Na_2SO_4) and concentrated under reduced pressure. Flash column chromatography (silica gel, hexanes:EtOAc 4:1) afforded tricyclic lactone **3b** (160 mg, 60%) as an amorphous white solid. **3b**: R_f = 0.30 (silica gel, hexanes:EtOAc 3:1); IR (film) ν_{max} 2992, 1732, 1566, 1257, 1173, 760 cm^{-1}; ^1H NMR (499 MHz, $CDCl_3$): δ 8.17 (d, *J* = 9.1 Hz, 1H), 7.18 (d, *J* = 9.1 Hz, 1H), 7.10 (d, *J* = 9.1 Hz, 1H), 7.00 (d, *J* = 9.1 Hz, 1H), 4.02 (s, 3H), 3.95 (s, 3H), 3.48 (s, 3H), 2.40 ppm (s, 3H); ^{13}C NMR (126 MHz, $CDCl_3$): δ 160.8, 158.2, 145.3, 145.1, 140.6, 129.0, 128.8, 127.2, 126.6, 117.5, 117.1, 112.3, 112.2, 60.9, 56.3, 56.3, 22.7 ppm; HRMS calcd. For $C_{17}H_{16}O_5Na^+$ $[M + Na]^+$ 323.0890, found 323.0891.

Allylic Alcohols 6f and 6f′

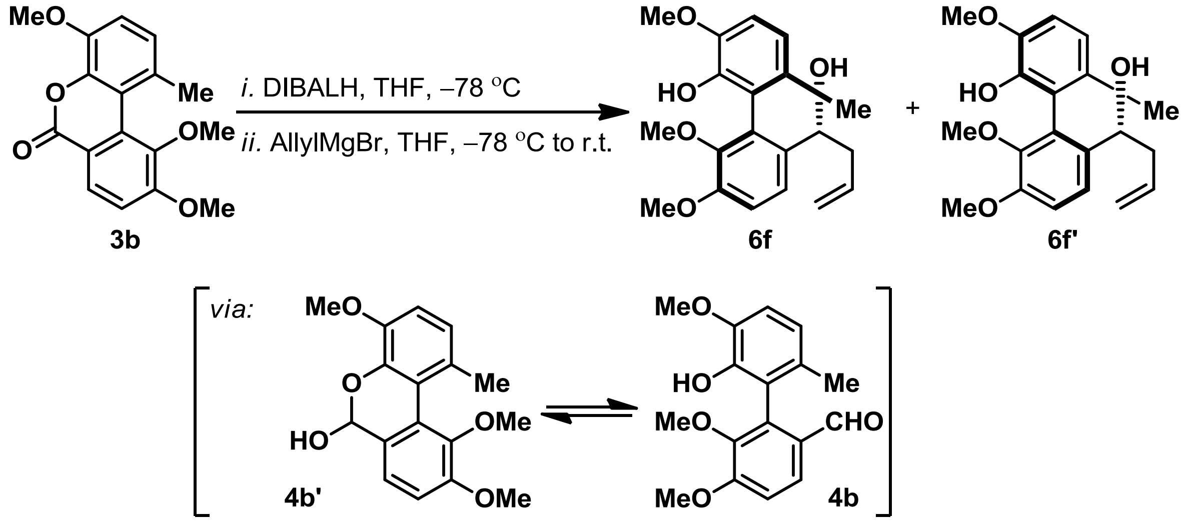

(i) To a stirred solution of lactone **3b** (40.0 mg, 0.13 mmol) in THF (3 mL) at –78 °C was added DIBAL-H (1.0 M in hexane, 0.27 mL, 0.27 mmol). The resulting mixture was stirred for 7 min before it was quenched with sodium potassium tartrate (5 mL, sat. aq.) and diluted with EtOAc (5 mL), and stirred vigorously for 1 h. The layers were separated and the aqueous layer was extracted with ethyl acetate (3 × 8 mL), the combined organic layer was washed with water (15 mL), brine (15 mL), dried (Na_2SO_4) and concentrated under reduced pressure to afford a crude mixture of hydroxy aldehyde **4b** and hemiacetal **4b′** (18 mg, 45%) as an amorphous white solid, which was used directly in the subsequent step without further purification.

(ii) To a stirred solution of a hemiacetal hydroxy aldehyde mixture (**4b**+**4b′**, obtained above) in THF (1.0 mL) at –78 °C was added allylmagnesium bromide (1.0 M in Et_2O, 71 μL, 71 μmol). The resulting mixture was warmed to room temperature and stirred for 16 h before it was quenched with water (3 mL). The layers were separated and the aqueous layer was extracted with Et_2O (3 × 5 mL), the combined organic layer was washed with water (10 mL), brine (10 mL), dried (Na_2SO_4) and concentrated under reduced pressure. Flash column chromatography (silica gel, hexanes:EtOAc 4:1) afforded alcohols **6f** (8.5 mg, 41%) and **6f′** (7.5 mg, 37%) as a yellow amorphous solid.

6f (relative stereochemistry arbitrarily assigned): R_f = 0.39 (silica gel, hexanes:EtOAc 1:1); IR (film) ν_{max} 3151, 2986, 1731, 1266, 1139, 757 cm^{-1}; ^{1}H NMR (400 MHz, C_6D_6): δ 7.46 (d,

J = 8.2 Hz, 1H), 6.71 (t, *J* = 9.2 Hz, 2H), 6.42 (d, *J* = 8.2 Hz, 1H), 5.80–5.68 (m, 1H), 5.60 (s, 1H), 4.90 (d, *J* = 17.0 Hz, 1H), 4.84 (d, *J* = 10.1 Hz, 1H), 4.67 (t, *J* = 6.1 Hz, 1H), 3.53 (s, 3H), 3.28 (s, 3H), 3.17 (s, 3H), 2.74–2.57 (m, 2H), 2.12 ppm (s, 3H); ^{13}C NMR (101 MHz, C_6D_6): δ 152.8, 147.0, 144.7, 143.0, 135.9, 135.6, 131.3, 130.9, 123.5, 122.1, 121.0, 117.0, 112.7, 109.8, 70.8, 60.2, 55.2, 41.3, 20.0 ppm; HRMS calcd. For $C_{20}H_{24}O_5Na^+$ $[M + Na]^+$ 367.1516, found 367.1517.

6f′ (relative stereochemistry arbitrarily assigned): R_f = 0.48 (silica gel, hexanes:EtOAc 1:1); IR (film) ν_{max} 3054, 2986, 1730, 1266, 1165, 763 cm^{-1}; ^{1}H NMR (400 MHz, C_6D_6): δ 7.47 (d, *J* = 8.5 Hz, 1H), 6.71 (dd, *J* = 8.2, 3.9 Hz, 2H), 6.45 (d, *J* = 8.1 Hz, 1H), 5.80–5.70 (m, 1H), 5.52 (s, 1H), 4.93 (d, *J* = 17.1 Hz, 1H), 4.88 (d, *J* = 9.4 Hz, 1H), 4.51 (dd, *J* = 8.3, 3.4 Hz, 1H), 3.69 (s, 3H), 3.36 (s, 3H), 3.19 (s, 3H), 2.69–2.63 (m, 1H), 2.50–2.41 (m, 1H), 2.10 ppm (s, 3H); ^{13}C NMR (101 MHz, $CDCl_3$): δ 152.1, 146.1, 144.5, 142.9, 135.4, 135.3, 129.8, 129.4, 122.4, 121.6, 120.6, 117.8, 112.0, 110.0, 70.1, 60.4, 56.0, 55.7, 42.9, 19.5 ppm; HRMS calcd. For $C_{20}H_{24}O_5Na^+$ $[M + Na]^+$ 367.1516, found 367.1516.

Atropisomer Thermal Stability Studies:

NMR samples of atropisomerically pure **6f** and **6f′** in C_6D_6 were subjected to heating (oil bath) at temperatures 40 °C, 70 °C, 90 °C, 100 °C, 110 °C, 120 °C over 1 hour intervals. The samples were then cooled to room temperature after each incremental heating, and degree of atropisomerization analyzed by ^1H NMR analysis. Finally, heating was maintained at 120 °C until atropisomeric ratio remained constant.

^1H NMR (400 MHz, C_6D_6)

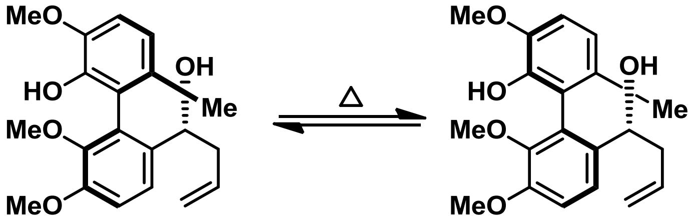

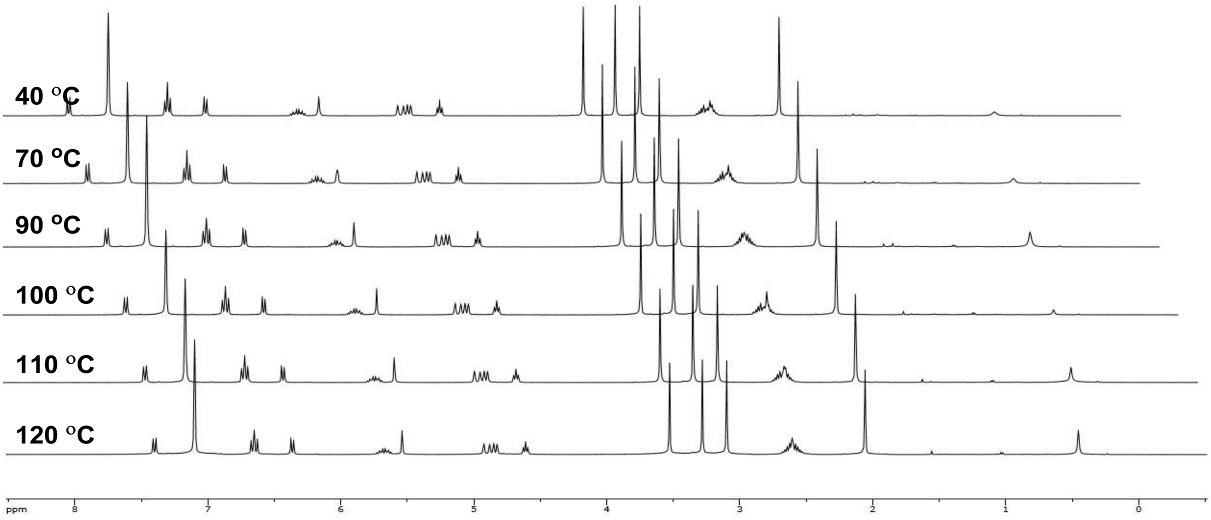

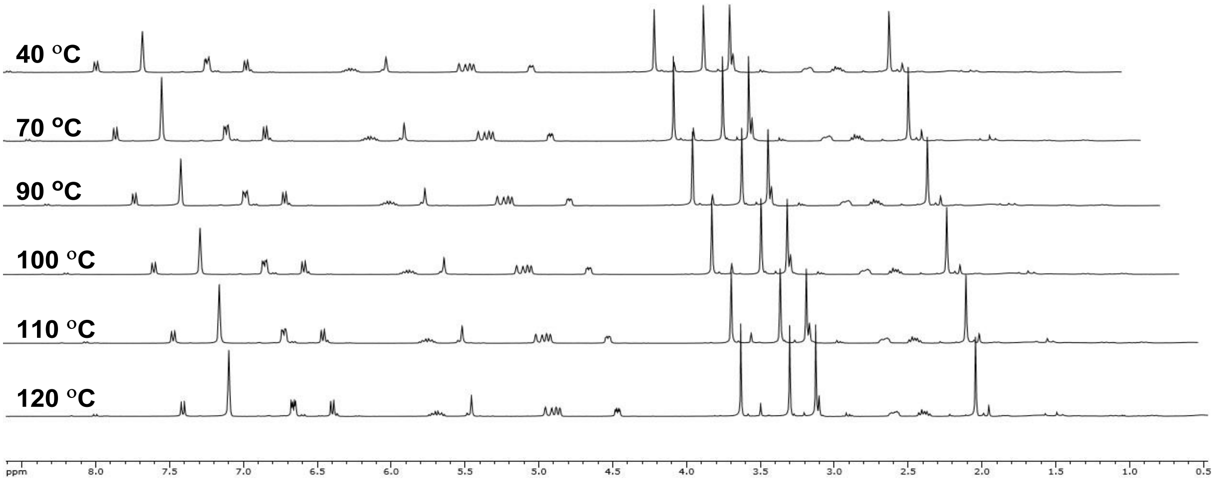

Tetracycle 8f

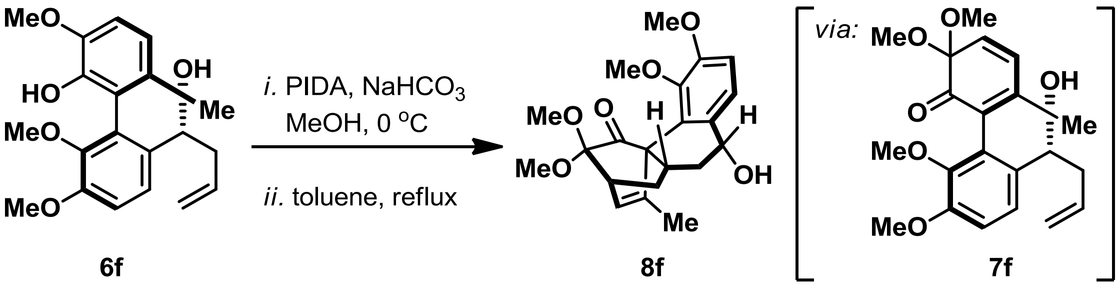

(i) To a stirred solution of PIDA (9.5 mg, 29 μmol) and $NaHCO_3$ (24.9 mg, 0.30 mmol) in MeOH (1.0 mL) at 0 °C was added a solution of phenol **6f** (8.5 mg, 25 μmol) in MeOH (0.2 mL) dropwise. The resulting mixture was stirred for 5 min before it was warmed to room temperature and quenched with $Na_2S_2O_3$ (3 mL, sat. aq.) and water (3 mL). The resulting mixture was extracted with Et_2O (3 × 8 mL), the combined organic layer was washed with $NaHCO_3$ (12 mL, sat. aq.), brine (12 mL), dried (Na_2SO_4) and concentrated under reduced pressure. Flash column chromatography (silica gel, hexanes:EtOAc 4:1) afforded dienone **7f** as an amorphous yellow solid.

ii) A solution of dienone **7f** (obtained above) in toluene (1.0 mL) was warmed to reflux and stirred for 4 h before it was cooled to room temperature and concentrated under reduced pressure. Flash column chromatography (silica gel, hexanes:EtOAc 4:1) afforded tetracycle **8f** (6.5 mg, 70% over two steps) as an amorphous yellow solid. **8f** (relative stereochemistry arbitrarily assigned): R_f = 0.48 (silica gel, hexanes:EtOAc 1:1); IR (film) ν_{max} 3478, 2957, 1747, 1467, 1239, 764 cm^{-1}; ^1H NMR (400 MHz, $CDCl_3$): δ 7.33 (d, *J* = 8.6 Hz, 1H), 6.98 (d, *J* = 8.6 Hz, 1H), 6.20 (d, *J* = 6.7 Hz, 1H), 4.78–4.67 (m, 1H), 3.86 (s, 3H), 3.80 (s, 3H), 3.37 (s, 6H), 3.00 (d, *J* = 7.7 Hz, 1H), 2.26 (t, *J* = 9.6 Hz, 1H), 2.15–2.08 (m, 1H), 2.02–1.97 (m, 1H), 1.69 (d, *J* = 7.7 Hz, 1H), 1.53 (s, 3H), 1.30 (q, *J* = 12.5, 1H), 1.01–0.96 ppm (m, 1H); ^{13}C NMR (101 MHz, $CDCl_3$): δ 200.5, 151.4, 146.8, 137.1, 134.3, 126.5, 125.1, 121.3, 113.0, 94.9, 69.3, 59.9, 59.2, 55.8, 50.8, 49.2, 38.4, 37.5, 36.5, 29.1, 21.0 ppm; HRMS calcd. For $C_{21}H_{26}O_6Na^+$ $[M + Na]^+$ 397.1622, found 397.1624.

Tetracycle 8f′

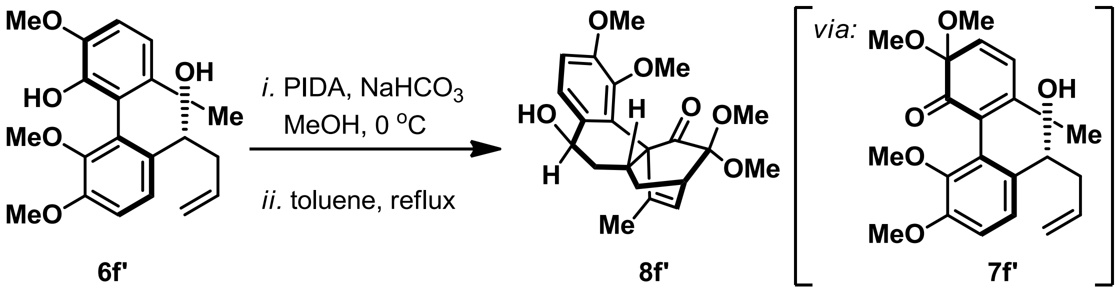

(i) To a stirred solution of PIDA (8.4 mg, 26 μmol) and $NaHCO_3$ (18.3 mg, 0.22 mmol) in MeOH (1.0 mL) at 0 °C was added a solution of phenol **6f′** (7.5 mg, 22 μmol) in MeOH (0.2 mL) dropwise. The resulting mixture was stirred for 5 min before it was warmed to room temperature and quenched with $Na_2S_2O_3$ (3 mL, sat. aq.) and water (3 mL). The resulting mixture was extracted with Et_2O (3 × 6 mL), the combined organic layer was washed with $NaHCO_3$ (10 mL, sat. aq.), brine (10 mL), dried (Na_2SO_4) and concentrated under reduced pressure. Flash column chromatography (silica gel, hexanes:EtOAc 4:1) afforded dienone **7f′** as an amorphous yellow solid.

ii) A solution of dienone **7f′** (obtained above) in toluene (1.0 mL) was warmed to reflux and stirred for 4 h before it was cooled to room temperature and concentrated under reduced pressure. Flash column chromatography (silica gel, hexanes:EtOAc 4:1) afforded tetracycle **8f′** (5.0 mg, 61% over two steps) as an amorphous yellow solid. **8f′** (relative stereochemistry arbitrarily assigned): R_f = 0.49 (silica gel, hexanes:EtOAc 1:1); IR (film) ν_{max} 3469, 2956, 1747, 1464, 1239, 762 cm^{-1}; ^1H NMR (400 MHz, $CDCl_3$): δ 7.10 (d, J = 7.4 Hz, 1H), 6.96 (d, J = 7.4 Hz, 1H), 6.20 (d, J = 7.4 Hz, 1H), 4.69 (br s, 1H), 3.87 (s, 3H), 3.81 (s, 3H), 3.38 (s, 6H), 3.04–3.01 (m, 1H), 2.57–2.46 (m, 1H), 2.28 (t, J = 11.9 Hz, 1H), 1.87 (d, J = 13.5 Hz, 1H), 1.77 (m, 1H), 1.51 (s, 3H), 1.53–1.46 (m, 1H) 1.00–0.95 ppm (m, 1H); ^{13}C NMR (101 MHz, $CDCl_3$): δ 201.5, 152.3, 147.3, 135.9, 132.1, 127.1, 125.4, 125.0, 113.0, 95.0, 67.8, 59.9, 58.8, 55.8, 50.8, 49.2, 37.6, 36.2, 32.2, 28.7, 20.8 ppm; HRMS calcd. For $C_{21}H_{26}O_6Na^+$ $[M + Na]^+$ 397.1622, found 397.1623.

^{1}H NMR (400 MHz, CDCl$_3$) Comparison Between Dienones 7f and 7f′, and Between Intramolecular Diels-Alder Products 8f and 8f′

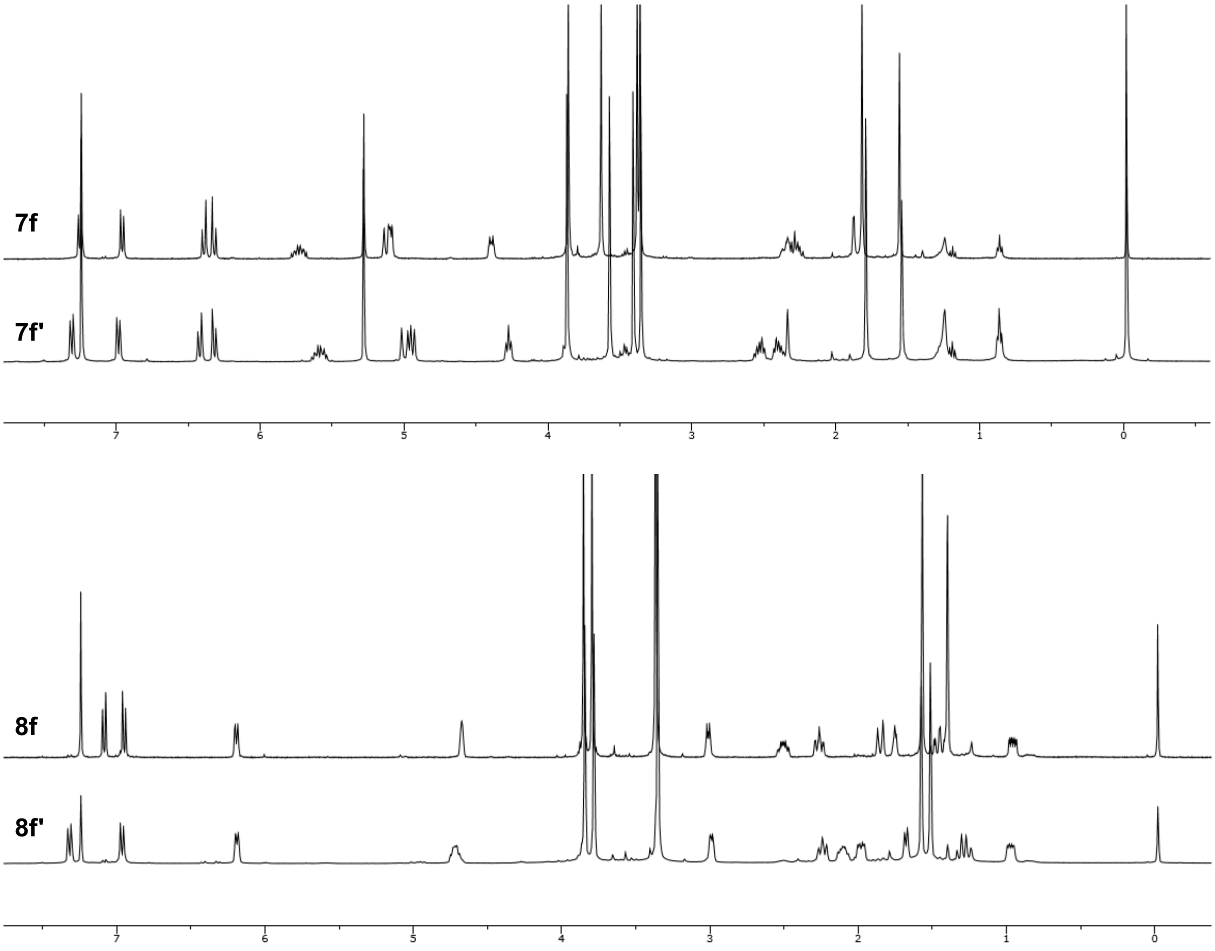

Ketone 9f

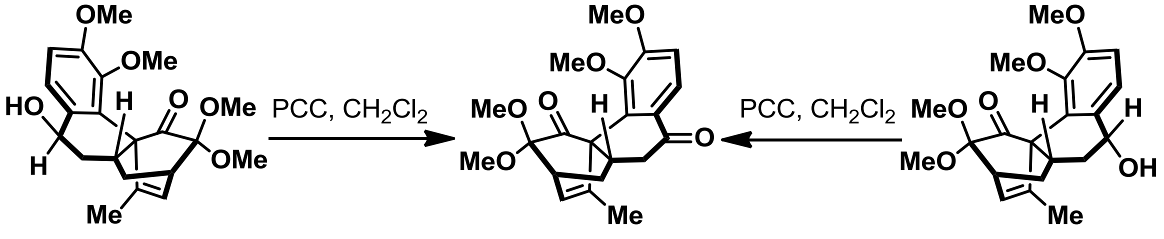

8f' 9f 8f

To a stirred solution of alcohols **8f** and **8f'** (5.2 mg, 14 μmol) in CH_2Cl_2 (0.5 mL) at room temperature was added pyridinium chlorochromate (8.9 mg, 41 μmol). The resulting mixture was stirred for 3 h before it was directly subjected to flash column chromatography (silica gel, hexanes:EtOAc 2:1) afforded ketones **9f** (3.5 mg, 68%) as an amorphous clear solid. **9f**: R_f =

0.76 (silica gel, hexanes:EtOAc 1:1); IR (film) ν_{max} 3053, 2987, 1738, 1712 1485, 747 cm^{-1}; ^{1}H NMR (400 MHz, $CDCl_3$): δ 7.89 (d, *J* = 8.7 Hz, 1H), 7.02 (d, *J* = 8.7 Hz, 1H), 6.31 (d, *J* = 7.2 Hz, 1H), 3.94 (s, 3H), 3.84 (s, 3H), 3.38 (s, 6H), 3.09 (d, *J* = 6.1 Hz, 1H), 2.60–2.50 (m, 1H), 2.46 (d, *J* = 16.5 Hz, 1H), 2.34–2.18 (m, 1H), 2.35–2.24 (m, 1H), 1.40 (s, 3H), 1.03–0.99 (m, 1H); ^{13}C NMR (101 MHz, $CDCl_3$): δ 199.8, 195.7, 157.0, 146.4, 134.7, 132.5, 126.8, 126.2, 123.8, 112.0, 94.9, 60.1, 58.6, 55.7, 50.9, 49.2, 42.7, 37.6, 28.4, 20.8 ppm; HRMS calcd. For $C_{21}H_{24}O_6Na^+$ $[M + Na]^+$ 395.1465, found 395.1464.

Tetracycles 8 and 8′

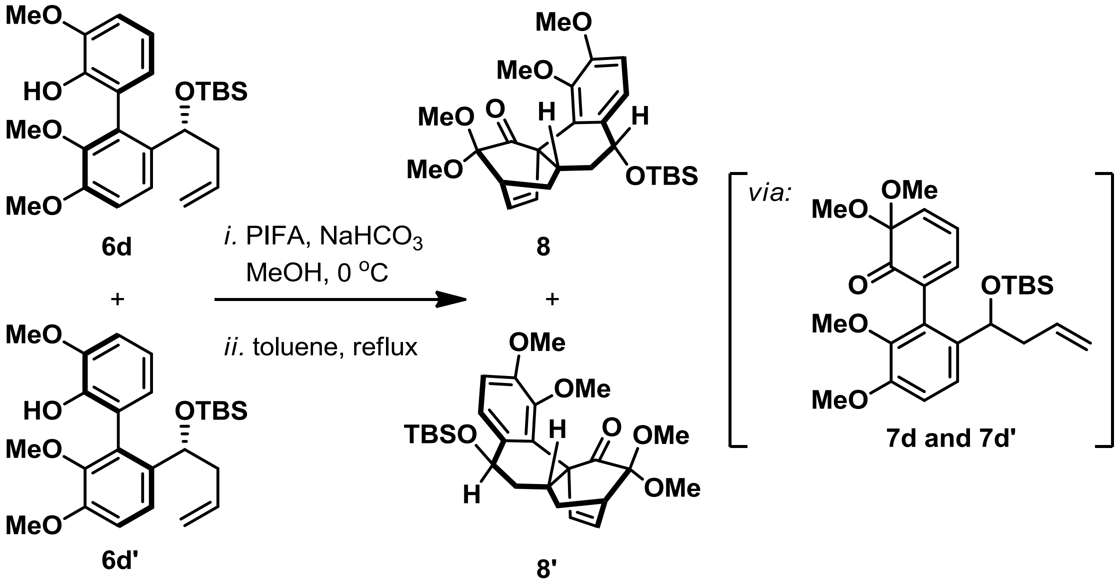

(i) To a stirred solution of phenol **6d** and **6d′** (8.50 g, 19.1 mmol) in MeOH (300 mL) at 0 °C was added PIFA (9.40 g, 21.9 mmol) and $NaHCO_3$ (15.0 g, 179 mmol). The resulting mixture was stirred for 15 min before it was warmed to room temperature and quenched with $Na_2S_2O_3$ (100 mL, sat. aq.) and water (100 mL). The resulting mixture was extracted with Et_2O (3 × 75 mL), the combined organic layer was washed with $NaHCO_3$ (100 mL, sat. aq.), brine (100 mL), dried (Na_2SO_4) and concentrated under reduced pressure. Flash column chromatography (silica gel, CH_2Cl_2:Et_2O 15:1) afforded dienones **7d** and **7d′** as an orange amorphous solid.

(ii) Dienones **7d** and **7d′** (obtained above) was redissolved in toluene (550 mL), and warmed to reflux and stirred for 1 h before it was cooled to room temperature and concentrated under reduced pressure. Flash column chromatography (silica gel, hexanes:EtOAc 10:1) afforded tetracycles **8** and **8′** (~1:1 based on ^{1}H NMR analysis, 6.57 g, 72% over two steps) as an amorphous yellow solid. **8**+**8′**: R_f = 0.59 (silica gel, hexanes:EtOAc 7:3); IR (film) ν_{max} 3154, 2983, 1702, 1210, 1052, 723 cm^{-1}; ^{1}H NMR (400 MHz, $CDCl_3$): δ 7.22 (d, *J* = 8.6 Hz, 0.5H), 6.92–6.85 (m, 1.5H), 6.42–6.38 (m, 1H), 5.82 (d, *J* = 8.1 Hz, 0.4H), 5.73 (d, *J* = 8.1 Hz, 0.6H), 4.76–4.66 (m, 1H), 3.86 (s, 1.8H), 3.85 (s, 1.2H), 3.81 (s, 1.8H), 3.79 (s, 1.2H), 3.39 (s, 3.6H), 3.38 (s, 2.4H), 3.10 (br s, 1H), 2.74–2.66 (m, 1H), 2.27–2.25 (m, 1H), 2.22–2.13 (m, 1H), 1.88–1.80 (m, 0.4H), 1.73 (dt, *J* = 13.4, 2.7 Hz, 0.6H), 1.54–1.45 (m, 1H), 0.95 (s, 3.6H), 0.84 (s, 5.4H), 0.18 (s, 1.2H), 0.12 (s, 1.2H), 0.05 (s, 1.8H), 0.00 ppm (s, 1.8H); ^{13}C NMR (101

MHz, $CDCl_3$): δ 199.7, 199.5, 151.9, 151.4, 147.0, 146.7, 134.6, 132.2, 131.4, 131.2, 130.8, 129.1, 128.9, 128.5, 128.2, 125.0, 121.1, 112.6, 112.1, 94.7, 94.7, 70.0, 68.2, 60.2, 60.1, 55.9, 55.8, 55.7, 55.4, 50.8, 50.7, 49.3, 49.3, 38.2, 37.9, 37.8, 37.6, 34.5, 31.6, 29.7, 28.5, 28.2, 25.9, 25.8, 22.6, 18.2, 18.0, 14.1, −4.1, −4.2, −4.4, −4.8 ppm; HRMS calcd. For $C_{26}H_{38}O_6SiNa^+$ [M + Na]$^+$ 497.2330, found 497.2333.

Ketones 8b and 8b′

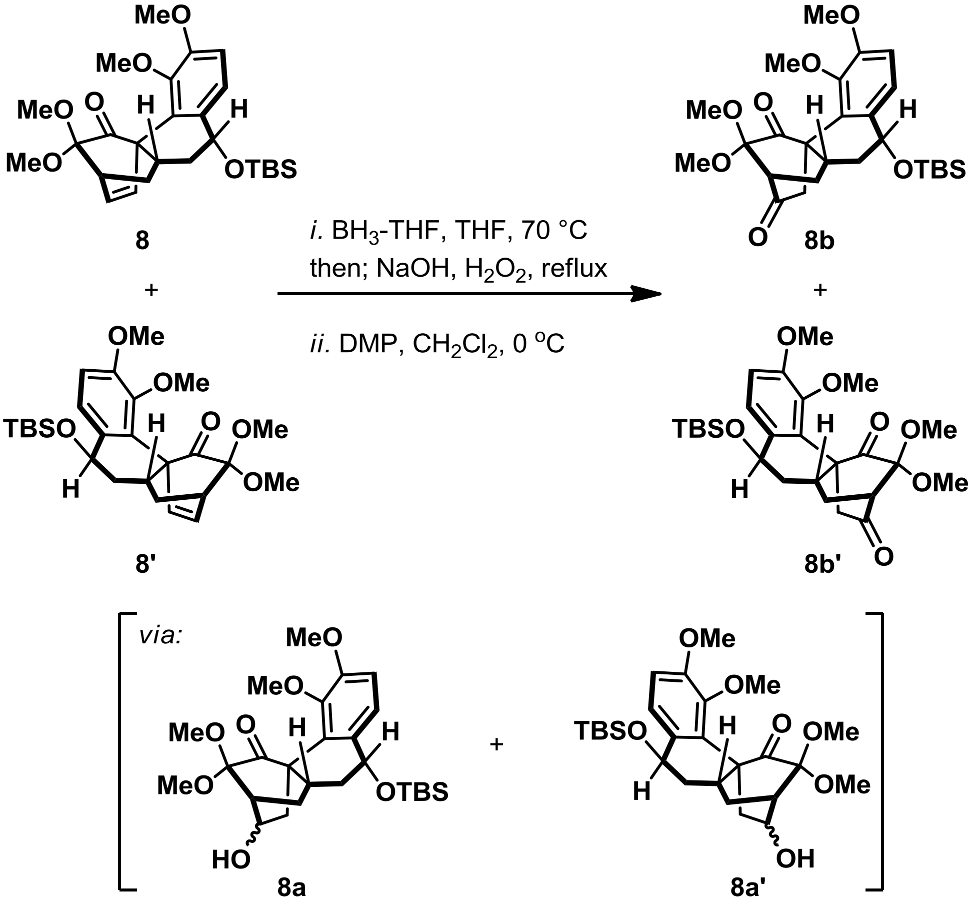

(i) To a stirred solution of alkenes **8** and **8′** (5.40 g, 11.4 mmol) in THF (250 mL) at −78 °C was added borane tetrahydrofuran complex (1.0 M in THF, 50.0 mL, 50.0 mmol). The resulting mixture was warmed to 70 °C and stirred for 4 h before it was cooled to 0 °C and treated with NaOH (2.0 N aq., 21.0 mL, 42.0 mmol) and H_2O_2 (34.5% aq., 21.0 mL, 210 mmol). The resulting mixture was warmed to reflux and stirred for 4 h before it was cooled to room temperature and quenched with NH_4Cl (100 mL, sat. aq.) and water (40 mL). The layers were separated and the aqueous layer was extracted with ethyl acetate (3 × 100 mL), the

combined organic layer was washed with water (50 mL), brine (50 mL), dried (Na_2SO_4) and concentrated under reduced pressure. Flash column chromatography (silica gel, hexanes:EtOAc 4:1) afforded alcohols **8a** and **8a′** (3.40 g, 61%) as an amorphous white solid. **8a**+**8a′**: R_f = 0.40 (silica gel, hexanes:EtOAc 1:1); HRMS calcd. For $C_{26}H_{40}O_7SiNa^+$ [M + Na]$^+$ 515.2436, found 515.2434.

(ii) To a stirred solution of alcohols **8a** and **8a′** (4.70 g, 9.54 mmol) in CH_2Cl_2 (470 mL) at 0 °C was added Dess-Martin periodinane (12.5 g, 29.5 mmol) portion-wise. The resulting mixture was warmed to room temperature and stirred for 3 h before it was quenched with $Na_2S_2O_3$ (40 mL, sat. aq.) and water (40 mL). The layers were separated and the aqueous layer was extracted with CH_2Cl_2 (3 × 100 mL), the combined organic layer was washed with water (50 mL), brine (50 mL), dried (Na_2SO_4) and concentrated under reduced pressure. Flash column chromatography (silica gel, hexanes:EtOAc 9:1) afforded ketones **8b** and **8b′** (4.24 g, 91%) as an amorphous yellow solid. **8b**+**8b′**: R_f = 0.76 (silica gel, hexanes:EtOAc 1:1); IR (film) ν_{max} 3456, 3154, 2970, 1710, 1200, 725 cm^{-1}; ^{1}H NMR (400 MHz, $CDCl_3$): δ 7.20 (d, *J* = 8.1 Hz, 0.5H), 6.91 (dd, *J* = 8.0 Hz, 0.5H), 6.92 (s, 1H), 4.72 (dd, *J* = 5.7 Hz, 0.5H), 4.67 (br t, *J* = 2.5 Hz, 0.5H), 3.86–3.76 (m, 4.5H), 3.70 (s, 1.2H), 3.56 (s, 0.3H), 3.43–3.37 (m, 6H), 3.30 (d, *J* = 20.5 Hz, 0.5H), 3.08 (br s, 0.25H), 3.06–2.96 (m, 0.25H), 2.91 (d, *J* = 19.3 Hz, 0.25H), 2.79 (br s, 0.25H), 2.71 (d, *J* = 20.6 Hz, 0.5H), 2.51–2.24 (m, 2H), 2.02–1.92 (m, 1H), 1.88–1.76 (m, 1H), 1.50–1.42 (m, 1H), 1.35–1.24 (m, 1H), 0.94 (s, 5H), 0.80 (s, 4H), 0.17 (s, 1.7H), 0.11 (s, 1.7H), 0.10 (s, 1.3H), 0.03 ppm (s, 1.3H); ^{13}C NMR (126 MHz, $CDCl_3$): δ 206.9, 204.3, 201.4, 196.0, 151.4, 147.0, 146.0, 133.6, 132.2, 126.6, 124.9, 123.1, 121.8, 113.0, 112.9, 100.0, 97.5, 96.7, 80.0, 69.5, 68.9, 67.5, 60.0, 59.8, 58.4, 55.8, 55.7, 51.9, 51.5, 50.7, 50.0, 49.9, 49.6, 41.7, 40.9, 36.6, 36.1, 34.3, 33.8, 31.3, 28.3, 25.9, 25.8, 24.9, 18.2, 17.9, −0.01, −4.1, −4.5, −4.8 ppm; HRMS calcd. For $C_{26}H_{38}O_7SiNa^+$ [M + Na]$^+$ 513.2279, found 513.2281.

Ketones 8b and 8b′

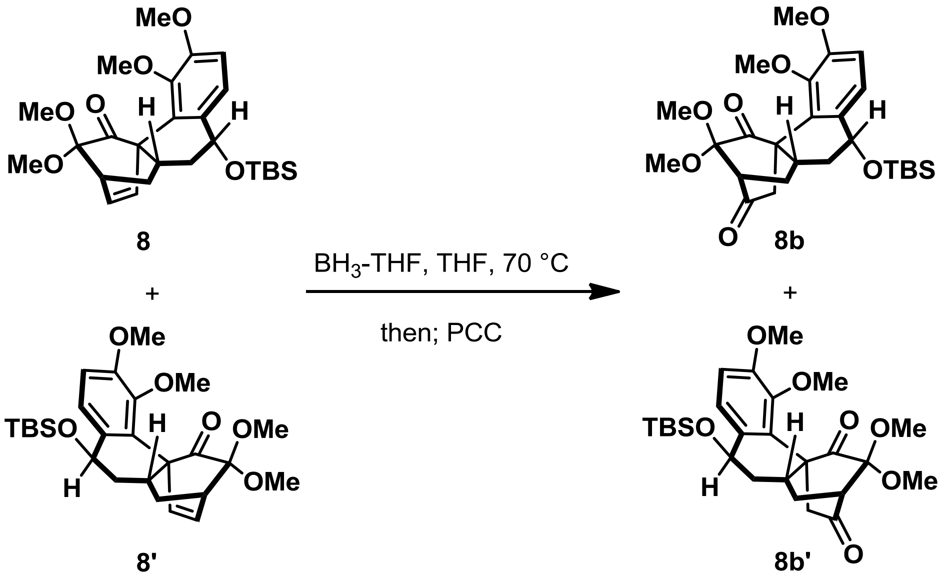

To a stirred solution of alkenes **8** and **8′** (98.0 mg, 0.21 mmol) in THF (2.0 mL) at –78 °C was added borane tetrahydrofuran complex (1.0 M in THF, 1.03 mL, 1.03 mmol). The resulting mixture was warmed to 70 °C and stirred for 4 h before it was cooled to room temperature and treated with PCC (223 mg, 1.03 mmol). The resulting mixture was stirred for 5 h before it was filtered through Celite® and concentrated under reduced pressure. Flash column chromatography (silica gel, hexanes:Et_2O 1:1) afforded ketones **8b** and **8b′** (42.0 mg, 41%) as an amorphous white solid. All physical data of ketone **8b** and **8b′** are identical to those obtained from stepwise hydroboration of alkenes **8** and **8′** followed by DMP oxidation.

Alkene 9

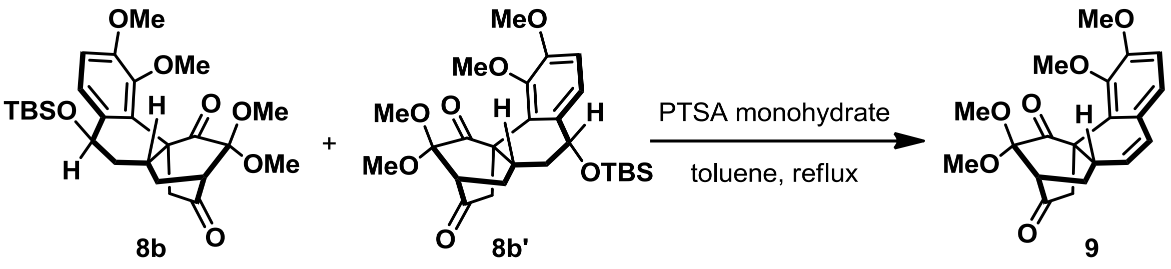

To a stirred solution of TBS ethers **8b** and **8b′** (4.20 g, 8.56 mmol) in toluene (350 mL) at room temperature was added *p*-toluenesulfonic acid monohydrate (1.63 g, 8.57 mmol). The resulting mixture was warmed to reflux and stirred for 20 min before it was cooled to room

temperature and quenched with $NaHCO_3$ (40 mL, sat. aq.) and water (40 mL). The layers were separated and the aqueous layer was extracted with ethyl acetate (3 × 80 mL), the combined organic layer was washed with water (50 mL), brine (50 mL), dried (Na_2SO_4) and concentrated under reduced pressure. Flash column chromatography (silica gel, hexanes:EtOAc 9:1) afforded alkene **9** (2.90 g, 95%) as an amorphous white solid. **9**: R_f = 0.48 (silica gel, hexanes:EtOAc 1:1); IR (film) ν_{max} 3455, 3154, 2924, 1704, 1597, 722 cm^{-1}; ^{1}H NMR (499 MHz, $CDCl_3$): δ 6.82–6.70 (m, 2H), 6.37 (d, *J* = 9.4 Hz, 1H), 5.43 (d, *J* = 9.3 Hz, 1H), 3.85 (s, 6H), 3.42 (s, 3H), 3.39 (s, 3H), 3.14–3.04 (m, 2H), 2.77 (d, *J* = 19.5 Hz, 1H), 2.67 (d, *J* = 21.1 Hz, 1H), 2.53–2.49 (m, 1H), 1.77–1.66 ppm (m, 1H); ^{13}C NMR (101 MHz, $CDCl_3$): δ 206.3, 202.8, 152.9, 145.9, 128.0, 126.8, 125.7, 125.6, 123.1, 111.8, 97.5, 59.8, 55.7, 52.4, 51.9, 51.1, 49.3, 38.6, 37.4, 24.7 ppm; HRMS calcd. For $C_{20}H_{22}O_6Na^+$ $[M + Na]^+$ 381.1309, found 381.1308.

Oxime 10

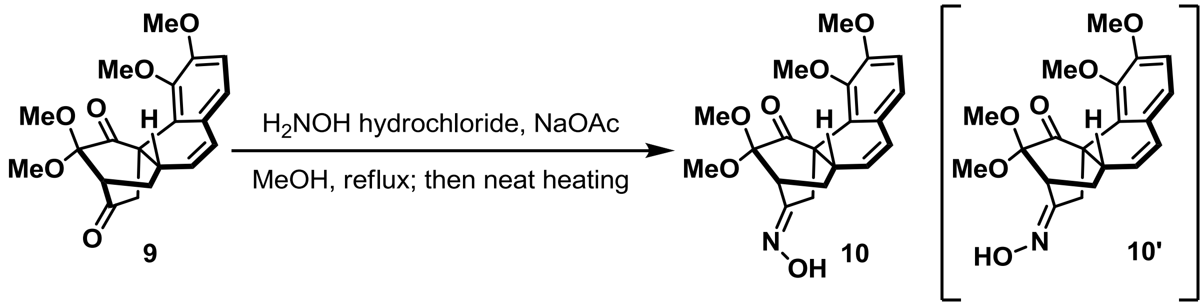

To a stirred solution of alkene **9** (3.20 g, 8.93 mmol) in MeOH (28.0 mL) at room temperature was added hydroxylamine hydrochloride (1.86 g, 26.8 mmol) and NaOAc (2.20 g, 26.8 mmol). The resulting mixture was warmed to reflux and gradually allow the reaction mixture to evaporate to dryness, and the resulting solid residue was heated for 16 h before it was cooled to room temperature and quenched with $NaHCO_3$ (40 mL, sat. aq.) and MeOH (40 mL). The resulting mixture was extracted with ethyl acetate (3 × 100 mL), the combined organic layer was washed with water (50 mL), brine (50 mL), dried (Na_2SO_4) and concentrated under reduced pressure. Flash column chromatography (silica gel, hexanes:EtOAc 4:1) afforded oxime **10** (3.0 g, 90%) as an amorphous yellow solid.

Performing the reaction without "neat-heating" gave rise to a mixture of oxime **10** and its geometric isomer **10′**.

10: R_f = 0.24 (silica gel, hexanes:EtOAc 1:1); IR (film) ν_{max} 3707, 3456, 2926, 1705, 740 cm^{-1}; ^1H NMR (400 MHz, $CDCl_3$): δ 7.89 (s, 1H), 6.81 (s, 2H), 6.35 (d, *J* = 9.3 Hz, 1H), 5.43 (d, *J* = 9.4 Hz, 1H), 3.85 (s, 6H), 3.41 (s, 6H), 3.20–3.15 (m, 1H), 3.01–2.95 (m, 1H), 2.85 (s, 2H), 2.45 (t, *J* = 12.1 Hz, 1H), 1.70–1.64 ppm (m, 1H); ^{13}C NMR (101 MHz, $CDCl_3$): δ 203.7, 157.9, 152.9, 146.3, 127.7, 127.1, 126.6, 126.2, 122.9, 111.8, 97.7, 59.8, 55.7, 51.3, 50.9, 49.4, 41.6, 37.6, 26.5, 26.3 ppm; HRMS calcd. For $C_{20}H_{23}NO_6Na^+$ $[M + Na]^+$ 396.1418, found 396.1416.

10′: R_f = 0.37 (silica gel, hexanes:EtOAc 1:1); IR (film) ν_{max} 3705, 3457, 2926, 1702, 733 cm^{-1}; ^1H NMR (499 MHz, $CDCl_3$): δ 7.41 (s, 1H), 6.82 (dd, *J* = 2.5, 0.9 Hz, 2H), 6.36 (dd, *J* = 9.3, 2.2 Hz, 1H), 5.46 (dd, *J* = 9.3, 2.2 Hz, 1H), 4.17 (t, *J* = 3.0 Hz, 1H), 3.86 (s, 6H), 3.45 (s, 3H), 3.44 (s, 3H), 3.12–2.99 (m, 1H), 2.84 (d, *J* = 18.0 Hz, 1H), 2.66 (d, *J* = 17.4 Hz, 1H), 2.43–2.38 (m, 1H), 1.70–1.60 ppm (m, 1H); ^{13}C NMR (101 MHz, $CDCl_3$): δ 204.1, 156.8, 152.9, 146.3, 127.7, 127.2, 126.5, 126.5, 123.0, 111.7, 98.1, 59.9, 55.7, 51.7, 51.2, 49.5, 37.7, 35.5, 28.7, 25.5 ppm; HRMS calcd. For $C_{20}H_{23}NO_6Na^+$ $[M + Na]^+$ 396.1418, found 396.1416.

Tosylate 11

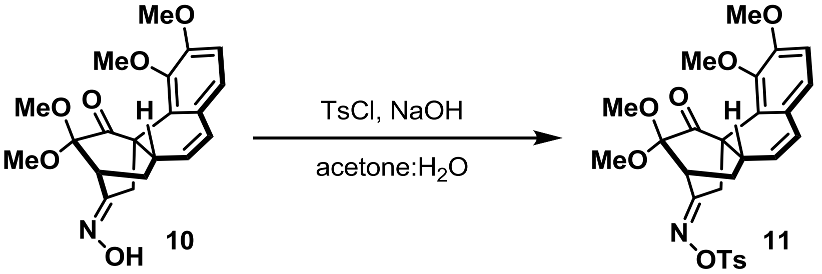

To a stirred solution of oxime **10** (5.10 g, 13.7 mmol) in acetone/H_2O (1:1, 260 mL) at room temperature was added NaOH (1.37 g, 34.3 mmol) and TsCl (3.91 g, 20.5 mmol). The resulting mixture was stirred for 2 h before it was quenched with $NaHCO_3$ (100 mL, sat. aq.) and water (60 mL). The resulting mixture was extracted with ethyl acetate (3 × 100 mL), the combined organic layer was washed with water (70 mL), brine (70 mL), dried (Na_2SO_4) and concentrated under reduced pressure. Flash column chromatography (silica gel,

hexanes:EtOAc 10:1) afforded tosylate **11** (7.02 g, 97%) as an amorphous yellow solid. **11**: R_f = 0.43 (silica gel, hexanes:EtOAc 1:1); IR (film) ν_{max} 3695, 3457, 3154, 2918, 1704, 723 cm^{-1}; ^1H NMR (499 MHz, $CDCl_3$): δ 7.83 (d, *J* = 8.3 Hz, 2H), 7.32 (d, *J* = 8.3 Hz, 2H), 6.82 (s, 2H), 6.34 (dd, *J* = 9.3, 3.3 Hz, 1H), 5.33 (dd, *J* = 9.3, 2.3 Hz, 1H), 3.86 (s, 3H), 3.82 (s, 3H), 3.39 (s, 3H), 3.24 (s, 3H), 3.23–3.20 (m, 1H), 3.08–2.98 (m, 1H), 2.85 (d, *J* = 3.4 Hz, 2H), 2.49–2.40 (m, 1H), 2.48 (s, 3H), 1.57–1.51 ppm (m, 1H); ^{13}C NMR (101 MHz, $CDCl_3$): δ 202.6, 167.3, 153.0, 146.2, 144.7, 132.9, 129.5, 128.6, 128.0, 126.9, 125.4, 125.4, 123.2, 112.1, 97.1, 59.8, 55.8, 50.6, 50.6, 49.8, 41.8, 37.2, 27.5, 26.0, 21.7 ppm; HRMS calcd. For $C_{27}H_{29}NO_8SNa^+$ $[M + Na]^+$ 550.1506, found 550.1503.

Lactam 12

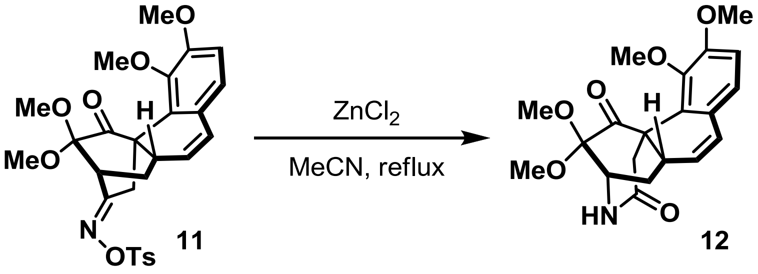

To a stirred solution of tosylate **11** (388 mg, 0.74 mmol) in MeCN (14.4 mL) at room temperature was added $ZnCl_2$ (200 mg, 1.47 mmol). The resulting mixture was warmed to reflux and stirred for 4 h before additional $ZnCl_2$ (200 mg, 1.47 mmol) was added. The resulting mixture was refluxed for further 4 h before it was cooled down to room temperature, quenched with sodium potassium tartrate (30 mL, sat. aq.) and diluted with EtOAc (30 mL), and stirred vigorously for 1 h. The layers were separated and the aqueous layer was extracted with ethyl acetate (3 × 70 mL). The combined organic layer was washed with water (70 mL), brine (70 mL), dried (Na_2SO_4) and concentrated under reduced pressure. Flash column chromatography (silica gel, EtOAc:MeOH 10:1) afforded lactam **12** (200 mg, 73%) as a brown amorphous solid. **12**: R_f = 0.32 (silica gel, EtOAc); IR (film) ν_{max} 3695, 3457, 3154, 2918, 1704, 723 cm^{-1}; ^1H NMR (400 MHz, $CDCl_3$): δ 7.06 (d, *J* = 7.6 Hz, 1H), 6.86–6.76 (m, 2H), 6.38 (dd, *J* = 9.4, 2.5 Hz, 1H), 5.48 (dd, *J* = 9.3, 2.1 Hz, 1H), 3.92 (s, 3H), 3.85 (s, 3H), 3.72–3.69 (m, 1H), 3.41 (s, 3H), 3.39 (s, 3H), 3.09–2.97 (m, 1H), 2.93–2.78 (m, 2H), 2.72–

2.68 (m, 1H), 2.10–2.04 ppm (m, 1H); ^{13}C NMR (126 MHz, $CDCl_3$): δ 200.5, 174.2, 153.1, 147.1, 128.1, 128.0, 126.7, 123.6, 122.5, 112.2, 98.9, 60.3, 55.8, 54.1, 50.8, 49.8, 49.3, 37.3, 37.1, 32.4 ppm; HRMS calcd. For $C_{20}H_{23}NO_6Na^+$ $[M + Na]^+$ 396.1418, found 396.1417.

Lactam 12′

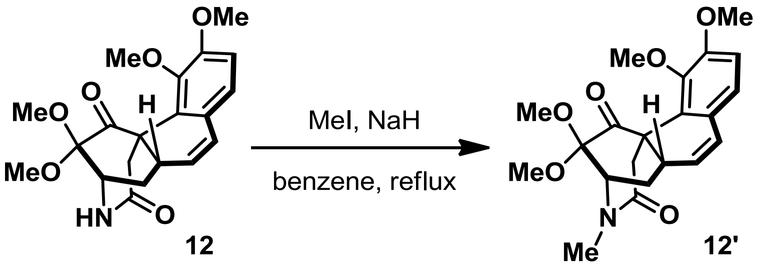

To a stirred solution of lactam **12** (360 mg, 0.96 mmol) in benzene (15.0 mL) at room temperature was added NaH (160 mg, 6.67 mmol) and MeI (0.59 mL, 9.48 mmol). The resulting mixture was warmed to reflux and stirred for 10 h before it was cooled to room temperature and quenched with MeOH (15 mL) and brine (15 mL). The layers were separated and the aqueous layer was extracted with ethyl acetate (3 × 50 mL), the combined organic layer was washed with water (50 mL), brine (50 mL), dried (Na_2SO_4) and concentrated under reduced pressure. Flash column chromatography (silica gel, EtOAc:MeOH 10:1) afforded lactam **12′** (299 mg, 80%) as a brown amorphous solid. **12′**: R_f = 0.43 (silica gel, EtOAc); IR (film) ν_{max} 3646, 3464, 2979, 1738, 1236, 786 cm^{-1}; ^{1}H NMR (400 MHz, $CDCl_3$): δ 6.81 (s, 2H), 6.37 (d, J = 9.3 Hz, 1H), 5.46 (d, J = 9.3 Hz, 1H), 3.88 (s, 3H), 3.84 (s, 3H), 3.70 (d, J = 7.6 Hz, 1H), 3.46 (s, 3H), 3.39 (s, 3H), 3.12 (s, 3H), 3.00 (t, J = 10.1 Hz, 1H), 2.94 (d, J = 18.6 Hz, 1H), 2.84 (d, J = 18.6 Hz, 1H), 2.71–2.52 (m, 1H), 2.13–2.01 ppm (m, 1H); ^{13}C NMR (101 MHz, $CDCl_3$): δ 202.2, 170.7, 153.0, 146.5, 128.6, 128.1, 126.7, 124.3, 122.8, 112.0, 99.5, 60.0, 58.8, 55.8, 54.2, 51.6, 49.8, 38.1, 37.9, 37.6, 31.4 ppm; HRMS calcd. For $C_{21}H_{25}NO_6Na^+$ $[M + Na]^+$ 410.1574, found 410.1575.

Tertiary Amine 12a

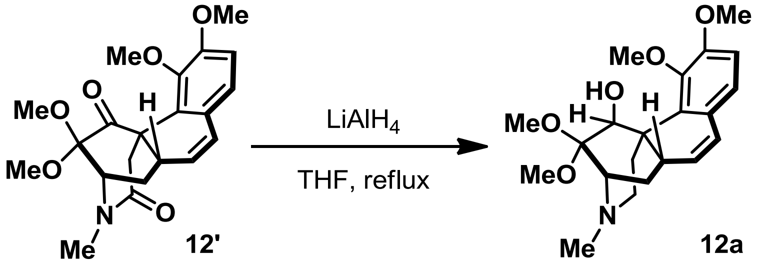

To a stirred solution of $LiAlH_4$ (300 mg, 7.91 mmol) in THF (120 mL) at 0 °C was added a solution of lactam **12'** (300 mg, 0.77 mmol) in THF (30.0 mL). The resulting mixture was warmed to reflux and stirred for 2 h before it was cooled to room temperature, quenched with sodium potassium tartrate (80 mL, sat. aq.) and diluted with EtOAc (50 mL), and stirred vigorously for 1 h. The layers were separated and the aqueous layer was extracted with ethyl acetate (3 × 80 mL), the combined organic layer was washed with water (50 mL), brine (50 mL), dried (Na_2SO_4) and concentrated under reduced pressure. Flash column chromatography (silica gel, EtOAc:MeOH 8:1) afforded tertiary amine **12a** (212 mg, 73%) as a brown amorphous solid. **12a**: R_f = 0.43 (silica gel, EtOAc:MeOH 1:1); IR (film) ν_{max} 3653, 3276, 2824, 1261, 1060, 684 cm^{-1}; ^1H NMR (400 MHz, CD_3OD): δ 6.80 (d, J = 8.3 Hz, 1H), 6.71 (d, J = 8.3 Hz, 1H), 6.24 (dd, J = 9.4, 3.2 Hz, 1H), 5.45 (dd, J = 9.3, 2.2 Hz, 1H), 5.08 (s, 1H), 3.95 (s, 3H), 3.82 (s, 3H), 3.40 (s, 3H), 3.36 (s, 3H), 3.15 (d, J = 7.9 Hz, 1H), 2.93 (t, J = 10.4 Hz, 1H), 2.80–2.69 (m, 1H), 2.59–2.52 (m, 1H), 2.48 (s, 3H), 2.24–2.08 (m, 2H), 1.74 (dd, J = 14.6, 10.5 Hz, 1H), 1.61 ppm (m, 1H); ^{13}C NMR (101 MHz, CD_3OD): δ 154.1, 148.1, 136.1, 128.9, 128.8, 127.6, 123.7, 111.7, 102.8, 75.0, 60.8, 60.8, 56.1, 51.5, 49.5, 49.4, 46.8, 33.6, 31.8, 31.6 ppm; HRMS calcd. For $C_{21}H_{31}NO_5^+$ $[M + H]^+$ 376.2118, found 376.2119.

Ammonium Salt 13

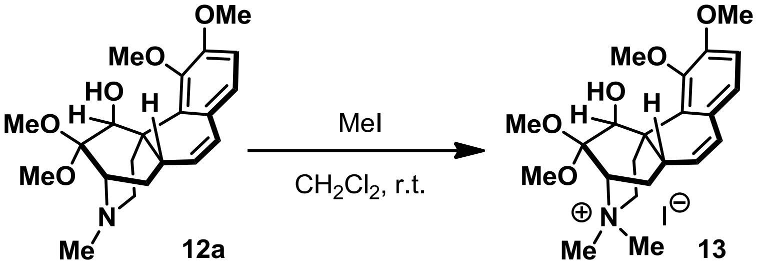

To a stirred solution of tertiary amine **12a** (430 mg, 1.15 mmol) in CH_2Cl_2 (10.0 mL) at room temperature was added MeI (2.00 mL, 32.1 mmol). The resulting mixture was stirred for 10 h before it was concentrated under reduced pressure to afford the crude ammonium salt **13** (500 mg, 84%) as a brown solid, which was used directly without further purification. **13**: R_f = 0.05 (silica gel, EtOAc:MeOH 1:1); IR (film) ν_{max} 3287, 2824, 1457, 1257, 1060, 710 cm^{-1}; ^1H NMR (400 MHz, CD_3OD): δ 6.88 (d, *J* = 8.2 Hz, 1H), 6.78 (d, *J* = 8.3 Hz, 1H), 6.36 (d, *J* = 9.5 Hz, 1H), 5.49 (d, *J* = 9.5 Hz, 1H), 5.23 (s, 1H), 4.12 (m, 1H), 4.01 (s, 3H), 3.84 (s, 3H), 3.68 (td, *J* = 14.0, 3.4 Hz, 1H), 3.54 (s, 3H), 3.53 (s, 3H), 3.44 (s, 3H), 3.28–3.26 (m, 1H), 3.22 (s, 3H), 3.16–3.05 (m, 1H), 2.51–2.42 (m, 2H), 2.17 (dd, *J* = 17.4, 9.0 Hz, 1H), 1.71 ppm (d, *J* = 16.3 Hz, 1H); ^{13}C NMR (101 MHz, CD_3OD): δ 154.3, 147.8, 134.1, 128.6, 128.4, 127.2, 123.9, 112.4, 101.6, 73.8, 71.3, 61.0, 60.6, 58.1, 56.3, 53.8, 50.6, 50.1, 48.5, 31.1, 28.5, 27.8 ppm; HRMS calcd. For $C_{22}H_{32}NO_5^+$ $[M]^+$ 390.2275, found 390.2274.

Preparation of Ammonium salt 13 via Reduction of 12 and Double Methylation

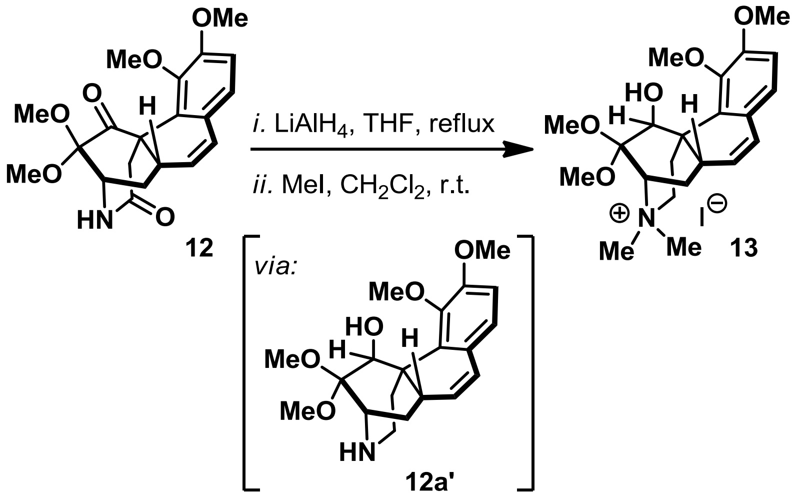

(i) To a stirred solution of $LiAlH_4$ (122 mg, 3.20 mmol) in THF (20 mL) at 0 °C was added a solution of lactam **12** (120 mg, 0.32 mmol) in THF (15 mL). The resulting mixture was warmed to reflux and stirred for 8 h before it was cooled to room temperature, quenched with sodium potassium tartrate (30 mL, sat. aq.) and diluted with EtOAc (30 mL), and stirred vigorously for 1 h. The layers were separated and the aqueous layer was extracted with ethyl acetate (3 × 30 mL), the combined organic layer was washed with water (50 mL), brine (50

mL), dried (Na_2SO_4) and concentrated under reduced pressure.

(ii) To a stirred solution of crude amine **12a′** (obtained above) in CH_2Cl_2 (2.0 mL) at room temperature was added MeI (0.28 mL, 4.40 mmol). The resulting mixture was stirred for 10 h before it was concentrated under reduced pressure afforded crude ammonium salt **13** (70.3 mg, 42% over two steps). All physical data of ammonium salt **13** is identical to those obtained from methylation of tertiary amine **12a**.

Diene 14

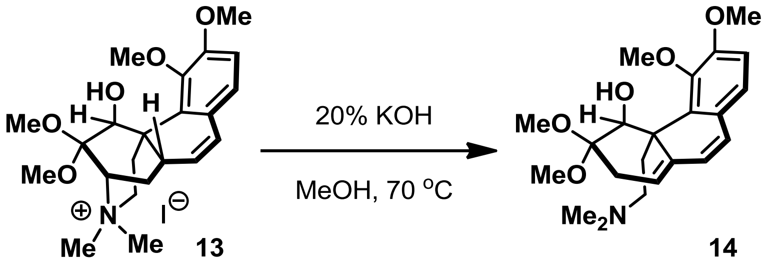

13 14

A stirred solution of ammonium salt **13** (0.100 g, 0.19 mmol) in KOH (20% in MeOH, 5.8 mL) was warmed to 70 °C and stirred for 10 h before it was cooled to room temperature and diluted with water (10 mL). The resulting mixture was extracted with CH_2Cl_2 (3 × 20 mL), the combined organic layer was dried (Na_2SO_4) and concentrated under reduced pressure. Flash column chromatography (silica gel, EtOAc:MeOH 4:1) afforded diene **14** (60.0 mg, 80%) as a brown amorphous solid. **14**: R_f = 0.38 (silica gel, EtOAc:MeOH 1:1); IR (film) ν_{max} 3345, 3043, 1648, 1386, 1123, 822 cm^{-1}; ^1H NMR (400 MHz, CD_3OD): δ 6.82 (d, J = 8.1 Hz, 1H), 6.72 (d, J = 8.3 Hz, 1H), 6.13–6.07 (m, 2H), 5.56 (t, J = 4.2 Hz, 1H), 5.09 (s, 1H), 3.91 (s, 3H), 3.81 (s, 3H), 3.28 (s, 3H), 3.31 (s, 3H), 2.65 (dd, J = 21.1, 4.3 Hz, 1H), 2.44 (dd, J = 20.0, 4.7 Hz, 1H), 2.36–2.30 (m, 1H), 2.18 (td, J = 11.8, 4.1 Hz, 1H), 2.05–2.00 (m, 1H), 2.00 (s, 6H), 2.08 ppm (td, J = 11.1, 4.2 Hz, 1H); ^{13}C NMR (101 MHz, $CDCl_3$): δ 152.4, 147.6, 138.9, 130.7, 128.5, 127.2, 125.1, 123.0, 122.4, 110.6, 100.9, 70.6, 60.2, 55.6, 55.3, 49.0, 48.3, 48.1, 45.4, 45.4, 37.2, 31.4 ppm; HRMS calcd. For $C_{22}H_{32}NO_5^+$ $[M]^+$ 390.2275, found 390.2277.

Ethyl Carbamate 15

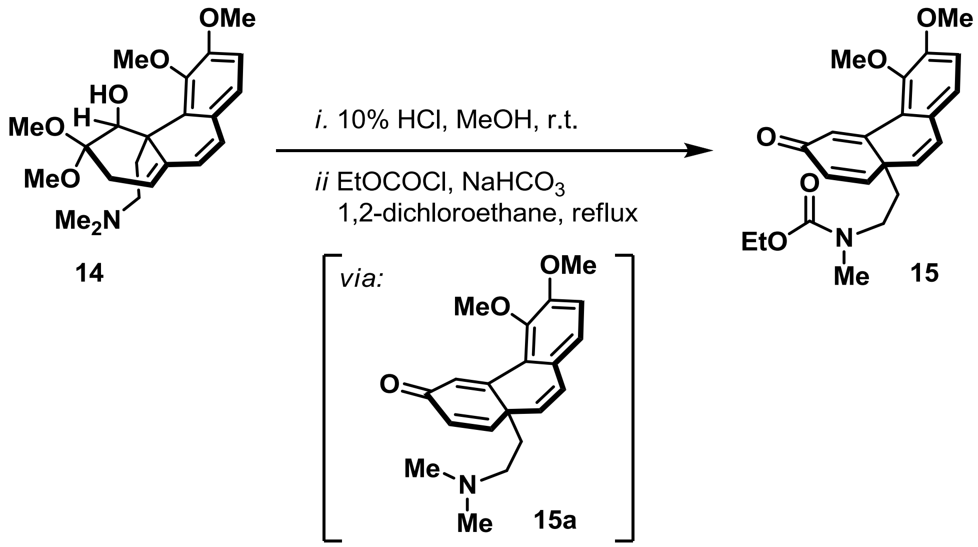

(i) To a stirred solution of dimethyl ketal **14** (220 mg, 0.56 mmol) in MeOH (5.0 mL) at room temperature was added HCl (10% aq., 20 mL). The resulting mixture was stirred for 1 h before it was slowly quenched with $NaHCO_3$ (40 mL, sat. aq.) and water (20 mL). The resulting mixture was extracted with CH_2Cl_2 (3 × 70 mL), the combined organic layer was dried (Na_2SO_4) and concentrated under reduced pressure to afford the crude dienone **15a (**124 mg, 67%) as a dark purple solid, which was used directly without further purification.

(ii) To a stirred solution of crude dienone **15a** (obtained above) in 1,2-dichloroethane (10.0 mL) at room temperature was added $NaHCO_3$ (640 mg, 7.62 mmol) followed by ethyl chloroformate (0.18 mL, 1.89 mmol). The resulting mixture was warmed to reflux and stirred for 2 h before it was cooled to room temperature and quenched with $NaHCO_3$ (40 mL, sat. aq.) and water (30 mL). The resulting mixture was extracted with CH_2Cl_2 (3 × 80 mL), the combined organic layer was dried (Na_2SO_4) and concentrated under reduced pressure. Flash column chromatography (silica gel, hexanes:EtOAc 5:1) afforded ethyl carbamate **15** (136 mg, 93%) as an amorphous yellow solid. **15**: R_f = 0.58 (silica gel, hexanes:EtOAc 2:1); IR (film) ν_{max} 3054, 2987, 1693, 1659, 1422, 909, 743 cm^{-1}; ^1H NMR (400 MHz, $CDCl_3$): δ 6.96 (br s, 1 H), 6.91–6.77 (m, 3H), 6.39 (d, J = 9.8 Hz, 2H), 5.87 (d, J = 9.4 Hz, 1H), 4.01 (br s, 2H), 3.89 (s, 6H), 3.26–2.99 (m, 2H), 2.70 (br s, 3H), 2.09–1.76 (m, 2H), 1.27–1.03 ppm (m, 3H); ^{13}C NMR (101 MHz, $CDCl_3$): δ 186.6, 156.1, 155.7, 153.4, 150.6, 130.1, 130.0, 129.1, 126.7,

126.5, 124.3, 122.7, 113.3, 61.2, 60.8, 55.9, 45.3, 37.4, 37.1, 34.6, 34.1, 14.4 ppm; HRMS calcd. For $C_{22}H_{32}NO_5^+$ $[M]^+$ 406.1625, found 406.1627.

Ketone 18

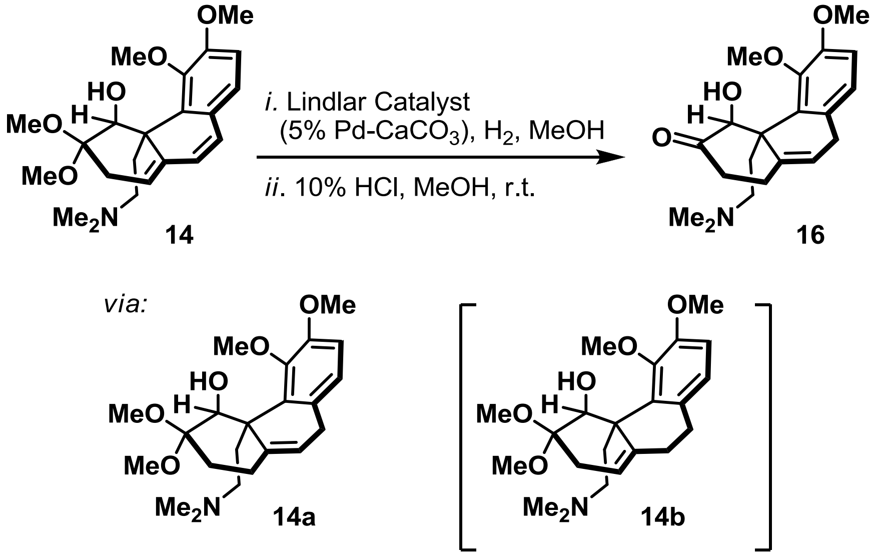

(i) To a stirred solution of diene **14** (168 mg, 0.43 mmol) in MeOH (10 mL) at room temperature was added 5% Pd–$CaCO_3$ (916 mg, 0.43 mmol). The resulting mixture was evacuated and filled with hydrogen (3 ×) and stirred under an atmosphere of H_2 (balloon) for 16 h. The resulting mixture was filtered through Celite® and eluted with MeOH (3 × 8 mL), and concentrated under reduced pressure to afford crude alkene **14a** (as a ~5:1 mixture with 1,2-hydrogenated product **14b** based on ^1H NMR analysis, 154 mg, 91% combined yield) as an amorphous yellow solid, which was used directly without further purification. R_f = 0.18 (silica gel, EtOAc:MeOH 1:1)

(ii) To a stirred solution of the crude alkene **14a** (**14a**:**14b**~5:1, obtained above) in MeOH (5 mL) at room temperature was added HCl (10% aq., 10 mL). The resulting mixture was stirred for 1 h before it was slowly quenched with $NaHCO_3$ (15 mL, sat. aq.) and water (15 mL). The resulting mixture was extracted with CH_2Cl_2 (3 × 50 mL), the combined organic layer was dried (Na_2SO_4) and concentrated under reduced pressure. Flash column chromatography (silica gel, EtOAc:MeOH 4:1) afforded ketone **16** (98.0 mg, 72%) as an amorphous yellow solid. *Note: Due the highly polar nature of dimethylamine ketone **16**, this compound was*

semi-purified by flash column chromatography and subjected directly to the following reaction.

16: R_f = 0.15 (silica gel, EtOAc:MeOH 1:1); IR (film) ν_{max} 3690, 3053, 3005, 1721, 1458, 1260 cm^{-1}; ^{1}H NMR (400 MHz, $CDCl_3$): δ 6.89 (d, *J* = 8.3 Hz, 1H), 6.85 (d, *J* = 8.3 Hz, 1H), 6.11 (br s, 1H), 4.69 (s, 1H), 3.92 (s, 3H), 3.85 (s, 3H), 3.43 (d, *J* = 13.9 Hz, 2H), 3.03–2.92 (m, 1H), 2.74–2.62 (m, 1H), 2.60–2.50 (m, 2H), 2.38–2.30 (m, 2H), 2.17 (s, 6H), 1.85–1.65 ppm (m, 2H); ^{13}C NMR (101 MHz, $CDCl_3$): δ 211.0, 151.1, 147.3, 132.2, 128.8, 127.2, 125.7, 123.6, 112.5, 79.8, 60.2, 55.8, 54.8, 50.7, 44.7, 37.2, 31.3, 29.9, 28.8 ppm; HRMS calcd. For $C_{20}H_{28}NO_4^+$ $[M]^+$ 346.2013, found 346.2011.

16: ^{1}H NMR (400 MHz, $CDCl_3$, semi-purified)

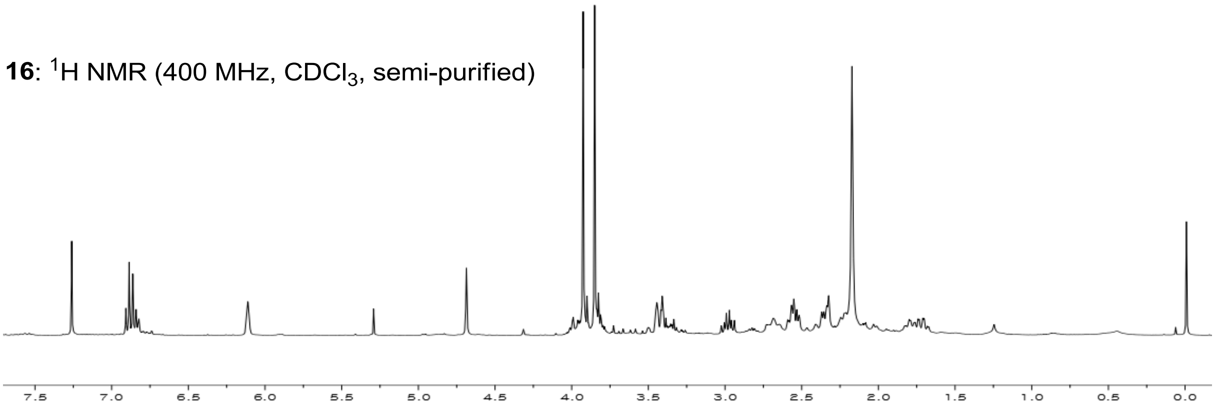

16: ^{13}C NMR (101 MHz, $CDCl_3$, semi-purified)

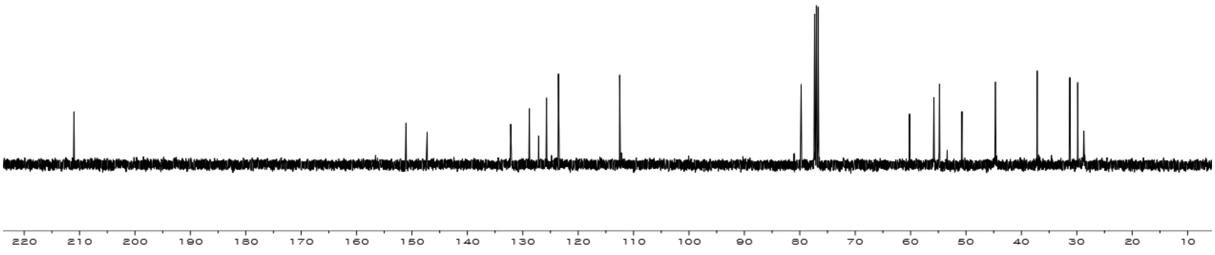

Ketone 17

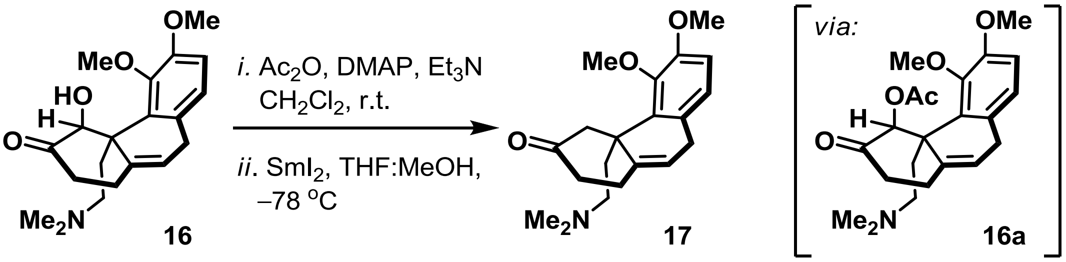

(i) To a stirred solution of ketone **16** (254 mg, 0.74 mmol) in CH_2Cl_2 (5.0 mL) at room temperature was added acetic anhydride (0.70 mL, 7.41 mmol), Et_3N (1.0 mL, 7.17 mmol) and DMAP (9.0 mg, 74 μmol). The resulting mixture was stirred for 4 h before it was quenched with $NaHCO_3$ (5.0 mL, sat. aq.). The resulting mixture was extracted with CH_2Cl_2 (3 × 15 mL), the combined organic layer was dried (Na_2SO_4) and concentrated under reduced pressure to afford crude acetate **16a** as a brown amorphous solid, which was used directly without further purification. R_f = 0.25 (silica gel, EtOAc:MeOH 1:1).

(ii) To a stirred solution of the crude acetate **16a** (obtained above) in anhydrous THF:MeOH (1:1, 10.0 mL) at –78 °C was added SmI_2 (0.1 M in THF) until a blue color persists (~30 mL, 3.0 mmol). The resulting mixture was stirred for 0.5 h before it was quenched with K_2CO_3 (10 mL, sat. aq.). The resulting mixture was extracted with CH_2Cl_2 (4 × 30 mL), the combined organic layer was dried (Na_2SO_4) and concentrated under reduced pressure. Flash column chromatography (silica gel, EtOAc:MeOH 4:1) afforded ketone **17** (189 mg, 78% over two steps) as an amorphous white solid. *Note: Due the highly polar nature of dimethylamine ketone **17**, this compound was semi-purified by flash column chromatography and subjected directly to the following reaction.*

17: R_f = 0.30 (silica gel, EtOAc:MeOH 1:1); IR (film) ν_{max} 3691, 3059, 1707, 1661, 1550, 1262 cm^{-1}; ^1H NMR (400 MHz, $CDCl_3$): δ 6.85 (d, J = 8.1 Hz, 1H), 6.81 (d, J = 8.7 Hz, 1H), 5.90 (s, 1H), 3.90 (s, 3H), 3.84 (s, 3H), 3.53 (d, J = 14.6 Hz, 1H), 3.41 (s, 2H), 2.77–2.65 (m, 2H), 2.63–2.52 (m, 2H), 2.48 (d, J = 14.6 Hz, 1H), 2.47–2.36 (m, 2H), 2.19 (s, 1H), 2.11 (s, 6H), 1.86–1.63 ppm (m, 2H); ^{13}C NMR (101 MHz, $CDCl_3$): δ 210.9, 151.3, 147.8, 135.3, 131.7, 126.6, 123.2, 121.7, 112.0, 60.3, 55.9, 55.4, 53.3, 45.1, 44.3, 42.0, 32.9, 31.2, 30.0, 29.7 ppm; HRMS calcd. For $C_{20}H_{28}NO_3^+$ $[M]^+$ 330.2064, found 330.2065.

17: ^{1}H NMR (400 MHz, $CDCl_3$, semi-purified)

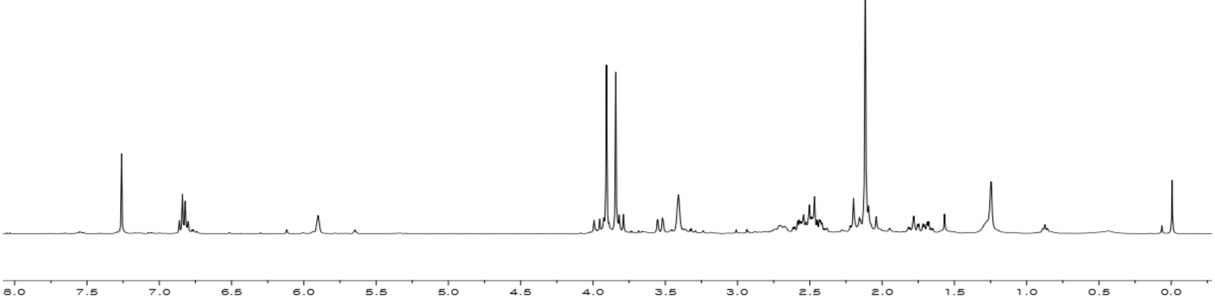

17: ^{13}C NMR (101 MHz, $CDCl_3$, semi-purified)

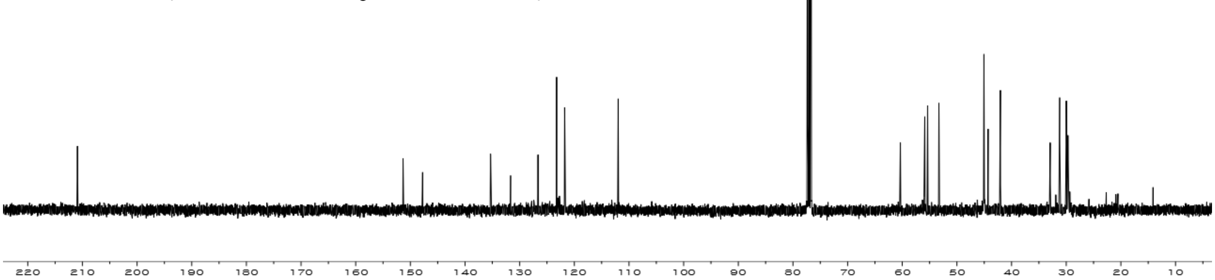

Ketone 18

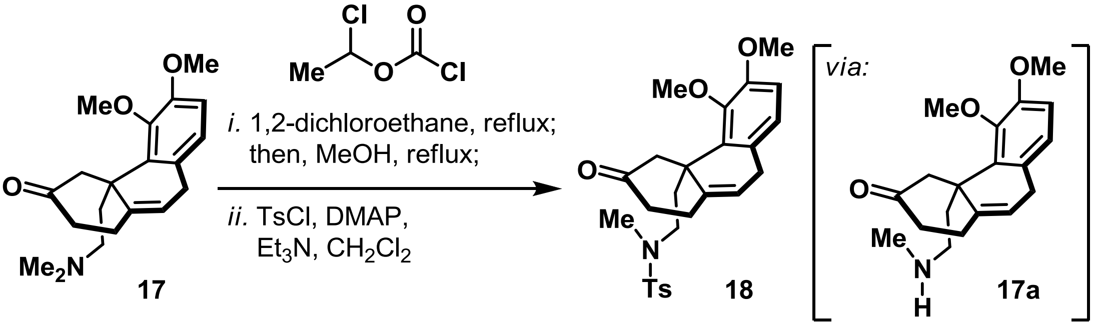

(i) To a stirred solution of dimethyl amine **17** (16.0 mg, 49 μmol) in 1,2-dichloroethane (3 mL) at room temperature was added $NaHCO_3$ (81.6 mg, 0.97 mmol) followed by 1-chloroethyl chloroformate (0.11 mL, 1.02 mmol). The resulting mixture was warmed to reflux and stirred for 2 h before it was cooled to room temperature and quenched with $NaHCO_3$ (2 mL, sat. aq.) and water (2 mL). The layers were separated and the aqueous layer was extracted with CH_2Cl_2 (3 × 8 mL), the combined organic layer was dried (Na_2SO_4) and concentrated under reduced pressure to afford a crude residue, which was dissolved in MeOH (3 mL) and warmed to reflux and heated for 1.5 h before it was cooled to room temperature and

concentrated under reduced pressure to afford crude amine **17a** (14.3 mg, 93%) as an amorphous yellow solid. **17a**: R_f = 0.10 (silica gel, EtOAc:MeOH 1:1).

(ii) To a stirred solution of crude amine **17a** (9.8 mg, 31 μmol) in CH_2Cl_2 (1.5 mL) at room temperature was added TsCl (11.8 mg, 62 μmol), DMAP (1.5 mg, 12 μmol) and Et_3N (0.10 mL, 0.72 mmol). The resulting mixture was stirred for 1.5 h before it was diluted with water (2 mL). The layers were separated and the aqueous layer was extracted with CH_2Cl_2 (3 × 5 mL), the combined organic layer was dried (Na_2SO_4) and concentrated under reduced pressure. Flash column chromatography (silica gel, hexanes:EtOAc 4:1) afforded ketone **18** (10.0 mg, 69%) as an amorphous yellow solid. **18**: R_f = 0.68 (silica gel, hexanes:EtOAc 1:1); IR (film) ν_{max} 2960, 1700, 1610, 1490, 1160 cm^{-1}; ^1H NMR (400 MHz, $CDCl_3$): δ 7.50 (d, J = 6.5 Hz, 2H), 7.23 (d, J = 6.5 Hz, 2H), 6.85 (d, J = 8.7 Hz, 1H), 6.80 (d, J = 8.7 Hz, 1H), 5.93 (br s, 1H), 3.90 (s, 3H), 3.83 (s, 3H), 3.47 (d, J = 12.5 Hz, 1H), 3.40 (br s, 2H), 2.91 (dt, J = 12.8, 3.4 Hz, 1H), 2.56 (s, 3H), 2.47 (d, J = 12.4 Hz, 1H), 2.40 (s, 3H), 2.74–2.27 (m, 6H), 1.79 ppm (dt, J = 12.3, 3.8 Hz, 1H); ^{13}C NMR (101 MHz, $CDCl_3$): δ 210.5, 151.4, 147.7, 143.1, 135.1, 134.8, 130.9, 129.5, 127.2, 126.6, 123.3, 122.2, 112.2, 60.4, 55.9, 53.1, 46.9, 44.0, 42.0, 34.7, 33.5, 31.1, 29.9, 21.5 ppm; HRMS calcd. For $C_{26}H_{31}NO_5SNa^+$ $[M + Na]^+$ 492.1815, found 492.1818.

Dioxolane 18a

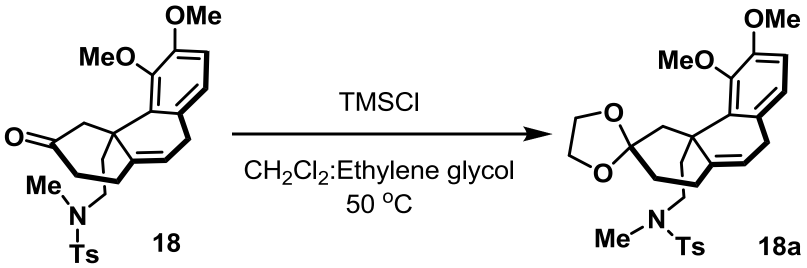

To a stirred solution of ketone **18** (18.0 mg, 38 μmol) in CH_2Cl_2/ethylene glycol (1:1, 3.0 mL) at room temperature was added TMSCl (10 μL, 79 μmol). The resulting mixture was warmed to 50 °C and stirred for 5 h before it was cooled to 0 °C and quenched with $NaHCO_3$ (3 mL, sat. aq.). The layers were separated and the aqueous layer was extracted with CH_2Cl_2 (3 × 3 mL), the combined organic layer was washed with water (5 mL), brine (5 mL), dried

(Na_2SO_4) and concentrated under reduced pressure. Flash column chromatography (silica gel, hexanes:EtOAc 3:1) afforded dioxolane **18a** (18.2 mg, 92%) as an amorphous yellow solid. **18a**: R_f = 0.45 (silica gel, hexanes:EtOAc 2:1); IR (film) ν_{max} 2900, 1510, 1460, 1160, 1080 cm^{-1}; ^1H NMR (400 MHz, $CDCl_3$): δ 7.50 (d, *J* = 7.2 Hz, 2H), 7.22 (d, *J* = 7.2 Hz, 2H), 6.80 (d, *J* = 8.6 Hz, 1H), 6.74 (d, *J* = 8.6 Hz, 1H), 5.71 (br s, 1H), 4.09–4.01 (m, 1H), 4.01–3.95 (m, 1H), 3.88 (s, 3H), 3.93–3.84 (m, 2H), 3.82 (s, 3H), 3.32 (d, *J* = 22.8 Hz, 1H), 3.23 (d, *J* = 22.0 Hz, 1H), 2.99 (dd, *J* = 14.4, 1.7 Hz, 1H), 2.81–2.69 (m, 1H), 2.59 (s, 3H), 2.64–2.49 (m, 2H), 2.38 (s, 3H), 2.44–2.28 (m, 2H), 2.17 (td, *J* = 13.7, 3.9 Hz, 1H), 1.84 (d, *J* = 12.2 Hz, 1H), 1.64 (d, *J* = 14.2 Hz, 1H), 1.57 ppm (dt, *J* = 13.7, 4.9 Hz, 1H); ^{13}C NMR (101 MHz, $CDCl_3$): δ 151.3, 147.8, 142.7, 137.3, 134.9, 133.1, 129.3, 127.2, 126.7, 123.1, 119.8, 111.6, 109.0, 64.5, 63.6, 60.2, 55.8, 47.6, 45.0, 42.3, 36.2, 34.7, 32.7, 30.1, 29.9, 21.4 ppm; HRMS calcd. For $C_{28}H_{35}NO_6SNa^+$ $[M + Na]^+$ 536.2077, found 536.2079.

Phenolic Ketone 19

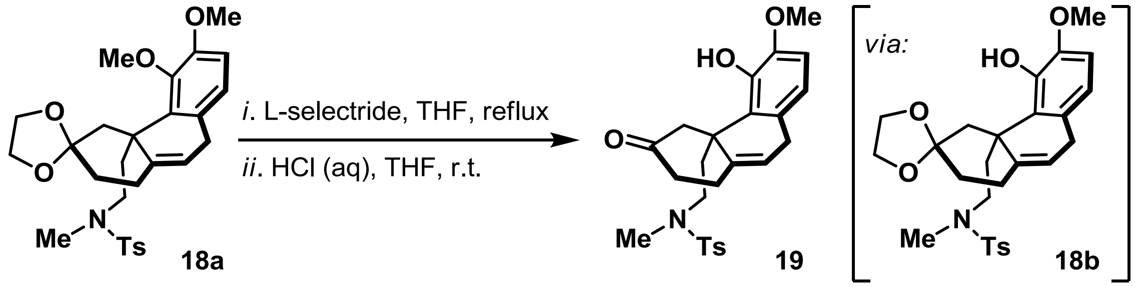

(i) To a stirred solution of dioxolane **18a** (22 mg, 43 μmol) in THF (1.5 mL) at room temperature was added L-selectride (1.0 M in THF, 0.21 mL, 0.21 mmol). The resulting mixture was warmed to reflux and stirred for 24 h before it was cooled to room temperature and quenched with sodium potassium tartrate (3 mL, sat. aq.) and diluted with EtOAc (5 mL), and stirred vigorously for 1 h. The layers were separated and the aqueous layer was extracted with ethyl acetate (3 × 8 mL), the combined organic layer was dried (Na_2SO_4) and concentrated under reduced pressure to afford crude phenol **18b** as a clear amorphous solid, which was used directly in the subsequent step without further purification. **18b**: R_f = 0.39 (silica gel, hexanes:EtOAc 2:1); ^1H NMR (400 MHz, $CDCl_3$): δ 7.51 (d, *J* = 8.3 Hz, 2H), 7.23 (d, *J* = 8.3 Hz, 2H), 6.73 (d, *J* = 8.3 Hz, 1H), 6.57 (d, *J* = 8.3 Hz, 1H), 6.01 (s, 1H), 5.71 (br s,

1H), 4.10–4.02 (m, 1H), 4.02–3.93 (m, 1H), 3.86 (s, 3H), 3.93–3.82 (m, 2H), 3.33 (br d, *J* = 22.6 Hz, 1H), 3.25 (br d, *J* = 22.0 Hz, 1H), 3.15 (dd, *J* = 14.4, 2.4 Hz, 1H), 2.92–2.82 (m, 1H), 2.58 (s, 3H), 2.59–2.49 (m, 3H), 2.40 (s, 3H), 2.36–2.26 (m, 1H), 2.18 (br d, *J* = 13.3 Hz, 1H), 1.87 (br d, *J* = 12.3 Hz, 1H), 1.61 (d, *J* = 13.8 Hz, 1H), 1.62–1.56 ppm (m, 1H).

18b: ^{1}H NMR (400 MHz, $CDCl_3$, crude)

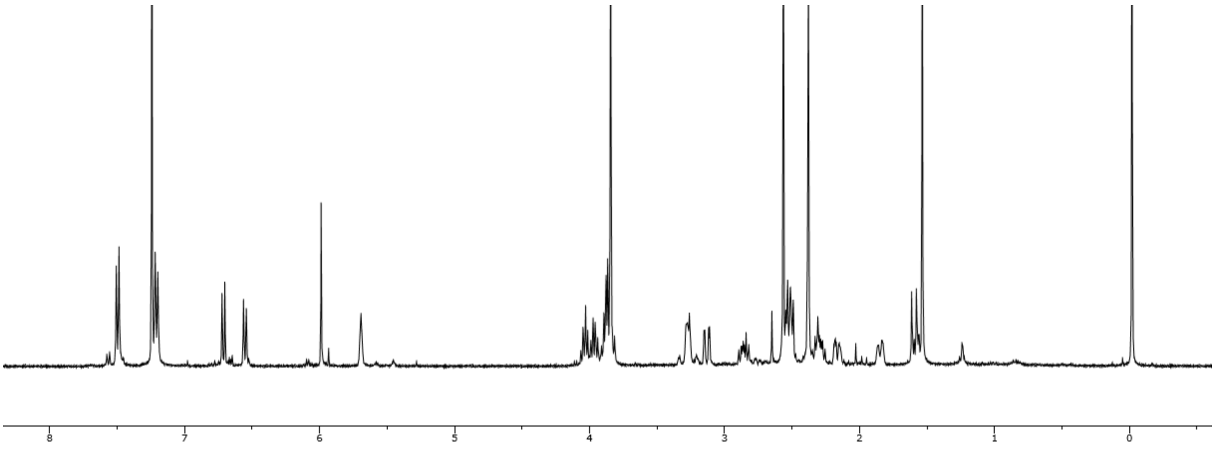

(ii) To a stirred solution of crude phenolic dioxolane **18b** (obtained above) in MeOH (1.5 mL) at room temperature was added HCl (4.0 N aq., 0.1 mL, 0.40 mmol). The resulting mixture was warmed to 45 °C stirred for 3 h before it was cooled to 0 °C quenched with $NaHCO_3$ (5 mL). The layers were separated and the aqueous layer was extracted with CH_2Cl_2 (3 × 8 mL), the combined organic layer was dried (Na_2SO_4) and concentrated under reduced pressure. Flash column chromatography (silica gel, hexanes:EtOAc 4:1) afforded phenolic ketone **19** (15 mg, 77% over two steps) as a clear amorphous solid. **19**: R_f = 0.30 (silica gel, hexanes:EtOAc 2:1); IR (film) ν_{max} 3440, 2950, 1690, 1490, 1160 cm^{-1}; ^{1}H NMR (400 MHz, $CDCl_3$): δ 7.51 (d, *J* = 8.6 Hz, 2H), 7.23 (d, *J* = 8.6 Hz, 2H), 6.77 (d, *J* = 7.6 Hz, 1H), 6.63 (d, *J* = 7.6 Hz, 1H), 6.05 (s, 1H), 5.92 (br s, 1H), 3.88 (s, 3H), 3.65 (d, *J* = 13.6 Hz, 1H), 3.40 (br s, 2H), 2.98 (dt, *J* = 12.3, 4.8 Hz, 1H), 2.82 (dt, *J* = 12.3, 4.1 Hz, 1H), 2.67 (br t, *J* = 13.6 Hz 1H), 2.57 (s, 3H), 2.60–2.51 (m, 1H), 2.40 (s, 3H), 2.49–2.37 (m, 3H), 2.32 (dt, *J* = 12.3, 4.1 Hz, 1H), 1.70 ppm (dt, *J* = 12.3, 5.0 Hz, 1H); ^{13}C NMR (101 MHz, $CDCl_3$): δ 210.6, 144.8, 143.1, 143.0, 135.3, 134.8, 129.5, 127.2, 126.9, 123.8, 122.1, 119.2, 109.5, 56.2, 51.9, 46.9, 43.5, 42.2, 34.5, 31.5, 31.0, 29.8, 21.5 ppm; HRMS calcd. For $C_{25}H_{29}NO_5SNa^+$ [M + Na]$^+$ 478.1659, found 478.1660.

Tetracyclic Ketone 20 and 20a

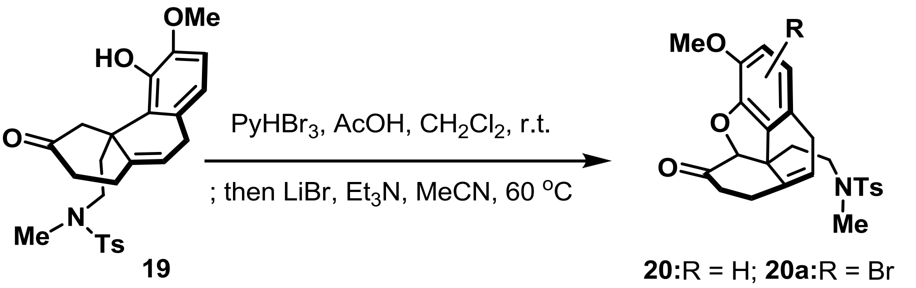

(i) To a stirred solution of phenolic ketone **19** (47 mg, 0.10 mmol) in CH_2Cl_2 (2.0 mL) at room temperature was added a solution of pyridinium tribromide (freshly recrystallized, 66 mg, 0.20 mmol) in acetic acid (5.0 mL) dropwise. The resulting mixture was stirred for 30 min before it was diluted with toluene and concentrated under reduced pressure. The resulting residue was redissolved in CH_2Cl_2 (5 mL) and water (5 mL), extracted with CH_2Cl_2 (3 × 5 mL), the combined organic layer was washed with water (10 mL), brine (10 mL), dried (Na_2SO_4) and concentrated under reduced pressure. To a stirred solution of the crude reaction mixture (obtained above) in MeCN (5.0 mL) at room temperature was added LiBr (44.8 mg, 0.52 mmol) and Et_3N (0.14 mL, 1.03 mmol). The resulting mixture was warmed to 60 °C and stirred for 20 min before it was cooled to room temperature and quenched with NH_4Cl (5 mL, sat. aq.) and water (5 mL). The layers were separated and the aqueous layer was extracted with EtOAc (3 × 10 mL), the combined organic layer was washed with water (15 mL), brine (15 mL), dried (Na_2SO_4) and concentrated under reduced pressure. Flash column chromatography (silica gel, hexanes:EtOAc 7:1) afforded an inseparable mixture of tetracycles **20** and **20a** (25 mg, 53% over two steps) as an amorphous yellow solid. **20+20a**: R_f = 0.28 (silica gel, hexanes:EtOAc 1:1); ^{1}H NMR (400 MHz, $CDCl_3$): δ 7.59 (d, J = 7.7 Hz, 2H), 7.32–7.27 (m, 2H), 6.88 (s, 0.3H), 6.68 (s, 1.3H), 5.91–5.85 (m, 1H), 5.11 (s, 0.4H), 5.06 (s, 0.6H), 3.89 (s, 3H), 3.33–3.18 (m, 1H), 3.12 (dd, J = 5.6 Hz, 1H), 3.13–2.96 (m, 3H), 2.71–2.57 (m, 2H), 2.65 (s, 3H), 2.42 (s, 3H), 2.33–2.14 (m, 2H), 2.18 ppm (td, J = 12.0, 5.5 Hz, 1H); ^{13}C NMR (101 MHz, $CDCl_3$): δ 205.9, 205.4, 144.6, 144.1, 143.9, 143.6, 143.5, 143.1, 137.9, 137.6, 134.2, 134.1, 131.6, 130.4, 129.8, 129.7, 127.3, 126.8, 126.3, 124.2, 123.7, 120.5, 116.6, 113.5, 113.2, 90.4, 90.3, 56.9, 56.8, 50.2, 49.7, 46.2, 46.1, 37.4, 37.4, 36.5, 36.4, 35.3, 35.2, 29.9, 29.2, 27.8, 27.6, 21.5 ppm; **20**: HRMS calcd. For $C_{25}H_{27}$

NO_5SNa^+ $[M + Na]^+$ 476.1502, found 476.1505; **20a**: HRMS calcd. For $C_{25}H_{26}BrNO_5SNa^+$ $[M + Na]^+$ 554.0607, found 554.0604.

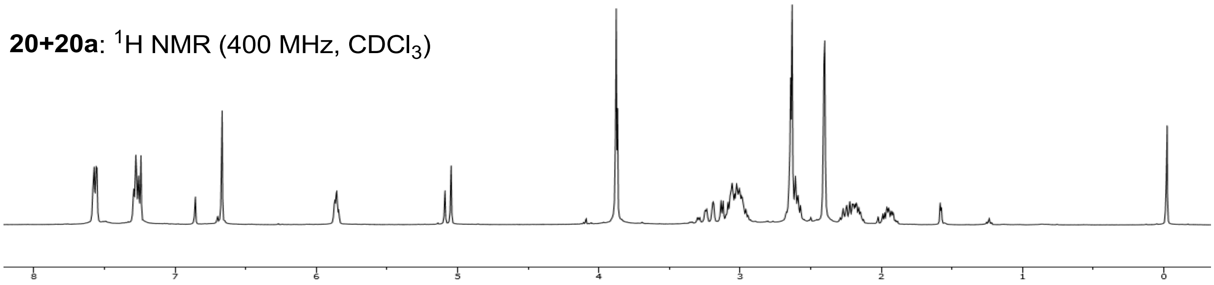

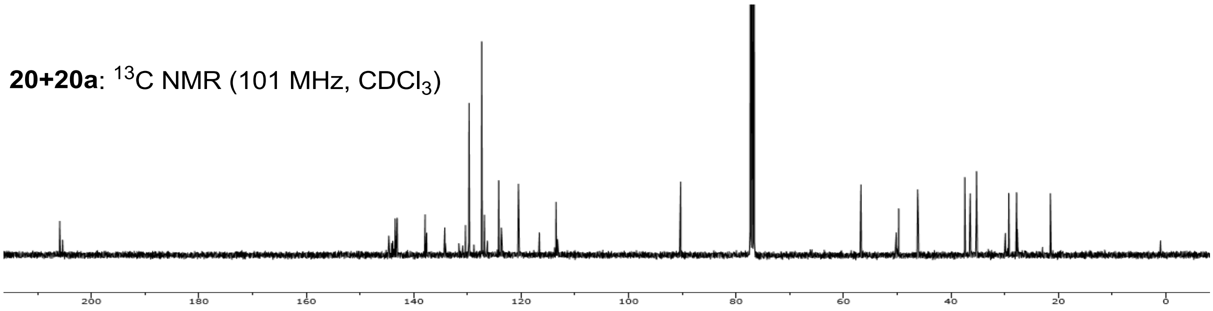

Pentacyclic Alkene 21

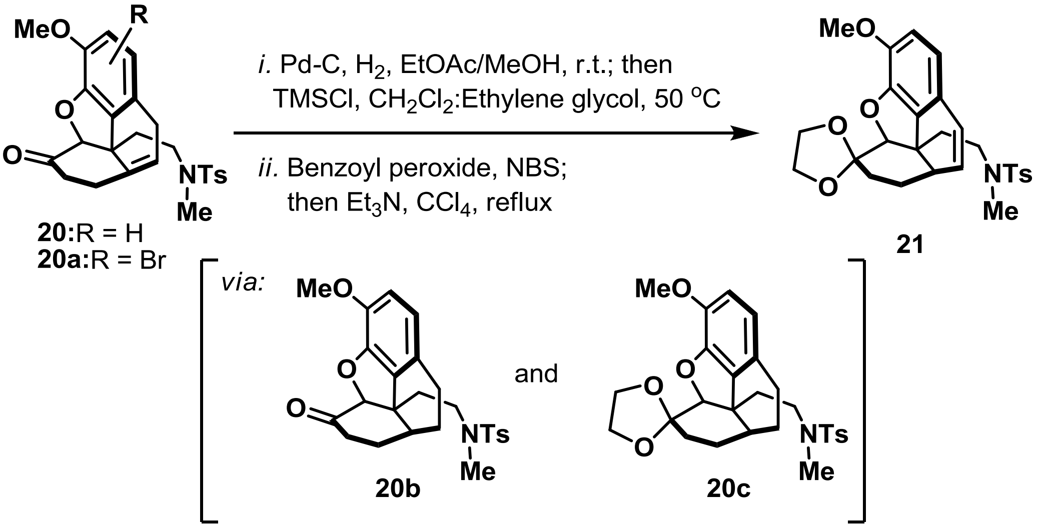

(i) To a stirred solution of tetracyclic ketone **20+20a** (14.0 mg, 31 μmol) in EtOAc/MeOH (1:3, 3.0 mL) at room temperature was added Pd/C (10% wt/wt, 6.6 mg, 6 μmol). The resulting mixture was evacuated and filled with hydrogen (3 ×) and stirred under an atmosphere of H_2 (balloon) for 1 h. The resulting mixture was filtered through Celite® and eluted with EtOAc (3 × 8 mL), and concentrated under reduced pressure. Flash column chromatography (silica gel, hexanes:EtOAc 3:1) afforded tetracyclic ketone **20b** (12.7 mg,

90%) as a clear amorphous solid. To a stirred solution of tetracyclic ketone **20b** (7.8 mg, 17 μmol) in CH_2Cl_2/ethylene glycol (1:1, 1.0 mL) at room temperature was added TMSCl (50 μL, 0.39 mmol). The resulting mixture was warmed to 50 °C and stirred for 5 h before it was cooled to 0 °C and quenched with $NaHCO_3$ (1 mL, sat. aq.). The layers were separated and the aqueous layer was extracted with CH_2Cl_2 (3 × 3 mL), the combined organic layer was washed with water (5 mL), brine (5 mL), dried (Na_2SO_4) and concentrated under reduced pressure. Flash column chromatography (silica gel, hexanes:EtOAc 3:1) afforded dioxolane **20c** (6.9 mg, 80%) as a clear amorphous solid.

20b: R_f = 0.30 (silica gel, hexanes:EtOAc 2:1); IR (film) ν_{max} 2820, 1720, 1600, 1520, 1350 cm^{-1}; ^1H NMR (400 MHz, $CDCl_3$): δ 7.66 (d, *J* = 7.8 Hz, 2H), 7.33 (d, *J* = 7.8 Hz, 2H), 6.70 (d, *J* = 7.8 Hz, 1H), 6.60 (d, *J* = 7.8 Hz, 1H), 5.08 (s, 1H), 3.89 (s, 3H), 3.34–3.23 (m, 1H), 3.15–3.06 (m, 1H), 2.82–2.66 (m, 2H), 2.72 (s, 3H), 2.59–2.46 (m, 2H), 2.43 (s, 3H), 2.33 (d, *J* = 12.7 Hz, 1H), 2.20–2.07 (m, 1H), 2.05–1.96 (m, 1H), 1.96–1.88 (m, 2H), 1.88–1.77 (m, 1H), 1.35 ppm (q, *J* = 13.3 Hz, 1H); ^{13}C NMR (101 MHz, $CDCl_3$): ^{13}C NMR (101 MHz, $CDCl_3$): δ 207.5, 146.0, 143.6, 142.6, 133.8, 129.8, 128.7, 127.4, 124.9, 121.3, 114.5, 90.4, 56.7, 50.3, 46.7, 39.4, 34.9, 34.2, 33.7, 27.0, 23.4, 21.5, 20.8 ppm; HRMS calcd. For $C_{25}H_{29}NO_5SNa^+$ $[M + Na]^+$ 478.1659, found 478.1661.

20c: R_f = 0.45 (silica gel, hexanes:EtOAc 2:1); IR (film) ν_{max} 2960, 2360, 1510, 1340, 1270 $cm^{-1;}$ ^1H NMR (400 MHz, $CDCl_3$) δ 7.60 (d, *J* = 8.8 Hz, 2H), 7.27 (d, *J* = 8.8 Hz, 2H), 6.72 (d, *J* = 7.5 Hz, 1H), 6.60 (d, *J* = 7.5 Hz, 1H), 4.57 (s, 1H), 4.19–4.12 (m, 1H), 3.97–3.89 (m, 1H), 3.87–3.78 (m, 1H), 3.84 (s, 3H), 3.77–3.70 (m, 1H), 3.37–3.24 (m, 1H), 2.89–2.77 (m, 1H), 2.72–2.61 (m, 1H), 2.66 (s, 3H), 2.59–2.46 (m, 1H), 2.40 (s, 3H), 2.17–2.02 (m, 2H), 1.93–1.81 (m, 1H), 1.81–1.67 (m, 1H), 1.65–1.42 (m, 4H), 1.35–1.21 ppm (m, 1H); ^{13}C NMR (101 MHz, $CDCl_3$) δ 147.0, 143.2, 141.9, 134.5, 130.0, 129.7, 127.4, 125.3, 120.3, 113.7, 108.3, 93.3, 66.4, 64.8, 56.6, 46.5, 46.2, 36.8, 34.9, 34.3, 33.0, 23.8, 23.5, 21.5, 21.0 ppm; HRMS calcd. For $C_{27}H_{33}NO_6SNa^+$ $[M + Na]^+$ 522.1921, found 522.1922.

(ii) To a stirred solution of pentacyclic dioxolane **20c** (23.0 mg, 46 μmol) in carbon tetrachloride (freshly distilled, 3.6 mL) at room temperature was added benzoyl peroxide (freshly recrystallized, 5.6 mg, 23 μmol) and *N*-bromosuccinimide (8.6 mg, 48 mmol). The

resulting mixture was warmed to reflux and stirred for 1 h before it was cooled to room temperature and added Et_3N (50 μL, 0.36 mmol). The resulting mixture was warmed to reflux and stirred for 15 min before it was cooled to room temperature. The resulting mixture was washed with $NaHCO_3$ (5 mL, sat. aq.), $Na_2S_2O_3$ (5 mL, sat. aq.), brine (5 mL), dried (Na_2SO_4) and concentrated under reduced pressure. Flash column chromatography (silica gel, hexanes:EtOAc 4:1) afforded an inseparable mixture of alkene **21** and starting material **20c** (**21**:**20c** ~ 3:1, 18 mg, 60% yield for **21** based on ^{1}H NMR calculation) as an amorphous solid. **21**+**20c**: R_f = 0.45 (silica gel, hexanes:EtOAc 2:1); ^{1}H NMR (400 MHz, CD_3CN, selected signals for **23**): δ 7.50 (d, J = 7.7 Hz, 2H), 7.36 (d, J = 7.7 Hz, 2H), 6.77 (d, J = 8.1 Hz, 1H), 6.66 (d, J = 8.1 Hz, 1H), 6.38 (d, J = 9.3 Hz, 1H), 5.81(dd, J = 12.7, 8.7 Hz, 1H), 4.65 (s, 1H), 4.14–4.02 (m, 1H), 3.90–3.72 (m, 3H), 3.86 (s, 3H), 3.03–2.92 (m, 1H), 2.88–2.77 (m, 1H), 2.64–2.57 (m, 1H), 2.59 (s, 3H), 2.42 (s, 3H), 1.80–1.62 (m, 3H), 1.56–1.38 (m, 2H), 1.08 ppm (qd, J = 12.7, 3.3 Hz, 1H).

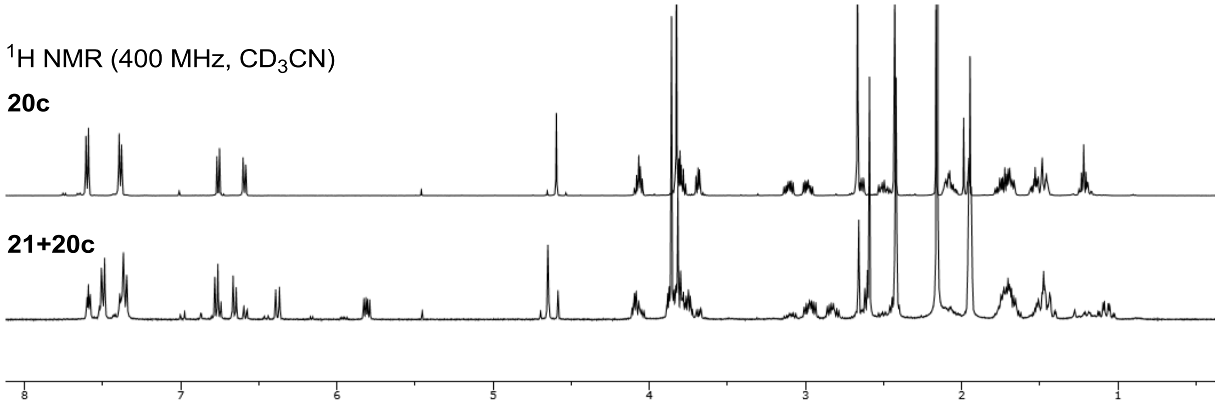

Dihydrocodeinone

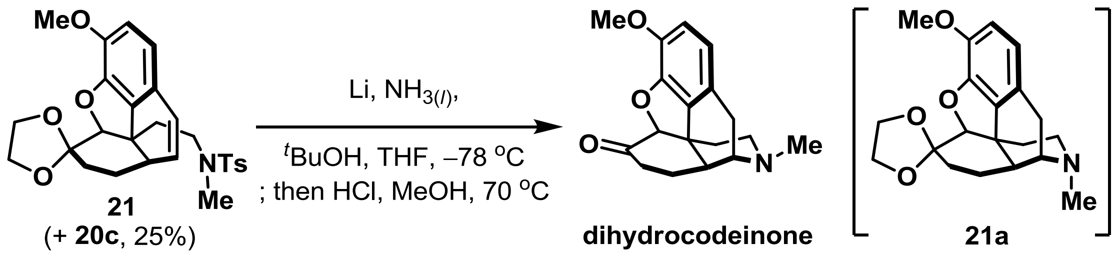

To a stirred solution liquid ammonia (10 mL), THF (1.0 mL) and ^{t}BuOH (0.1 mL) at –78 °C was added lithium metal (finely-cut, 30 mg) in small portions. The resulting solution was

stirred for 15 min before a solution of tosylamide **21** (ca. ~3:1 mixture with **20c**, 8.0 mg, 16 μmol) in THF (1.5 mL) was added *via* a cannula. The resulting mixture was stirred for 10 min before it was quenched with NH_4Cl (10 mL, sat. aq.) and MeOH (10 mL). The layers were separated and the aqueous layer was extracted with CH_2Cl_2 (3 × 15 mL), the combined organic layer was dried (Na_2SO_4) and concentrated under reduced pressure. Flash column chromatography (silica gel, CH_2Cl_2:MeOH:NH_4OH 94:2:1) afforded hexacyclic dioxolane **21a** (3.8 mg, 69%) as an amorphous yellow solid.

To a stirred solution of dioxolane **21a** (obtained above, 3.8 mg, 11 μmol) in MeOH (3.0 mL) at room temperature was added HCl (4.0 N aq., 0.2 mL, 0.80 mmol). The resulting mixture was warmed to 70 °C stirred for 6 h before it was cooled to 0 °C quenched with $NaHCO_3$ (5 mL). The layers were separated and the aqueous layer was extracted with CH_2Cl_2 (3 × 8 mL), the combined organic layer was dried (Na_2SO_4) and concentrated under reduced pressure. Flash column chromatography (silica gel, CH_2Cl_2:MeOH:NH_4OH 94:2:1) afforded dihydrocodeinone (2.5 mg, 75%) as a clear amorphous solid.

21a: R_f = 0.20 (silica gel, EtOAc:MeOH 1:1); IR (film) ν_{max} 2920, 2360, 1510, 1440, 1280 cm^{-1}; ^1H NMR (400 MHz, $CDCl_3$): δ 6.74 (d, J = 8.2 Hz, 1H), 6.62 (d, J = 8.2 Hz, 1H), 4.49 (s, 1H), 4.24–4.14 (m, 1H), 4.04 (q, J = 6.5 Hz, 1H), 3.87 (s, 3H), 3.96–3.83 (m, 1H), 3.79 (q, J = 6.2 Hz, 1H), 3.13–3.06 (br s, 1H), 3.00 (d, J = 18.3 Hz, 1H), 2.51 (dd, J = 12.2, 4.8 Hz, 1H), 2.43–2.29 (m, 1H), 2.41 (s, 3H), 2.20 (td, J = 12.1, 3.7 Hz, 2H), 1.86 (td, J = 12.2, 4.9 Hz, 1H), 1.74–1.61 (m, 2H), 1.60–1.46 (m, 2H), 1.15 ppm (qd, J = 12.9, 3.2 Hz, 1H); ^{13}C NMR (101 MHz, $CDCl_3$) δ 146.5, 142.1, 129.1, 126.4, 118.6, 113.4, 108.6, 94.4, 66.4, 64.9, 59.5, 56.5, 47.1, 43.6, 42.9, 42.6, 36.5, 33.4, 22.3, 20.1 ppm; HRMS calcd. For $C_{20}H_{26}NO_4^+$ $[M + H]^+$ 344.1856, found 344.1859.

Dihydrocodeinone: R_f = 0.17 (silica gel, EtOAc:MeOH 1:1); IR (film) ν_{max} 3410, 2960, 1725, 1510, 1430 cm^{-1}; ^1H NMR (400 MHz, $CDCl_3$): δ 6.68 (d, J = 8.2 Hz, 1H), 6.62 (d, J = 8.2 Hz, 1H), 4.64 (s, 1H), 3.89 (s, 3H), 3.16 (br s, 1H), 3.01 (d, J = 18.5 Hz, 1H), 2.61–2.50 (m, 2H), 2.47–2.38 (m, 2H), 2.41 (s, 3H), 2.31 (td, J = 14.2, 4.3 Hz, 1H), 2.17 (td, J = 11.8, 2.9 Hz, 1H), 2.05 (td, J = 12.2, 4.8 Hz, 1H), 1.89–1.78 (m, 2H), 1.24 ppm (qd, J = 13.1, 3.8 Hz, 1H); ^{13}C NMR (101 MHz, $CDCl_3$): δ 207.9, 145.4, 142.8, 127.2, 126.3, 119.8, 114.5, 91.4, 59.2,

56.7, 46.9, 46.8, 42.9, 42.7, 40.2, 35.6, 25.6, 20.0 ppm; HRMS calcd. For $C_{18}H_{22}NO_3^+$ [M + H]$^+$ 300.1594, found 300.1597.

Dihydrocodeine

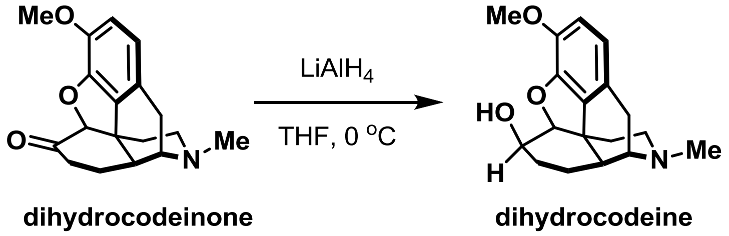

To a stirred solution of dihydrocodeinone (2.5 mg, 8.4 μmol) in THF (1.0 mL) at 0 °C was added $LiAlH_4$ (10 mg, 0.26 mmol). The resulting mixture was stirred for 45 min before it was quenched with sodium potassium tartrate (3 mL, sat. aq.) and diluted with EtOAc (3 mL), and stirred vigorously for 1 h. The layers were separated and the aqueous layer was extracted with EtOAc (4 × 5 mL), the combined organic layer was dried (Na_2SO_4) and concentrated under reduced pressure. Flash column chromatography (silica gel, CH_2Cl_2:MeOH:NH_4OH 94:2:1) afforded dihydrocodeine (1.9 mg, 74%) as a clear amorphous solid. **Dihydrocodeine**: R_f = 0.23 (silica gel, EtOAc:MeOH 1:3); IR (film) ν_{max} 3470, 2950, 1510, 1250, 1050 cm^{-1}; ^{1}H NMR (400 MHz, $CDCl_3$): δ 6.70 (d, J = 8.2 Hz, 1H), 6.61 (d, J = 8.2 Hz, 1H), 4.59 (d, J = 5.2 Hz, 1H), 4.02 (br s, 1H), 3.86 (s, 3H), 3.06 (br s, 1H), 2.98 (d, J = 18.5 Hz, 1H), 2.49 (dd, J = 12.0, 4.8 Hz, 1H), 2.39–2.30 (m, 1H), 2.38 (s, 3H), 2.27–2.13 (m, 2H), 1.83 (td, J = 12.0, 4.8 Hz, 1H), 1.68 (d, J = 12.5 Hz, 1H), 1.59–1.51 (m, 1H), 1.49–1.37 (m, 2H), 1.17–1.02 ppm (m, 1H); ^{13}C NMR (101 MHz, $CDCl_3$): 146.2, 141.7, 130.0, 126.5, 119.2, 113.3, 90.3, 67.1, 60.0, 56.4, 47.0, 42.8, 41.9, 40.4, 37.0, 27.2, 20.2, 18.9 ppm; HRMS calcd. For $C_{18}H_{24}NO_3^+$ [M + H]$^+$ 302.1754, found 302.1751.

Comparison Table of Notable Contributions in the Synthesis of Morphinans:

(Longest linear sequence and approximated overall yield were calculated based on the best of our knowledge with reference to the original literature)

**Author (Year)[ref]**	**Final Target**	**Racemic /Asymmetric**	**Longest linear sequence (steps)**	**Overall yield**
This work	dihydrocodeinone	Racemic /Asymmetric	25 steps	0.1%
Fukuyama/Inoue (2017)[w]	Morphine	Asymmetric	18 steps	0.3%
Smith (2016)[v]	Morphine	Racemic	14 steps	6.6%
Zhang (2015)[u]	Codeine	Racemic	19 steps	3.0%
Opatz (2014)[t]	Dihydrocodeine	Asymmetric	12 steps	31.0%
Hudlicky (2014)[s]	Hydromorphone	Asymmetric	12 steps	2.3%
Chida (2013)[r]	Morphine (Formal)	Asymmetric	28 steps	2.0%
Metz (2011)[q]	Codeine	Racemic	23 steps	3.5%
Fukuyama (2010)[p]	Morphine	Asymmetric	17 steps	5.0%
Stork (2009)[o]	Codeine	Racemic	23 steps	2.1%
Magnus (2009)[n]	Codeine	Racemic	13 steps	20.0%
Guillou/Iorga (2008)[m]	Codeine	Racemic	14 steps	0.6%
Trost (2005)[l]	Morphine	Asymmetric	16 steps	3.2%
Taber (2002)[k]	Morphine	Asymmetric	23 steps	0.8%
White (1997)[j]	Morphine	Asymmetric	28 steps	3.0%
Mulzer (1996)[i]	Dihydrocodeinone	Asymmetric	11 steps	11.5%
Overman (1993)[h]	Dihydrocodeinone	Asymmetric	15 steps	4.7%
Parker (1992)[g]	Dihydro-isocodeine	Racemic	11 steps	11.4%
Fuchs (1987)[f]	Morphine	Racemic	27 steps	1.1%
Rapoport (1983)[e]	Codeine	Racemic	26 steps	1.2%
Rice (1980)[d]	Dihydrocodeinone	Asymmetric	10 steps	29.0%
Grewe (1967)[c]	Dihydro-thebainone	Racemic	9 steps	0.8%
Ginsberg (1954)[b]	Dihydro-thebainone	Racemic	21 steps	8.9%
Gates (1952)[a]	Morphine	Asymmetric	23 steps	0.01%

References: (a) Gates, M.; Tschudi, G. *J. Am. Chem. Soc.* **1952**, *74*, 1109. (b) Elad, D.; Ginsburg, D. *J. Am. Chem. Soc.* **1954**, *76*, 312. (c) Grewe, R.; Friedrichsen, W. *Chem. Ber.* **1967**, *100*, 1550. (d) Rice, K. C. *J. Org. Chem.* **1980**, *45*, 3135. (e) Moos, W. H.; Gless, R. D.; Rapoport, H. *J. Org. Chem.* **1983**, *48*, 227. (f) Toth, J. E.; Fuchs, P. L. *J. Org. Chem.* **1987**, *52*, 473. (g) Parker, K. A.; Fokas, D. *J. Am. Chem. Soc.* **1992**, *114*, 9688. (h) Hong, C. Y.; Kado,

N.; Overman, L. E. *J. Am. Chem. Soc.* **1993**, *115*, 11028. (i) Mulzer, J.; Duerner, G.; Trauner, D. *Angew. Chem. Int. Ed.* **1996**, *35*, 2830. (j) White, J. D.; Hrnciar, P.; Stappenbeck, F. *J. Org. Chem.* **1997**, *62*, 5250. (k) Taber, D. F.; Neubert, T. D.; Rheingold, A. L. *J. Am. Chem. Soc.* **2002**, *124*, 12416. (l) Trost, B. M.; Tang, W.; Toste, F. D. *J. Am. Chem. Soc.* **2005**, *127*, 14785. (m) Verin, M.; Barre, E.; Iorga, B.; Guillou, C. *Chem. Eur. J.* **2008**, *14*, 6606. (n) Magnus, P.; Sane, N.; Fauber, B. P.; Lynch, V. *J. Am. Chem. Soc.* **2009**, *131*, 16045. (o) Stork, G.; Yamashita, A.; Adams, J.; Schulte, G. R.; Chesworth, R.; Miyazaki, Y.; Farmer, J. J. *J. Am. Chem. Soc.* **2009**, *131*, 11402. (p) Koizumi, H.; Yokoshima, S.; Fukuyama, T. *Chem. Asian J.* **2010**, *5*, 2192. (q) Erhard, T.; Ehrlich, G.; Metz, P. *Angew. Chem. Int. Ed.* **2011**, *50*, 3892. (r) Ichiki, M.; Tanimoto, H.; Miwa, S.; Saito, R.; Sato, T.; Chida, N. *Chem. Eur. J.* **2013**, *19*, 264. (s) Varghese, V.; Hudlicky, T. *Angew. Chem. Int. Ed.* **2014**, *53*, 4355. (t) Geffe, M.; Opatz, T. *Org. Lett.* **2014**, *16*, 5282. (u) Li, Q.; Zhang, H. *Chem. Eur. J.* **2015**, *21*, 16379. (v) Chu, S.; Munster, N.; Balan, T.; Smith, M. D. *Angew. Chem. Int. Ed.* **2016**, *55*, 14306. (w) Umihara, H.; Yokoshima, S.; Inoue, M.; Fukuyama, T. *Chem. Eur. J.* **2017**, *23*, 6993.

Alkene 2j

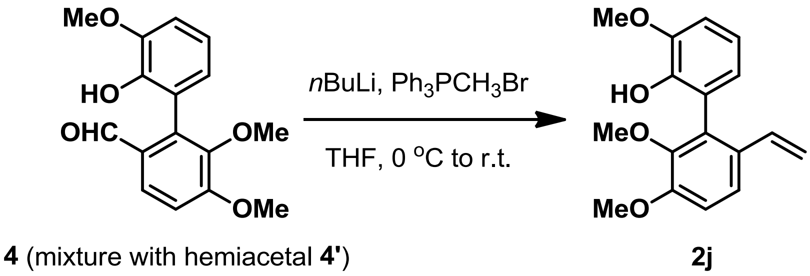

To a stirred solution of methyltriphenylphosphonium bromide (dried over P_2O_5, 10.0 g, 30.0 mmol) in THF (150 mL) at 0 °C was added *n*BuLi (2.5 M in hexanes, 8.90 mL, 22.3 mmol). The resulting mixture was stirred for 15 min before a solution of hemiacetal-hydroxy aldehyde mixture (**4**+**4′**, 1.60 g, 5.55 mmol) in THF (30 mL) was added. The resulting mixture was stirred for 1 h before it was warmed to room temperature and quenched with NH_4Cl (40 mL, sat. aq.) and water (40 mL). The layers were separated and the aqueous layer was extracted with Et_2O (3 × 50 mL), the combined organic layer was washed with water (80 mL), brine (80 mL), dried (Na_2SO_4) and concentrated under reduced pressure. Flash column chromatography (silica gel, hexanes:EtOAc 4:1) afforded alkene **2j** (1.13 g, 71%) as an amorphous white solid. **2j**: R_f = 0.50 (silica gel, hexanes:EtOAc 1:1); IR (film) ν_{max} 3533, 3086, 3009, 1621, 1594, 1471, 1357 cm^{-1}; ^{1}H NMR (400 MHz, $CDCl_3$): δ 7.43 (d, *J* = 8.6 Hz, 1H), 6.97 (d, *J* = 8.7 Hz, 1H), 6.93–6.89 (m, 2H), 6.76–6.72 (m, 1H), 6.37 (dd, *J* = 17.5, 11.0 Hz, 1H), 5.68 (s, 1H), 5.56 (d, *J* = 17.5 Hz, 1H), 5.01 (d, *J* = 10.9 Hz, 1H), 3.93 (s, 3H), 3.91 (s, 3H), 3.62 ppm (s, 3H); ^{13}C NMR (101 MHz, $CDCl_3$): δ 152.3, 146.7, 146.6, 143.1, 134.6, 131.0, 130.3, 123.7, 122.7, 120.6, 119.3, 112.7, 112.0, 109.9, 60.6, 60.6, 55.8 ppm; HRMS calcd. For $C_{17}H_{18}O_4Na^+$ $[M + Na]^+$ 309.1097, found 309.1095.

Alcohol 2k

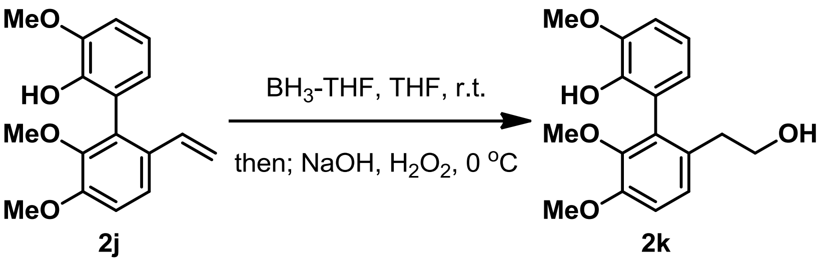

To a stirred solution of alkene **2j** (1.10 g, 3.84 mmol) in THF (100 mL) at −78 °C was added borane tetrahydrofuran complex (1.0 M in THF, 38.4 mL, 38.4 mmol). The resulting mixture was warmed to room temperature and stirred for 4 h before it was cooled to 0 °C and treated with NaOH (2.0 N aq., 5.16 mL, 10.3 mmol) and H_2O_2 (34.5% aq., 5.16 mL, 52.3 mmol). The resulting mixture was stirred for 4 h before it was quenched with NH_4Cl (80 mL, sat. aq.) and water (20 mL). The layers were separated and the aqueous layer was extracted with ethyl acetate (3 × 100 mL), the combined organic layer was washed with water (100 mL), brine (100 mL), dried (Na_2SO_4) and concentrated under reduced pressure. Flash column chromatography (silica gel, hexanes:EtOAc 3:1) afforded alcohol **2k** (1.02 g, 87%) as an amorphous white solid. **2k**: R_f = 0.19 (silica gel, hexanes:EtOAc 1:1); IR (film) ν_{max} 3620, 3233, 3004, 1620, 1459, 946 cm^{-1}; ^1H NMR (400 MHz, C_6D_6): δ 6.94 (d, J = 8.4 Hz, 1H), 6.83 (d, J = 7.7 Hz, 1H), 6.76 (t, J = 8.0 Hz, 1H), 6.63 (d, J = 8.5 Hz, 1H), 6.45 (d, J = 7.9 Hz, 1H), 3.68 (s, 3H), 3.65–3.59 (m, 2H), 3.37 (s, 3H), 3.15 (s, 3H), 2.78–2.61 ppm (m, 2H); ^{13}C NMR (101 MHz, $CDCl_3$): δ 151.3, 147.7, 146.7, 142.8, 132.3, 130.1, 124.9, 123.4, 123.3, 119.5, 112.0, 109.8, 63.1, 60.5, 55.8, 55.8, 35.8 ppm; HRMS calcd. For $C_{17}H_{20}O_5Na^+$ [M + Na]$^+$ 327.1203, found 327.1203.

Allylic Alcohol 6g

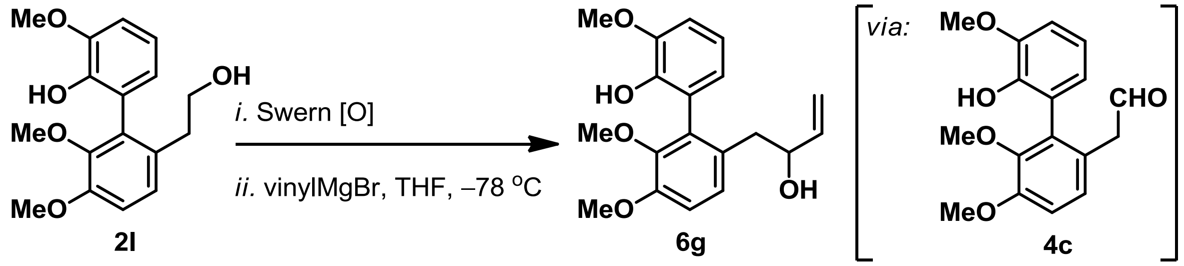

(i) To a stirred solution of oxalyl chloride (0.43 mL, 5.01 mmol) in CH_2Cl_2 (13.0 mL) at −78 °C was added DMSO (0.70 mL, 9.86 mmol) dropwise. The resulting mixture was stirred for 15 min before a solution of alcohol **2l** (150 mg, 0.49 mmol) in CH_2Cl_2 (13.0 mL) was added. The resulting mixture was stirred for 1 h before Et_3N (2.10 mL, 14.8 mmol) was added, and the resulting mixture was stirred for 1 h before it was warmed to room temperature and quenched with NH_4Cl (80 mL, sat. aq.) and water (20 mL). The layers were separated and the

aqueous layer was extracted with Et_2O (3 × 100 mL), the combined organic layer was washed with water (100 mL), brine (100 mL), dried (Na_2SO_4) and concentrated under reduced pressure to afford crude aldehyde **4c** (112 mg, 75%) as an amorphous yellow solid, which was used directly in the following step without further purification.

(ii) To a stirred solution of crude aldehyde (**4c**, obtained via above procedure, 380 mg, 1.26 mmol) in THF (50.0 mL) at −78 °C was added vinyl magnesium bromide (1.0 M in THF, 7.54 mL, 7.54 mmol) dropwise. The resulting mixture was warmed to room temperature and stirred for 16 h before it was quenched with water (50 mL). The layers were separated and the aqueous layer was extracted with Et_2O (3 × 150 mL), the combined organic layer was washed with water (100 mL), brine (100 mL), dried (Na_2SO_4) and concentrated under reduced pressure. Flash column chromatography (silica gel, hexanes:EtOAc 4:1) afforded allylic alcohol **6g** (370 mg, 89%) as an amorphous yellow solid. **6g**: R_f = 0.30 (silica gel, hexanes:EtOAc 1:1); IR (film) ν_{max} 3690, 3528, 3054, 1602, 1264, 700 cm^{-1}; ^1H NMR (400 MHz, $CDCl_3$, mixture of atropisomers): δ 7.06 (t, J = 7.9 Hz, 1H), 6.97–6.84 (m, 3H), 6.75–6.73 (m, 1H), 5.84 (s, 1H), 5.78–5.67 (m, 1H), 5.12–4.94 (m, 2H), 4.17–4.03 (m, 1H), 3.92 (s, 3H), 3.88 (s, 3H), 3.60 (s, 1.5H), 3.58 (s, 1.5H), 2.74–2.68 (m, 1H), 2.59–2.49 (m, 1H), 1.74 ppm (br s, 1H); ^{13}C NMR (101 MHz, $CDCl_3$, mixture of atropisomers): δ 151.4, 151.4, 147.1, 147.0, 146.8, 146.7, 143.0, 142.5, 140.5, 132.4, 132.2, 129.7, 129.5, 125.9, 125.2, 123.5, 123.4, 123.4, 123.2, 119.6, 119.5, 114.3, 114.0, 112.0, 111.7, 109.9, 109.8, 73.4, 72.3, 60.5, 60.4, 55.9, 55.8, 55.8, 55.7, 41.0, 40.6 ppm; HRMS calcd. For $C_{19}H_{22}O_5Na^+$ $[M + Na]^+$ 353.1359, found 353.1362.

Alcohol 2n

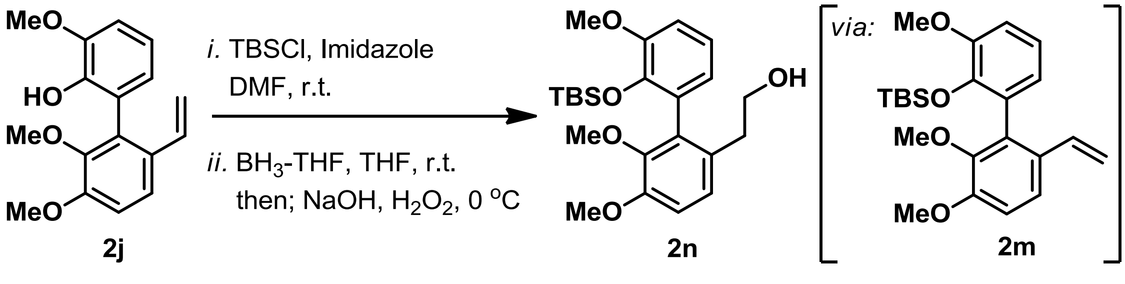

(i) To a stirred solution of phenol **2j** (0.22 g, 0.77 mmol) in DMF (12.0 mL) at room temperature was added TBSCl (0.46 g, 3.05 mmol) and imidazole (0.21 g, 3.07 mmol). The resulting mixture was warmed to 45 °C and stirred for 24 h before it was cooled to room temperature and quenched with NH_4Cl (10 mL, sat. aq.) and water (10 mL). The resulting mixture was extracted with ethyl acetate (3 × 25 mL), the combined organic layer was washed with water (50 mL), brine (50 mL), dried (Na_2SO_4) and concentrated under reduced pressure to afford TBS ether **2m** (0.25 g, 81%) as an amorphous solid, which was used directly in the following step without further purification.

(ii) To a stirred solution of alkene **2m** (0.25 g, 0.62 mmol) in THF (15 mL) at −78 °C was added borane tetrahydrofuran complex (1.0 M in THF, 5.0 mL, 5.0 mmol). The resulting mixture was warmed to room temperature and stirred for 4 h before it was cooled to 0 °C and treated with NaOH (2.0 N aq., 2.5 mL, 5.0 mmol) and H_2O_2 (34.5% aq., 2.5 mL, 25.3 mmol). The resulting mixture was stirred for 4 h before it was quenched with NH_4Cl (10 mL, sat. aq.) and water (10 mL). The layers were separated and the aqueous layer was extracted with ethyl acetate (3 × 25 mL), the combined organic layer was washed with water (50 mL), brine (50 mL), dried (Na_2SO_4) and concentrated under reduced pressure. Flash column chromatography (silica gel, hexanes:EtOAc 6:1) afforded alcohol **2n** (0.23 g, 88%) as an amorphous white solid. **2n**: R_f = 0.19 (silica gel, hexanes:EtOAc 2:1); IR (film) ν_{max} 3615, 2932, 2856, 1576, 1470, 739 cm^{-1}; ^1H NMR (400 MHz, $CDCl_3$): δ 7.00–6.90 (m, 2H), 6.87 (d, *J* = 8.4 Hz, 2H), 6.73 (d, *J* = 7.0 Hz, 1H), 3.86 (s, 3H), 3.83 (s, 3H), 3.69–3.51 (m, 2H), 3.15 (s, 3H), 2.69–2.52 (m, 2H), 1.68 (s, 1H), 0.62 (s, 9H), 0.11 (s, 3H), –0.07 ppm (s, 3H); ^{13}C NMR (101 MHz, $CDCl_3$): δ 151.5, 150.1, 147.2, 142.2, 133.9, 130.6, 128.9, 124.6, 123.3, 120.3, 111.6, 110.4, 63.2, 60.5, 56.0, 54.6, 36.3, 25.5, 18.4, −3.9, −4.7 ppm; HRMS calcd. For $C_{23}H_{34}O_5SiNa^+$ [M + Na]$^+$ 441.2068, found 441.2066.

Allylic Alcohol 2o

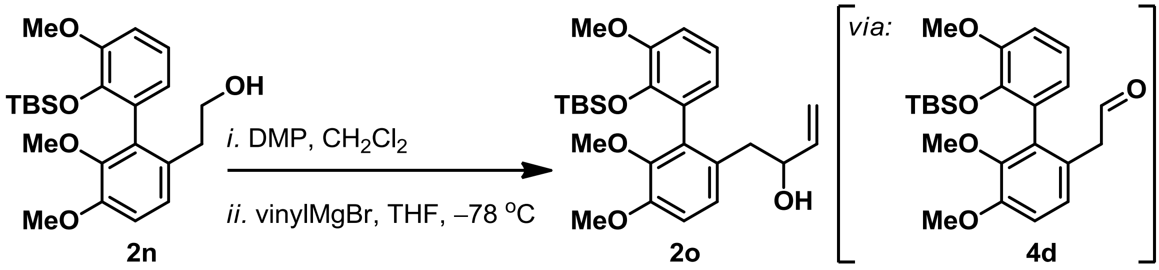

(i) To a stirred solution of alcohol **2n** (0.23 g, 0.55 mmol) in CH_2Cl_2 (15.0 mL) at 0 °C was added Dess-Martin periodinane (0.35 g, 0.83 mmol). The resulting mixture was warmed to room temperature and stirred for 3 h before it was quenched with $Na_2S_2O_3$ (40 mL, sat. aq.) and water (40 mL). The layers were separated and the aqueous layer was extracted with CH_2Cl_2 (3 × 100 mL), the combined organic layer was washed with water (50 mL), brine (50 mL), dried (Na_2SO_4) and concentrated under reduced pressure to afford crude aldehyde **4d** as an amorphous white solid, which was used directly in the following step without further purification.

(ii) To a stirred solution of crude aldehyde **4d** (obtained above) in THF (30.0 mL) at –78 °C was added vinyl magnesium bromide (1.0 M in THF, 2.0 mL, 2.0 mmol) dropwise. The resulting mixture was warmed to room temperature and stirred for 16 h before it was quenched with water (30 mL). The layers were separated and the aqueous layer was extracted with Et_2O (3 × 25 mL), the combined organic layer was washed with water (70 mL), brine (70 mL), dried (Na_2SO_4) and concentrated under reduced pressure. Flash column chromatography (silica gel, hexanes:EtOAc 6:1) afforded allylic alcohol **2o** (0.22 g, 90% over two steps) as an amorphous yellow solid. **2o**: R_f = 0.40 (silica gel, hexanes:EtOAc 4:1); IR (film) ν_{max} 3690, 3536, 3054, 1550, 1265, 706 cm^{-1}; ^{1}H NMR (400 MHz, $CDCl_3$): δ 7.00 (d, J = 8.0 Hz, 1H), 6.93 (t, J = 8.0 Hz, 1H), 6.86 (d, J = 8.0 Hz, 2H), 6.74 (d, J = 8.0 Hz, 1H), 5.75–5.62 (m, 1H), 5.12–4.94 (dd, J = 16.9, 8.7 Hz, 2H), 3.99 (br s, 1H), 3.87 (s, 3H), 3.82 (s, 3H), 3.59 (s, 3H), 2.67 (d, J = 14.0 Hz, 1H), 2.42 (dd, J = 9.0, 4.0 Hz, 1H), 1.72 (s, 1H), 0.61 (s, 9H), 0.12 (s, 3H), –0.10 ppm (s, 3H); ^{13}C NMR (101 MHz, $CDCl_3$): δ 151.7, 150.2, 147.2, 142.1, 140.7, 133.8, 130.3, 128.8, 125.4, 123.6, 120.3, 113.9, 111.5, 110.6, 73.1, 60.5, 55.9, 54.8, 41.4, 25.5, 18.4, –3.9, –4.8 ppm.

Enone 6i

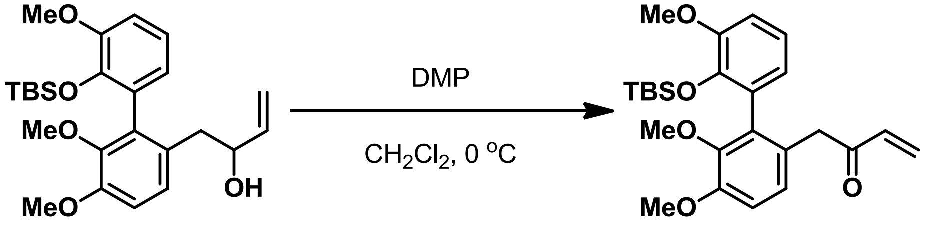

2o **6i**

To a stirred solution of alcohol **2o** (110 mg, 0.25 mmol) in CH_2Cl_2 (10.0 mL) at 0 °C was added Dess-Martin periodinane (209 mg, 0.49 mmol). The resulting mixture was warmed to room temperature and stirred for 3 h before it was quenched with $Na_2S_2O_3$ (10 mL, sat. aq.) and water (10 mL). The layers were separated and the aqueous layer was extracted with CH_2Cl_2 (3 × 10 mL), the combined organic layer was washed with water (30 mL), brine (30 mL), dried (Na_2SO_4) and concentrated under reduced pressure. Flash column chromatography (silica gel, hexanes:EtOAc 6:1) afforded enone **6i** (93.0 mg, 85%) as an amorphous white solid. **6i**: R_f = 0.60 (silica gel, hexanes:EtOAc 4:1); IR (film) ν_{max} 2853, 1809, 1647, 1459, 1065, 910 cm^{-1}; ^1H NMR (400 MHz, $CDCl_3$): δ 6.98–6.81 (m, 4H), 6.68 (d, *J* = 7.0 Hz, 1H), 6.13 (dd, *J* = 11.0, 7.0 Hz, 1H), 5.93 (d, *J* = 18.0 Hz, 1H), 5.56 (d, *J* = 11.0 Hz, 1H), 3.85 (s, 3H), 3.82 (s, 3H), 3.70 (d, *J* = 16.0 Hz, 1H), 3.60 (s, 3H), 3.46 (d, *J* = 16.5 Hz, 1H), 0.63 (s, 9H), 0.12 (s, 3H), –0.09 ppm (s, 3H); ^{13}C NMR (101 MHz, $CDCl_3$): δ 198.6, 151.8, 150.1, 147.1, 142.1, 135.7, 133.6, 128.6, 127.8, 126.9, 125.0, 123.6, 120.4, 111.6, 110.6, 60.4, 55.8, 54.7, 44.0, 25.4, 18.3, –4.1, –4.8 ppm; HRMS calcd. For $C_{25}H_{34}O_5SiNa^+$ $[M + Na]^+$ 465.2068, found 465.2066.

Optically Active Allylic Alcohol 6j and 6k

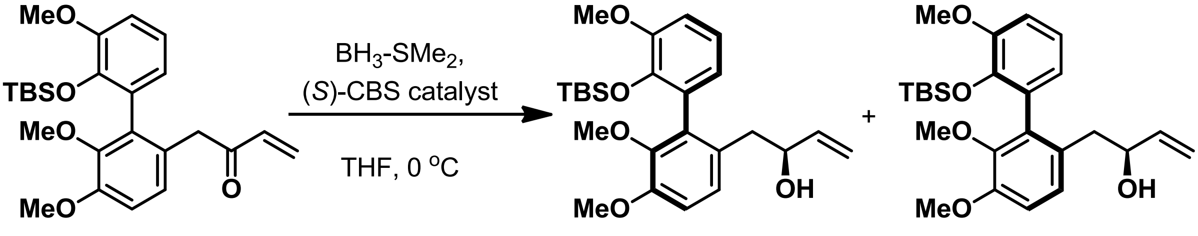

To a strirred solution of (*S*)–(–)–2–Methyl-CBS-oxazaborolidine (165 mg, 56 μmol) in THF (1.0 mL) at 0 °C was added borane dimethyl sulfide complex (1.0 M in THF, 0.37 mL, 0.37 mmol) dropwise. The resulting mixture was stirred for 10 min before the addition of a solution of enone **6i** (83.0 mg, 0.19 mmol) in THF (5.0 mL) over 2 h. The resulting mixture was stirred for 1 h before it was quenched with MeOH (5 mL) and water (5 mL). The layers were separated and the aqueous layer was extracted with ethyl acetate (3 × 10 mL), the combined organic layer was washed with water (15 mL), brine (15 mL), dried (Na_2SO_4) and concentrated under reduced pressure. Flash column chromatography (silica gel, hexanes:EtOAc 6:1) afforded allylic alcohols **6j** and **6k** (69.0 mg, 84%) as an amorphous white solid. **6j** (relative stereochemistry arbitrarily assigned): $[\alpha]_D^{25}$ = +10° (*c* = 0.1, $CHCl_3$); **6k** (relative stereochemistry arbitrarily assigned): $[\alpha]_D^{25}$ = –14° (*c* = 0.1, $CHCl_3$).

Chiral HPLC (ChiralPak1A, flow rate: 1.00 mL/min, isocratic hexane:isopropanol 98:2**)** **Chromatographs of Racemic (above) and Optically Active (below) Allylic Alcohol 6j** (relative stereochemistry arbitrarily assigned)

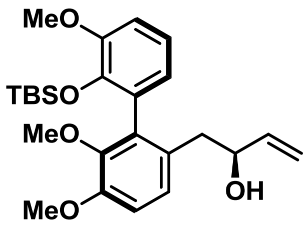

6j

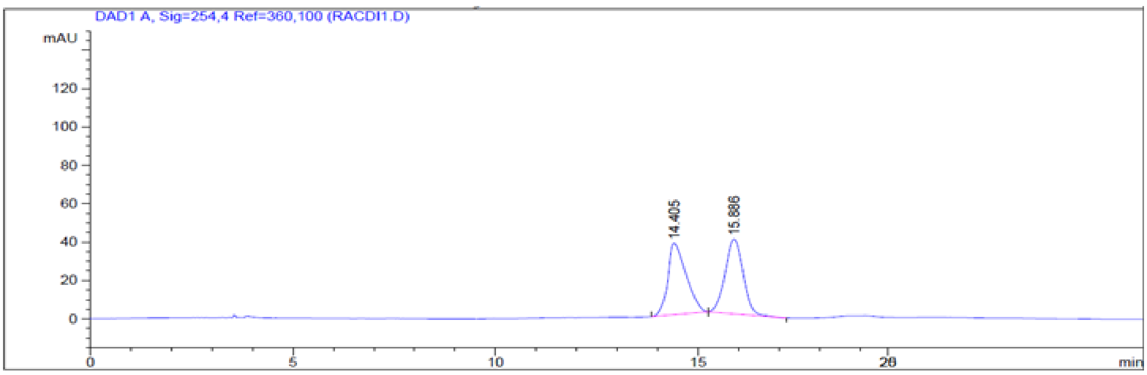

Signal 1: DAD1 A, Sig=254,4 Ref=360,100

Peak #	RetTime [min]	Type	Width [min]	Area [mAU*s]	Height [mAU]	Area %
1	14.405	PB	0.4729	1168.31641	37.14697	49.2450
2	15.886	BBA	0.4775	1204.13867	38.86147	50.7550
Totals :				2372.45508	76.00844	

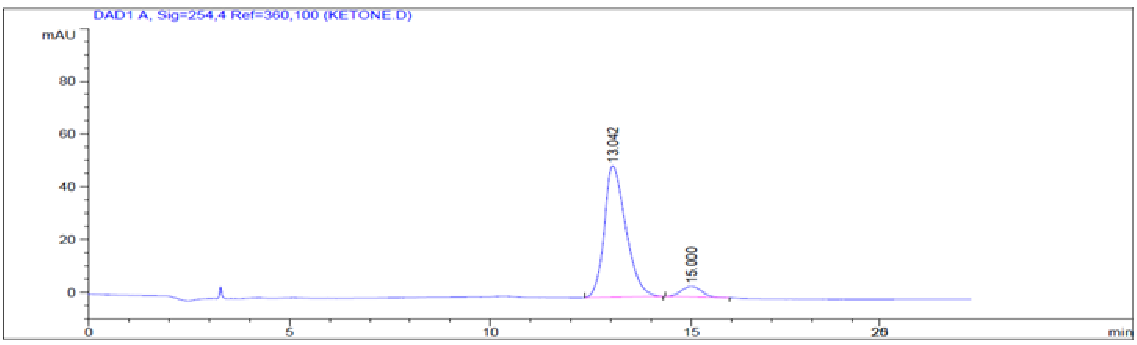

Signal 1: DAD1 A, Sig=254,4 Ref=360,100

Peak #	RetTime [min]	Type	Width [min]	Area [mAU*s]	Height [mAU]	Area %
1	13.042	BBA	0.5673	1851.99902	49.63151	93.0604
2	15.000	BBA	0.5499	138.10510	3.95039	6.9396
Totals :				1990.10413	53.58190	

Chiral HPLC (ChiralPak1A, flow rate: 1.00 mL/min, isocratic hexane:isopropanol 98:2) **Chromatographs of Racemic (above) and Optically Active (below) Allylic Alcohol 6k** (relative stereochemistry arbitrarily assigned)

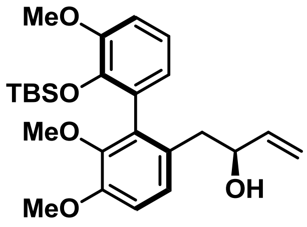

6k

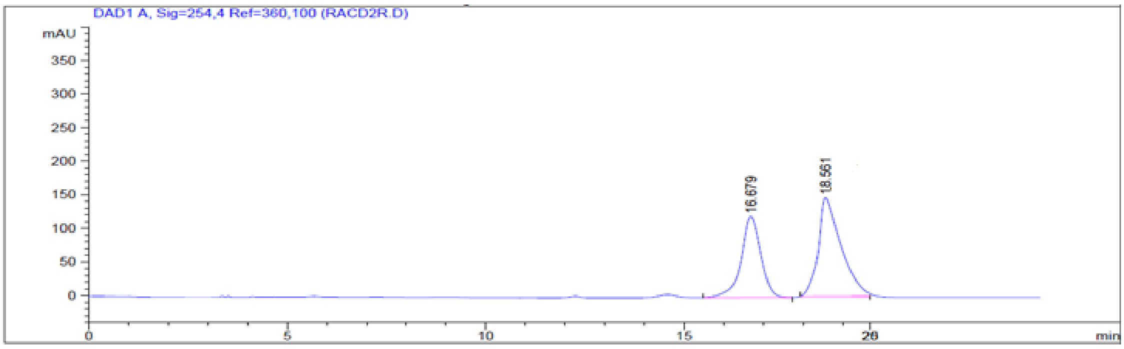

Signal 1: DAD1 A, Sig=254,4 Ref=360,100

Peak #	RetTime [min]	Type	Width [min]	Area [mAU*s]	Height [mAU]	Area %
1	16.679	BB	0.5136	4148.57080	120.50980	41.2988
2	18.561	MM	0.6676	5896.67822	147.21384	58.7012
Totals :				1.00452e4	267.72363	

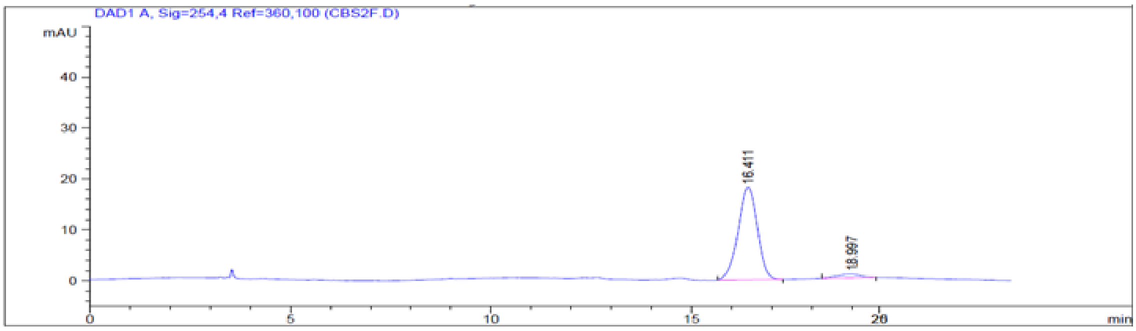

Signal 1: DAD1 A, Sig=254,4 Ref=360,100

Peak #	RetTime [min]	Type	Width [min]	Area [mAU*s]	Height [mAU]	Area %
1	16.411	BBA	0.5210	608.64044	18.15982	95.4314
2	18.997	MM	0.6843	29.13777	7.09636e-1	4.5686
Totals :				637.77821	18.86946	

Optically Active Allylic Alcohol 6g′

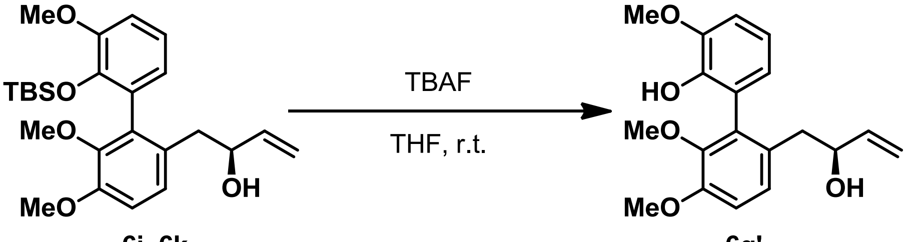

To a stirred solution of TBS ether **6j**/**6k** (66.0 mg, 0.15 mmol) in THF (1.5 mL) at room temperature was added TBAF (1.0 M in THF, 0.45 mL, 0.45 mmol). The resulting mixture was stirred for 1 h before it was diluted with water (10 mL). The resulting mixture was extracted with Et_2O (3 × 10 mL), the combined organic layer was washed with water (20 mL), brine (20 mL), dried (Na_2SO_4) and concentrated under reduced pressure. Flash column chromatography (silica gel, hexanes:EtOAc 5:1) afforded alcohol **6g′** (40.0 mg, 82%) as an amorphous solid. All physical data of allylic alcohol **6g′** are identical to those obtained for the racemic allylic alcohol **6g**.

Tetracycle 8g and 8g′

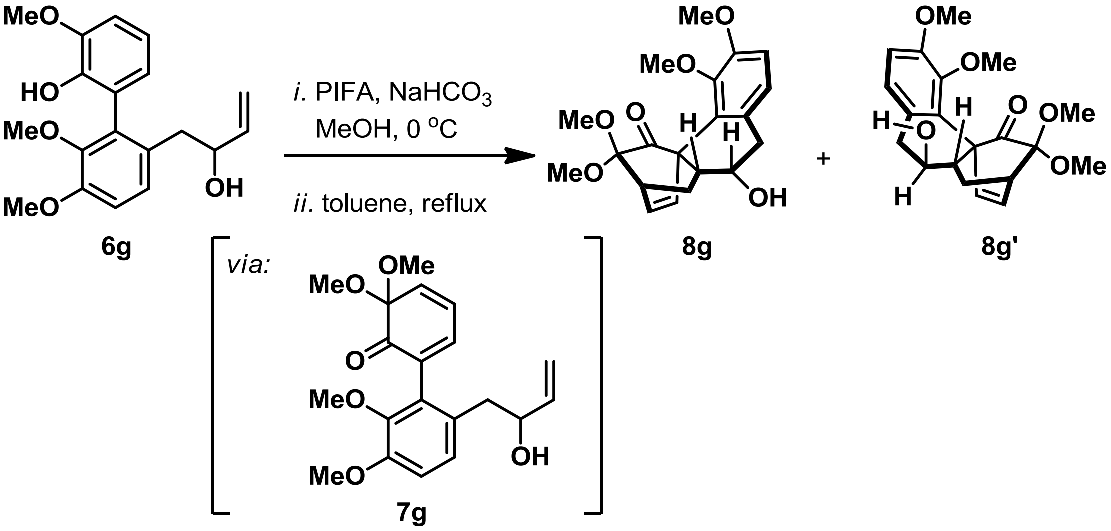

(i) To a stirred solution of phenol **6g** (115 mg, 0.35 mmol) in MeOH (15.0 mL) at 0 °C was added PIFA (164 mg, 0.38 mmol) and $NaHCO_3$ (350 mg, 4.17 mmol). The resulting mixture was stirred for 15 min before it was warmed to room temperature and quenched with $Na_2S_2O_3$ (20 mL, sat. aq.) and water (10 mL). The resulting mixture was extracted with Et_2O (3 × 30

mL), the combined organic layer was washed with $NaHCO_3$ (50 mL, sat. aq.), brine (50 mL), dried (Na_2SO_4) and concentrated under reduced pressure. Flash column chromatography (silica gel, CH_2Cl_2:Et_2O 15:1) afforded dienone **7g** as an orange amorphous solid.

(ii) Dienone **7g** (obtained above) was redissolved in toluene (30.0 mL), and warmed to reflux and stirred for 1 h before it was cooled to room temperature and concentrated under reduced pressure. Flash column chromatography (silica gel, hexanes:EtOAc 4:1) afforded tetracycles **8g** and **8g′** (~5:1 based on ^{1}H NMR analysis, 77.0 mg, 61% over two steps) as an amorphous yellow solid. **8g**+**8g′**: R_f = 0.30 (silica gel, hexanes:EtOAc 1:1); IR (film) ν_{max} 3475, 3002, 2861, 2705, 1690, 703 cm^{-1}; ^{1}H NMR (400 MHz, $CDCl_3$): δ 6.87 (d, *J* = 8.5 Hz, 1H), 6.81 (d, *J* = 8.4 Hz, 1H), 6.43 (t, *J* = 8.1 Hz, 1H), 5.87 (d, *J* = 8.2 Hz, 0.2H), 5.75 (d, *J* = 8.2 Hz, 0.8H), 3.84 (s, 3H), 3.79 (s, 3H), 3.55 (td, *J* = 10.7, 4.8 Hz, 1H), 3.39 (s, 6H), 3.19–3.13 (m, 1H), 3.02 (dd, *J* = 15.5, 4.8 Hz, 1H), 2.71 (dd, *J* = 15.5, 1.2 Hz, 1H), 2.28–2.18 (m, 1H), 2.15–2.05 (m, 1H), 1.51–1.42 ppm (m, 1H); ^{13}C NMR (101 MHz, $CDCl_3$): δ 199.2, 150.9, 147.2, 132.0, 131.4, 128.3, 127.9, 123.6, 113.0, 94.6, 70.6, 60.2, 57.0, 55.9, 50.8, 49.3, 44.2, 39.6, 37.7, 24.9 ppm; HRMS calcd. For $C_{20}H_{24}O_6Na^+$ $[M + Na]^+$ 383.1465, found 383.1466.

Tetracycle 8g and 8g′

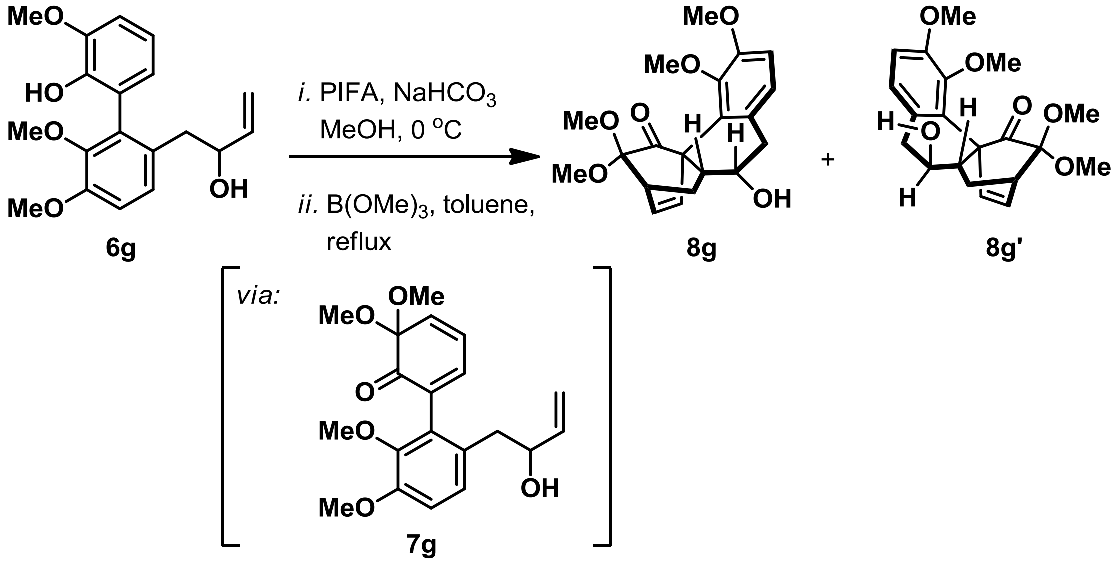

(i) To a stirred solution of phenol **6g** (282 mg, 0.85 mmol) in MeOH (17.0 mL) at 0 °C was added PIFA (302 mg, 0.94 mmol) and $NaHCO_3$ (860 mg, 10.2 mmol). The resulting mixture

was stirred for 15 min before it was warmed to room temperature and quenched with $Na_2S_2O_3$ (20 mL, sat. aq.) and water (10 mL). The resulting mixture was extracted with Et_2O (3 × 30 mL), the combined organic layer was washed with $NaHCO_3$ (70 mL, sat. aq.), brine (70 mL), dried (Na_2SO_4) and concentrated under reduced pressure. Flash column chromatography (silica gel, CH_2Cl_2:Et_2O 15:1) afforded dienone **7g** as an orange amorphous solid.

(ii) To a solution of dienone **7g** (obtained above) in toluene (30.0 mL) was added trimethyl borate (0.48 mL, 4.31 mmol). The resulting mixture was stirred for 30 min, warmed to reflux and stirred for 1 h before it was cooled to room temperature and concentrated under reduced pressure. Flash column chromatography (silica gel, hexanes:EtOAc 4:1) afforded tetracycles **8g** and **8g′** (~5:1 based on ^{1}H NMR analysis, 172 mg, 56% over two steps) as an amorphous yellow solid. All physical data of tetracycles **8g** and **8g′** are identical to those obtained from the intramolecular Diels-Alder reaction in the absence of $B(OMe)_3$.

TBS Ether 6h

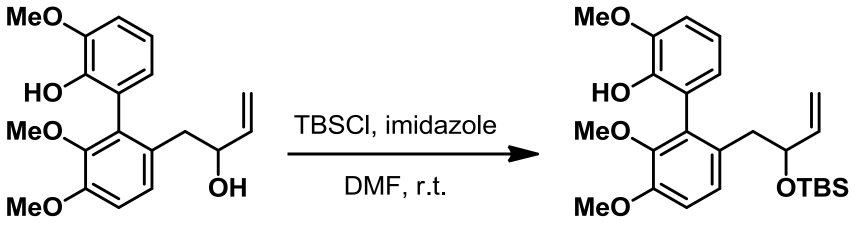

To a stirred solution of alcohol **6g** (134 mg, 0.41 mmol) in DMF (5.0 mL) at room temperature was added TBSCl (183 mg, 1.21 mmol) and imidazole (82.8 mg, 1.22 mmol). The resulting mixture was stirred for 24 h before it was quenched with NH_4Cl (10 mL, sat. aq.) and water (10 mL). The resulting mixture was extracted with Et_2O (3 × 20 mL), the combined organic layer was washed with water (50 mL), brine (50 mL), dried (Na_2SO_4) and concentrated under reduced pressure. Flash column chromatography (silica gel, hexanes:EtOAc 10:1) afforded TBS ether **6h** (127 mg, 70%) as an amorphous solid. **6h**: R_f = 0.37 (silica gel, hexanes:EtOAc 4:1); IR (film) ν_{max} 3622, 3460, 3155, 2987, 1590, 1280, 705 cm^{-1}; ^{1}H NMR (400 MHz, $CDCl_3$): δ 6.99 (d, J = 8.4 Hz, 1H), 6.95–6.84 (m, 3H), 6.76 (d, J = 7.2 Hz, 1H), 5.69–5.58 (m, 2H), 4.98–4.82 (m, 2H), 4.10–3.92 (m, 1H), 3.92 (s, 3H), 3.87 (s,

3H), 3.58 (s, 3H), 2.66 (dd, J = 13.4, 7.3 Hz, 1H), 2.53–2.48 (m, 1H), 0.80 (s, 9H), –0.18 (s, 3H), –0.26 ppm (s, 3H); ^{13}C NMR (101 MHz, $CDCl_3$): δ 151.2, 151.2, 146.9, 146.8, 146.8, 142.9, 142.8, 141.2, 141.2, 132.0, 131.8, 130.4, 130.2, 126.9, 126.6, 123.8, 123.5, 123.4, 119.3, 119.3, 113.3, 113.2, 111.5, 109.8, 73.7, 73.5, 60.5, 55.9, 55.8, 55.8, 42.3, 42.2, 25.8, 25.8, 18.1, –5.1, –5.1, –5.3, –5.4 ppm; HRMS calcd. For $C_{25}H_{36}O_5SiNa^+$ $[M + Na]^+$ 467.2224, found 467.2226.

Tetracycle 8h and 8h′

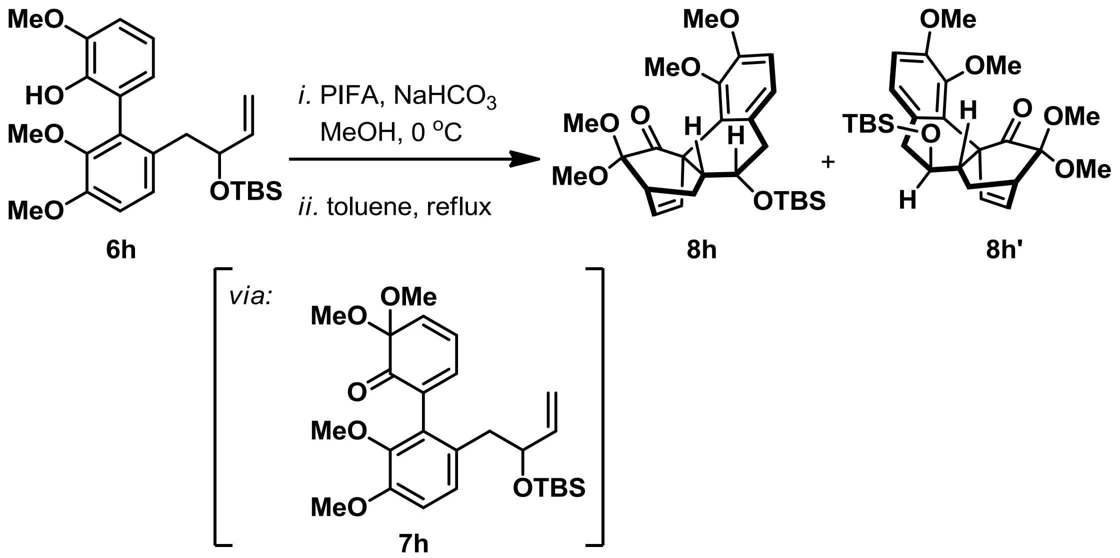

(i) To a stirred solution of phenol **6h** (135 mg, 0.30 mmol) in MeOH (20.0 mL) at 0 °C was added PIFA (137 mg, 0.32 mmol) and $NaHCO_3$ (860 mg, 10.2 mmol). The resulting mixture was stirred for 15 min before it was warmed to room temperature and quenched with $Na_2S_2O_3$ (20 mL, sat. aq.) and water (10 mL). The resulting mixture was extracted with Et_2O (3 × 30 mL), the combined organic layer was washed with $NaHCO_3$ (50 mL, sat. aq.), brine (50 mL), dried (Na_2SO_4) and concentrated under reduced pressure. Flash column chromatography (silica gel, CH_2Cl_2:Et_2O 15:1) afforded dienone **7h** as an orange amorphous solid.

(ii) Dienone **7h** (obtained above) was redissolved in toluene (30.0 mL), and warmed to reflux and stirred for 1 h before it was cooled to room temperature and concentrated under reduced pressure. Flash column chromatography (silica gel, hexanes:EtOAc 10:1) afforded tetracycles **8h** and **8h′** (~4:1 based on ^{1}H NMR analysis, 79.6 mg, 55% over two steps) as an amorphous

yellow solid. **8h**+**8h′**: R_f = 0.37 (silica gel, hexanes:EtOAc 4:1); IR (film) ν_{max} 3105, 3000, 1710, 1468, 808, 694 cm^{-1}; ^1H NMR (400 MHz, $CDCl_3$): δ 6.92–6.68 (m, 2H), 6.43 (t, *J* = 7.6 Hz, 0.7H), 6.16 (t, *J* = 7.6 Hz, 0.3H), 5.77 (d, *J* = 7.0 Hz, 1H), 3.84 (s, 3H), 3.80 (s, 3H), 3.60–3.50 (m, 1H), 3.39 (s, 6H), 3.18–3.05 (m, 1H), 2.92–2.80 (m, 1H), 2.80–2.68 (m, 1H), 2.22–2.10 (m, 2H), 1.42–1.30 (m, 1H), 0.90 (s, 7.2H), 0.71 (s, 1.8H), 0.07 (s, 2.2H), 0.05 (s, 2.2H), 0.00 (s, 0.9H), −0.08 ppm (s, 0.7H); ^{13}C NMR (101 MHz, $CDCl_3$): δ 199.3, 150.9, 147.3, 132.1, 131.3, 128.9, 128.0, 123.6, 113.0, 94.7, 71.3, 60.2, 57.0, 55.9, 50.7, 49.4, 44.4, 40.3, 37.7, 25.8, 25.6, 18.0, −3.9, −4.7 ppm; HRMS calcd. For $C_{26}H_{38}O_6SiNa^+$ $[M + Na]^+$ 497.2330, found 497.2327.

Ketone 8j and 8j′

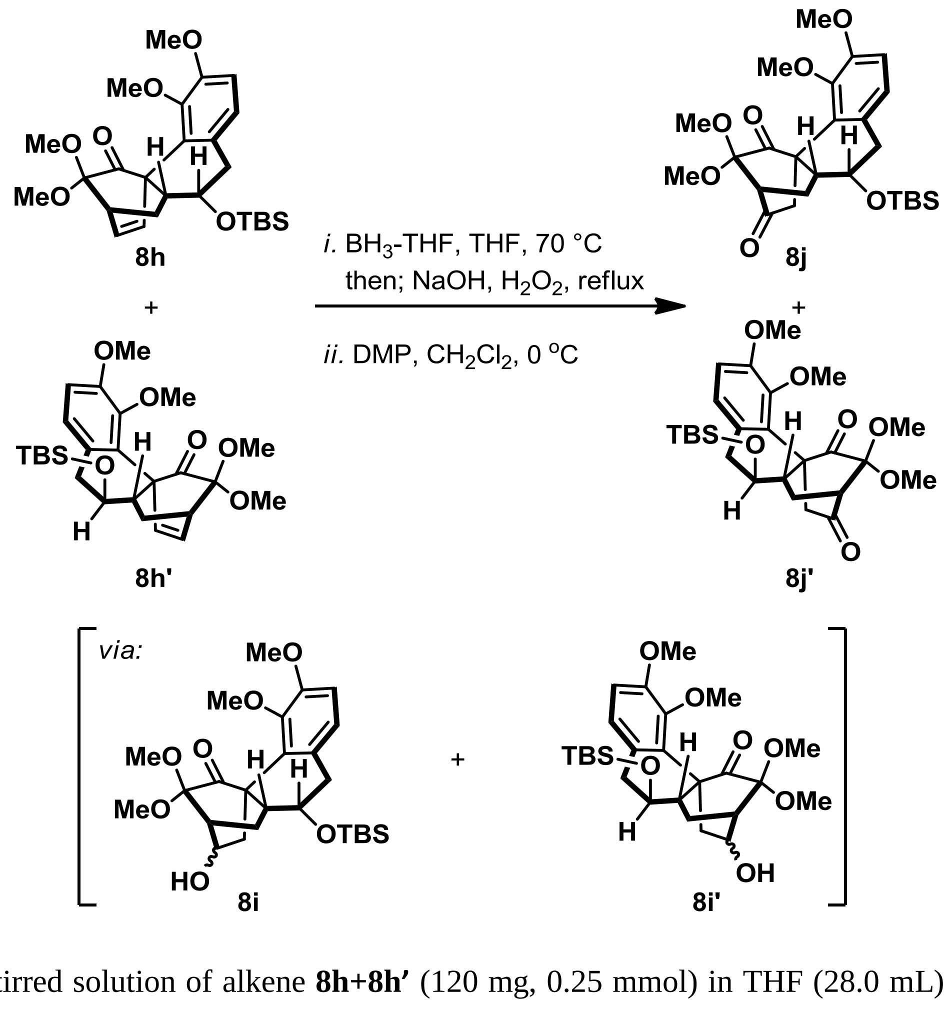

(i) To a stirred solution of alkene **8h+8h′** (120 mg, 0.25 mmol) in THF (28.0 mL) at −78 °C was added borane tetrahydrofuran complex (1.0 M in THF, 3.10 mL, 3.10 mmol). The

resulting mixture was warmed to room temperature and stirred for 4 h before it was cooled to 0 °C and treated with NaOH (2.0 N aq., 1.00 mL, 2.00 mmol) and H_2O_2 (34.5% aq., 1.00 mL, 10.1 mmol). The resulting mixture was stirred for 4 h before it was quenched with NH_4Cl (20 mL, sat. aq.) and water (20 mL). The layers were separated and the aqueous layer was extracted with ethyl acetate (3 × 30 mL), the combined organic layer was washed with water (50 mL), brine (50 mL), dried (Na_2SO_4), filtered and concentrated under reduced pressure. The resulting crude alcohols **8i** and **8i′** (98.0 mg, 79%) was used directly in the following step without further purification.

(ii) To a stirred solution of crude alcohol **8i**+**8i′** (obtained above) in CH_2Cl_2 (30.0 mL) at 0 °C was added Dess-Martin periodinane (370 mg, 0.87 mmol). The resulting mixture was warmed to room temperature and stirred for 3 h before it was quenched with $Na_2S_2O_3$ (40 mL, sat. aq.) and water (40 mL). The layers were separated and the aqueous layer was extracted with CH_2Cl_2 (3 × 100 mL), the combined organic layer was washed with water (50 mL), brine (50 mL), dried (Na_2SO_4) and concentrated under reduced pressure. Flash column chromatography (silica gel, hexanes:EtOAc 9:1) afforded ketones **8j** and **8j′** (65.0 mg, 67% combined yield) as an amorphous white solid. Major isomer **8j** was chromatographically separated and advanced forward in the subsequent steps. **8j**: R_f = 0.23 (silica gel, hexanes:Et_2O 4:1); IR (film) ν_{max} 2852, 2682, 1809, 1647, 1459, 1181 cm^{-1}; ^1H NMR (400 MHz, $CDCl_3$): δ 6.87 (d, *J* = 8.5 Hz, 1H), 6.79 (d, *J* = 8.4 Hz, 1H), 3.88 (dd, *J* = 10.5, 5.1 Hz, 1H), 3.83 (s, 3H), 3.82 (s, 3H), 3.42 (s, 3H), 3.41 (s, 3H), 3.35 (d, *J* = 19.5 Hz, 1H), 3.16–3.11 (m, 1H), 2.97 (dd, *J* = 15.7, 5.1 Hz, 1H), 2.72 (dd, *J* = 15.7, 10.3 Hz, 1H), 2.53 (d, *J* = 19.5 Hz, 1H), 2.36–2.26 (m, 1H), 2.22–2.12 (m, 1H), 1.76 (dd, *J* = 14.7, 6.5 Hz, 1H), 0.90 (s, 9H), 0.11 (s, 3H), 0.07 ppm (s, 3H); ^{13}C NMR (101 MHz, $CDCl_3$): δ 207.1, 200.6, 150.9, 146.7, 127.9, 126.5, 124.2, 113.3, 96.8, 68.9, 60.0, 55.9, 53.5, 51.4, 50.7, 49.7, 43.2, 41.8, 40.1, 25.7, 22.0, 18.0, −3.8, −4.8 ppm; HRMS calcd. For $C_{26}H_{38}O_7SiNa^+$ $[M + Na]^+$ 513.2279, found 513.2279.

Mesylate 8l

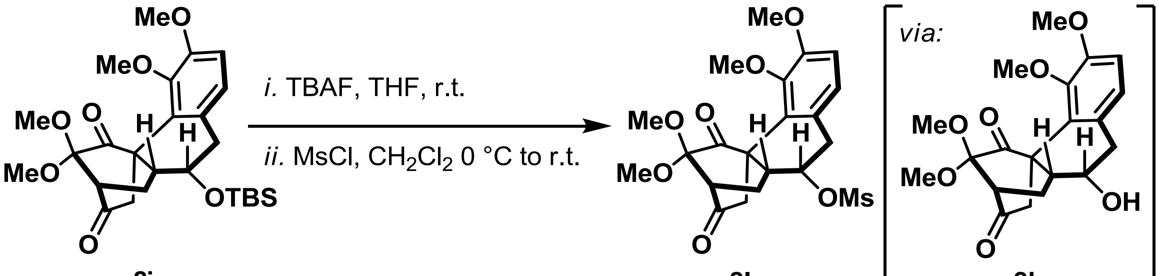

8j 8l 8k

(i) To a stirred solution of TBS ether **8j** (40.0 mg, 82 μmol) in THF (5.0 mL) at room temperature was added TBAF (1.0 M in THF, 0.30 mL, 0.30 mmol). The resulting mixture was stirred for 1 h before it was diluted with water (10 mL). The resulting mixture was extracted with CH_2Cl_2 (3 × 10 mL), the combined organic layer was washed with water (40 mL), brine (40 mL), dried (Na_2SO_4) and concentrated under reduced pressure. Flash column chromatography (silica gel, hexanes:EtOAc 5:1) afforded alcohol **8k** (28.2 mg, 92%) as an amorphous solid. **8k**: R_f = 0.13 (silica gel, hexanes:EtOAc 1:1); IR (film) ν_{max} 3452, 3067, 2825, 1704, 1453, 676 cm^{-1}; ^{1}H NMR (400 MHz, $CDCl_3$): δ 6.88 (d, *J* = 8.4 Hz, 1H), 6.82 (d, *J* = 8.4 Hz, 1H), 3.97–3.87 (m, 1H), 3.84 (s, 3H), 3.83 (s, 3H), 3.43 (s, 3H), 3.41 (s, 3H), 3.34 (d, *J* = 19.8 Hz, 1H), 3.19–3.06 (m, 2H), 2.77–2.68 (m, 1H), 2.52 (d, *J* = 19.5 Hz, 1H), 2.44–2.34 (m, 1H), 2.21–2.09 (m, 1H), 1.95–1.87 (m, 1H), 1.75 ppm (br s, 1H); ^{13}C NMR (101 MHz, $CDCl_3$): δ 206.9, 200.7, 150.9, 146.6, 127.4, 126.4, 124.2, 113.3, 96.8, 68.1, 60.0, 55.9, 53.6, 51.3, 50.8, 49.6, 42.9, 41.8, 39.6, 21.3 ppm.

(ii) To a stirred solution of alcohol **8k** (25.0 mg, 66 μmol) in CH_2Cl_2 (5.0 mL) at 0 ^{o}C was added MsCl (26 μL, 0.34 mmol). The resulting mixture was stirred for 1 h before it was diluted with water (10 mL). The resulting mixture was extracted with CH_2Cl_2 (3 × 10 mL), the combined organic layer was washed with water (40 mL), brine (40 mL), dried (Na_2SO_4) and concentrated under reduced pressure. Flash column chromatography (silica gel, hexanes:EtOAc 5:1) afforded mesylate **8l** (28.0 mg, 93%) as an amorphous solid. **8l**: R_f = 0.23 (silica gel, hexanes:EtOAc 1:1); IR (film) ν_{max} 3013, 2894, 1704, 1632, 1162, 708 cm^{-1}; ^{1}H NMR (400 MHz, $CDCl_3$): δ 6.90 (d, *J* = 8.5 Hz, 1H), 6.81 (d, *J* = 8.5 Hz, 1H), 4.96–4.84 (m, 1H), 3.84 (s, 3H), 3.82 (s, 3H), 3.43 (s, 3H), 3.40 (s, 3H), 3.36 (d, *J* = 18.2 Hz, 1H), 3.17 (s, 1H), 3.09 (s, 3H), 3.07–2.98 (m, 1H), 2.57 (d, *J* = 18.2 Hz, 1H), 2.47–2.36 (m, 2H), 1.94–1.80

ppm (m, 1H); ^{13}C NMR (101 MHz, $CDCl_3$): δ 205.4, 200.0, 151.3, 146.6, 125.5, 124.3, 113.6, 96.6, 78.2, 60.1, 55.9, 53.5, 50.8, 49.7, 41.6, 40.4, 39.0, 37.0, 21.6, 9.3, –0.0 ppm; HRMS calcd. For $C_{21}H_{26}O_9SNa^+$ $[M + Na]^+$ 477.1190, found 477.1188.

Alkene 9

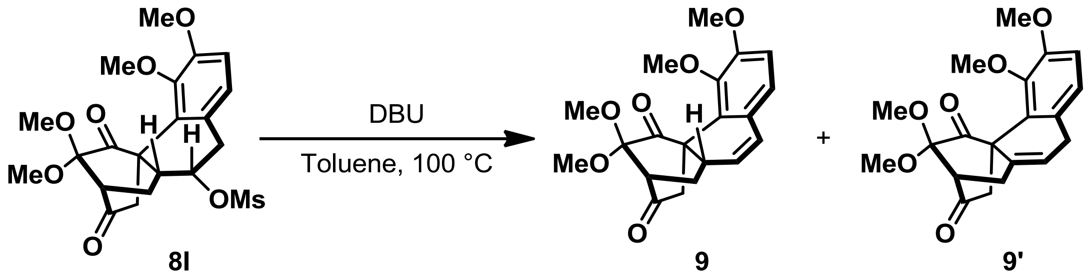

8l 9 9'

To a stirred solution of mesylate **8l** (8.0 mg, 18 μmol) in toluene (1.2 mL) at room temperature was added DBU (0.10 mL, 0.67 mmol). The resulting mixture was heated to reflux and stirred for 4 h before it was cooled to room temperature and quenched with NH_4Cl (2 mL, sat. aq.) and water (2 mL). The resulting mixture was extracted with CH_2Cl_2 (3 × 3 mL), the combined organic layer was washed with water (10 mL), brine (10 mL), dried (Na_2SO_4) and concentrated under reduced pressure. Flash column chromatography (silica gel, hexanes:EtOAc 5:1) afforded alkene **9** (as a ~1:2 mixture with **9′** based on ^{1}H NMR analysis, 4.5 mg, 71% combined yield). All physical data of alkene **9** are identical to those obtained from the PTSA mediated elimination of TBS ethers **8b** and **8b′**.

^{1}H NMR ($CDCl_3$, 400 MHz): crude reaction mixture

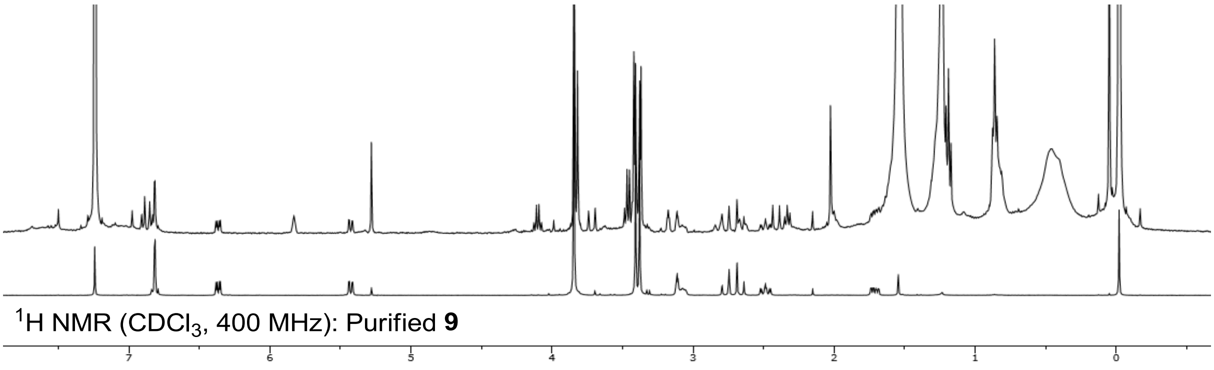

^{1}H NMR ($CDCl_3$, 400 MHz): Purified **9**

II) X-ray Crystallographic Analysis of Compound 12a

Data Collection

A colorless crystal with approximate dimensions 0.2 × 0.1 × 0.08 mm^3 was selected under oil under ambient conditions and attached to the tip of a MiTeGen MicroMount©. The crystal was mounted in a stream of cold nitrogen at 120 K and centered in the X-ray beam by using a video camera. The crystal evaluation and data collection were performed on a Bruker D8 Venture diffractometer with Mo K_α (λ = 0.71073 Å) radiation and the diffractometer to crystal distance of 4.00 cm. The initial cell constants were obtained from two series of ω scans at different starting angles. Each series consisted of 12 frames collected at intervals of 0.5° in a 6° range about ω with the exposure time of 10 seconds per frame. The reflections were successfully indexed by an automated indexing routine built in the APEXII program. The final cell constants were calculated from a set of 4218 strong reflections from the actual data collection. The data were collected by using the half sphere data collection routine to survey the reciprocal space to the extent of a full sphere to a resolution of 0.81 Å. A total of 18865 data were harvested by collecting 3 sets of frames with 0.5° scans in ω and φ with an exposure time 10 sec per frame. These highly redundant datasets were corrected for Lorentz and polarization effects. The absorption correction was based on fitting a function to the empirical transmission surface as sampled by multiple equivalent measurements.[1]

Structure Solution and Refinement

The systematic absences in the diffraction data were uniquely consistent for the space group $P2_1/c$ that yielded chemically reasonable and computationally stable results of refinement.[2,3] A successful solution by the direct methods provided most non-hydrogen atoms from the *E*-map. The remaining non-hydrogen atoms were located in an alternating series of least-squares cycles and difference Fourier maps. All non-hydrogen atoms were refined with anisotropic displacement coefficients. All hydrogen atoms were included in the structure factor calculation at idealized positions and were allowed to ride on the neighboring atoms with relative isotropic displacement coefficients. The final least-squares refinement of 250 parameters against 3815 data resulted in residuals *R* (based on F^2 for $I \geq 2\sigma$) and *wR* (based on

F^2 for all data) of 0.0464 and 0.1058, respectively. The final difference Fourier map was featureless.

Summary

Crystal Data for $C_{21}H_{29}NO_5$ (M =375.45 g/mol): monoclinic, space group $P2_1/c$ (no. 14), a = 19.8561(10) Å, b = 7.9076(4) Å, c = 12.5788(6) Å, β = 101.5550(16)°, V = 1935.02(17) Å^3, Z = 4, T = 120.0 K, μ(MoKα) = 0.091 mm^{-1}, $Dcalc$ = 1.289 g/cm^3, 18865 reflections measured (5.562° ≤ 2Θ ≤ 52.11°), 3815 unique (R_{int} = 0.0635, R_{sigma} = 0.0473) which were used in all calculations. The final R_1 was 0.0464 (I > 2σ(I)) and wR_2 was 0.1058 (all data).

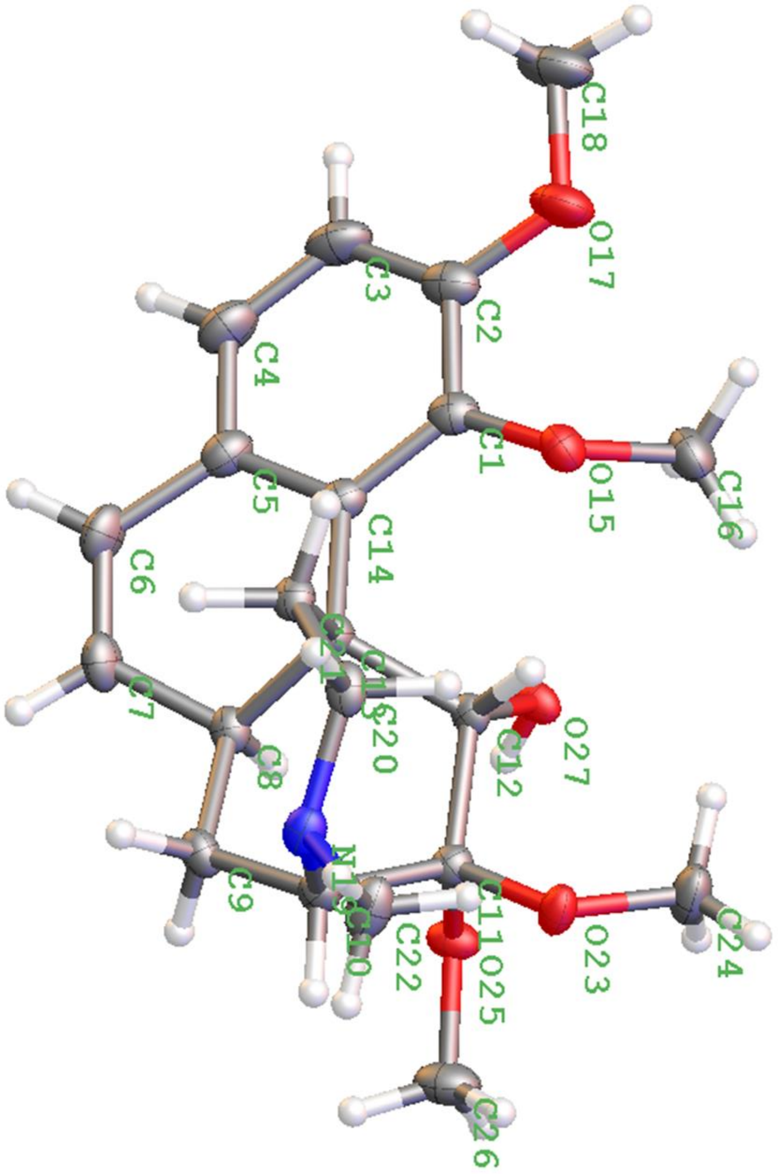

Table 1: Crystal data and structure refinement for RC438 (Compound 12a)

Identification code	RC438
Empirical formula	$C_{21}H_{29}NO_5$
Formula weight	375.45
Temperature/K	120.0
Crystal system	monoclinic
Space group	$P2_1/c$
a/Å	19.8561(10)
b/Å	7.9076(4)
c/Å	12.5788(6)
α/°	90
β/°	101.5550(16)
γ/°	90
Volume/Å ^{3}	1935.02(17)
Z	4
ρ_{calc}g/cm^3	1.289
μ/mm^{-1}	0.091
F(000)	808.0
Crystal size/mm^3	0.2 × 0.1 × 0.08
Radiation	MoKα (λ = 0.71073)
2Θ range for data collection/°	5.562 to 52.11
Index ranges	-24 ≤ h ≤ 24, -9 ≤ k ≤ 9, -15 ≤ l ≤ 15
Reflections collected	18865
Independent reflections	3815 [R_{int} = 0.0635, R_{sigma} = 0.0473]
Data/restraints/parameters	3815/231/250
Goodness-of-fit on F^2	1.016
Final R indexes [I>=2σ (I)]	R_1 = 0.0464, wR_2 = 0.0924
Final R indexes [all data]	R_1 = 0.0848, wR_2 = 0.1058
Largest diff. peak/hole / e Å $^{-3}$	0.27/-0.22

Table 2: Fractional Atomic Coordinates (×10[4]) and Equivalent Isotropic Displacement Parameters (Å ^{2}×10^3) for RC438 (Compound 12a). U_{eq} is defined as 1/3 of of the trace of the orthogonalised U_{IJ} tensor.

Atom	*x*	*y*	*z*	U(eq)
O15	6629.5(6)	10358.3(16)	5135.9(10)	24.1(3)
O17	5403.2(7)	9886(2)	3876.3(11)	37.1(4)
O23	8961.7(6)	10526.3(16)	6884.4(10)	22.8(3)
O25	9054.6(6)	8000.9(17)	5969.1(10)	22.9(3)
O27	7899.6(6)	8865.8(17)	4841.5(9)	20.4(3)
N19	8417.0(8)	8788(2)	8555.9(12)	20.9(4)
C1	6391.0(9)	8722(3)	4971.6(15)	22.0(4)
C2	5735.1(10)	8453(3)	4322.0(16)	28.3(5)
C3	5463(1)	6850(3)	4203.1(17)	33.8(5)
C4	5823.3(10)	5527(3)	4760.8(16)	32.3(5)
C5	6462.8(10)	5770(2)	5438.9(15)	24.6(4)
C6	6799.9(11)	4356(2)	6078.2(16)	28.3(5)
C7	7446.8(10)	4454(2)	6610.9(15)	25.2(5)
C8	7863.2(9)	6009(2)	6530.9(14)	19.2(4)
C9	8471.3(9)	6153(2)	7500.3(14)	21.0(4)
C10	8735.8(9)	7970(2)	7731.0(14)	19.3(4)
C11	8674.7(9)	8893(2)	6638.3(14)	18.2(4)
C12	7922.2(9)	8987(2)	5969.4(13)	16.7(4)
C13	7444.6(9)	7661(2)	6361.9(14)	17.8(4)
C14	6765.5(9)	7393(2)	5532.8(14)	20.0(4)
C16	6738.1(11)	11251(3)	4181.7(16)	30.7(5)
C18	4698.7(10)	9706(4)	3343.6(18)	44.2(6)
C20	7723.6(10)	9454(2)	8154.9(15)	22.4(4)
C21	7235.0(9)	8266(2)	7433.7(14)	20.2(4)
C22	8845.4(10)	10093(3)	9177.9(15)	29.4(5)
C24	8959.2(11)	11595(3)	5968.8(16)	31.4(5)
C26	9776.7(9)	7837(3)	6365.8(17)	34.4(5)

Table 3: Anisotropic Displacement Parameters (Å ^{2}×10^3) for RC438 (Compound 12a). The Anisotropic displacement factor exponent takes the form: $-2\pi^2[h^2a^{*2}U_{11}+2hka^*b^*U_{12}+\ldots]$.

Atom	U_{11}	U_{22}	U_{33}	U_{23}	U_{13}	U_{12}
O15	26.3(7)	23.5(8)	22.2(7)	1.7(6)	4.4(5)	3.5(6)
O17	23.0(8)	51.6(10)	34.0(8)	8.0(7)	-0.8(6)	8.2(7)
O23	26.6(7)	23.8(7)	17.7(7)	3.4(6)	3.3(5)	-7.9(6)
O25	18.2(7)	32.7(8)	17.7(7)	-1.0(6)	3.7(5)	5.1(6)
O27	21.9(7)	26.2(8)	13.1(6)	1.8(6)	3.4(5)	6.0(6)
N19	26.4(8)	21.7(9)	14.8(8)	-2.8(7)	4.5(6)	-5.0(7)
C1	20.1(9)	27.4(11)	19.5(10)	-3.5(8)	6.2(7)	0.3(8)
C2	23.3(10)	40.9(13)	20.9(10)	-1.1(9)	5.0(8)	2.8(9)
C3	21.4(11)	50.3(14)	28.0(12)	-6.7(10)	1.0(9)	-7.2(10)
C4	29.0(11)	35.6(13)	32.6(12)	-9.5(10)	7.0(9)	-11.6(9)
C5	23.3(10)	27.2(11)	24.3(10)	-6.0(9)	7.5(8)	-4.8(8)
C6	36.6(12)	19.4(11)	30.1(11)	-5.2(9)	9.7(9)	-9.5(9)
C7	38.0(12)	15.3(10)	22.9(10)	-1.1(8)	7.4(9)	-0.2(8)
C8	24.9(10)	18.6(10)	14.8(9)	-0.7(8)	5.5(7)	0.8(8)
C9	27.2(10)	19.4(10)	16.4(9)	1.8(8)	4.3(8)	3.6(8)
C10	19.6(9)	22.1(10)	15.3(9)	1.1(8)	1.0(7)	0.0(8)
C11	19.1(9)	20(1)	16.0(9)	-1.5(8)	5.1(7)	-0.8(8)
C12	20.0(9)	18.6(10)	11.5(9)	0.7(7)	3.5(7)	1.0(8)
C13	20.6(9)	16.8(10)	16.4(9)	-1.2(8)	4.4(7)	0.0(8)
C14	19.5(9)	23.8(10)	17.5(10)	-4.4(8)	5.6(7)	-1.9(8)
C16	34.1(11)	29.1(12)	29.2(11)	7.6(10)	7.2(9)	7.1(10)
C18	22.6(11)	74.2(19)	32.8(12)	6.3(12)	-1.4(9)	10.1(11)
C20	33.7(11)	19.5(10)	16.4(9)	-1.0(8)	10.4(8)	0.0(8)
C21	22.5(10)	21.8(10)	17.9(9)	0.0(8)	8.0(8)	0.2(8)
C22	41.0(12)	28.6(12)	18.9(10)	-5.1(9)	7.1(9)	-12.9(10)
C24	37.2(12)	30.8(12)	25.7(11)	6.9(9)	4.8(9)	-12.2(10)
C26	21.3(11)	51.6(15)	30.3(12)	-0.5(11)	4.8(9)	6.7(10)

Table 4: Bond Lengths for RC438 (Compound 12a).

Atom	Atom	Length/Å		Atom	Atom	Length/Å
O15	C1	1.379(2)		C3	C4	1.378(3)
O15	C16	1.445(2)		C4	C5	1.394(3)
O17	C2	1.373(2)		C5	C6	1.459(3)
O17	C18	1.432(2)		C5	C14	1.412(3)
O23	C11	1.421(2)		C6	C7	1.327(3)
O23	C24	1.428(2)		C7	C8	1.496(3)
O25	C11	1.425(2)		C8	C9	1.539(2)
O25	C26	1.427(2)		C8	C13	1.540(3)
O27	C12	1.414(2)		C9	C10	1.537(3)
N19	C10	1.469(2)		C10	C11	1.539(2)
N19	C20	1.466(2)		C11	C12	1.563(2)
N19	C22	1.461(2)		C12	C13	1.559(2)
C1	C2	1.408(3)		C13	C14	1.545(2)
C1	C14	1.395(3)		C13	C21	1.564(2)
C2	C3	1.374(3)		C20	C21	1.515(3)

Table 5: Bond Angles for RC438 (Compound 12a)

Atom	Atom	Atom	Angle/°		Atom	Atom	Atom	Angle/°
C1	O15	C16	115.94(15)		N19	C10	C9	111.44(14)
C2	O17	C18	116.93(18)		N19	C10	C11	116.99(15)
C11	O23	C24	115.22(14)		C9	C10	C11	108.17(14)
C11	O25	C26	116.89(14)		O23	C11	O25	110.01(13)
C20	N19	C10	115.02(13)		O23	C11	C10	106.27(14)
C22	N19	C10	113.72(14)		O23	C11	C12	111.86(15)
C22	N19	C20	109.64(15)		O25	C11	C10	109.67(15)
O15	C1	C2	118.61(17)		O25	C11	C12	105.53(13)
O15	C1	C14	119.93(16)		C10	C11	C12	113.52(14)
C14	C1	C2	121.13(18)		O27	C12	C11	111.83(13)
O17	C2	C1	115.12(18)		O27	C12	C13	111.90(14)
O17	C2	C3	124.81(18)		C13	C12	C11	111.96(14)
C3	C2	C1	120.03(19)		C8	C13	C12	105.59(14)
C2	C3	C4	119.50(19)		C8	C13	C14	110.53(15)
C3	C4	C5	121.6(2)		C8	C13	C21	111.74(15)
C4	C5	C6	119.54(18)		C12	C13	C21	110.53(14)
C4	C5	C14	119.78(19)		C14	C13	C12	112.48(14)
C14	C5	C6	120.64(17)		C14	C13	C21	106.08(13)
C7	C6	C5	121.66(18)		C1	C14	C5	117.89(17)
C6	C7	C8	120.39(18)		C1	C14	C13	122.88(16)
C7	C8	C9	111.35(15)		C5	C14	C13	118.71(17)
C7	C8	C13	114.59(15)		N19	C20	C21	115.33(15)
C9	C8	C13	111.25(15)		C20	C21	C13	117.21(14)
C10	C9	C8	113.77(15)					

Table 6: Hydrogen Atom Coordinates (Å × 10^4) and Isotropic Displacement Parameters (Å ^{2}× 10^3) for RC438 (Compound 12a)

Atom	*x*	*y*	*z*	U(eq)
H27	8165	8093	4723	31
H3	5030	6656	3740	41
H4	5631	4423	4681	39
H6	6550	3341	6114	34
H7	7647	3531	7046	30
H8	8067	5862	5872	23
H9A	8328	5704	8156	25
H9B	8853	5439	7358	25
H10	9238	7880	8049	23
H12	7740	10130	6104	20
H16A	6771	10435	3608	46
H16B	7166	11903	4360	46
H16C	6352	12019	3930	46
H18A	4670	8977	2706	66
H18B	4507	10821	3115	66
H18C	4437	9198	3845	66
H20A	7762	10507	7745	27
H20B	7521	9758	8787	27
H21A	6782	8833	7238	24
H21B	7173	7252	7866	24
H22A	9317	9672	9401	44
H22B	8662	10380	9823	44
H22C	8845	11104	8727	44
H24A	9184	11013	5447	47
H24B	9208	12643	6207	47
H24C	8484	11865	5624	47
H26A	10006	7625	5757	52
H26B	9865	6891	6878	52
H26C	9956	8883	6735	52

III) Abbreviations

Cy = cyclohexyl

DBU = 1,8-diazabicyclo[5.4.0]-7-undecene

DCC = *N,N'*-dicyclohexylcarbodiimide

DDQ = 2,3-dichloro-5,6-dicyano-1,4-benzoquinone

Dibal-H = diisobutylaluminium hydride

DMF = *N*,*N'*-dimethylformamide

DMAP = *N,N'*-dimethylaminopyridine

DMP = Dess-Martin periodinane

LDA = lithium diisopropylamide

L-selectride = lithium tri-sec-butylborohydride

MOMCl = chloromethyl methyl ether

MOM = methoxymethyl

Ms = methanesulfonyl

MsCl = methanesulfonyl Chloride

PCC = pyridinium chlorochromate

PivOH = pivalic acid

PIDA = [bis(acetoxy)iodo]benzene

PIFA = [bis(trifluoroacetoxy)iodo]benzene

PTSA = *p*-toluenesulfonic acid

PyH•Br_3 = pyridnium tribromide

TBAB = tetrabutylammonium bromide

TBHP = *tert*-butyl hydroperoxide

TBAF = tetrabutylammonium fluoride

TBS = *tert*-butyldimethylsilyl

TBSCl = *tert*-butyldimethylsilyl chloride

TMSCl = trimethylsilyl chloride

TsCl = *p*-toluenesulfonyl chloride

Ts = *p*-toluenesulfonyl

IV) References

1. Bruker-AXS (2007-2013), APEX2 (Ver. 2013.2-0), SADABS (2012-1) and SAINT+ (Ver. 8.30C) Software Reference Manuals. Bruker-AXS, Madison, Wisconsin, USA.
2. Sheldrick, G. M. *Acta Cryst.* **2008**, *A64*, 112−122.
3. Dolomanov, O.V.; Bourhis, L.J.; Gildea, R.J.; Howard, J.A.K.; Puschmann, H. *J. Appl. Cryst.* **2009**, *42*, 339−341.

V) ^{1}H and ^{13}C NMR Spectra for Compounds

^{1}H NMR (400 MHz, $CDCl_3$)

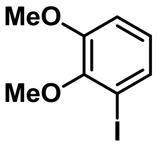

2

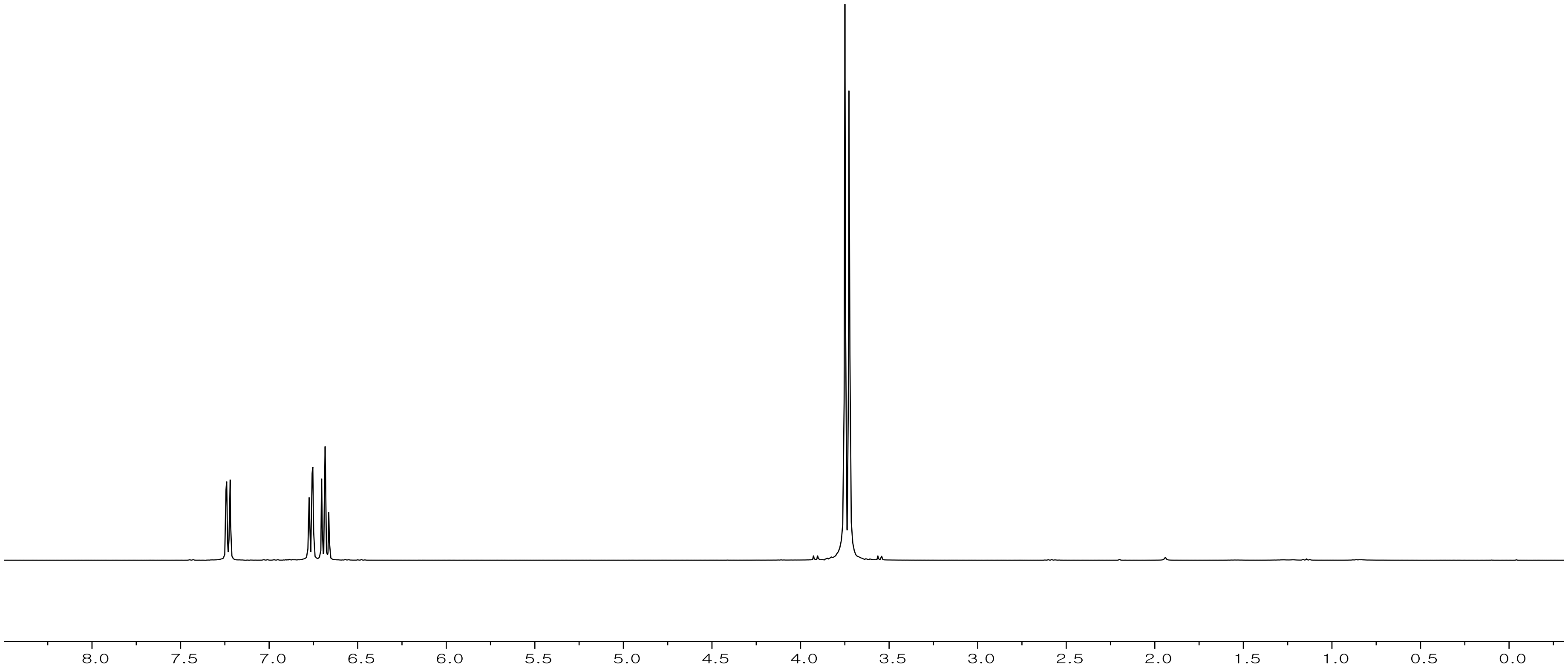

^{13}C NMR (101 MHz, $CDCl_3$)

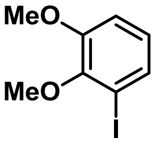

2

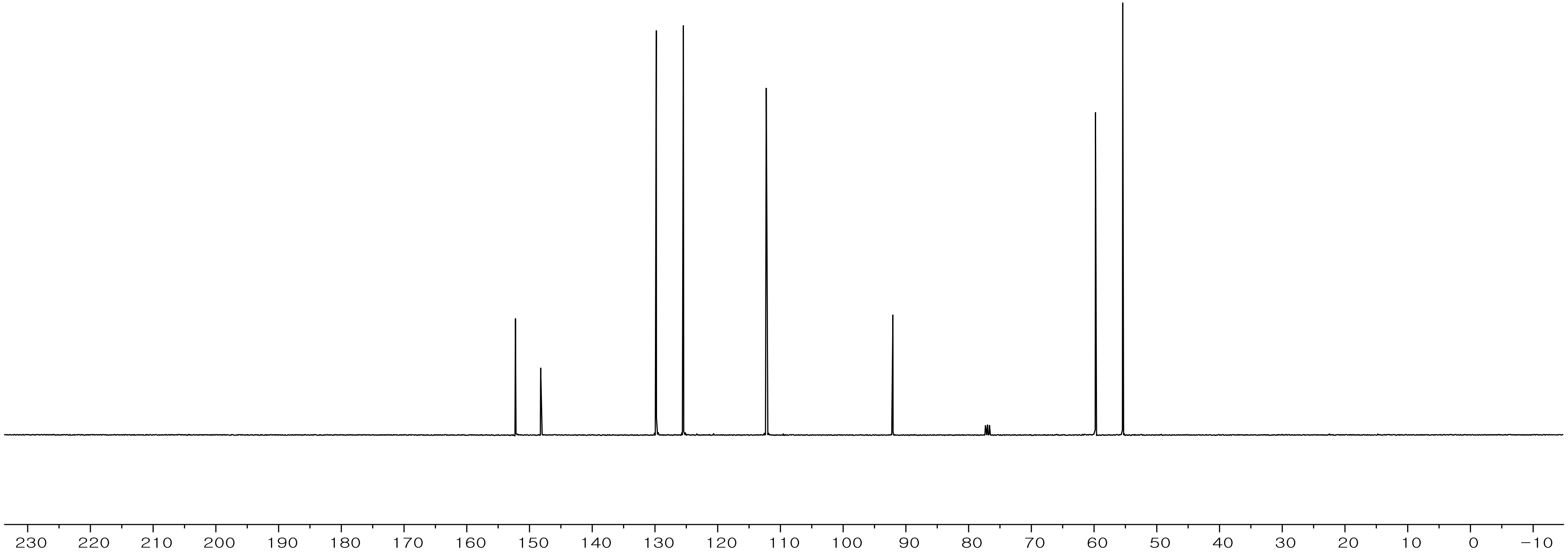

^{1}H NMR (499 MHz, $CDCl_3$)

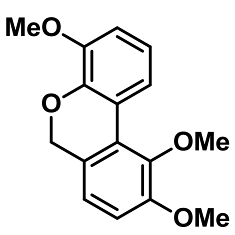

3

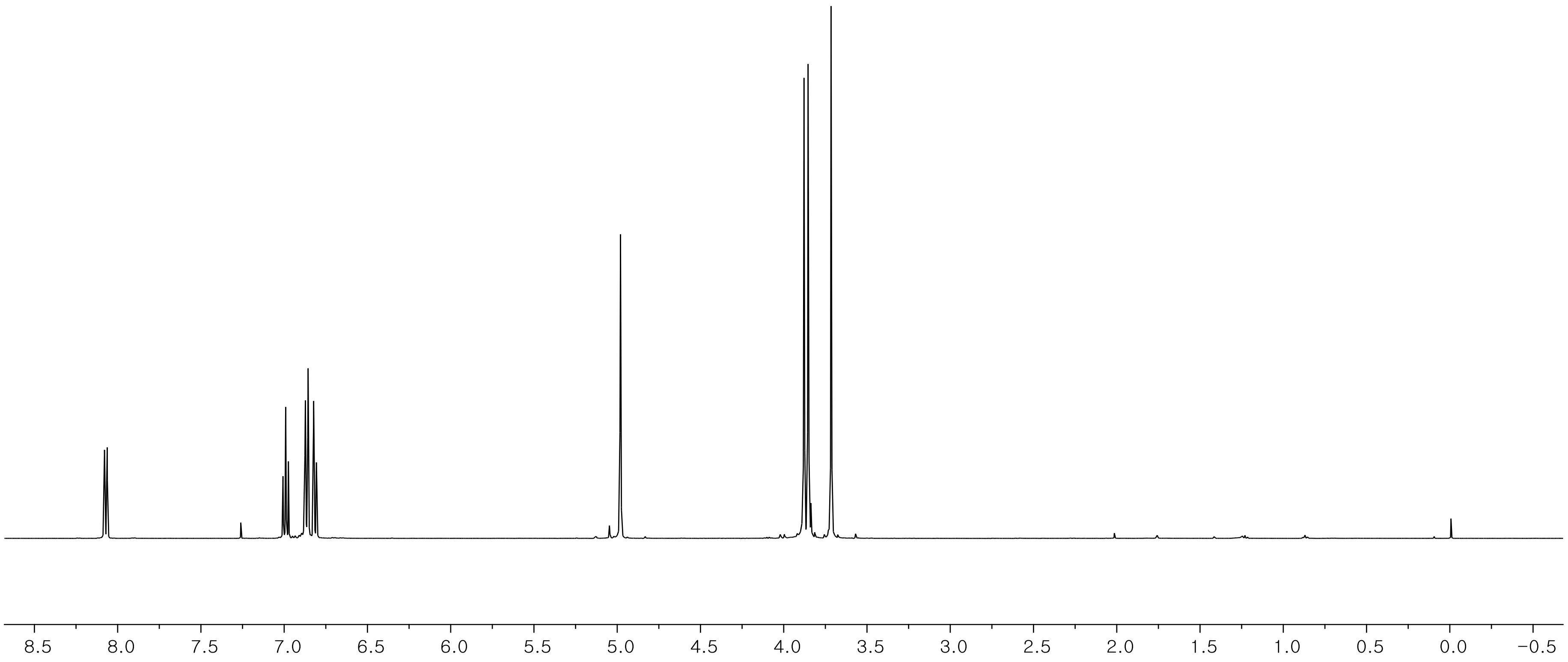

^{13}C NMR (126 MHz, $CDCl_3$)

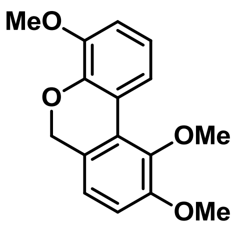

3

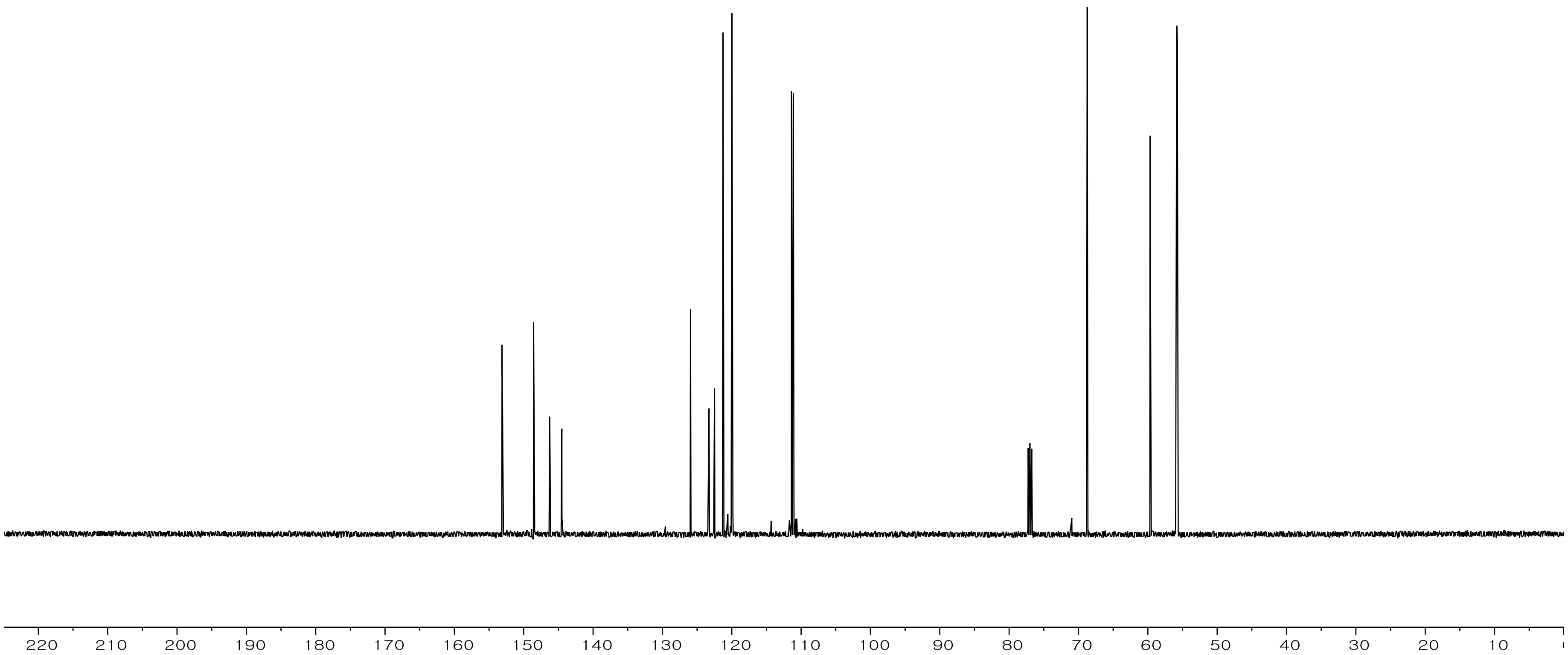

^{1}H NMR (499 MHz, $CDCl_3$)

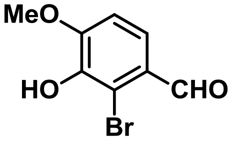

2a

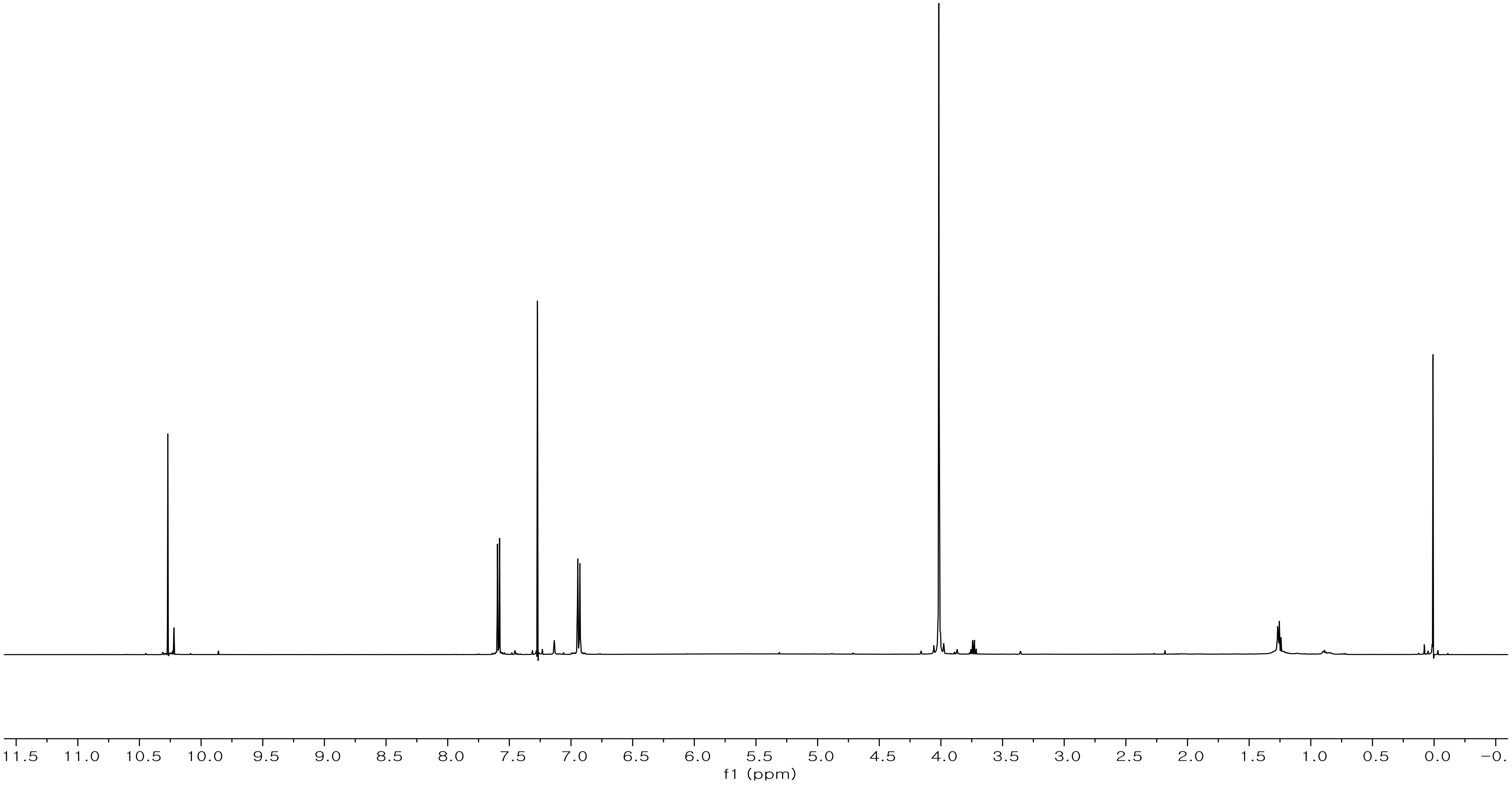

^{13}C NMR (101 MHz, DMSO)

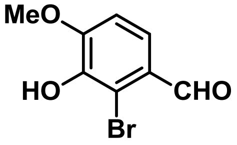

2a

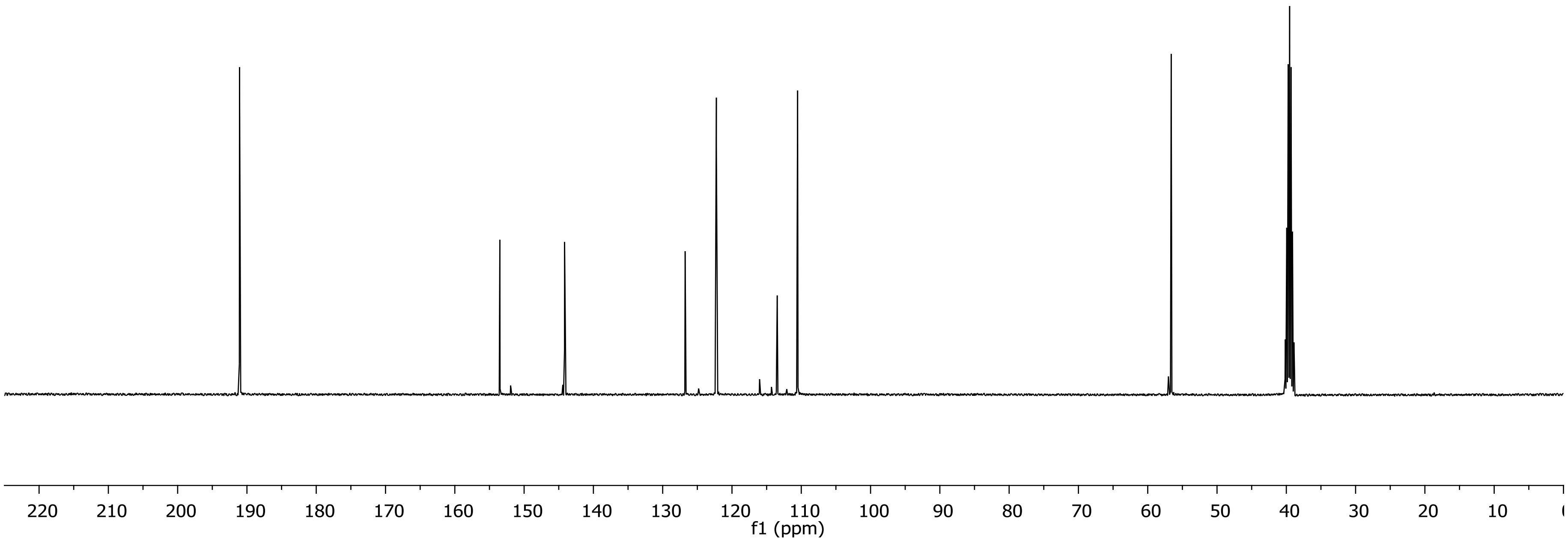

^{1}H NMR (499 MHz, $CDCl_3$)

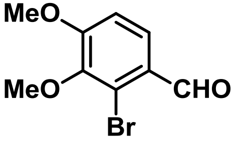

2b

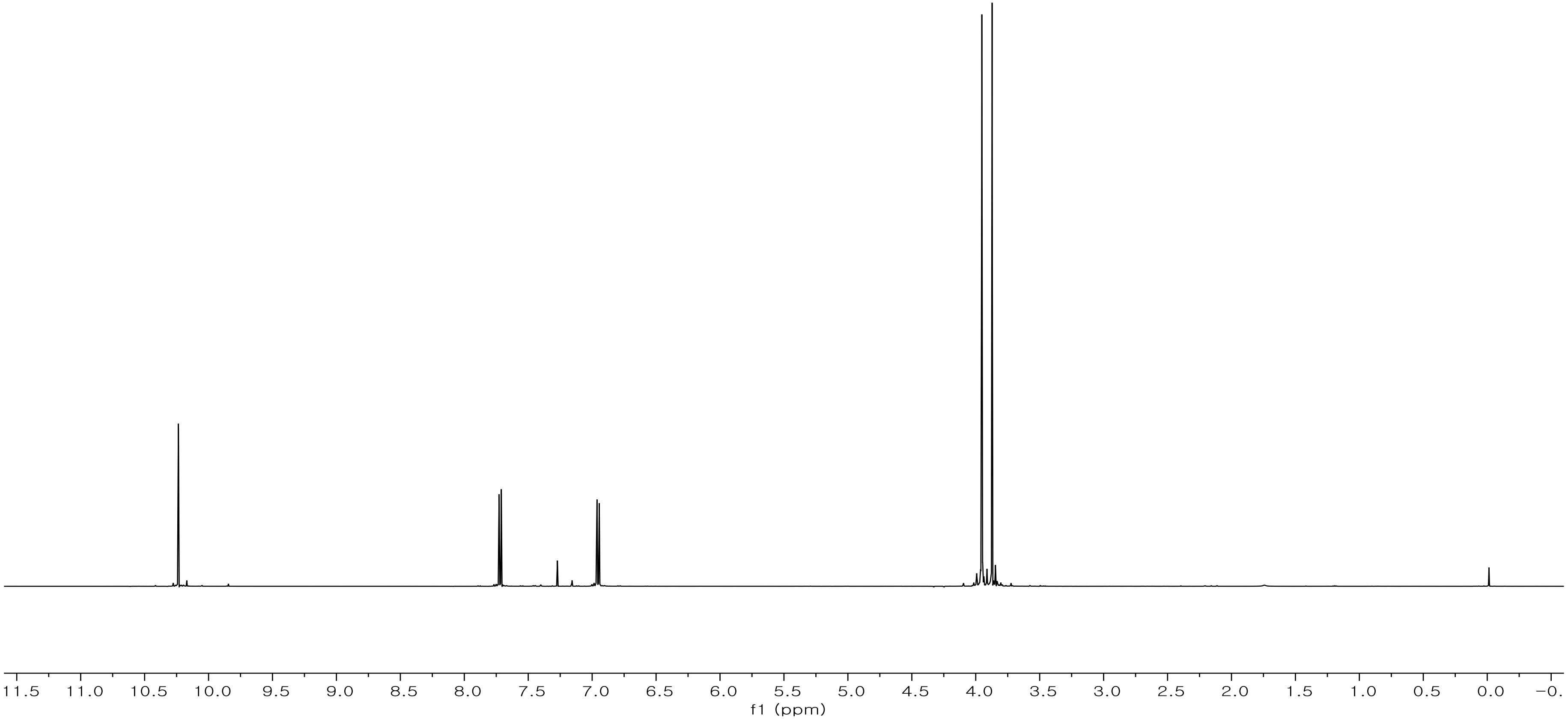

^{13}C NMR (126 MHz, $CDCl_3$)

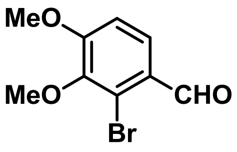

2b

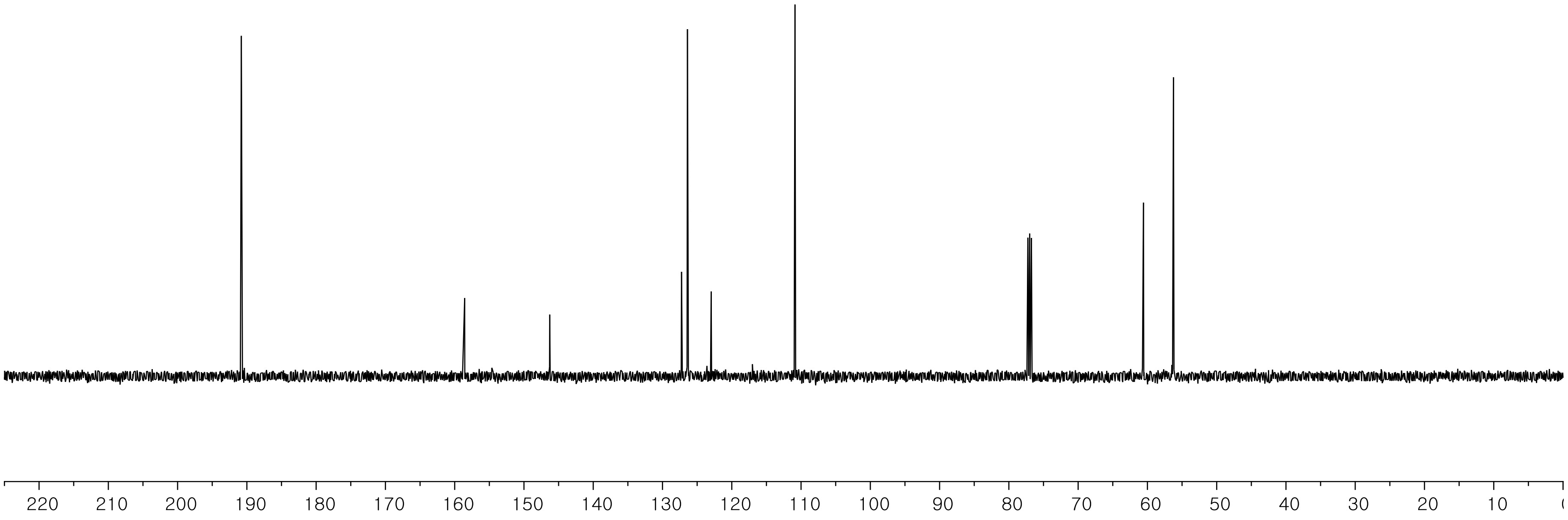

^{1}H NMR (499 MHz, $CDCl_3$)

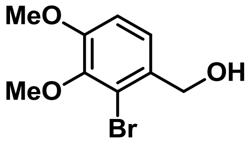

2c

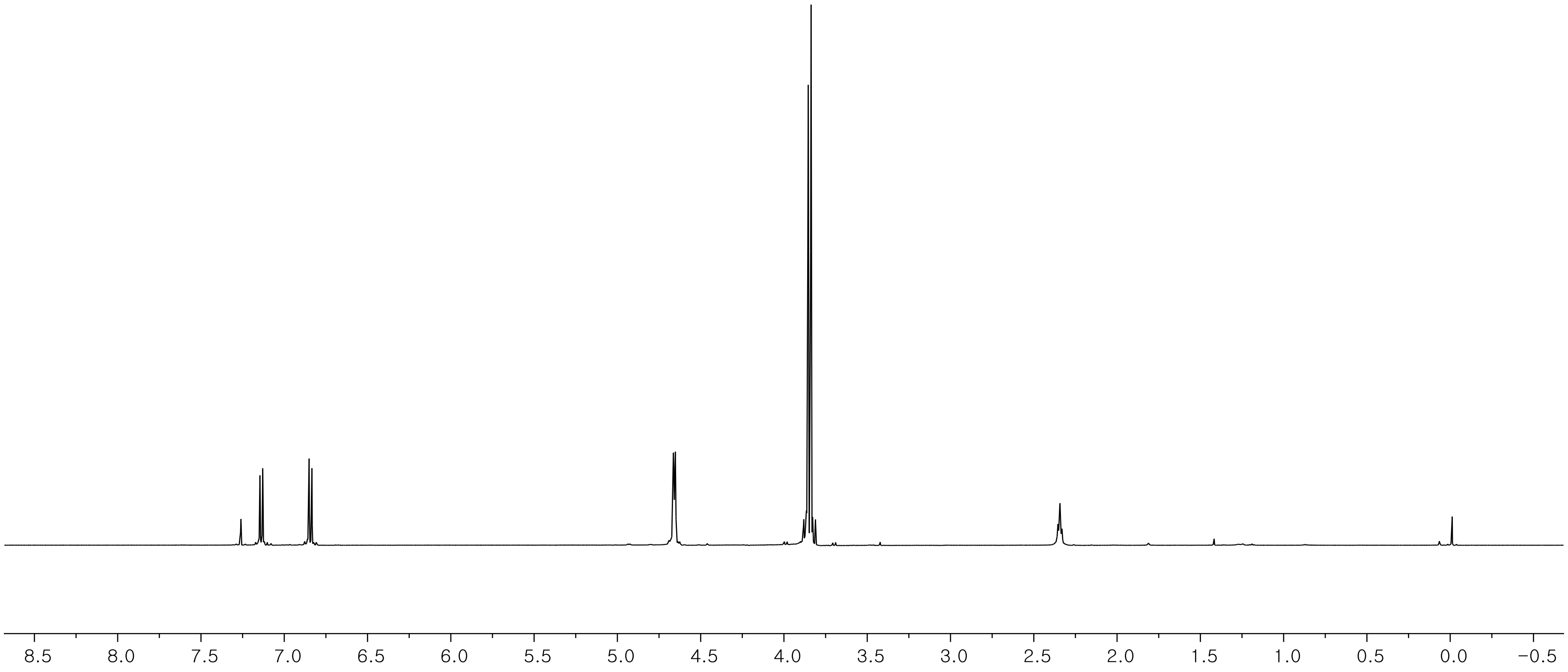

^{13}C NMR (126 MHz, $CDCl_3$)

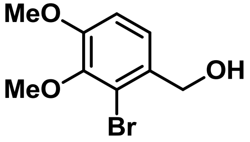

2c

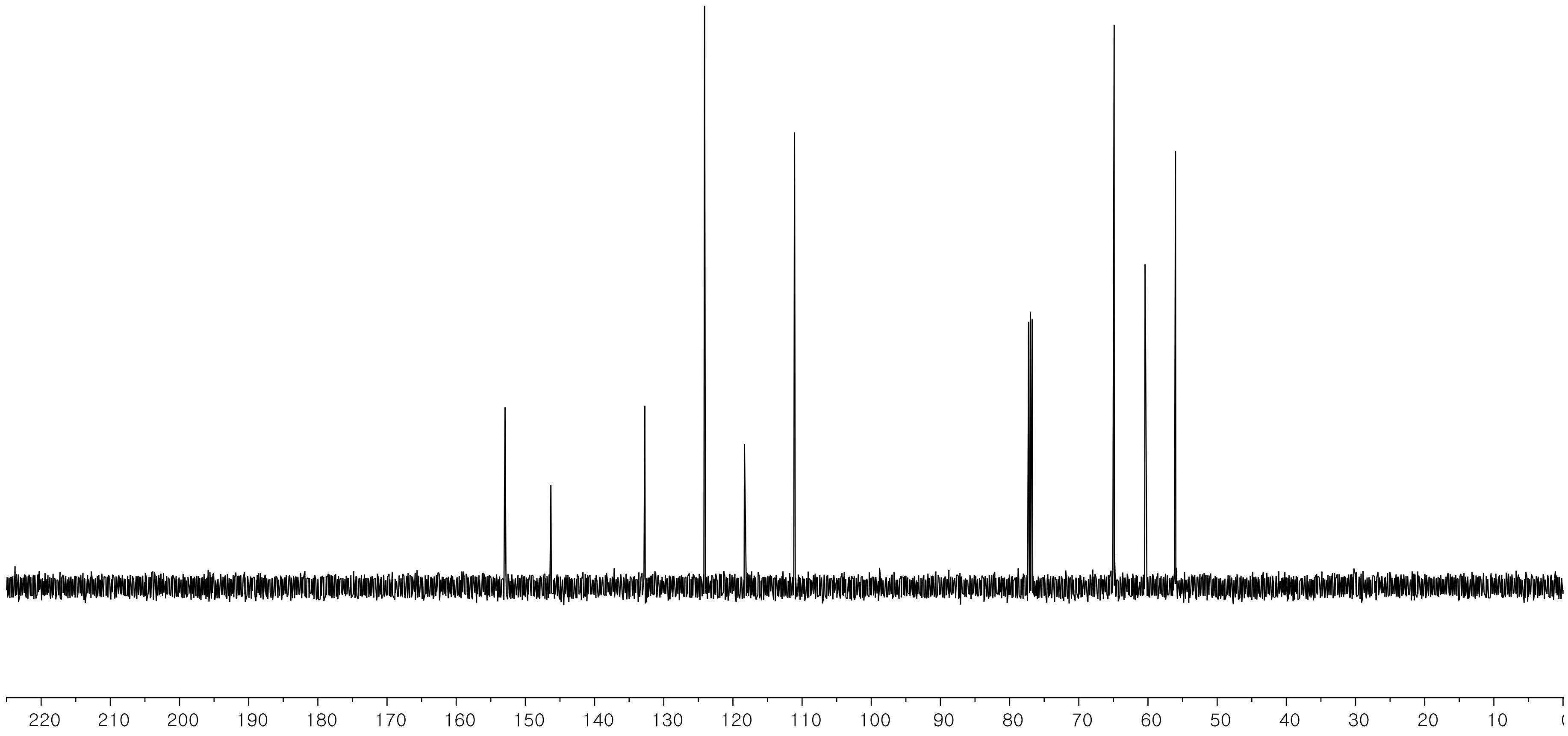

^{1}H NMR (499 MHz, $CDCl_3$)

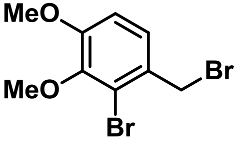

2d

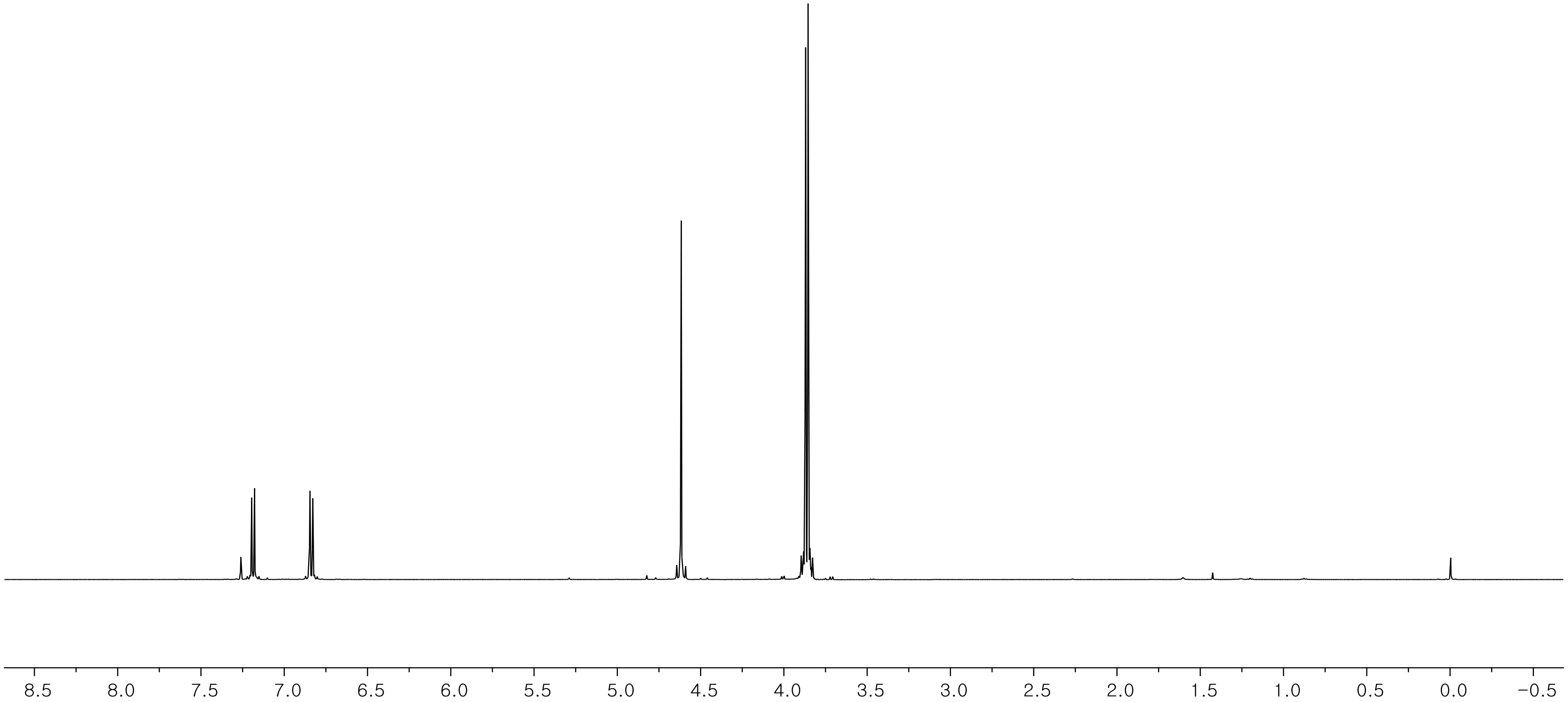

^{13}C NMR (126 MHz, $CDCl_3$)

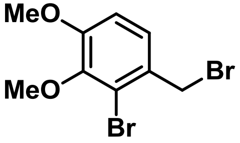

2d

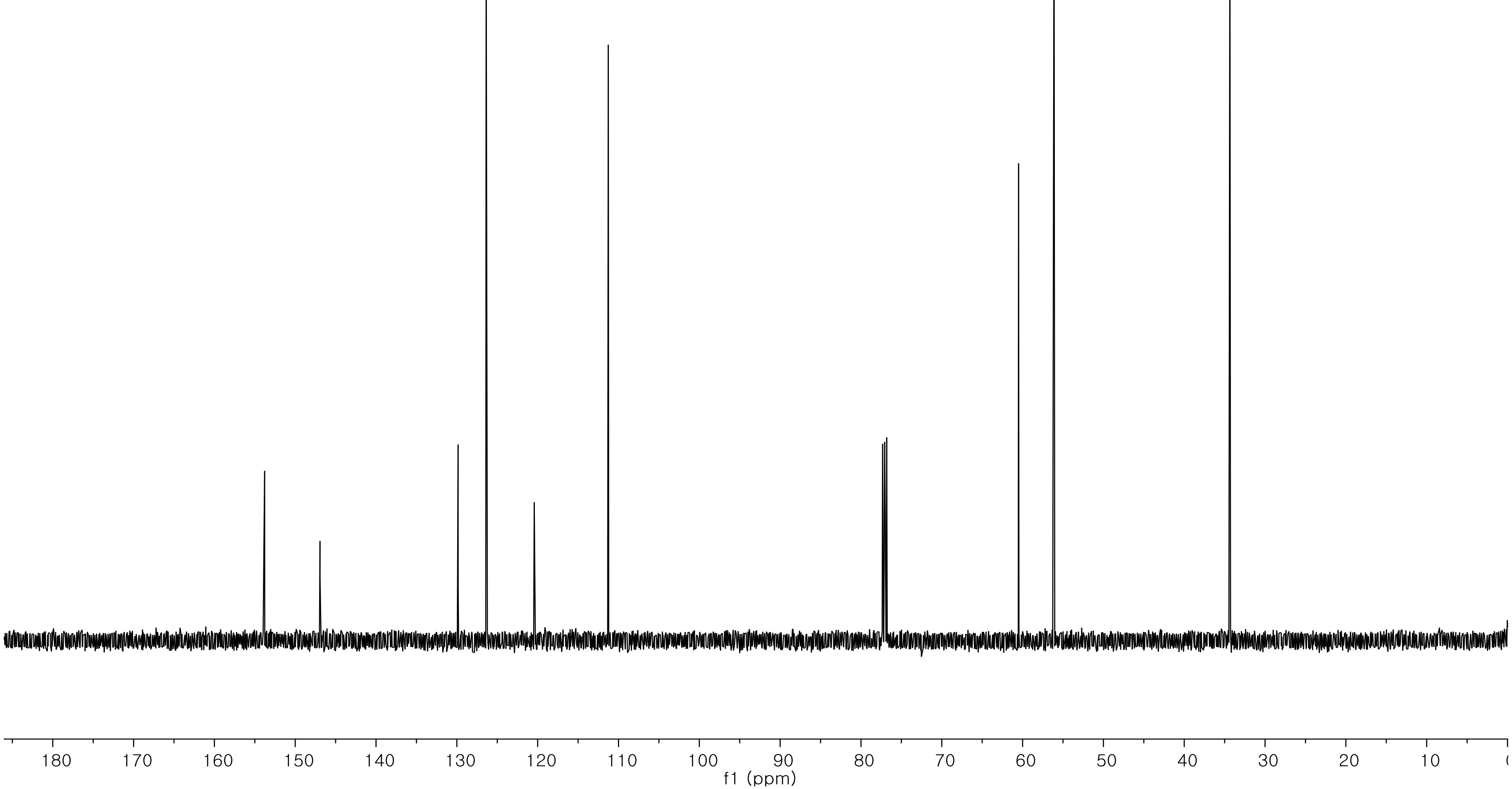

^{1}H NMR (499 MHz, $CDCl_3$)

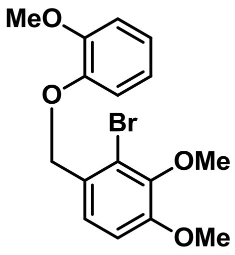

2e

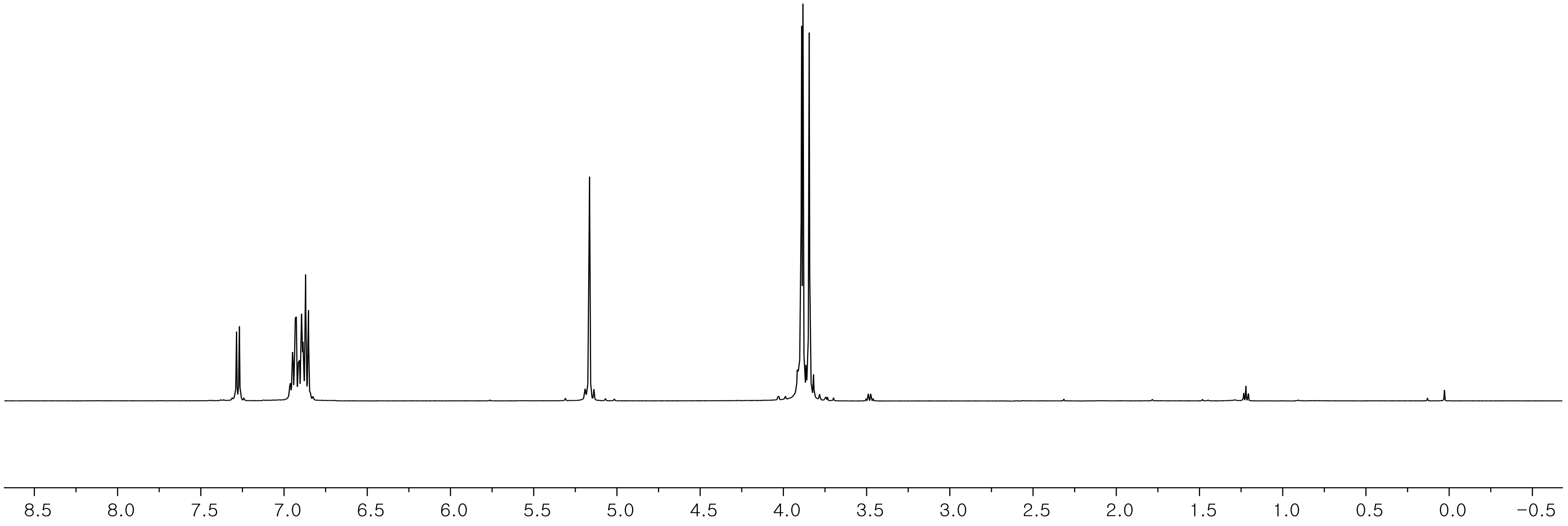

^{13}C NMR (101 MHz, $CDCl_3$)

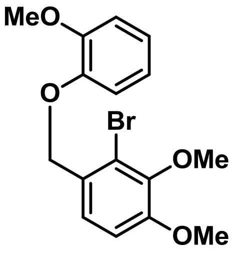

2e

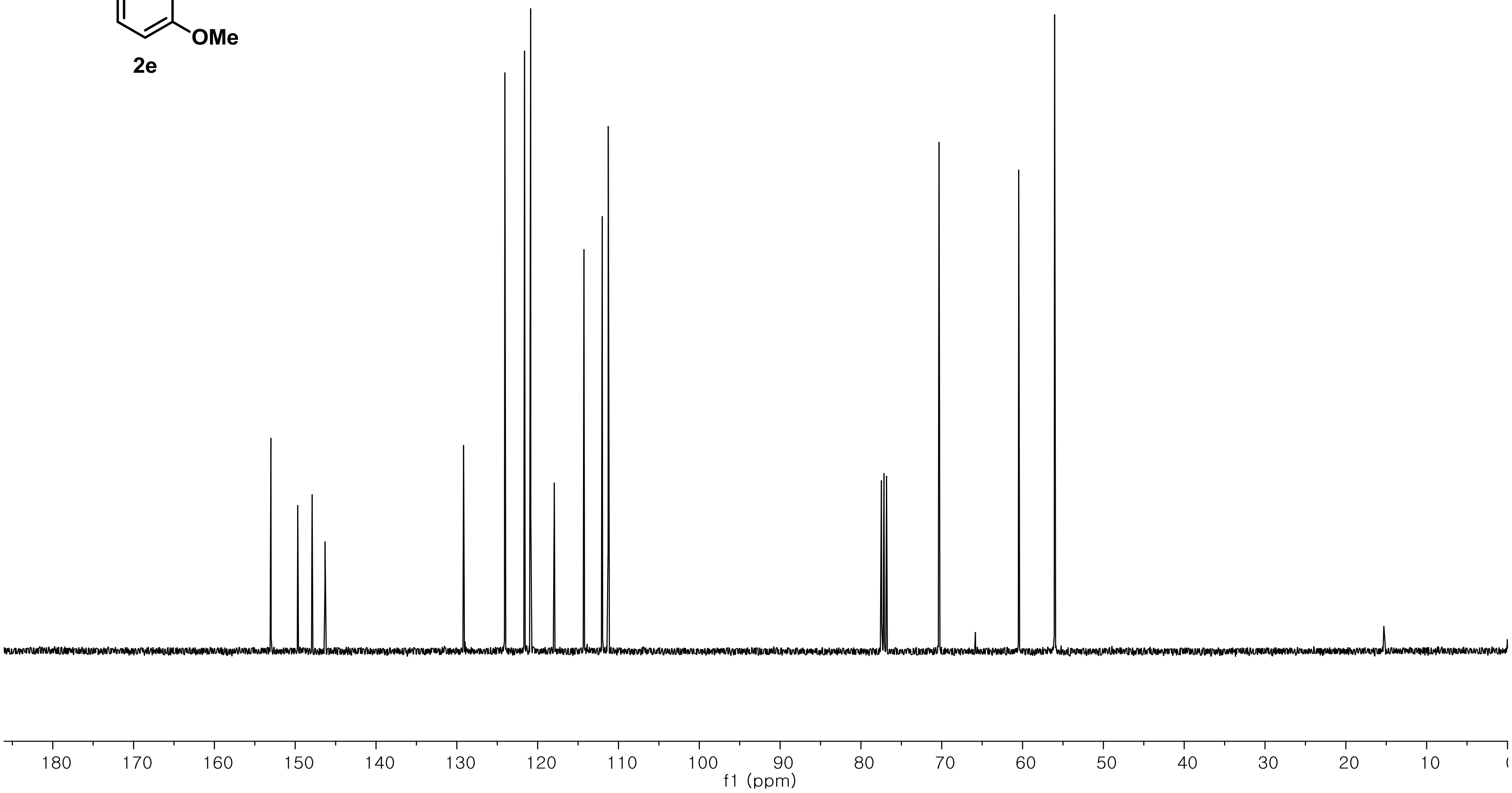

^{1}H NMR (499 MHz, $CDCl_3$)

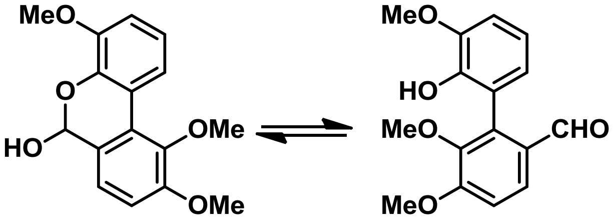

4'   4

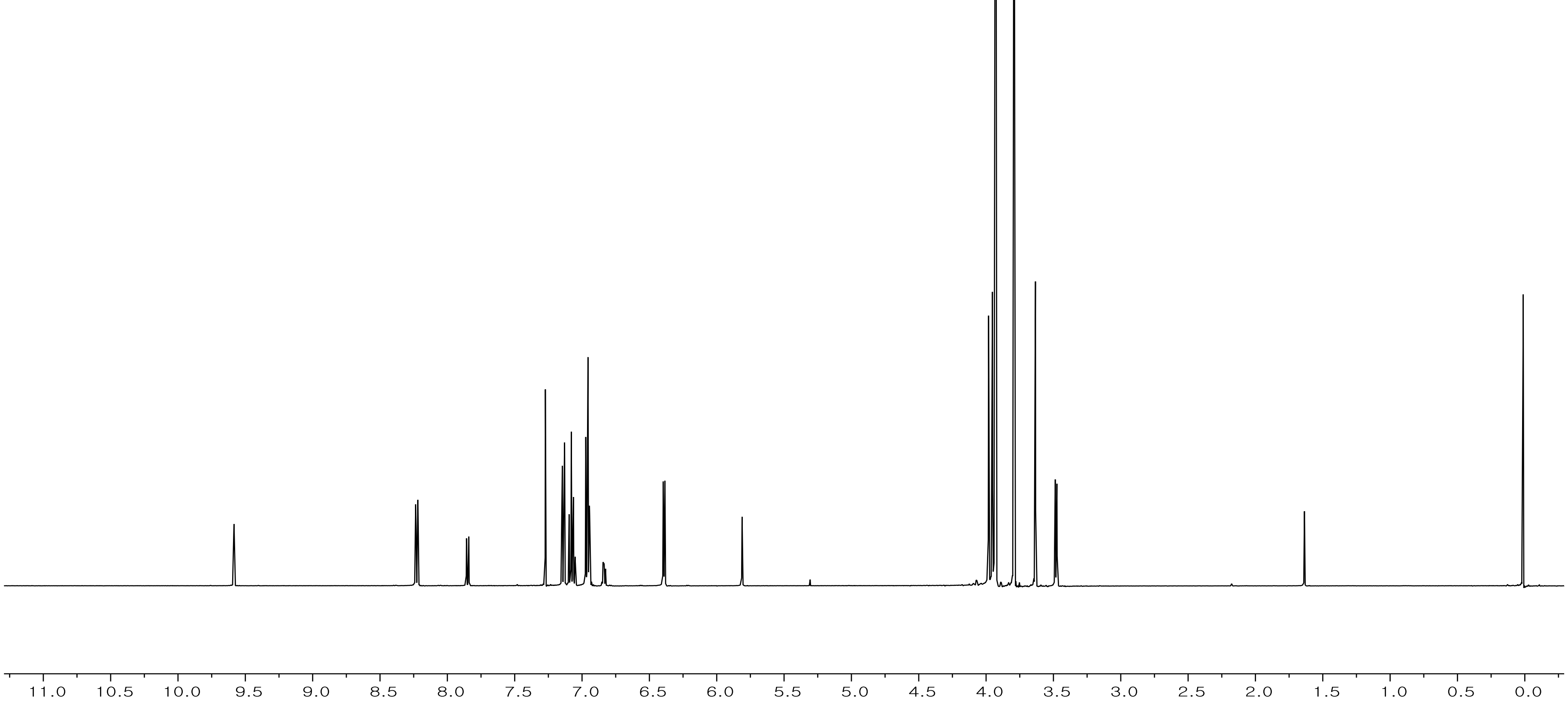

^{13}C NMR (101 MHz, DMSO)

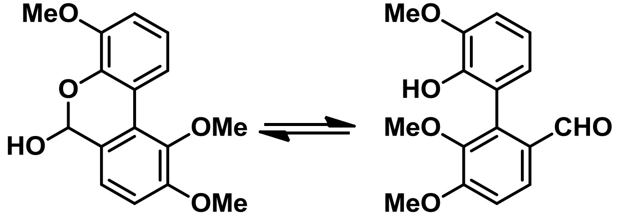

4'   4

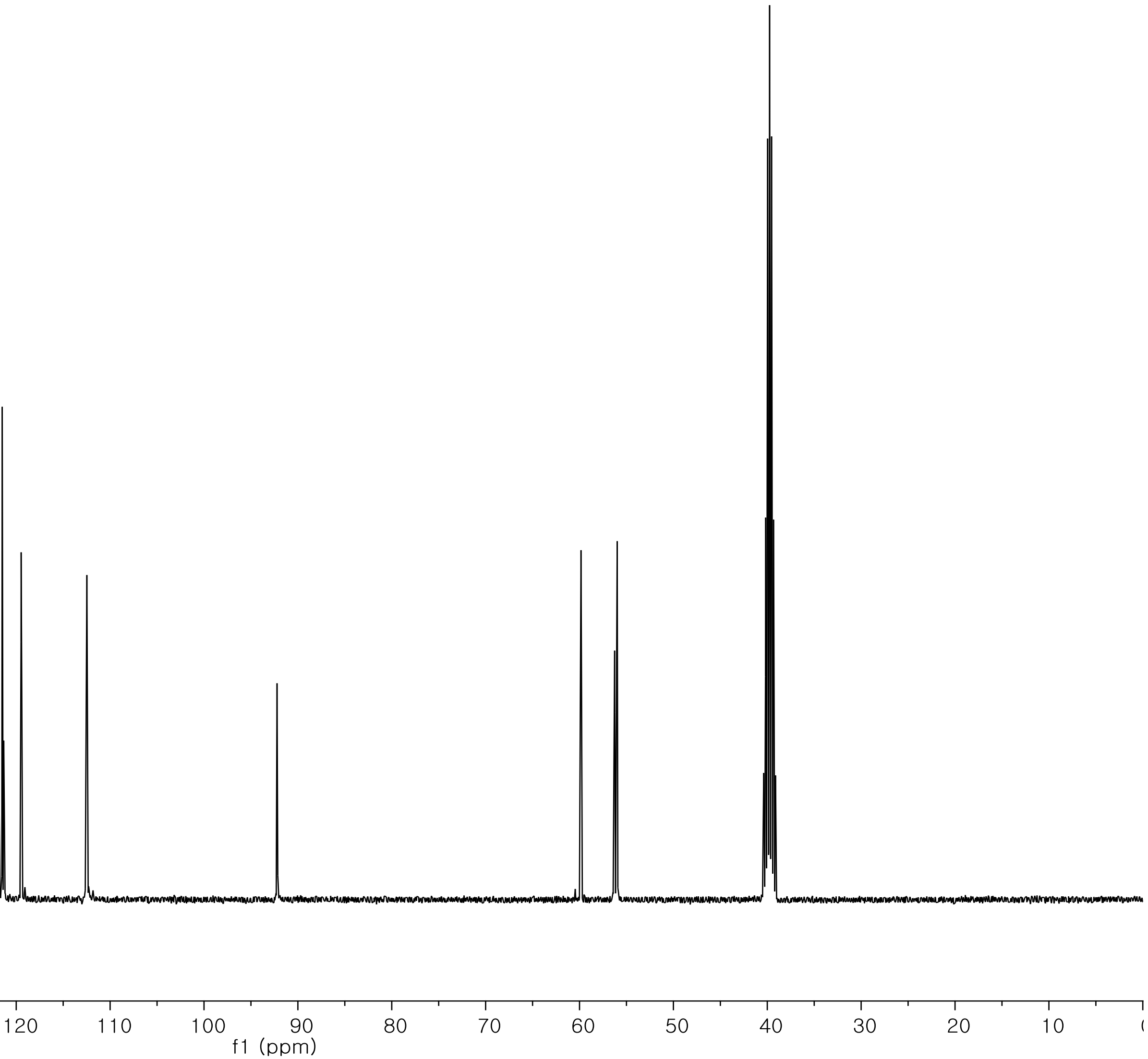

^{1}H NMR (400 MHz, C_6D_6)

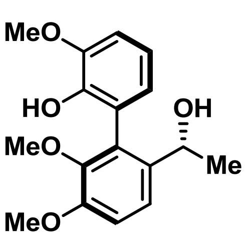

5a

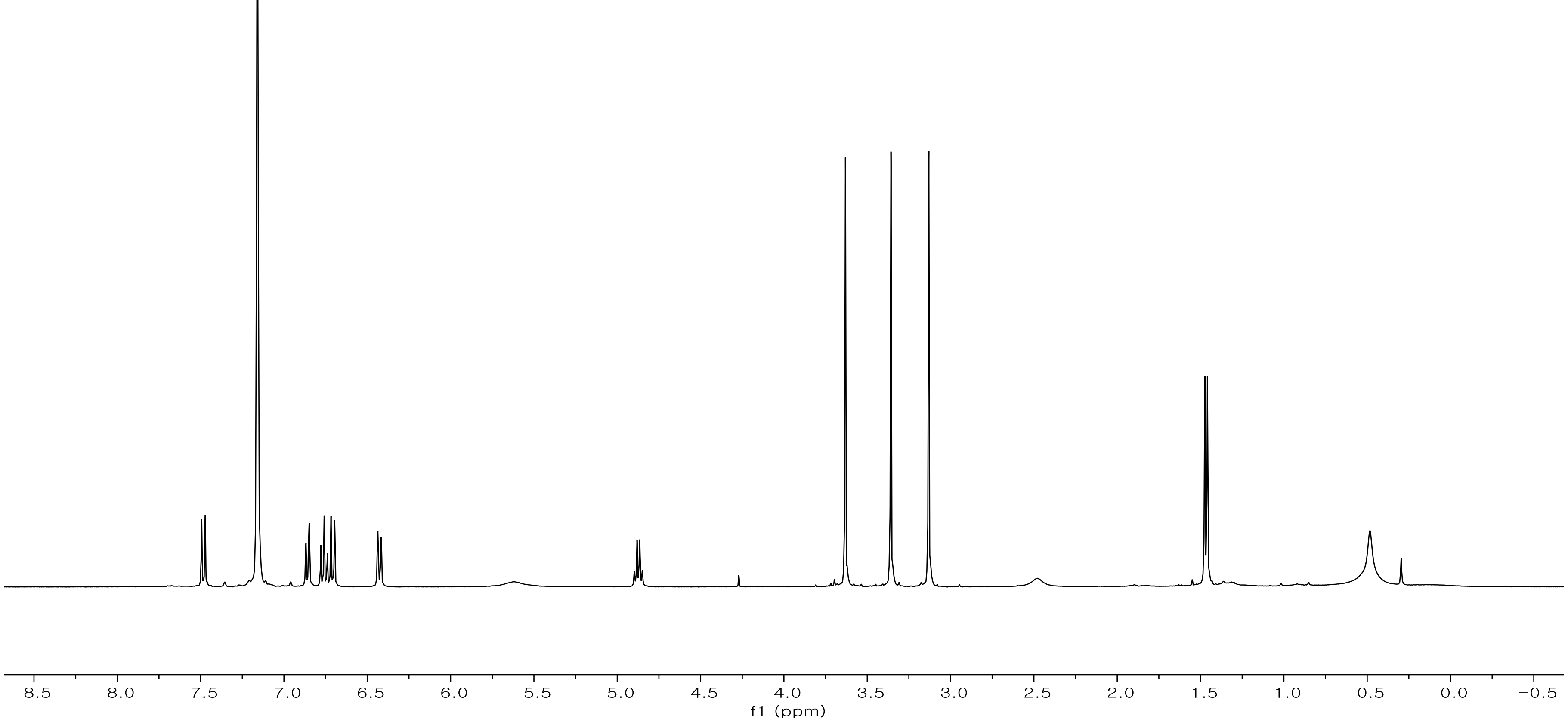

^{13}C NMR (101 MHz, $CDCl_3$)

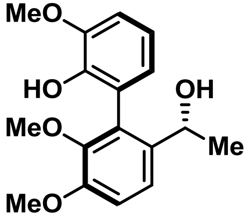

5a

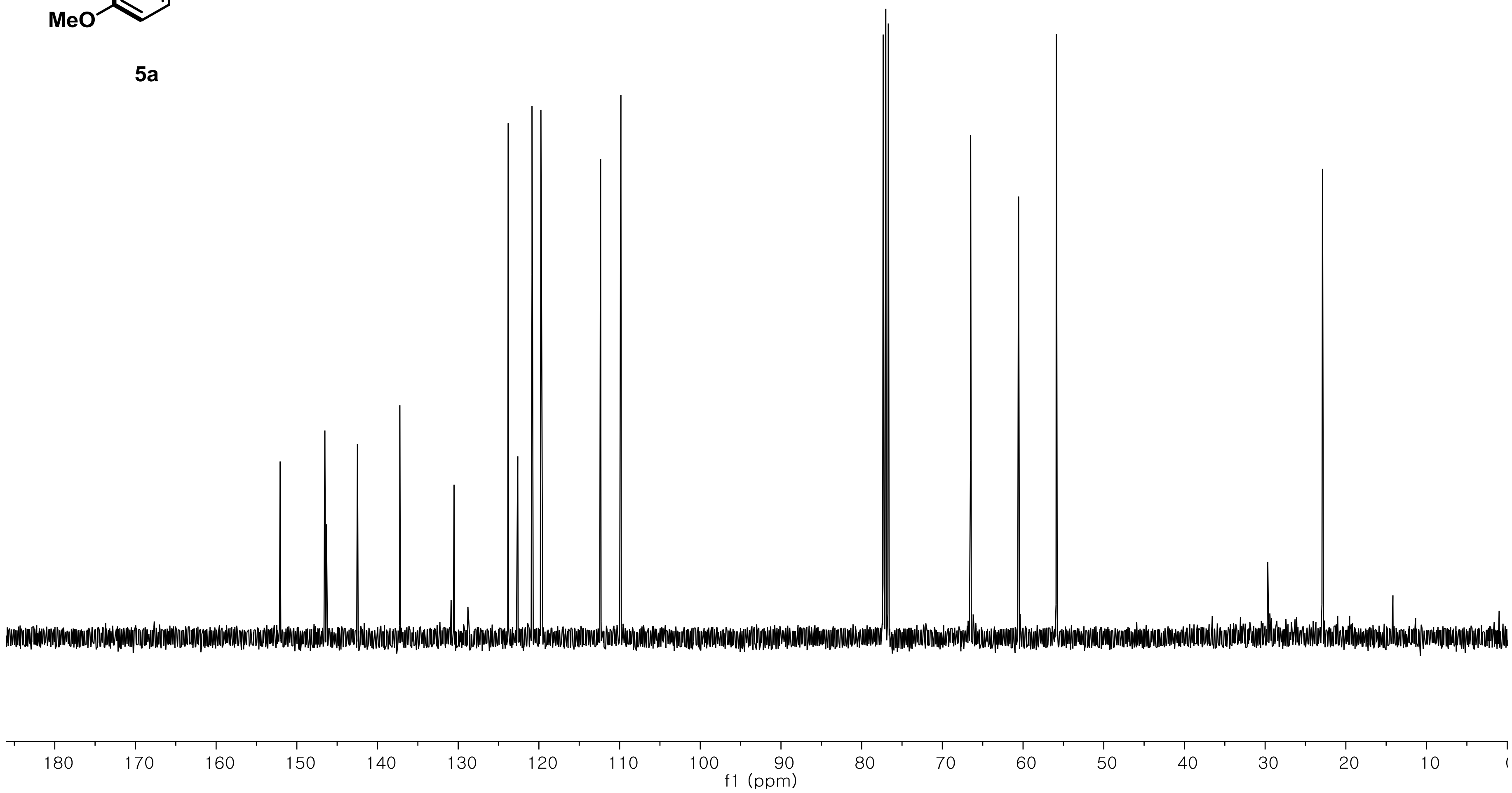

^1H NMR (400 MHz, C_6D_6)

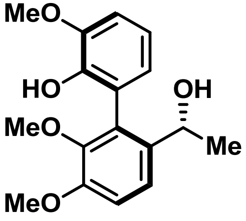

5a'

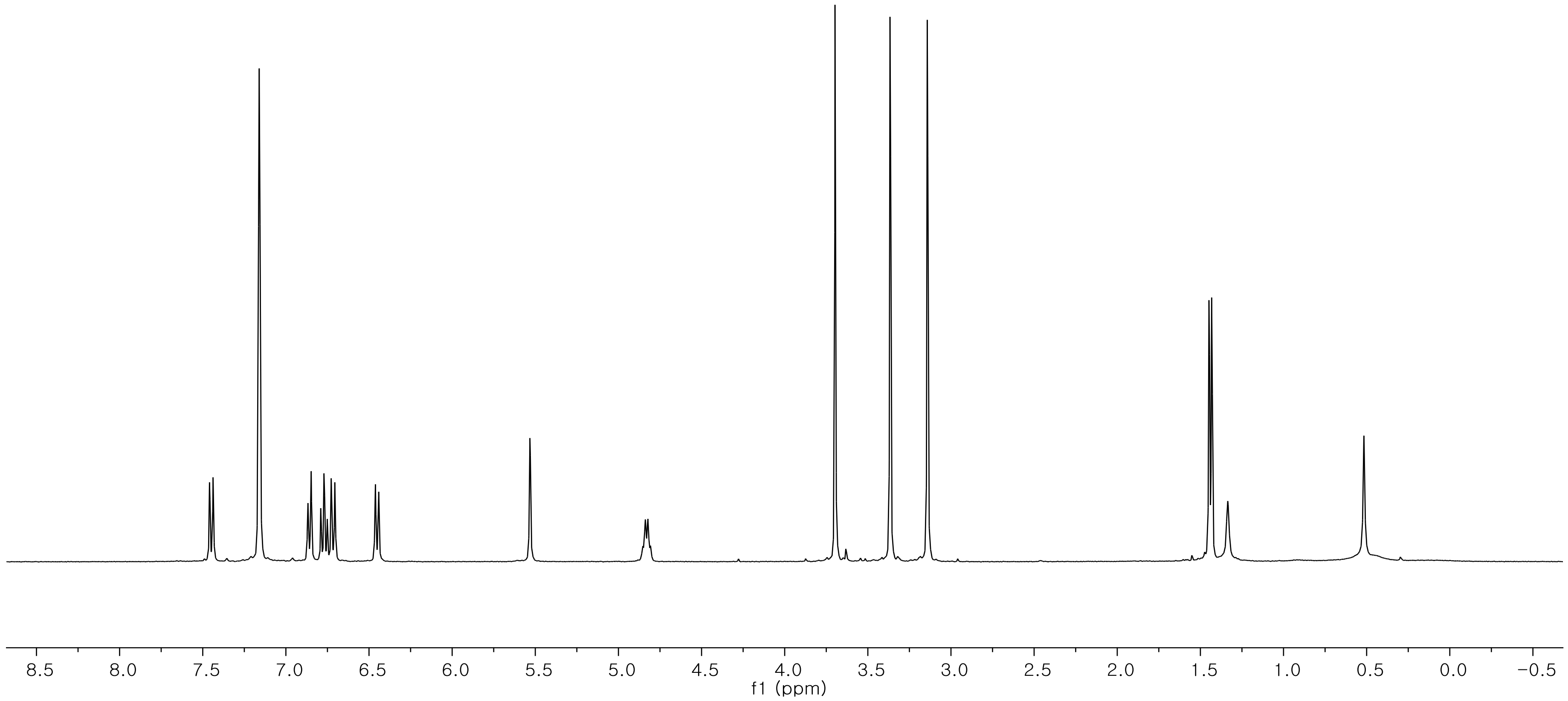

^{13}C NMR (101 MHz, $CDCl_3$)

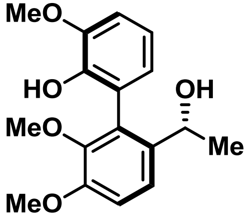

5a'

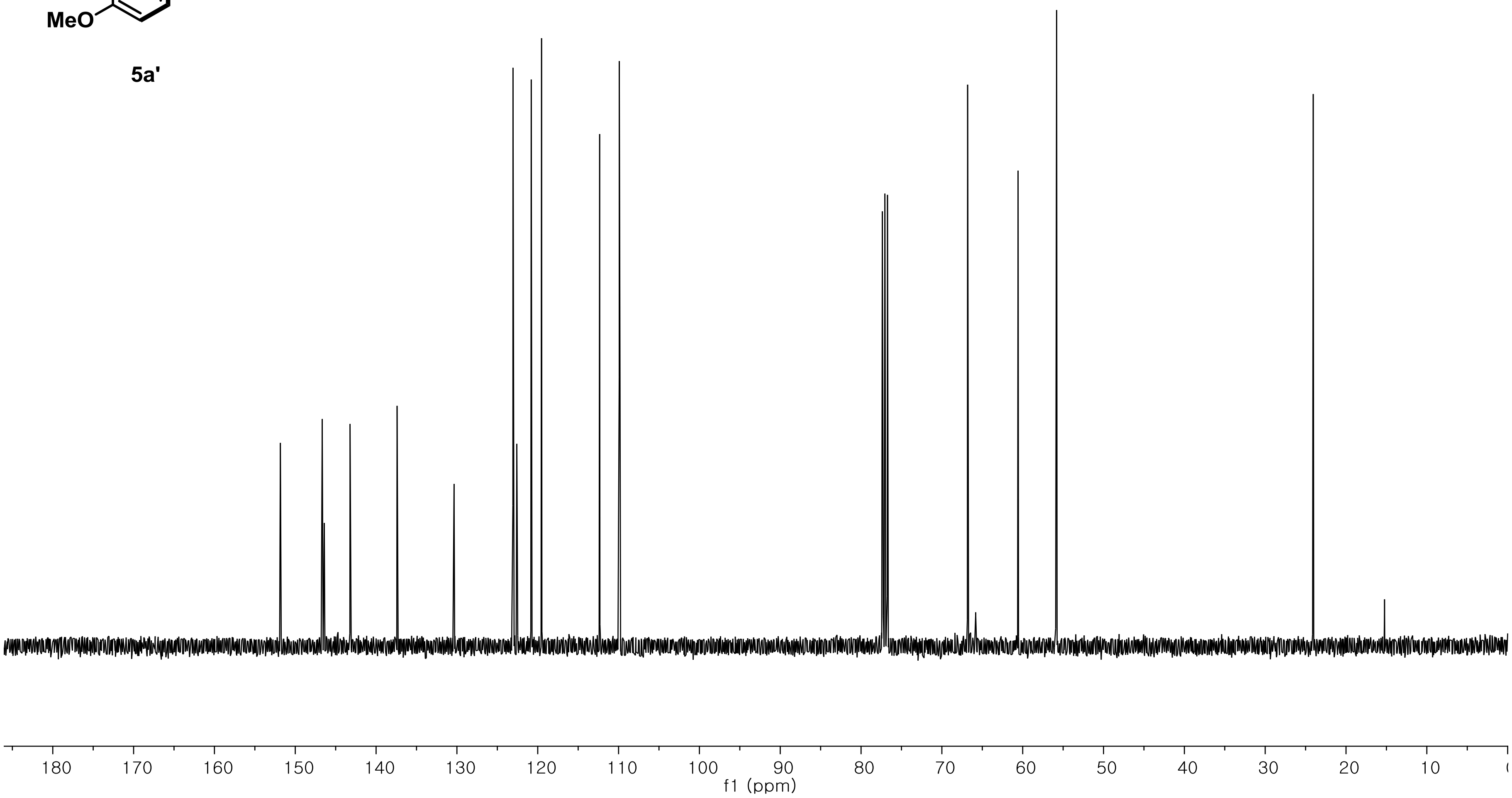

^{1}H NMR (400 MHz, C_6D_6)

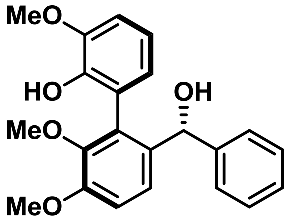

5b

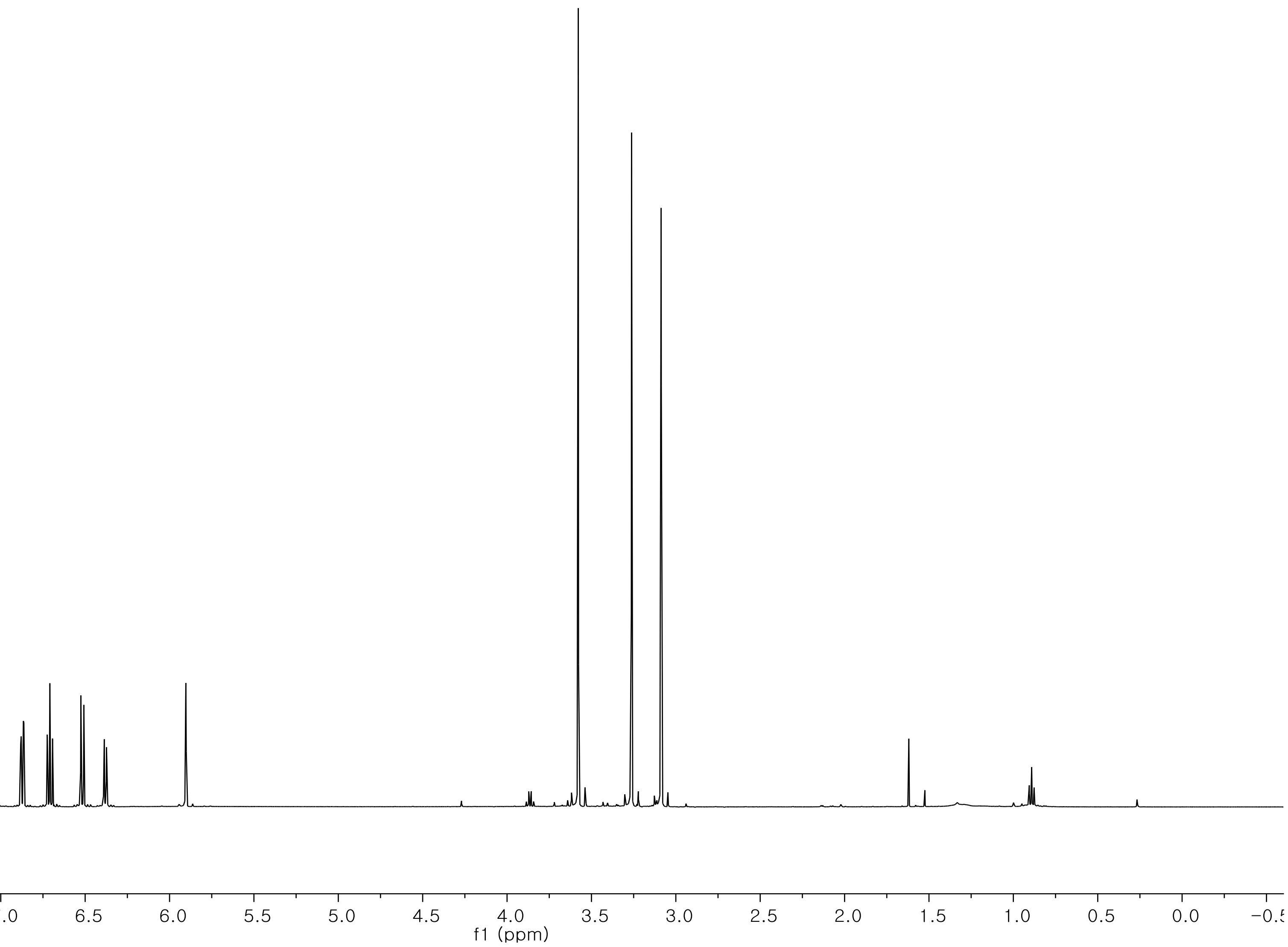

^{13}C NMR (126 MHz, C_6D_6)

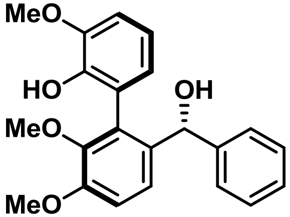

5b

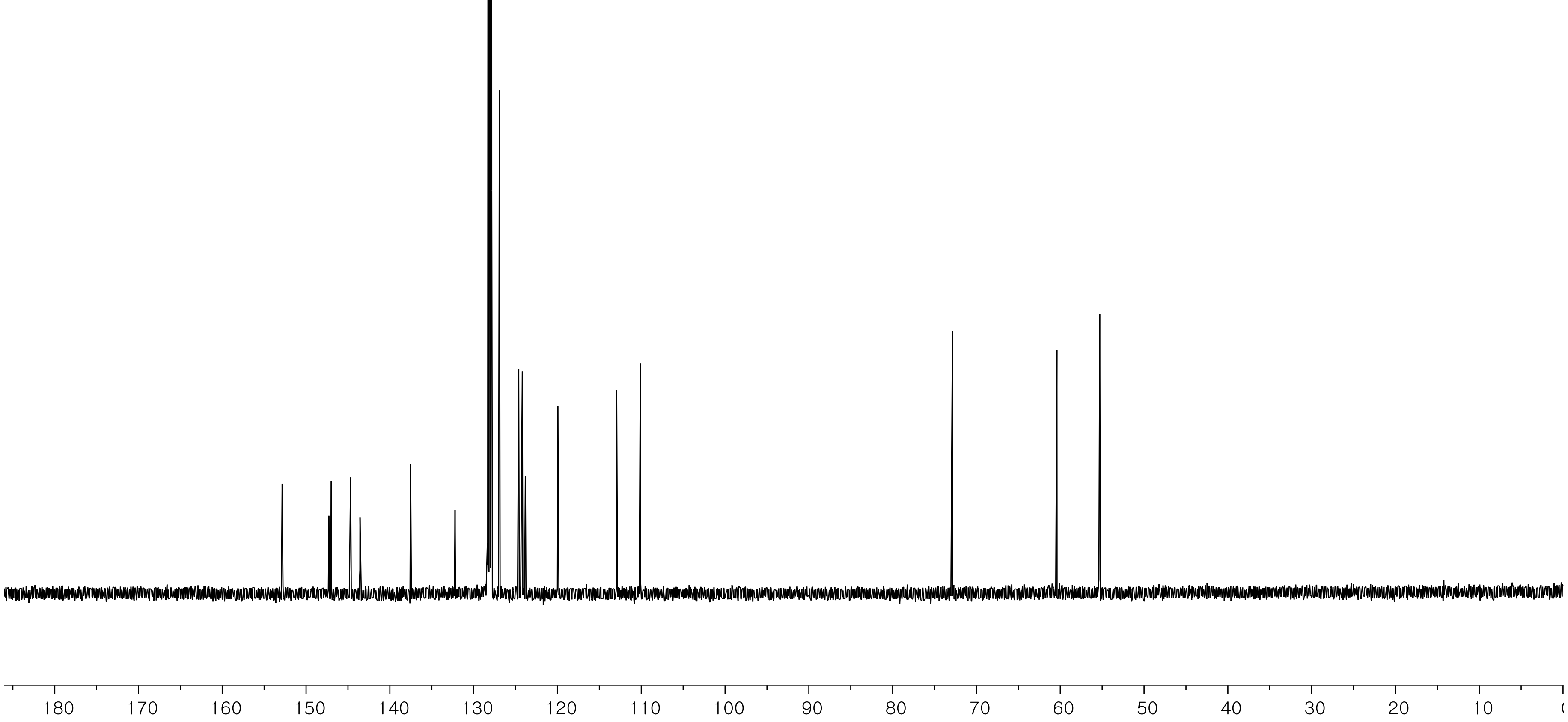

^{1}H NMR (400 MHz, C_6D_6)

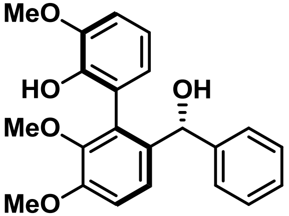

5b'

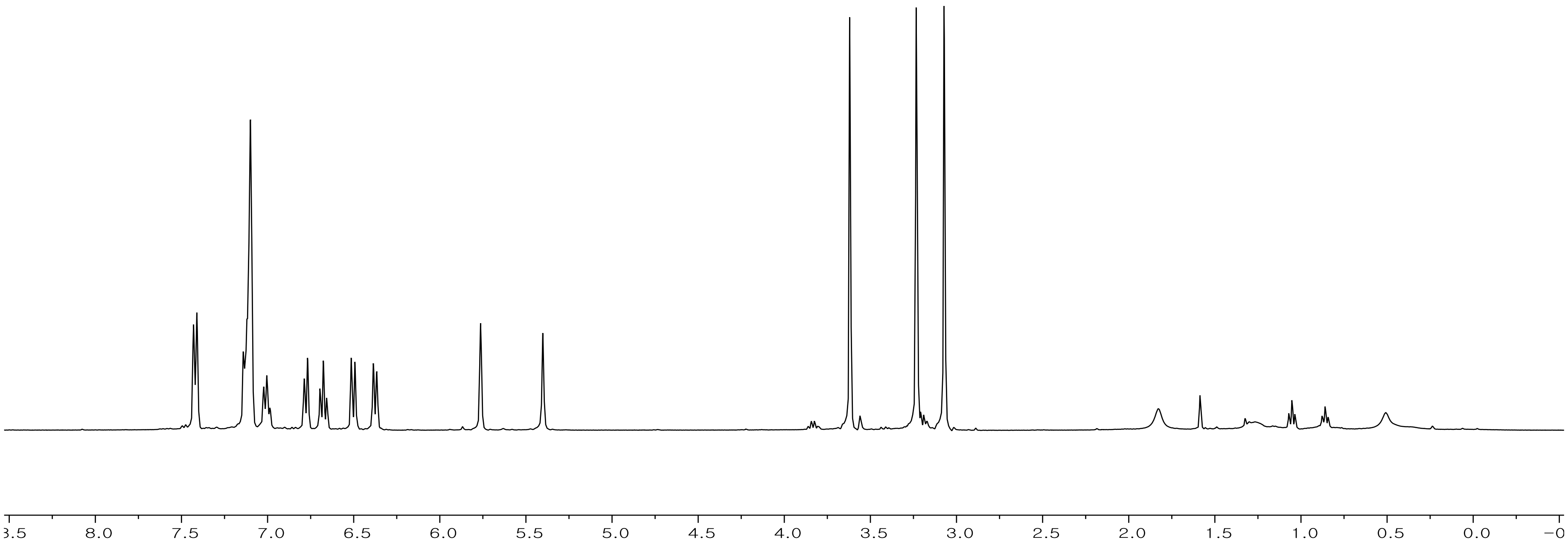

^{13}C NMR (101 MHz, C_6D_6)

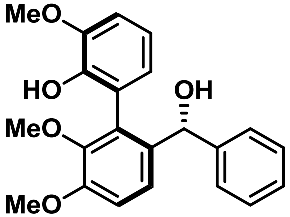

5b'

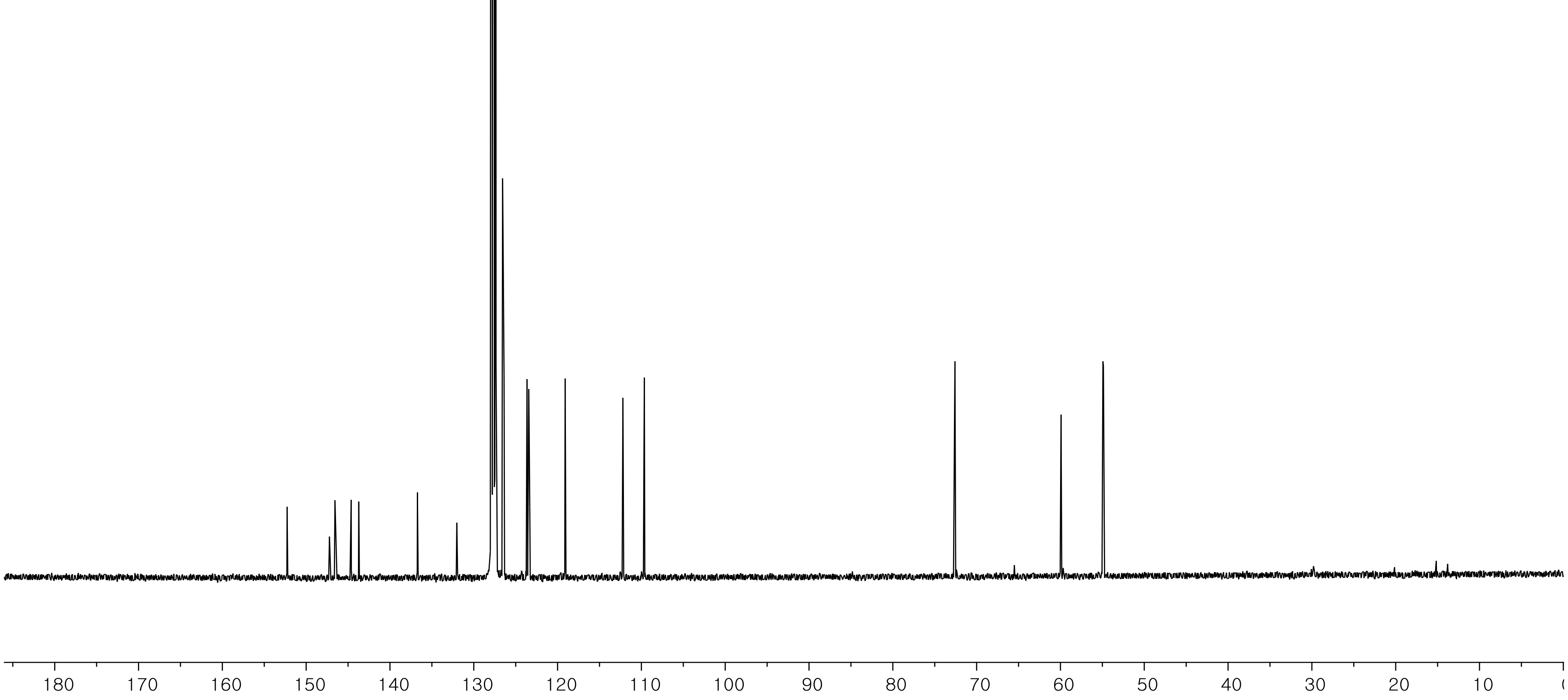

^{1}H NMR (499 MHz, $CDCl_3$)

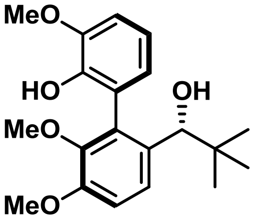

5c

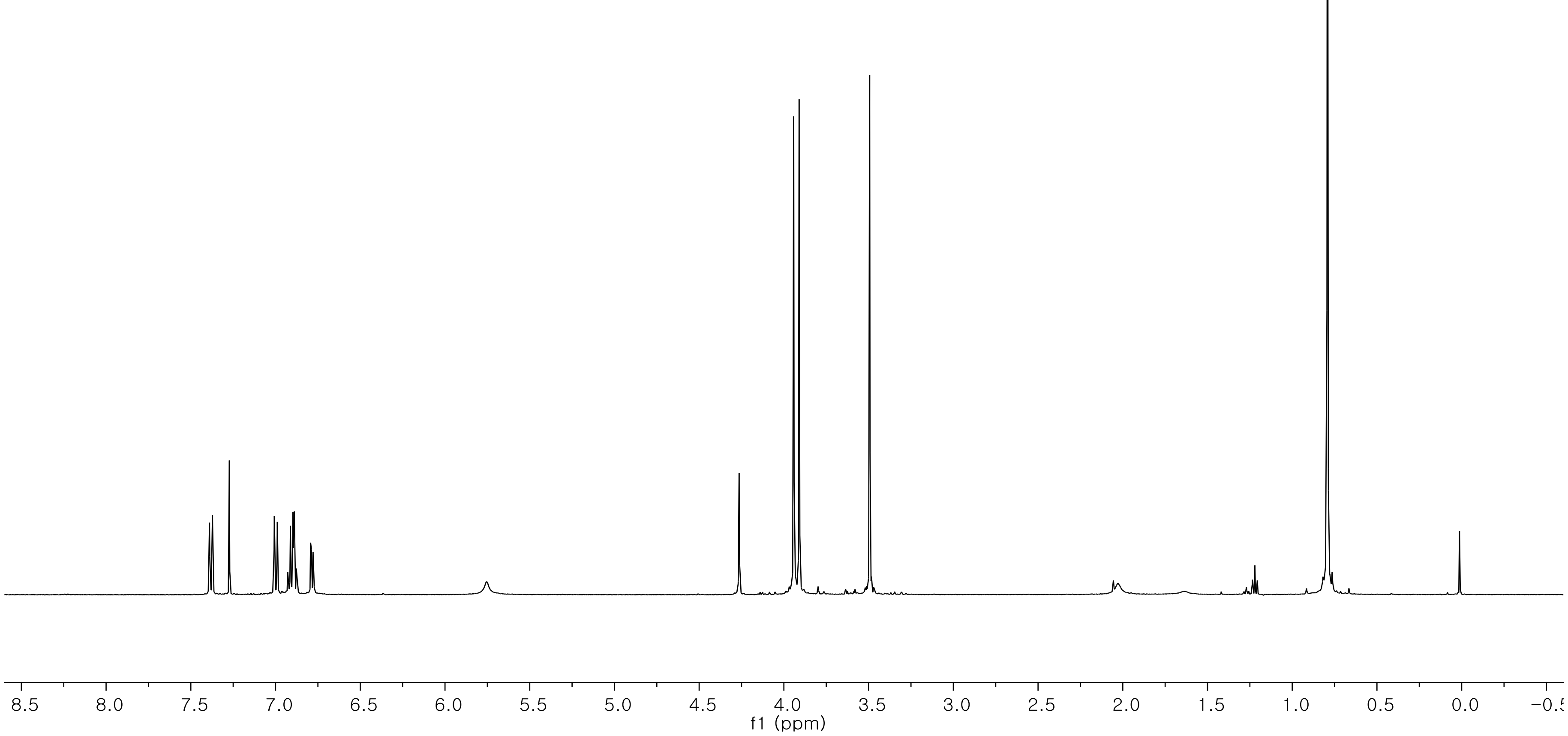

^{13}C NMR (101 MHz, $CDCl_3$)

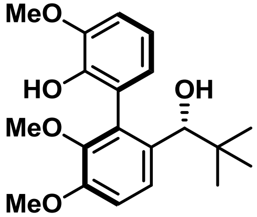

5c

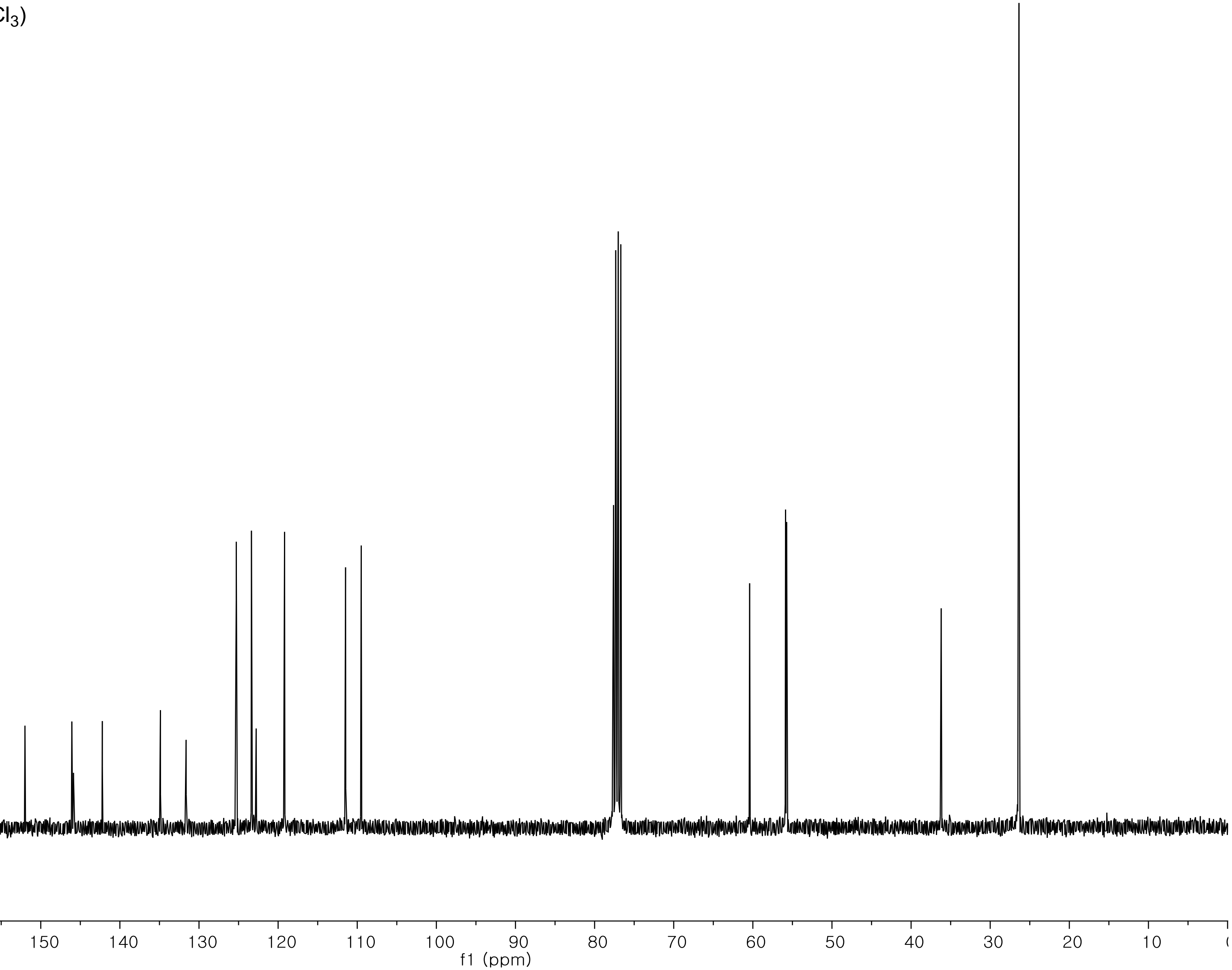

^{1}H NMR (499 MHz, $CDCl_3$)

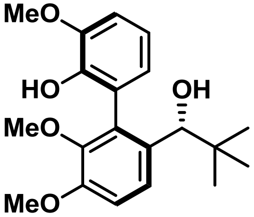

5c'

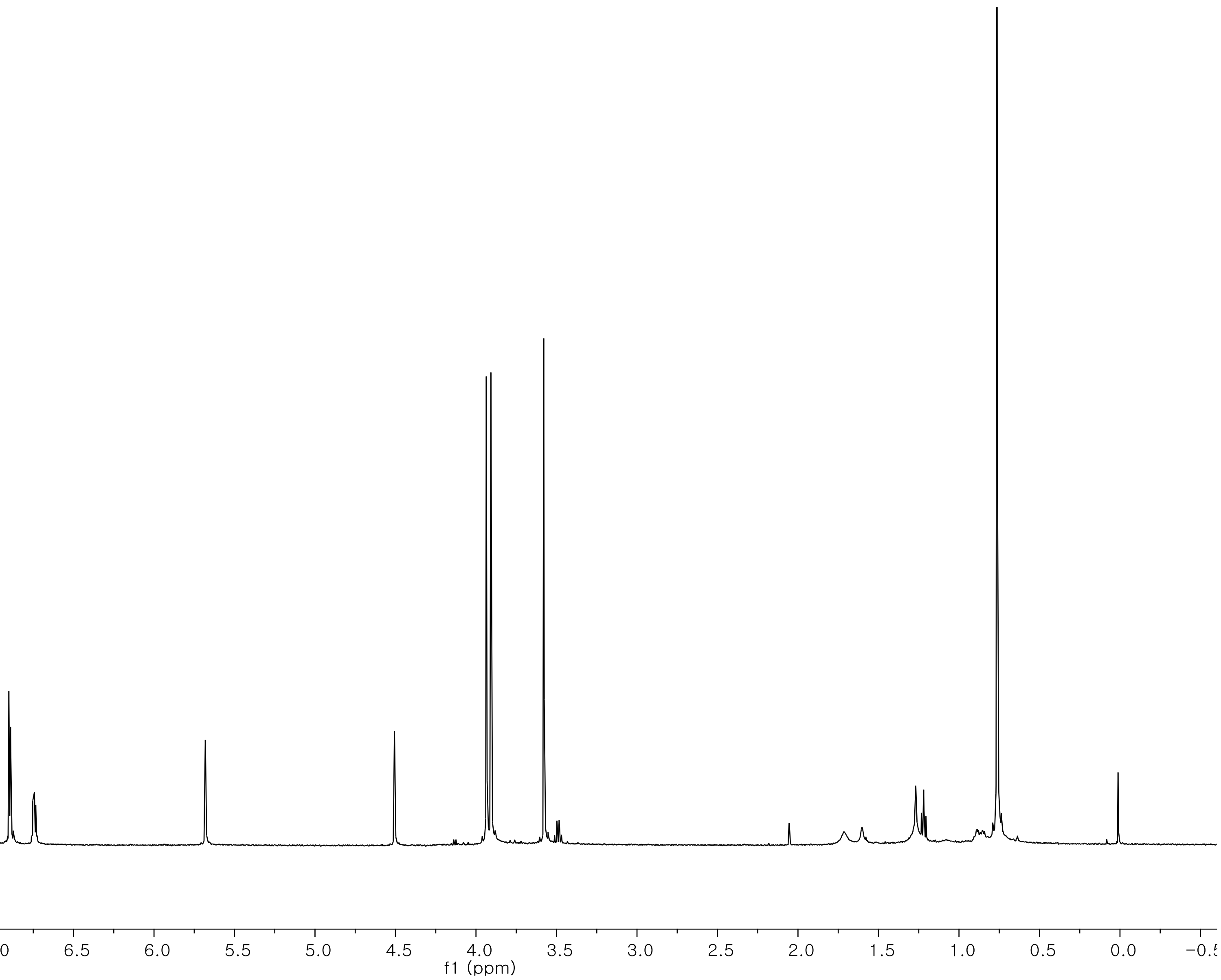

^{13}C NMR (101 MHz, $CDCl_3$)

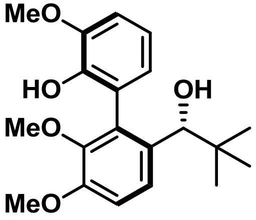

5c'

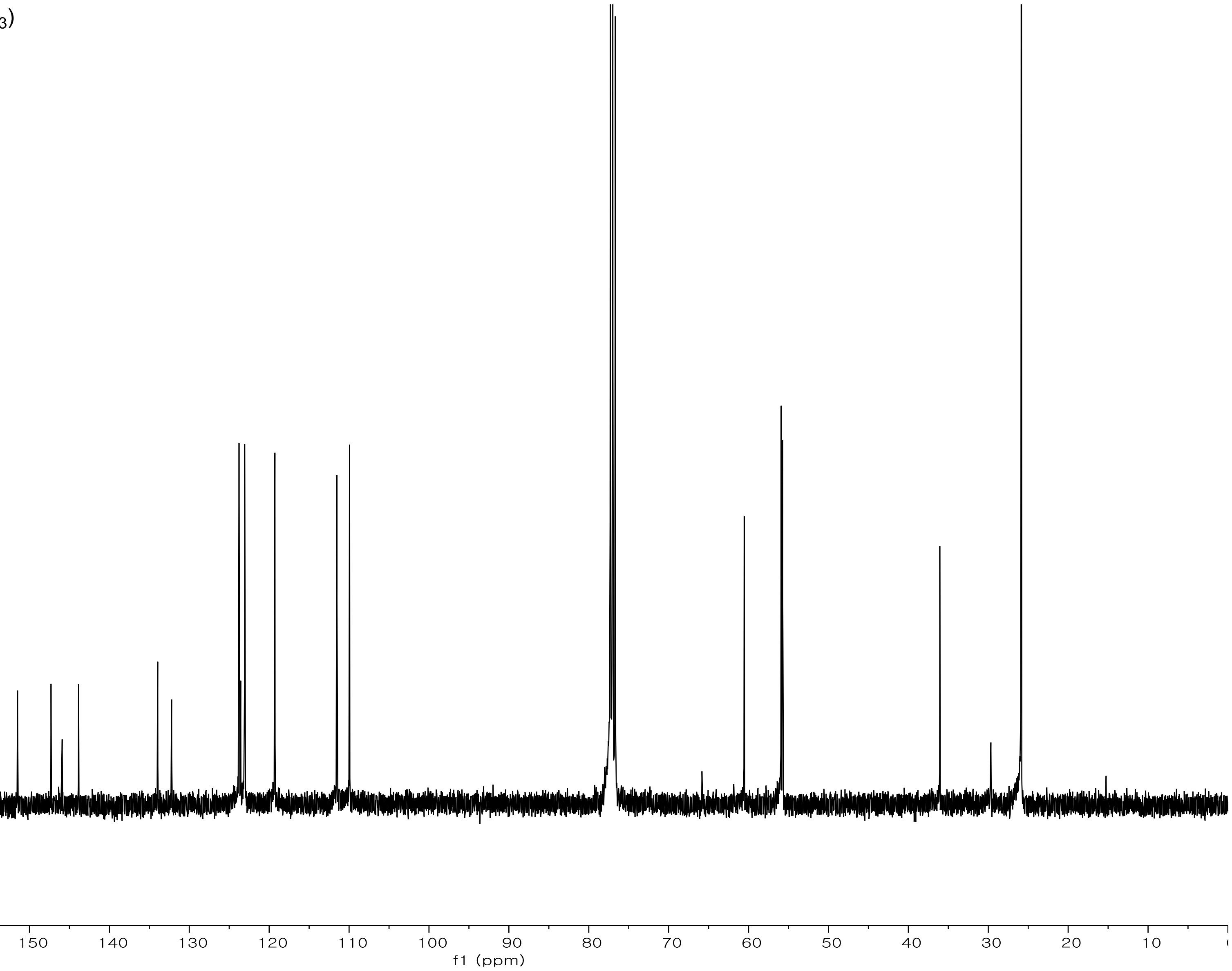

^1H NMR (400 MHz, $CDCl_3$)

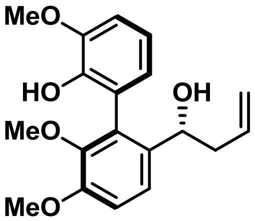

5d

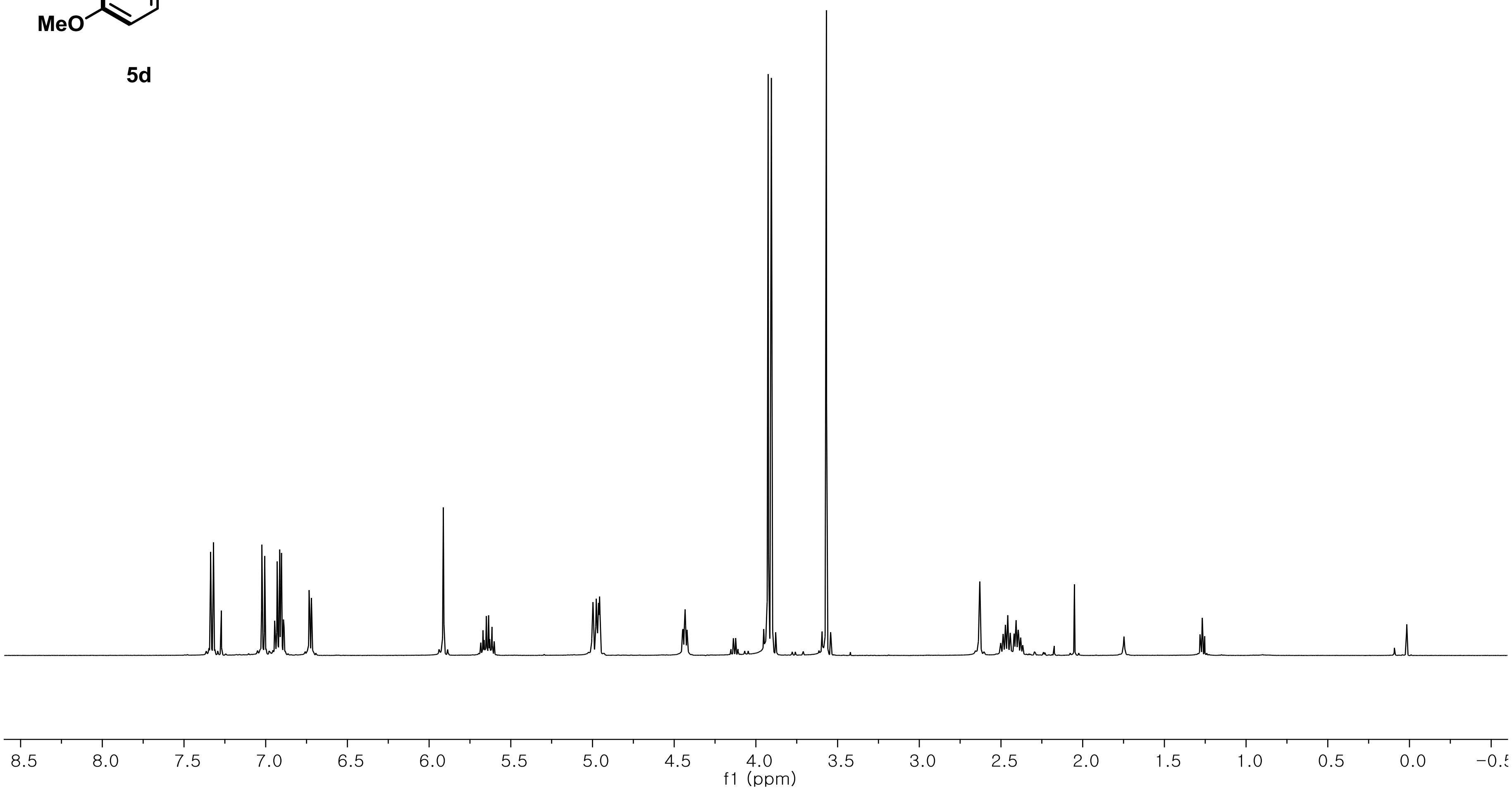

^{13}C NMR (101 MHz, $CDCl_3$)

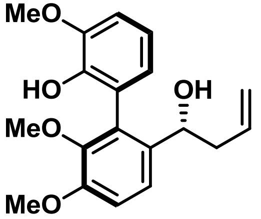

5d

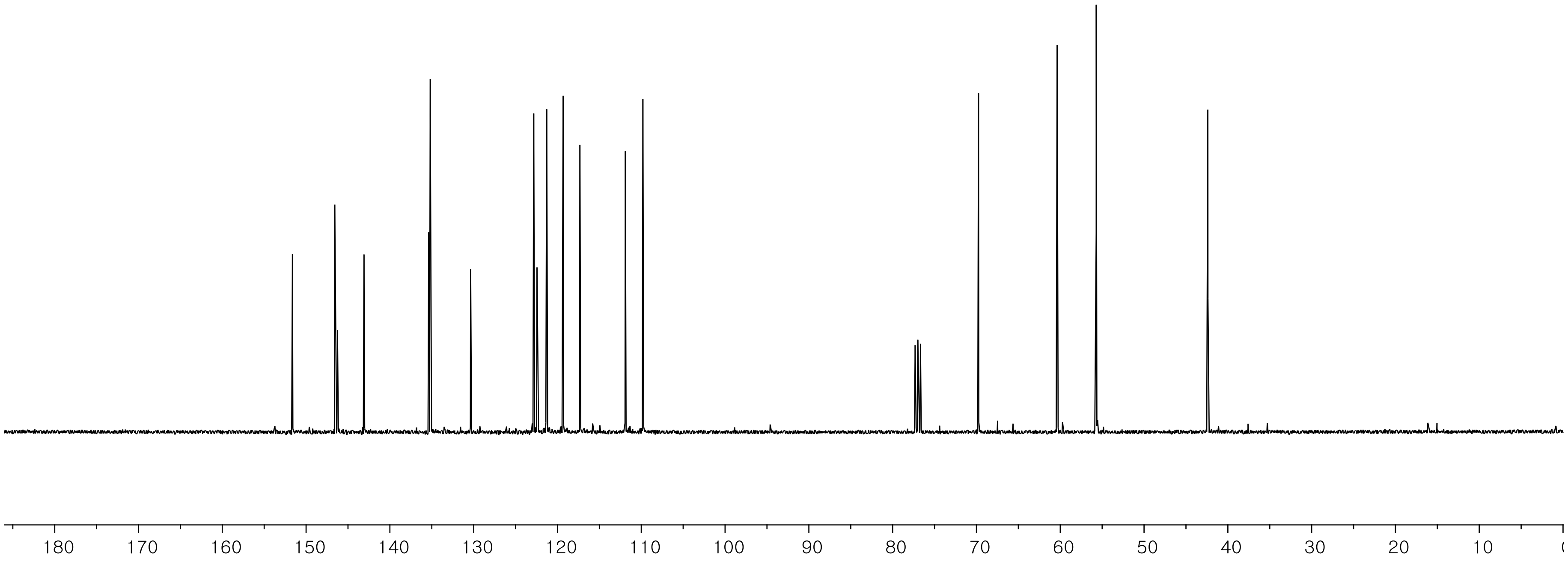

^{1}H NMR (400 MHz, $CDCl_3$)

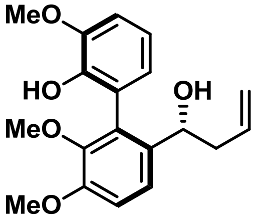

5d'

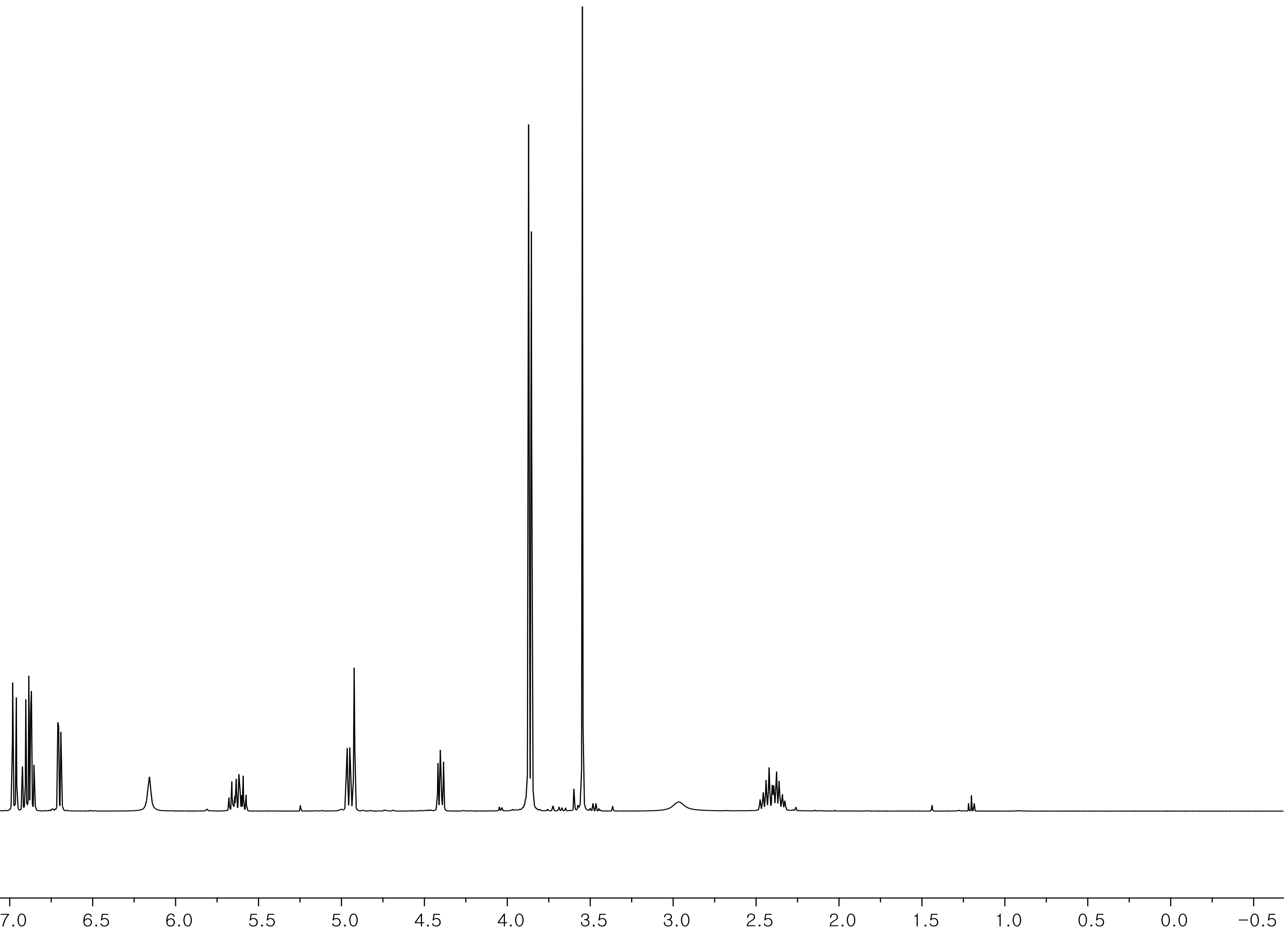

^{13}C NMR (101 MHz, $CDCl_3$)

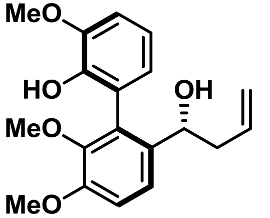

5d'

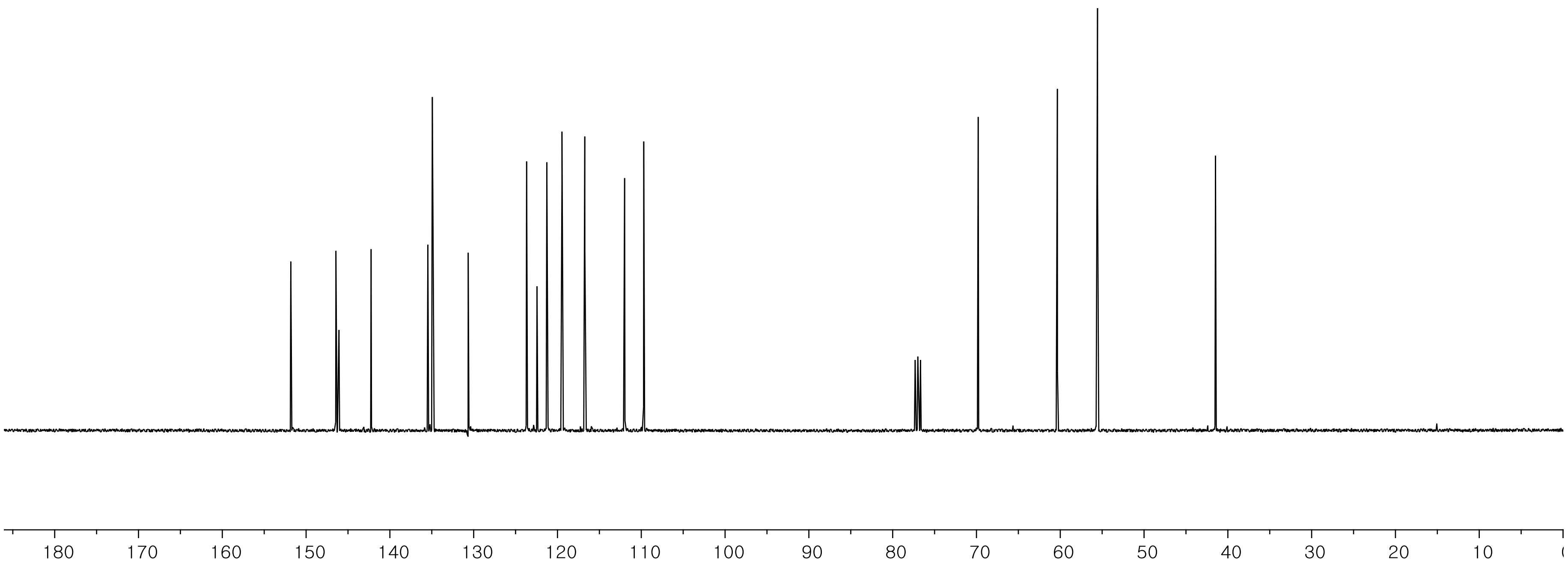

^{1}H NMR (499 MHz, $CDCl_3$)

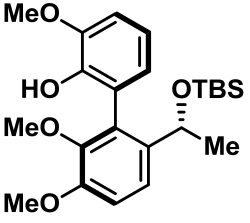

6a

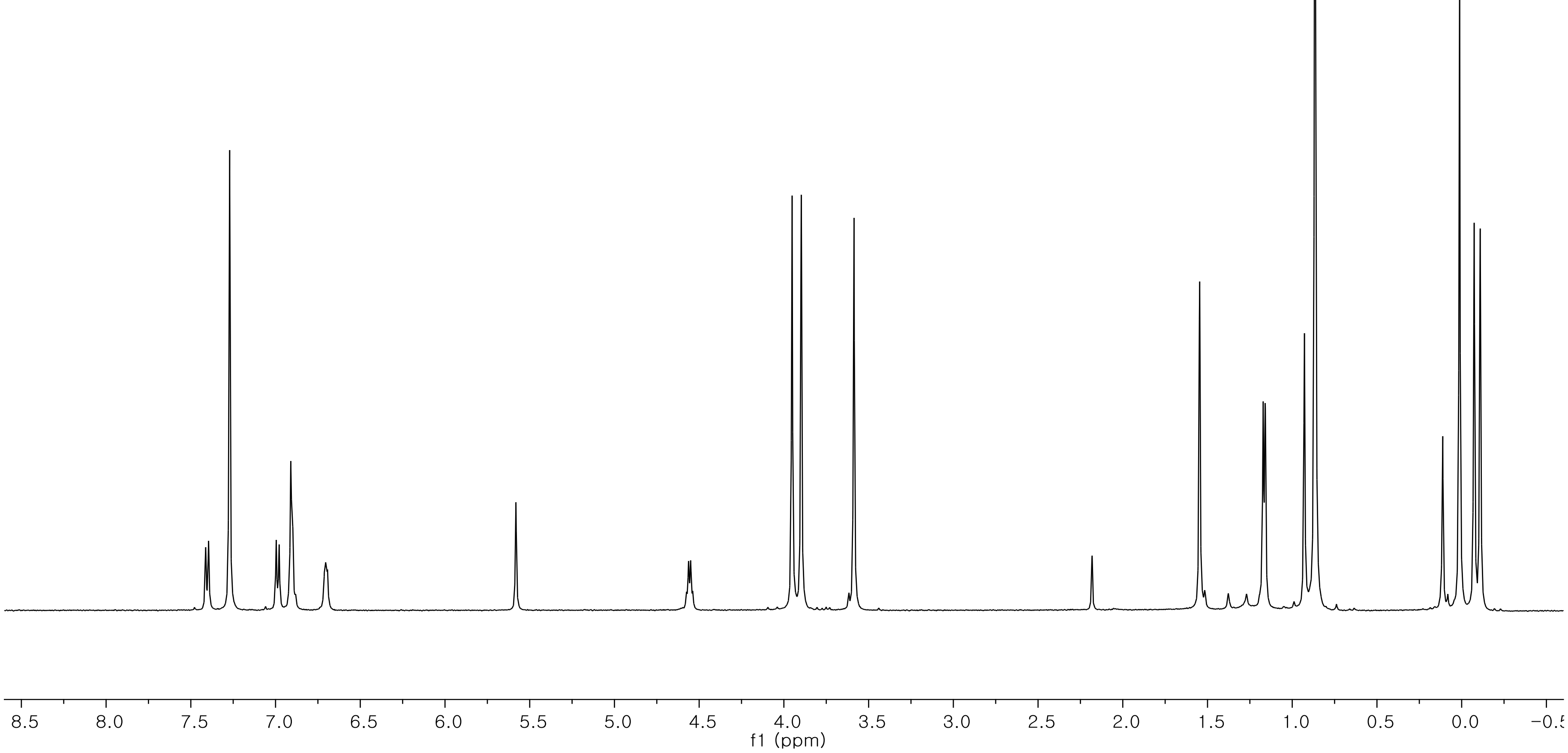

^{1}H NMR (101 MHz, $CDCl_3$)

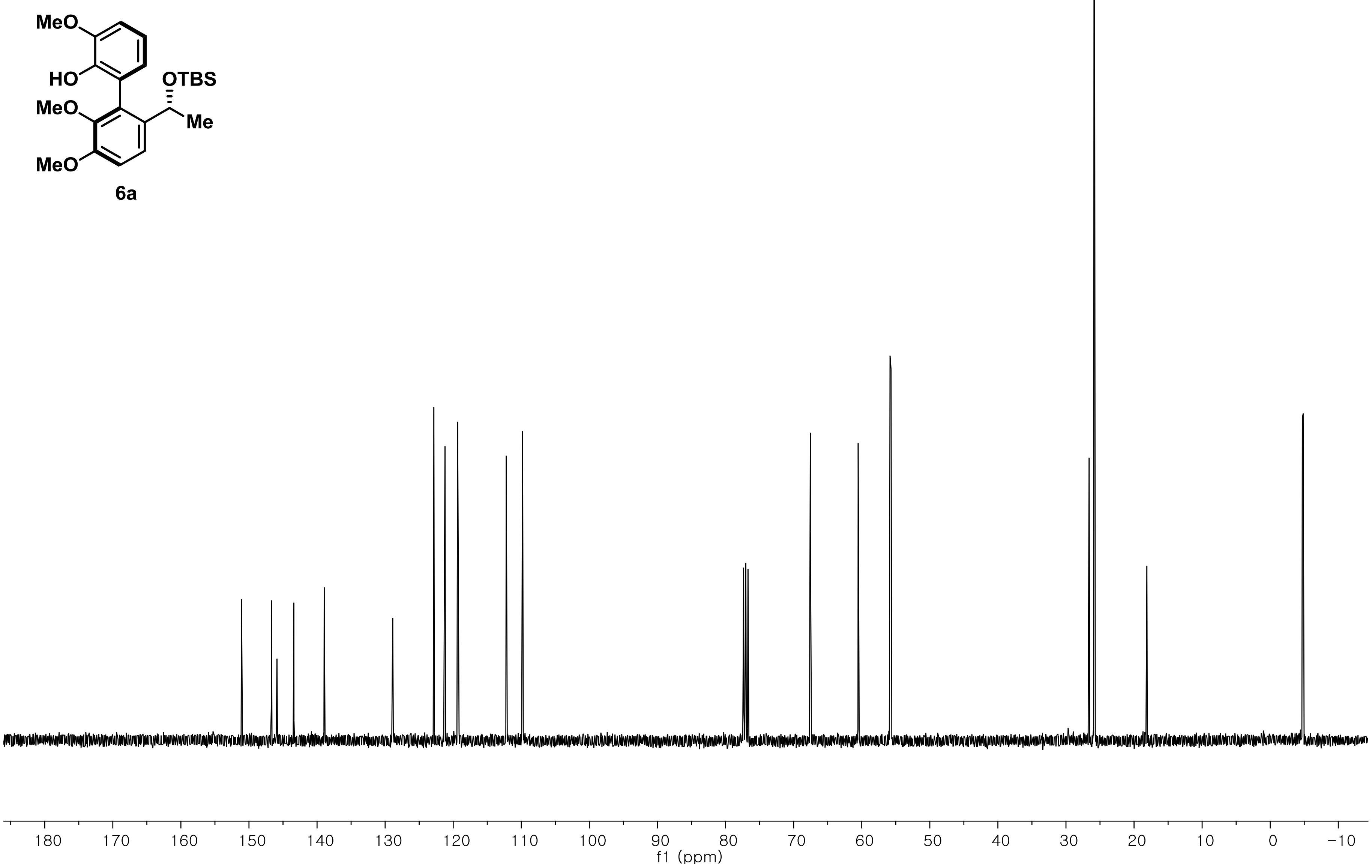

^{1}H NMR (499 MHz, $CDCl_3$)

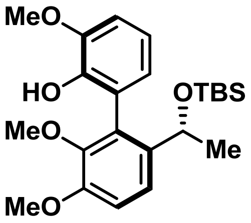

6a'

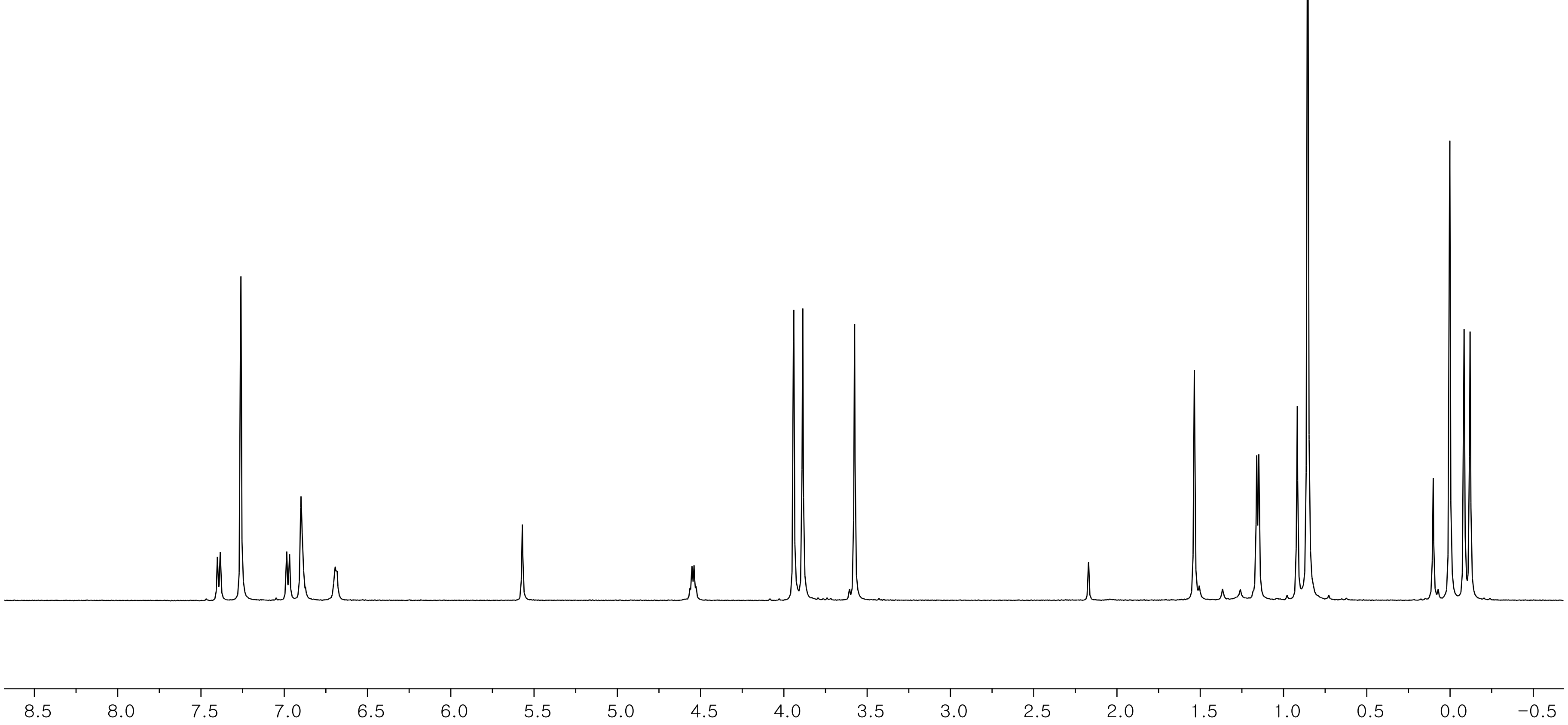

^{13}C NMR (101 MHz, $CDCl_3$)

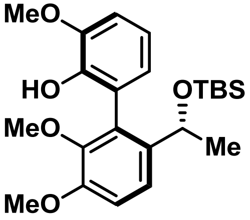

6a'

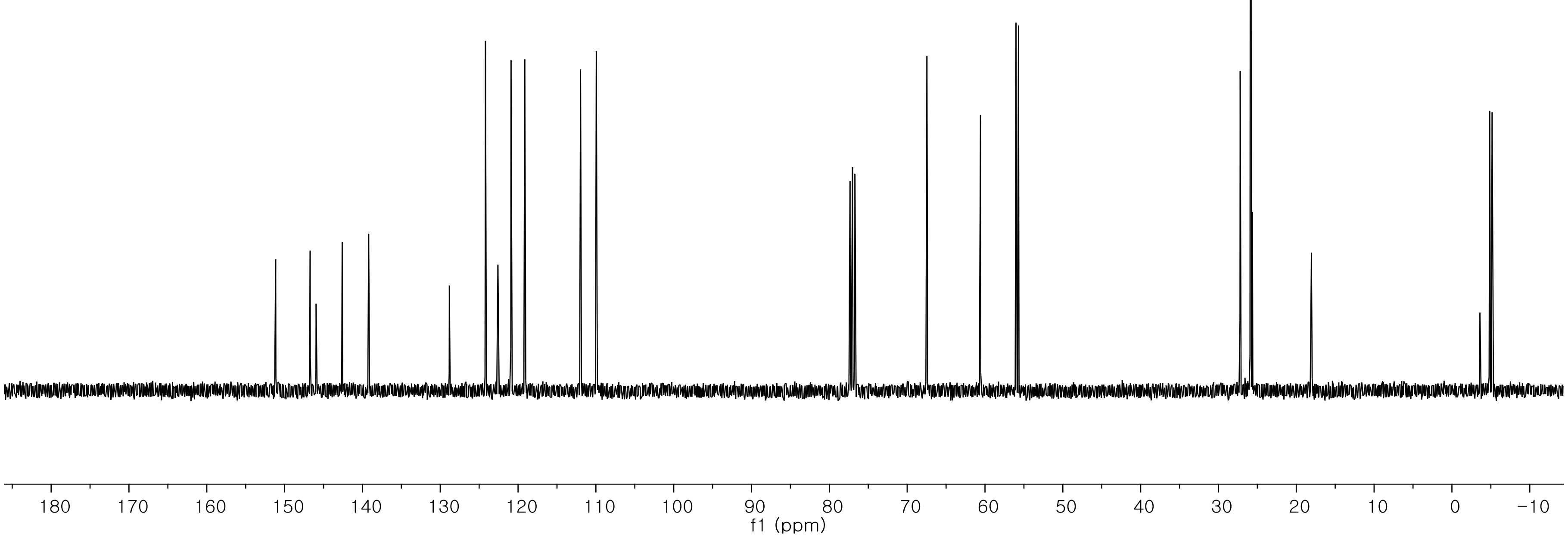

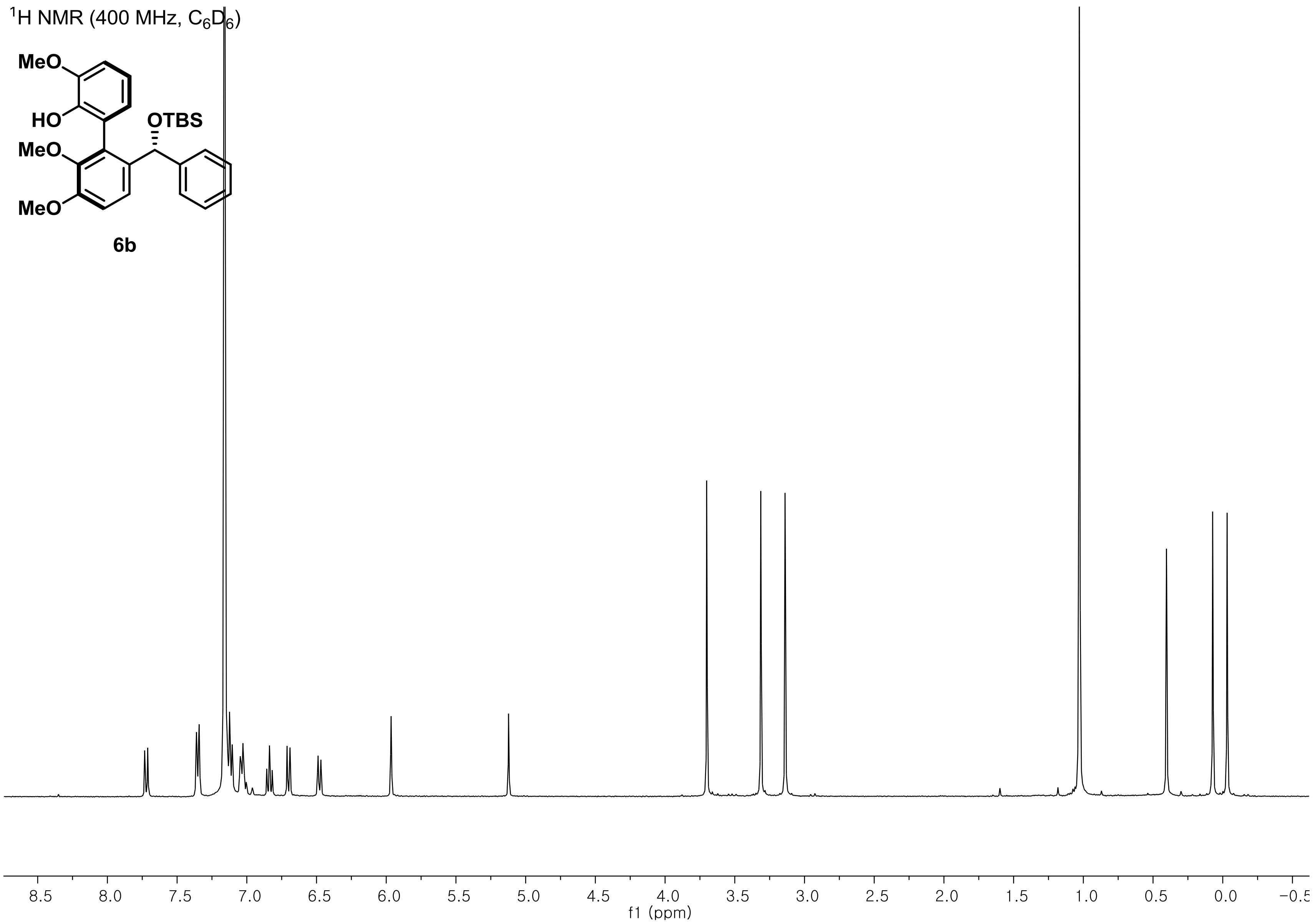

1H NMR (400 MHz, C6D6)
MeO
HO
OTBS
MeO
MeO
6b
8.5
8.0
7.5
7.0
6.5
6.0
5.5
5.0
4.5
4.0
3.5
3.0
2.5
2.0
1.5
1.0
0.5
0.0
-0.5
f1 (ppm)

^{13}C NMR (101 MHz, C_6D_6)

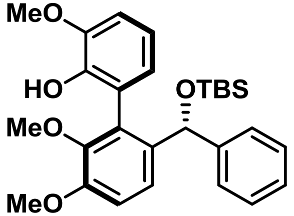

6b

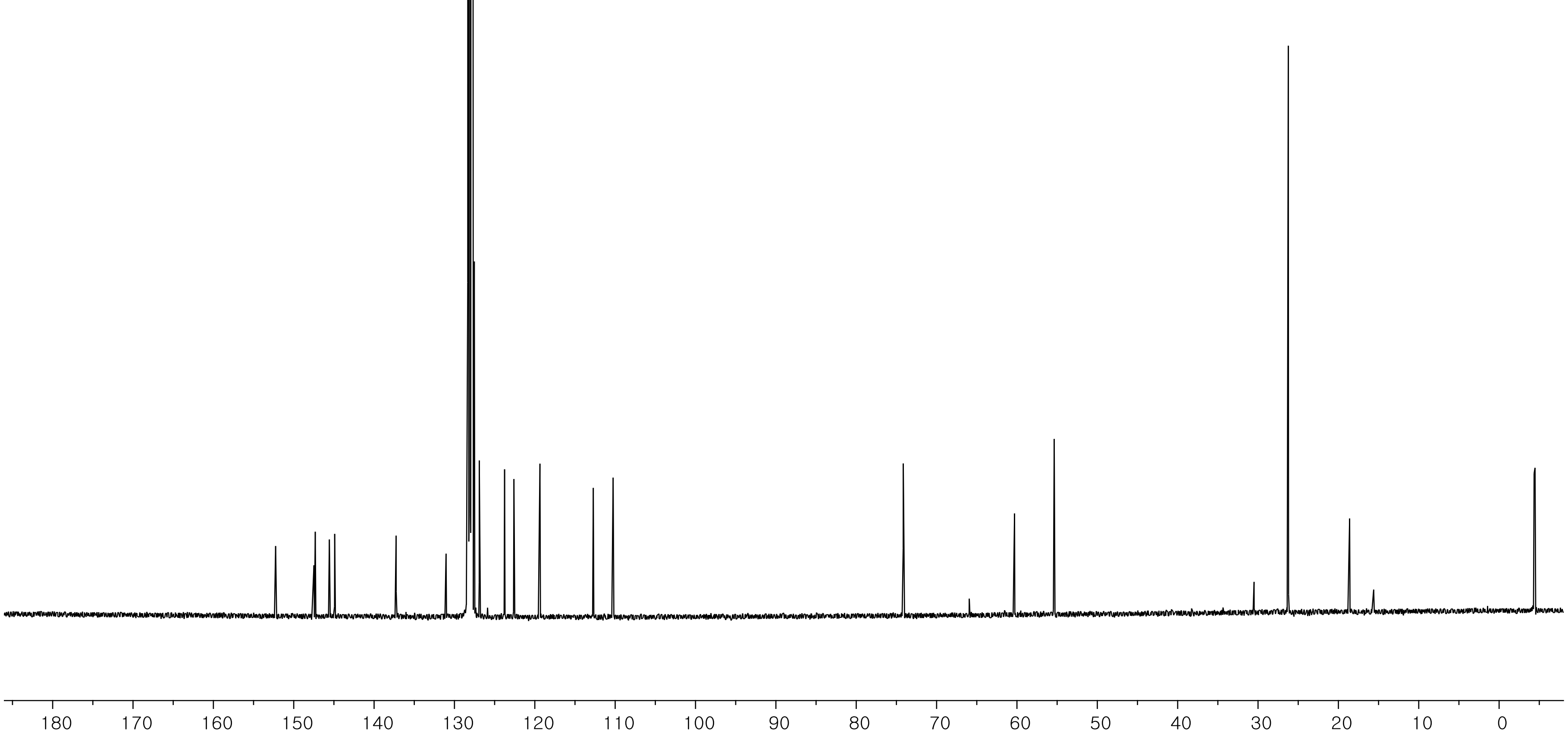

^{1}H NMR (400 MHz, C_6D_6)

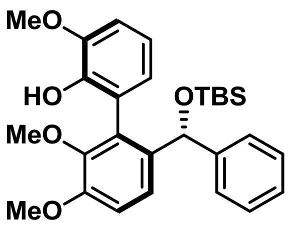

6b'

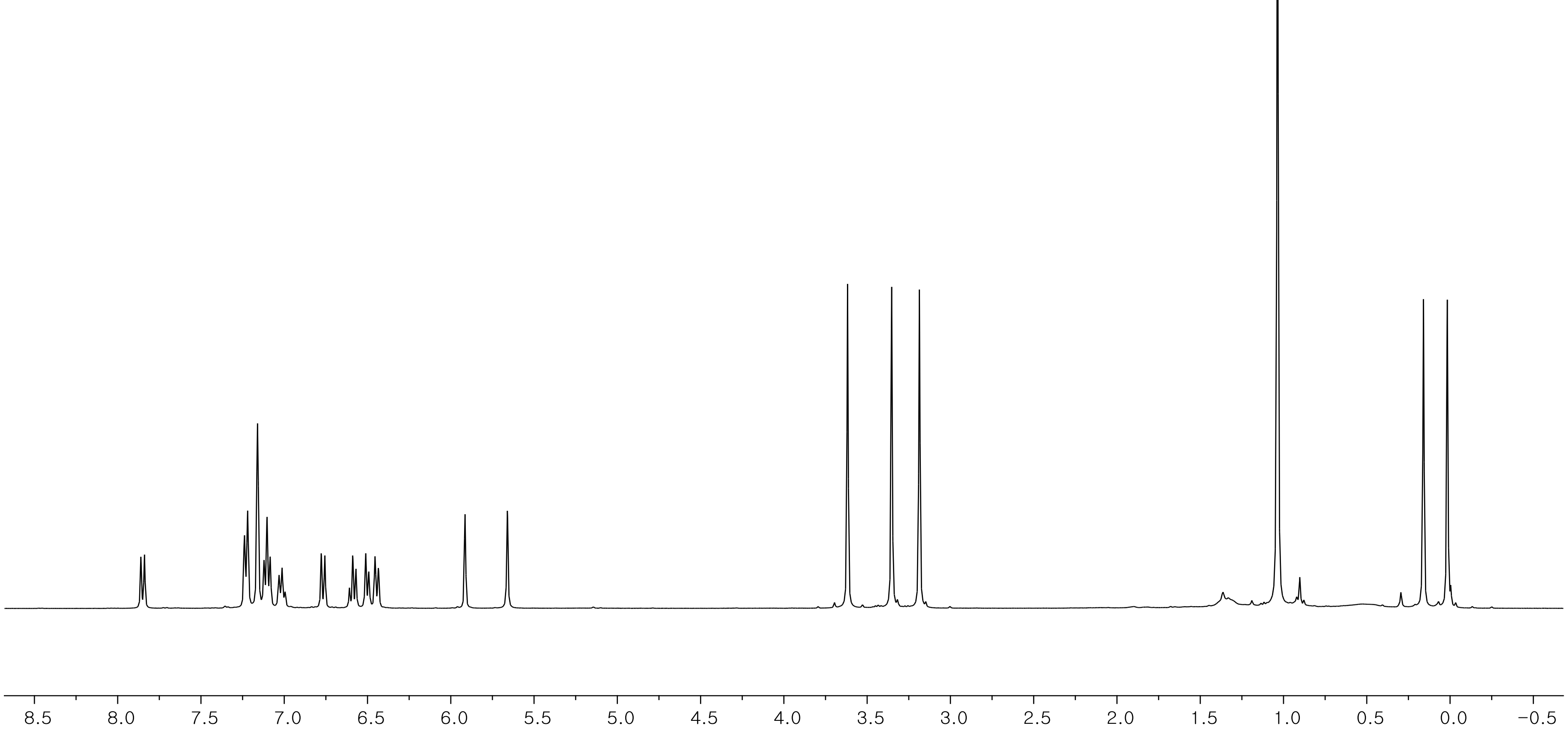

^{13}C NMR (101 MHz, C_6D_6)

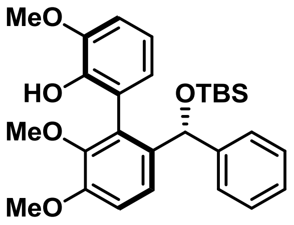

6b'

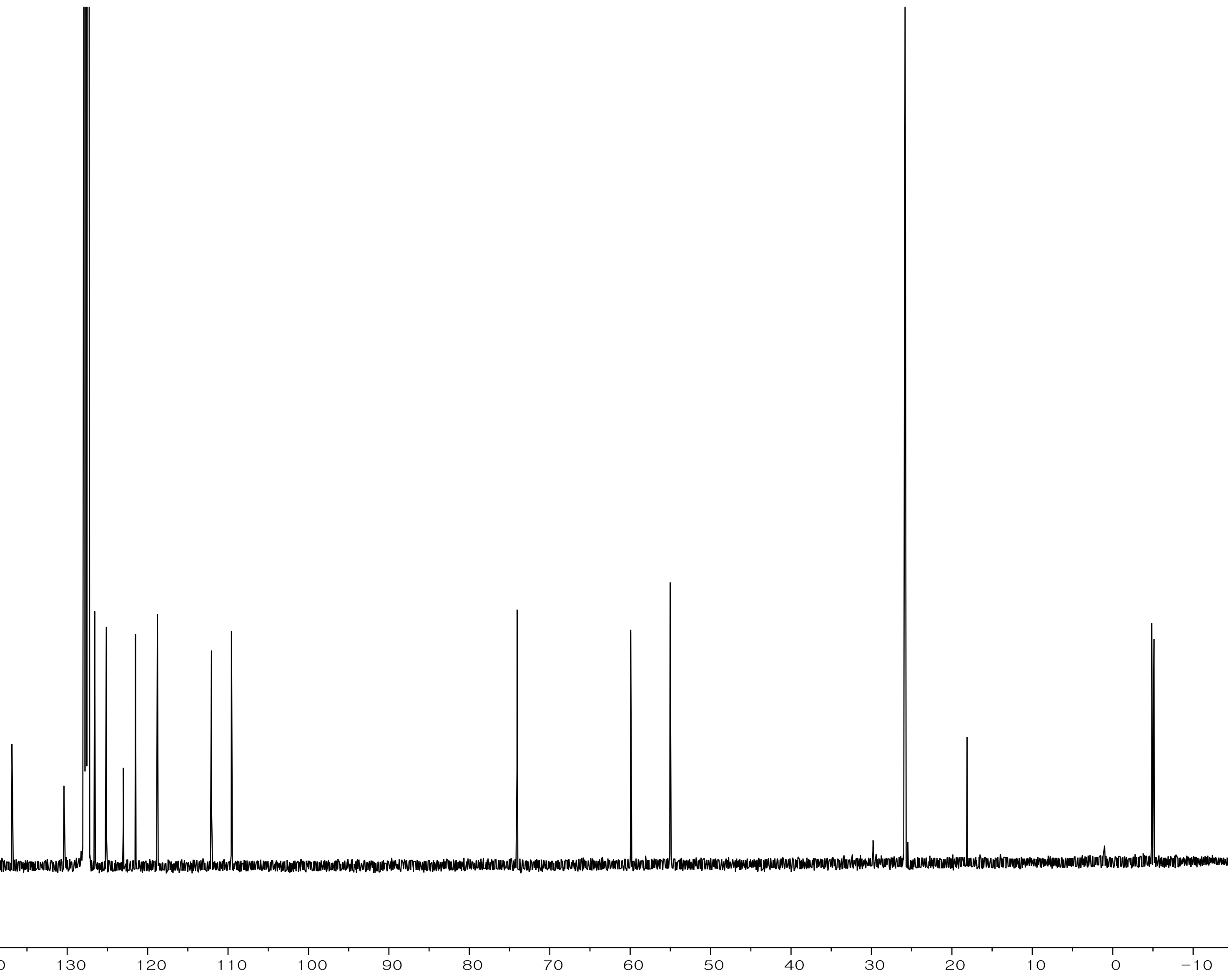

^{1}H NMR (499 MHz, $CDCl_3$)

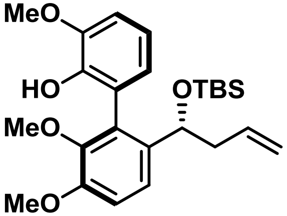

6d

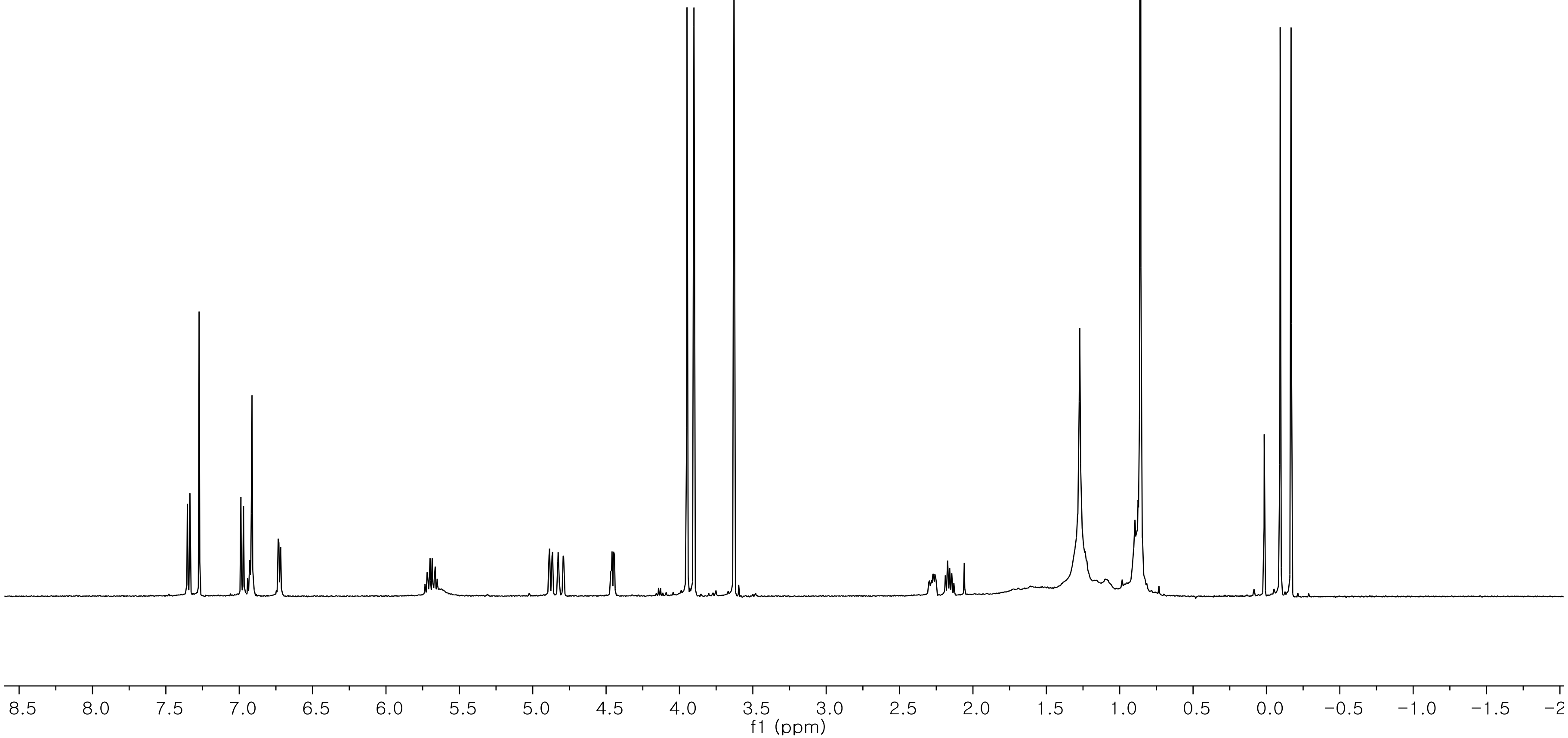

^{13}C NMR (101 MHz, $CDCl_3$)

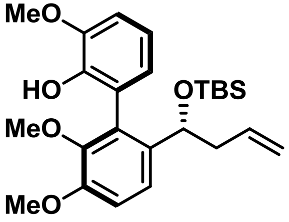

6d

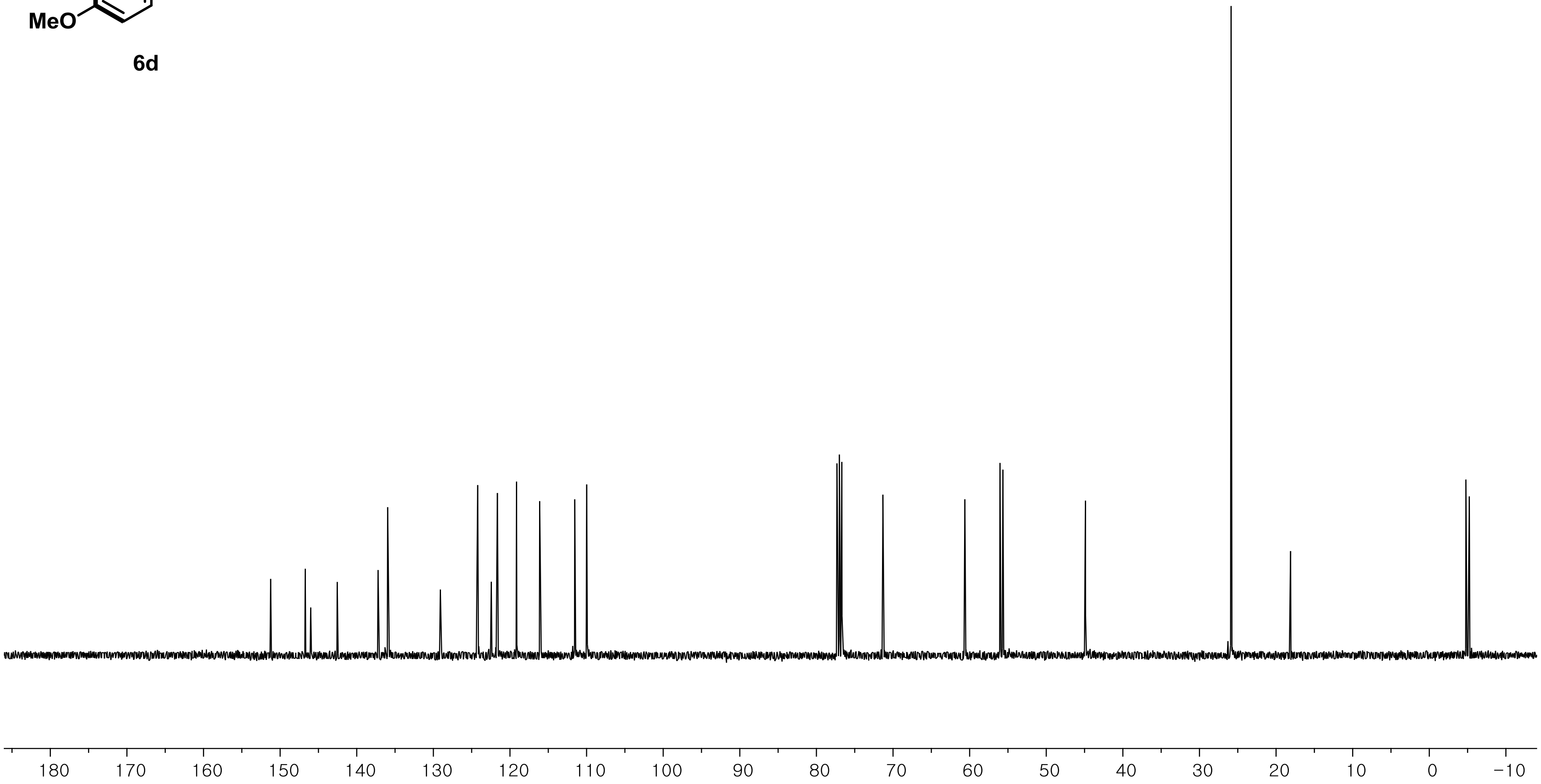

^{1}H NMR (499 MHz, $CDCl_3$)

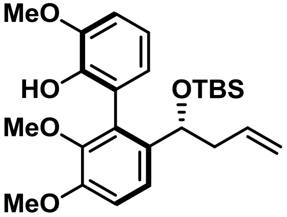

6d'

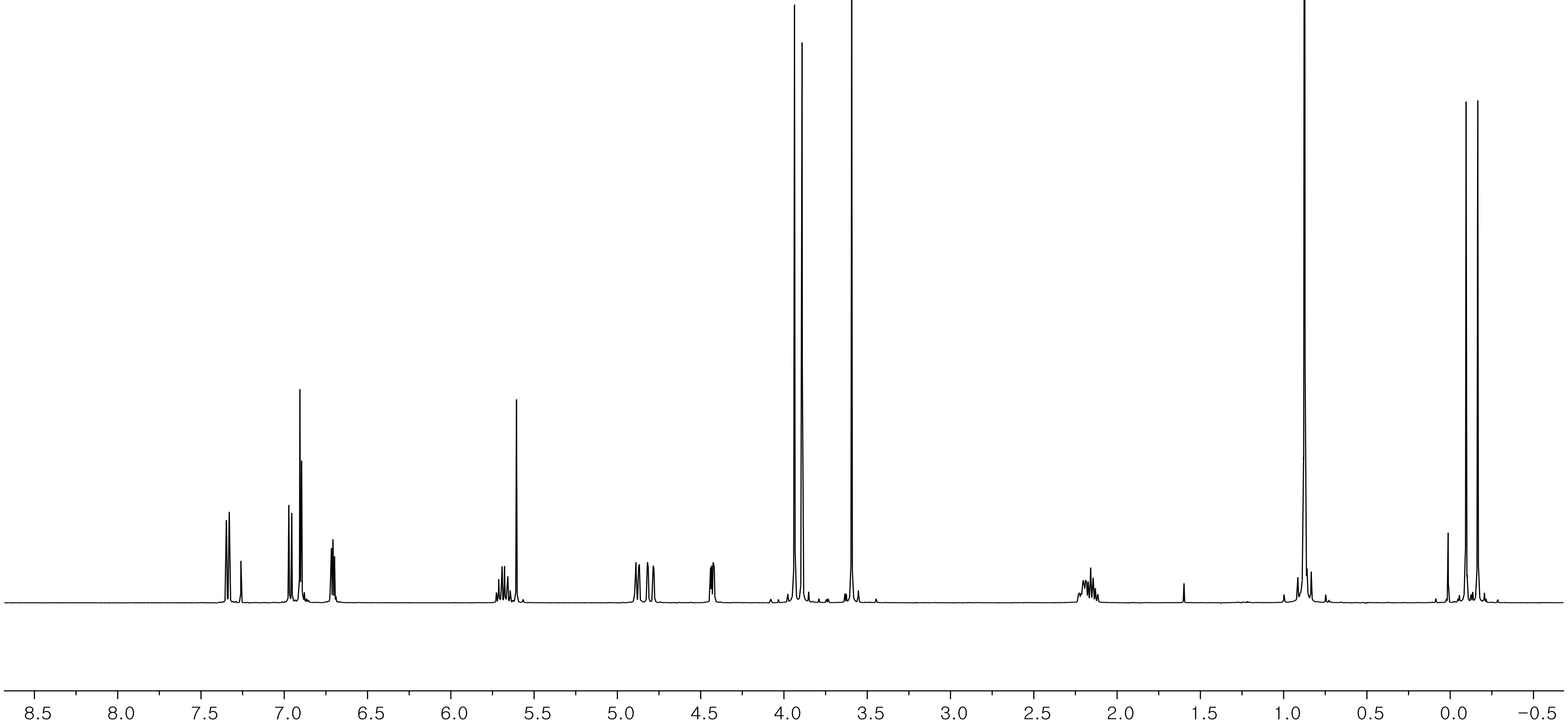

^{13}C NMR (101 MHz, $CDCl_3$)

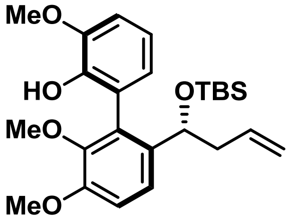

6d'

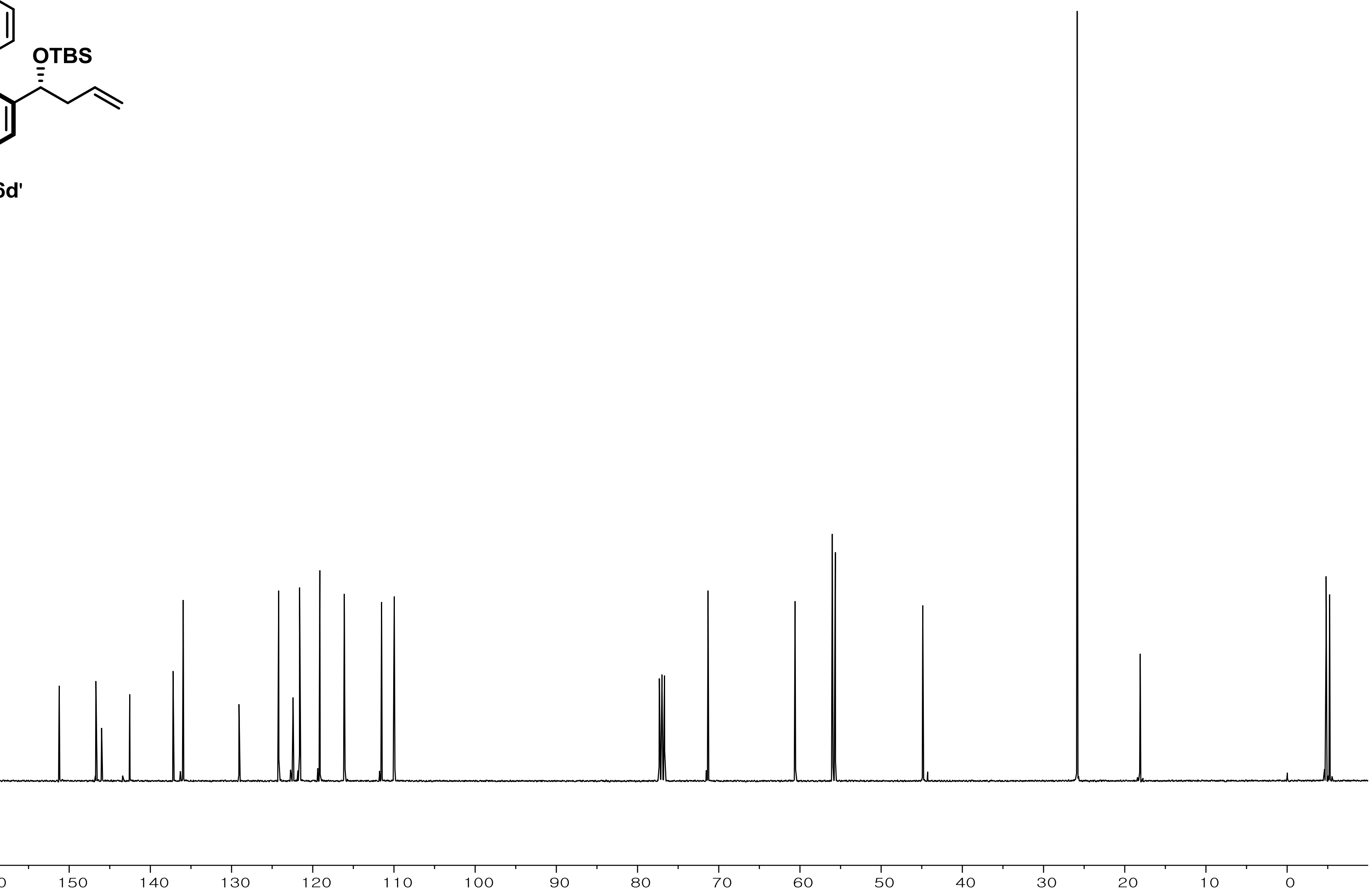

^{1}H NMR (400 MHz, $CDCl_3$)

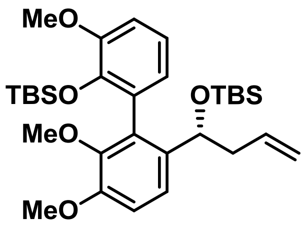

TBS-6d + TBS-6d'

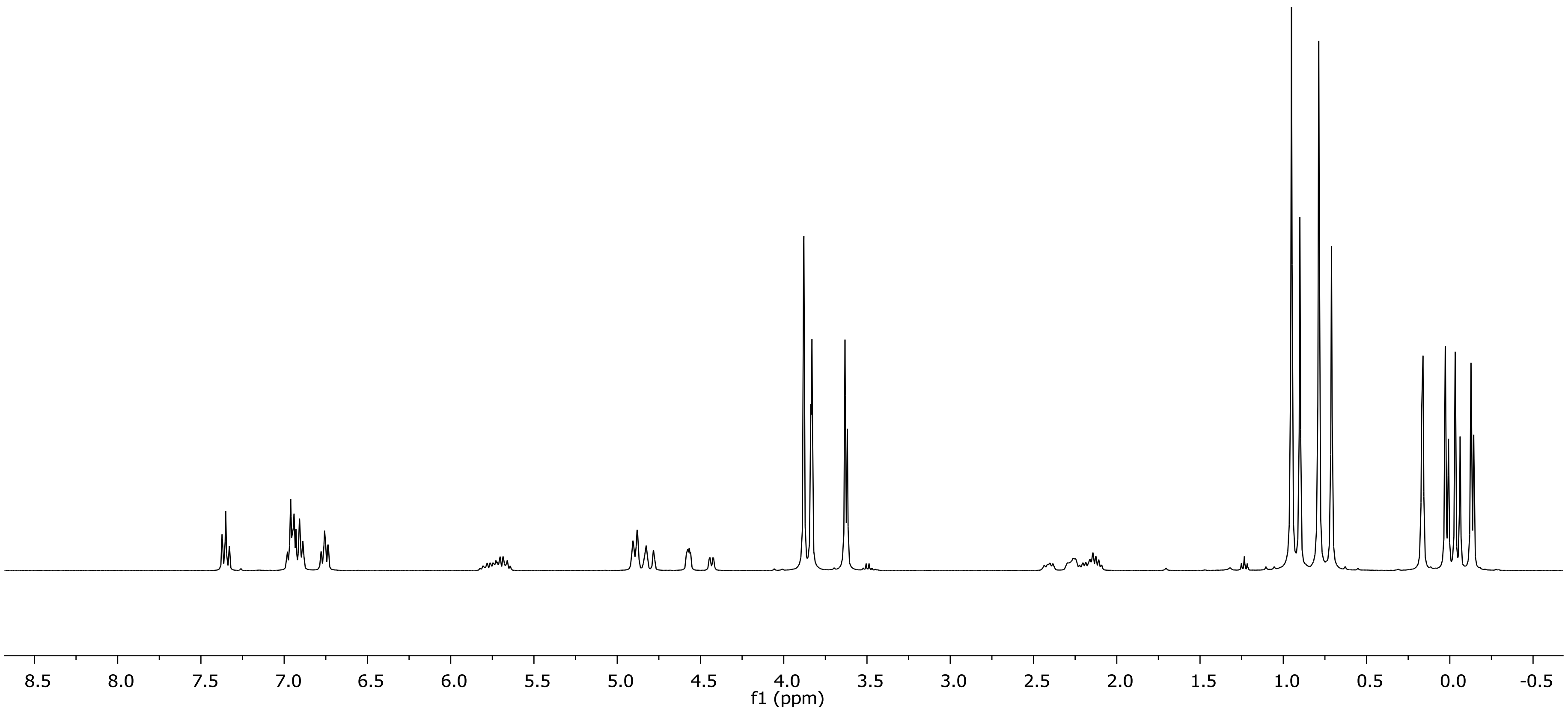

^{13}C NMR (101 MHz, $CDCl_3$)

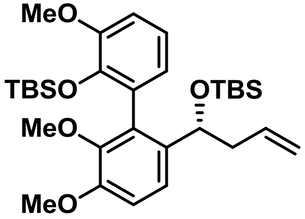

TBS-6d + TBS-6d'

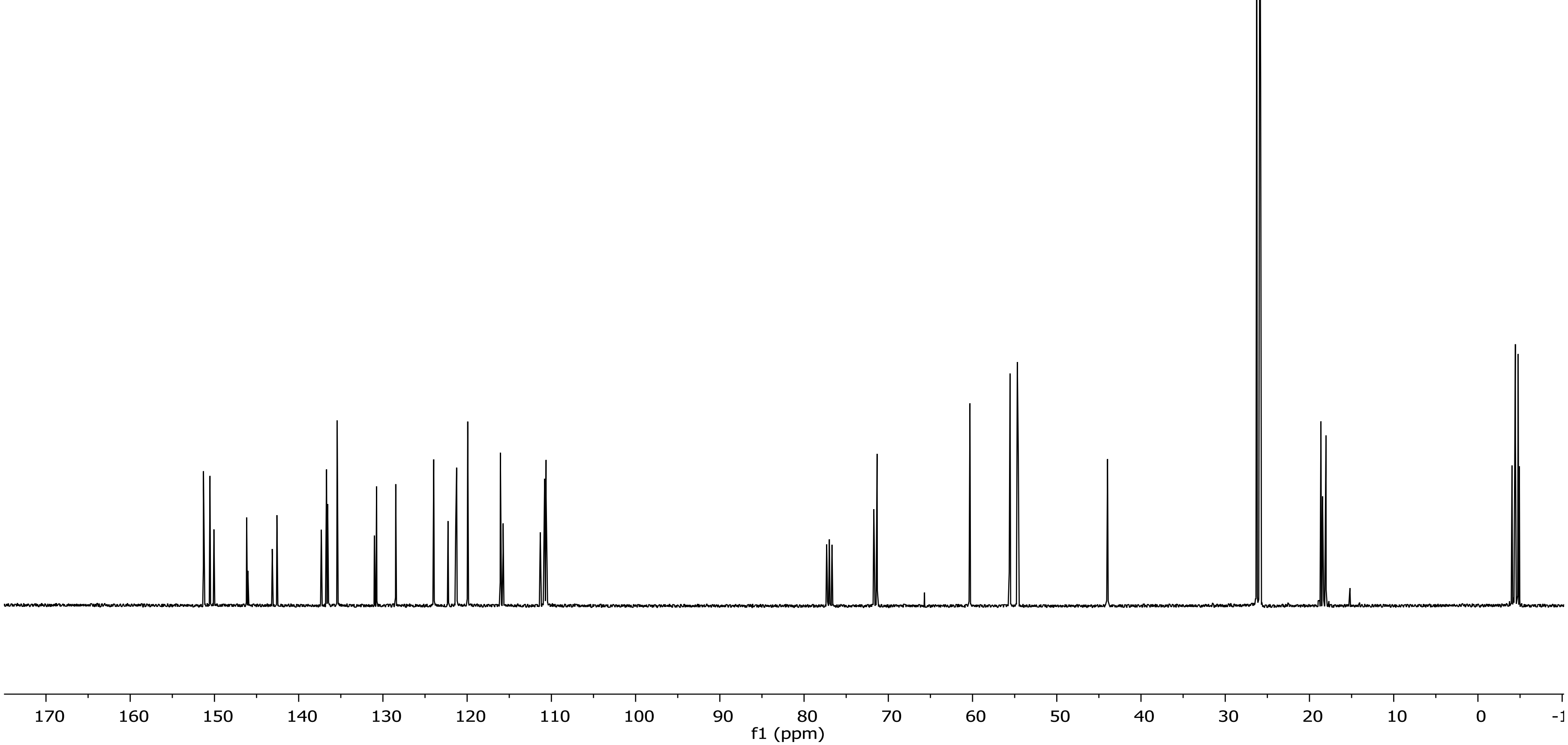

^{1}H NMR (499 MHz, $CDCl_3$)

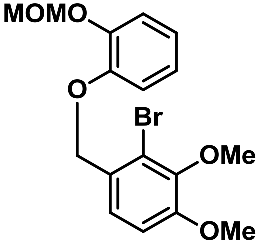

2g

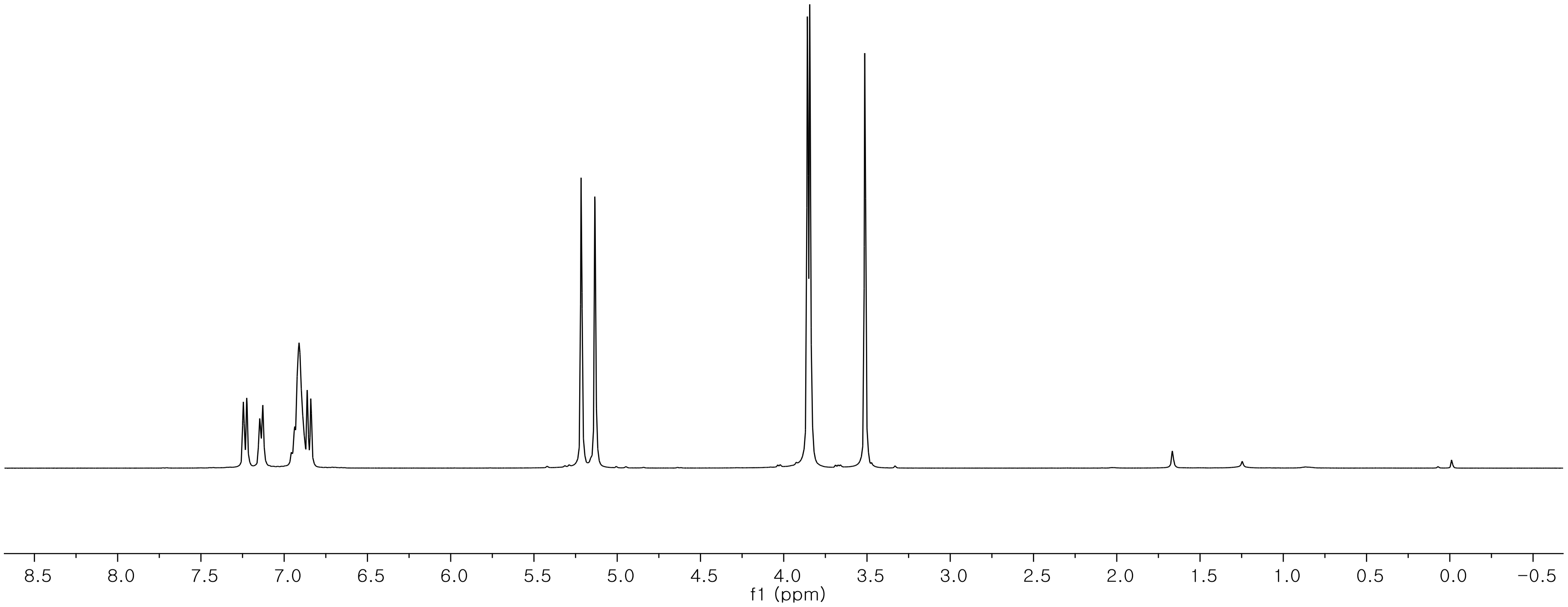

^{13}C NMR (101 MHz, $CDCl_3$)

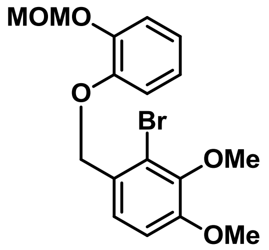

2g

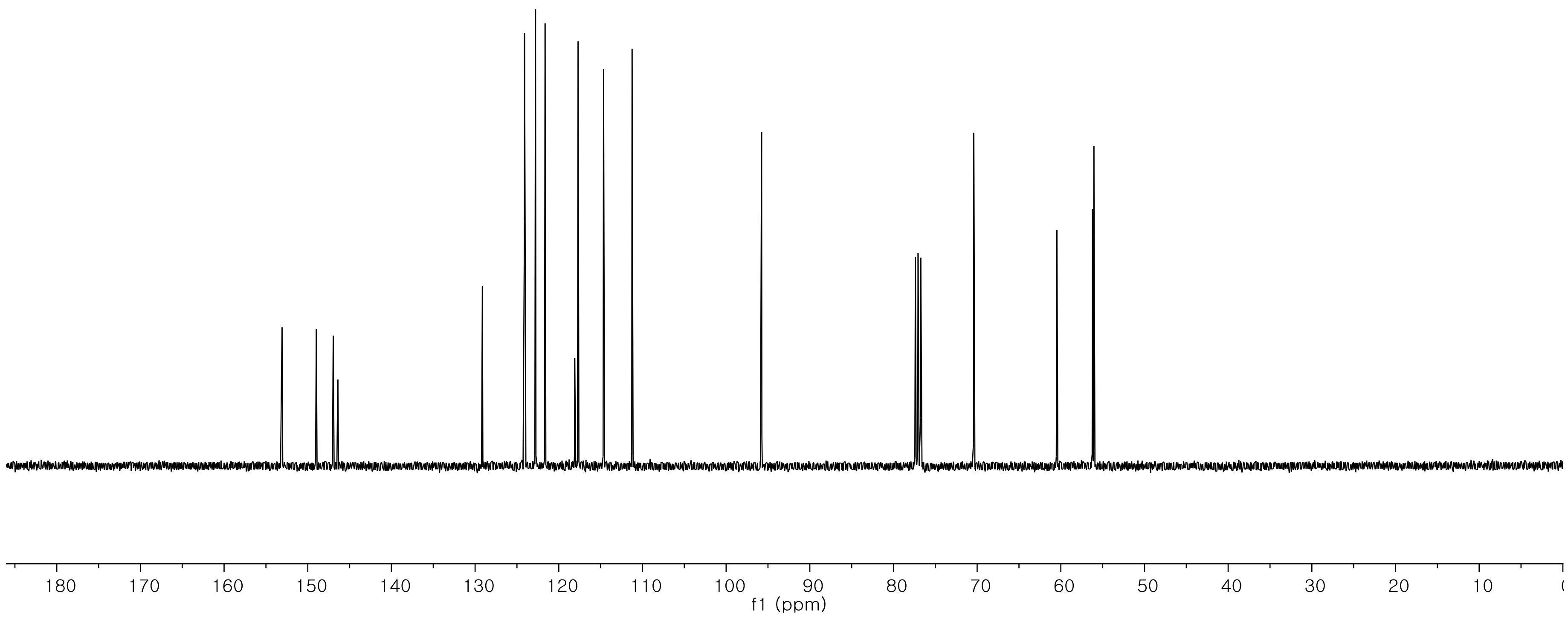

^1H NMR (400 MHz, $CDCl_3$)

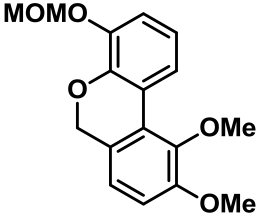

3a

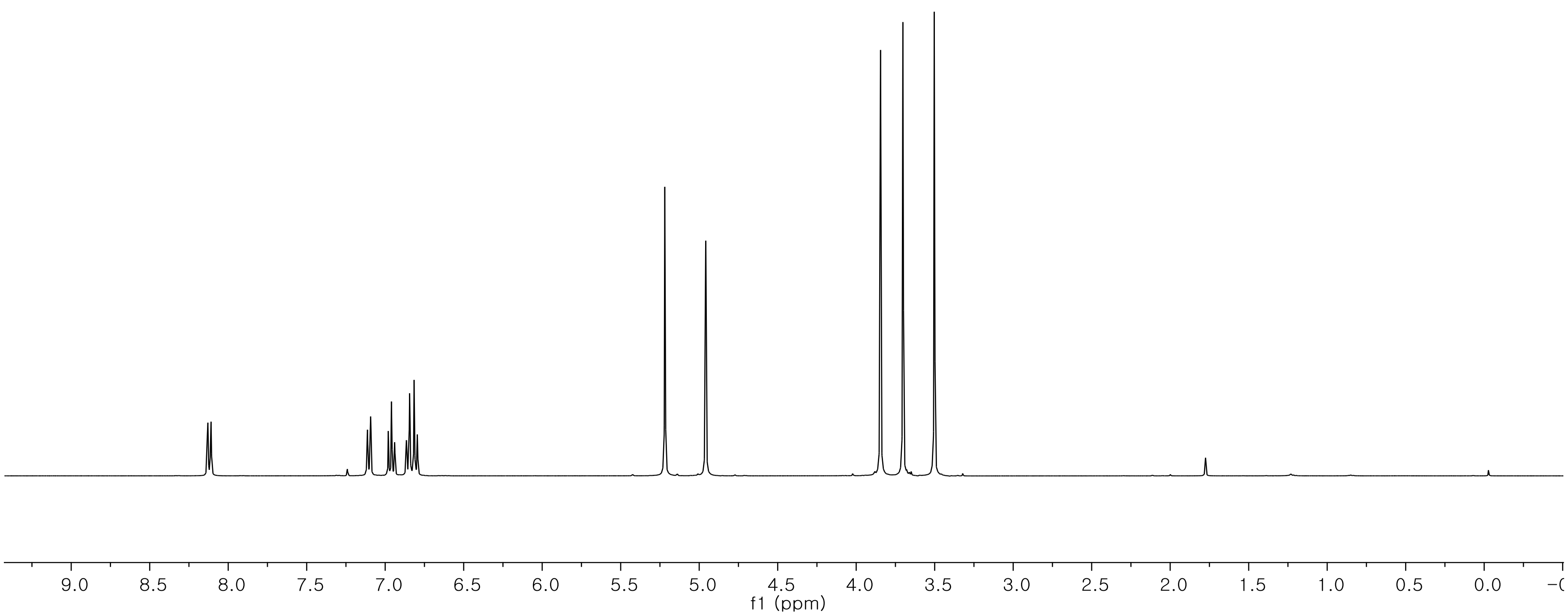

^{13}C NMR (101 MHz, $CDCl_3$)

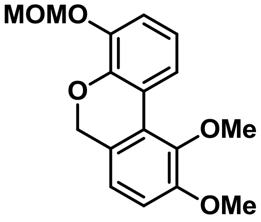

3a

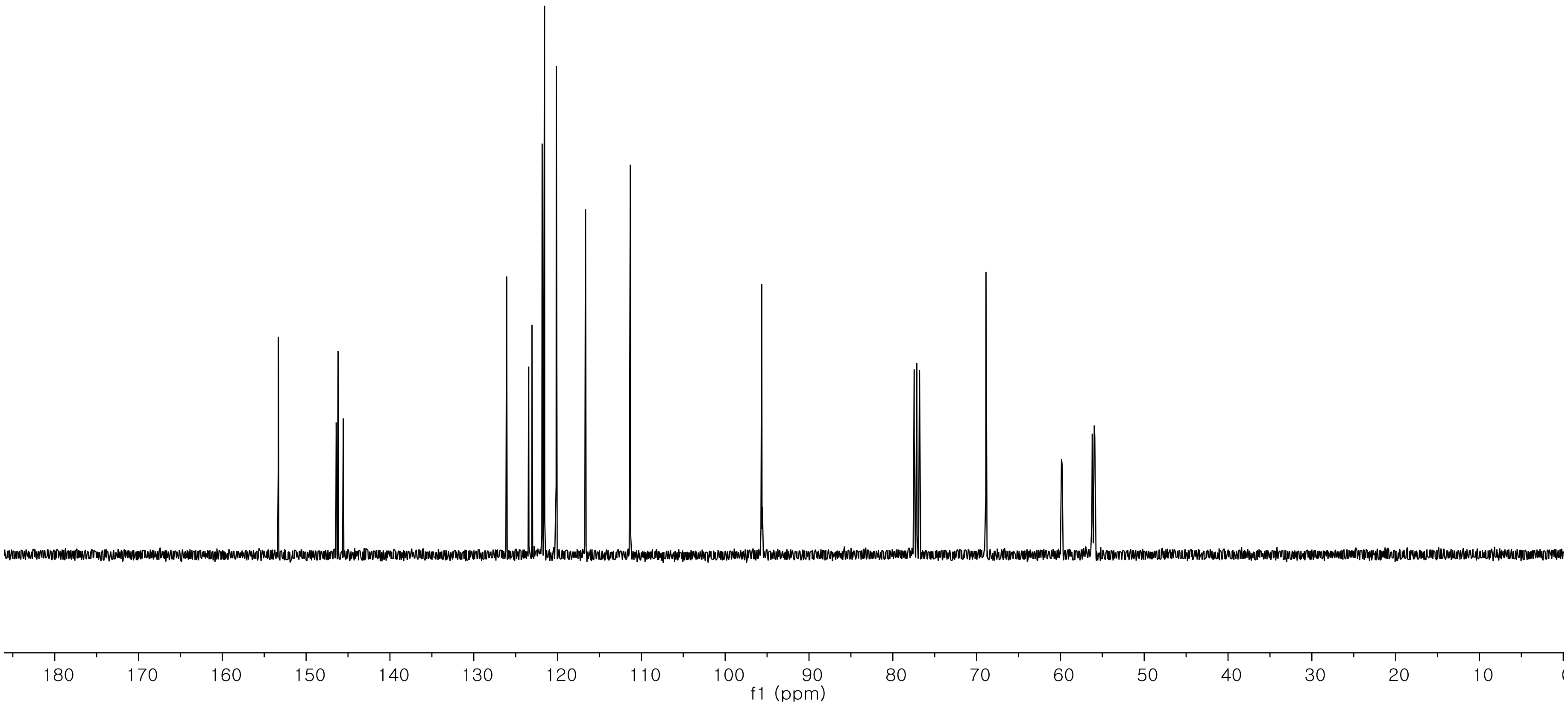

^{1}H NMR (400 MHz, $CDCl_3$)

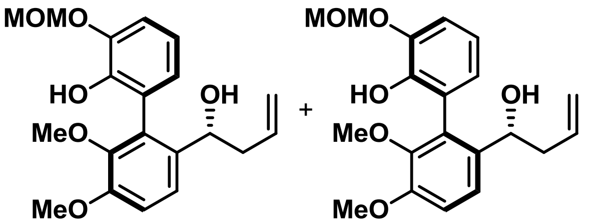

5e **5e'**

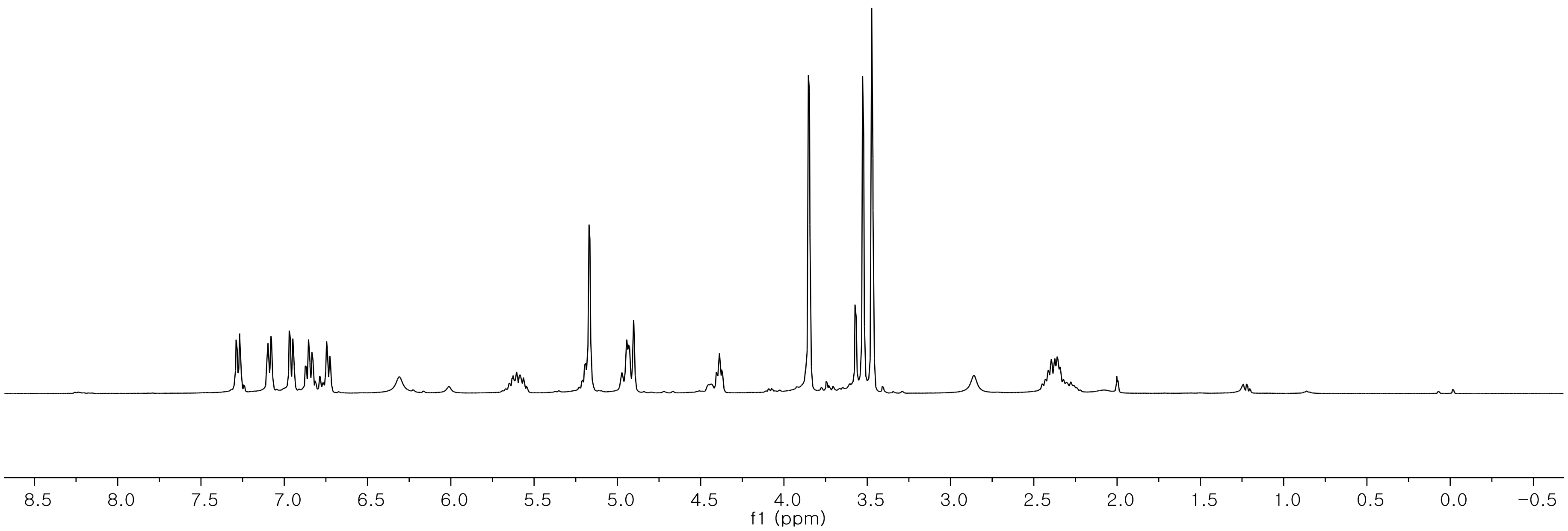

^{13}C NMR (101 MHz, $CDCl_3$)

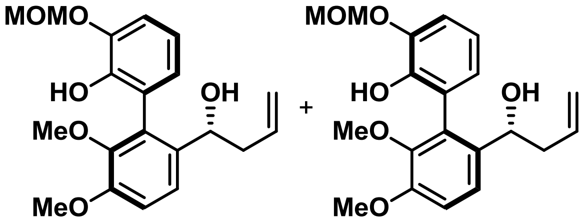

5e **5e'**

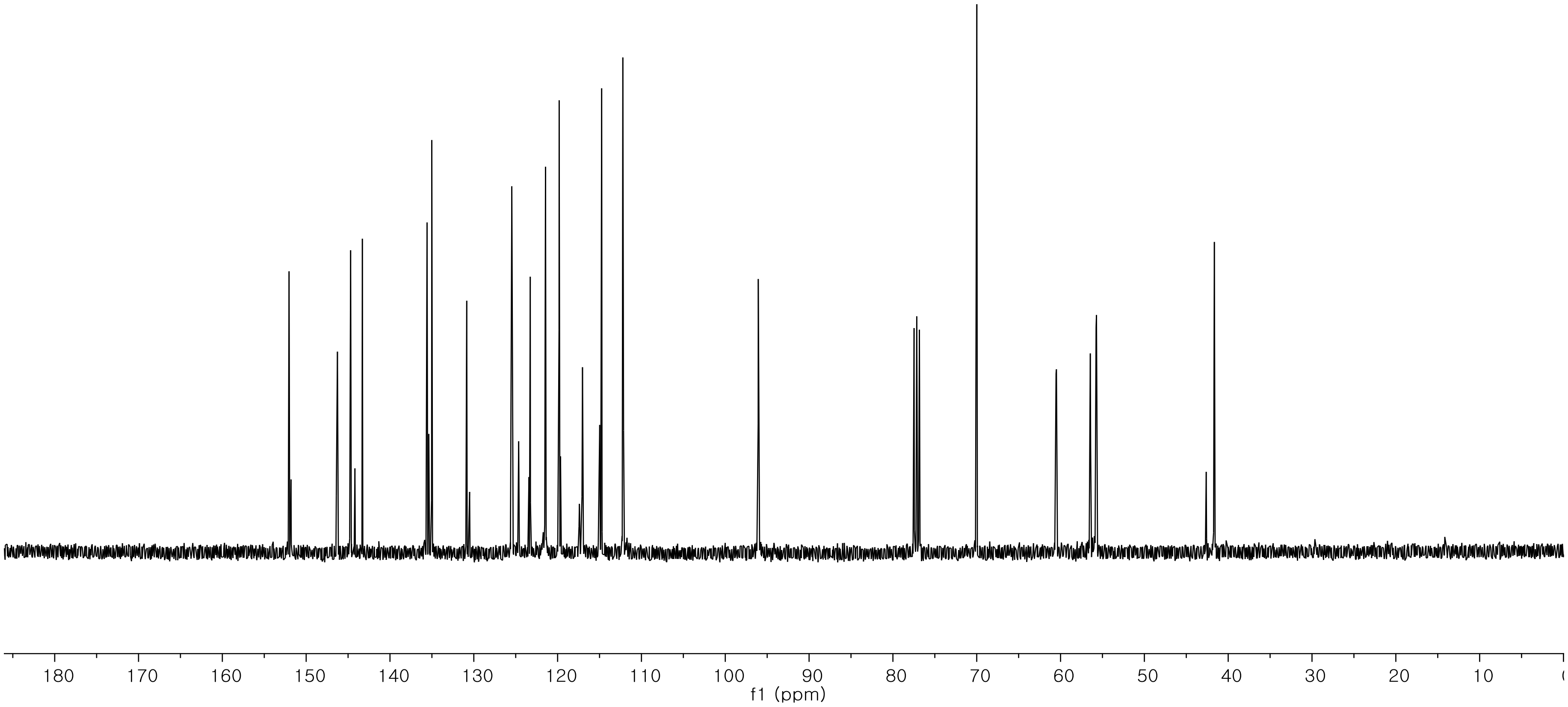

^{1}H NMR (400 MHz, $CDCl_3$)

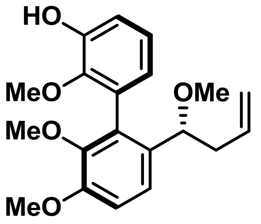

6e

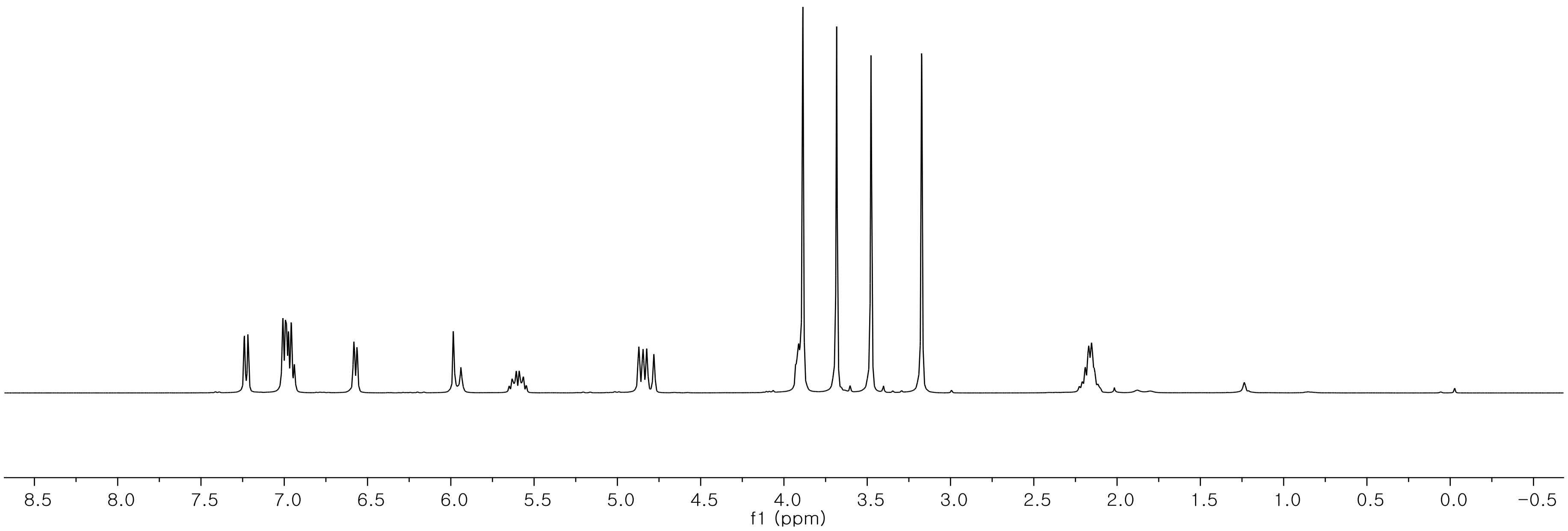

^{13}C NMR (101 MHz, $CDCl_3$)

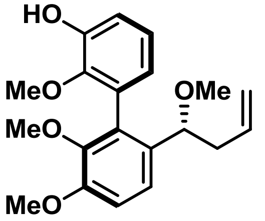

6e

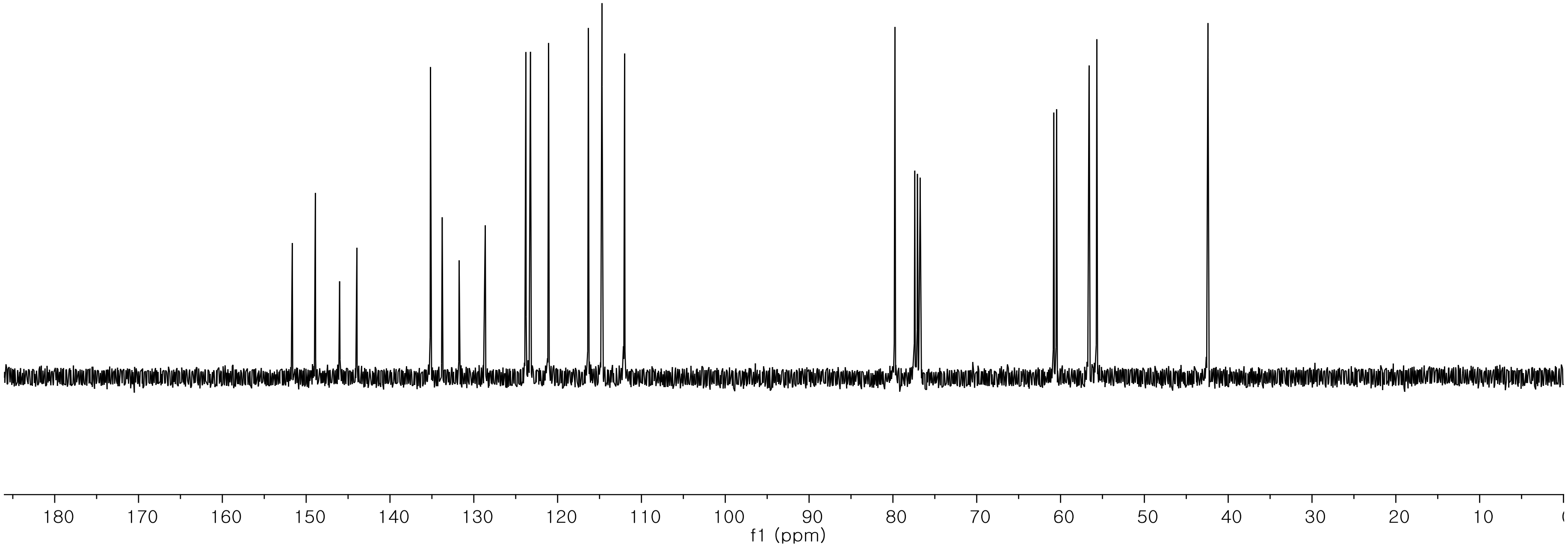

^{1}H NMR (400 MHz, C_6D_6)

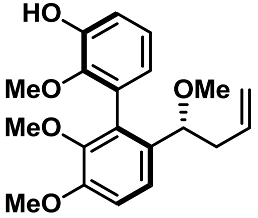

6e'

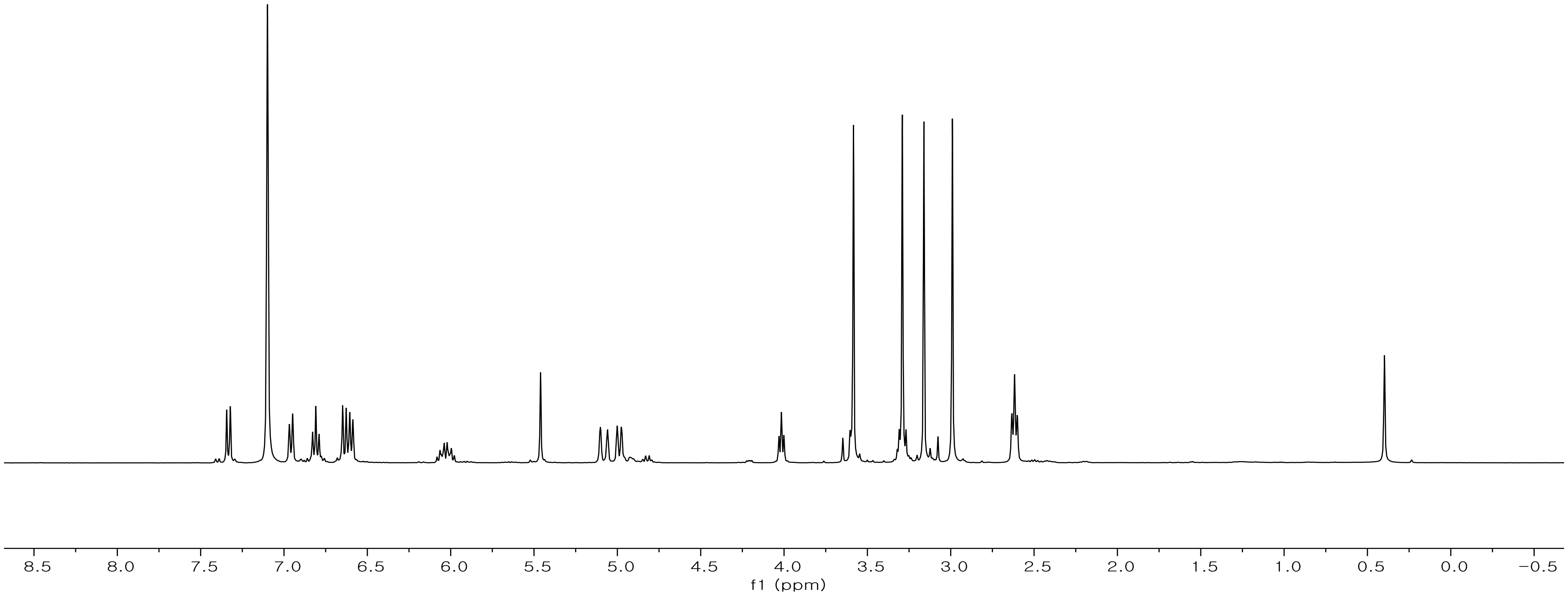

^{13}C NMR (101 MHz, $CDCl_3$)

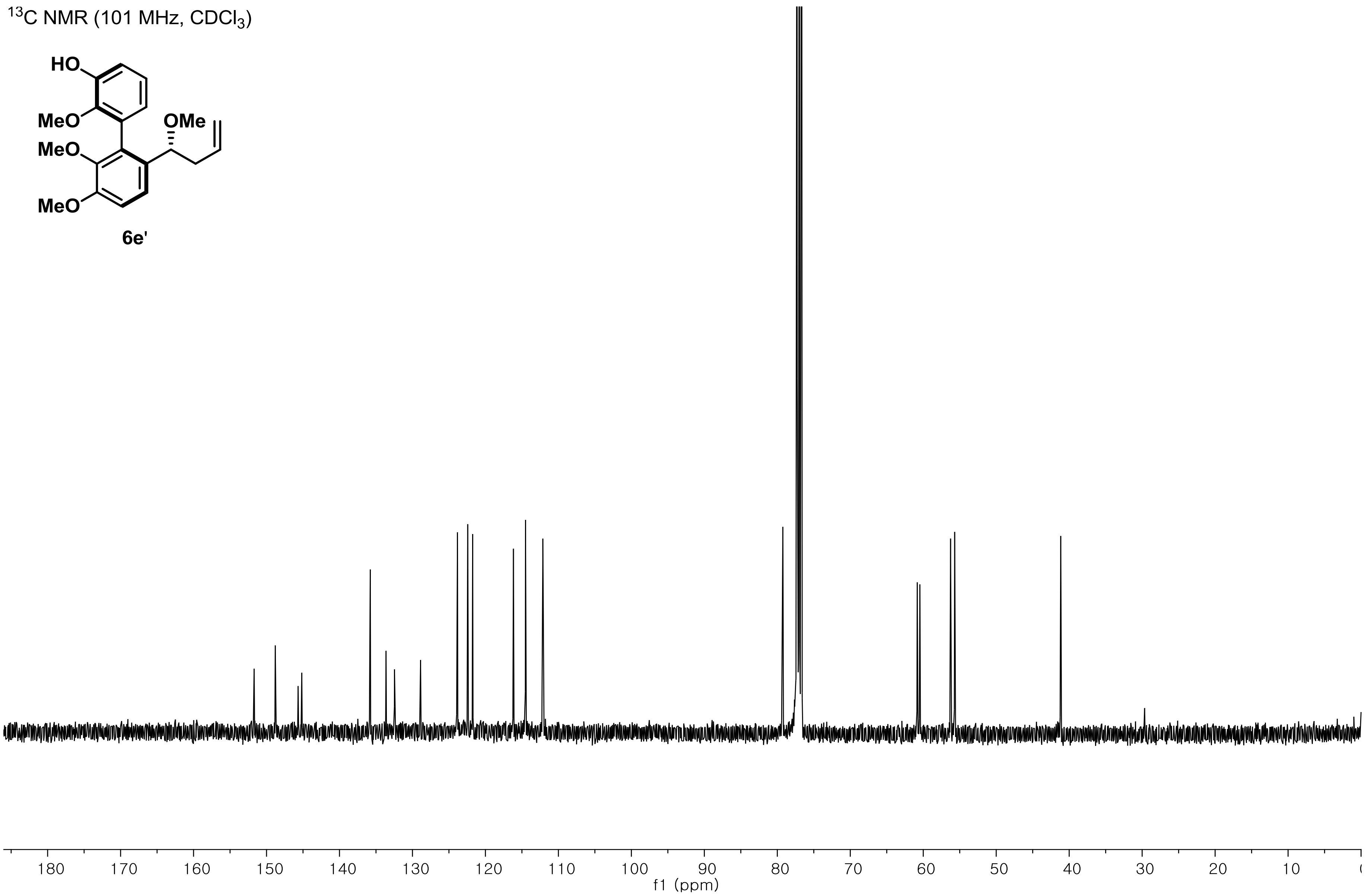

^1H NMR (400 MHz, $CDCl_3$)

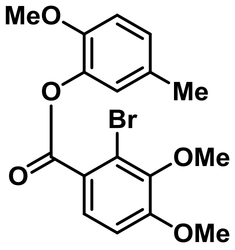

2i

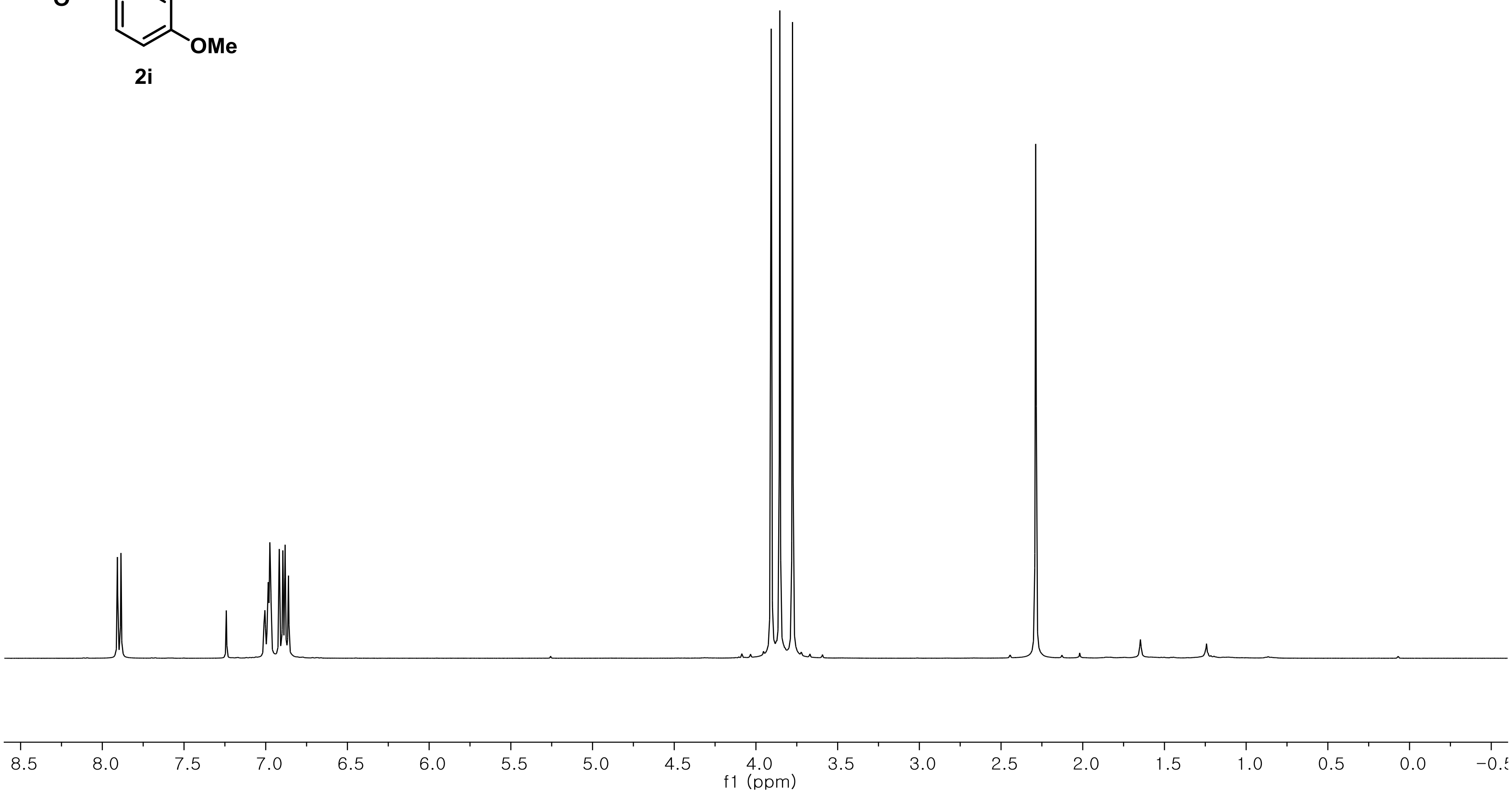

^{13}C NMR (101 MHz, $CDCl_3$)

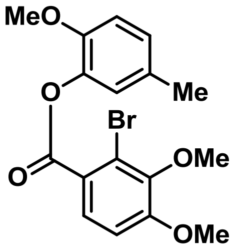

2i

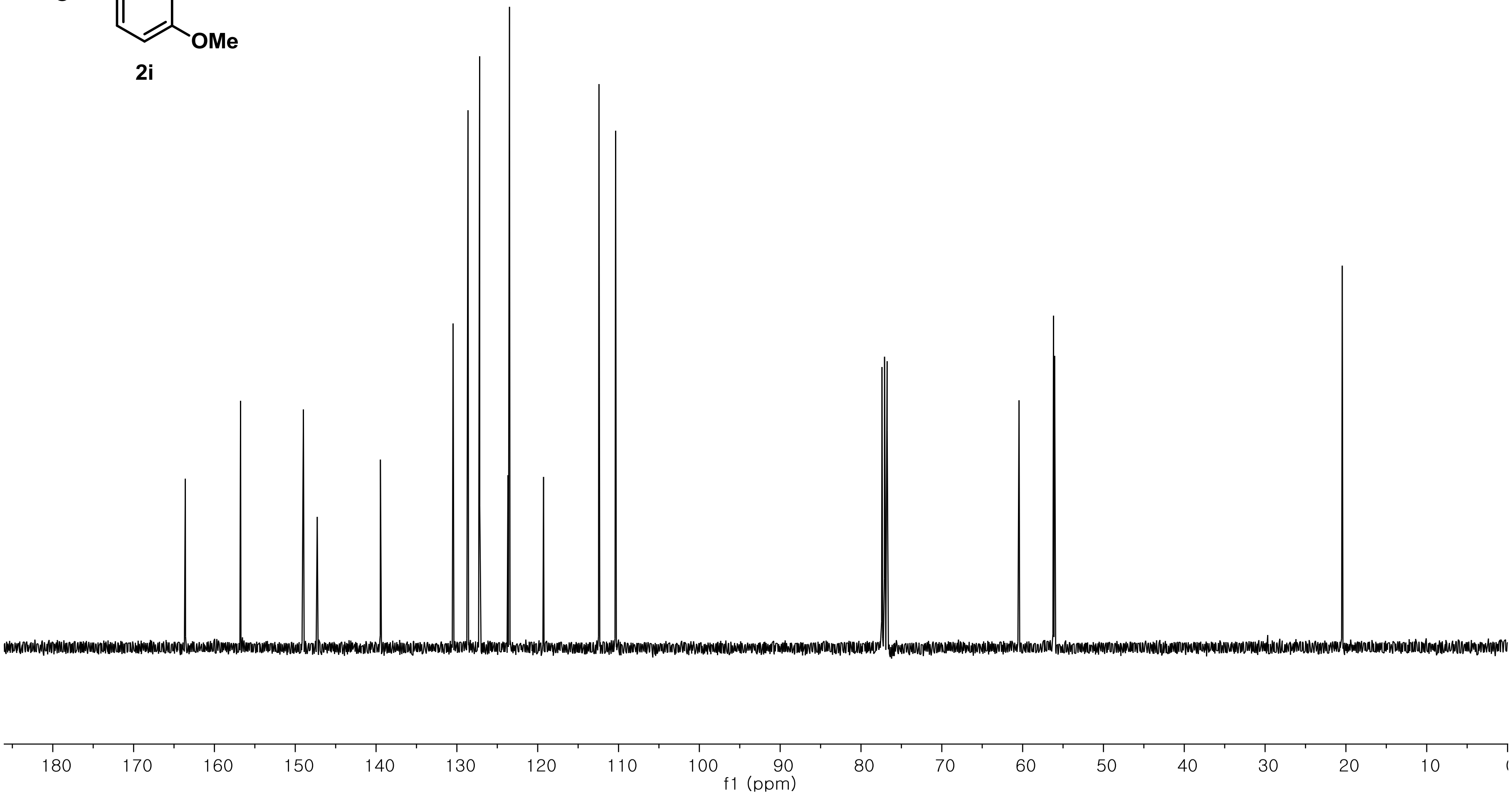

^{1}H NMR (499 MHz, $CDCl_3$)

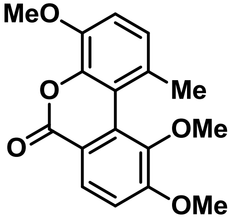

3b

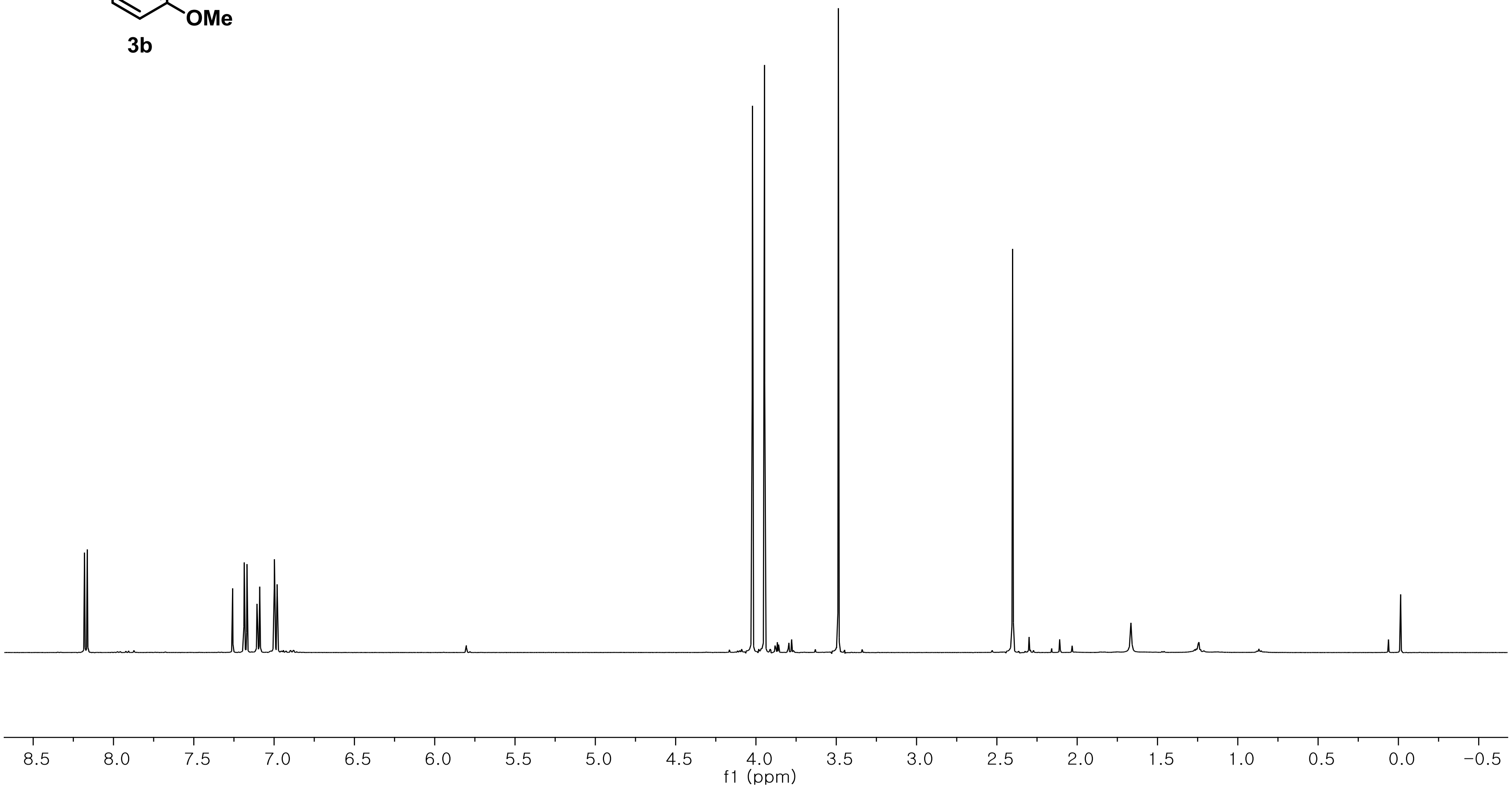

^{13}C NMR (126 MHz, $CDCl_3$)

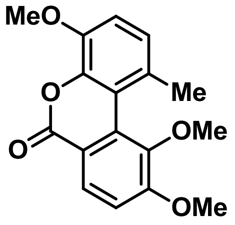

3b

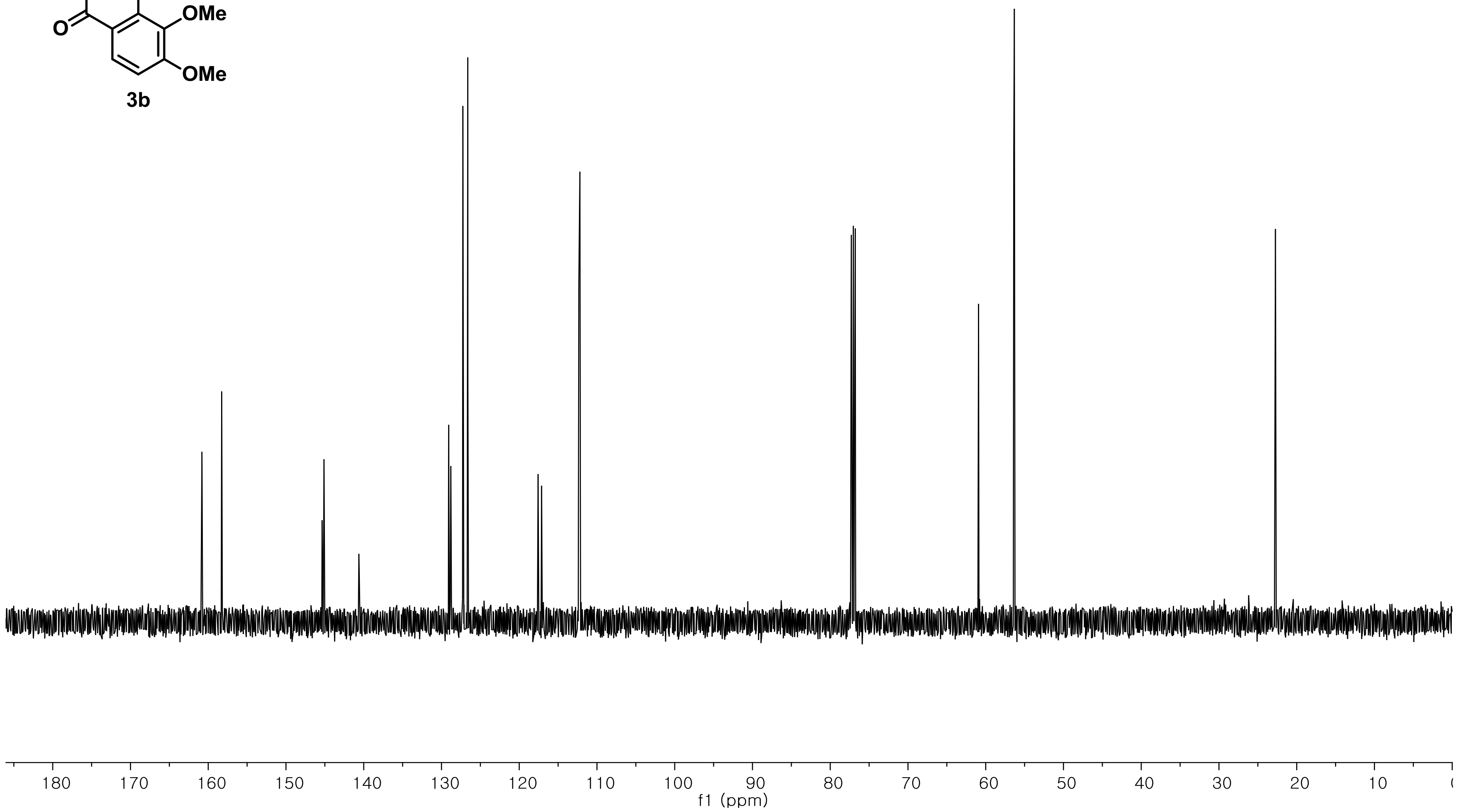

^1H NMR (400 MHz, C_6D_6)

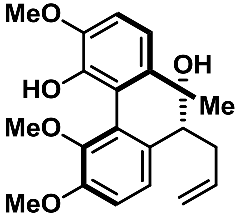

6f

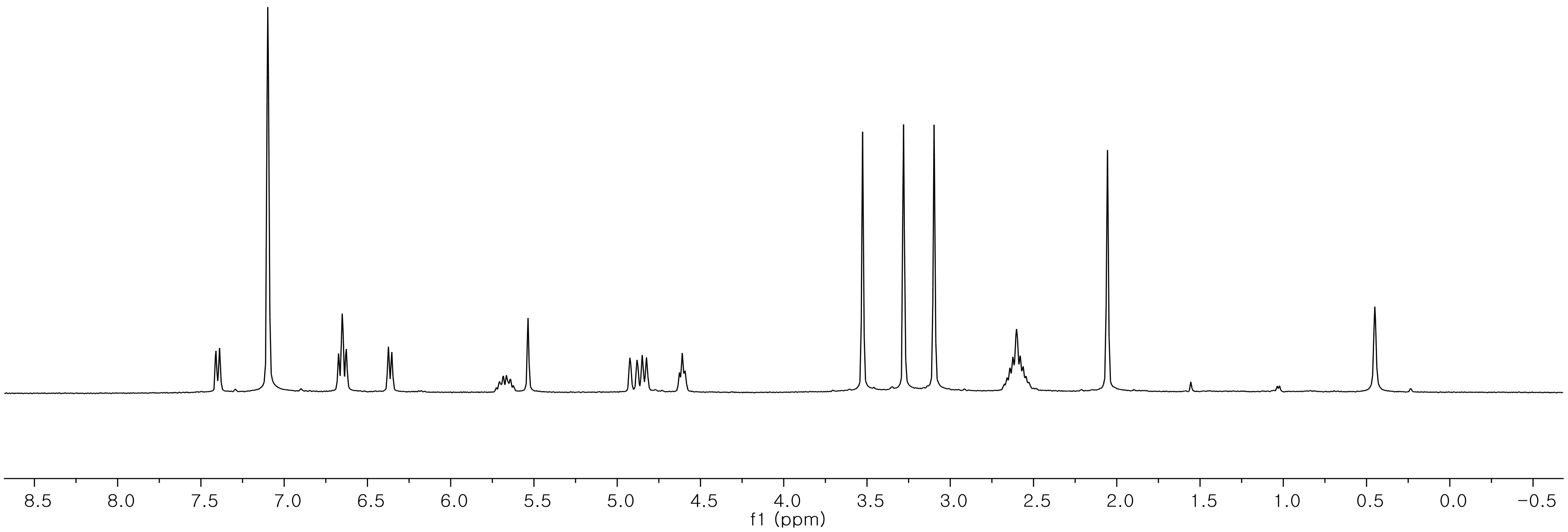

^{13}C NMR (101 MHz, C_6D_6)

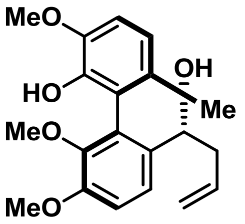

6f

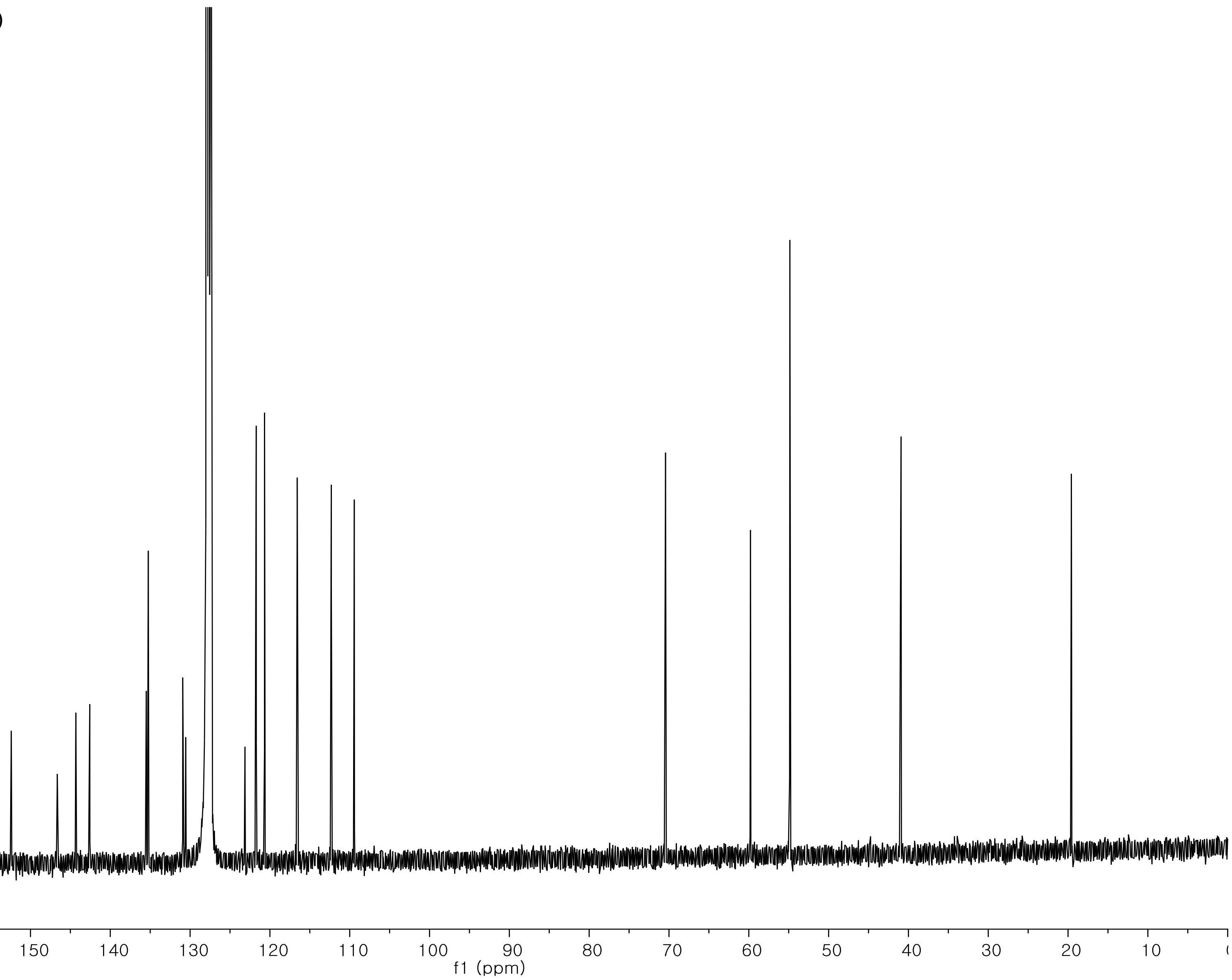

^{1}H NMR (400 MHz, $CDCl_3$)

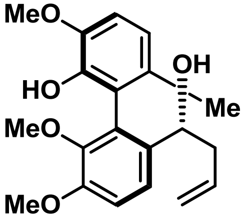

6f'

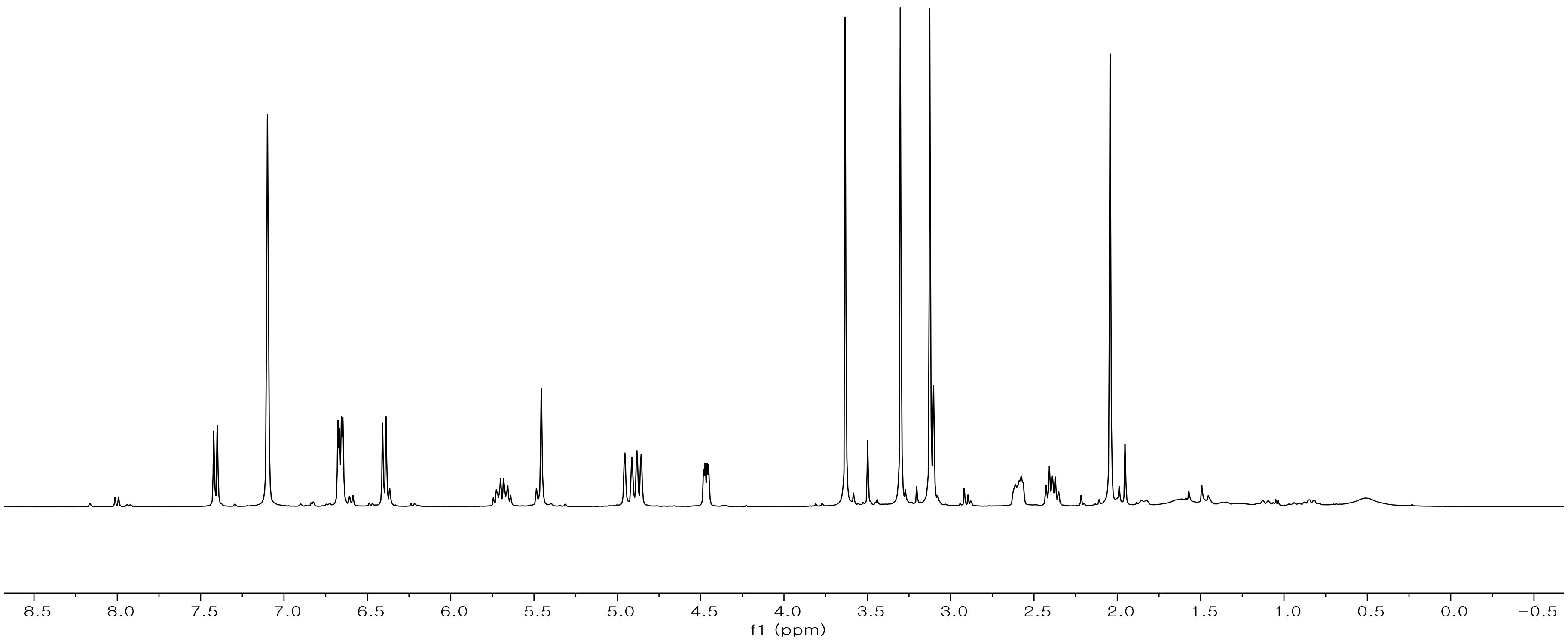

^{13}C NMR (101 MHz, $CDCl_3$)

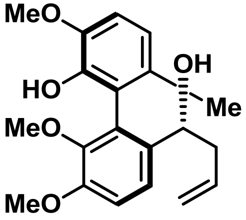

6f'

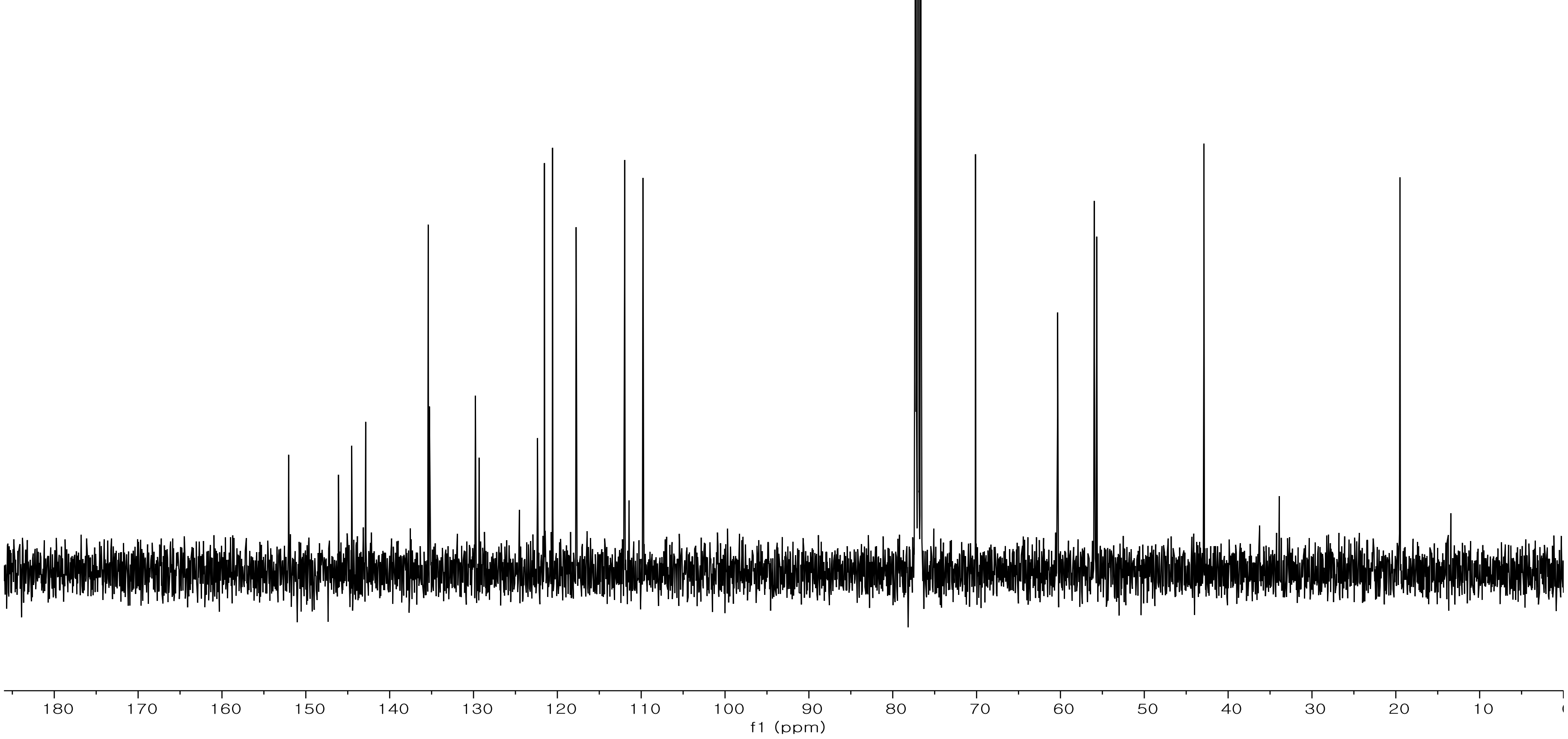

^{1}H NMR (400 MHz, $CDCl_3$)

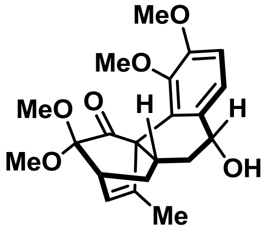

8f

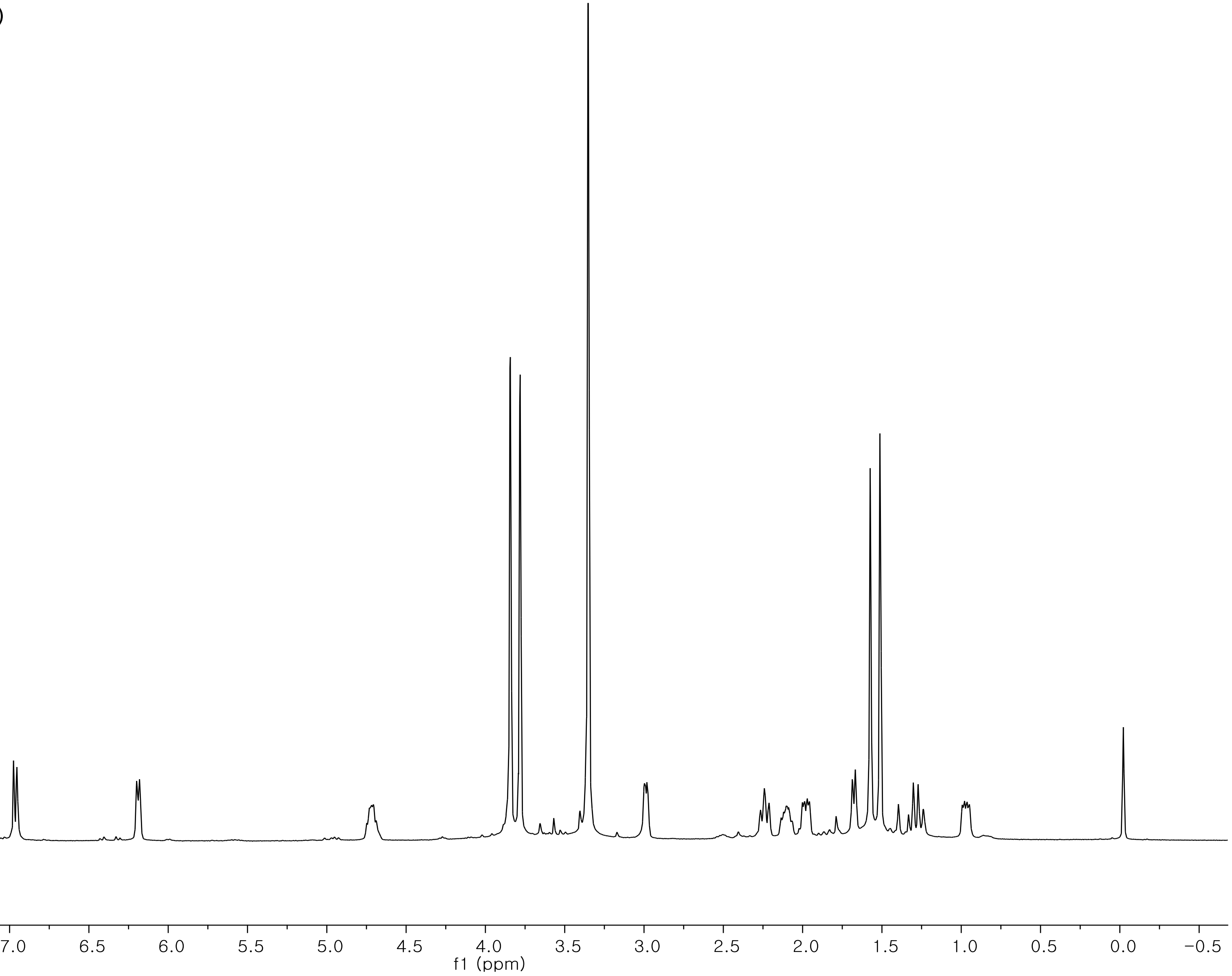

^{13}C NMR (101 MHz, $CDCl_3$)

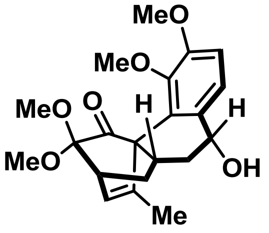

8f

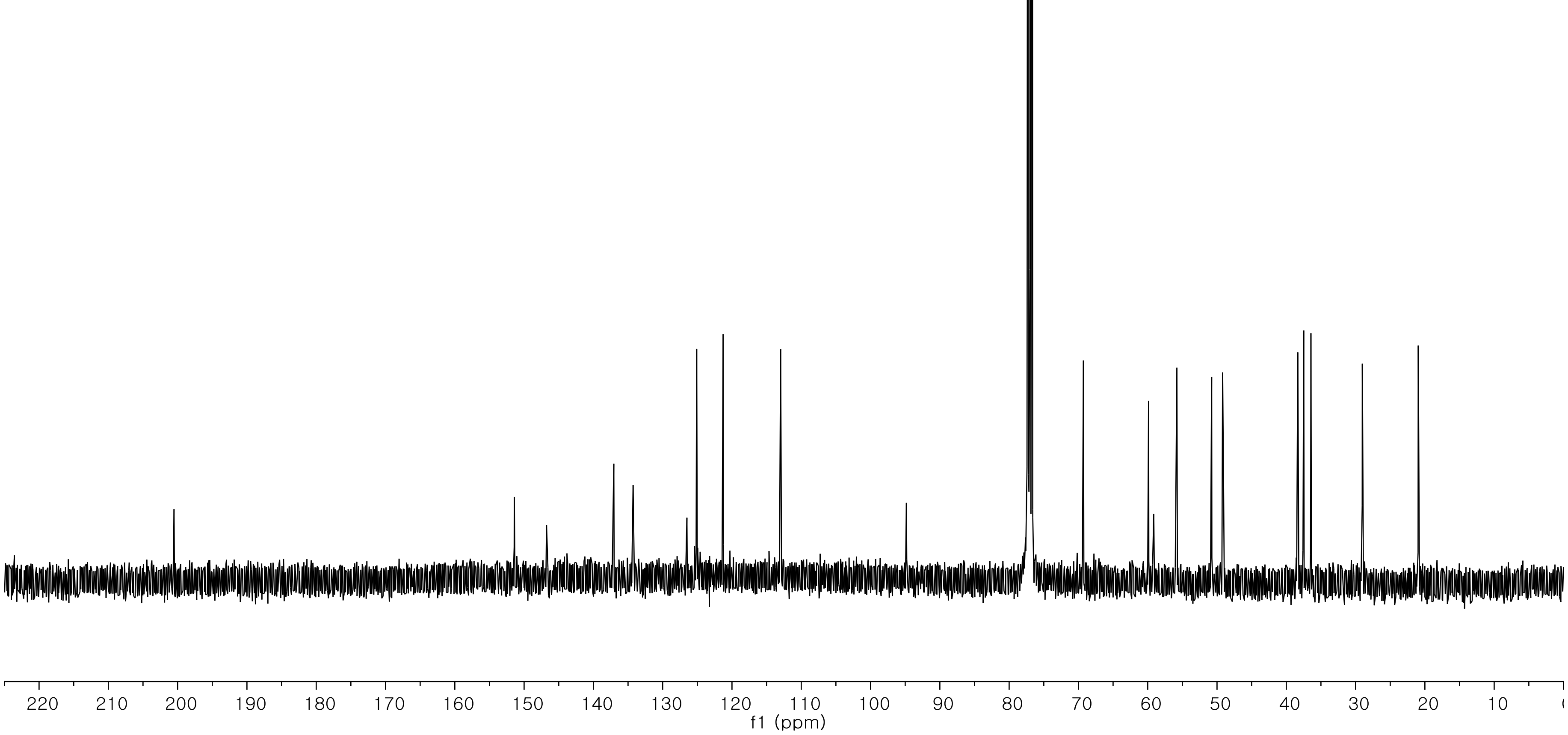

^{1}H NMR (400 MHz, $CDCl_3$)

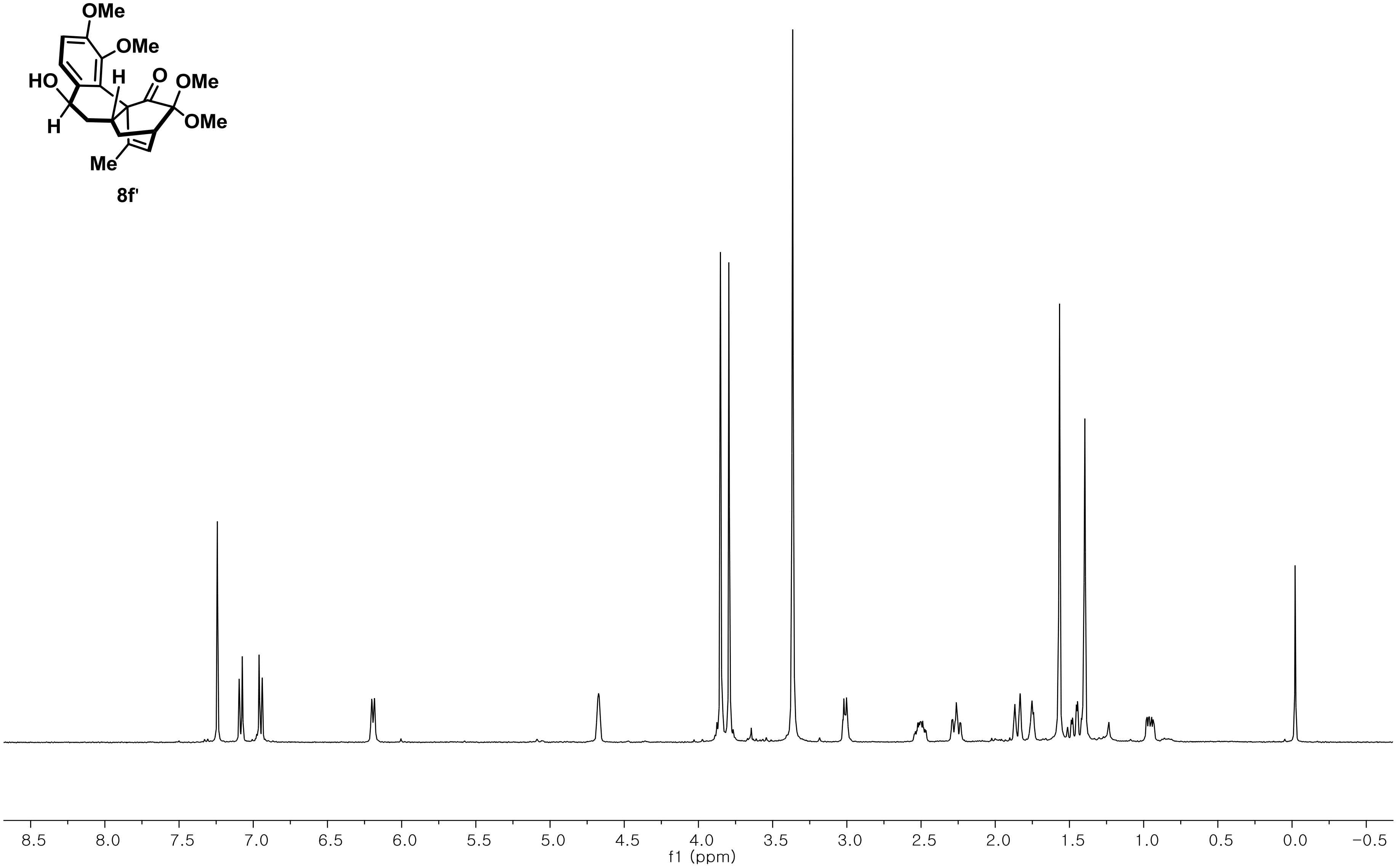

8f'

^{13}C NMR (101 MHz, $CDCl_3$)

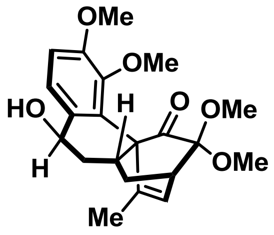

8f'

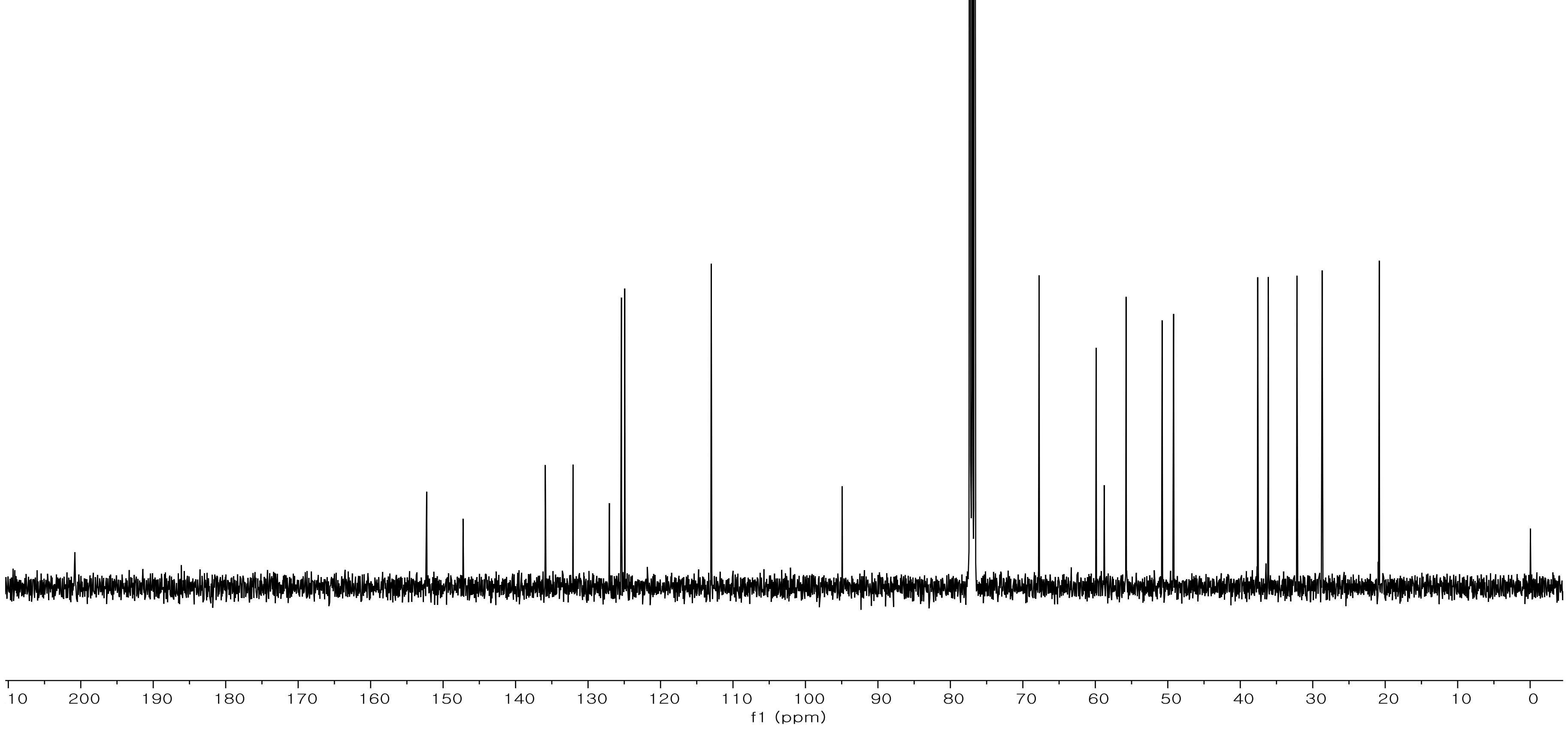

^1H NMR (400 MHz, $CDCl_3$)

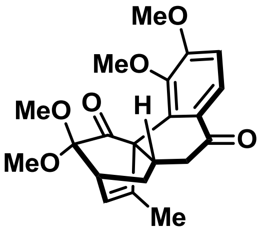

9f

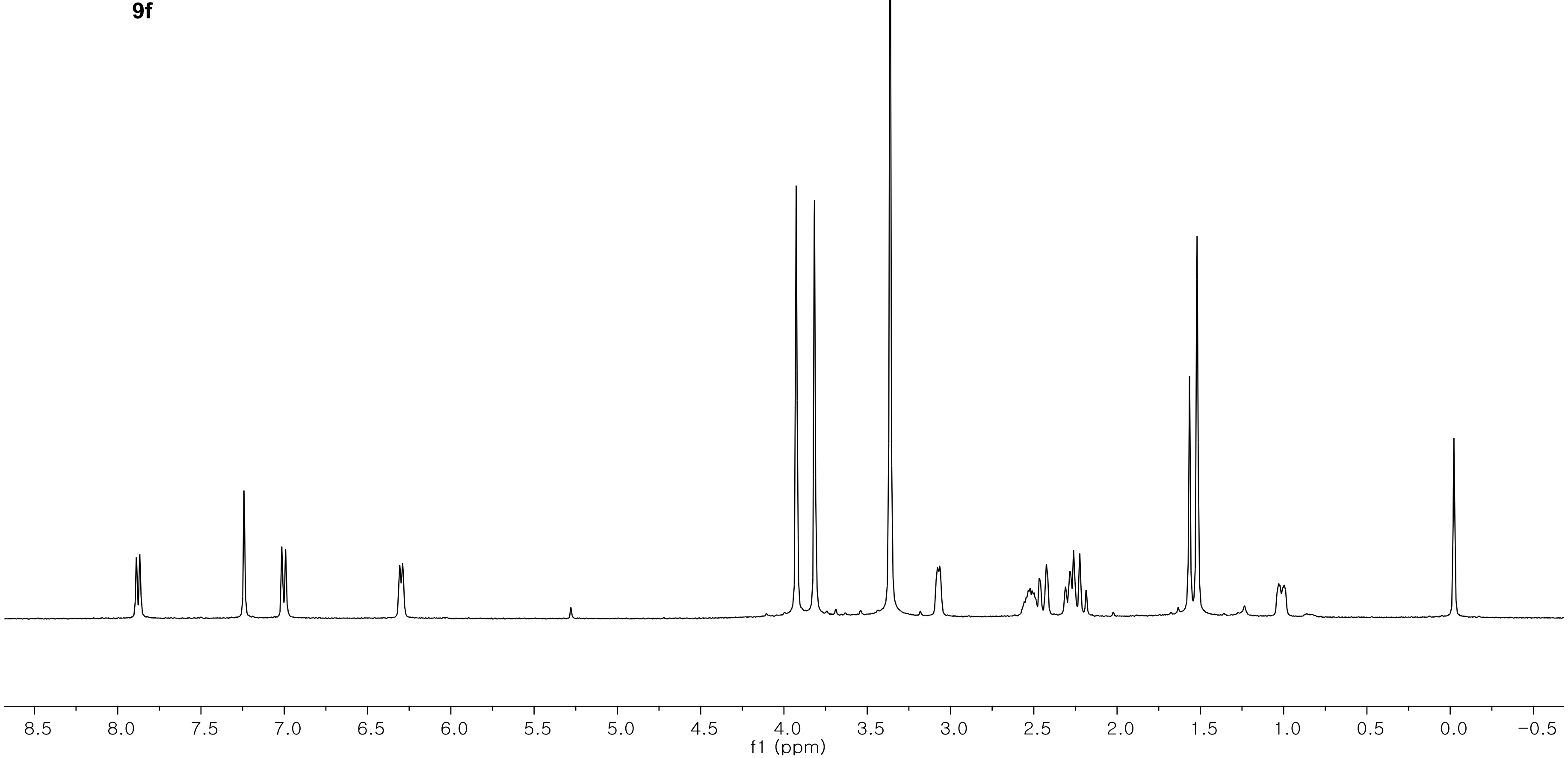

^{13}C NMR (101 MHz, $CDCl_3$)

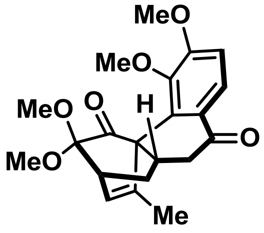

9f

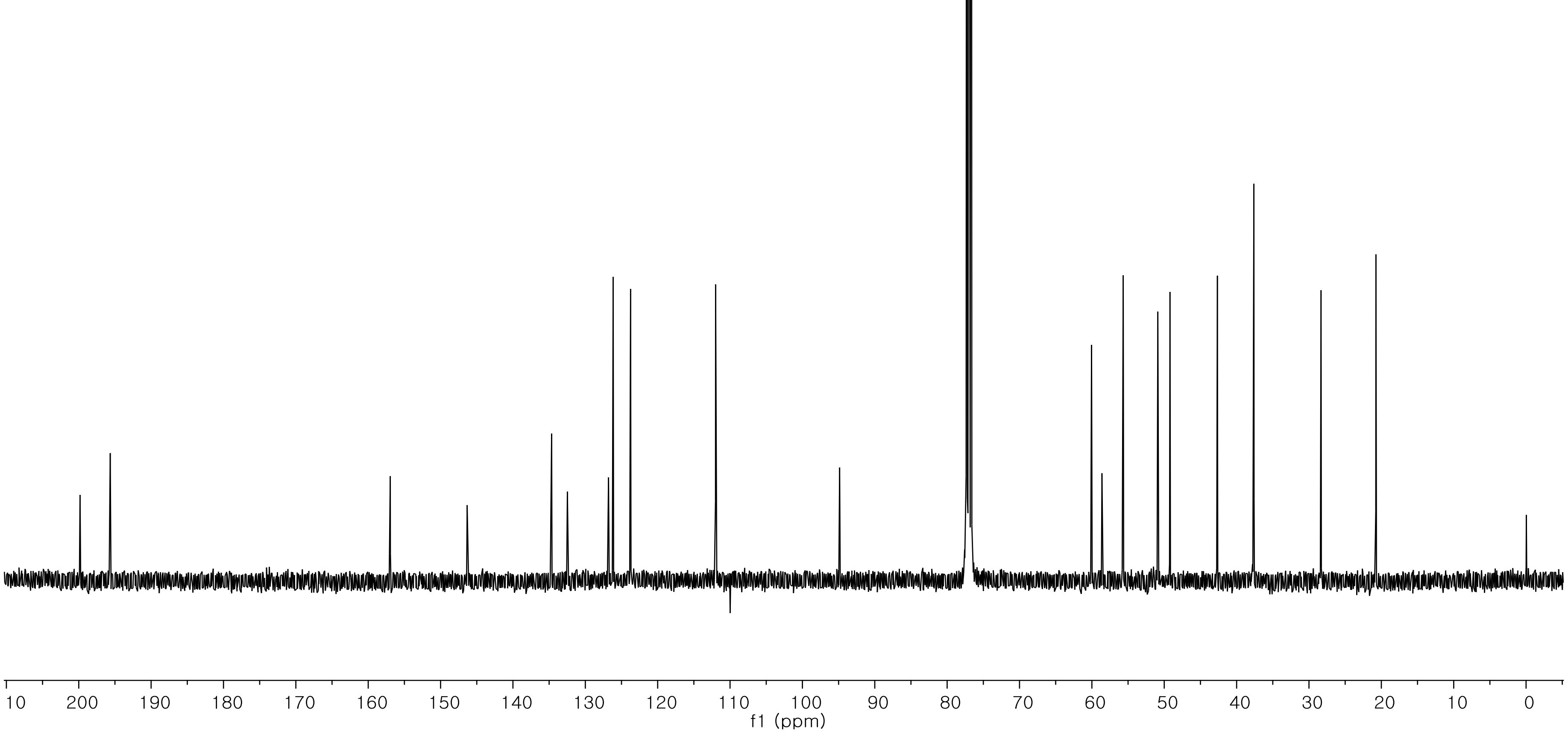

^1H NMR (400 MHz, $CDCl_3$)

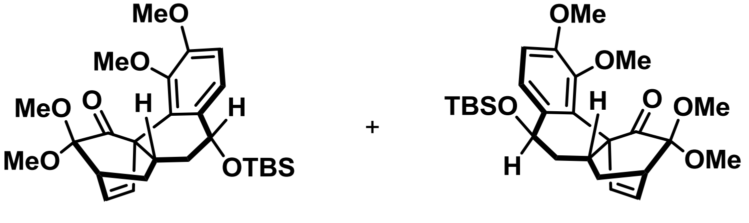

8 8'

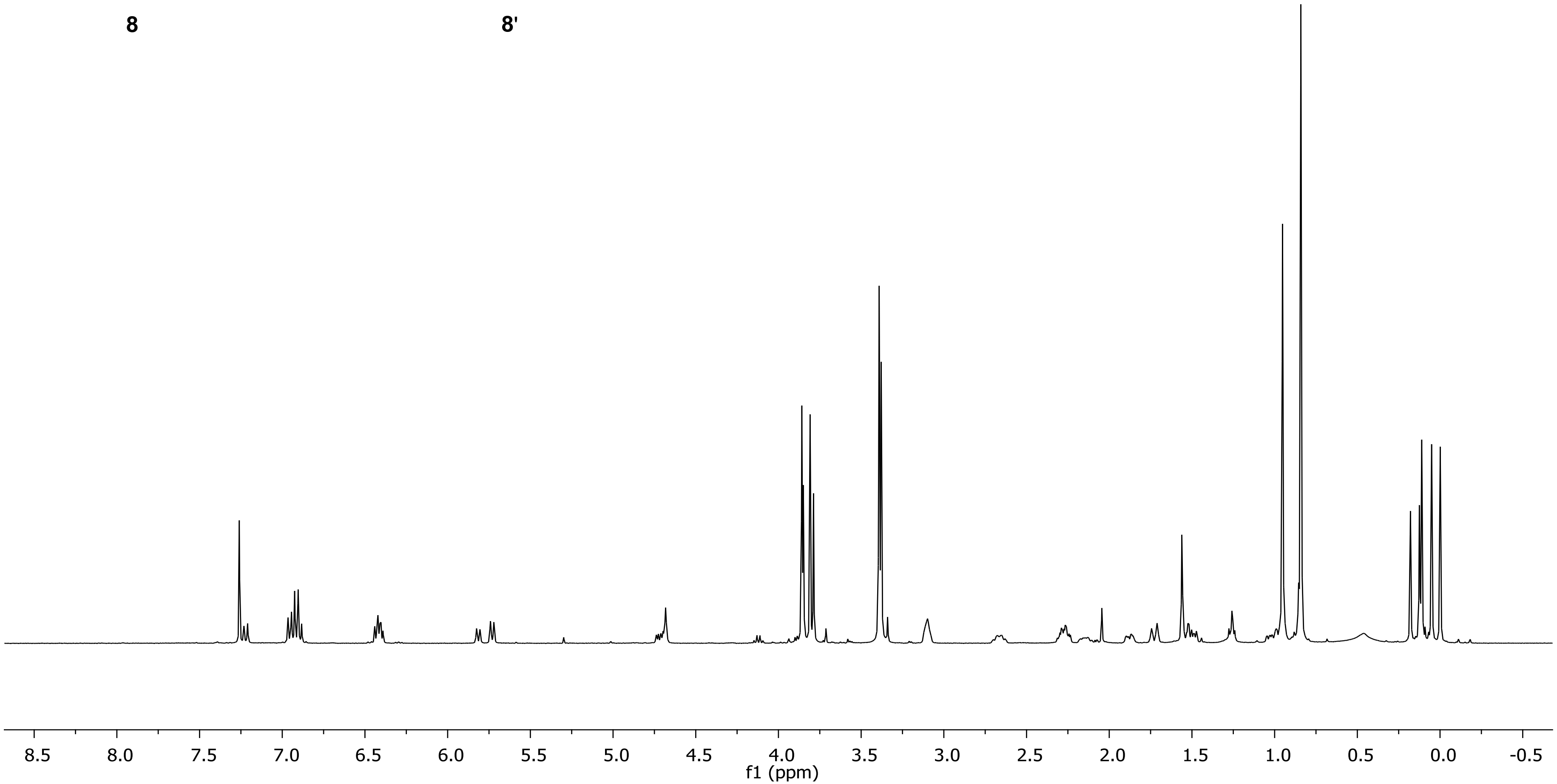

^{13}C NMR (101 MHz, $CDCl_3$)

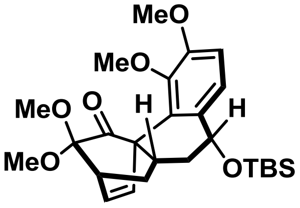

8

+

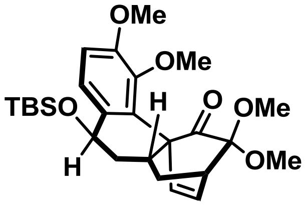

8'

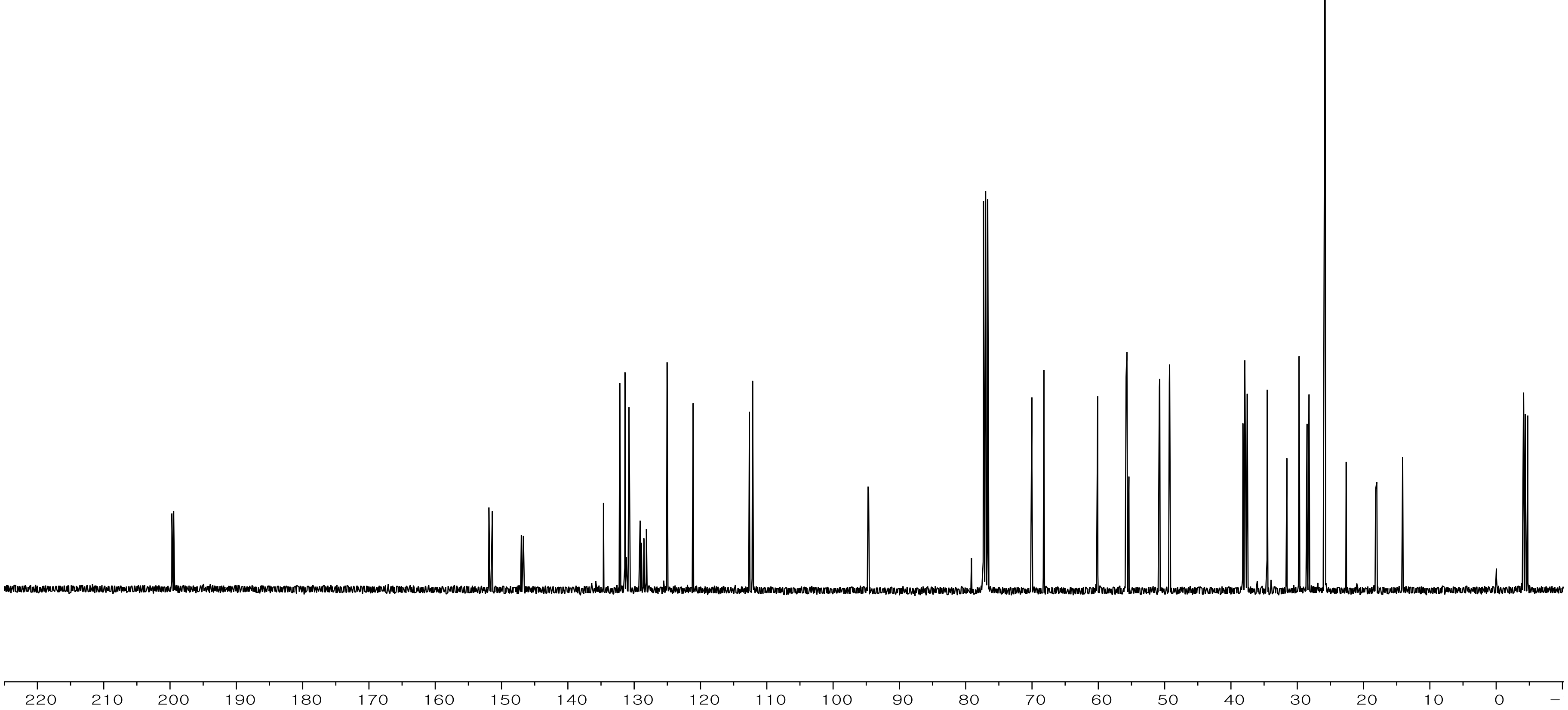

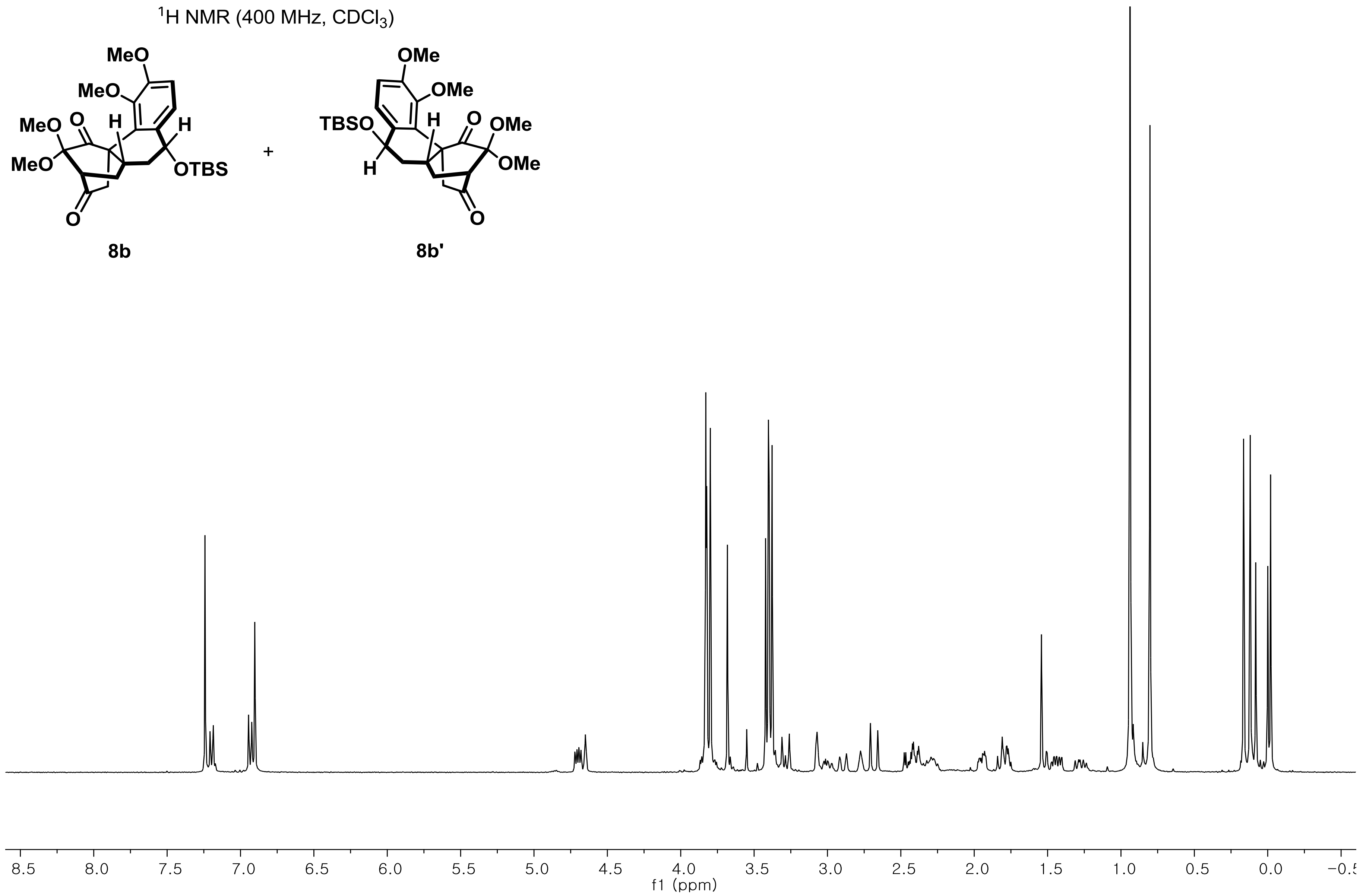

1H NMR (400 MHz, CDCl3)
MeO
MeO
MeO
MeO
O
O
H
H
OTBS
8b
+
OMe
OMe
TBSO
H
H
O
OMe
OMe
O
8b'
8.5 8.0 7.5 7.0 6.5 6.0 5.5 5.0 4.5 4.0 3.5 3.0 2.5 2.0 1.5 1.0 0.5 0.0
f1 (ppm)

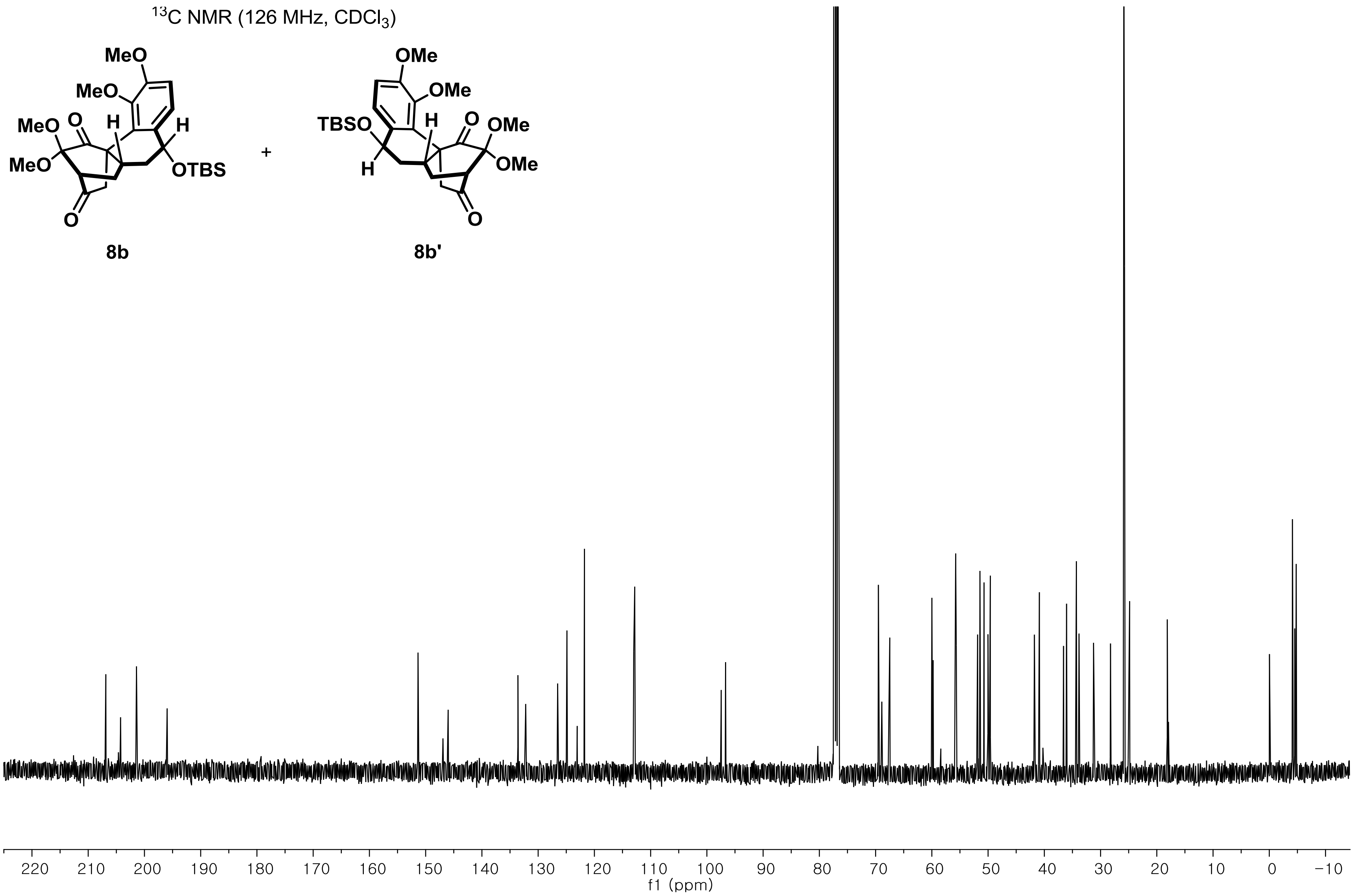

^{13}C NMR (126 MHz, CDCl$_3$)
MeO
MeO
MeO
MeO
O
O
H
H
OTBS
8b
+
OMe
OMe
TBSO
H
H
O
OMe
OMe
O
8b'
220
210
200
190
180
170
160
150
140
130
120
110
100
90
80
70
60
50
40
30
20
10
0
−10
f1 (ppm)

^{1}H NMR (499 MHz, $CDCl_3$)

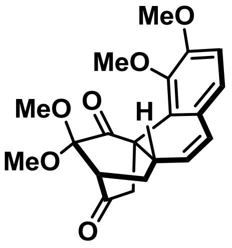

9

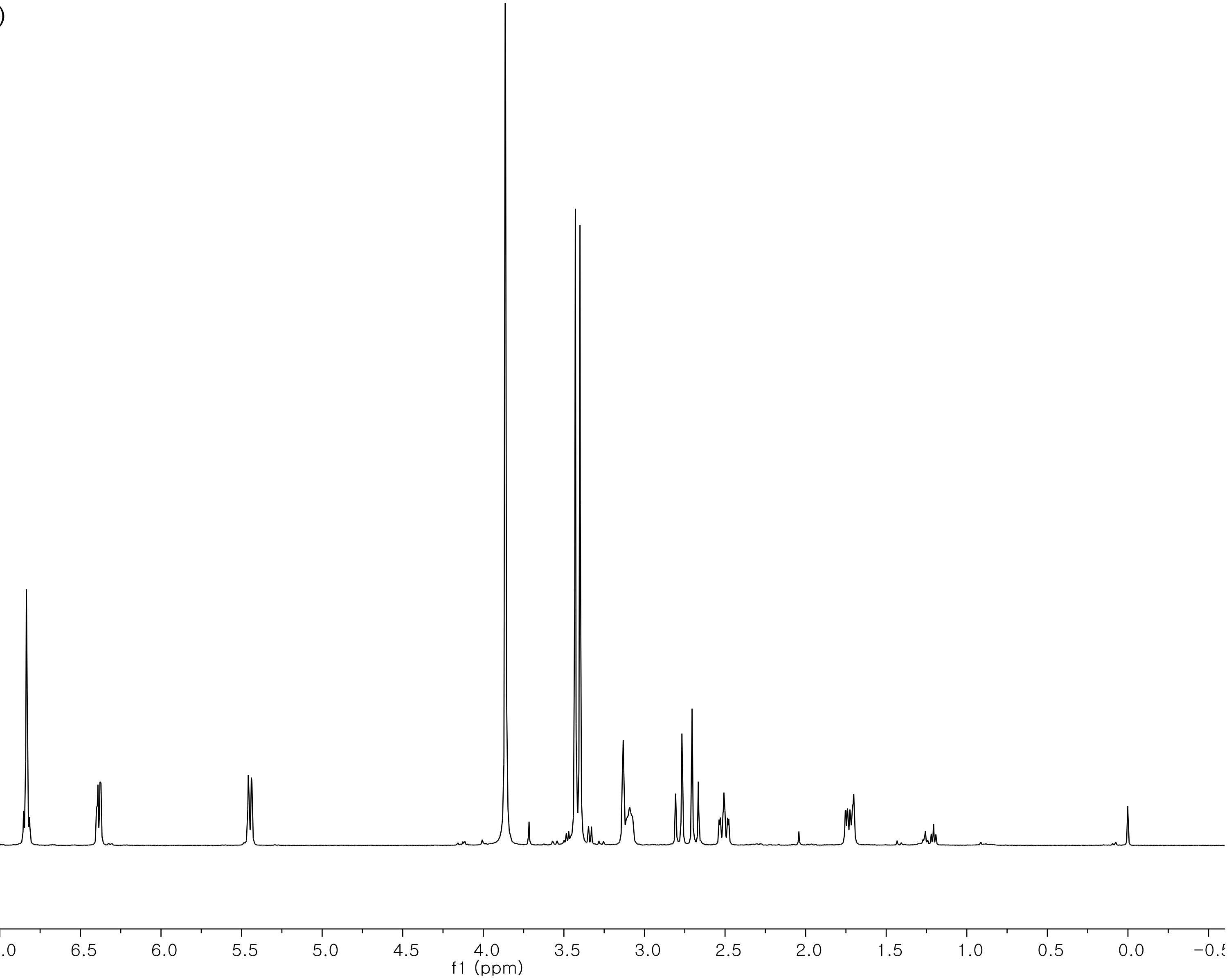

^{13}C NMR (101 MHz, $CDCl_3$)

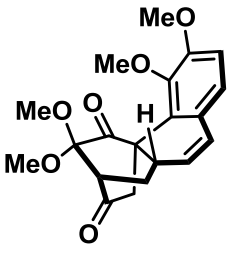

9

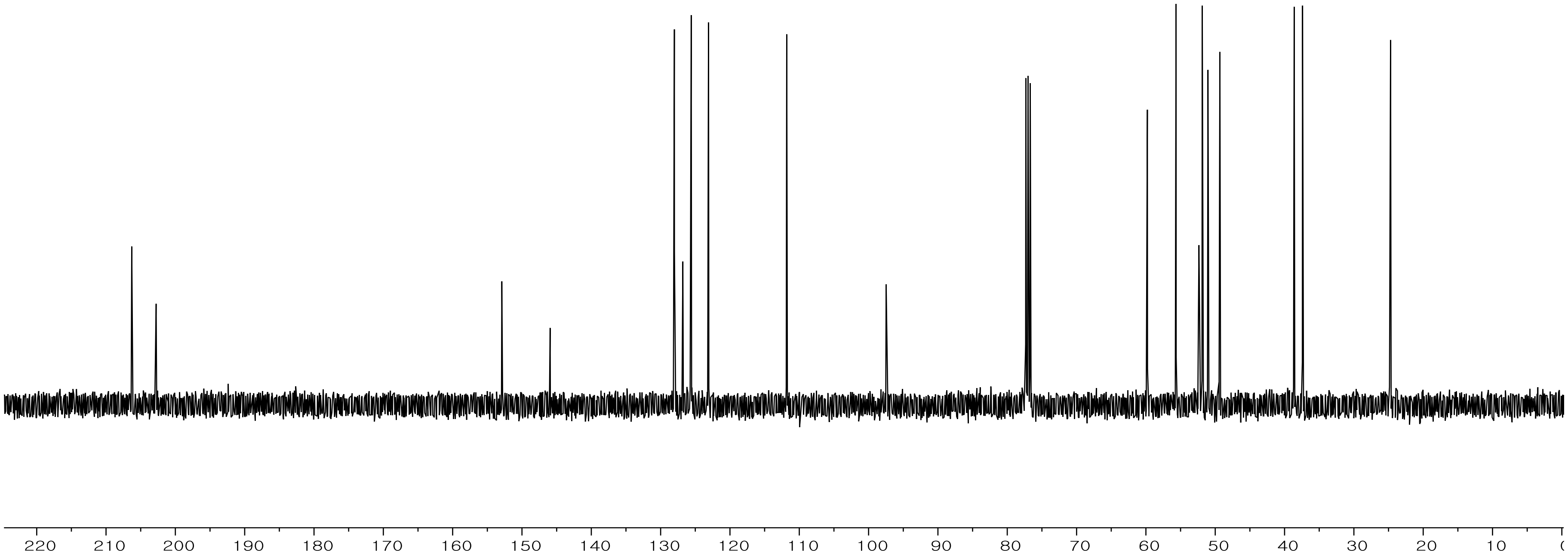

^{1}H NMR (400 MHz, $CDCl_3$)

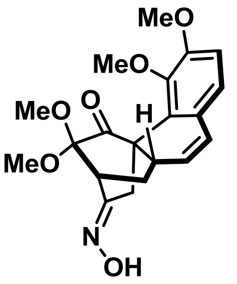

10

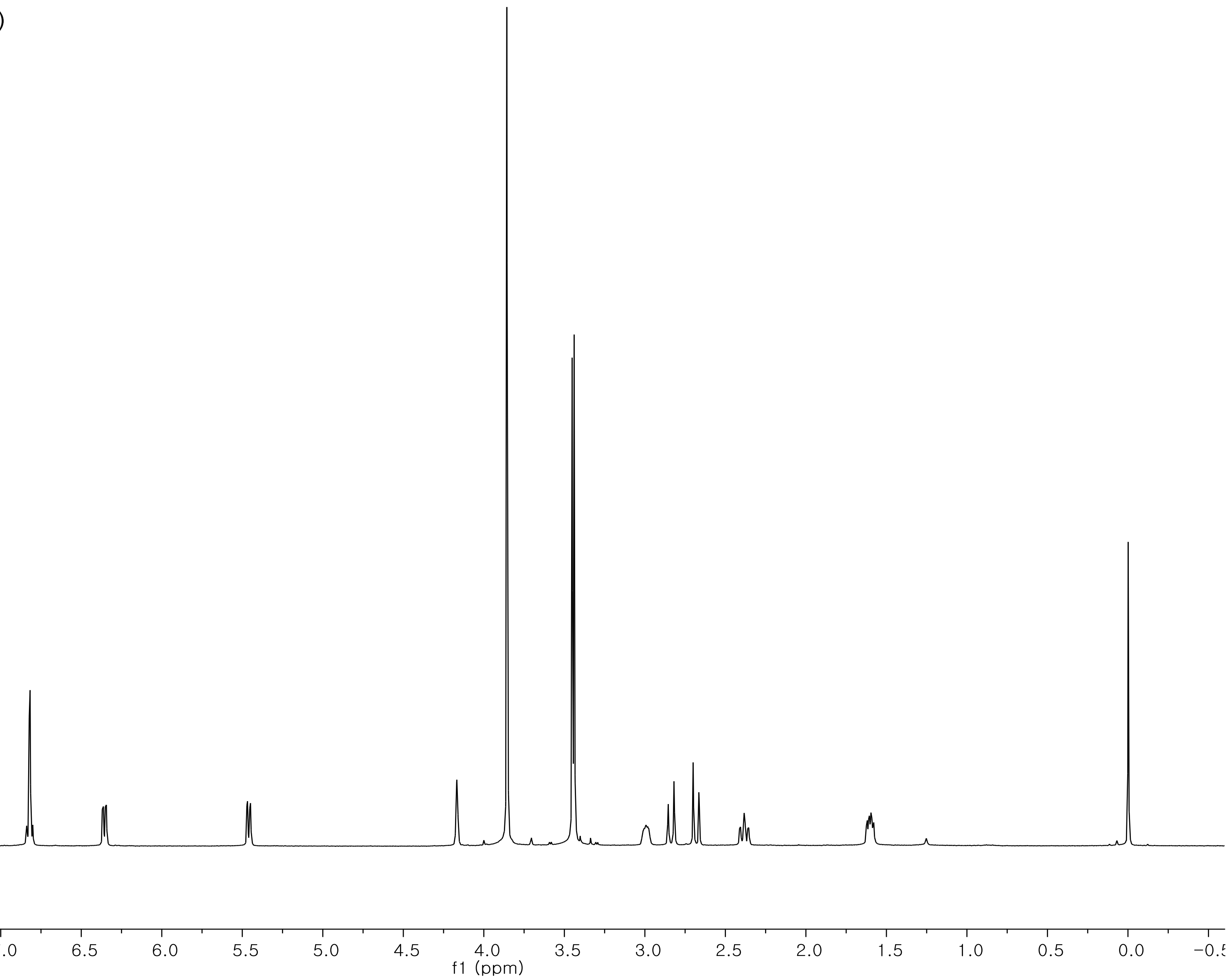

^{13}C NMR (101 MHz, $CDCl_3$)

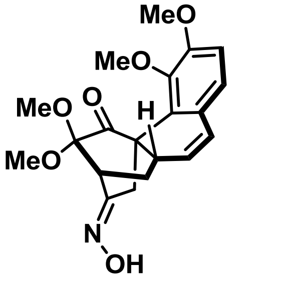

10

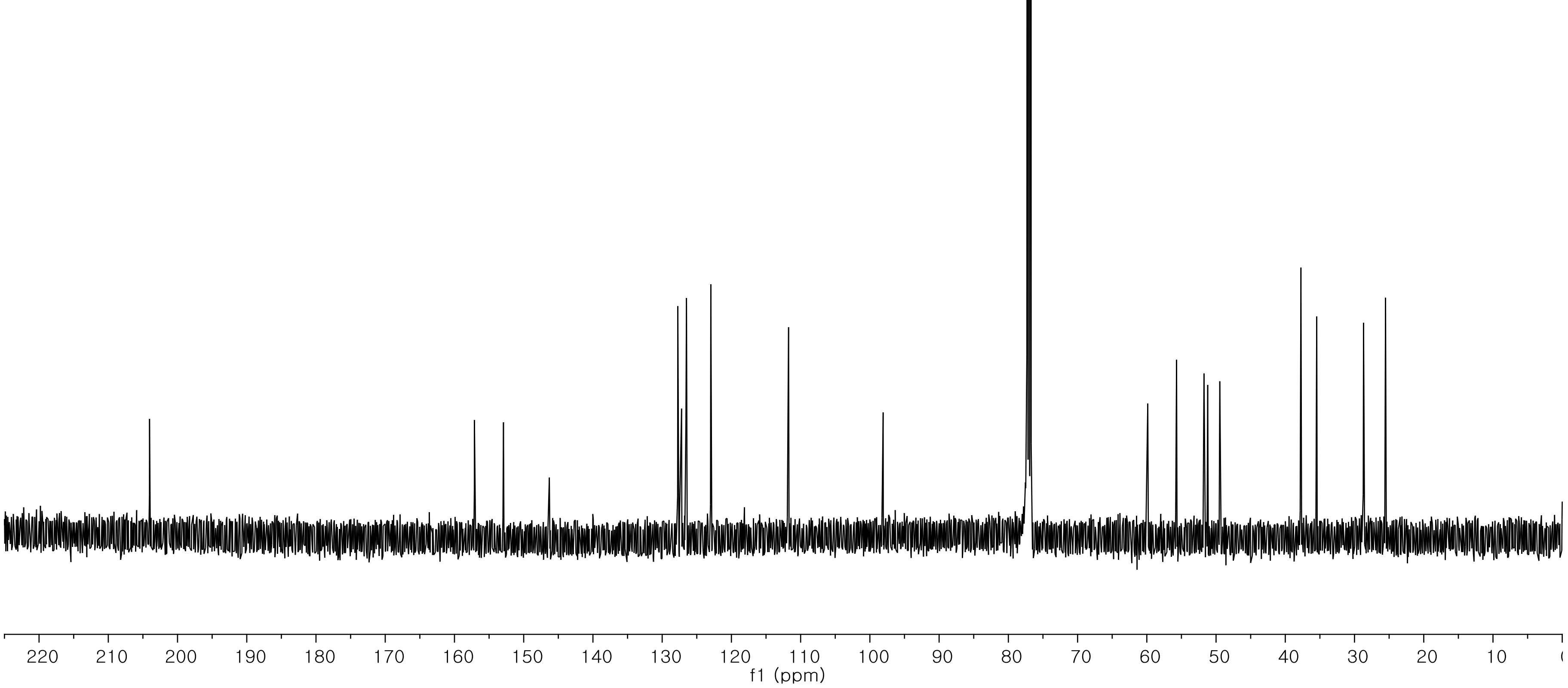

^{1}H NMR (400 MHz, $CDCl_3$)

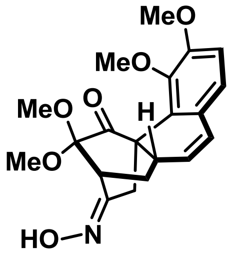

10'

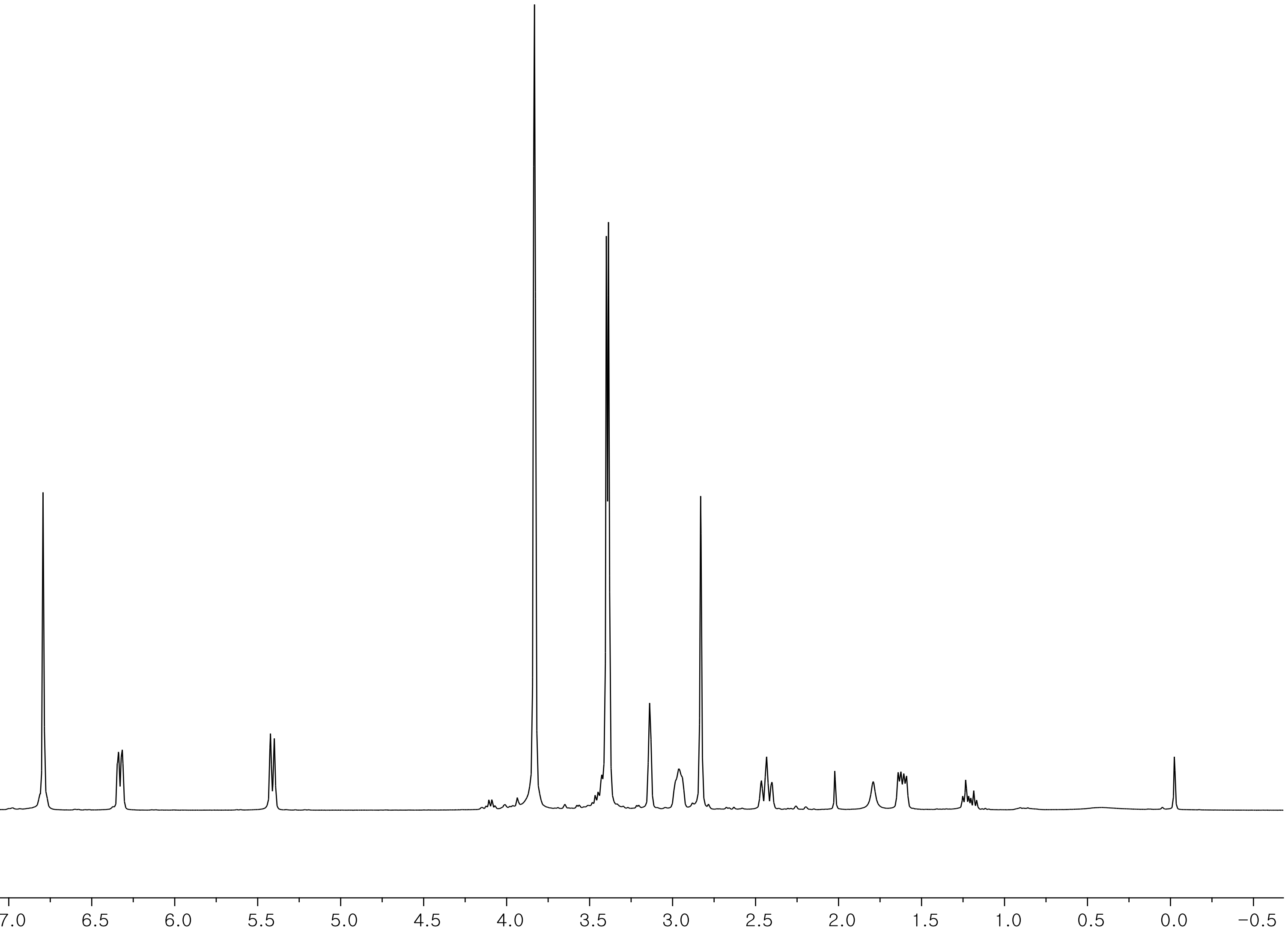

^{13}C NMR (101 MHz, $CDCl_3$)

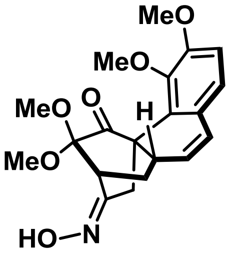

10'

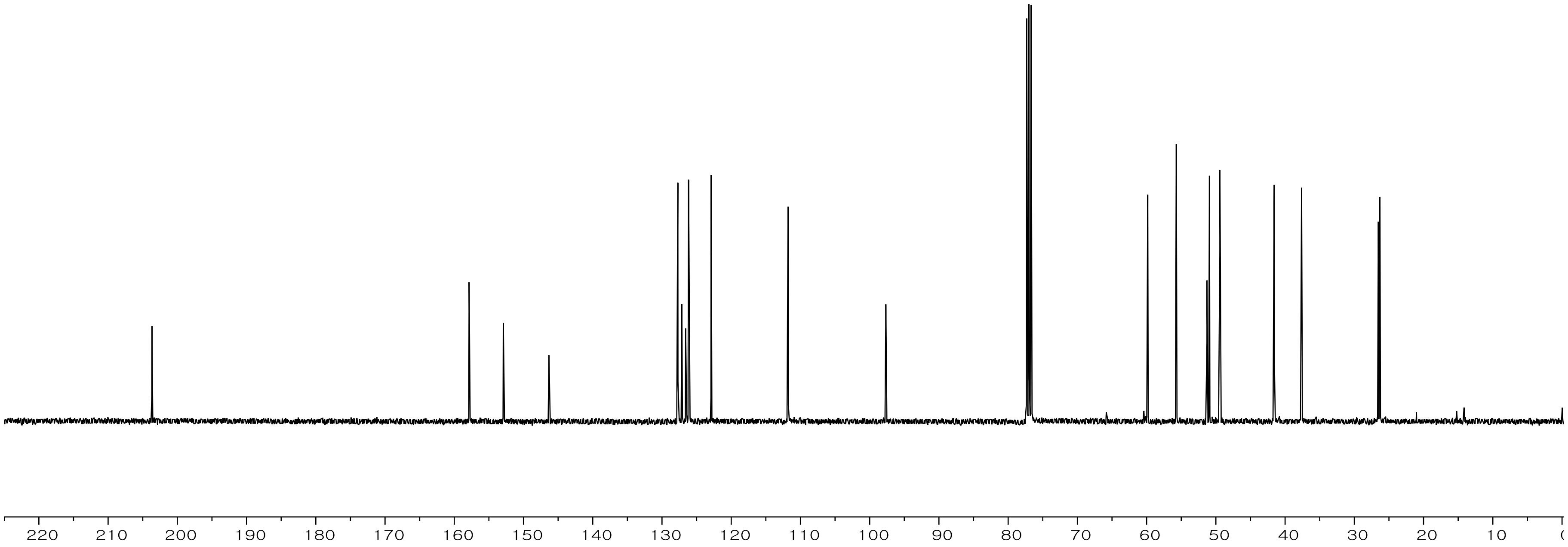

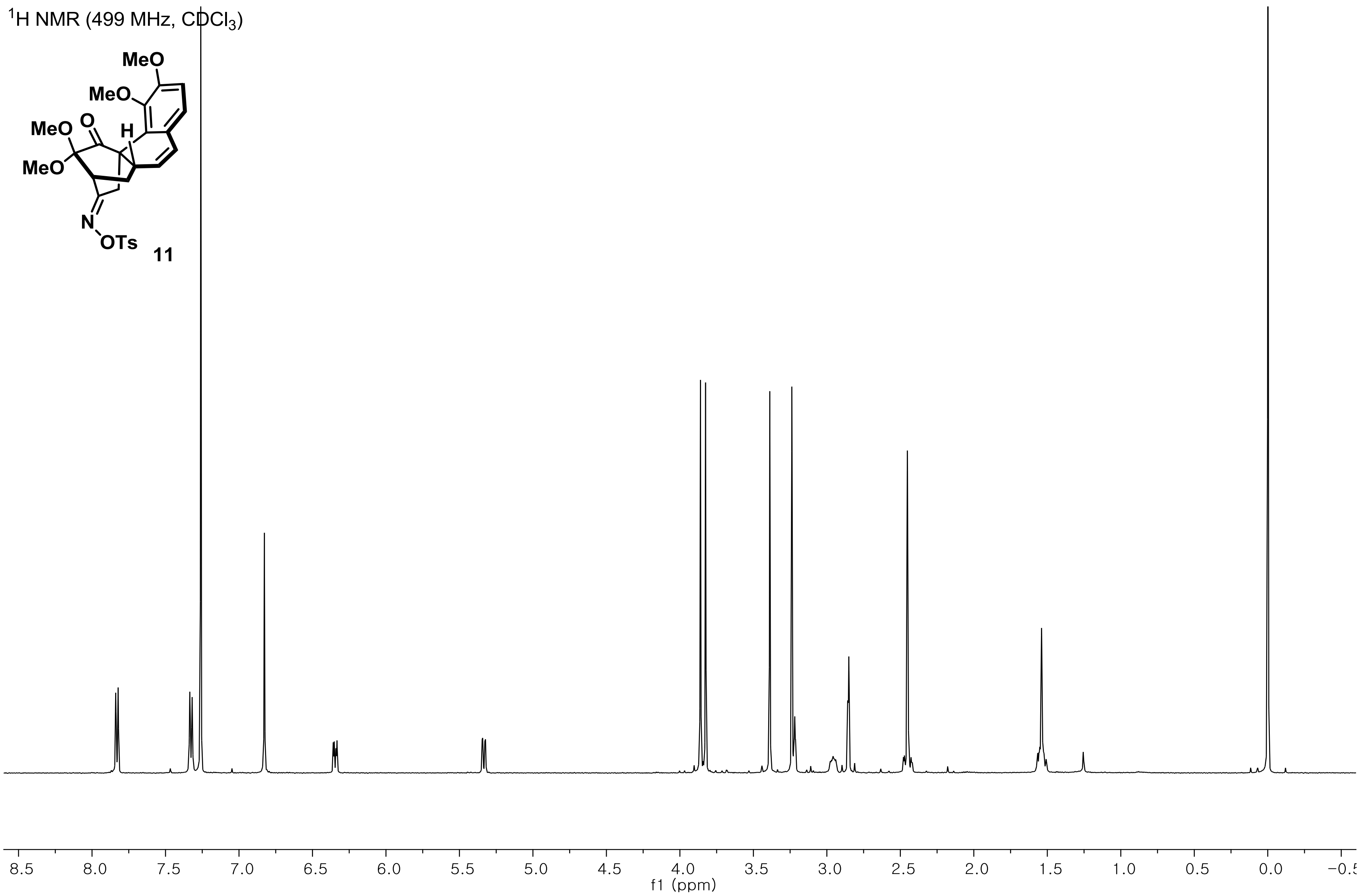

^{1}H NMR (499 MHz, CDCl$_3$)
MeO
MeO
MeO
O
H
MeO
N
OTs
11
8.5
8.0
7.5
7.0
6.5
6.0
5.5
5.0
4.5
4.0
3.5
3.0
2.5
2.0
1.5
1.0
0.5
0.0
f1 (ppm)

^{13}C NMR (101 MHz, $CDCl_3$)

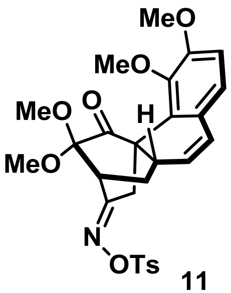

11

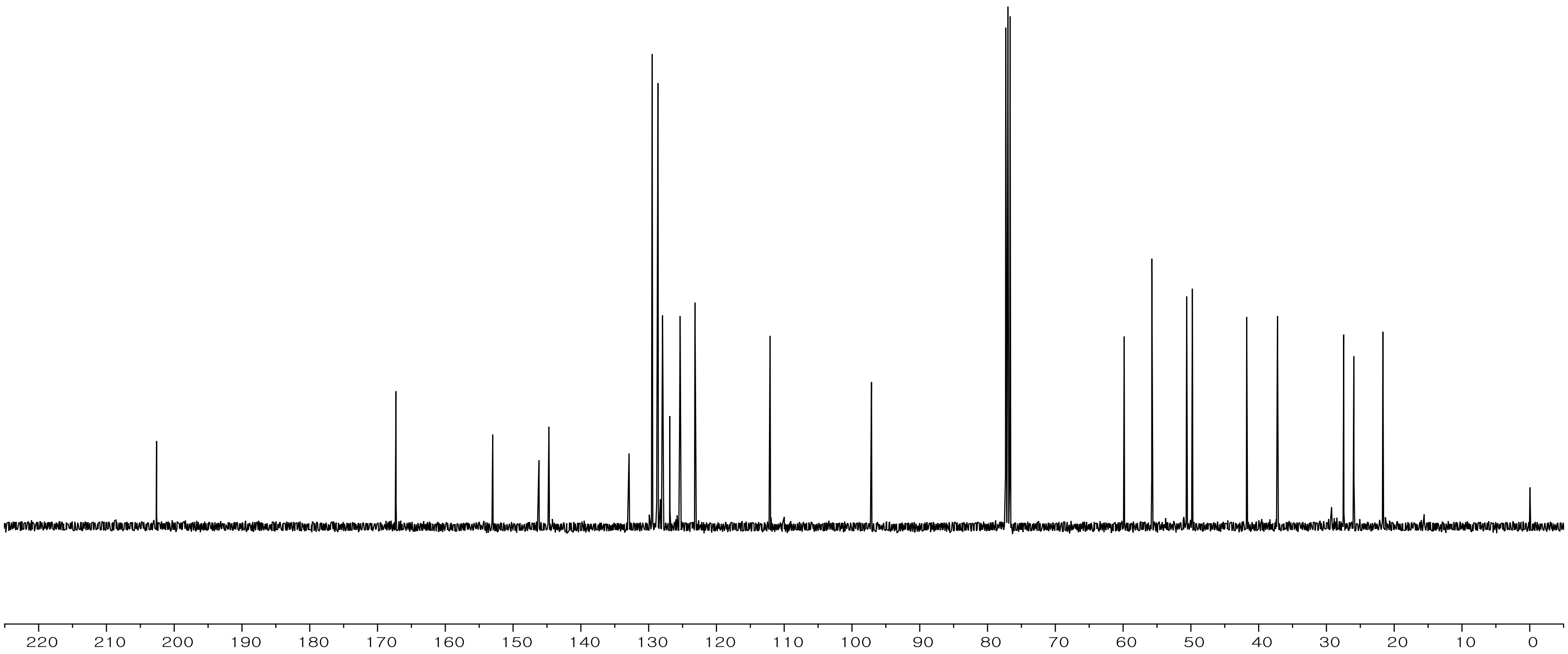

^{1}H NMR (400 MHz, $CDCl_3$)

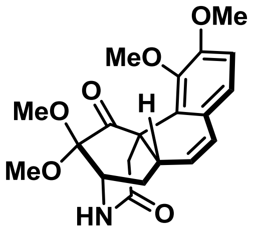

12

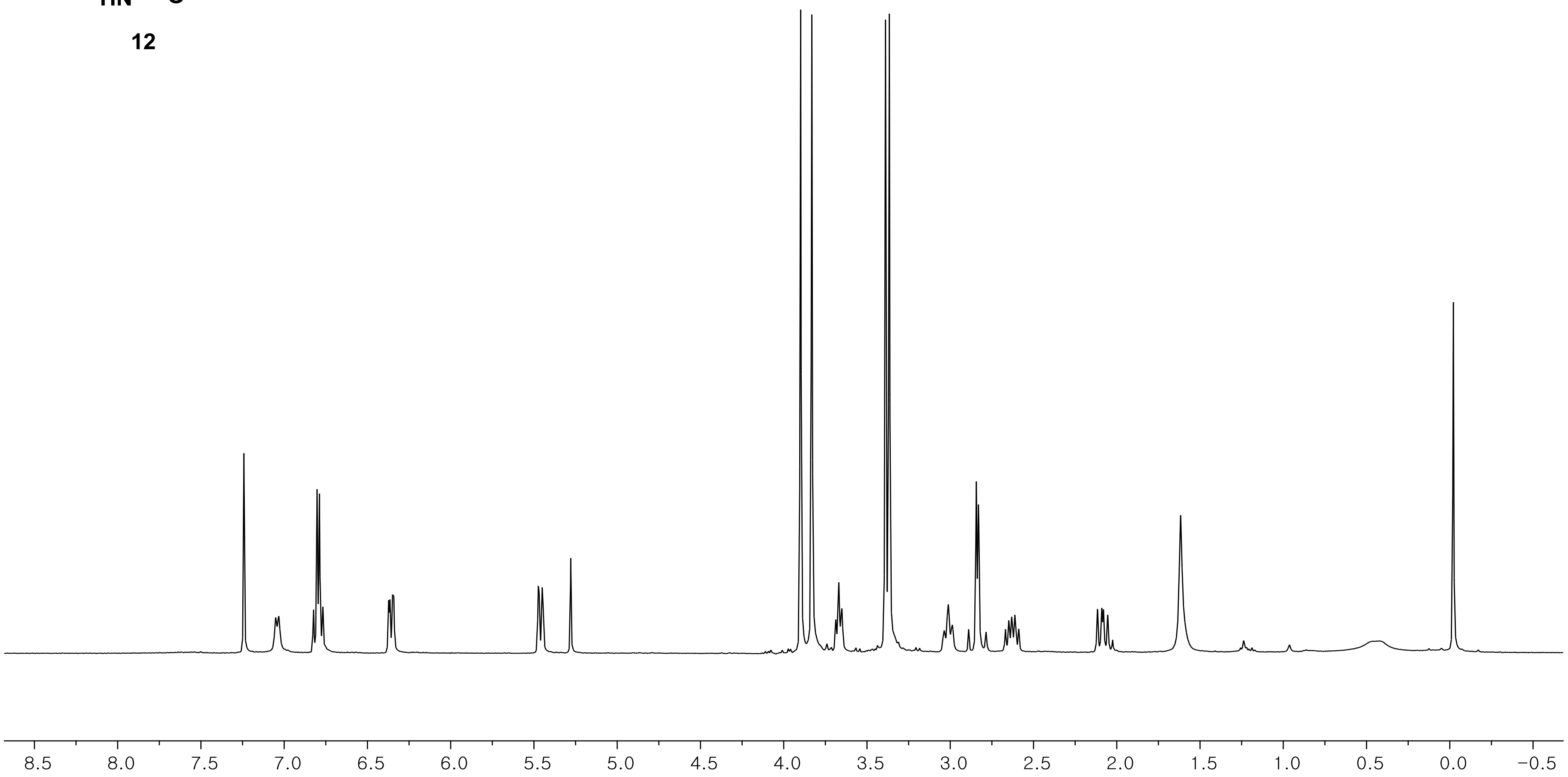

^{13}C NMR (126 MHz, $CDCl_3$)

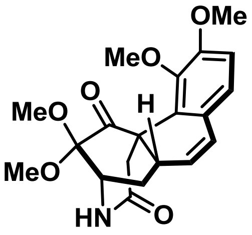

12

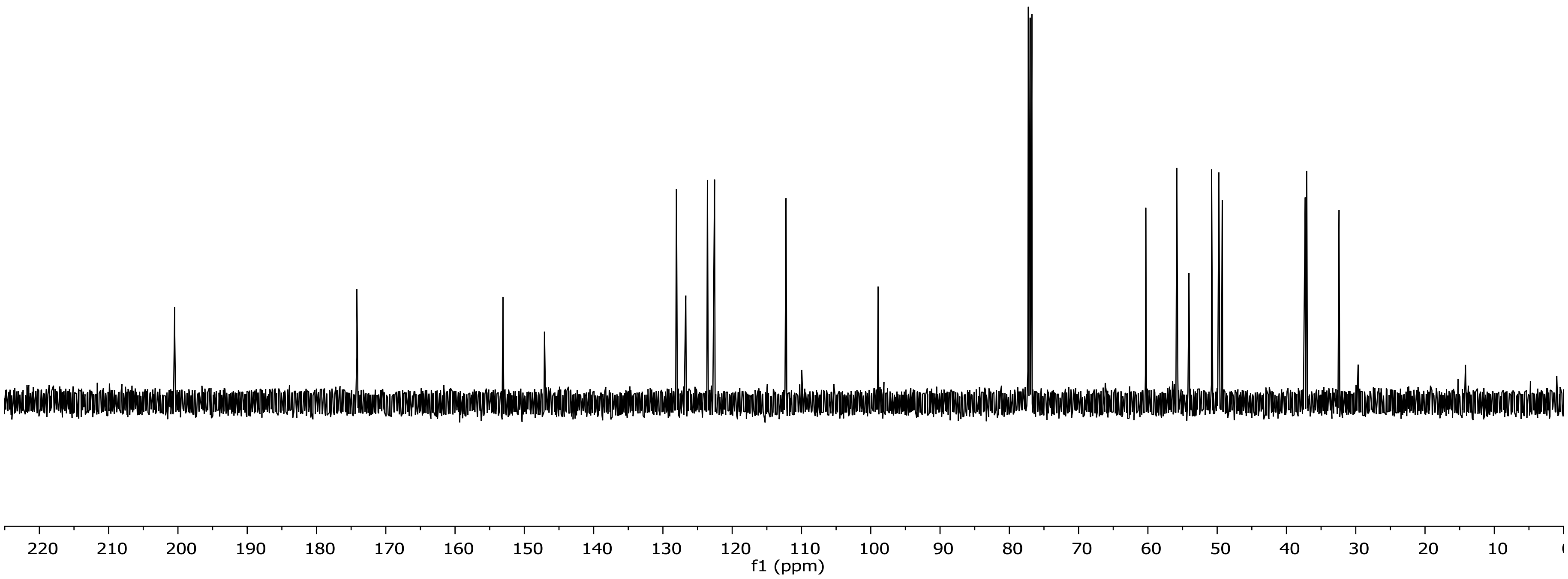

^{1}H NMR (400 MHz, $CDCl_3$)

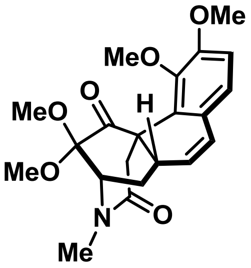

12'

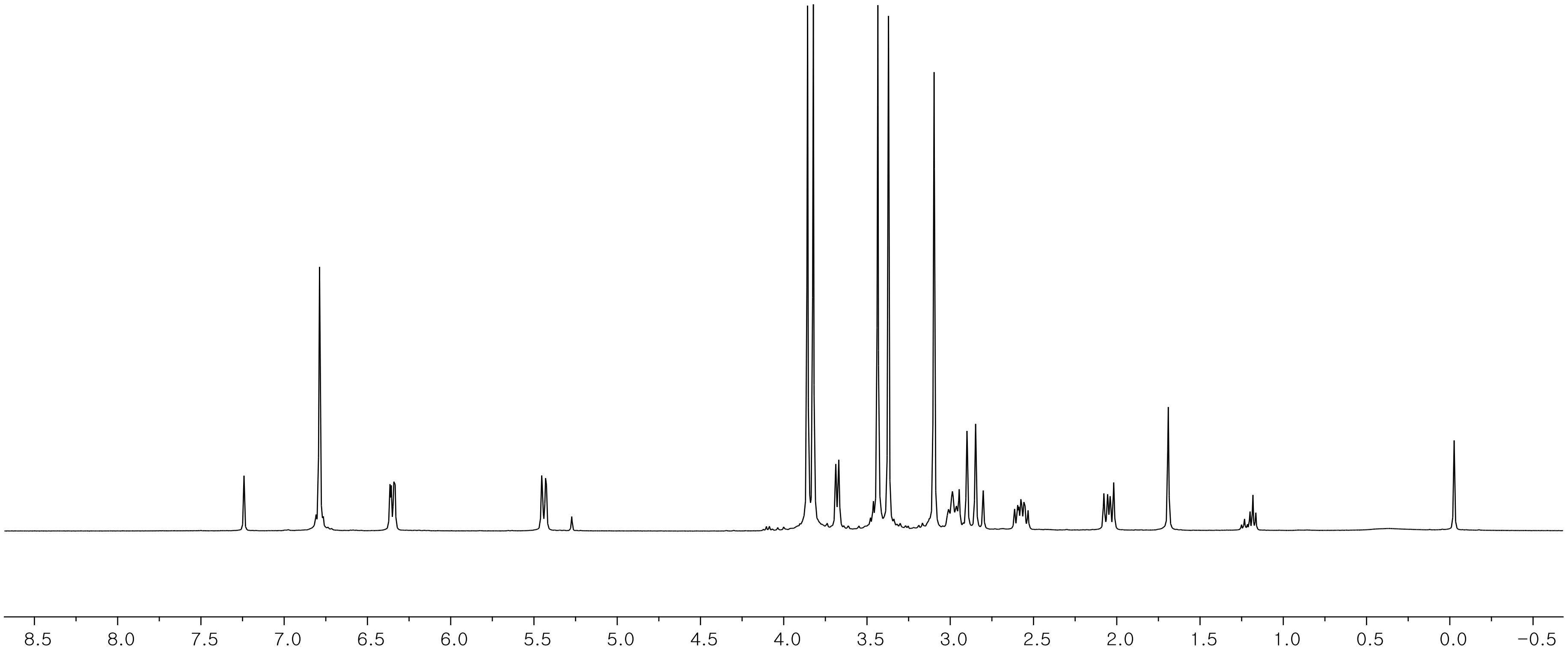

^{13}C NMR (101 MHz, $CDCl_3$)

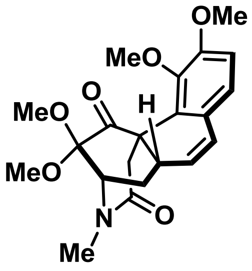

12'

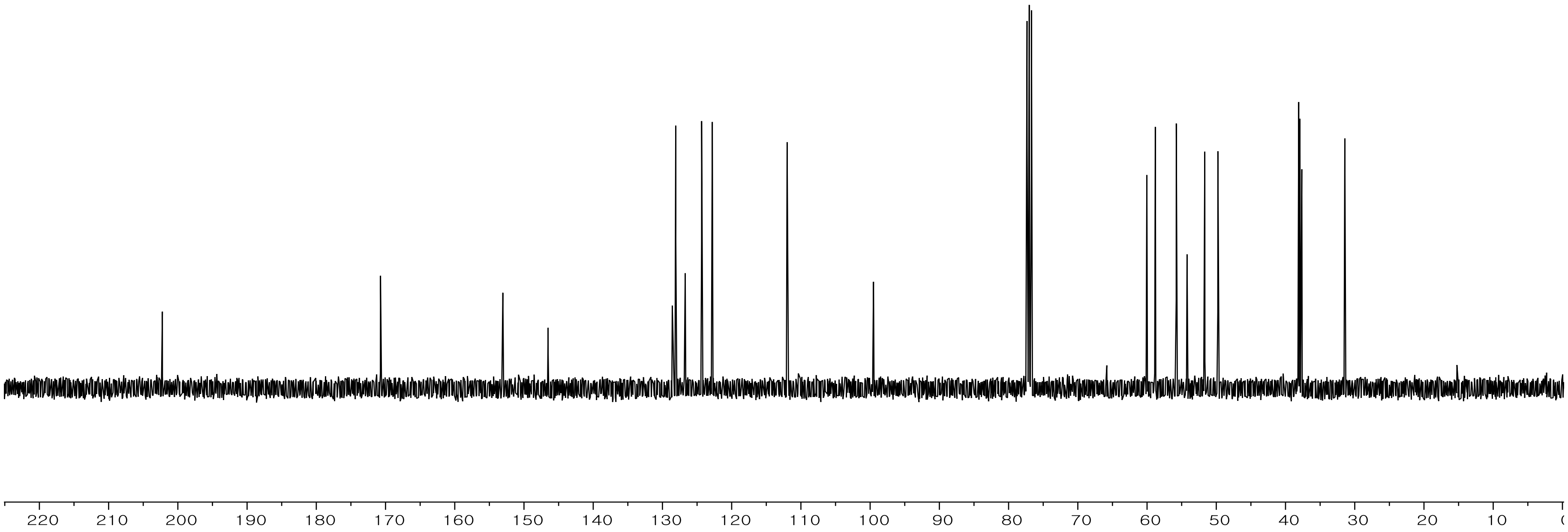

^{1}H NMR (400 MHz, CD_3OD)

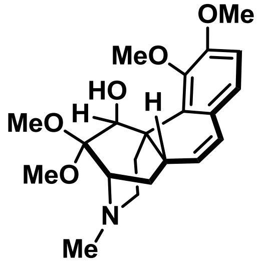

12a

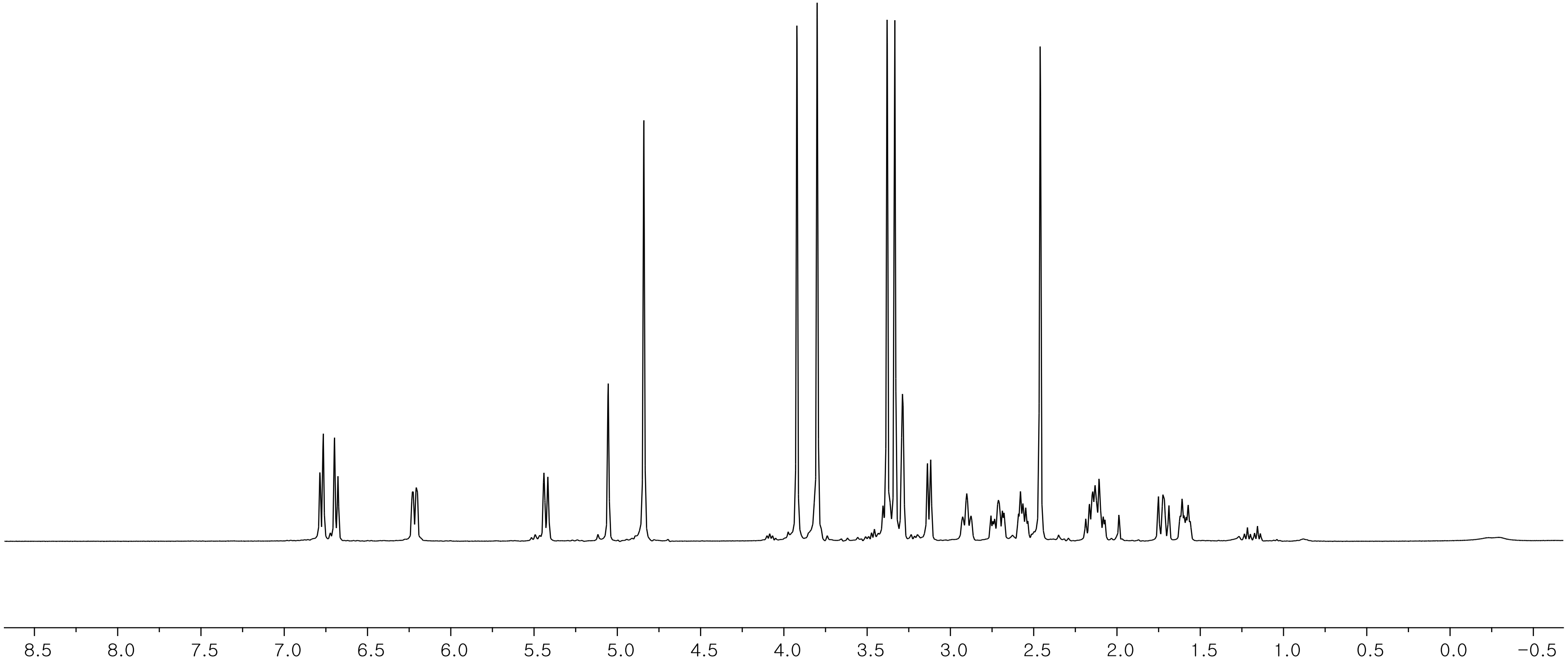

^{13}C NMR (101 MHz, CD_3OD)

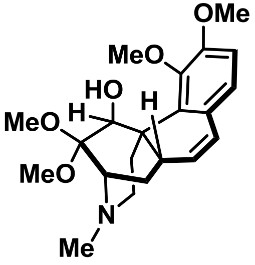

12a

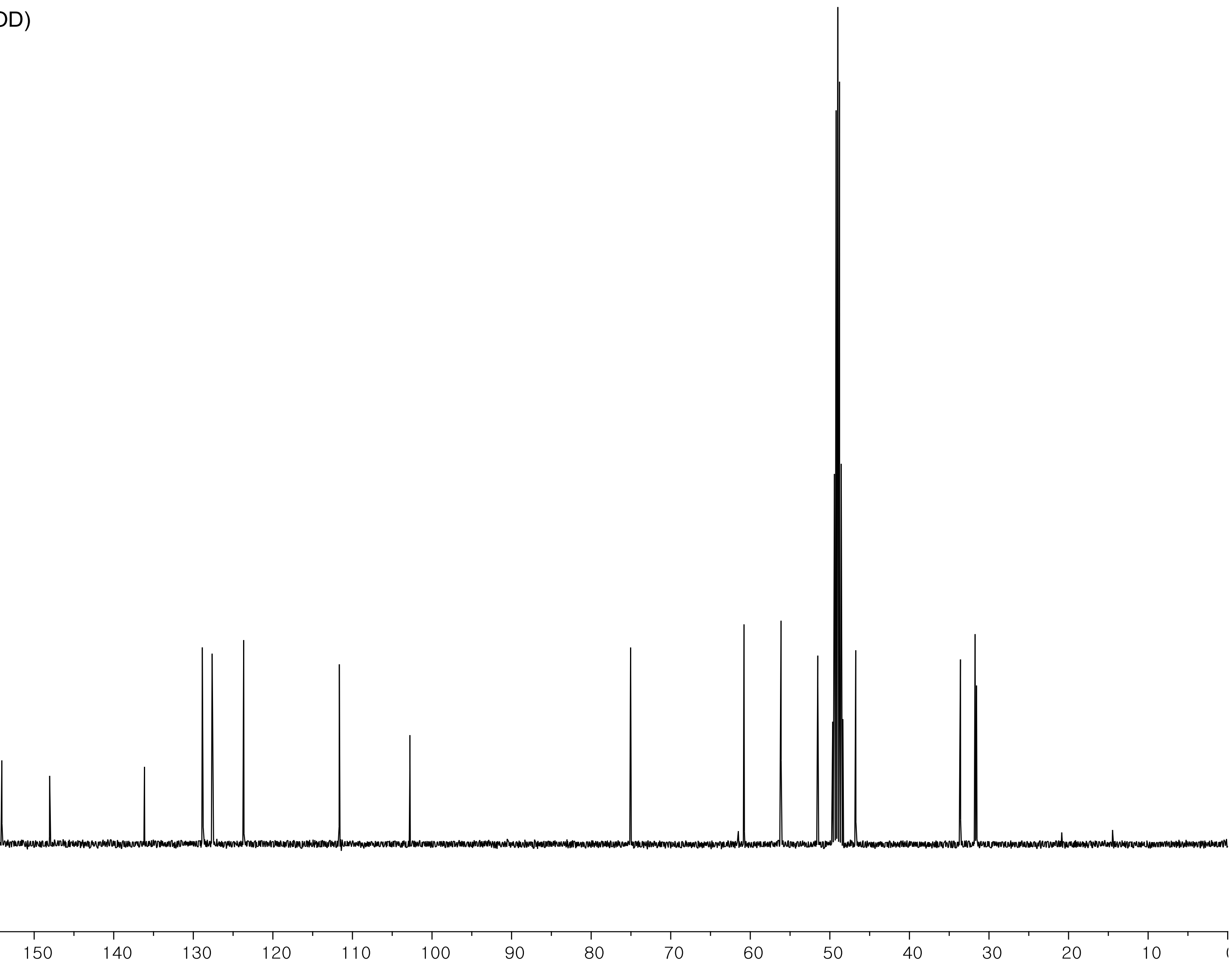

^1H NMR (400 MHz, CD_3OD)

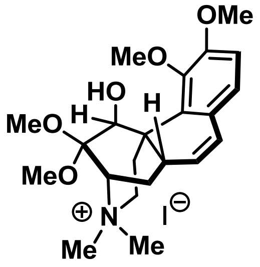

13

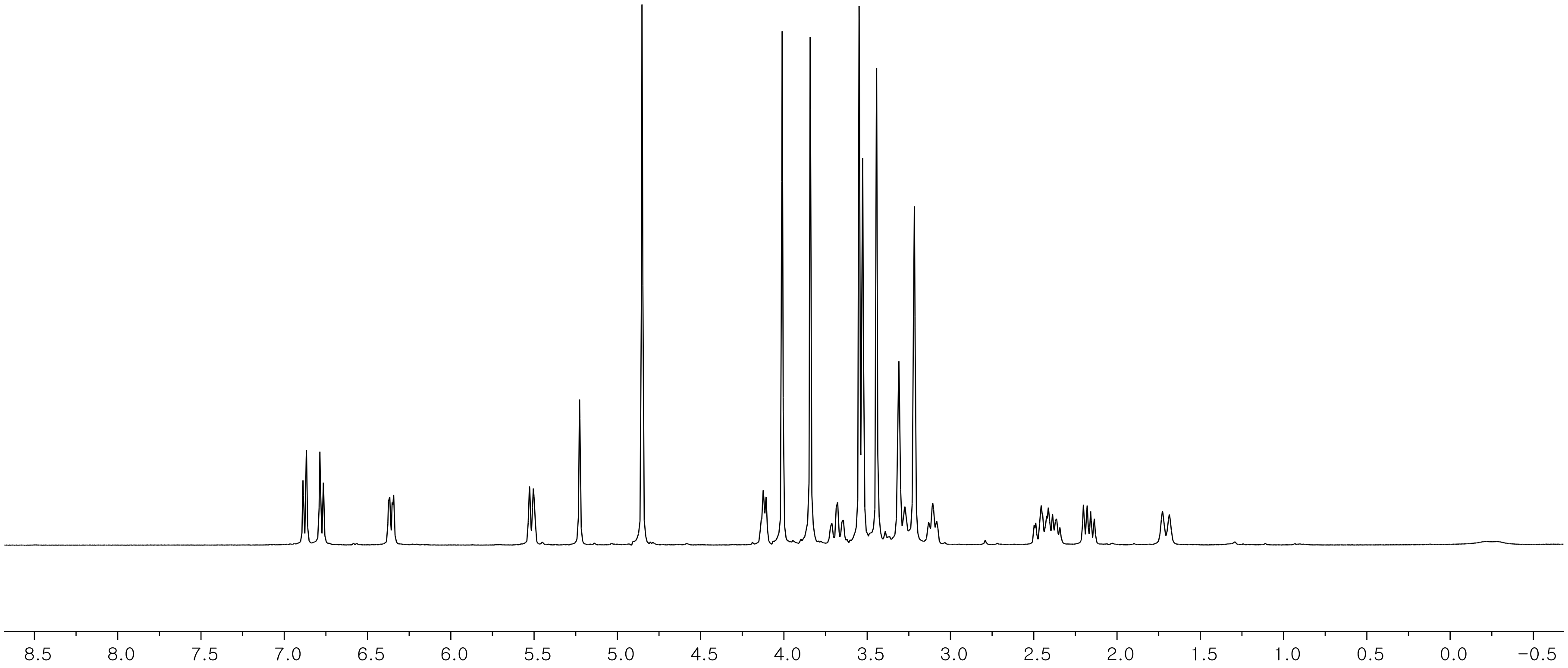

^{13}C NMR (101 MHz, CD_3OD)

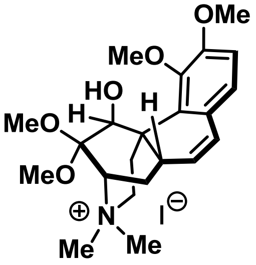

13

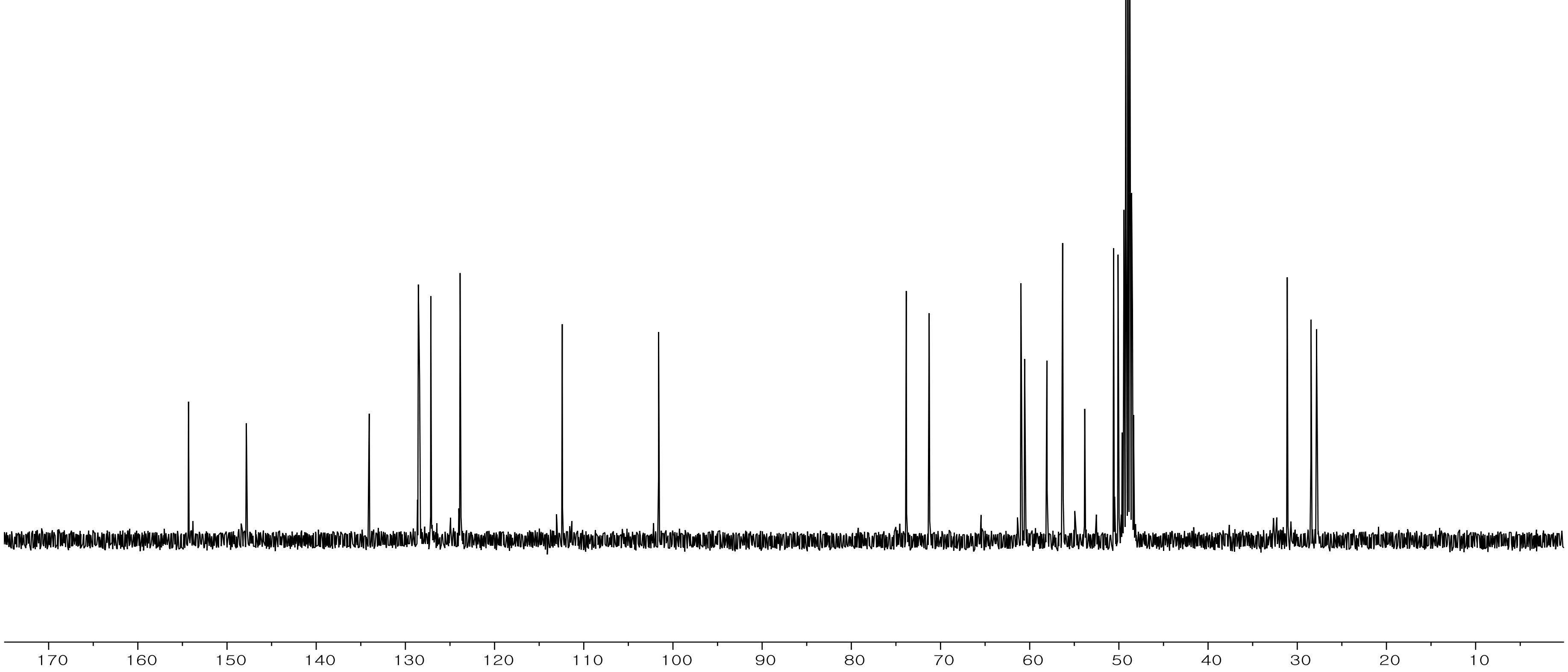

^1H NMR (400 MHz, CD_3OD)

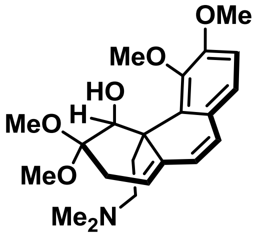

14

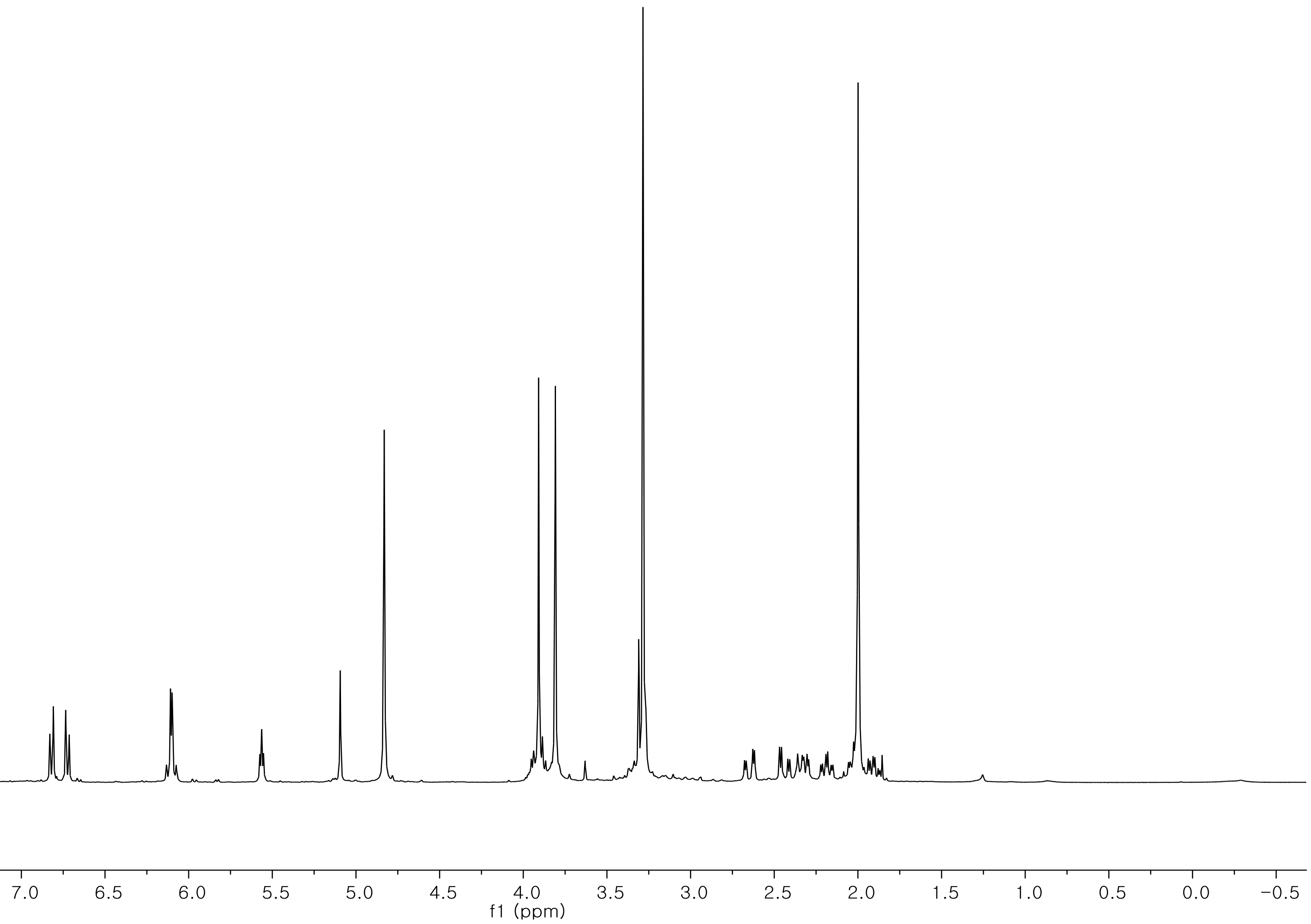

^{13}C NMR (101 MHz, $CDCl_3$)

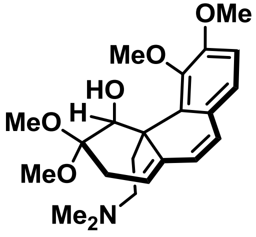

14

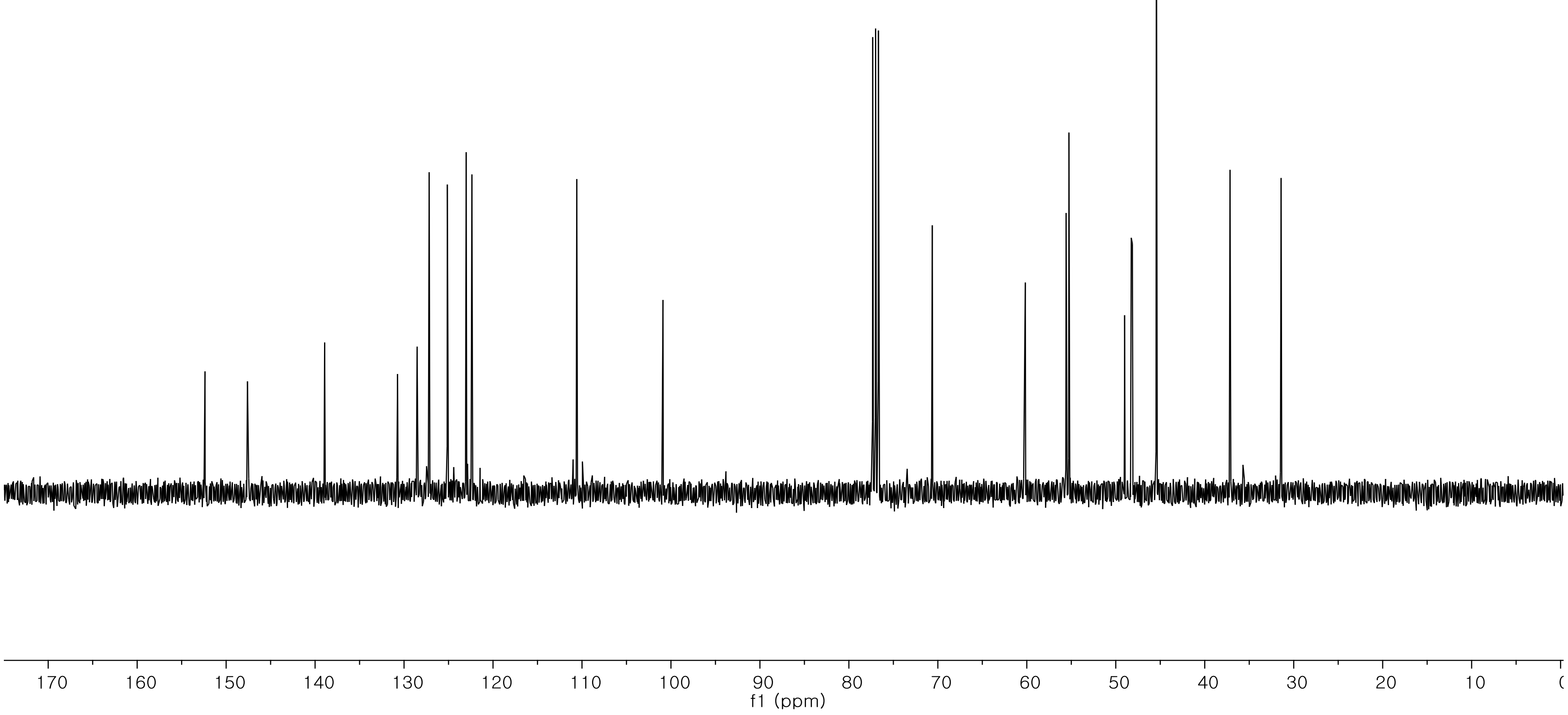

^1H NMR (400 MHz, $CDCl_3$)

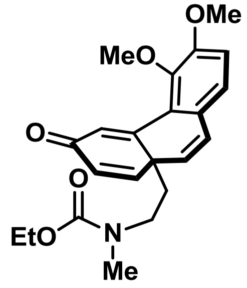

15

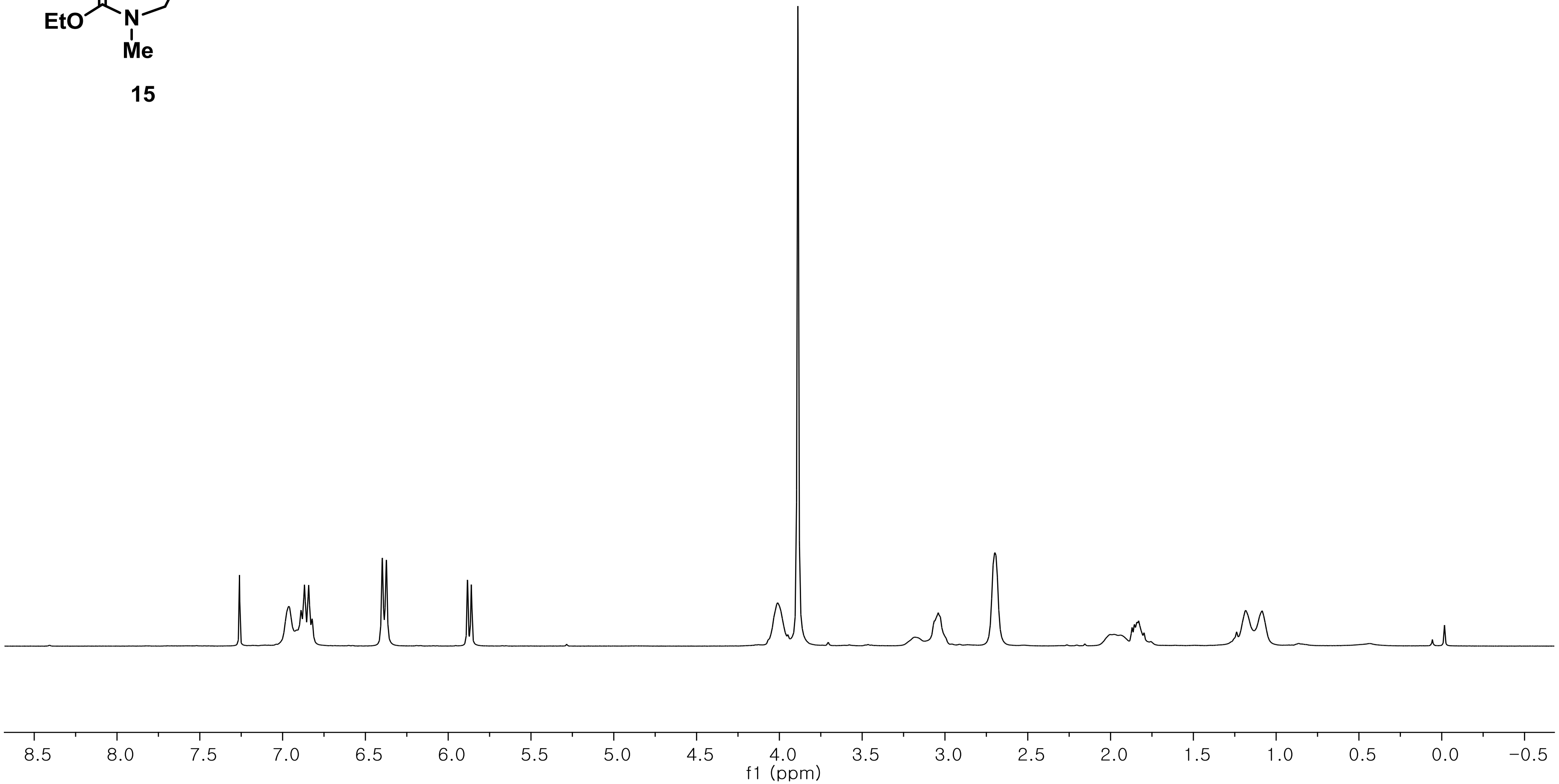

^{13}C NMR (101 MHz, $CDCl_3$)

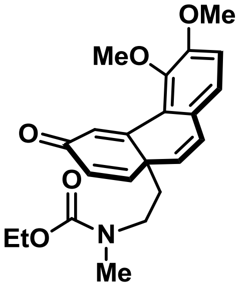

15

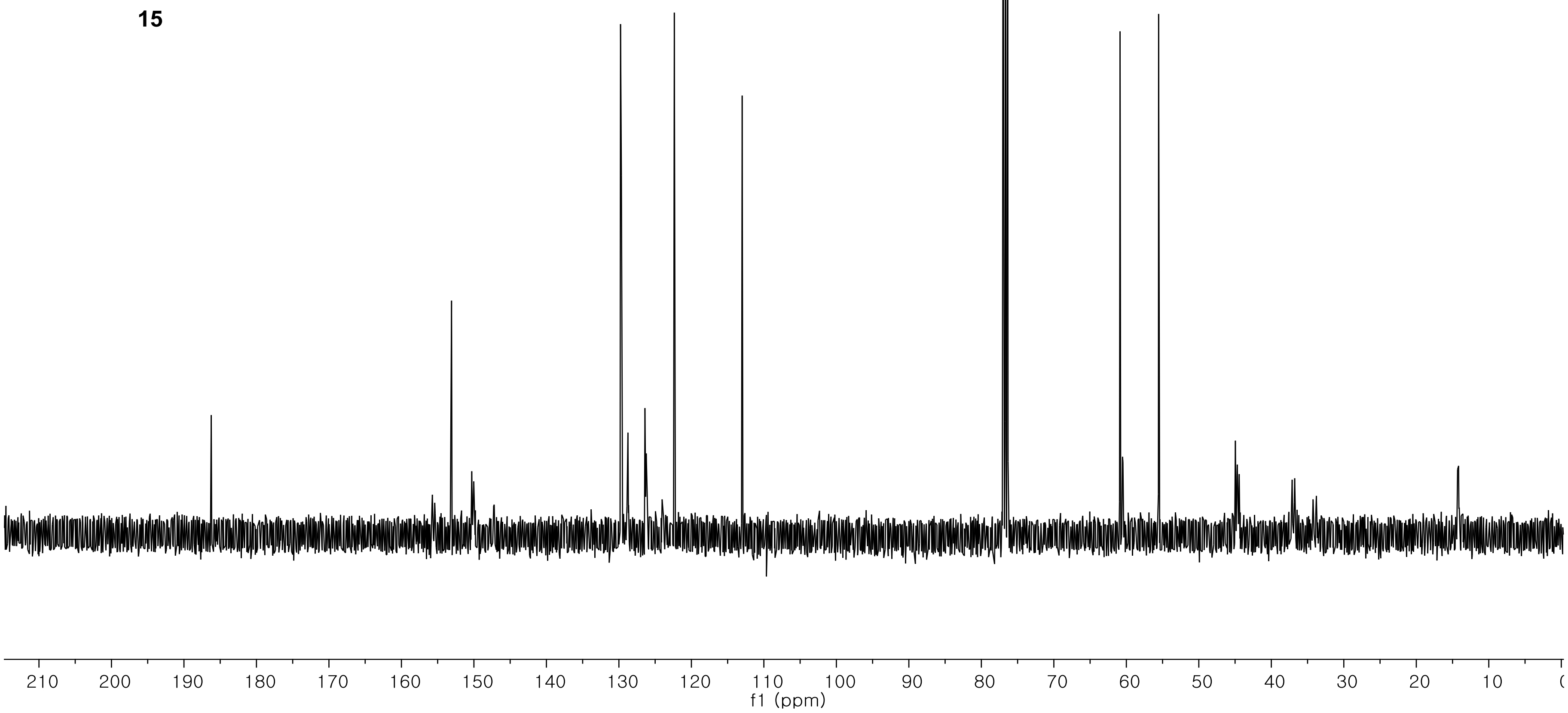

^{1}H NMR (400 MHz, $CDCl_3$)

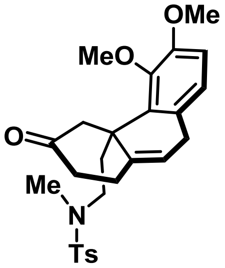

18

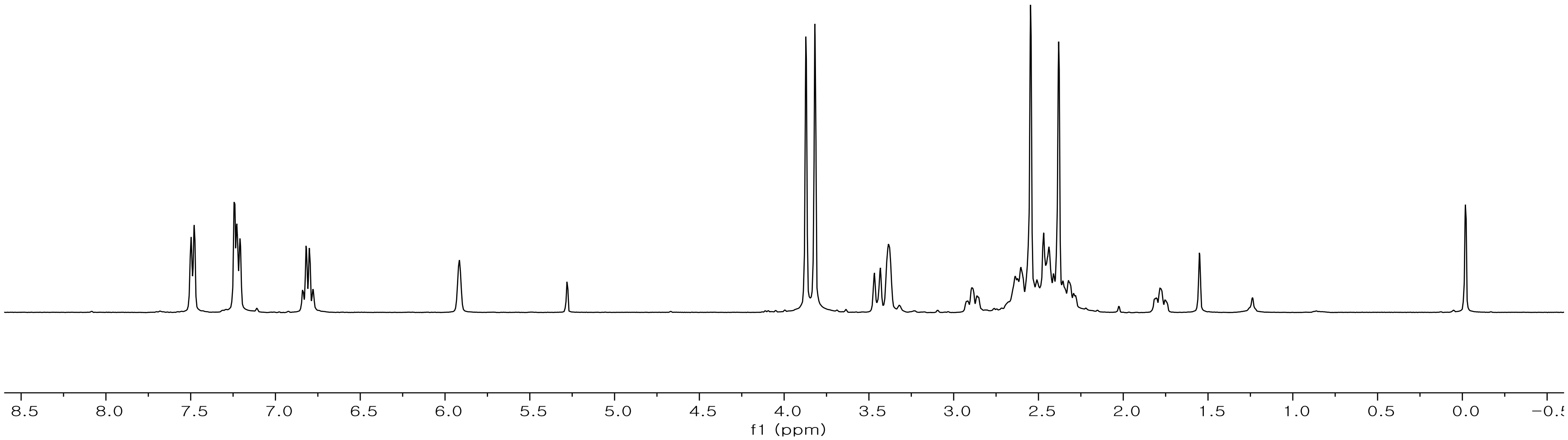

^{13}C NMR (101 MHz, $CDCl_3$)

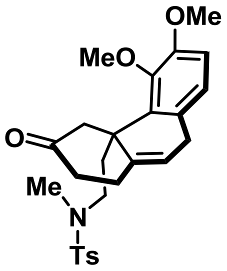

18

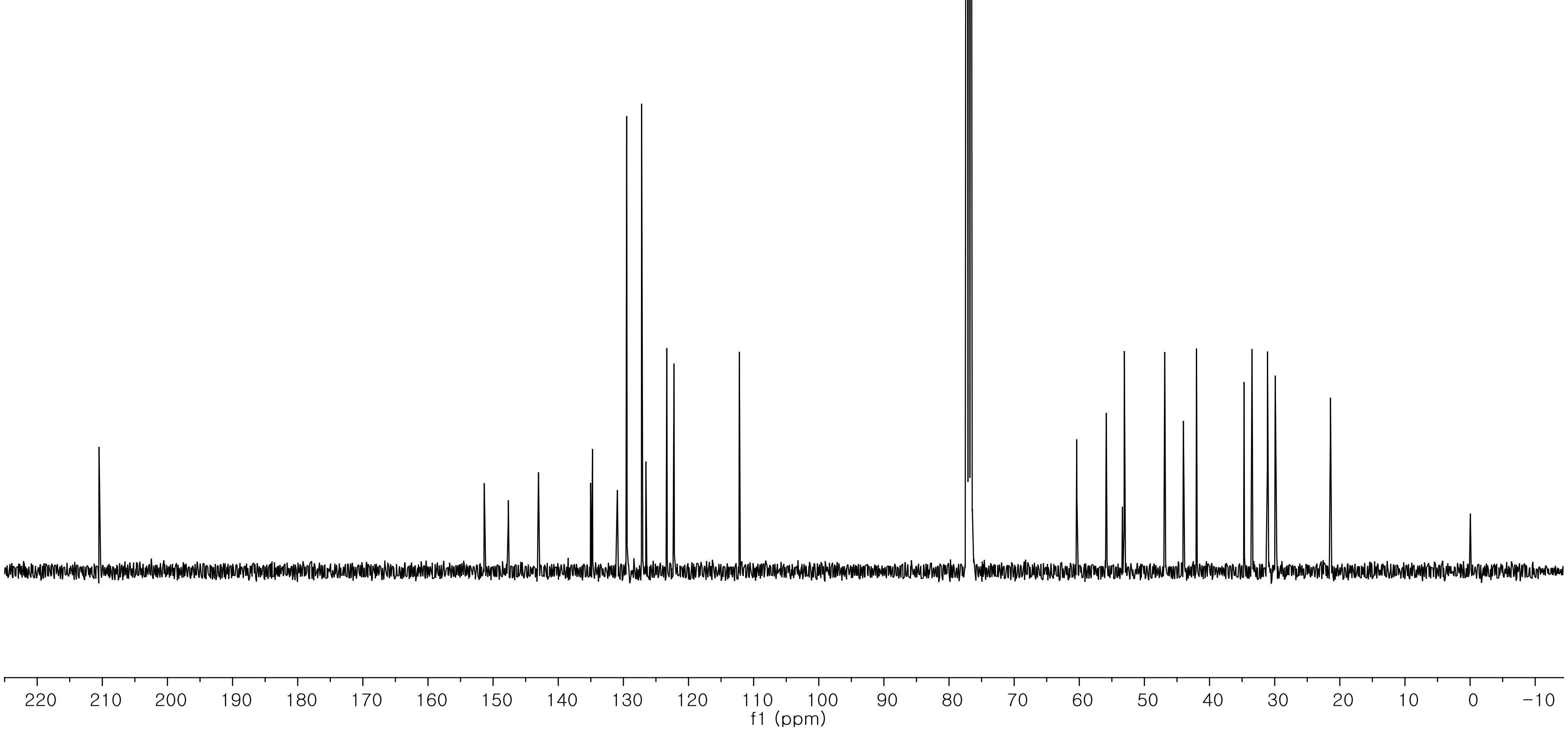

^{1}H NMR (500 MHz, $CDCl_3$)

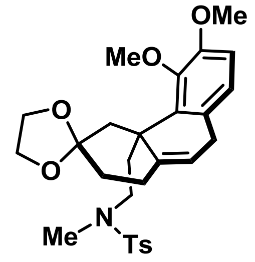

18a

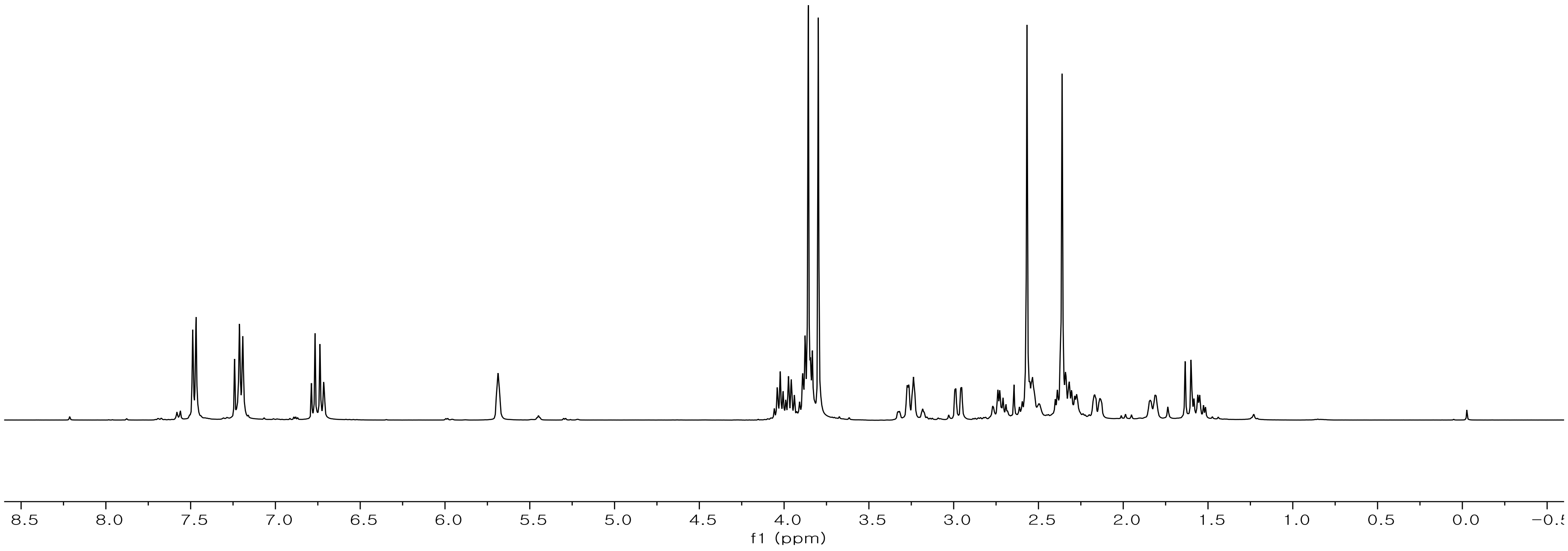

^{13}C NMR (101 MHz, $CDCl_3$)

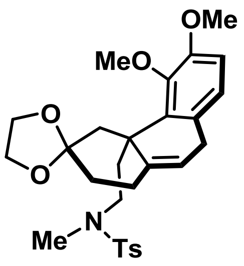

18a

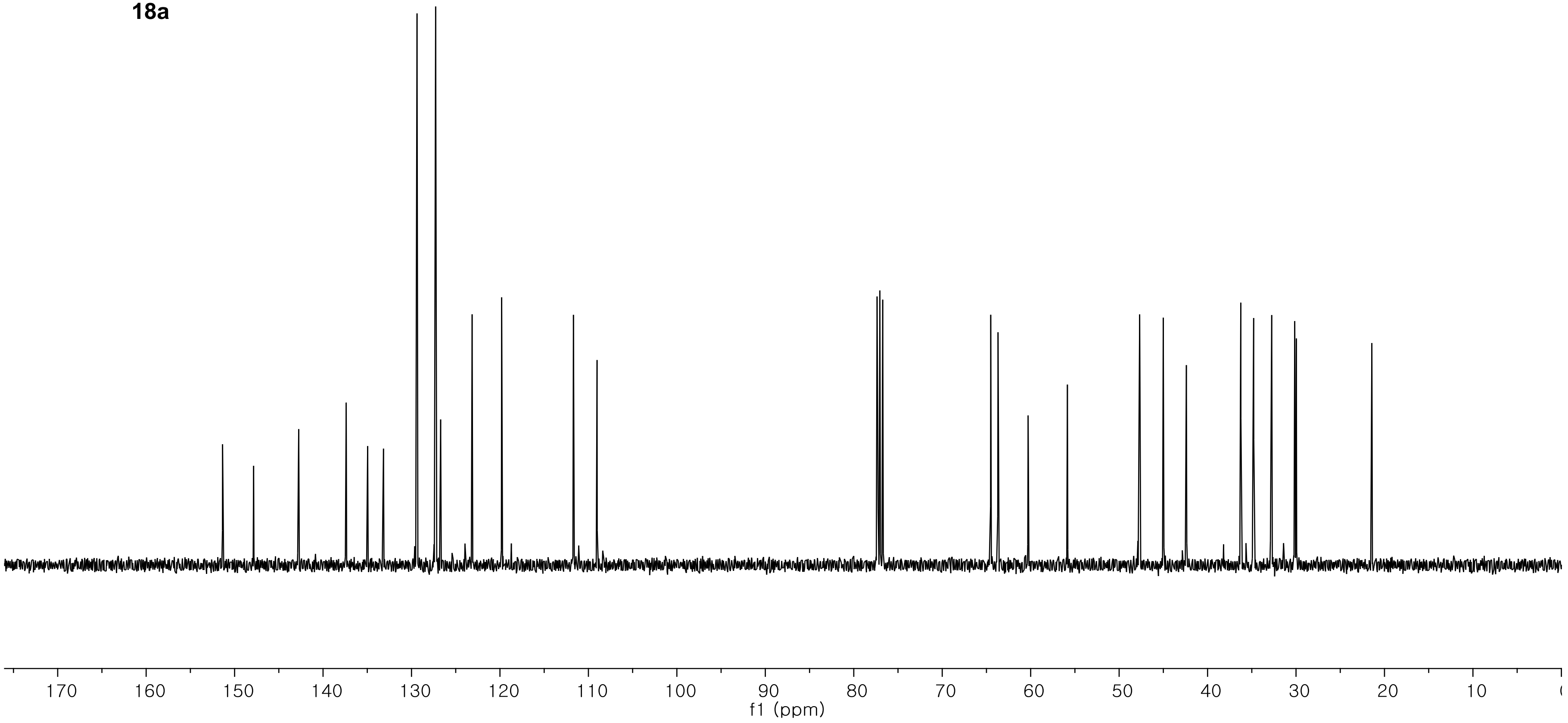

^1H NMR (500 MHz, $CDCl_3$)

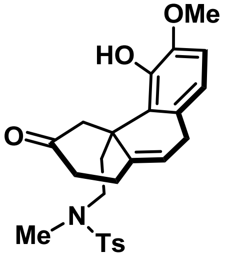

19

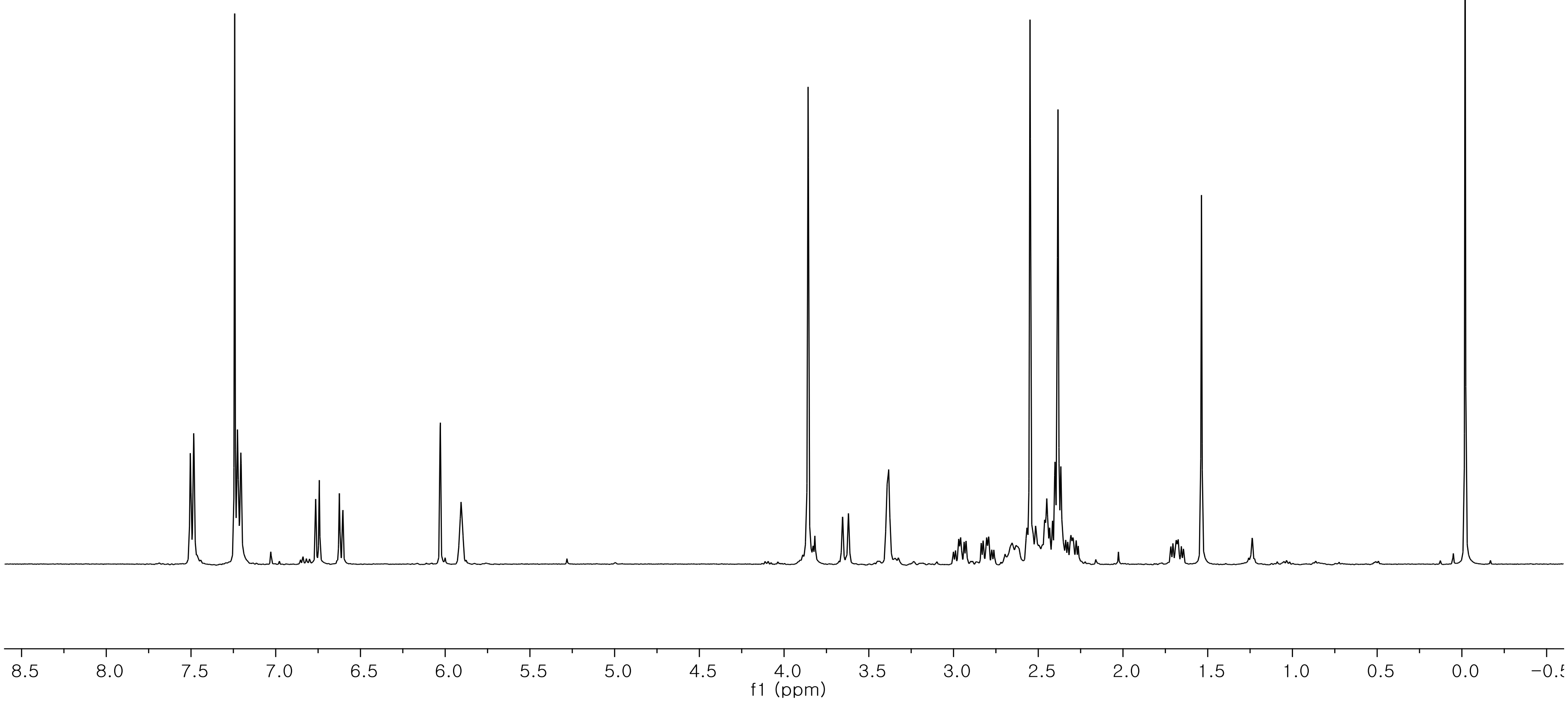

^{13}C NMR (101 MHz, $CDCl_3$)

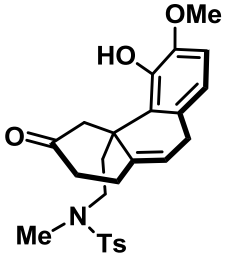

19

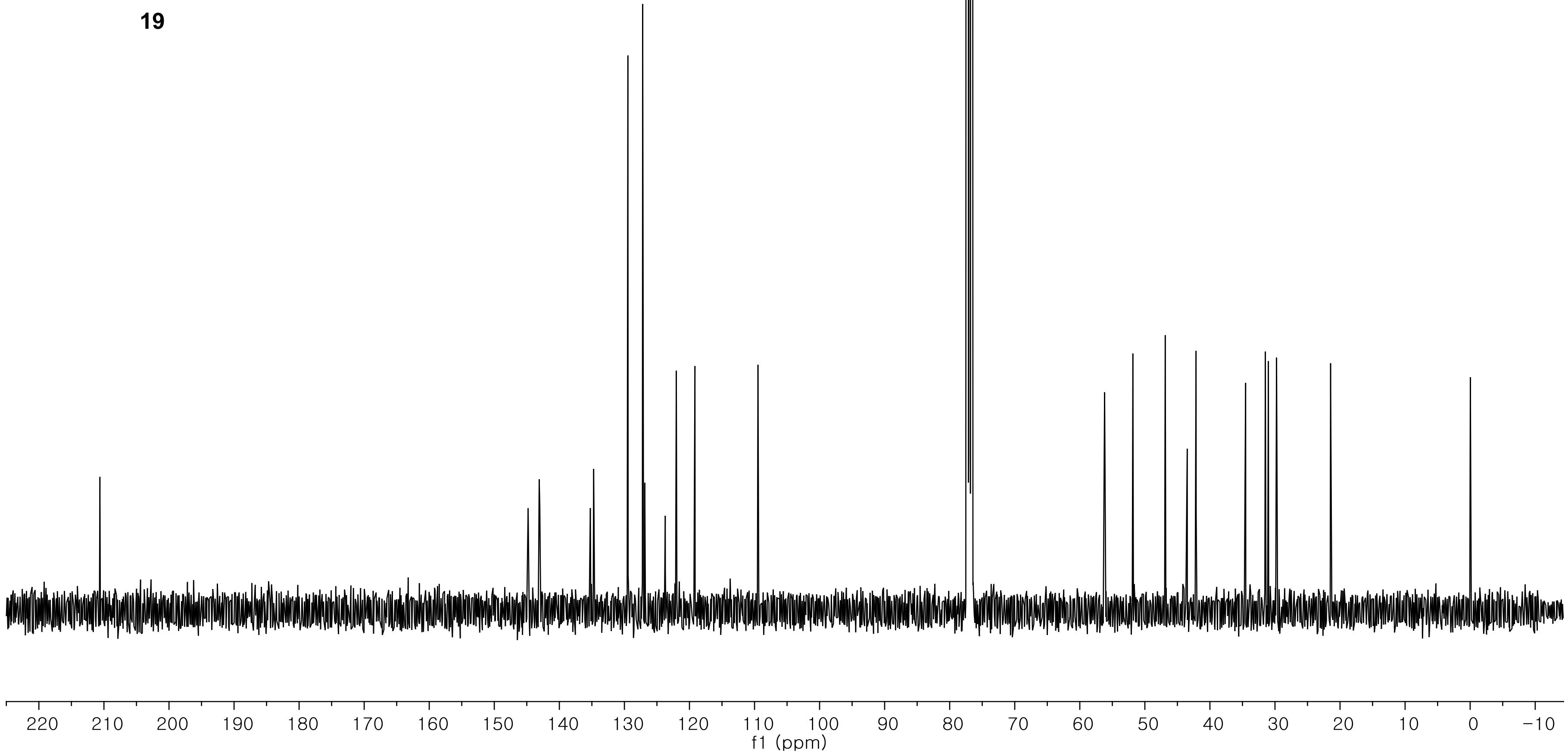

^1H NMR (400 MHz, $CDCl_3$)

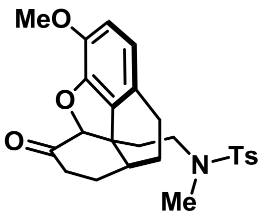

20b

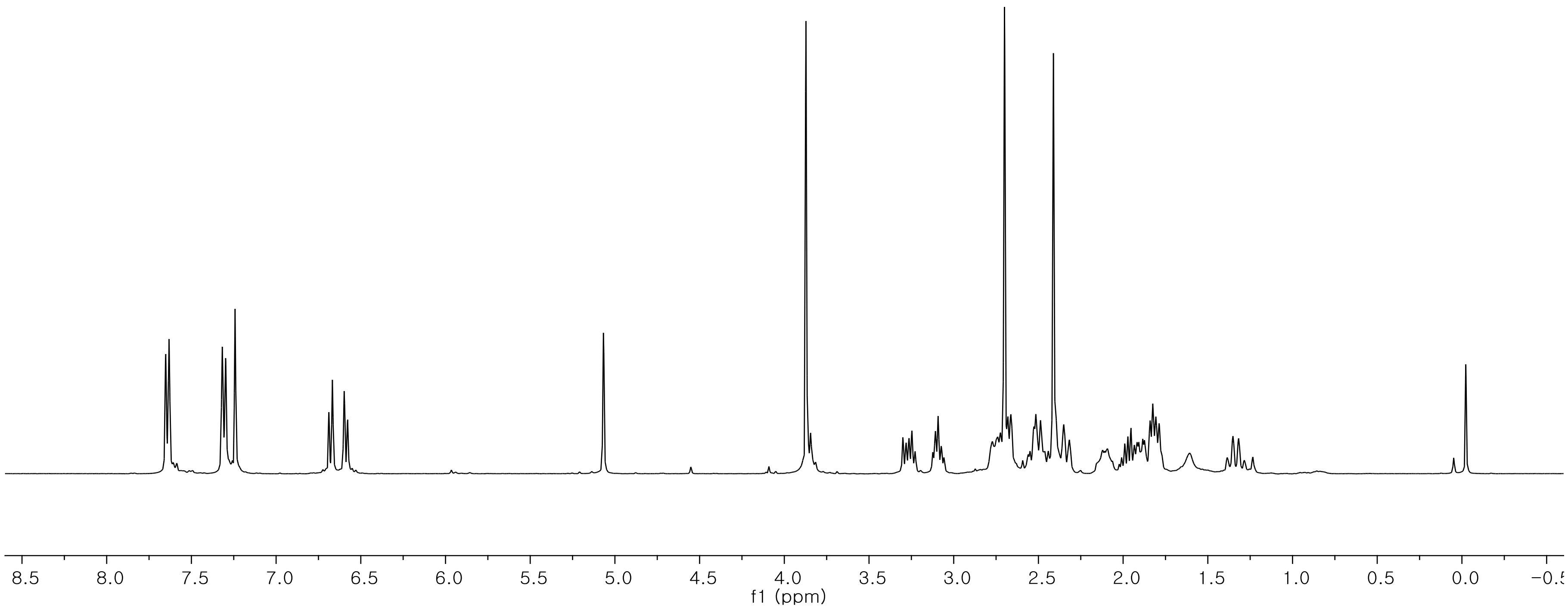

^{13}C NMR (101 MHz, $CDCl_3$)

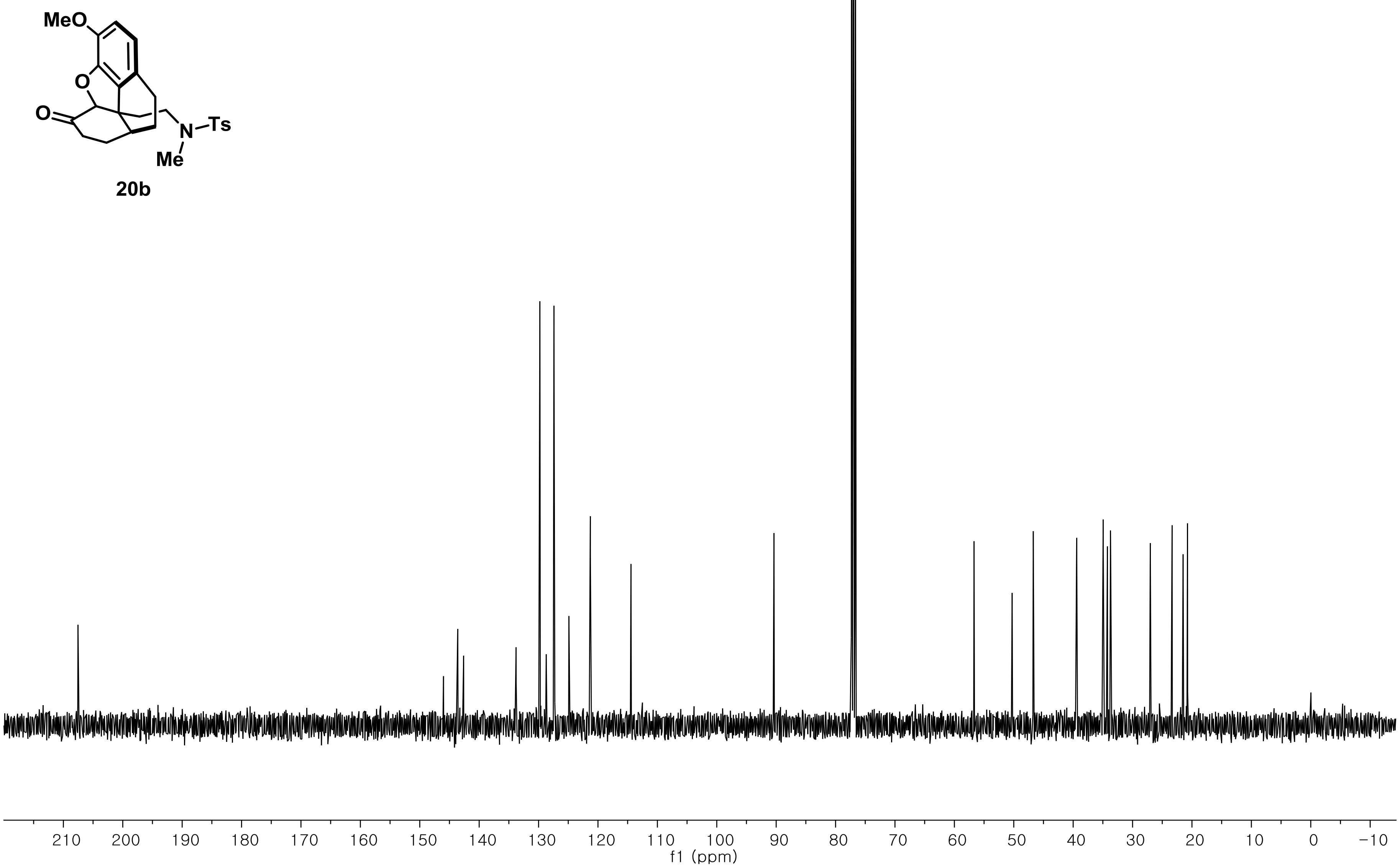

^{1}H NMR (400 MHz, $CDCl_3$)

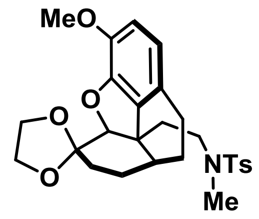

20c

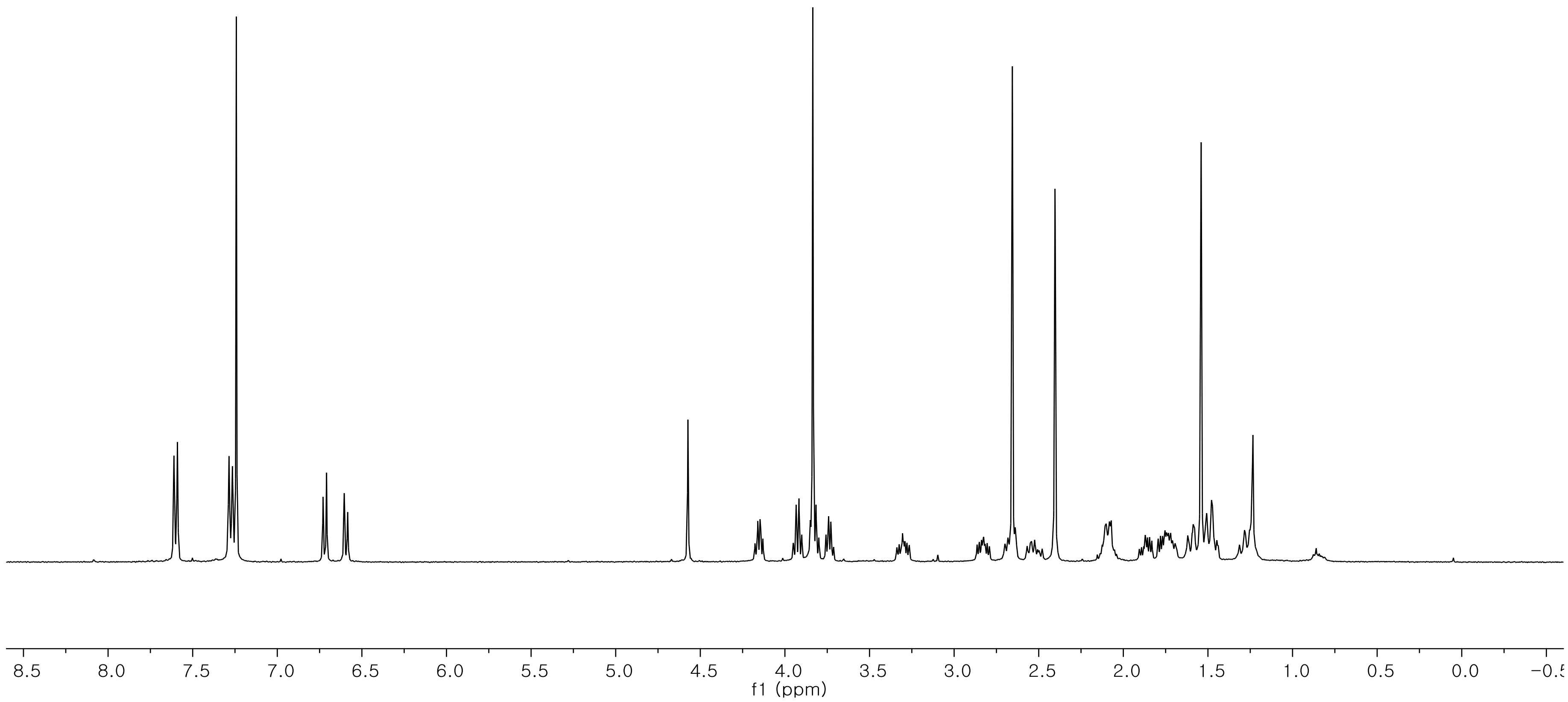

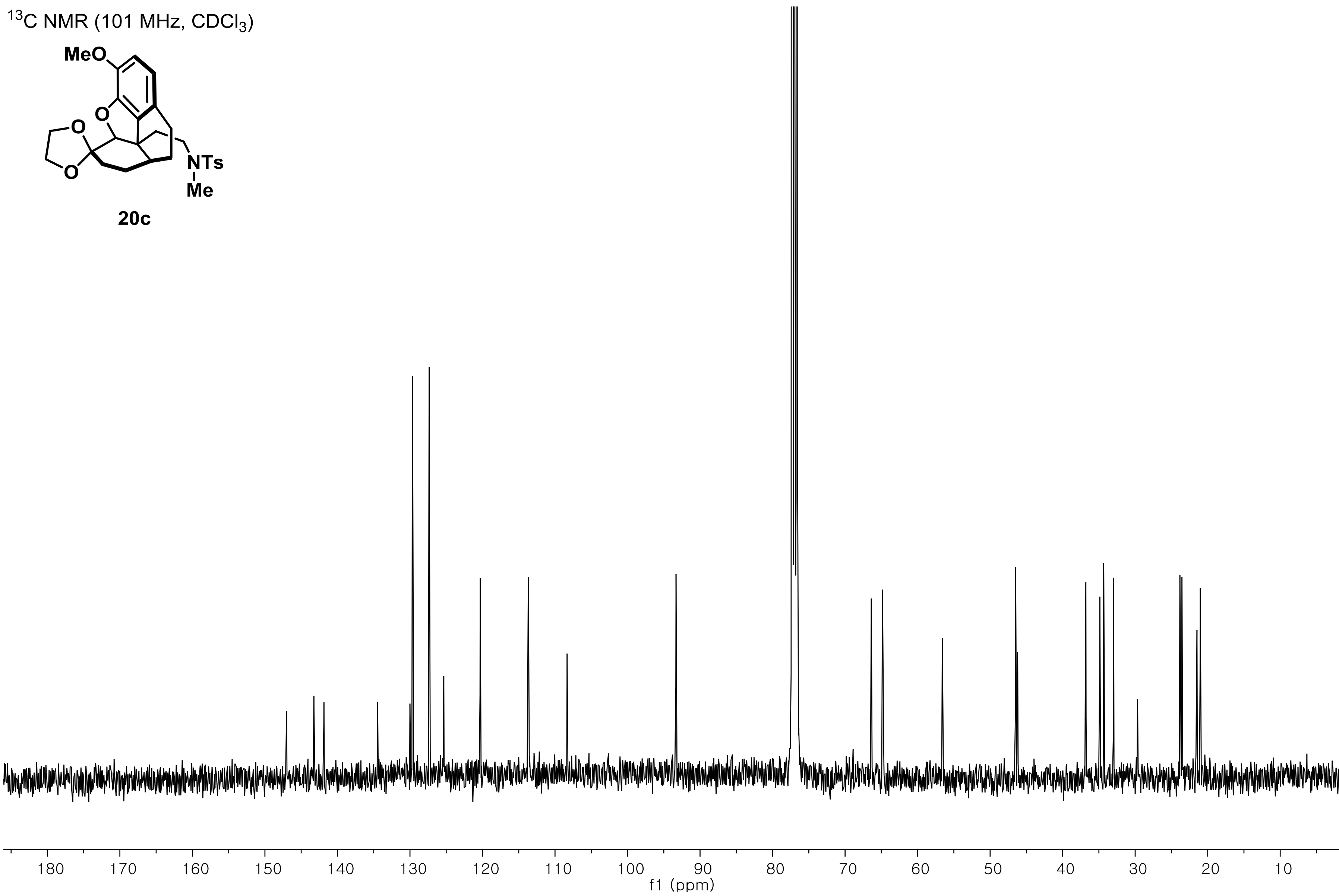
^{13}C NMR (101 MHz, CDCl$_3$)
MeO
O
O
O
NTs
Me
20c
180
170
160
150
140
130
120
110
100
90
80
70
60
50
40
30
20
10
f1 (ppm)

^{1}H NMR (400 MHz, $CDCl_3$)

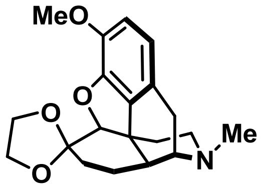

21a

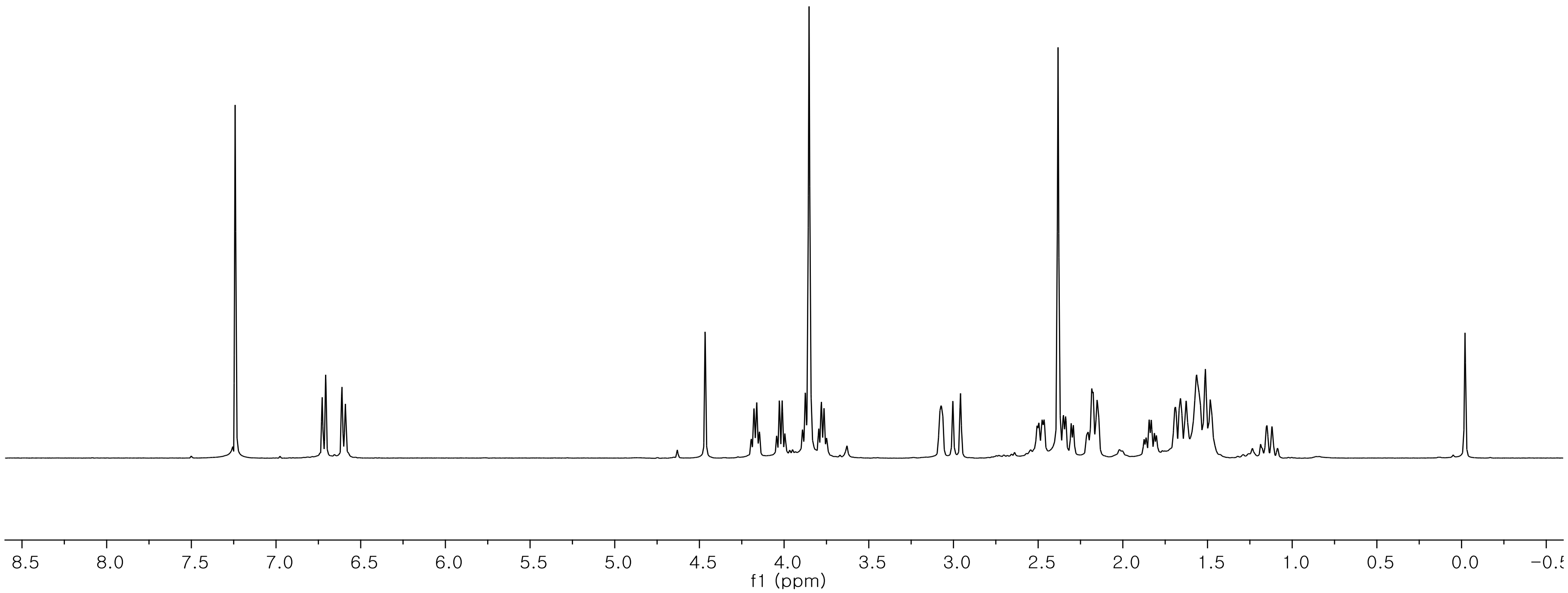

^{13}C NMR (101 MHz, $CDCl_3$)

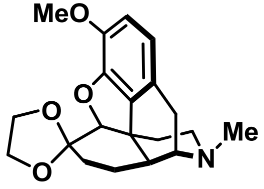

21a

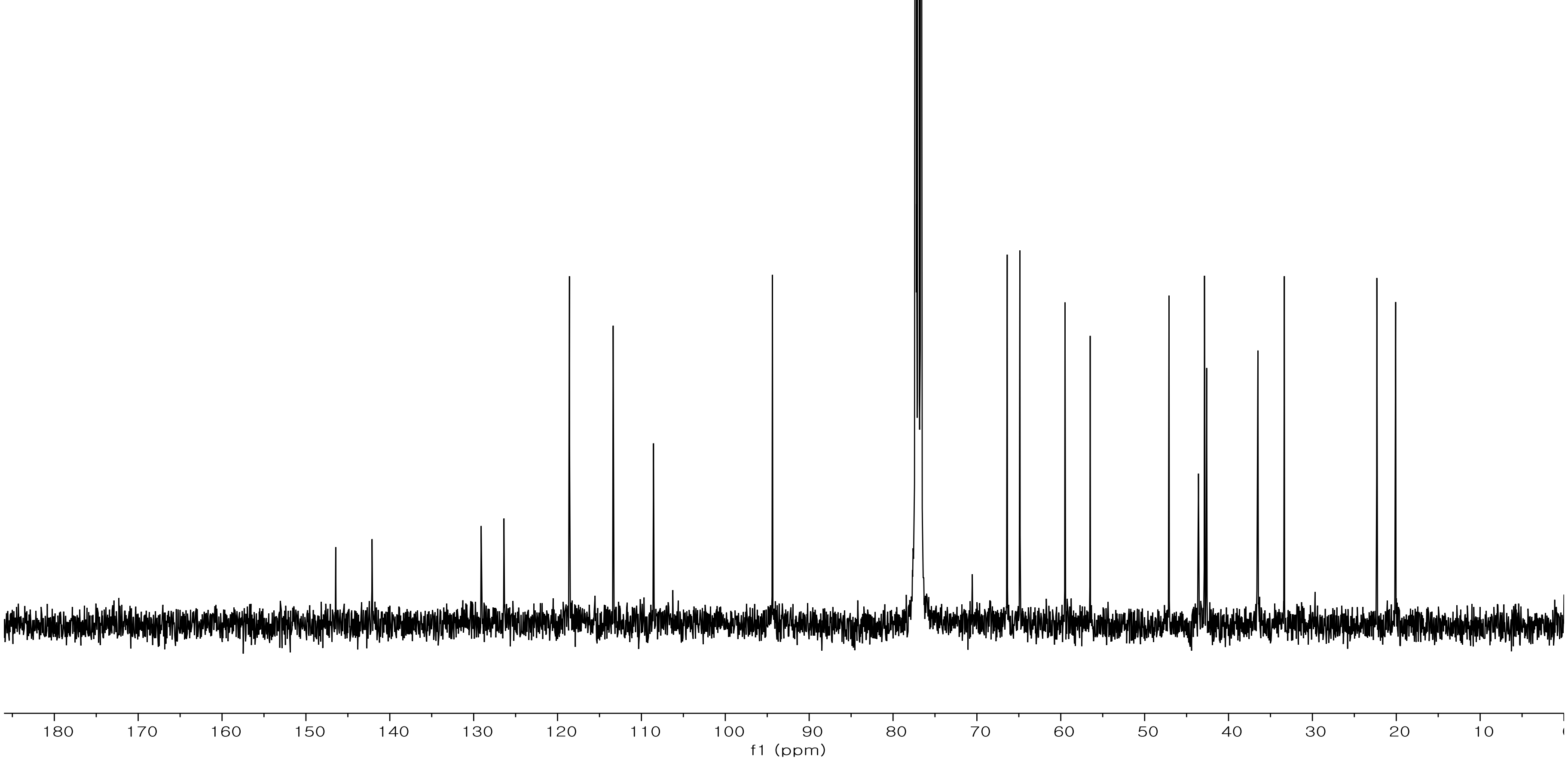

^{1}H NMR (400 MHz, $CDCl_3$)

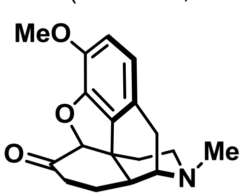

dihydrocodeinone

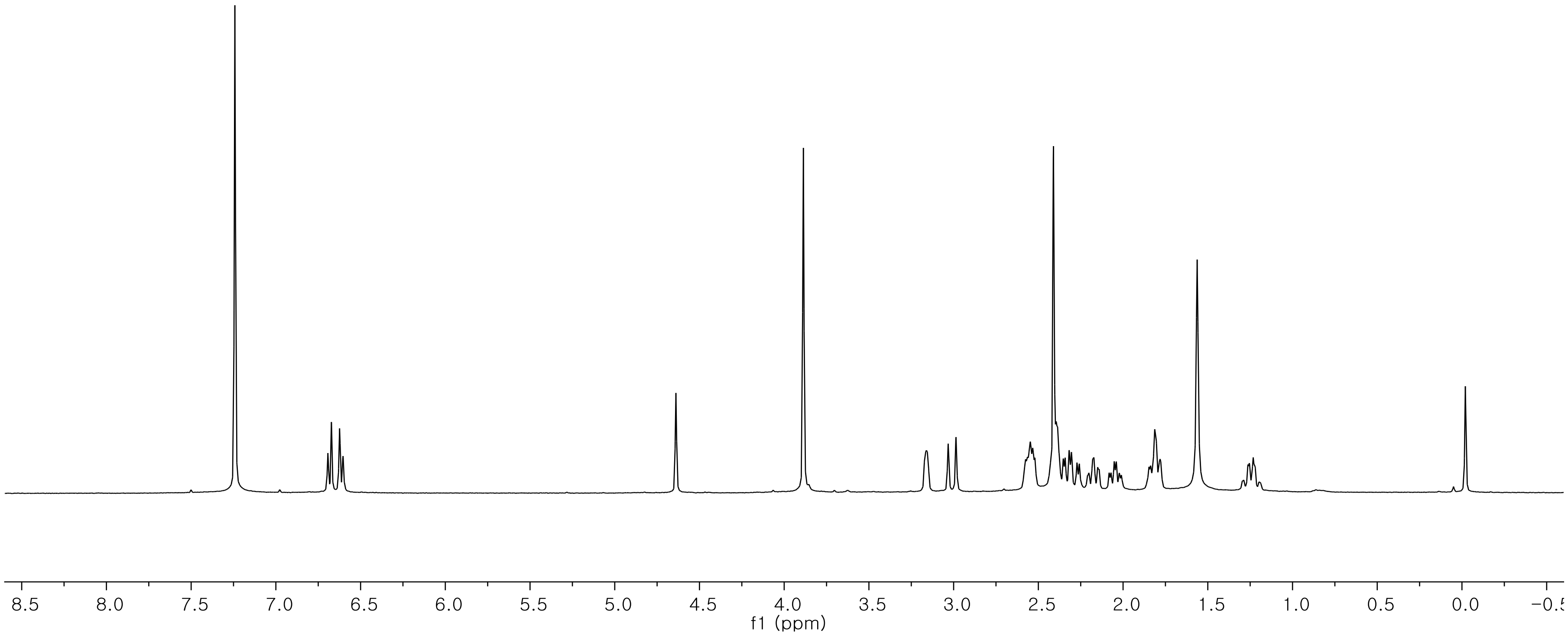

^{13}C NMR (101 MHz, $CDCl_3$)

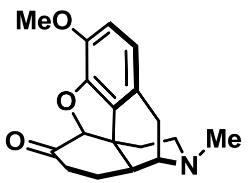

dihydrocodeinone

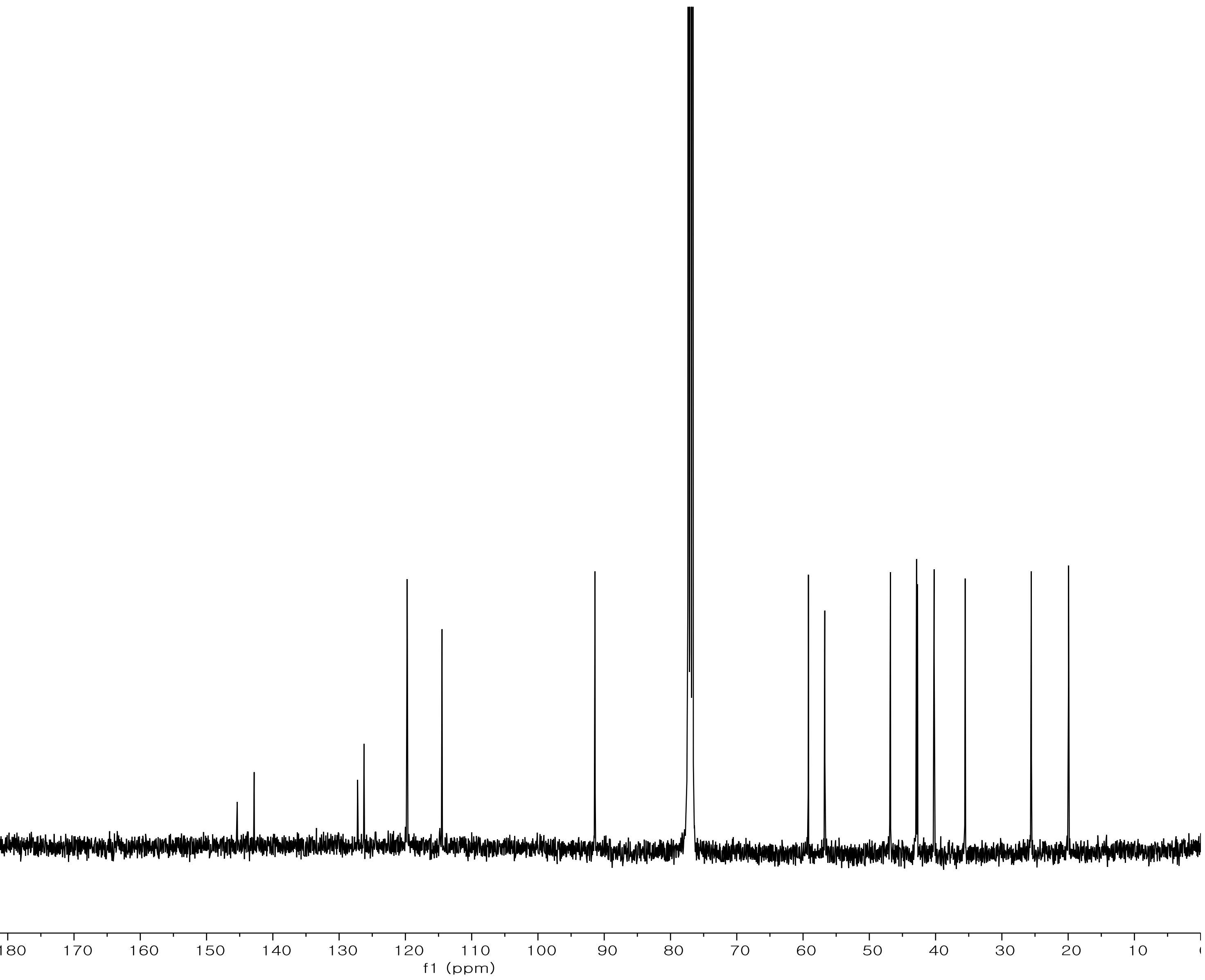

^{1}H NMR (400 MHz, $CDCl_3$)

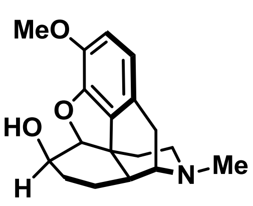

dihydrocodeine

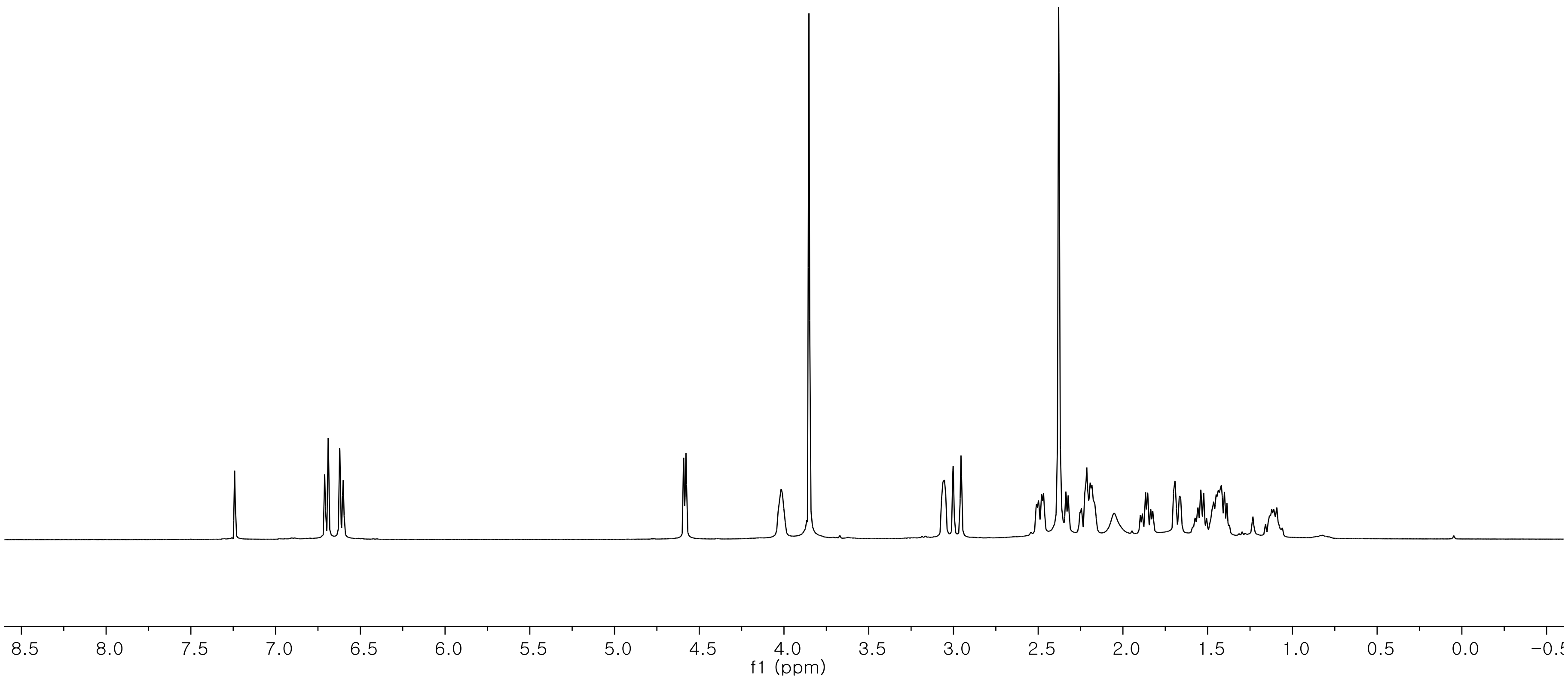

^{13}C NMR (101 MHz, $CDCl_3$)

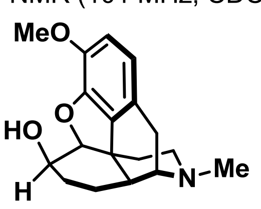

dihydrocodeine

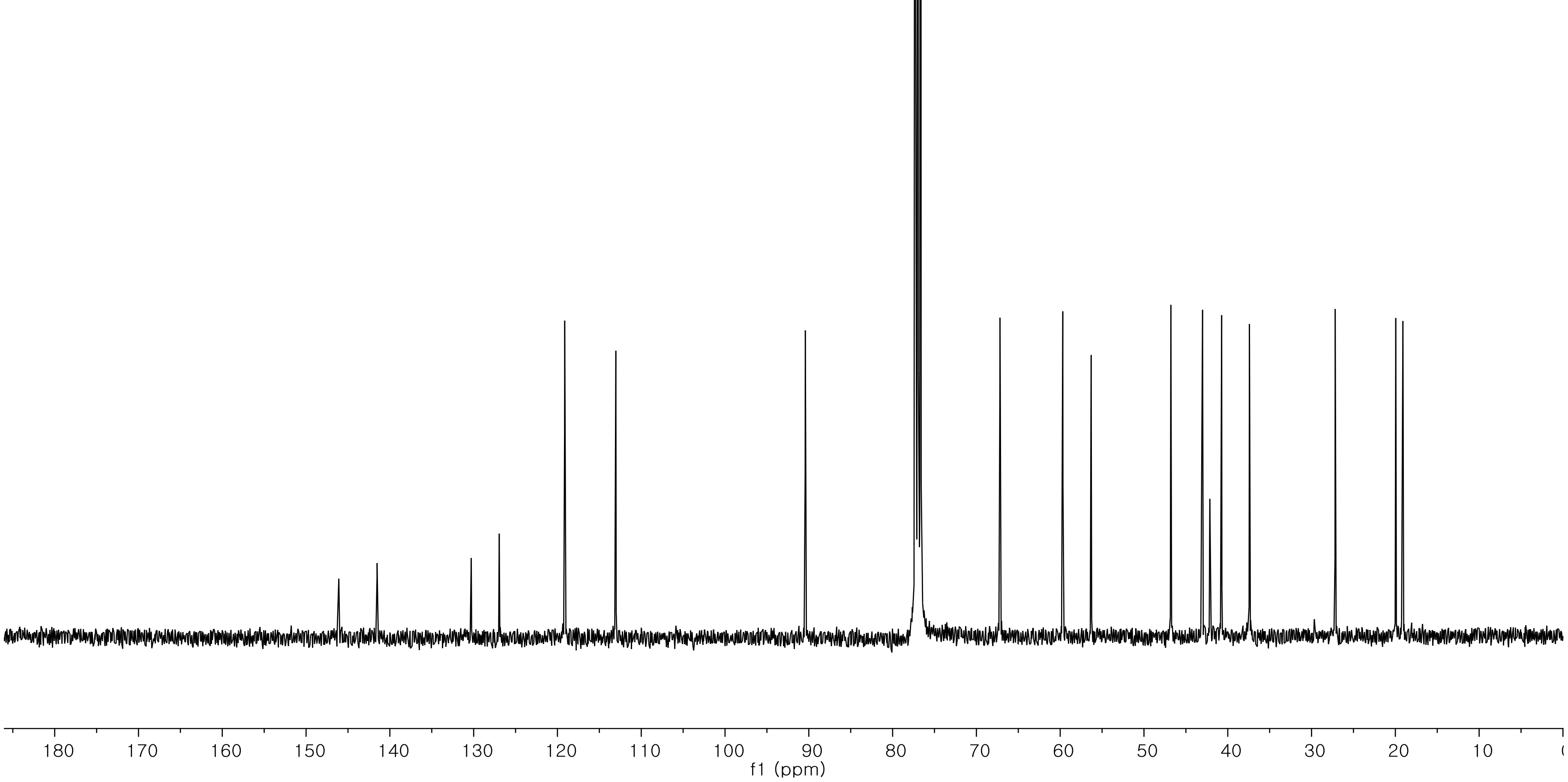

^{1}H NMR (400 MHz, $CDCl_3$)

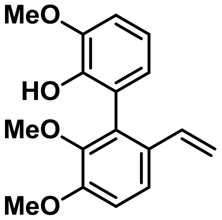

2j

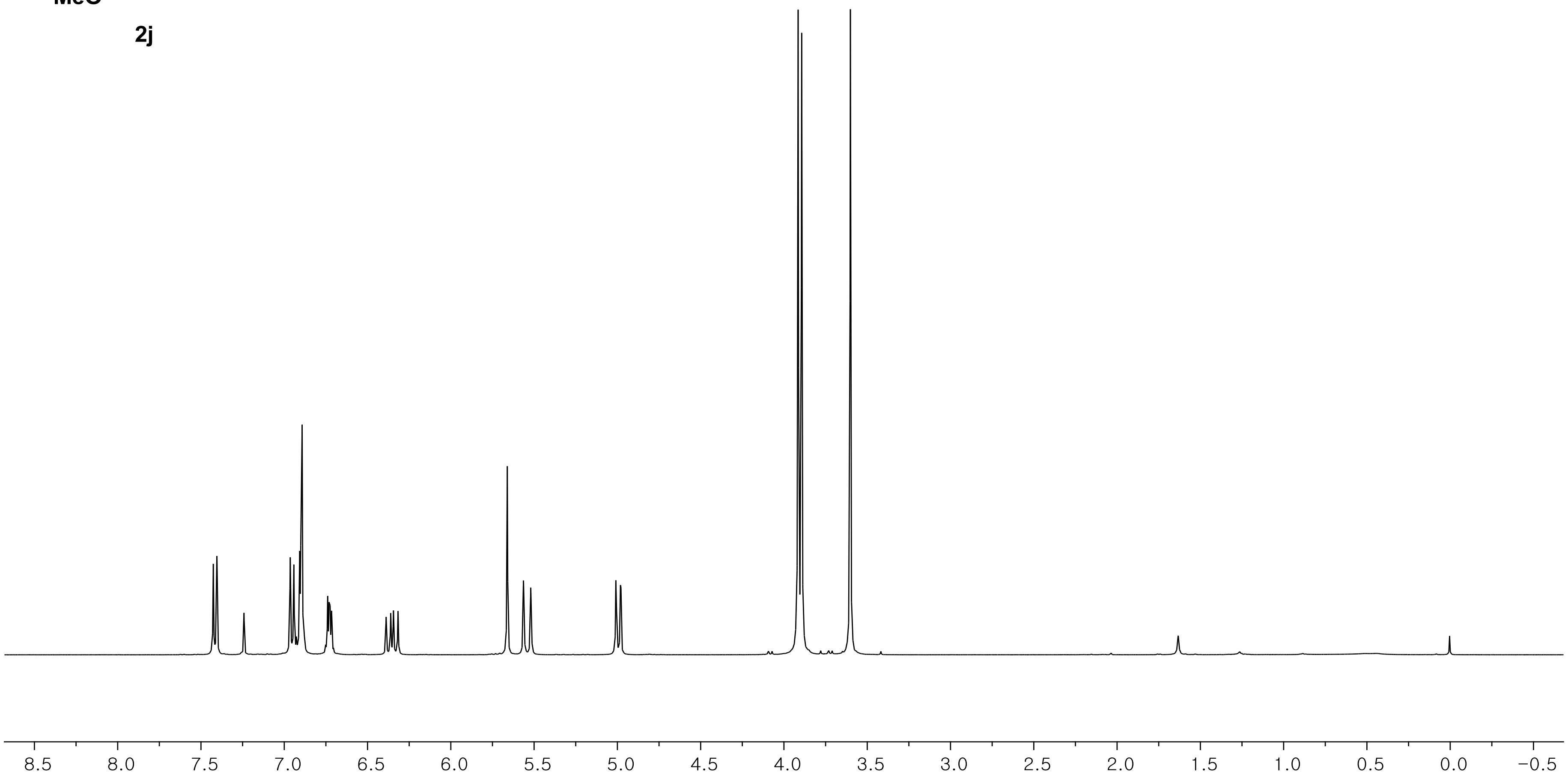

^{13}C NMR (101 MHz, $CDCl_3$)

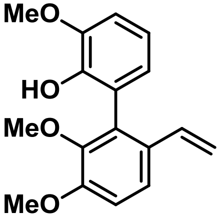

2j

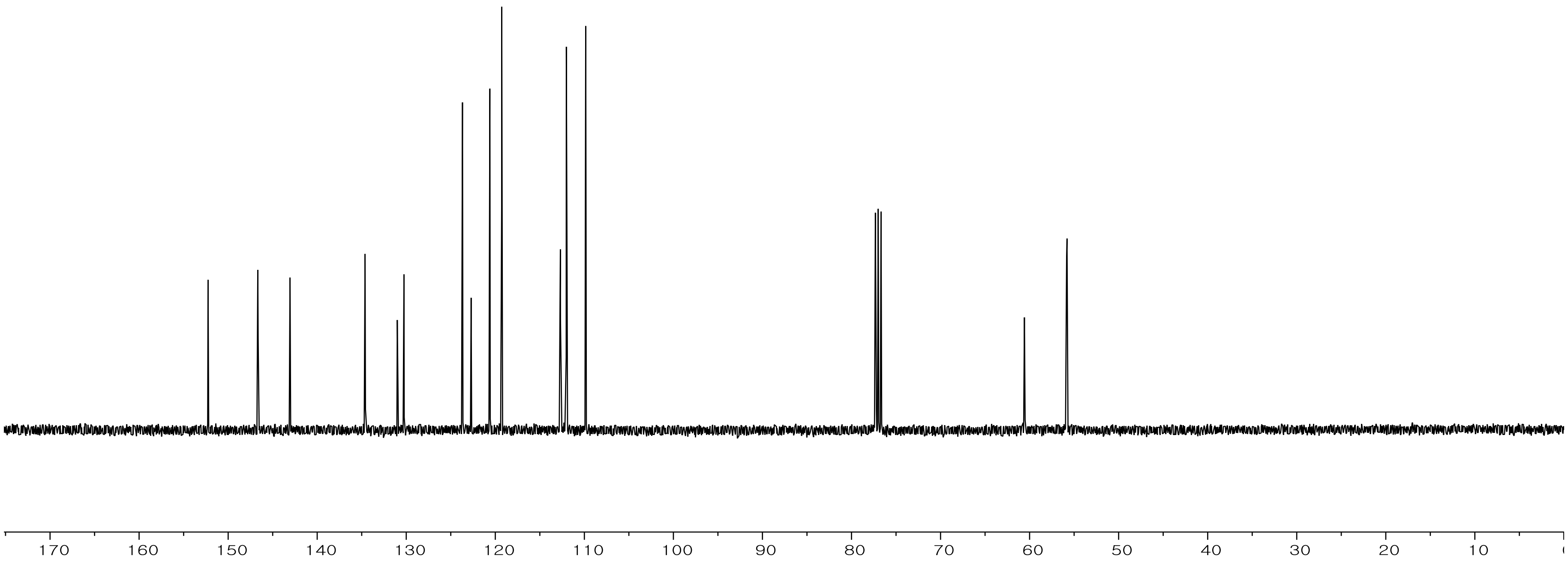

^{1}H NMR (400 MHz, C_6D_6)

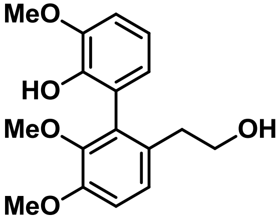

2k

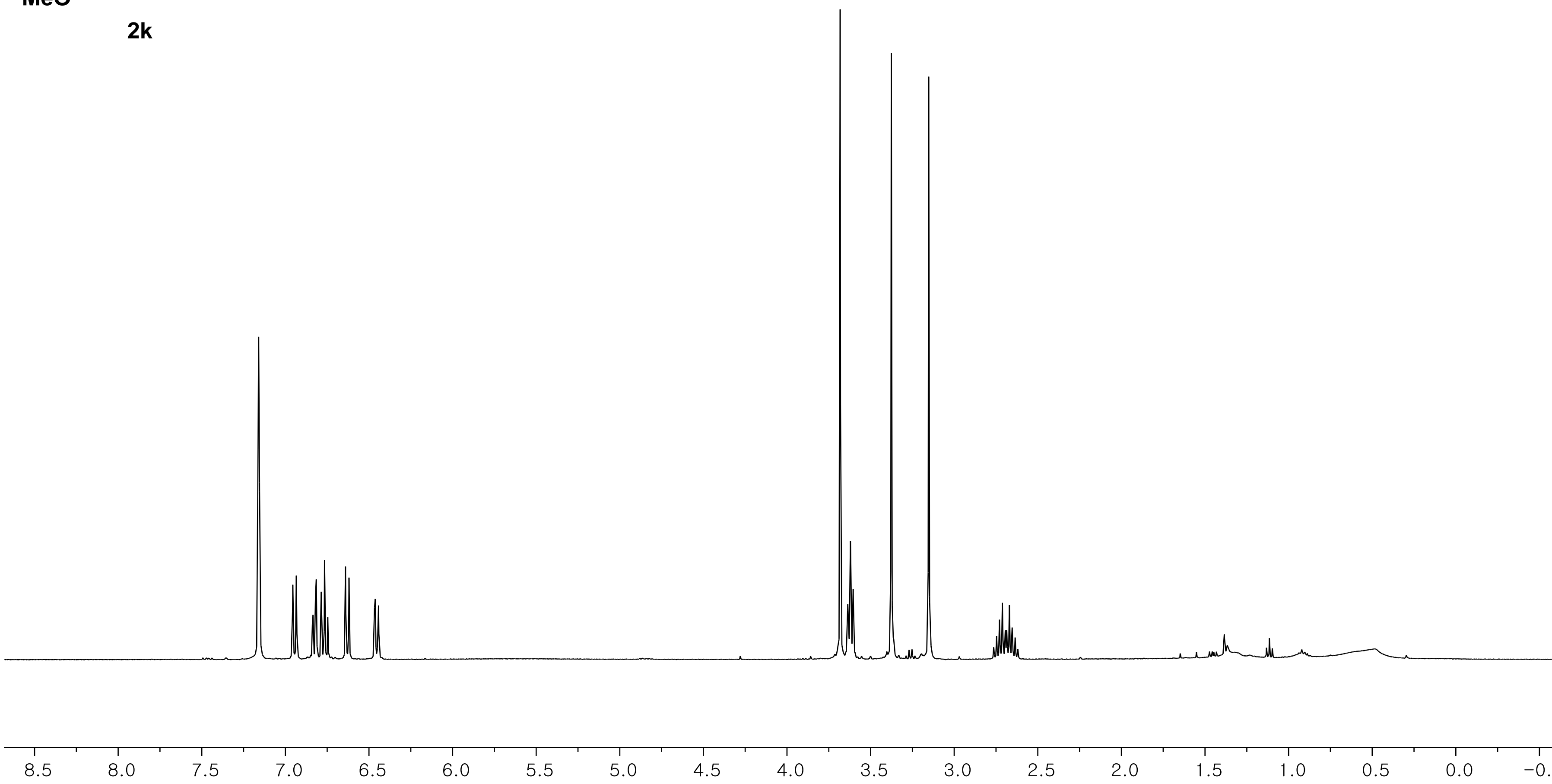

^{13}C NMR (101 MHz, $CDCl_3$)

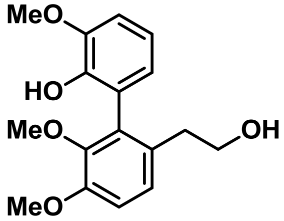

2k

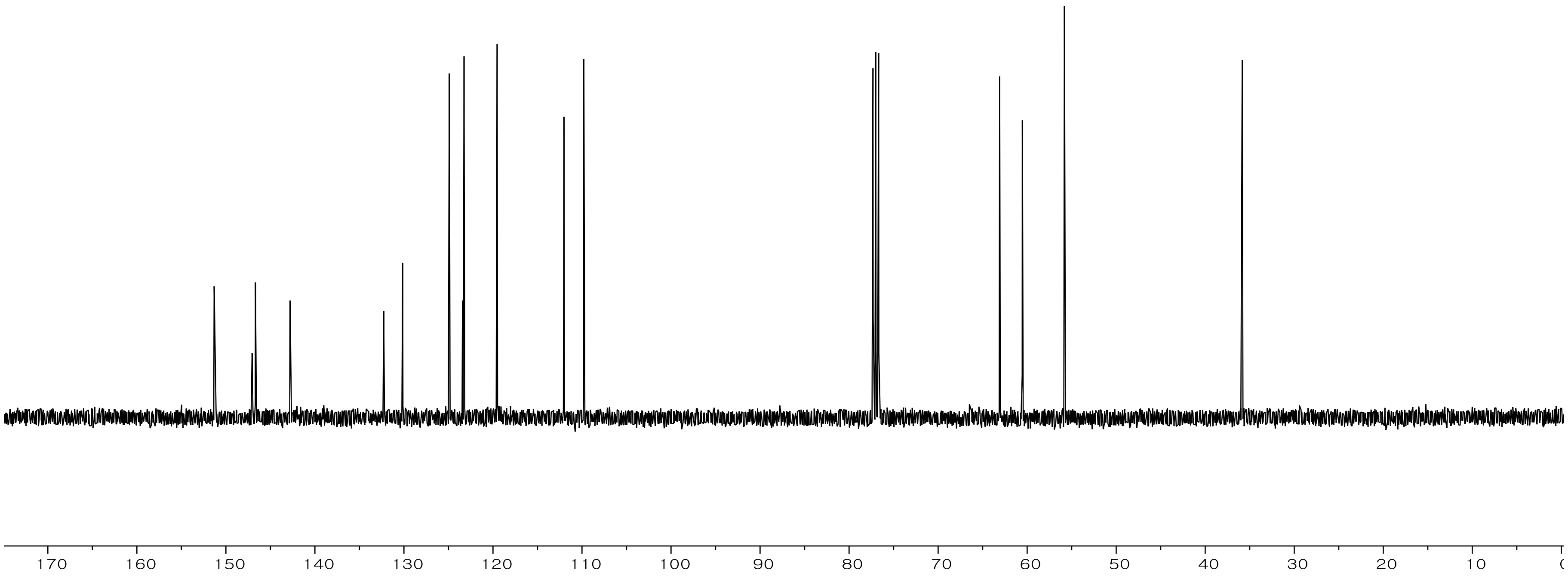

^{1}H NMR (400 MHz, $CDCl_3$)

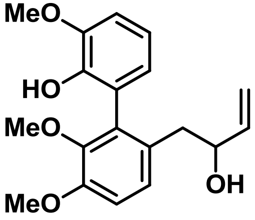

6g

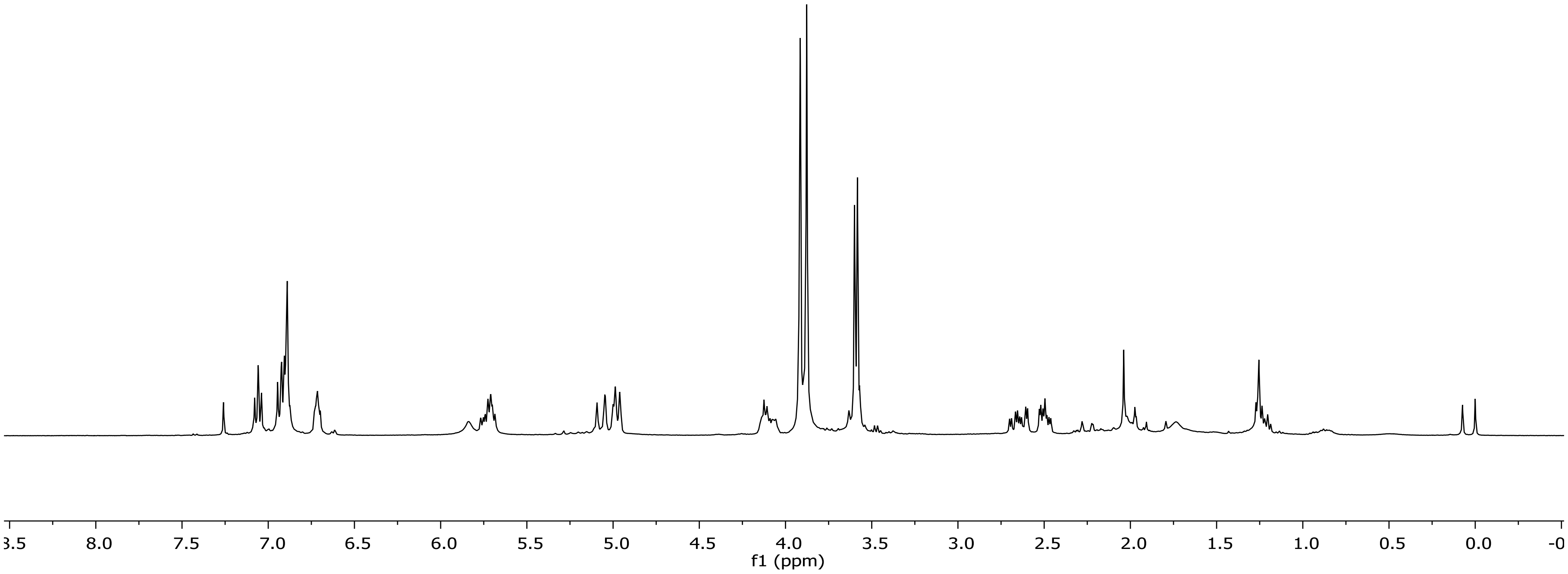

^{13}C NMR (101 MHz, $CDCl_3$)

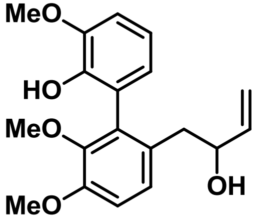

6g

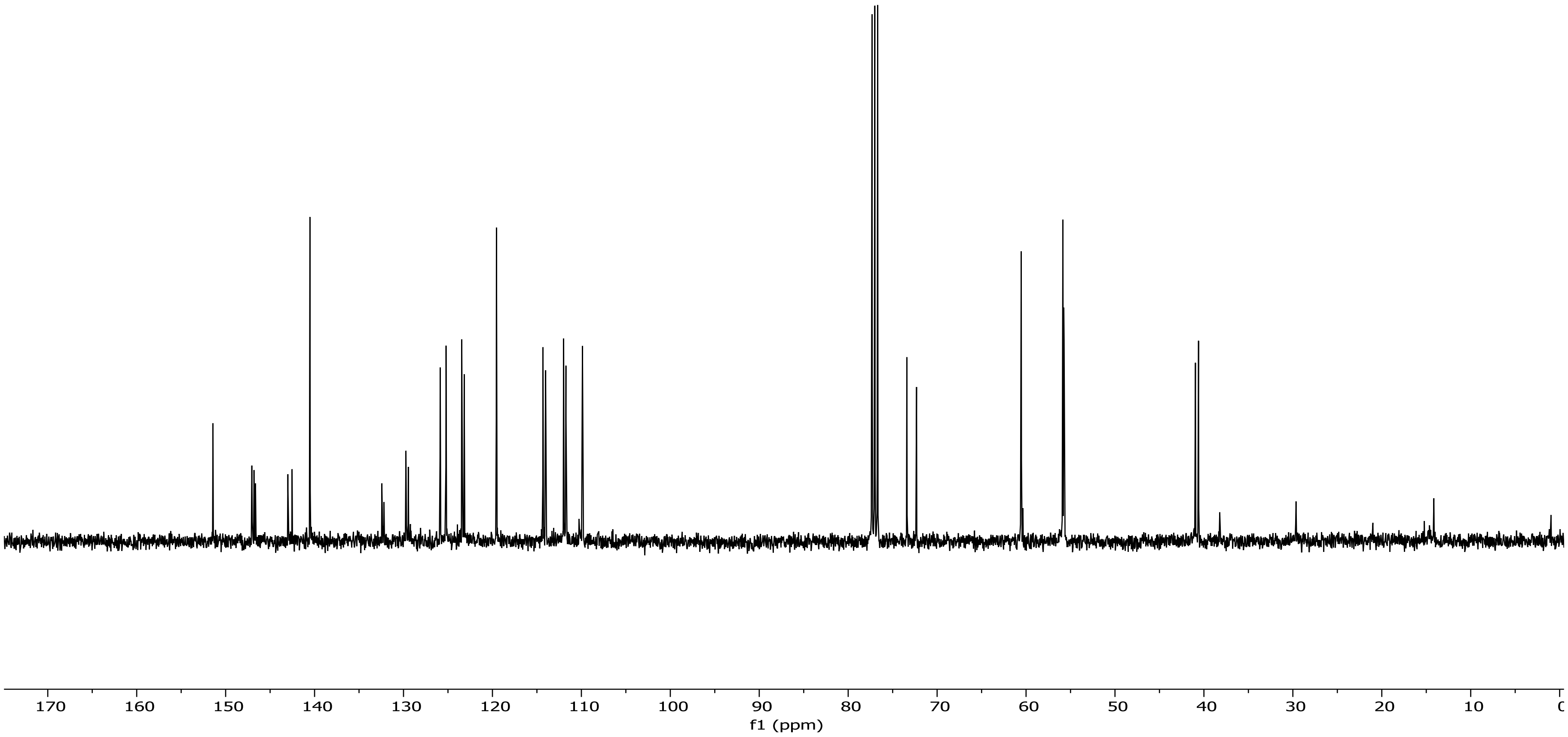

^{1}H NMR (400 MHz, $CDCl_3$)

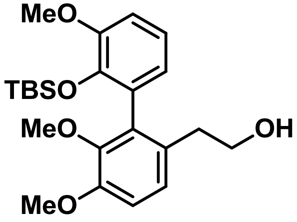

2n

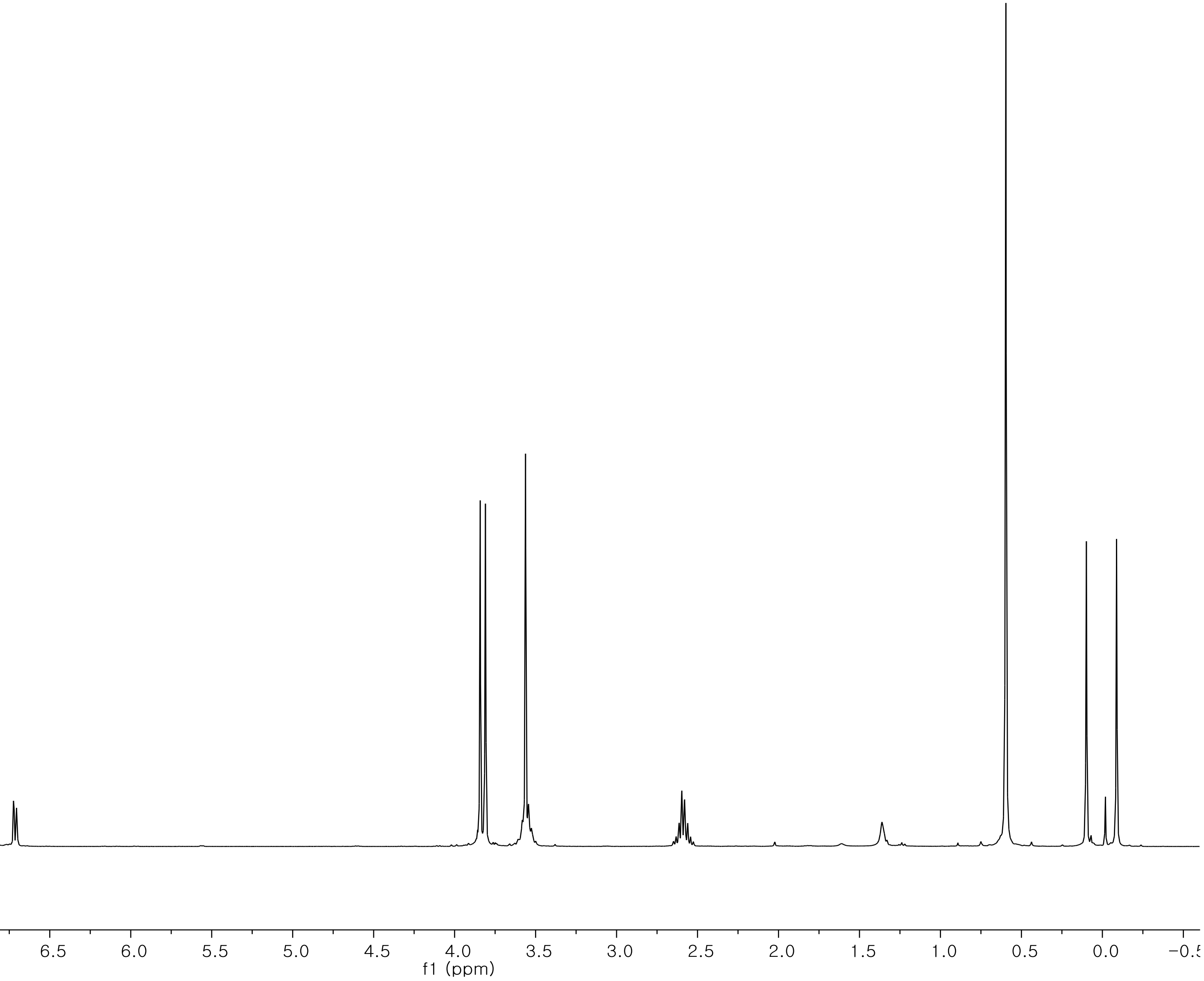

^{13}C NMR (101 MHz, $CDCl_3$)

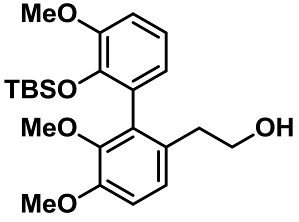

2n

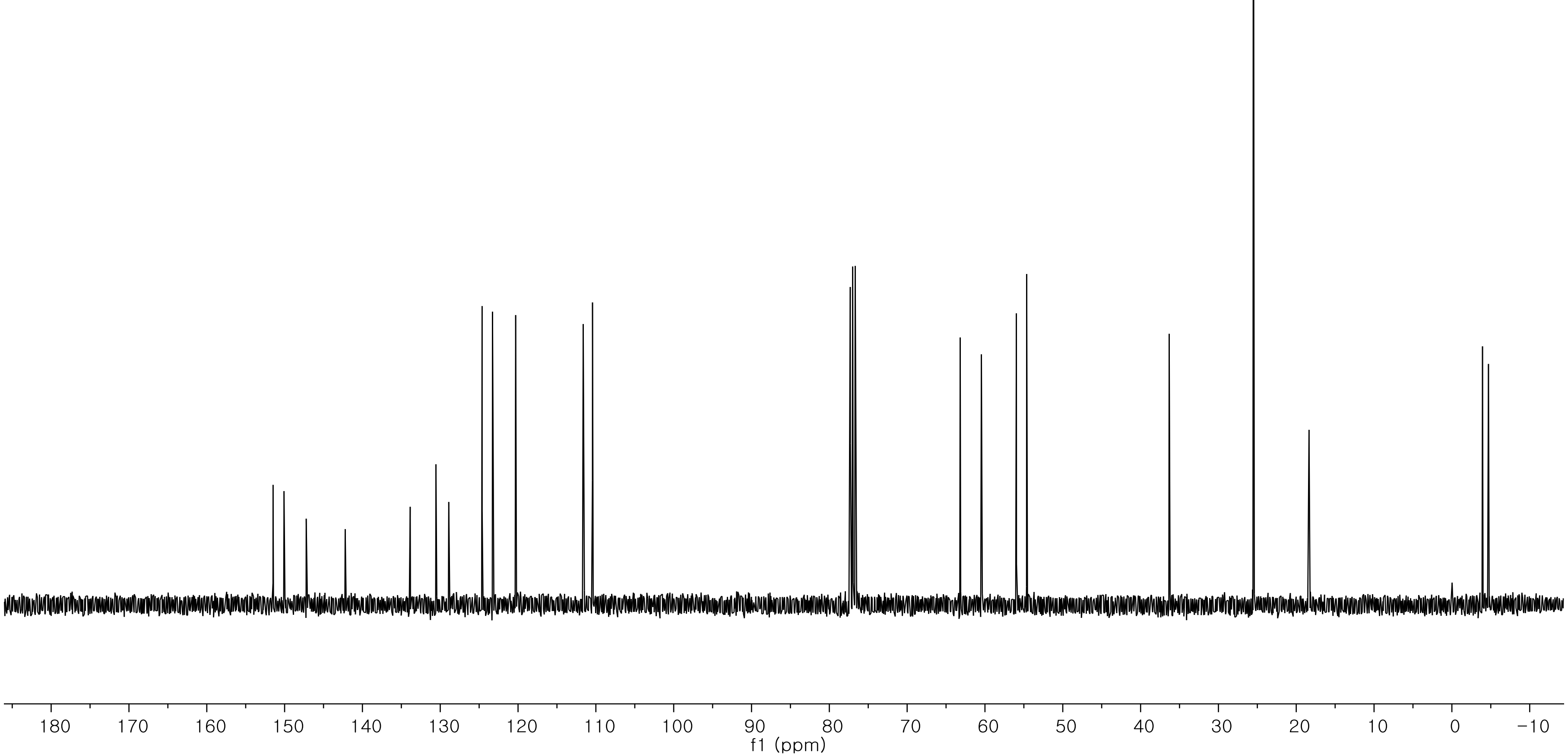

^{1}H NMR (400 MHz, $CDCl_3$)

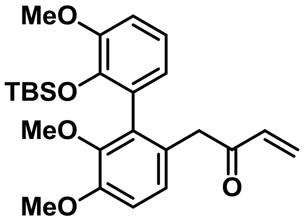

6i

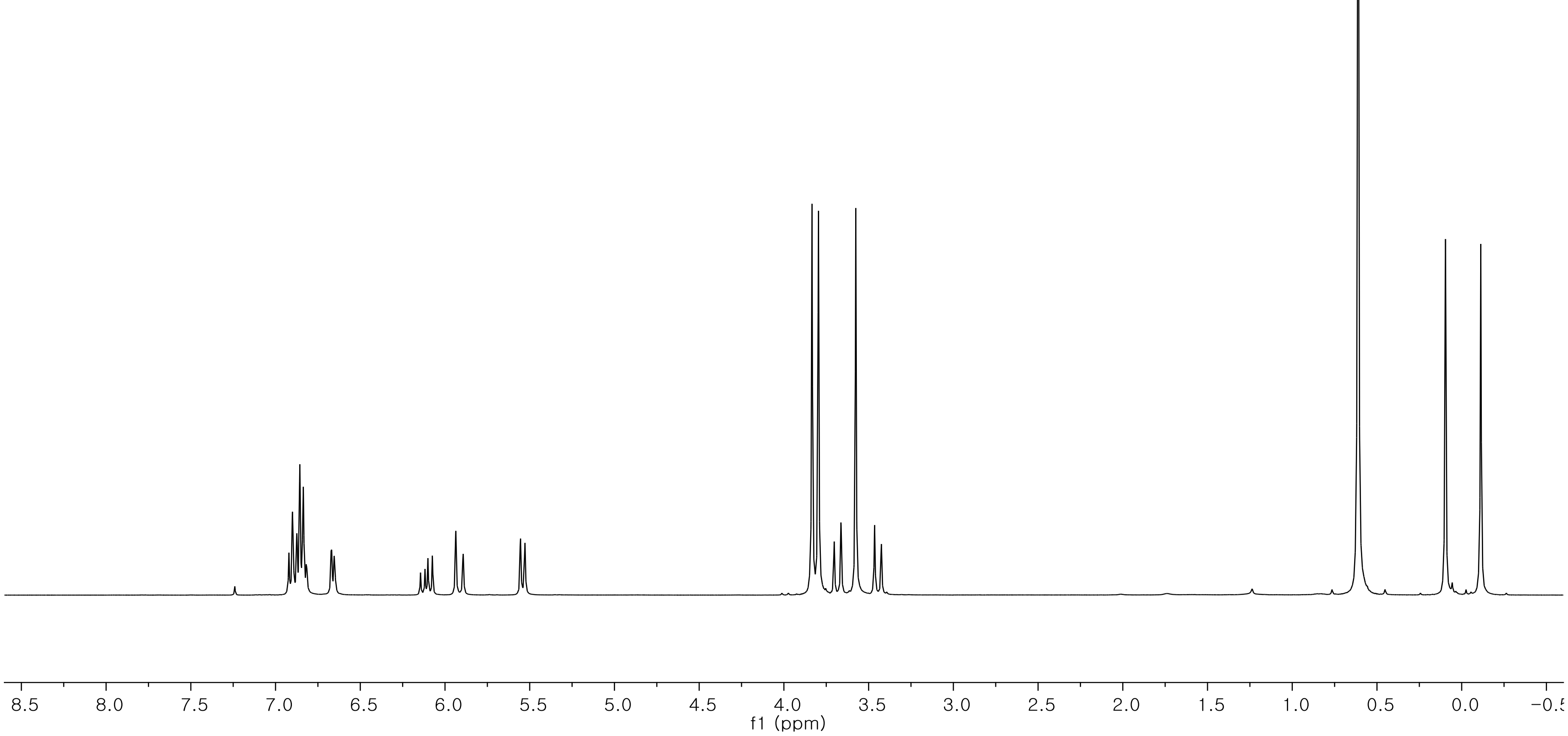

^{13}C NMR (101 MHz, $CDCl_3$)

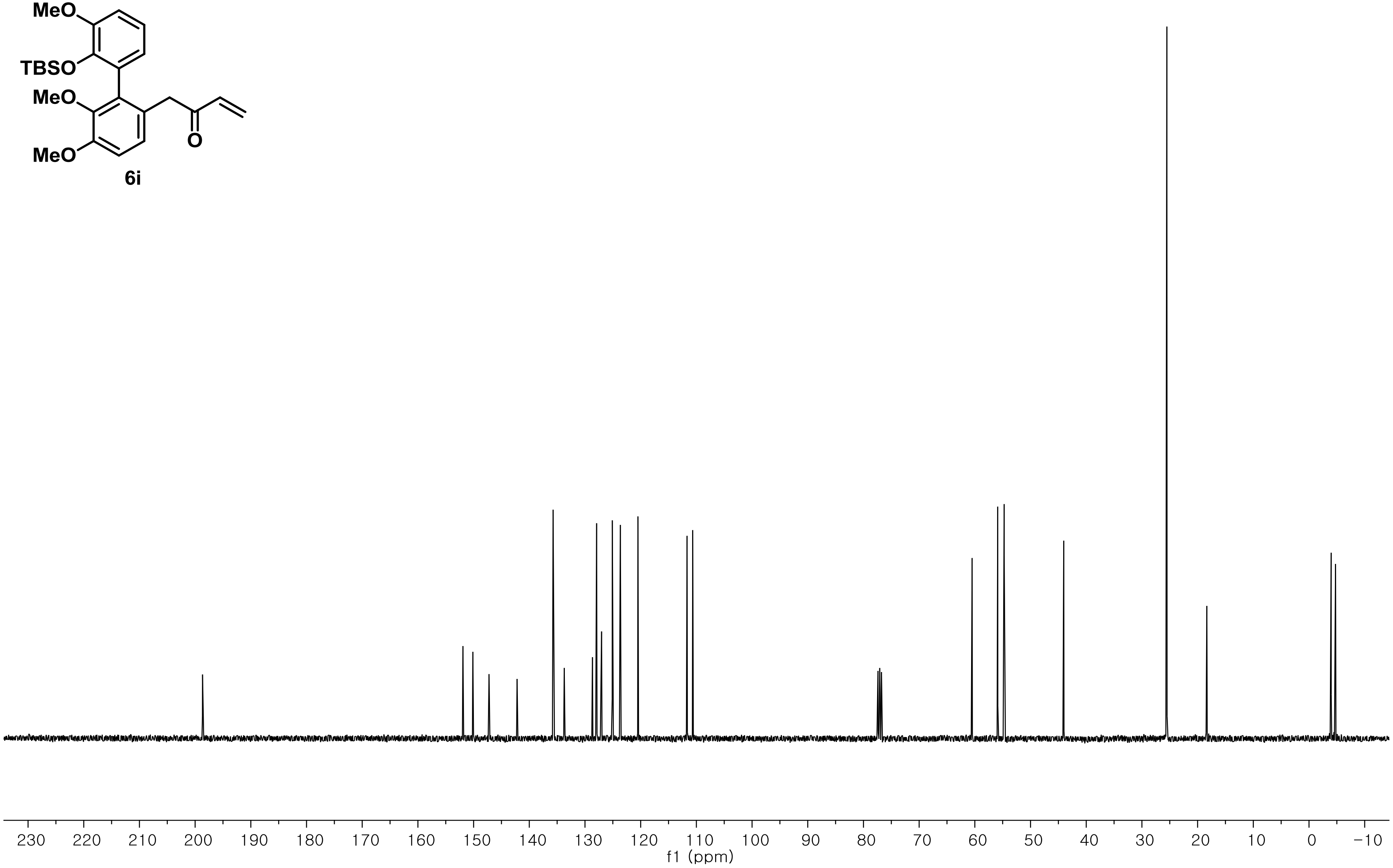

6i

^{1}H NMR (400 MHz, $CDCl_3$)

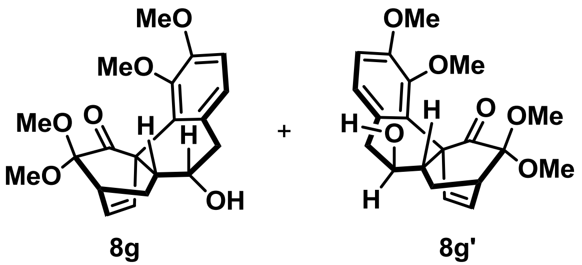

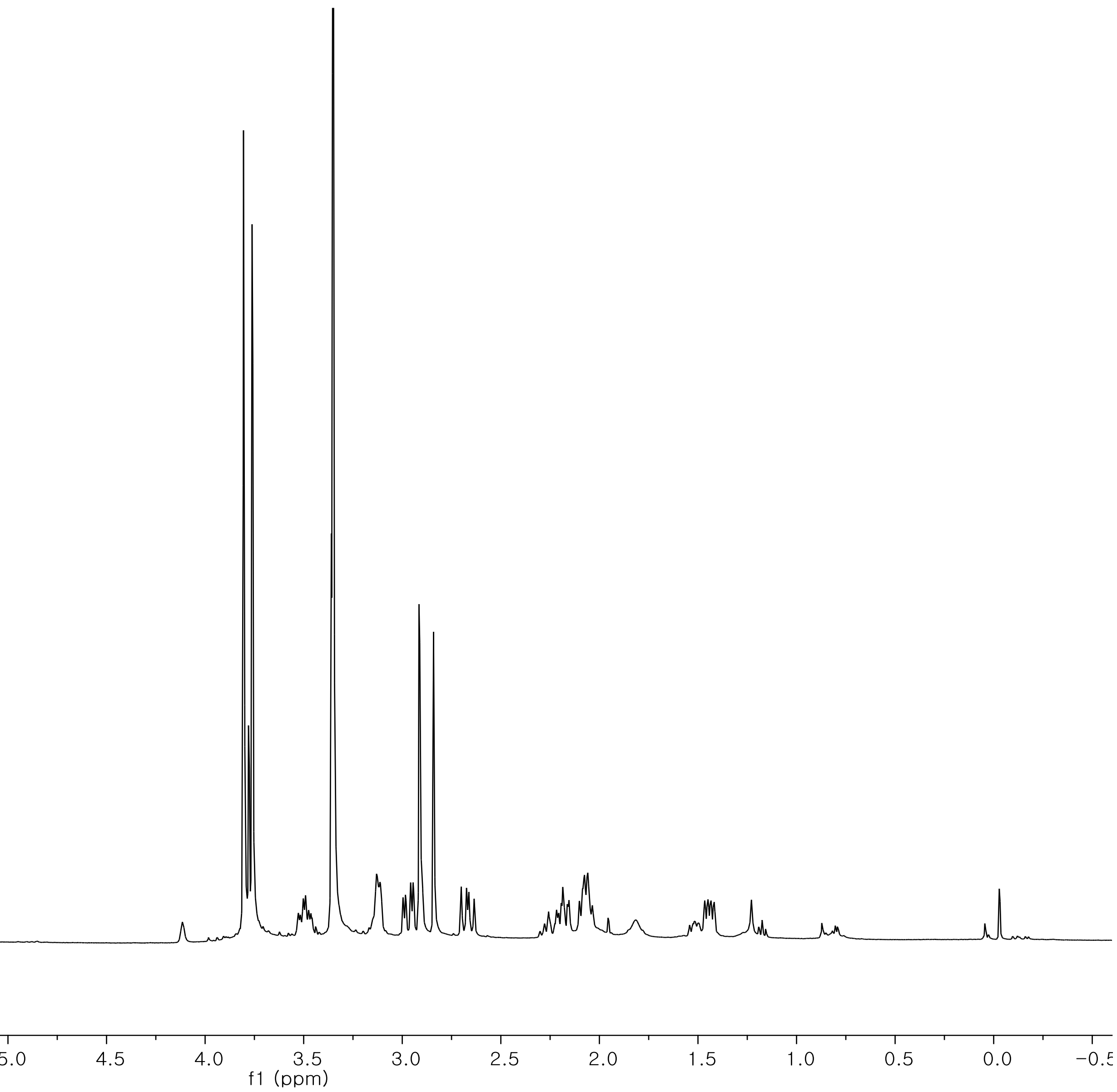

^{13}C NMR (101 MHz, $CDCl_3$)

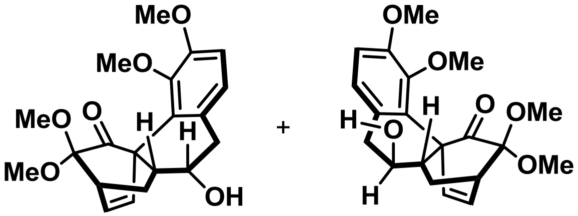

8g **8g'**

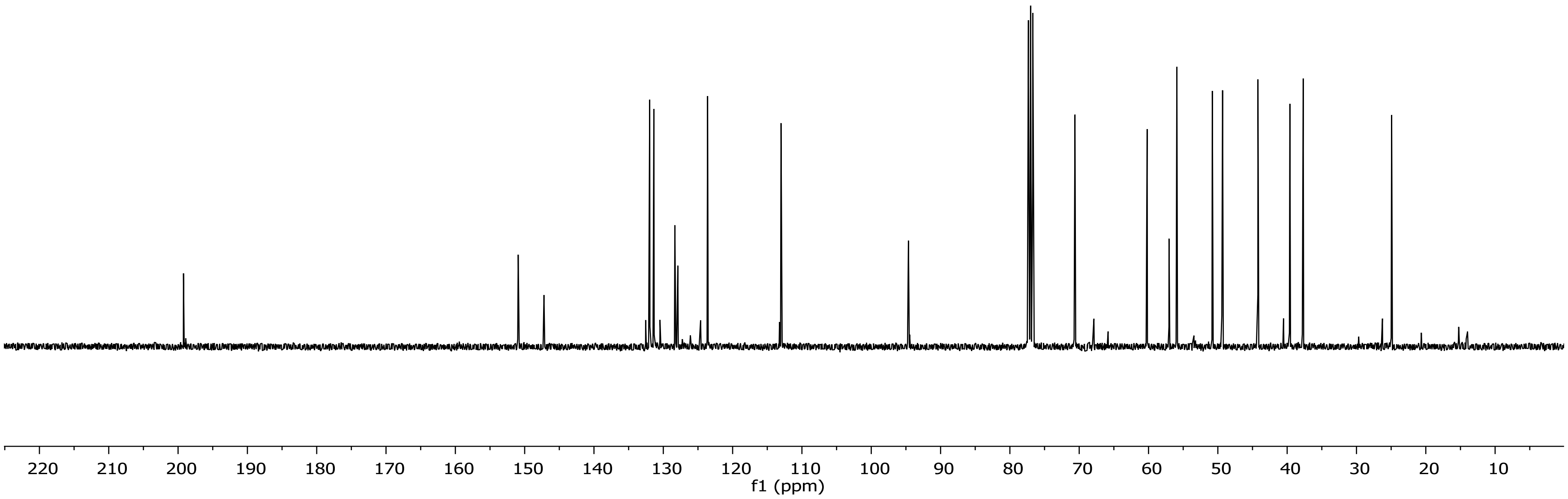

^{1}H NMR (400 MHz, $CDCl_3$)

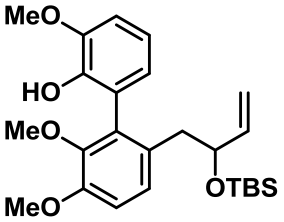

6h

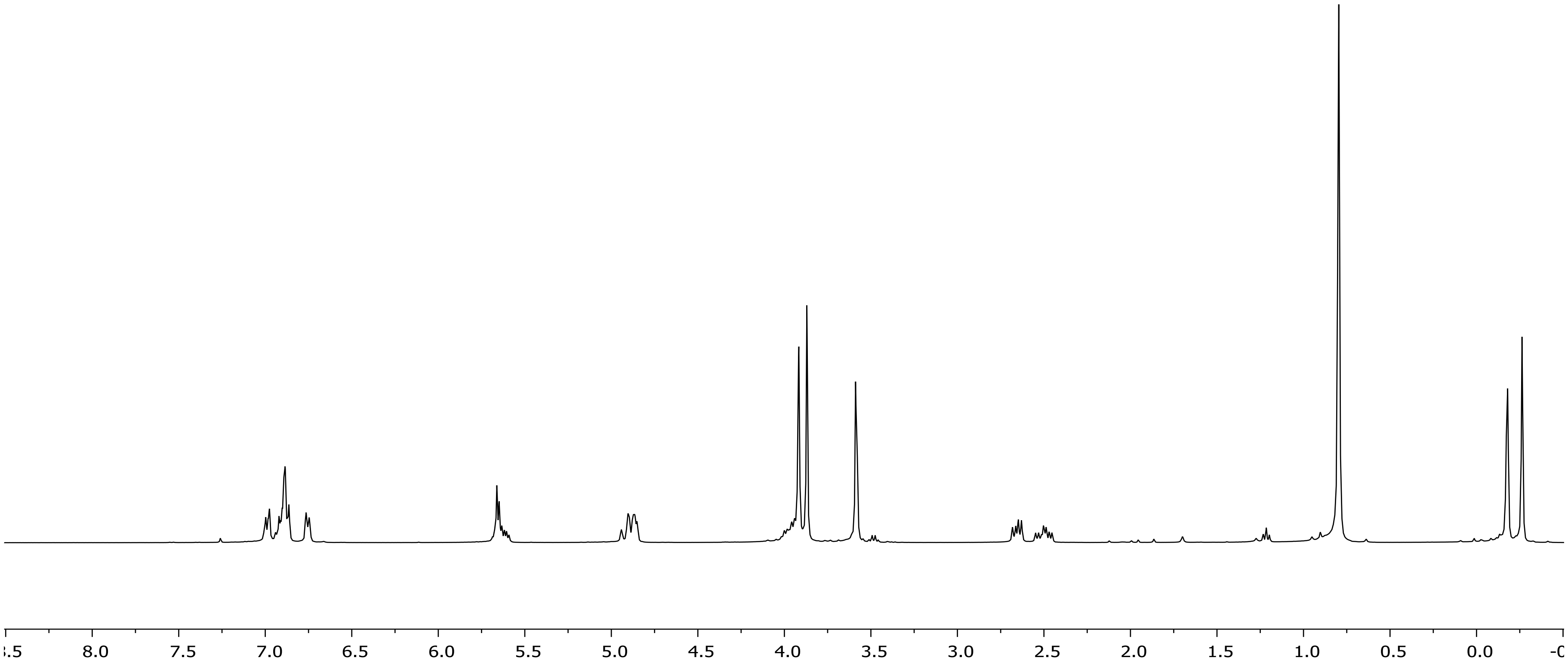

^{13}C NMR (101 MHz, $CDCl_3$)

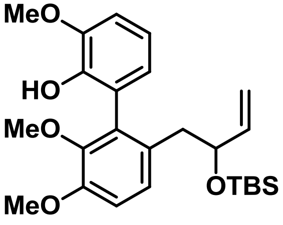

6h

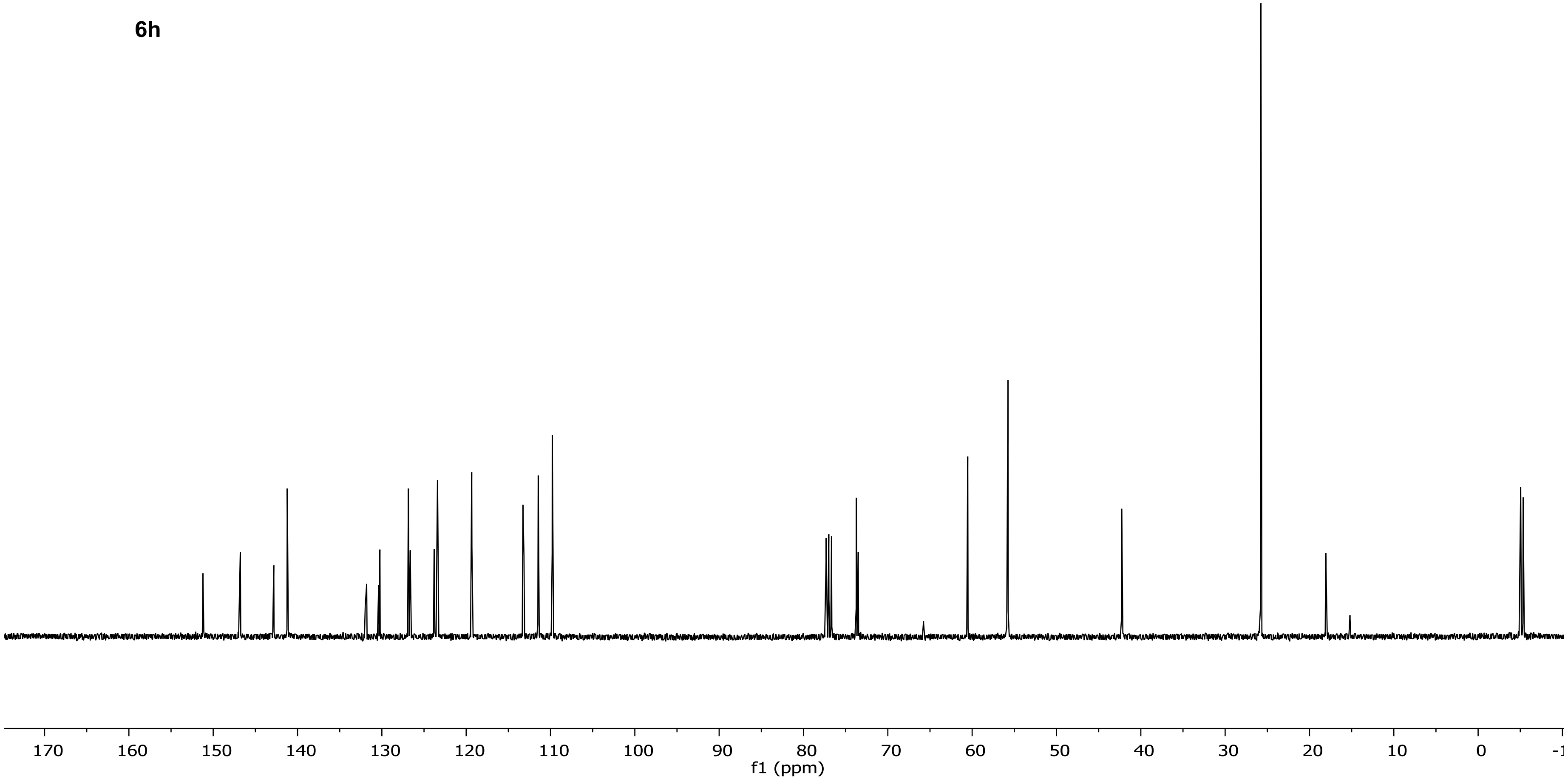

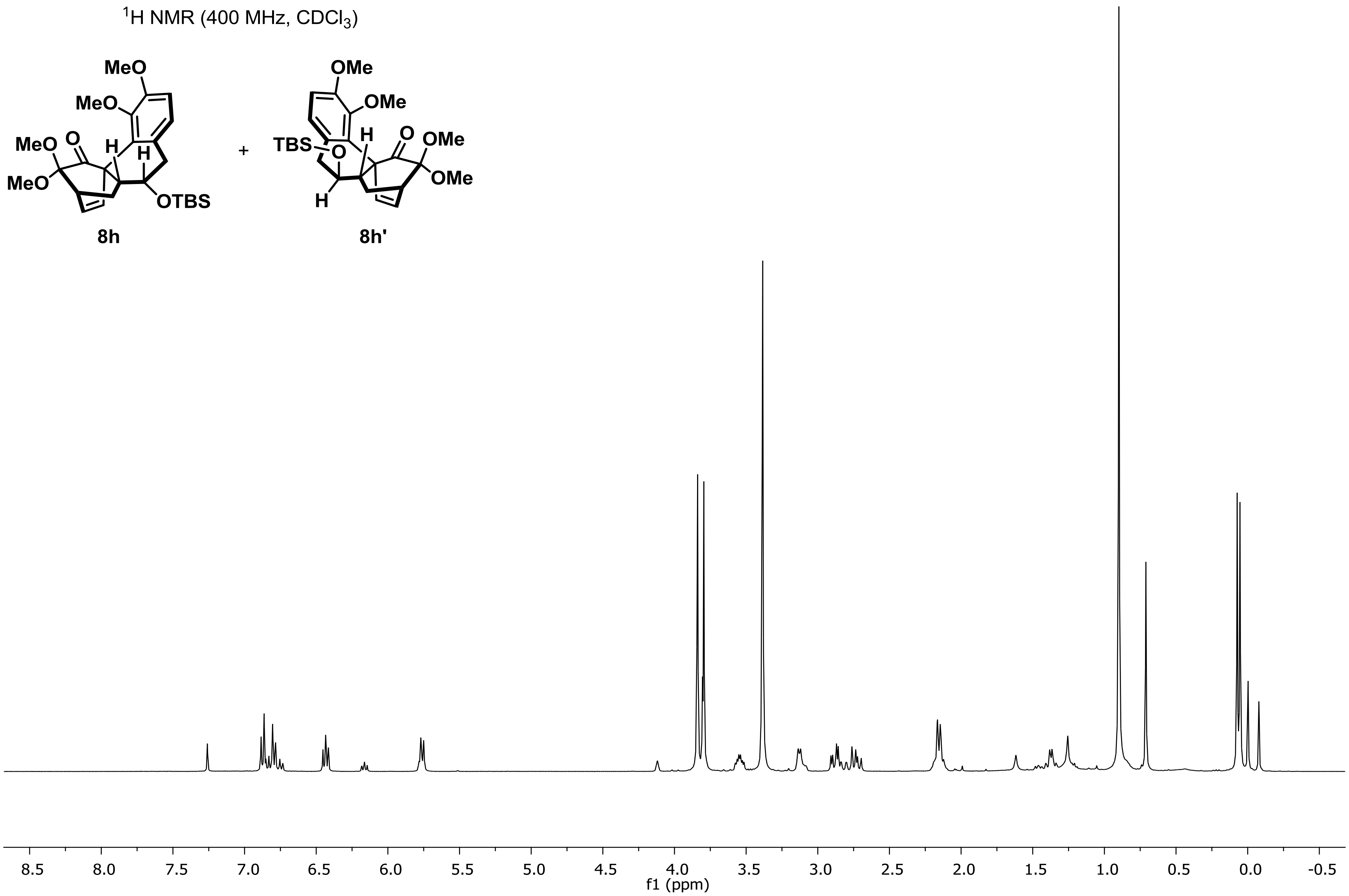

1H NMR (400 MHz, CDCl3)
MeO
MeO
MeO
MeO
H
H
OTBS
8h
+
OMe
OMe
TBS
O
H
H
O
OMe
OMe
8h'
8.5
8.0
7.5
7.0
6.5
6.0
5.5
5.0
4.5
4.0
3.5
3.0
2.5
2.0
1.5
1.0
0.5
0.0
-0.5
f1 (ppm)

^{13}C NMR (101 MHz, $CDCl_3$)

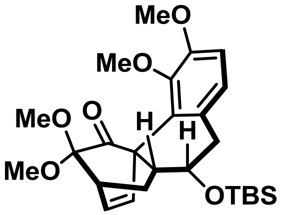

8h

+

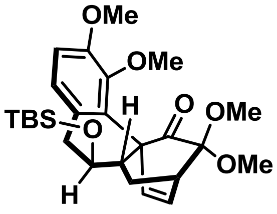

8h'

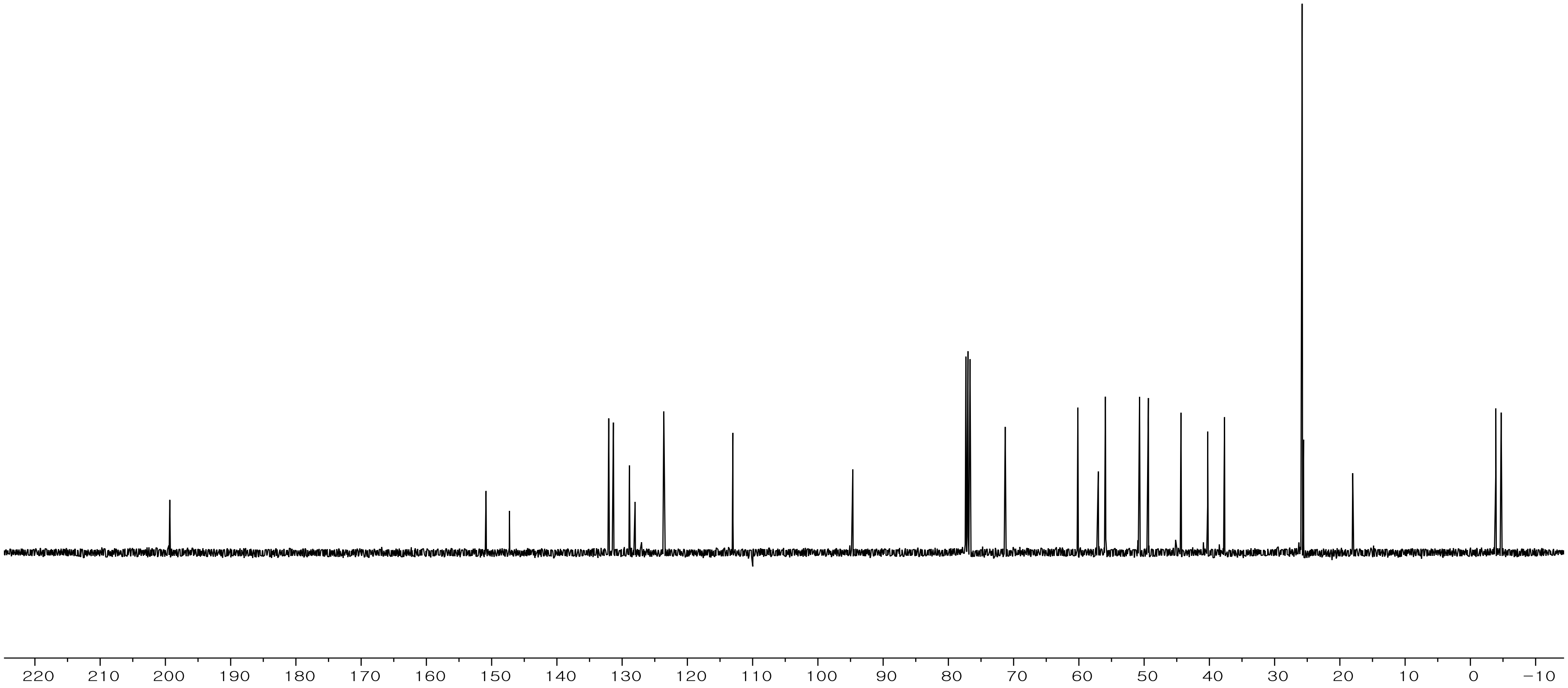

^{1}H NMR (400 MHz, $CDCl_3$)

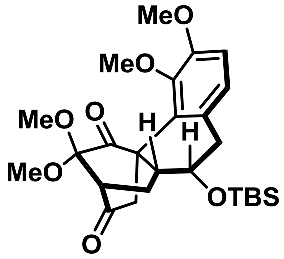

8j

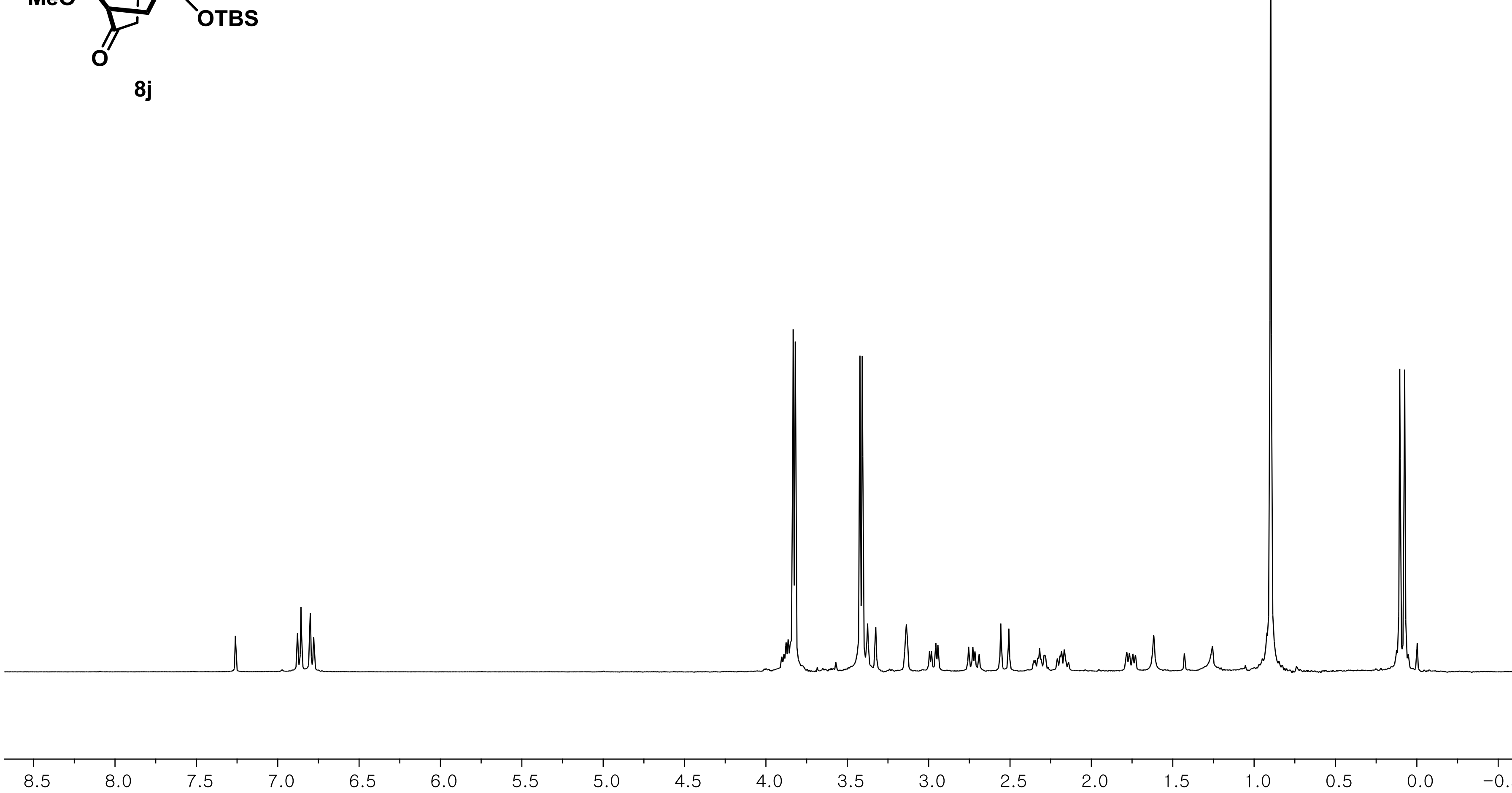

^{13}C NMR (101 MHz, $CDCl_3$)

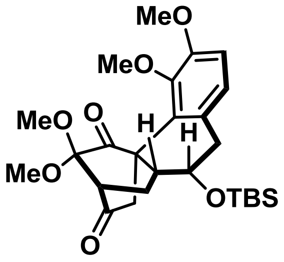

8j

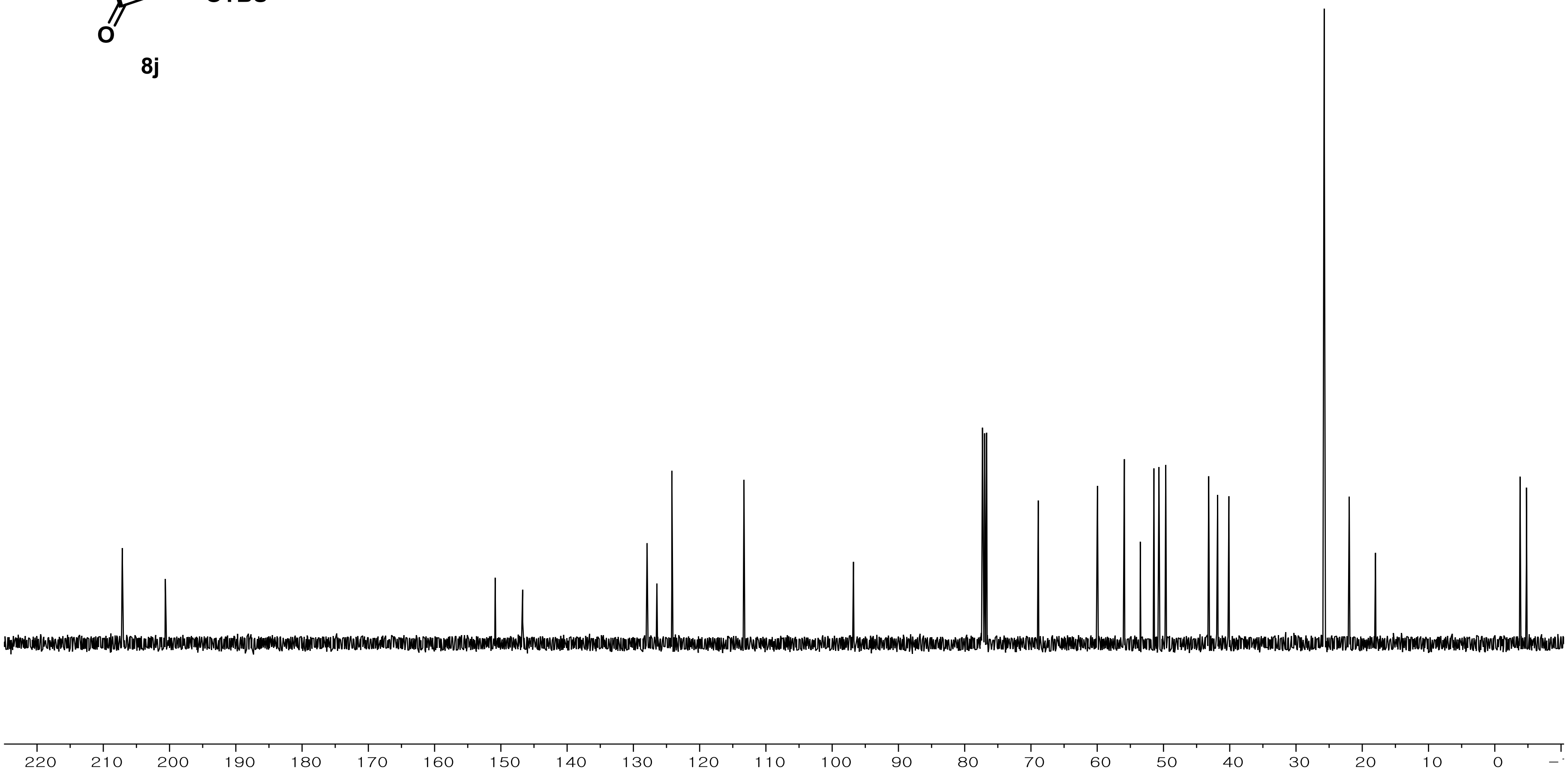

^{1}H NMR (400 MHz, $CDCl_3$)

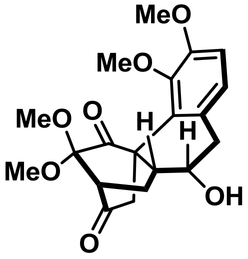

8k

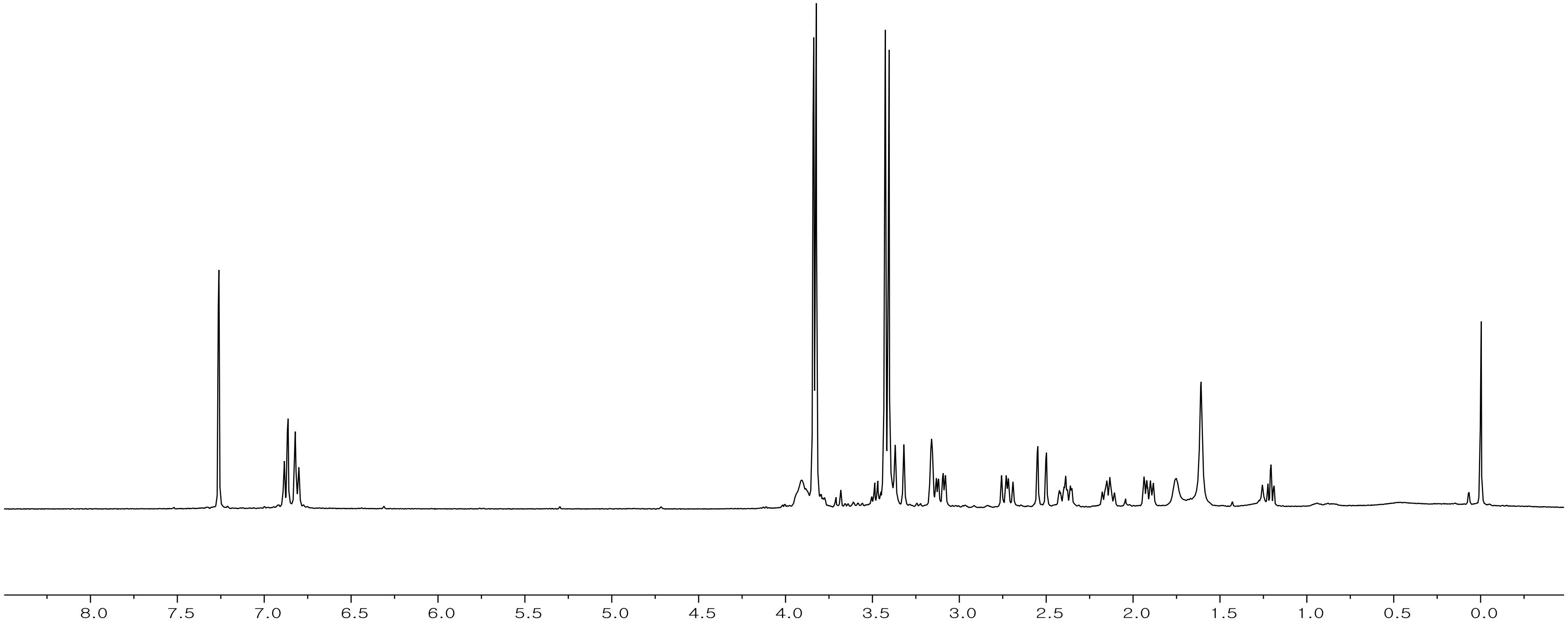

^{13}C NMR (101 MHz, $CDCl_3$)

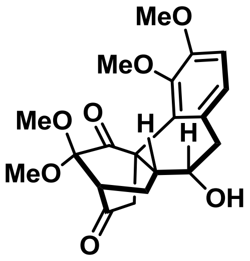

8k

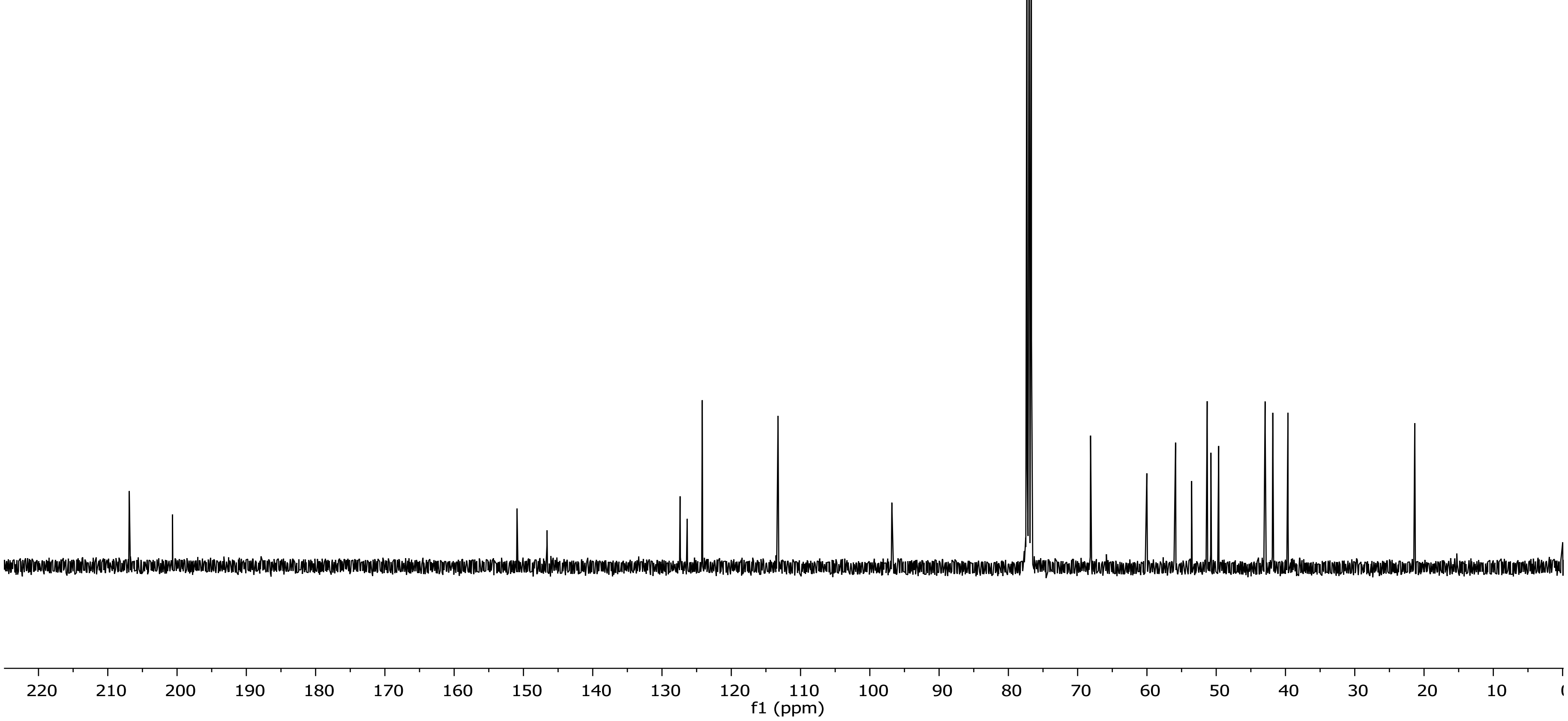

^{1}H NMR (400 MHz, $CDCl_3$)

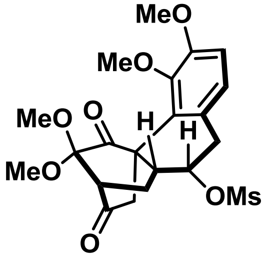

8l

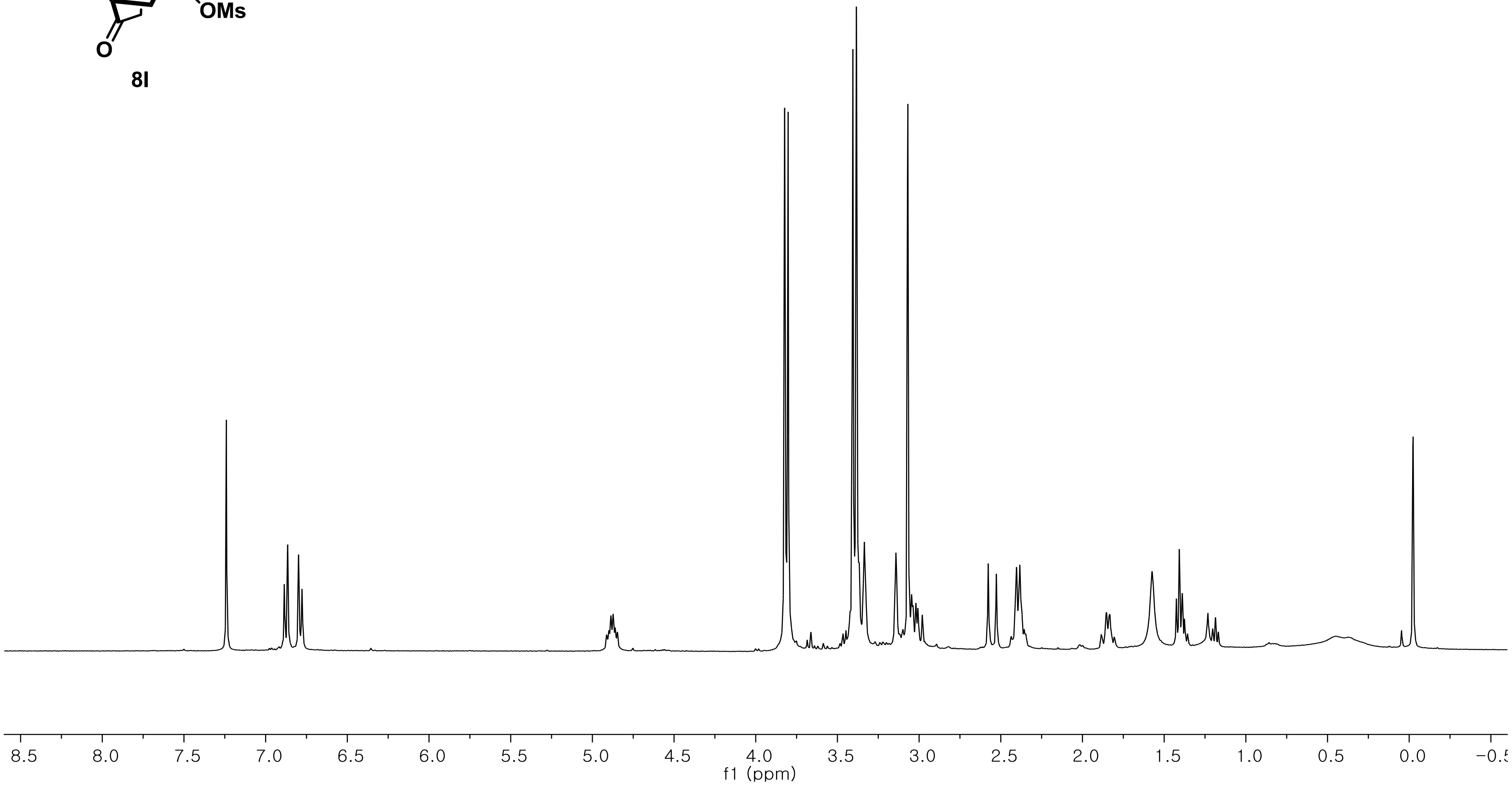

^{13}C NMR (101 MHz, $CDCl_3$)

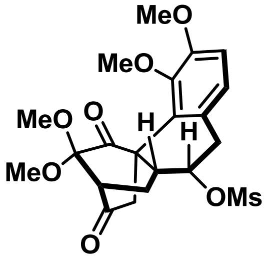

8l

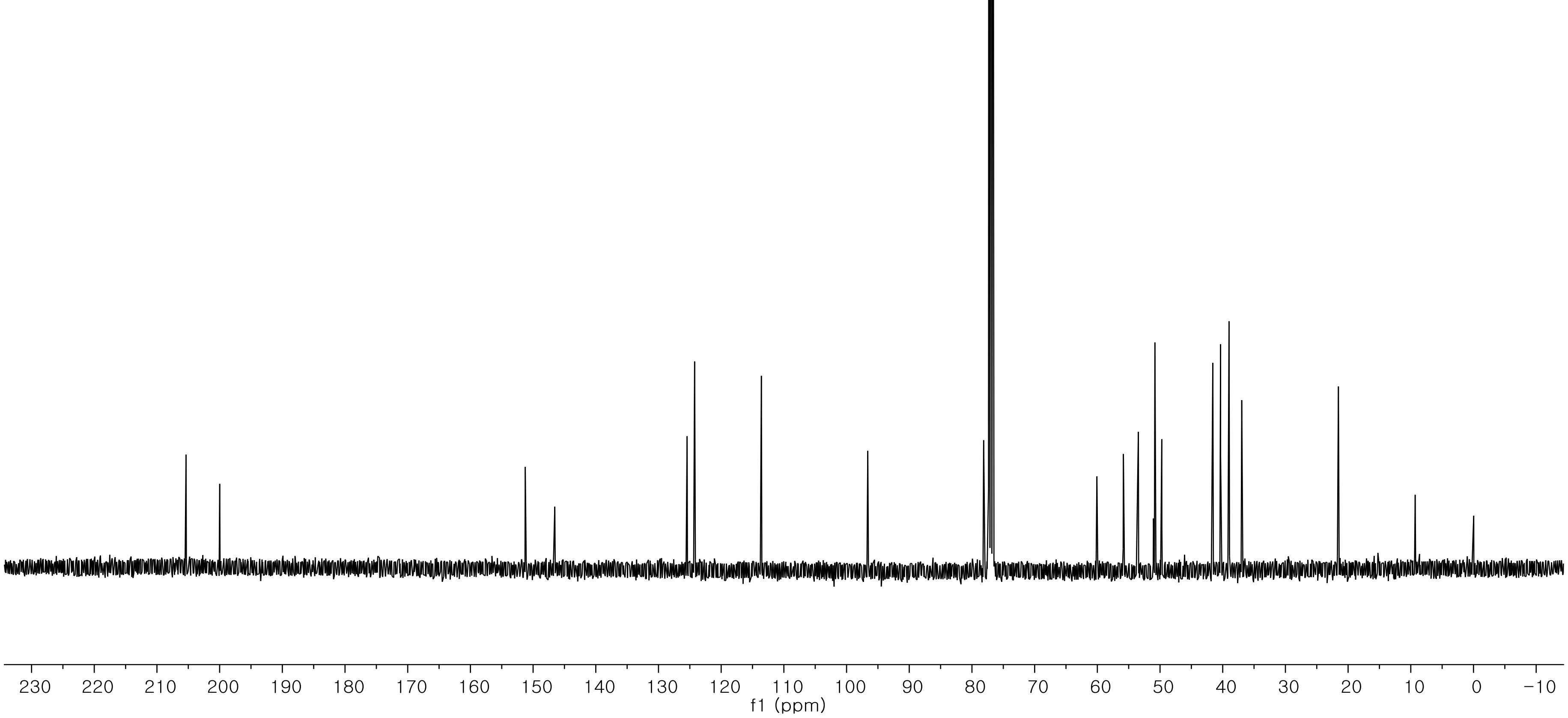

Supplement: Supplementary file 1 [file SC-008-C7SC03189K-s001.pdf]
